# *Pseudomonas savastanoi* Two-Component System RhpRS Switches between Virulence and Metabolism by Tuning Phosphorylation State and Sensing Nutritional Conditions

**DOI:** 10.1128/mBio.02838-18

**Published:** 2019-03-19

**Authors:** Yingpeng Xie, Xiaolong Shao, Yingchao Zhang, Jingui Liu, Tingting Wang, Weitong Zhang, Canfeng Hua, Xin Deng

**Affiliations:** a Department of Biomedical Sciences, City University of Hong Kong, Kowloon Tong, Hong Kong SAR, China; b Key Laboratory of Molecular Microbiology and Technology, Ministry of Education, TEDA Institute of Biological Sciences and Biotechnology, Nankai University, Tianjin, China; c Shenzhen Research Institute, City University of Hong Kong, Shenzhen, Guangdong, China; College of Veterinary Medicine, Cornell University

**Keywords:** *Pseudomonas savastanoi*, RhpRS, T3SS, two-component system

## Abstract

Pseudomonas savastanoi uses a type III secretion system (T3SS) to invade host plants. Our previous studies have demonstrated that a two-component system (TCS), RhpRS, enables P. savastanoi to coordinate the T3SS gene expression, which depends on the phosphorylation state of RhpR under different environmental conditions. Orthologues of RhpRS are distributed in a wide range of bacterial species, indicating a general regulatory mechanism. How RhpRS uses external signals and the phosphorylation state to exercise its regulatory functions remains unknown. We performed chromatin immunoprecipitation sequencing (ChIP-seq) assays to identify the specific binding sites of RhpR and RhpR^D70A^ in either King’s B medium (KB [a T3SS-inhibiting medium]) or minimal medium (MM [a T3SS-inducing medium]). We identified 125 KB-dependent binding sites and 188 phosphorylation-dependent binding sites of RhpR. In KB, RhpR directly and positively regulated cytochrome *c*_550_ production (via *ccmA*) and alcohol dehydrogenase activity (via *adhB*) but negatively regulated anthranilate synthase activity (via *trpG*) and protease activity (via *hemB*). In addition, phosphorylated RhpR (RhpR-P) directly and negatively regulated the T3SS (via *hrpR* and *hopR1*), swimming motility (via *flhA*), c-di-GMP levels (via PSPPH_2590), and biofilm formation (via *algD*). It positively regulated twitching motility (via *fimA*) and lipopolysaccharide production (via PSPPH_2653). Our transcriptome sequencing (RNA-seq) analyses identified 474 and 840 new genes that were regulated by RhpR in KB and MM, respectively. We showed nutrient-rich conditions allowed RhpR to directly regulate multiple metabolic pathways of *P. savastanoi* and phosphorylation enabled RhpR to specifically control virulence and the cell envelope. The action of RhpRS switched between virulence and regulation of multiple metabolic pathways by tuning its phosphorylation and sensing environmental signals in KB, respectively.

## Abstract (precis)

The plant pathogen Pseudomonas savastanoi invades host plants through a type III secretion system, which is strictly regulated by a two-component system called RhpRS. The orthologues of RhpRS are widely distributed in the bacterial kingdom. The master regulator RhpR specifically depends on the phosphorylation state to regulate the majority of the virulence-related genes. Under nutrient-rich conditions, it modulates many important metabolic pathways, which consist of one-fifth of the genome. We propose that RhpRS uses phosphorylation- and nutrition-dependent mechanisms to switch between regulating virulence and metabolism, and this functionality is widely conserved among bacterial species.

## INTRODUCTION

Pseudomonas savastanoi pv. *phaseolicola* (formerly named Pseudomonas syringae pv. *phaseolicola*) is a model plant-pathogenic bacterium and is widely considered the leading plant pathogen, causing deadly diseases and huge economic losses in agriculture worldwide (1). P. syringae relies on a needle-like type III secretion system (T3SS) to secret a group of T3SS effector proteins that facilitate infection (2). The T3SS is encoded by a cluster of hypersensitive response and pathogenicity (*hrp*) genes, which are capable of causing diseases on host plants and hypersensitivity reactions (HRs) on non-host plants (3). The regulation of the P. syringae T3SS is coordinated by a variety of environmental signals and host factors (4). The T3SS genes are expressed at modest levels in a rich medium like King's B medium (KB) but are induced rapidly in plants or in a nutrient-depleted minimal medium (MM) (5). Several plant-specific signals such as phenolic compounds and environmental conditions, including low temperature, low osmolarity, and high acidity, are responsible for the induction of the T3SS (5–8).

The transcription of *hrp* genes is regulated by a HrpRS-HrpL pathway (9). The *hrpRS* operon encodes two NtrC-family transcription factors, HrpR and HrpS, which carry an enhancer-binding motif and form a heterodimer that binds to the *hrpL* promoter (9). With the interaction of the sigma factor RpoN (σ^54^), the HrpRS heterodimer activates the transcription of *hrpL* under the T3SS-inducing conditions (9, 10). HrpL negatively regulates itself but activates various T3SS genes by specifically binding to an *hrp*-box sequence in promoter regions (11–13). Our recent study has shown that HrpS alone directly activates *hrpK1*, *hrpA2*, and *hopAJ1* independent of HrpL (14). The ATP-dependent protease Lon specifically recognizes and degrades HrpR but is negatively regulated by the T3SS genes via feedback control (15, 16). HrpV directly binds and represses the activity of HrpS to negatively regulate the T3SS, while HrpG removes HrpV from HrpS and works as an antirepressor (17, 18). The epiphytic trait regulator AefR positively regulates both the quorum-sensing T3SS and bacterial pathogenicity in host plants (19, 20). The Hrp pilus structural protein HrpA controls the transcription and/or RNA stability of *hrpRS* (21).

In addition, the expression of the *hrpRS* operon is regulated by at least two two-component systems (TCSs): GacAS and RhpRS (22–24). The mutation of the response regulator gene *gacA* severely compromises the T3SS by abolishing the expression of *hrpRS*, *rpoN*, and *hrpL* (24), suggesting that GacAS is located upstream of the T3SS regulatory cascade. However, the signaling and regulatory mechanisms are still elusive. Our previous work has identified RhpRS as a new TCS controlling the P. syringae T3SS gene expression (22). The expression of *rhpR* severely reduces expression of the T3SS genes, indicating that RhpR functions as the negative regulator of the T3SS (22). Our microarray analysis has shown that the regulons of RhpR are distinct when cultured in either KB or MM (25). These results indicate that RhpR can alter its role to modulate gene transcription in response to environmental changes.

We have shown that RhpS functions as an autokinase and has dual kinase/phosphatase activities on RhpR, thus acting as a switch for the T3SS (20). Phosphorylated RhpR (RhpR-P) specifically recognizes an inverted repeat (IR) element, GTATC-N_6_-GATAC, in the promoter regions of the *rhpRS* operon and other genes such as PSPTO_2767 to modulate their transcription (26). RhpR-P directly suppresses the T3SS cascade genes by repressing the promoter activity of *hrpR* and inducing *lon* (15, 16). A mutation of Asp70 to Ala in RhpR largely compromises the T3SS-repressing activity of RhpR and its interaction with the *lon* promoter (22). These results suggest that the phosphorylation state of RhpR is essential to its DNA binding affinity and the repression of the T3SS.

Although we have preliminarily characterized RhpRS, two key questions remain to be answered. How does RhpRS sense and respond to different nutrient environments? How does the phosphorylation state tune the functions of RhpR? Using chromatin immunoprecipitation sequencing (ChIP-seq) and transcriptome sequencing (RNA-seq) analyses, we identified specific RhpR binding regions that were phosphorylation or KB dependent and improved the characterization of the RhpRS regulon. We also characterized a group of KB-dependent and phosphorylation-dependent genes with biochemical and genetic assays. Our phenotypic experiments showed that RhpR directly and precisely regulated virulence and metabolic pathways under different conditions. The importance of the IR element was confirmed by an electrophoretic mobility shift assay (EMSA) *in vitro* and by luciferase gene (*lux*)-based reporter experiments *in vivo*. Overall, our findings suggest RhpR is a master regulator with distinct KB-dependent and phosphorylation-dependent mechanisms and provide new insights into the elusive signaling pathways of the P. savastanoi T3SS. We expect molecular mechanisms similar to those of RhpRS to be highly conserved in a wide range of bacterial species.

## RESULTS

### Orthologues of RhpRS were distributed in a wide range of bacterial species.

**FIG 1:**
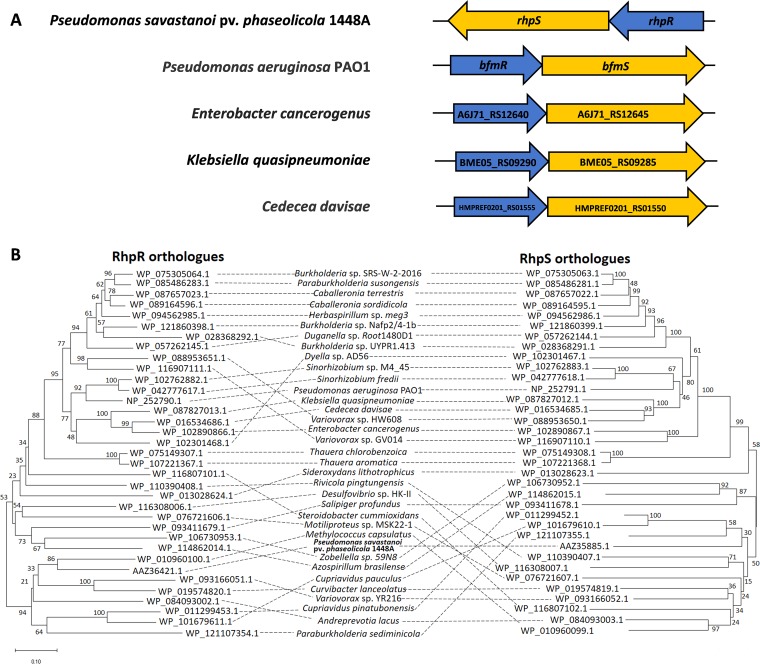
Prevalence of RhpRS orthologues. Synteny analyses and phylogenetic tree for widely distributed RhpRS orthologues. (A) Genetic organization of the RhpRS TCS in *P. savastanoi* 1448A and 4 human pathogens. The corresponding response regulator genes are depicted in blue, the histidine kinases are depicted in yellow, and the direction of the arrow represents the direction of transcription. (B) Phylogenetic tree of RhpR and RhpS. Thirty-five TCSs from 21 genera were included in the phylogenetic tree. The phylogenetic relations were inferred using the neighbor-joining method, the bootstrap values are shown next to the branches, and evolutionary distances were computed using the Poisson correction method. Analyses were performed with MEGA7 software.

The TCS RhpRS is a key regulator that switches the induction of the T3SS in P. syringae (22). To explore whether RhpRS was widely distributed among bacteria, we searched for orthologues in the National Center for Biotechnology Information (NCBI) database and sorted the results by identity. As shown in Fig. 1A, a syntenic analysis for RhpRS orthologues in other species revealed that the response regulators and their cognate kinase genes were located in the same operon, which is a typical feature of a TCS. The top 35 TCS orthologues of RhpRS were further subjected to protein sequence alignment and phylogenetic analysis. Using the Constraint-based Multiple Alignment Tool (COBALT), a group of conserved domains were identified in the RhpRS orthologues. For example, all 35 RhpR orthologues had a highly conserved receiver domain and DNA binding domain, while all 35 RhpS orthologues had a conserved transmembrane domain, phosphorylation domain, and ATP-binding domain. These results indicate a similar pattern of stimulus-response coupling mechanisms across the RhpRS orthologues (see Fig. S1A and B in the supplemental material). The phylogenetic trees showed that the RhpRS orthologues were widely found in alphaproteobacteria, gammaproteobacteria, betaproteobacteria, and sigmaproteobacteria (Fig. 1B). The RhpRS orthologues were well distributed in a wide spectrum of species in the bacterial kingdom, suggesting that the orthologues likely originated from a common descent and have similar functions.

10.1128/mBio.02838-18.1 FIG S1 Protein sequence alignments of 35 RhpR and RhpS orthologues. RhpR and RhpS orthologues have highly conserved functional domains. (A) Multiple-sequence alignment of RhpR and (B) multiple-sequence alignment of RhpS among 35 RhpRS orthologues were performed. Red indicates highly conserved sequences, and blue indicates less-conserved ones. Two-way arrows indicate the function of conserved domains. The alignments were generated using the Constraint-based Multiple Alignment Tool (COBALT; https://www.ncbi.nlm.nih.gov/tools/cobalt/cobalt.cgi?CMD=Web). Download FIG S1, TIF file, 2.1 MB. Copyright © 2019 Xie et al. 2019 Xie et al. This content is distributed under the terms of the Creative Commons Attribution 4.0 International license.

### Phosphorylation- and KB-dependent RhpR binding regions in the *P. syringae* genome.

**FIG 2:**
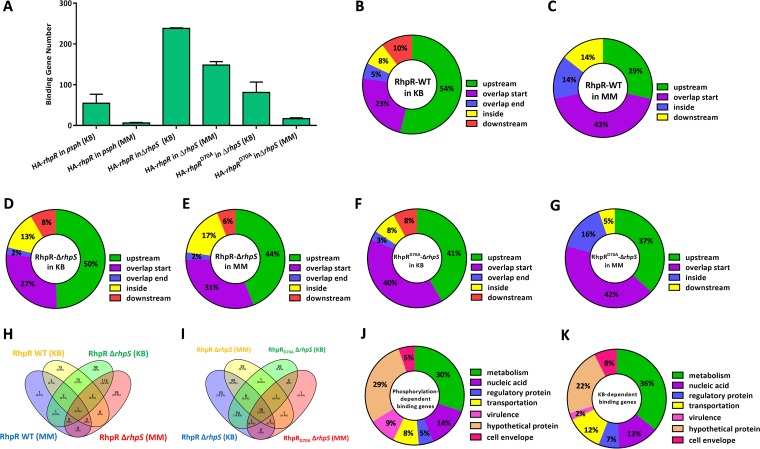
Genome-wide analysis of the KB- or phosphorylation-dependent RhpR-binding regions by ChIP-seq. ChIP-seq reveals *in vivo* binding sites of RhpR. (A) The numbers of RhpR or RhpR^D70A^ binding peaks under different culture conditions are shown. The ChIP-seq analyses were repeated twice. (B to G) The positions of the RhpR or RhpR^D70A^ binding peaks are represented in a pie chart. (H) A Venn diagram shows the comparisons of the RhpR-binding genes in the wild-type or Δ*rhpS* strain cultured in a different medium. Purple represents RhpR binding sites in the wild-type strain cultured in MM, yellow represents RhpR binding sites in the wild-type strain cultured in KB, green represents RhpR binding sites in the Δ*rhpS* strain cultured in KB, and pink represents RhpR binding sites in the Δ*rhpS* strain cultured in MM. (I) A Venn diagram shows the comparisons of the RhpR or RhpR^D70A^ binding genes in the wild-type or Δ*rhpS* strain cultured in different media. Purple represents RhpR binding sites in the wild-type strain cultured in MM, yellow represents RhpR binding sites in the wild-type strain cultured in KB, green represents RhpR^D70A^ binding sites in the Δ*rhpS* strain cultured in KB, and red represents RhpR^D70A^ binding sites in the Δ*rhpS* strain cultured in MM. **(**J and K**)** The pie charts display the percentage of KB- or phosphorylation-dependent RhpR targets with functional categories based on the *Pseudomonas* database (http://pseudomonas.com).

Our previous ChIP-seq and microarray analyses revealed that RhpR binds to 167 loci and regulates more than 900 genes in *P. savastanoi* (25). However, how RhpR relies on external signals and phosphorylation to exercise its regulatory functions remains elusive. To this end, we tried to identify the binding sites of RhpR and RhpR^D70A^ (Asp70 substituted by Ala) in KB or MM medium by ChIP-seq analysis. We performed six sets of ChIP-seq samples: (i) RhpR in the wild-type strain in KB, (ii) RhpR in the wild-type strain in MM, (iii) RhpR in the Δ*rhpS* strain in KB, (iv) RhpR in the Δ*rhpS* strain in MM, (v) RhpR^D70A^ in the Δ*rhpS* strain in KB, and (vi) RhpR^D70A^ in the Δ*rhpS* strain in MM (see Table S3A to F in the supplemental material). As shown in Fig. 2A, RhpR had 140 and 136 more binding loci than RhpR^D70A^ in KB and MM, respectively. This finding demonstrates that the phosphorylation of RhpR was important to its regulatory role. In both the wild-type and Δ*rhpS* strains, RhpR had 60 more binding sites when bacteria were grown in KB than in MM, indicating that RhpR had more regulatory functions under nutrient-rich conditions (Fig. 2A). More than 70% of the RhpR or RhpR^D70A^ binding loci were located upstream of or overlapping the start regions (Fig. 2B to G), indicating the potential regulatory functions of RhpR on these genes. Our results suggest that RhpR is a global regulator with both phosphorylation-dependent and KB-dependent functions in *P. savastanoi*.

To further characterize the specific phosphorylation- or KB-dependent binding sites of RhpR, we determined and compared binding peaks between RhpR and RhpR^D70A^. We identified 188 phosphorylation-dependent binding sites of RhpR (Fig. 2H and Table S3G). Similarly, by analyzing the RhpR binding sites in KB and MM, we identified 125 KB-dependent RhpR binding sites (Fig. 2I and Table S3H). Among the phosphorylation-dependent binding genes, 44% were associated with nucleic acid biosynthesis and metabolism, and 9% were associated with virulence and the cell envelope, such as *algD*, *fimA*, *fliF*, *flgF*, *flhA*, and *fliN* (Fig. 2J). However, only two KB-dependent RhpR binding regions were located on the virulence-related genes *fleS* and *flgA* (Fig. 2K). These results indicate that the phosphorylation status and KB medium were two important factors affecting the regulatory functions of RhpR.

### Transcriptome analysis expanded the RhpRS regulon.

**FIG 3:**
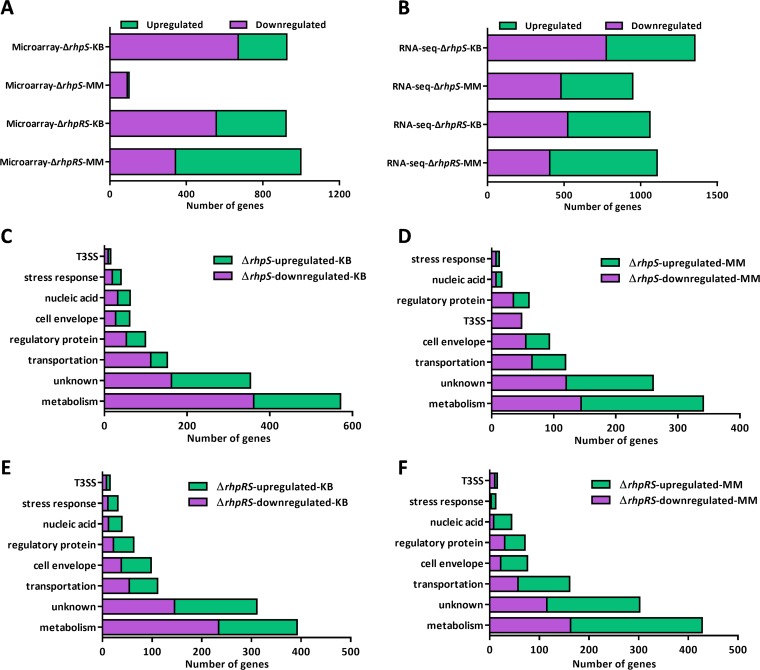
Transcriptome-revealed RhpRS-regulated genes in KB and MM. Transcriptome analysis of the RhpRS regulon in both KB and MM. (A) Number of RhpRS-regulated genes identified by our previous microarray assay. (B) Number of RhpRS-regulated genes identified by RNA-seq in this study. (C) Functional categories of the RhpR-regulated genes in KB identified by RNA-seq. Details of the genes are listed in Table S4A and B. (D) Functional categories of the RhpR-regulated genes in MM identified by RNA-seq. Details of the genes are listed in Table S4C and D. (E) Functional enrichment of the RhpRS-regulated genes in KB identified by RNA-seq. Details of the genes are listed in Table S4E and F. (F) Functional enrichment of the RhpRS-regulated genes in MM identified by RNA-seq. Details of the genes are listed in Table S4G and H.

Our previous microarray analyses have explored the RhpRS regulons in both KB and MM (Fig. 3A) (25). By comparing our previous ChIP-seq and microarray results, we found that RhpR had 54 more binding loci than its regulated genes in MM, indicating that our previous microarray analyses did not fully uncover the RhpRS regulons. We therefore performed RNA-seq analyses for the wild-type, Δ*rhpS*, and Δ*rhpRS* strains in KB and MM. By comparing the gene expression profiles in these strains, we defined the genes that showed a >2-fold difference as differentially expressed genes (DEGs). In the Δ*rhpS* strain in KB, 578 genes were upregulated (see Table S4A in the supplemental material) and 775 were downregulated (Table S4B) compared to the wild type. By mutating *rhpS* in MM (Fig. 3B), 468 genes were upregulated (Table S4C) and 480 were downregulated (Table S4D) compared to the wild type. In the Δ*rhpRS* strain, 536 genes were upregulated (Table S4E) and 524 were downregulated (Table S4F) in KB. Under the MM condition (Fig. 3B), 700 genes were upregulated (Table S4G) and 407 were downregulated (Table S4H). In the Δ*rhpS* strain, 571 and 341 metabolism-related genes were differentially expressed in KB and MM, respectively. This result indicates a more significant role for RhpR in the metabolic regulation in KB than MM (Fig. 3C and D).

Based on the RNA-seq results, we discovered a group of new RhpR functions that were missing in our previous microarray assay. In KB, RhpS negatively regulated four cytochrome biosynthesis genes (*cyoD*, *cyoC*, *ccoQ*, and PSPPH_0227), suggesting that RhpS plays a role in the process of oxidative phosphorylation. RhpS also positively regulated 80 genes encoding ABC transporters for amino acids, sugars, metal ions, and various other metabolites, implying its role in promoting the transmembrane transport of substances under nutrient-rich conditions. Among the genes downregulated in the Δ*rhpS* strain in KB, nine encoded proteins that are associated with transporting and sensing metal ions, including two metal-sensing histidine kinases (PSPPH_3295 and PSPPH_4827), two nickel ABC transporters (*nikB* and PSPPH_2293), two siderophore biosynthesis proteins (*iucD* and PSPPH_3734), one copper-translocating protein (PSPPH_4643), one potassium transporter (TrkA), and one magnesium chelatase ATPase (BchI). This result suggests that RhpS positively tunes membrane permeability for metal ions when nutrients are sufficient. Among the genes upregulated in the Δ*rhpS* strain in MM, seven were associated with the type II secretion pathway (T2SS) and three with the type I secretion pathway (T1SS). Consistent with our previous findings, 48 T3SS genes were downregulated in the Δ*rhpS* strain in MM (22). Two genes encoding RNA polymerase sigma factors (PSPPH_0927 and PSPPH_4765) were downregulated in the Δ*rhpS* strain in MM, while three genes (PSPPH_1092, PSPPH_0345, and PSPPH_2067) were upregulated in the Δ*rhpS* strain in KB, suggesting that RhpR controlled global gene transcription by tuning these sigma factors in response to different nutrient conditions.

In the Δ*rhpRS* strain, 1,060 and 1,107 genes were differentially expressed in KB and MM, respectively, compared to the wild type (Fig. 3B). We found 139 (KB) and 109 (MM) more RhpRS-regulated genes than in our previous microarray data. The functional classifications of the DEGs in both KB and MM, summarized in Fig. 3E and F, show new cellular functions of the RhpRS system under different cultural conditions. Among the genes upregulated in the Δ*rhpRS* strain in KB, 31 were related to chemotaxis and sensory proteins, including five major facilitator family proteins, two TonB-dependent receptors, 11 sensory proteins, and 13 methyl-accepting chemotaxis proteins. This result indicates the negative role of RhpRS in sensing an external stimulus under nutrient-rich conditions. In addition, three drug resistance genes (PSPPH_3553, PSPPH_3554, and *marR*) were downregulated in the Δ*rhpRS* strain in KB. Four genes involved in DNA repair and recombination (*baeS1*, *topB1*, *radA*, and PSPPH_0753) and four genes encoding diguanylate cyclase (synthesizing c-di-GMP) were upregulated in Δ*rhpRS* in MM. Six prophage genes that encode toxins (27) and three type IV pilus biogenesis genes (*pilZ*, *pilO*, and *pilR*) were downregulated by RhpRS.

### Phosphorylated RhpR inhibited the T3SS by directly binding to the promoters of *hrpR* and *hopR1*.

**FIG 4:**
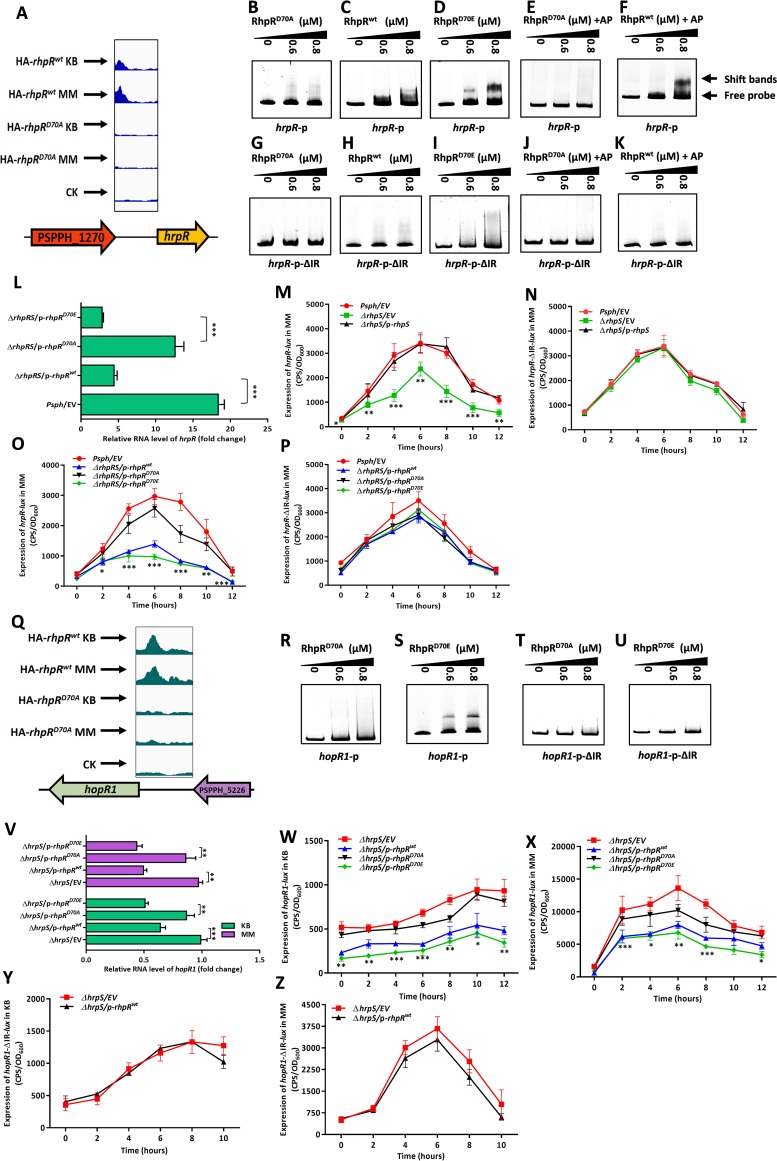
RhpR binds to *hrpR* and *hopR1* promoter regions by targeting the IR element and represses the induction of T3SS. RhpR directly inhibits *hrpR* and *hopR1* by targeting the IR element. (A) RhpR binds to the promoter region of *hrpR*. (B to F) Validation of binding of RhpR to *hrpR* promoter regions by EMSA. The full-length *hrpR* promoter was subjected to EMSA with RhpR, RhpR pretreated with 20 mM acetyl phosphate, RhpR^D70A^, RhpR^D70A^ pretreated with 20 mM acetyl phosphate, and RhpR^D70E^. (G to K) Validation of the binding site of RhpR to the *hrpR* promoter regions by EMSA. The IR element in the *hrpR* promoter was deleted by using overlap PCR, and products were added to the EMSA reaction mixtures. (L) RT-qPCR reveals that RhpR suppresses the expression of *hrpR*. pHM1-RhpR, pHM1-RhpR^D70A^, pHM1-RhpR^D70E^, or pHM1 empty vector was transformed into the *P. savastanoi* pv. *phaseolicola* 1448A Δ*rhpRS* strain. RT-qPCR was performed to measure the transcription level of *hrpR* in all strains. (M and N) Regulation of *hrpR* and *hrpR*-ΔIR promoters by RhpR *in vivo*. Activities of *hrpR*-*lux* or *hrpR*-ΔIR-*lux* were introduced into the wild-type 1448A strain, Δ*rhpS* strain, and Δ*rhpS* strain carrying the pHM1-*rhpS* plasmid. The bacteria were grown in KB and induced in MM before measurement of luciferase (*lux*) activities. (O and P) Regulation of *hrpR* and *hrpR*-ΔIR promoters by RhpR in the Δ*rhpRS* strain. Activities of *hrpR*-*lux* or *hrpR*-ΔIR-*lux* were introduced into the Δ*rhpRS* strain, Δ*rhpRS* strain carrying the pHM1-*rhpR* plasmid, Δ*rhpRS* strain carrying the pHM1-*rhpR*^D70A^ plasmid, and Δ*rhpRS* strain carrying the pHM1-*rhpR*^D70E^ plasmid. (Q) Original sequence peaks show the RhpR binding regions in the *hopR1* promoter. The binding peaks diminished in RhpR^D70A^ background strains. (R and S) EMSA shows that RhpR directly binds to the *hopR1* promoter region. The full-length *hopR1* promoter was subjected to EMSA with RhpR or RhpR^D70E^. (T and U) The *hrpR* promoter lacking the IR element was used in the EMSA reaction. The *hopR1*-ΔIR promoter was subjected to EMSA with RhpR or RhpR^D70E^. (V) RT-qPCR shows that RhpR independently suppresses the expression of *hopR1*. pHM1-RhpR, pHM1-RhpR^D70A^, pHM1-RhpR^D70E^, or pHM1 empty vector was transformed into the Δ*hrpS* strain. RT-qPCR was performed to measure the transcription level of *hopR1* in both strains. (W to Z) Regulation of *hopR1* promoters and *hopR1*-ΔIR promoters by RhpR in the Δ*hrpS* strain. Activities of *hopR1*-*lux* and *hopR1*-ΔIR-*lux* were introduced into the Δ*hrpS* strain, Δ*hrpS* strain carrying the pHM1-*rhpR* plasmid, Δ*hrpS* strain carrying the pHM1-*rhpR*^D70A^ plasmid, and Δ*hrpS* strain carrying the pHM1-*rhpR*^D70E^ plasmid. *, *P* < 0.05, **, *P* < 0.01, and ***, *P* < 0.001, compared to the Δ*rhpS*, Δ*rhpRS*/p*-rhpR*^D70A^, or Δ*hrpS* strain by Student's *t* test. Each experiment was performed three times. Error bars represent standard error.

RhpR had a phosphorylation-dependent binding peak that was located in the 5′ terminus of the *hrpR* promoter (Fig. 4A). To verify the interaction, we analyzed the EMSA results and found at the same protein concentrations that RhpR and RhpR^D70E^ (Asp70 replaced by Glu, a constitutively active mutation) efficiently bound to the *hrpR* promoter (1,081 bp), while the RhpR^D70A^ did not (Fig. 4B to D). The addition of 20 mM acetyl phosphate (AP) significantly increased the binding affinity of RhpR, but not RhpR^D70A^, to the full-length *hrpR* promoter (Fig. 4E and F), in agreement with previous work (25). This result indicates that the phosphorylation was important for RhpR to bind to the *hrpR* promoter. Because RhpR specifically binds to promoters carrying an inverted repeat (IR) element (26), we searched for an IR in the binding region. As expected, an imperfect IR sequence with two mismatches (ATTTC-N_6_-GATAC [mutations underlined]) was found at 958 bp upstream of the coding region of *hrpR* (see Table S1 in the supplemental material). We further hypothesized that RhpR would bind to the *hrpR* promoter by specifically targeting this putative IR element. We repeated the EMSA using an *hrpR*-p-ΔIR probe (full-length *hrpR* promoter without a 16-bp putative IR element that was made by overlap PCR). The *hrpR*-p-ΔIR probe failed to interact with any forms of RhpR, even in the presence of acetyl phosphate (Fig. 4G to K).

10.1128/mBio.02838-18.7 TABLE S1 RhpR-binding motifs in promoter regions. Mismatches are underlined in IR sequences. Download Table S1, DOCX file, 0.01 MB. Copyright © 2019 Xie et al. 2019 Xie et al. This content is distributed under the terms of the Creative Commons Attribution 4.0 International license.

Because RhpR is a negative regulator of the *hrpR* promoter (22), we hypothesized that RhpR would repress the *hrpR* expression in a phosphorylation-dependent manner. We first overexpressed *rhpR* or *rhpR*^D70E^ in the Δ*rhpRS* strain, which resulted in significant inhibition of *hrpR* in MM (Fig. 4L). However, the introduction of *rhpR*^D70A^ did not alter the mRNA levels of *hrpR* (Fig. 4L), which suggests that RhpR relied on phosphorylation to inhibit the *hrpR* transcription. To test whether the promoter activity of *hrpR* was regulated by RhpR, the full-length or IR-deleting (ΔIR) *hrpR* promoter was cloned into the pMS402-*lux* reporter plasmids and transformed into the *P. savastanoi* pv. *phaseolicola* 1448A wild-type strain, the Δ*rhpS* strain, and its complemented (Δ*rhpS*/p*-rhpS*) strain. As shown in Fig. 4M, the activity of the *hrpR-lux*-carrying full-length promoter was ∼2-fold lower in the Δ*rhpS* strain than in the parental strain in MM, while the expression of *rhpS* restored its activity to wild-type levels. However, the relative activity of the *hrpR-*ΔIR*-lux* promoter without IR showed no difference in these two strains (Fig. 4N), indicating that RhpR directly suppressed the *hrpR* transcription by targeting the IR element. To determine whether the suppression of RhpR on *hrpR* was phosphorylation dependent, we measured the *hrpR*-*lux* activity in the wild-type and Δ*rhpRS* strains expressing *rhpR*, *rhpR*^D70A^, or *rhpR*^D70E^. As shown in Fig. 4O, the expression of RhpR or RhpR^D70E^ in the Δ*rhpRS* strain reduced the activity of *hrpR-lux* by ∼3-fold, while RhpR^D70A^ had no effect. As expected, the introduction of p-*rhpR*/*rhpR*^D70A^/*rhpR*^D70E^ had no influence on the *hrpR-*ΔIR*-lux* activity in the Δ*rhpS* strain (Fig. 4P). Taken together, the results from these *in vivo* and *in vitro* analyses demonstrate that the phosphorylation of RhpR promoted its binding affinity to the IR element in the *hrpR* promoter, thus inhibiting the transcription of *hrpRS*.

The T3SS effector gene *hopR1* is positively regulated by HrpL (28). Our ChIP-seq results revealed a binding peak of RhpR in the promoter region of *hopR1* (Fig. 4Q), which carries a putative IR sequence (Table S1). As shown in Fig. 4R to U, RhpR^D70E^ had a stronger binding activity with the *hopR1* promoter than RhpR^D70A^. However, neither RhpR^D70A^ nor RhpR^D70E^ bound to the *hopR1*-ΔIR promoter under the same concentrations, suggesting that RhpR bound to and directly regulated the *hopR1* promoter by recognizing the IR element in a phosphorylation-dependent manner. To further investigate whether RhpR directly regulated *hopR1* in the absence of the *hrpRS-hrpL* cascade, we subsequently performed a real-time quantitative PCR (RT-qPCR) analysis and a *lux* reporter assay in the Δ*hrpS* strain by expressing *rhpR*/*rhpR*^D70A^/*rhpR*^D70E^. As shown in Fig. 4V to X, the mRNA level of *hopR1* and the activity of the *hopR1* promoter were suppressed by about 2-fold from the expression of *rhpR* or *rhpR*^D70E^ in KB and MM. The *rhpR*^D70A^ gene had almost no effect on the mRNA level of *hopR1* and the activity of the *hopR1* promoter. As shown in Fig. 4Y and Z, the expression of *rhpR* in the Δ*hrpS* strain also failed to suppress the *hopR1*-ΔIR*-lux* activity. Taken together, these results suggest that RhpR-P directly inhibited the expression of *hopR1* independent of the *hrpRS-hrpL* cascade.

### RhpR-P negatively regulated the swimming motility but positively regulated the twitching motility by binding to the promoters of *flhA* and *fimA*.

**FIG 5:**
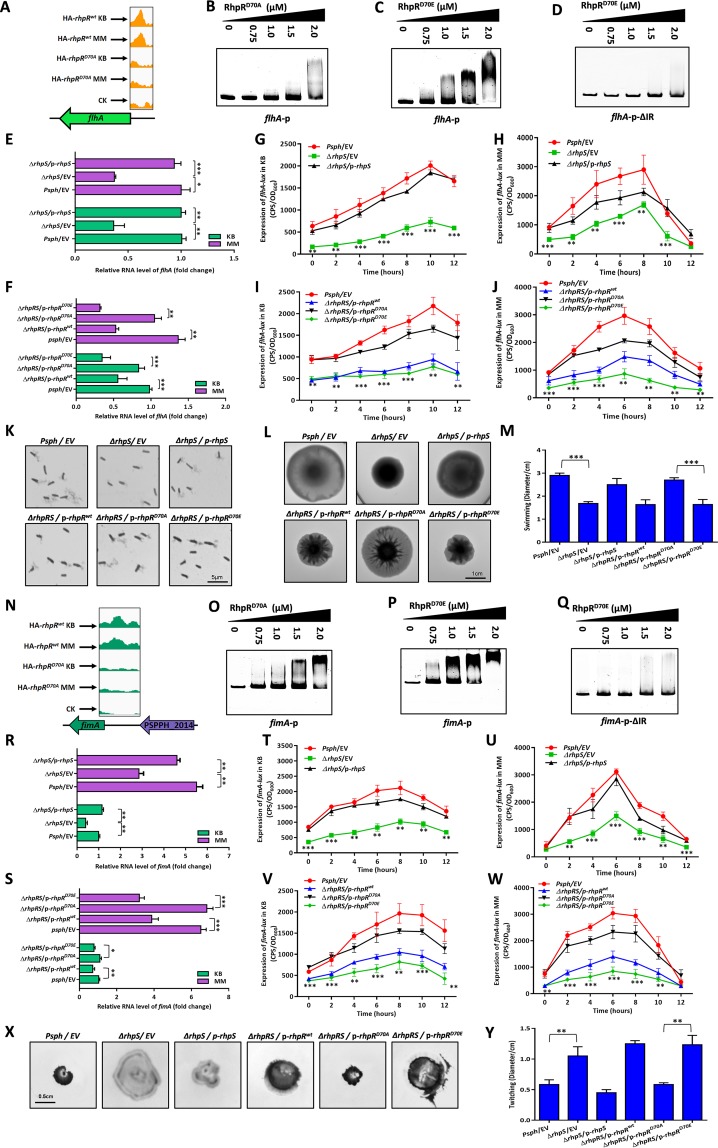
RhpR negatively regulates swimming and positively regulates twitching motility. RhpR directly regulates swimming and twitching motility. (A) RhpR binds to the promoter region of *flhA*. (B and C) The phosphorylation of RhpR enhances the binding activity with promoter regions of *flhA.* PCR products containing the *flhA* promoter sequence were added to the EMSA reaction mixtures. (D) Validation of binding of RhpR to *flhA*-ΔIR promoter regions by EMSA. The *flhA* promoter without the putative IR element was subjected to EMSA with RhpR^D70E^. (E) RT-qPCR reveals that RhpR suppresses the mRNA level of *flhA*. The wild-type strain, Δ*rhpS* strain, and Δ*rhpS* strain carrying the pHM1-*rhpS* plasmid were grown in KB and induced in MM for 6 h. RT-qPCR was performed to measure the transcription level of *flhA*. (F) RT-qPCR shows that RhpR suppresses the expression of *flhA*. pHM1-RhpR, pHM1-RhpR^D70A^, pHM1-RhpR^D70E^, or pHM1 empty vector was transformed into the Δ*rhpRS* strain. RT-qPCR was performed to measure the transcription level of *flhA*. (G and H) RhpR directly suppresses the expression of *flhA in vivo*. The *flhA*-*lux* reporter was transformed into the wild-type strain, Δ*rhpS* strain, and complemented strain. A single colony was cultured in KB until it reached an OD_600_ of 0.6 and then transferred into MM. (I and J) Regulation of *flhA* promoters by RhpR in the Δ*rhpRS* strain. Activities of *flhA*-*lux* were introduced into the Δ*rhpRS* strain, Δ*rhpRS* strain carrying the pHM1-*rhpR* plasmid, Δ*rhpRS* strain carrying the pHM1-*rhpR*^D70A^ plasmid, and Δ*rhpRS* strain carrying the pHM1-*rhpR*^D70E^ plasmid. (K) Visualization of flagellar abundance in *P. savastanoi* pv. *phaseolicola* 1448A strains taken from KB motility plates. Shown are light microphotographs of cells from KB motility plates stained by the Leifson method. The scale bar represents 5 μm. **(**L and M) Effect of RhpR overexpression on swimming motility. Overnight cultures were spotted onto swimming plates (2-μl aliquots), and the plates were incubated at 28°C. The images were captured after 36 h of growth. (N) RhpR binds to the promoter region of *fimA*. (O and P) The phosphorylated RhpR has higher binding activity with the *fimA* promoter. The *fimA* promoter fragments were added to the EMSA reaction mixtures. RhpR^D70A^ and RhpR^D70E^ proteins were added to reaction buffer in lanes. (Q) Validation of binding of RhpR to *fimA*-ΔIR promoter regions by EMSA. The *fimA* promoter without the putative IR element was subjected to EMSA with RhpR^D70E^. (R) RT-qPCR reveals that RhpR suppresses the mRNA level of *fimA*. The wild-type strain, Δ*rhpS* strain, and Δ*rhpS* strain carrying the pHM1-*rhpS* plasmid were grown in KB and induced in MM for 6 h. RT-qPCR was performed to measure the transcription level of *fimA*. (S) RT-qPCR shows that RhpR suppresses the expression of *fimA*. pHM1-RhpR, pHM1-RhpR^D70A^, pHM1-RhpR^D70E^, or the pHM1 empty vector was transformed into the Δ*rhpRS* strain. RT-qPCR was performed to measure the transcription level of *fimA*. (T and U) RhpR directly suppresses the expression of *fimA in vivo*. The *fimA*-*lux* reporter was transformed into the wild-type, Δ*rhpS*, and complemented strains. A single colony was cultured in KB until it reached an OD_600_ of 0.6 and then transferred into MM. (V and W) Regulation of *fimA* promoters by RhpR in the Δ*rhpRS* strain. Activities of *fimA*-*lux* were introduced into the Δ*rhpRS* strain, Δ*rhpRS* strain carrying the pHM1-*rhpR* plasmid, Δ*rhpRS* strain carrying the pHM1-*rhpR*^D70A^ plasmid, and Δ*rhpRS* strain carrying the pHM1-*rhpR*^D70E^ plasmid. (X and Y) Effect of RhpR overexpression on swimming motility. Overnight cultures were inoculated into twitching plates (3-μl aliquots), and the plates were incubated at 28°C. The images were captured after 36 h of growth. The experiments were repeated at least three times, and similar results were observed. *, *P* < 0.05, **, *P* < 0.01, and ***, *P* < 0.001, compared to the Δ*rhpS* or Δ*rhpRS*/p*-rhpR*^D70A^ strain by Student's *t* test. Data are representative of three independent experiments.

RhpR had a phosphorylation-dependent binding peak that was located in the *flhA* promoter region (Fig. 5A). The *flhA* gene encodes a membrane component of the flagellar export apparatus (29), which is essential for swimming motility in P. syringae (30). As shown in Fig. 5B and C, the EMSA results verified the interaction between RhpR^D70E^ and the *flhA* promoter. RhpR^D70A^ had a modest binding affinity at the same protein concentrations, indicating that the phosphorylation state was essential for RhpR to bind to the *flhA* promoter. As shown in Fig. 5D, RhpR failed to bind to an *flhA*-p-ΔIR sequence, which has a putative IR element deleted (Table S1). As shown in Fig. 5E, the transcription level of *flhA* in the Δ*rhpS* strain was ∼3-fold lower than that in the wild-type strain and Δ*rhpS*/p*-rhpS* complemented strain. The *flhA* expression was repressed by ∼2-fold by RhpR^D70E^, but not RhpR^D70A^, in the Δ*rhpRS* strain (Fig. 5F). These results were confirmed by corresponding *lux* assays (Fig. 5G to J; see Fig. S6A and B in the supplemental material). To determine whether RhpR directly regulated the biosynthesis of flagella via *flhA*, a flagellar stain for light microscopy was used in cells that were cultured on soft KB plates (0.3% agar). As shown in Fig. 5K, the Δ*rhpS* strain produced less and shorter flagella than did the wild-type strain, while the overexpression of *rhpS* restored the flagellar production and morphology to the wild-type level. Overexpression of *rhpR* or *rhpR*^D70E^ in the Δ*rhpRS* strain led to lower biosynthesis of flagella than *rhpR*^D70A^ (Fig. 5K). As shown in Fig. 5L and M, the Δ*rhpS* strain showed a reduction in swimming that was ∼44% less than that in the wild-type and Δ*rhpS*/p*-rhpS* complemented strains. The expression of *rhpR* or *rhpR*^D70E^ in the Δ*rhpRS* strain resulted in decreased swimming motility, compared to the strain overexpressing RhpR^D70A^ (Fig. 5L and M). Collectively, both the *in vivo* and *in vitro* results demonstrate that RhpR-P negatively regulated the *flhA* expression by targeting the IR element, thus suppressing the biosynthesis of flagella and swimming motility.

Another motility-related gene bound by RhpR-P is *fimA* (Fig. 5N), which encodes the type 1 fimbrial subunit and regulates twitching motility to partially restrict cell movement (31–33). As shown in Fig. 5O and P, RhpR^D70E^ had a higher binding affinity to the *fimA* promoter than RhpR^D70A^. As expected, RhpR^D70E^ did not bind to the *fimA* promoter when the putative IR sequence was deleted (Fig. 5Q and Table S1). The RT-qPCR assay and corresponding *lux*-based reporter assays verified that the transcription level of *fimA* was repressed ∼2-fold by either RhpR or RhpR^D70E^ (Fig. 5R to W and Fig. S6C and D). To further verify whether RhpR-P directly regulated twitching motility, we tested the twitching phenotype for these three strains. As shown in Fig. 5X and Y, the size of the twitching zone of the Δ*rhpS* strain was ∼2-fold larger than those in the other two strains. In the Δ*rhpRS* strain, the twitching motility was induced by ∼2-fold from the expression of RhpR and RhpR^D70E^ compared to RhpR^D70A^. Collectively, our results show that RhpR-P positively regulated the twitching motility by directly suppressing *fimA*.

### RhpR-P negatively regulated the production of exopolysaccharides and biofilm by directly binding to the promoter of *algD*.

As shown in Fig. S2A in the supplemental material, a phosphorylation-dependent binding peak was found in the *algD* promoter region. The *algD* gene encodes a GDP-mannose dehydrogenase that contributes to the formation of biofilm and extracellular polysaccharide (EPS) (34–36). The EMSA results showed that RhpR had a higher binding affinity to the *algD* promoter probe than RhpR^D70A^ (Fig. S2B and S2C), but the deletion in the IR sequence reduced the interaction between *algD*-p*-*ΔIR and RhpR (Fig. S2D and Table S1). As shown in Fig. S2E and S2F, the transcription level of *algD* in the Δ*rhpS* strain was ∼2.5-fold lower than that in the wild-type and Δ*rhpS*/p-*rhpS* complemented strain, and the RhpR-mediated regulation of *algD* was dependent on D70. The subsequent *algD-lux* assay demonstrated that RhpR directly regulated *algD* binding to the IR element (Fig. S2G to J and Fig. S6E and F). We also measured the EPS and biofilm production of these strains. As shown in Fig. S2K and L, the Δ*rhpS* strain had more smooth colonies, indicating less EPS production, and 2.5-fold less biofilm production than the other two strains. As expected, the introduction of p-*rhpR* or p-*rhpR*^D70E^ into the Δ*rhpRS* strain led to lower EPS and biofilm production than p-*rhpR*^D70A^ (Fig. S2K and L). Taken together, these results show that RhpR-P negatively regulated *algD*, which leads to the production of EPS and biofilm.

10.1128/mBio.02838-18.2 FIG S2 RhpR negatively regulates the production of EPS and biofilm. RhpR directly suppresses the production of EPS and biofilm. (A) RhpR binds to the promoter region of *algD*. (B and C) The phosphorylation of RhpR enhances the binding activity with promoter regions of *algD.* PCR products containing the *algD* promoter sequence were added to the EMSA reaction mixtures at 50 nM each. (D) Validation of binding of RhpR to *algD*-ΔIR promoter regions by EMSA. The *algD* promoter without the putative IR element was subjected to EMSA. (E) RT-qPCR reveals that RhpR suppresses the mRNA level of *algD*. The wild-type strain, Δ*rhpS* strain, and Δ*rhpS* strain carrying the pHM1-*rhpS* plasmid were grown in KB and induced in MM for 6 h. RT-qPCR was performed to measure the transcription level of *algD*. (F) RT-qPCR shows that RhpR suppresses the expression of *algD*. pHM1-RhpR, pHM1-RhpR^D70A^, pHM1-RhpR^D70E^, or pHM1 empty vector was transformed into the Δ*rhpRS* strain. RT-qPCR was performed to measure the transcription level of *algD*. (G and H) RhpR directly suppresses the expression of *algD in vivo*. The *algD*-*lux* reporter was transformed into the wild-type, Δ*rhpS*, and complemented strains. A single colony was cultured in KB until it reached an OD_600_ of 0.6 and then transferred into MM. Luciferase gene (*lux*) activities were measured over 6 h. (I and J) Regulation of *algD* promoters by RhpR in the *P. savastanoi* pv. *phaseolicola* 1448A Δ*rhpRS* strain. Activities of *algD*-lux were introduced into the Δ*rhpRS* strain, Δ*rhpRS* strain carrying the pHM1-*rhpR* plasmid, Δ*rhpRS* strain carrying the pHM1-*rhpR*^D70A^ plasmid, and Δ*rhpRS* strain carrying the pHM1-*rhpR*^D70E^ plasmid. The bacteria were grown in KB medium and induced in MM. (K) The overexpression of RhpR reduces the production of exopolysaccharides (EPS). Different levels of red of colony morphology on the Congo red plate represent the relative amounts of EPS. (L) Quantification of CV staining of biofilm grown in borosilicate tubes at 48 h after standing incubation at 28°C. Photos of the tubes from the binding assay were taken. *, *P* < 0.05, **, *P* < 0.01, and ***, *P* < 0.001, compared to the Δ*rhpS* or Δ*rhpRS*/p*-rhpR*^D70A^ strain by Student’s *t* test. Data are representative of three independent experiments. Download FIG S2, TIF file, 2.4 MB. Copyright © 2019 Xie et al. 2019 Xie et al. This content is distributed under the terms of the Creative Commons Attribution 4.0 International license.

### RhpR-P negatively regulated the c-di-GMP level *in vivo* and positively regulated the production of lipopolysaccharides by binding to the PSPPH_2590 and PSPPH_2653 promoter regions, respectively.

RhpR-P had a specific binding peak in the promoter region of PSPPH_2590 (see Fig. S3A in the supplemental material), whose product is predicted to carry a GGDEF domain (characteristic of a diguanylate cyclase) domain and an EAL domain (characteristic of a phosphodiesterase), which are responsible for the synthesis and degradation of c-di-GMP (37–39). The EMSA results showed that RhpR^D70E^ had stronger binding activity with the PSPPH_2590 promoter than RhpR^D70A^ (Fig. S3B and C). As shown in Fig. S3D and Table S1, the PSPPH_2590-p-ΔIR sequence was not bound by RhpR. The expression of PSPPH_2590 was repressed 3-fold by RhpR^D70E^, but not RhpR^D70A^ (Fig. S3E and Fig. S3F). These results were confirmed by the subsequent PSPPH_2590*-lux* assays (Fig. S3G to J and Fig. S6G and H). To verify whether RhpR regulated the intracellular level of c-di-GMP, the level of c-di-GMP was measured in the Δ*rhpS* strain using a PSPTO_5471 promoter-*lux* reporter, which was induced by increasing the intracellular levels of c-di-GMP in P. syringae (unpublished observations). As shown in Fig. S3K and L, the level of c-di-GMP in the Δ*rhpS* strain was reduced by ∼1.5-fold compared to the other two strains. In the Δ*rhpRS* strains, the c-di-GMP level was reduced by ∼2-fold from the expression of RhpR and RhpR^D70E^ compared to RhpR^D70A^. Taken together, the results of these analyses suggest that RhpR-P directly suppressed the expression of PSPPH_2590, thus inhibiting the production of c-di-GMP *in vivo*.

10.1128/mBio.02838-18.3 FIG S3 RhpR-P negatively regulated the c-di-GMP level *in vivo* and positively regulated the production of lipopolysaccharides by binding to the PSPPH_2590 and PSPPH_2653 promoter regions, respectively. RhpR directly regulates the production of c-di-GMP and lipopolysaccharides. (A) RhpR binds to the promoter region of PSPPH_2590. (B and C) The phosphorylation of RhpR enhances the binding activity with promoter regions of PSPPH_2590. PCR products containing the PSPPH_2590 promoter sequence were added to the EMSA reaction mixtures at 50 nM each. (D) Validation of binding of RhpR to PSPPH_2590-ΔIR promoter regions by EMSA. The PSPPH_2590 promoter without the putative IR element was subjected to EMSA with RhpR^D70E^. (E) RT-qPCR reveales that RhpR suppresses the mRNA level of PSPPH_2590. The wild-type strain, Δ*rhpS* strain, and Δ*rhpS* strain carrying the pHM1-*rhpS* plasmid were grown in KB and induced in MM for 6 h. RT-qPCR was performed to measure the transcription level of PSPPH_2590. (F) RT-qPCR shows that RhpR suppresses the expression of PSPPH_2590. pHM1-RhpR, pHM1-RhpR^D70A^, pHM1-RhpR^D70E^, or pHM1 empty vector was transformed into the Δ*rhpRS* strain. RT-qPCR was performed to measure the transcription level of PSPPH_2590. (G and H) RhpR directly suppresses the expression of PSPPH_2590 *in vivo*. The PSPPH_2590-*lux* reporter was transformed into the wild-type, Δ*rhpS*, and complemented strains. A single colony was cultured in KB until it reached an OD_600_ of 0.6 and then transferred into MM. Luciferase gene (*lux*) activities were measured over 6 h. (I and J) Regulation of PSPPH_2590 promoters by RhpR in the Δ*rhpRS* strain. PSPPH_2590-*lux* was introduced into the Δ*rhpRS* strain, Δ*rhpRS* strain carrying the pHM1-*rhpR* plasmid, Δ*rhpRS* strain carrying the pHM1-*rhpR*^D70A^ plasmid, and Δ*rhpRS* strain carrying the pHM1-*rhpR*^D70E^ plasmid. The bacteria were grown in KB and induced in MM before measurement of *lux* activities. (K and L) The overexpression of RhpR reduces the production of c-di-GMP in 1448A. PSPTO_5471-*lux* reporter plasmid was transformed into the wild-type strain, Δ*rhpS* strain, Δ*rhpS*/p-*rhpS* complemented strain, Δ*rhpRS* strain, Δ*rhpRS*/pHM1-*rhpR* complemented strain, Δ*rhpRS*/pHM1-*rhpR*^D70A^ strain, and Δ*rhpRS*/pHM1-*rhpR*^D70E^ strain. The bacteria were grown in MM for 0, 2, 4, 6, and 8 h before measurement of *lux* activities. (M) RhpR binds to the promoter regions of PSPPH_2653 in KB according to the ChIP-seq results. (N and O) The phosphorylation of RhpR enhanced the binding activity with the promoter region of PSPPH_2653. PCR products containing the PSPPH_2653 promoter sequence were added to the EMSA reaction mixtures at 50 nM each. (P) RT-qPCR reveals that RhpR promotes PSPPH_2653 expression. pHM1-RhpR, pHM1-RhpR^D70A^, pHM1-RhpR^D70E^, or pHM1 empty vector was transformed into the *P. savastanoi* pv. *phaseolicola* 1448A Δ*rhpRS* strain; all strains were grown in KB and induced in MM for 6 h. RT-qPCR was performed to measure the transcription level of PSPPH_2653. (Q and R) Regulation of PSPPH_2590 promoters by RhpR in the Δ*rhpRS* strain. PSPPH_2653-*lux* was introduced into the Δ*rhpRS* strain, Δ*rhpRS* strain carrying the pHM1-*rhpR* plasmid, Δ*rhpRS* strain carrying the pHM1-*rhpR*^D70A^ plasmid, and Δ*rhpRS* strain carrying the pHM1-*rhpR*^D70E^ plasmid. The bacteria were grown in KB and induced in MM before measurement of *lux* activities. (S and T) The production of LPS was enhanced by RhpR. The wild-type, Δ*rhpS*, Δ*rhpS/*p*-rhpS*, Δ*rhpRS*/p*-rhpR*, Δ*rhpRS*/p*-rhpR*^D70A^, and Δ*rhpRS*/p*-rhpR*^D70E^ strains were grown in KB to an OD of 0.6. The bacteria were collected, and LPS extraction was performed. The production of LPS was determined by silver staining and the anthrone-sulfuric acid colorimetric method. *, *P* < 0.05, **, *P* < 0.01, and ***, *P* < 0.001, compared to the Δ*rhpS* or Δ*rhpRS/*p*-rhpR*^D70A^ strain by Student’s *t* test. Data are representative of three independent experiments. Download FIG S3, TIF file, 2.3 MB. Copyright © 2019 Xie et al. 2019 Xie et al. This content is distributed under the terms of the Creative Commons Attribution 4.0 International license.

Our previous results revealed that RhpR positively regulated PSPTO_2767 by recognizing a putative IR element with one mismatch (underlined in GTATC-N_6_-GGTAC) in the promoter region (26). In this study, we further detected and verified the interaction between the PSPPH_2653 (orthologue of PSPTO_2767 in the *P. savastanoi* pv. *phaseolicola* 1448A strain) promoter and RhpR-P using ChIP-seq and an EMSA (Fig. S3M to O). PSPTO_2767 and PSPPH_2653 encode a putative lipopolysaccharide (LPS) core biosynthesis domain protein (40). RT-qPCR and the corresponding *lux*-based reporter assays in the Δ*rhpRS* strain showed that D70 was essential to the RhpR-mediated regulation of PSPPH_2653 (Fig. S3P to R). We also measured the production of LPS in these strains by using silver staining and an anthrone-sulfuric acid colorimetric method. As shown in Fig. S3S and T, the Δ*rhpS* strain synthesized ∼1.5-fold more LPS than the other two strains. The expression of either RhpR or RhpR^D70E^ enhanced the LPS production by ∼1.8-fold compared to RhpR^D70A^ in the Δ*rhpRS* strains. Altogether, our results indicate that RhpR depended on phosphorylation to promote the production of LPS by directly inducing the transcription of PSPPH_2653.

### RhpR positively modulated the accumulation of *c*-type cytochrome and alcohol dehydrogenase activity via *ccmA* and *adhB*, respectively, in a KB-dependent manner.

**FIG 6:**
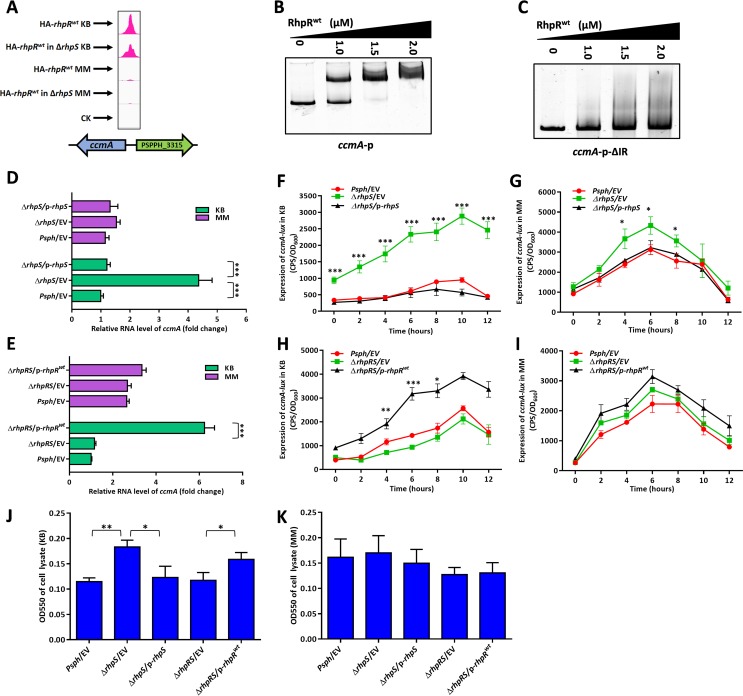
RhpR positively regulates the expression of *c*-type cytochrome in KB medium. RhpR positively regulates the expression of *c*-type cytochrome. (A) RhpR binds to the promoter regions of *ccmA* in KB but not in MM according to the ChIP-seq results. (B) RhpR binds with promoter regions of *ccmA.* PCR products containing the *ccmA* promoter sequence were added to the EMSA reaction mixtures at 50 nM each. (C) Validation of binding of RhpR to *ccmA*-ΔIR promoter regions by EMSA. The *ccmA* promoter without the putative IR element was subjected to EMSA with RhpR. (D) RT-qPCR reveals that RhpR suppresses *ccmA*. The wild-type strain, Δ*rhpS* strain, and Δ*rhpS* strain carrying the pHM1-*rhpS* plasmid were grown in KB and induced in MM for 6 h. RT-qPCR was performed to measure the transcription level of *ccmA* in all three strains. (E) RT-qPCR shows that RhpR suppresses the expression of *ccmA*. pHM1-RhpR or pHM1 empty vector was transformed into the Δ*rhpRS* strain. RT-qPCR was performed to measure the transcription level of *ccmA*. (F and G) RhpR directly suppresses the expression of *ccmA* in KB. The *ccmA*-*lux* reporter plasmid was transformed into the wild-type, Δ*rhpS*, and complemented strains. A single colony was cultured in KB until it reached an OD_600_ of 0.6 and then was transferred into MM, and luciferase gene (*lux*) activities were measured separately. (H and I) Regulation of *ccmA* promoters by RhpR in the Δ*rhpRS* strain. Activities of *ccmA-lux* were introduced into the Δ*rhpRS* strain and Δ*rhpRS* strain carrying the pHM1-*rhpR* plasmid. (J and K) Visible absorption in total soluble extracts from *P. savastanoi* pv. *phaseolicola* 1448A strains in KB or MM. The wild-type strain, Δ*rhpS* strain, complemented strain, Δ*rhpRS* strain, and Δ*rhpRS* strain carrying the pHM1-*rhpR* plasmid were grown with choline as the carbon source to maximize expression of polypeptides for *c*-type cytochromes. Total soluble extracts were adjusted to 15 mg protein per ml. All samples were reduced with sodium dithionite, and the OD_550_ was measured. *, *P* < 0.05, **, *P* < 0.01, and ***, *P* < 0.001, compared to the Δ*rhpS* or Δ*rhpRS/*p*-rhpR*^D70A^ strain by Student's *t* test. Each experiment was performed three times.

Although our previous study indicates that RhpR alters its role to modulate protein synthesis in response to nutrient conditions and regulates more genes in KB than in MM (25), the underlying regulatory mechanisms in different media are largely unknown. We therefore identified 125 KB-dependent RhpR binding sites by comparing the ChIP-seq results of RhpR between KB and MM. As shown in Fig. 6A and B, the EMSA verified that RhpR bound to the promoter region of *ccmA*. The product of *ccmA* is part of the ABC transporter complex that is involved in the biogenesis of *c*-type cytochromes, thus leading to the accumulation of apocytochrome *c*_550_ (41, 42). The deletion of a putative IR region (Table S1) abolished the interaction between the *ccmA* promoter and RhpR (Fig. 6C). Because RhpR binds to the *ccmA* promoter only in KB, we therefore investigated whether RhpR modulated the transcription level of *ccmA* in a KB-dependent manner. An RT-qPCR assay showed that the *ccmA* transcript levels were ∼4-fold higher in the Δ*rhpS* strain than in the wild-type and Δ*rhpS*/p*-rhpS* complemented strains in KB (Fig. 6D). As expected, these three strains had almost the same *ccmA* expression level when grown in MM (Fig. 6D). RT-qPCR in the Δ*rhpRS* strain showed that the RhpR-mediated regulation of *ccmA* was KB dependent (Fig. 6E). The subsequent *ccmA-lux* assay further demonstrated the direct regulation of *ccmA* by RhpR via binding to the IR element in KB, but this result was not found in MM (Fig. 6F to I and Fig. S6I and J). To determine whether RhpR controlled the production of apocytochrome *c*_550_, we performed a spectroscopic analysis of bacterial total soluble fractions prepared from these strains, with the absorption of cell lysate at 550 nm indicating the accumulation of cytochrome *c*_550_ (43). As shown in Fig. 6J, the Δ*rhpS* strain accumulated ∼1.5-fold more *c*-type cytochromes than the other two strains in KB. As expected, expression of RhpR enhanced the production of *c*-type cytochromes by ∼1.5-fold in the Δ*rhpRS* strain in KB but not in MM (Fig. 6K). Collectively, our results show that RhpR positively modulated the *c*-type cytochrome level via directly regulating *ccmA* when the strain was grown in KB.

**FIG 7:**
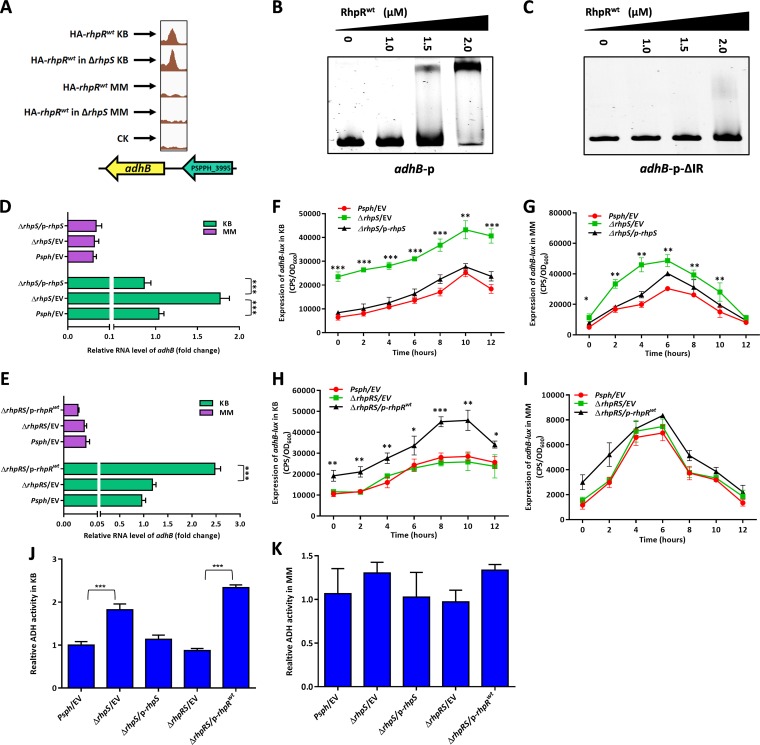
RhpR directly positively regulates alcohol dehydrogenase activity in KB. RhpR binds and positively regulates *adhB*. (A) RhpR binds to the promoter regions of *adhB* in KB but not in MM according to the ChIP-seq results. (B) RhpR binds with promoter regions of *adhB.* PCR products containing the *adhB* promoter sequence were added to the EMSA reaction mixtures at 50 nM each. RhpR protein was added to reaction buffer in lanes. (C) Validation of binding of RhpR to *adhB*-ΔIR promoter regions by EMSA. The *adhB* promoter without the putative IR element was subjected to EMSA with RhpR. (D) RT-qPCR reveals that RhpR suppresses *adhB*. The wild-type strain, Δ*rhpS* strain, and Δ*rhpS* strain carrying the pHM1-*rhpS* plasmid were grown in KB and induced in MM for 6 h. RT-qPCR was performed to measure the transcription level of *adhB* in all three strains. (E) RT-qPCR shows that RhpR suppresses the expression of *adhB*. pHM1-RhpR or pHM1 empty vector was transformed into the Δ*rhpRS* strain. RT-qPCR was performed to measure the transcription level of *adhB*. (F and G) RhpR directly suppresses the expression of *adhB* in KB. The *adhB*-*lux* reporter plasmid was transformed into the wild-type strain, Δ*rhpS* strain, and complemented strain. A single colony was cultured in KB until it reached an OD_600_ of 0.6 and then transferred into MM, and luciferase gene (*lux*) activities were measured separately. (H and I) Regulation of *adhB* promoters by RhpR in the Δ*rhpRS* strain. Activities of *adhB-lux* were introduced into the Δ*rhpRS* strain and Δ*rhpRS* strain carrying the pHM1-*rhpR* plasmid. (J and K) The activity of alcohol dehydrogenase was enhanced in the Δ*rhpS* strain in KB. The wild-type strain, Δ*rhpS* strain, complemented strain, Δ*rhpRS* strain, and Δ*rhpRS* strain carrying the pHM1-*rhpR* plasmid were grown in KB and then transferred to MM. The activity of alcohol dehydrogenase was determined. *, *P* < 0.05, **, *P* < 0.01, and ***, *P* < 0.001, compared to the Δ*rhpS* or Δ*rhpRS*/p*-rhpR*^D70A^ strain by Student's *t* test. Each experiment was performed three times.

RhpR bound to the promoter region of *adhB*, which was confirmed *in vitro* by EMSA (Fig. 7A and B). The product of *adhB* belongs to the alcohol dehydrogenase (ADH) family. ADH catalyzes the reversible reaction between ethanol and acetaldehyde (44). RhpR did not bind to the *adhB* promoter when the IR sequence was deleted (Fig. 7C and Table S1). In KB, using RT-qPCR and the corresponding *lux* assays, the transcription of *adhB* was activated ∼2-fold by RhpR (Fig. 7D to I and Fig. S6K and L). This result was not found in MM. To determine whether RhpR controlled the activity of alcohol dehydrogenase via *adhB*, we tested the enzymatic activity of the total soluble fractions prepared from these three strains. As shown in Fig. 7J and K, the Δ*rhpS* strain had a higher ADH activity (∼1.7-fold) than the other two strains in KB but not in MM. In the Δ*rhpRS* strain, the expression of RhpR enhanced the induction of ADH activity by ∼2-fold in KB but had no effect when the strains were transferred to MM. Our results show that RhpR directly activated the expression of *adhB* and positively regulated ADH activity in a KB-dependent manner.

### Anthranilate synthase activity and protease production were negatively regulated by RhpR via suppressing *trpG* and inducing *hemB*, respectively, in KB.

RhpR bound to the promoter region of *trpG* in KB (see Fig. S4A in the supplemental material), which was confirmed by an EMSA *in vitro* (Fig. S4B). The product of *trpG* is part of a heterotetrameric complex that catalyzes the two-step biosynthesis of anthranilate from chorismate (45). As shown in Fig. S4C to H, the mRNA level of *trpG* was repressed by RhpR in KB, but not in MM. We then measured the anthranilate synthase activity of these strains grown in KB and MM. As shown in Fig. S4I, the Δ*rhpS* strain had lower anthranilate synthase activity by ∼2.5-fold than the wild type in KB. The expression of RhpR in the Δ*rhpRS* strain also suppressed the anthranilate synthase activity by ∼1.5-fold in a KB-dependent manner, which was abolished when the bacteria were cultured in MM (Fig. S4J). Taken together, these results demonstrate that RhpR negatively modulated anthranilate synthase activity by suppressing *trpG* in KB.

10.1128/mBio.02838-18.4 FIG S4 Anthranilate synthase activity and protease production were negatively regulated by RhpR via suppressing *trpG* and inducing *hemB* respectively, in KB. RhpR directly regulates the expression of *trpG* and *hemB*. (A) RhpR binds to the promoter regions of *trpG* in KB but not in MM according to the ChIP-seq results. (B) RhpR binds with the *trpG* promoter. PCR products containing the *trpG* promoter sequence were added to the EMSA reaction mixtures at 50 nM each. (C) RT-qPCR reveals that RhpR suppresses *trpG*. The wild-type strain, Δ*rhpS* strain, and Δ*rhpS* strain carrying the pHM1-*rhpS* plasmid were grown in KB medium and induced in MM for 6 h. RT-qPCR was performed to measure the transcription level of *trpG* in all three strains. (D) RT-qPCR shows that RhpR suppresses the expression of *trpG*. pHM1-RhpR or pHM1 empty vector was transformed into the *P. savastanoi* pv. *phaseolicola* 1448A Δ*rhpRS* strain. RT-qPCR was performed to measure the transcription level of *trpG*. (E and F) RhpR directly suppresses the expression of *trpG* in KB. The *trpG-lux* reporter plasmid was transformed into wild-type P. savastanoi pv. *phaseolicola* 1448A, the Δ*rhpS* strain, and the complemented strain. A single colony was cultured in KB until it reached an OD_600_ of 0.6 and then transferred into MM. Luciferase gene (*lux*) activities were measured over 6 h. (G and H) Regulation of *trpG* promoters by RhpR in the Δ*rhpRS* strain. The activities of *trpG-lux* were introduced into the Δ*rhpRS* strain and Δ*rhpRS* strain carrying the pHM1-*rhpR* plasmid. The bacteria were grown in KB medium and induced in MM before measurement of *lux* activities. (I and J)The activity of anthranilate synthase was suppressed by RhpR in KB medium. The wild-type, Δ*rhpS*, Δ*rhpS*/p*-rhpS*, Δ*rhpRS*/p*-rhpR*, Δ*rhpRS*/p*-rhpR*^D70A^, and Δ*rhpRS*/p*-rhpR*^D70E^ strains were grown in KB to an OD of 0.6 and then transferred to MM for 6 h. The bacteria were collected and sonicated, and the cell lysate supernatant was added to reaction mixture. The production of anthranilate was determined by measuring fluorescence intensity at 400 nm. (K) RhpR binds to the promoter regions of *hemB* in KB but not in MM according to the ChIP-seq results. (L) RhpR binds with promoter regions of *hemB.* PCR products containing the *adhB* promoter sequence were added to the EMSA reaction mixtures at 50 nM each. (M) RT-qPCR reveals that RhpR enhances *hemB*. The wild-type strain, Δ*rhpS* strain, and Δ*rhpS* strain carrying the pHM1-*rhpS* plasmid were grown in KB and induced in MM for 6 h. RT-qPCR was performed to measure the transcription level of *hemB* in all three strains. (N) RT-qPCR shows that RhpR enhances the expression of *hemB*. The pHM1-RhpR or pHM1 empty vector was transformed into the Δ*rhpRS* strain. RT-qPCR was performed to measure the transcription level of *hemB*. (O and P) RhpR directly promotes the expression of *hemB* in KB. The *hemB-lux* reporter plasmid was transformed into the wild-type, Δ*rhpS*, and complemented strains. A single colony was cultured in KB until it reached an OD_600_ of 0.6 and then transferred into MM. *lux* activities were measured over 6 h. (Q and R) Regulation of *hemB* promoters by RhpR in the Δ*rhpRS* strain. Activities of *hemB-lux* were introduced into the Δ*rhpRS* strain and Δ*rhpRS* strain carrying the pHM1-*rhpR* plasmid. The bacteria were grown in KB medium and induced in MM before measurement of *lux* activities. (S and T) The production of protease was downregulated by RhpR in KB medium. The wild-type strain, Δ*rhpS* strain, Δ*rhpS*/p*-rhpS* strain, Δ*rhpRS* strain, and Δ*rhpRS* strain carrying the pHM1-*rhpR* plasmid were grown in KB and then transferred to MM. The bacteria were removed by centrifugation and added to an equal volume of Azocoll substrate. The mixture was incubated at 37°C for 2 h. The protease activity was determined by measuring the absorbance of reaction mixtures at 520 nm. One unit of protease activity was defined as an increase in OD of 0.001. *, *P* < 0.05, **, *P* < 0.01, and ***, *P* < 0.001, compared to the Δ*rhpS* or Δ*rhpRS*/p*-rhpR*^D70A^ strain by Student’s *t* test. Each experiment was performed three times. Download FIG S4, TIF file, 2.3 MB. Copyright © 2019 Xie et al. 2019 Xie et al. This content is distributed under the terms of the Creative Commons Attribution 4.0 International license.

As shown in Fig. S4K and L, RhpR also bound to the promoter region of *hemB*, which encodes a δ-aminolevulinic acid dehydratase in the biosynthesis of tetrapyrroles (46). The transcript level of *hemB* was induced ∼3-fold by RhpR in KB medium (Fig. S4M to R). Because the deletion of *hemB* leads to higher protease production *in vitro* than in the parental strain (47), we measured the protease activity of the wild-type and deletion strains. As shown in Fig. S4S, the Δ*rhpS* strain had ∼1.7-fold-lower *in vitro* protease activity than the parental strain when grown in KB. The expression of RhpR suppressed the protease activity by ∼2-fold in the Δ*rhpRS* background. In contrast, RhpR had no effect on the protease production once the bacteria were cultured in MM (Fig. S4T). Collectively, our results show that RhpR suppressed the protease activity via inducing *hemB* in a KB-dependent manner.

### RhpR positively regulated the expression of *rpoD* in KB but had no effect on thermotolerance.

RhpR bound to the promoter region of *rpoD* in KB (see Fig. S5A in the supplemental material), which was confirmed by an EMSA *in vitro* (Fig. S5B). The RpoD protein is the primary sigma factor during exponential growth and preferentially transcribes genes associated with fast growth (48). Inactivation of *rpoD* affects the heat shock response of bacteria (49). As shown in Fig. S5C and D, the transcription level of *rpoD* was induced by ∼3-fold from the expression of RhpR when cultured in KB but not in MM. These results were confirmed by corresponding *lux*-reporter assays (Fig. S5E to H). We then tested the thermotolerance in these strains and found no significant difference (data not shown). In sum, these results suggest that RhpR positively regulated the expression of *rpoD* in a KB-dependent manner but had no effect on thermotolerance.

10.1128/mBio.02838-18.5 FIG S5 RhpR upregulates the expression of *rpoD* in KB. RhpR directly upregulates the expression of *rpoD*. (A) RhpR binds to the promoter regions of *rpoD* in KB but not in MM according to the ChIP-seq results. (B) RhpR binds with promoter regions of *rpoD.* PCR products containing the *rpoD* promoter sequence were added to the EMSA reaction mixtures at 50 nM each. (C) RT-qPCR reveals that RhpR enhances the expression of *rpoD*. The wild-type strain, Δ*rhpS* strain, and Δ*rhpS* strain carrying the pHM1-*rhpS* plasmid were grown in KB and induced in MM for 6 h. RT-qPCR was performed to measure the transcription level of *rpoD* in all three strains. (D) RT-qPCR shows that RhpR enhances the expression of *rpoD*. pHM1-RhpR or pHM1 empty vector was transformed into the Δ*rhpRS* strain. RT-qPCR was performed to measure the transcription level of *rpoD*. (E and F) RhpR directly promotes the expression of *rpoD* in KB. *rpoD*-*lux* reporter plasmid was transformed into wild-type *P. savastanoi* pv. *phaseolicola* 1448A, the Δ*rhpS* strain, and the complemented strain. A single colony was cultured in KB until it reached an OD_600_ of 0.6 and then transferred into MM. Luciferase gene (*lux*) activities were measured over 6 h. (G and H) Regulation of *rpoD* promoters by RhpR in the Δ*rhpRS* strain. Activities of *rpoD-lux* were introduced into the Δ*rhpRS* strain and Δ*rhpRS* strain carrying the pHM1-*rhpR* plasmid. The bacteria were grown in KB and induced in MM before measurement of *lux* activities. Download FIG S5, TIF file, 0.8 MB. Copyright © 2019 Xie et al. 2019 Xie et al. This content is distributed under the terms of the Creative Commons Attribution 4.0 International license.

10.1128/mBio.02838-18.6 FIG S6 IR elements are required for the direct regulation by RhpR of *flhA*, *fimA*, *algD*, PSPPH_2590, *ccmA*, and *adhB*. (A and B) Regulation of *flhA*-ΔIR promoters by RhpR in the Δ*rhpRS* strain. *flhA*-ΔIR-*lux* was introduced into the Δ*rhpRS*, Δ*rhpRS*/p-*rhpR*, Δ*rhpRS*/p-*rhpR*^D70A^, and Δ*rhpRS*/p-*rhpR*^D70E^ strains. (C and D) Regulation of *fimA*-ΔIR promoters by RhpR in the Δ*rhpRS* strain. *fimA*-ΔIR-*lux* was introduced into the Δ*rhpRS*, Δ*rhpRS*/p-*rhpR*, Δ*rhpRS*/p-*rhpR*^D70A^, and Δ*rhpRS*/p-*rhpR*^D70E^ strains. (E and F) Regulation of *algD*-ΔIR promoters by RhpR in the Δ*rhpRS* strain. *algD*-ΔIR-*lux* was introduced into the Δ*rhpRS*, Δ*rhpRS*/p-*rhpR*, Δ*rhpRS*/p-*rhpR*^D70A^, and Δ*rhpRS*/p-*rhpR*^D70E^ strains. (G and H) Regulation of PSPPH_2590-ΔIR promoters by RhpR in the Δ*rhpRS* strain. PSPPH_2590-ΔIR-*lux* was introduced into the Δ*rhpRS*, Δ*rhpRS*/p-*rhpR*, Δ*rhpRS*/p-*rhpR*^D70A^, and Δ*rhpRS*/p-*rhpR*^D70E^ strains. (I and J) Regulation of *ccmA*-ΔIR promoters by RhpR in the Δ*rhpRS* strain. *ccmA*-ΔIR-*lux* was introduced into the Δ*rhpRS* and Δ*rhpRS*/p-*rhpR* strains. (K and L) Regulation of *adhB*-ΔIR promoters by RhpR in the Δ*rhpRS* strain. *adhB*-ΔIR-*lux* was introduced into the Δ*rhpRS* and Δ*rhpRS*/p-*rhpR* strains. All strains were grown in KB to an OD_600_ of 0.6 and induced in minimal medium (MM) for 0, 2, 4, 6, 8, 10, and 12 h before measurement of luciferase gene (*lux*) activities. *, *P* < 0.05, **, *P* < 0.01, and ***, *P* < 0.001, compared to the Δ*rhpS* or Δ*rhpRS* strains by Student’s *t* test. Each experiment was performed three times. Error bars represent standard error. Download FIG S6, TIF file, 1.7 MB. Copyright © 2019 Xie et al. 2019 Xie et al. This content is distributed under the terms of the Creative Commons Attribution 4.0 International license.

## DISCUSSION

Two-component systems sense signals and rely on the phosphorylation of response regulators to regulate downstream gene expression. Our previous studies have shown that RhpRS is a switch in regulating the T3SS in P. syringae (22, 26). RhpS senses unknown external signal(s) and phosphorylates RhpR, thus activating its own expression and inhibiting *hrpR* expression by binding to the IR element (25, 26). Compared to MM, RhpR regulates 825 more genes in KB, indicating a more important role of RhpR in KB (25). However, the specific effects of environmental conditions and phosphorylation on function of RhpR remain elusive.

Environmental signals are very important for bacteria to survive in changing environments. In the absence of RhpS, we found that the expressions of *ccmA*, *adhB*, *hemB*, *rpoD*, and *trpG* were regulated by RhpR in KB but not in MM, indicating that the functions of RhpR were regulated by the external environment independent of RhpS. This result suggested that RhpR can be phosphorylated by another noncognate sensor kinase in KB when *rhpS* is deleted. Alternatively, unphosphorylated RhpR may bind to the promoter regions of these genes. A well-studied example of robust cross talk between noncognate partners is the cross talk between QseBC and PmrAB in Escherichia coli. Similar to RhpRS, the sensor protein QseC is bifunctional, catalyzing both the phosphorylation and dephosphorylation of QseB (50). In the wild-type strain, QseC dephosphorylates QseB, while the addition of ferric iron in the medium leads to the phosphorylation of QseB by phosphorylated PmrB (51). In the absence of QseC, PmrB cannot dephosphorylate QseB, leading to increased levels of active QseB and compromised virulence (51). However, the presence of other noncognate kinases that phosphorylate RhpR needs to be explored.

The link between metabolism and virulence has been reported in a group of bacteria. In Vibrio cholerae, the NADH:ubiquinone oxidoreductase complex affects the expression of the virulence regulatory protein ToxT via respiration activity (52). A study on Yersinia pseudotuberculosis has shown that deletion of the pyruvate kinase gene (*pykF*) significantly reduces the bacterial virulence in an oral mouse infection model (53). We found that some KB-dependent RhpR-regulated genes not only were involved in metabolic pathways but also had certain effects on virulence. In Xanthomonas campestris, the *c*-type cytochromes contribute to the EPS production and extracellular enzyme activities to enhance virulence (54). In Staphylococcus aureus, HemB leads to the production of α-hemolysin, protein A, and thermonuclease to maintain virulence (55, 56). Therefore, RhpR might indirectly regulate bacterial virulence via tuning *hemB* and *ccmA*.

RhpR-P efficiently bound to the promoter regions of *hrpR*, *hopR1*, *algD*, *flhA*, *fimA*, PSPPH_2590, and PSPPH_2653. It also directly controlled a series of pathogenic phenotypes, including the T3SS, swimming mobility, and EPS and biofilm production. At the same time, RhpR-P contributed to virulence by enhancing twitching, repressing the c-di-GMP concentration, and promoting LPS production (31, 57, 58). The crystal structure of the unphosphorylated response regulator StyR in Pseudomonas fluorescens indicates that phosphorylation acts as an allosteric switch, shifting a preexisting StyR equilibrium toward the active, dimeric, DNA binding form (59). RhpR might therefore enhance its binding affinity with IR elements by using a similar allosteric switch, thus regulating downstream genes.

The RNA-seq analysis identified 474 and 840 new genes regulated by RhpS in KB and MM, respectively. These newly identified genes are known to be involved in transmembrane transportation, oxidative phosphorylation, sensing metal ions, general secretion pathways, and gene transcription, indicating that RhpR has a wider range of functions than have been previously discovered. A number of genes involved in the cell envelope and virulence were also identified in the RhpR regulon, such as for flagellar biosynthesis (*fliO*, *fliL*, *fliE*, *fliS*, and *fliG*), alginate biosynthesis (*algA*, *algF*, *algC*, *algK*, and *algD*), and type IV pilus biogenesis (*pilF*, *pilG*, and *pilH*). These identified genes emphasize the direct link between RhpR and pathogenicity. Meanwhile, 287 and 109 differentially expressed genes were identified as part of the RhpRS regulon in KB and MM, respectively, and included genes involved in chemotaxis (*cheR*, *cheW*, *cheZ*, and *cheA*), drug resistance (PSPPH_2378, PSPPH_3554, and PSPPH_3553), DNA stability (*baeS1*, *topB1*, and *radA*), and c-di-GMP production (PSPPH_0499). In sum, our RNA-seq results indicate that RhpR is central to the signaling network of *P. savastanoi*.

**FIG 8:**
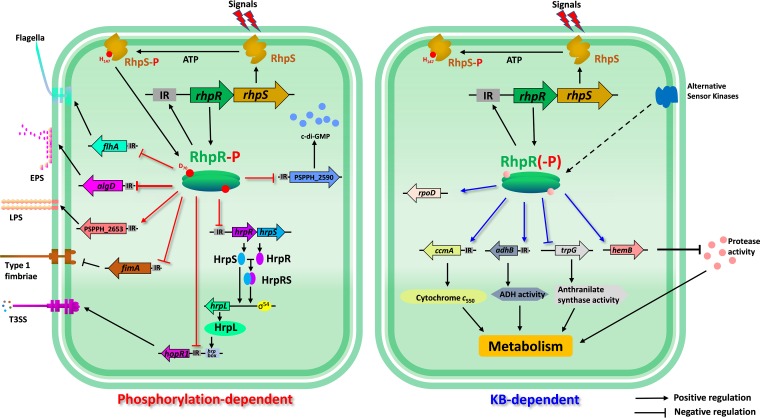
Schematic model of KB and phosphorylation-dependent RhpR regulation. Shown is a schematic diagram of RhpR involved in virulence factor and metabolism regulation of *P. savastanoi.* As a key TCS for regulating T3SS, histidine kinase RhpS phosphorylates RhpR by receiving an unknown signal. The transcription of the *rhpRS* operon is activated by phosphorylated RhpR. RhpR-P directly suppresses the *hrpRS*-*hrpL*-T3SS cascade, effector gene *hopR1*, swimming, and biofilm, EPS, and c-di-GMP production, but improves twitching motility and LPS production, thus regulating the pathogenicity of *P. savastanoi*. When cultured in KB, RhpR enhances the production of cytochrome *c*_550_ and alcohol dehydrogenase activity, while it negatively regulates protease activity and anthranilate synthase activity. Strikingly, the regulatory functions of RhpR found in KB are all significantly reduced or even disappear in MM, indicating the presence of other kinase(s) or regulator(s) that regulate RhpR under the KB condition.

Taken together, our results suggest a model for KB-dependent or phosphorylation-dependent regulation of RhpR (Fig. 8). The function of RhpR was regulated by external signals and phosphorylation state, which enabled switching of the regulatory functions of RhpR. Under nutrient-rich conditions, RhpR directly regulated multiple metabolic pathways, including cytochrome *c*_550_, alcohol dehydrogenase, anthranilate synthase, and protease. Meanwhile, the phosphorylation of RhpR determined its ability to bind to IR motifs and then exert its regulatory effects. RhpR depended on the phosphorylation of Asp70 to bind to the promoter regions of *hrpR*, *hopR1*, *flhA*, *fimA*, and *algD*, and thus it directly regulated the virulence-related phenotype associated with the cell envelope, such as the T3SS, swimming, twitching, biofilm, and EPS and LPS production. The RhpRS orthologues were widely present in various bacterial species, suggesting that the molecular regulatory mechanisms are conserved in the bacterial kingdom.

## MATERIALS AND METHODS

### Bacterial strains, plasmids, primers, and growth conditions.

The bacterial strains, primers, and plasmids used in this study are listed in Table S2 in the supplemental material. The *P. savastanoi* pv. *phaseolicola* 1448A strains used in this study were the wild type and the Δ*rhpS*, Δ*hrpS*, and Δ*rhpRS* strains. The *P. savastanoi* pv. *phaseolicola* 1448A strain was grown in KB medium (60) at 28°C until it reached an optical density at 600 nm (OD_600_) of 2.0 to 2.5. Then the bacteria were centrifuged and washed twice with MM [50 mM KH_2_PO_4_, 7.6 mM (NH_4_)_2_SO_4_, 1.7 mM MgCl_2_, 1.7 mM NaCl, and 10 mM fructose, pH 6.0] (5, 61) and cultured at OD_600_ of 0.2 in MM for 6 h before measurement of *lux* activity or extraction of RNA. The following antibiotic concentrations were used: rifampin, 25 μg/ml; kanamycin, 100 μg/ml; and spectinomycin, 100 μg/ml. The E. coli BL21(DE3) strain was grown in LB medium at 37°C. The antibiotic kanamycin was used at a concentration of 50 μg/ml.

10.1128/mBio.02838-18.8 TABLE S2 Bacterial strains, plasmids, and primers used in this study. The strains, plasmids, and primers used in this article are listed. Download Table S2, DOCX file, 0.03 MB. Copyright © 2019 Xie et al. 2019 Xie et al. This content is distributed under the terms of the Creative Commons Attribution 4.0 International license.

10.1128/mBio.02838-18.9 TABLE S3 ChIP-seq data of RhpR binding sites. (A) ChIP-seq result for RhpR binding sites in wild-type Pseudomonas savastanoi pv. *phaseolicola* 1448A in KB. Listed are all binding regions that were enriched by RhpR comparing HA-RhpR to the empty vector in KB. The headings “start_position” and “end_position” indicate the locations of RhpR binding regions. (B) ChIP-seq result for RhpR binding sites in wild-type P. savastanoi pv. *phaseolicola* 1448A in MM. Listed are all regions of the genome significantly enriched by RhpR comparing HA-RhpR to the empty vector in MM. (C) ChIP-seq result for RhpR binding sites in the P. savastanoi pv. *phaseolicola* 1448A *rhpS* mutant in KB. Listed are all binding regions that were enriched by RhpR comparing HA-RhpR to the empty vector in KB. (D) ChIP-seq result for RhpR binding sites in the P. savastanoi pv. *phaseolicola* 1448A *rhpS* mutant in MM. Listed are all binding regions that were enriched by RhpR comparing HA-RhpR to the empty vector in MM. (E) ChIP-seq result for RhpR^D70A^ binding sites in the P. savastanoi pv. *phaseolicola* 1448A *rhpS* mutant in KB. Listed are all regions of the genome significantly enriched by RhpR^D70A^ comparing HA-RhpR to the empty vector in KB. (F) ChIP-seq result for RhpR^D70A^ binding sites in the P. savastanoi pv. *phaseolicola* 1448A *rhpS* mutant in MM. Listed are all regions of the genome significantly enriched by RhpR^D70A^ comparing HA-RhpR^D70A^ to the empty vector in MM. (G) Phosphorylation-dependent RhpR binding sites. Listed are all regions of the genome significantly enriched by RhpR comparing the binding peaks between RhpR and RhpR^D70A^. (H) KB-dependent RhpR binding sites. Listed are all regions of the genome significantly enriched by RhpR comparing the RhpR binding sites in KB and MM. Download Table S3, DOCX file, 0.3 MB. Copyright © 2019 Xie et al. 2019 Xie et al. This content is distributed under the terms of the Creative Commons Attribution 4.0 International license.

10.1128/mBio.02838-18.10 TABLE S4 RNA-seq data. (A) List of genes upregulated in the *rhpS* mutant in KB. Listed are all genes whose expression is significantly upregulated comparing the wild-type *P. savastanoi* pv. *phaseolicola* 1448A strain to the *rhpS* mutant. Negative log_2_ fold change indicates that the expression was lower in the wild-type strain than the *rhpS* mutant (activated by RhpR). (B) List of genes downregulated in *rhpS* mutant in KB. Listed are all genes whose expression is significantly downregulated comparing the wild-type strain to the *rhpS* mutant. Positive log_2_ fold change indicates that the expression was higher in the wild-type strain than the *rhpS* mutant (suppressed by RhpR). (C) List of genes upregulated in the *rhpS* mutant in MM. Listed are all genes whose expression is significantly upregulated comparing the wild-type strain to the *rhpS* mutant. Negative log_2_ fold change indicates that the expression was lower in the wild-type strain than the *rhpS* mutant (activated by RhpR). (D) List of genes downregulated in the *rhpS* mutant in MM. Listed are all genes whose expression is significantly downregulated comparing the wild-type strain to the *rhpS* mutant. Positive log_2_ fold change indicates that the expression was higher in the wild-type strain than the *rhpS* mutant (suppressed by RhpR). (E) List of genes upregulated in the *rhpRS* mutant in KB. Listed are all genes whose expression is significantly upregulated comparing the wild-type strain to the *rhpRS* mutant. Negative log_2_ fold change indicates that the expression was lower in the wild-type strain than the *rhpRS* mutant. (F) List of genes downregulated in the *rhpRS* mutant in KB. Listed are all genes whose expression is significantly downregulated comparing the wild-type strain to the *rhpRS* mutant. Positive log_2_ fold change indicates that the expression was higher in the wild-type strain than the *rhpRS* mutant. (G) List of genes upregulated in the *rhpRS* mutant in MM. Listed are all genes whose expression is significantly upregulated comparing the wild-type strain to the *rhpRS* mutant. Negative log_2_ fold change indicates that the expression was lower in the wild-type strain than the *rhpRS* mutant. (H) List of genes downregulated in the *rhpRS* mutant in MM. Listed are all genes whose expression is significantly downregulated comparing the wild-type strain to the *rhpRS* mutant. Positive log_2_ fold change indicates that the expression was higher in the wild-type strain than the *rhpRS* mutant. Download Table S4, DOCX file, 1.1 MB. Copyright © 2019 Xie et al. 2019 Xie et al. This content is distributed under the terms of the Creative Commons Attribution 4.0 International license.

### Construction of the Δ*rhpS* and Δ*rhpRS* deletion mutants in *P. savastanoi* pv. *phaseolicola* 1448A.

*rhpS-*Up-F/R and *rhpRS-*Up-F/R were used to amplify 1-kb fragments upstream of *rhpS* and the *rhpRS* operon, while *rhpS*-Down-F/R and *rhpRS*-Down-F/R (Table S2) were used to amplify 1.4-kb fragments downstream of *rhpS* and *rhpRS*, respectively. The PCR products were purified and digested with BamHI and then linked by T4 ligase. The linked fragments were cloned into a pK18 suicide plasmid to construct the Δ*rhpS* and Δ*rhpRS* strains (62). Next, the constructed vectors were transformed into the *P. savastanoi* pv. *phaseolicola* 1448A wild-type strain in the KB plate with 25 μg/ml rifampin and 100 μg/ml kanamycin. The single colonies were picked to a sucrose plate and then cultured in both KB with kanamycin and rifampin and KB with rifampin alone. Loss of kanamycin resistance indicated a double crossover. Finally, the Δ*rhpS* and Δ*rhpRS* mutants were verified by PCR using primers *rhpS*-ORF-F/R and *rhpRS*-ORF-F/R (Table S2).

### ChIP-seq.

The ChIP assay was performed as previously described (63). An empty pHM2, pHM2-*rhpR_psph_*-HA, or pHM2-*rhpR_psph_*_(D70A)_-HA plasmid was transformed into the wild-type or Δ*rhpS* strain and then cultured in KB medium until it reached an OD_600_ of 0.6 before transfer to MM liquid medium for 6 h. The strains were treated with 1% formaldehyde for 10 min at 37°C. Cross-linking was stopped by the addition of 125 mM glycine. The bacteria were centrifuged, and the pellets were washed twice with Tris-buffer (20 mM Tris-HCl [pH 7.5], 150 mM NaCl) and then resuspended in 500 μl immunoprecipitation (IP) buffer (50 mM HEPES-KOH [pH 7.5], 1 mM EDTA, 150 mM NaCl, 1% Triton X-100, 0.1% sodium deoxycholate, 0.1% SDS, mini-protease inhibitor cocktail). Next, the DNA was subjected to sonication to produce 100- to 300-bp DNA fragments. The insoluble cellular debris was removed by centrifugation at 4°C, and the supernatant was saved as the input sample in the IP experiments. Both the control and IP samples were washed with protein A beads (General Electric) and mixed with 50 μl agarose-conjugated anti-hemagglutinin (anti-HA) antibodies (Sigma) in IP buffer. The following washing, cross-link reversal, and purification of ChIP-DNA steps were conducted as previously described (63). DNA fragments (150 to 250 bp) were selected for library construction, and libraries were constructed by using the NEXTflex ChIP-seq kit (Bioo Scientific). The libraries were sequenced using the HiSeq 2000 system (Illumina). ChIP-seq results were mapped to the Pseudomonas savastanoi pv. *phaseolicola* 1448A genome (NC_005773.3) by using Bowtie (version 1.2.1.1). Only the uniquely mapped reads were kept for the subsequent analyses. Binding peaks (*P* < 1e−5) were identified using MACS software (version 2.1.0). All experiments had two repeats, and the reported peaks were found in both experiments.

### RNA-seq analysis.

The wild-type, Δ*rhpS*, and Δ*rhpRS* strains were cultured in KB medium until they reached an OD_600_ of 0.6 before being transferred to liquid MM for 6 h. Then, 2 ml of bacterial cultures was collected by centrifugation (12,000 rpm at 4°C). RNA purification was conducted with an RNeasy minikit (Qiagen). After removal of rRNA by using the MICROBExpress kit (Ambion), mRNA was used to generate the cDNA library according to the NEBNext UltraTM II RNA Library Prep kit protocol (NEB), which was then sequenced using the HiSeq 2000 system (Illumina). Bacterial RNA-seq reads were mapped to the Pseudomonas savastanoi pv. *phaseolicola* 1448A genome (NC_005773.3) by using STAR. Only the uniquely mapped reads were kept for the subsequent analyses. The gene differential expression analysis was performed using Cuffdiff software (version 2.0.0) (64). GO enrichment analyses were conducted on all differentially transcribed genes using DAVID (65). Each sample analysis was repeated twice.

### Protein expression and purification.

The open reading frame (ORF) that encodes RhpR protein was amplified by PCR from Pseudomonas savastanoi pv. *phaseolicola* 1448A genomic DNA. The RhpR^D70A^ and RhpR^D70E^ protein ORFs were generated by overlap PCR. The PCR products were digested and ligated to pET28a (BamHI/XhoI), which has a His_6_ tag at its N terminus. pET28a-*rhpR*, pET28a-*rhpR*^D70A^, and pET28a-*rhpR*^D70E^ were then transformed into the E. coli strain BL21 Star(DE3). Briefly, a selected single colony was cultured in LB medium overnight, the culture was transferred into 1 liter LB medium, and the cells were grown at 37°C at 220 rpm to an OD_600_ of 0.6. Then 0.5 mM IPTG (isopropyl β-d-1-thiogalactopyranoside) was added to the culture to induce protein expression at 16°C for 16 h. The culture was centrifuged at 4°C at 5,000 rpm for 5 min to harvest the bacteria. The pellet was suspended in 20 ml buffer A (500 mM NaCl, 25 mM Tris-HCl [pH 7.4], 5% glycerol, 1 mM dithiothreitol, 1 mM phenylmethylsulfonyl fluoride [PMSF]). The cells were lysed with sonication and centrifuged at 4°C (12,000 rpm for 30 min). The supernatant was filtered with a 0.45-μm-pore filter, and the filtrate was added into a Ni-nitrilotriacetic acid (NTA) column (Bio-Rad) that had been equilibrated with buffer A before use. After the Ni-NTA column was washed five times with buffer A, the column was eluted with a 30-ml gradient of 100 to 500 mM imidazole prepared in buffer A, respectively. Fractions were collected, and sodium dodecyl sulfate-polyacrylamide gel electrophoresis (SDS-PAGE) was used to verify the molecular weight of target protein. Proteins were concentrated by centrifugation (Millipore) at 4°C and then supplemented with 20% glycerol and stored at −80°C.

### EMSAs.

DNA probes were PCR amplified using primers listed in Table S2. The IR deletion probes were prepared by using overlap PCR. The probe (40 ng) was mixed with various amounts of proteins in 20 μl of gel shift buffer (10 mM Tris-HCl [pH 7.4], 50 mM KCl, 5 mM MgCl2, 10% glycerol). After incubation at room temperature for 20 min, the samples were analyzed by 6% polyacrylamide gel electrophoresis (90 V for 45 min for sample separation). The gels were subjected to DNA dye for 5 min and photographed by using a gel imaging system (Bio-Rad). The assay was repeated at least three times with similar results.

### RT-qPCR.

For real-time quantitative PCR (RT-qPCR) analysis, all *P. savastanoi* strains were grown at 28°C with shaking at 220 rpm until they reached an OD_600_ of 0.6. To harvest the bacteria, the cultures were centrifuged as pellets at 8,000 rpm for 1 min. RNA purification was performed by using the RNeasy minikit (Qiagen). RNA concentration was measured by Nanodrop 2000 spectrophotometer (Thermo Fisher). The cDNA synthesis was performed by using a FastKing RT kit (Tiangen Biotech). RT-qPCR was performed by SuperReal Premix Plus (SYBR green) kit (Tiangen Biotech) and prepared according to the instructions of the manufacturer. Each reaction was performed in triplicate in 25-μl reaction volumes with 800 ng cDNA and 16S rRNA as the internal control. For each reaction, 200 nM concentrations of primers (Table S2) were used for RT-qPCR. The reactions were run at 42°C for 15 min and 95°C for 3 min and then kept at 4°C until used. The fold change represents the relative expression level of mRNA, which can be estimated by the threshold cycle (*C_T_*) values of 2^−ΔΔ^*^CT^*. All reactions were conducted with three repeats.

### Luminescence screening assays.

Expression of *lux*-based reporters from bacteria grown in liquid culture was measured as counts per second (cps) of light production. The *lux* reporters were transformed into Pseudomonas savastanoi pv. *phaseolicola* strains. The resulting strains were cultured to an OD_600_ of 0.6 in KB at first. Then the cultures were washed twice with MM and incubated in MM with shaking for measurement of Lux activities. Promoter activities were measured every 2 h for 12 h. Bacterial growth was monitored at the same time by measuring the OD_600_ in a Synergy 2 plate reader (BioTek).

### Motility assays.

Motilities were assayed using various media (66, 67). For swimming motility, a single colony was grown in KB liquid overnight, transferred at 1:100 to 2 ml fresh KB medium, and then grown at 28°C until it reached an OD_600_ of 0.6. Two microliters of the cultures was spotted onto soft KB plates (0.3% agar). The plates were incubated at 28°C for 48 h. For twitching ability, the 2-μl aliquots were inoculated in the center of KB plates (1% agar) and incubated at 25°C. The surface motility was observed after 48 h.

### Congo red assay and biofilm formation assay.

The Congo red assay was performed as previously described (68), with minor modifications to measure the production of exopolysaccharide. The overnight culture was diluted to an OD_600_ of 0.001 in KB, and 2 μl of the diluted culture was spotted onto the surface of the Congo red plates and grown at 28°C. The colony morphology and staining were recorded after 3 days.

Biofilm formation was detected in a modified static system as previously described (69). Visualization of biofilm formation was performed in 15-ml borosilicate tubes. Briefly, bacteria from overnight cultures were inoculated at 1:100 dilutions into KB medium supplemented with corresponding antibiotics and grown at 30°C for 96 h. Crystal violet (CV [0.1%]) was used to stain biofilm adhered to the tubes, and unbound dye was washed with distilled water. Quantification of biofilm formation was carried out in transparent 24-well polystyrene plates. Medium and corresponding antibiotics were inoculated to a final OD_600_ of 0.01. The plates were incubated for 16 h at 30°C, and the OD_600_ was measured. CV was added to each well, and cells were stained for 15 min. Wells were rinsed three times in distilled water, and the remaining CV was dissolved in 1 ml of 95% ethanol with shaking. A 100-μl concentration of this solution was transferred to a new transparent polystyrene 96-well plate, and the absorbance was detected at 590 nm. OD_590_/OD_600_ was used to represent the final biofilm production.

### Lipopolysaccharide extraction and quantification.

The bacteria were grown overnight in KB at 28°C, and a 5-ml aliquot of a suspension of bacteria was used to isolate LPS according to the instructions of the the manufacturer of the LPS extraction kit (iNtRON Biotechnology). For quantitative analysis of extracted LPS, 2 g of anthrone was freshly dissolved in 1 liter of 80% sulfuric acid. The extracted LPS was added to the anthrone-sulfuric acid solution and then boiled for 15 min and cooled in ice water. The absorbance of the mixture was measured at 620 nm with 10 mM Tris-HCl as a blank control. The fructose solution served as a positive control.

### Assay of cytochrome *c*_550_ concentrations.

Cytochrome *c*_550_ production was determined as previously described (41) with minor modifications. *P. savastanoi* pv. *phaseolicola* 1448A strains were cultured overnight to an OD of 0.6 on medium with choline (a gratuitous inducer of *c*-type cytochromes associated with methylotrophic growth) as a carbon source to maximize the expression of *c*-type cytochrome proteins and then transferred to MM for 6 h. Cells were collected by centrifugation (5,000 rpm for 5 min at 4°C) and washed twice with phosphate-buffered saline (PBS). Next, the cells were resuspended in PBS and sonicated, and the total soluble extracts were adjusted to 15 mg protein per ml. All samples were reduced with sodium dithionite, and the OD_550_ was measured.

### Assay of alcohol dehydrogenase activity.

*P. savastanoi* pv. *phaseolicola* 1448A strains were grown in KB overnight, adjusted to an OD of 0.6 with KB, and then transferred to MM for 6 h. Then the bacteria were collected by centrifugation. The alcohol dehydrogenase activity was measured according to the instructions of the manufacturer of the Micro alcohol dehydrogenase assay kit (Solarbio).

### Assay for protease activity in *P. savastanoi* pv. *phaseolicola* 1448A supernatant.

Proteolytic activities were determined using the insoluble proteolytic substrate Azocoll (Calbiochem) as previously described (70, 71) with minor modifications. *P. savastanoi* pv. *phaseolicola* 1448A strains were grown in KB overnight, adjusted to an OD of 1.0 with KB, and then transferred to MM for 6 h, and then the bacteria were removed by centrifugation (5,000 rpm for 5 min at 4°C). Azocoll (4 mg/ml) was suspended in 100 mM phosphate buffer (pH 7.0) and added in an equal volume to culture supernatant. The mixture was incubated for 2 h at 37°C, and the reaction was stopped by removing the substrate by centrifugation (5,000 rpm for 5 min at 4°C). The absorbance of reaction mixtures was measured at 520 nm. One unit of protease activity was defined as an increase in optical density of 0.001.

### Assay of anthranilate synthase activity.

Anthranilate synthase activity was determined as previously described (72) with minor modifications. *P. savastanoi* pv. *phaseolicola* 1448A strains were grown in KB overnight until they reached an OD_600_ of 0.6 before being transferred to liquid MM for 6 h. The bacteria were removed by centrifugation (5,000 rpm for 5 min at 4°C) and sonicated to obtain soluble cell lysate. The reaction mixture (total volume of 0.5 ml) contained 0.1 M Tris-HCl (pH 7.5), 1 mM barium chorismate, 20 mM l-glutamine, 10 mM MgCl_2_, and 250 μ1 of desalted supernatant of soluble cell lysate. The incubation was started by addition of chorismate. After incubation for 1 h at 30°C, the reaction was stopped by the addition of 125 μ1 of 1 M phosphoric acid. After centrifugation, the samples were analyzed by fluorospectrophotometer. The excitation wavelength of the product is 340 nm, and the emission wavelength is 400 nm.

### Statistical analysis.

Two-tailed Student’s *t* tests were performed using Microsoft Office Excel 2016. Asterisks indicate *P* values (*, *P* < 0.5; **, *P* < 0.01; and ***, *P* < 0.001), and results represent means ± standard deviation (SD). All experiments were repeated at least three times.

### Data availability.

The ChIP-seq data files have been submitted to Gene Expression Omnibus (GEO) and can be accessed through GEO Series accession no. GSE122629. The RNA-seq data sets have been submitted to National Center for Biotechnology Information (NCBI) under accession no. GSE122629.
