# Supplementary figures and images for: Pseudomonas savastanoi Two-Component System RhpRS Switches between Virulence and Metabolism by Tuning Phosphorylation State and Sensing Nutritional Conditions
Source: mBio. 2019 Mar 19;10(2):e02838-18. doi: 10.1128/mBio.02838-18 (PMC6426608; doi:10.1128/mBio.02838-18)

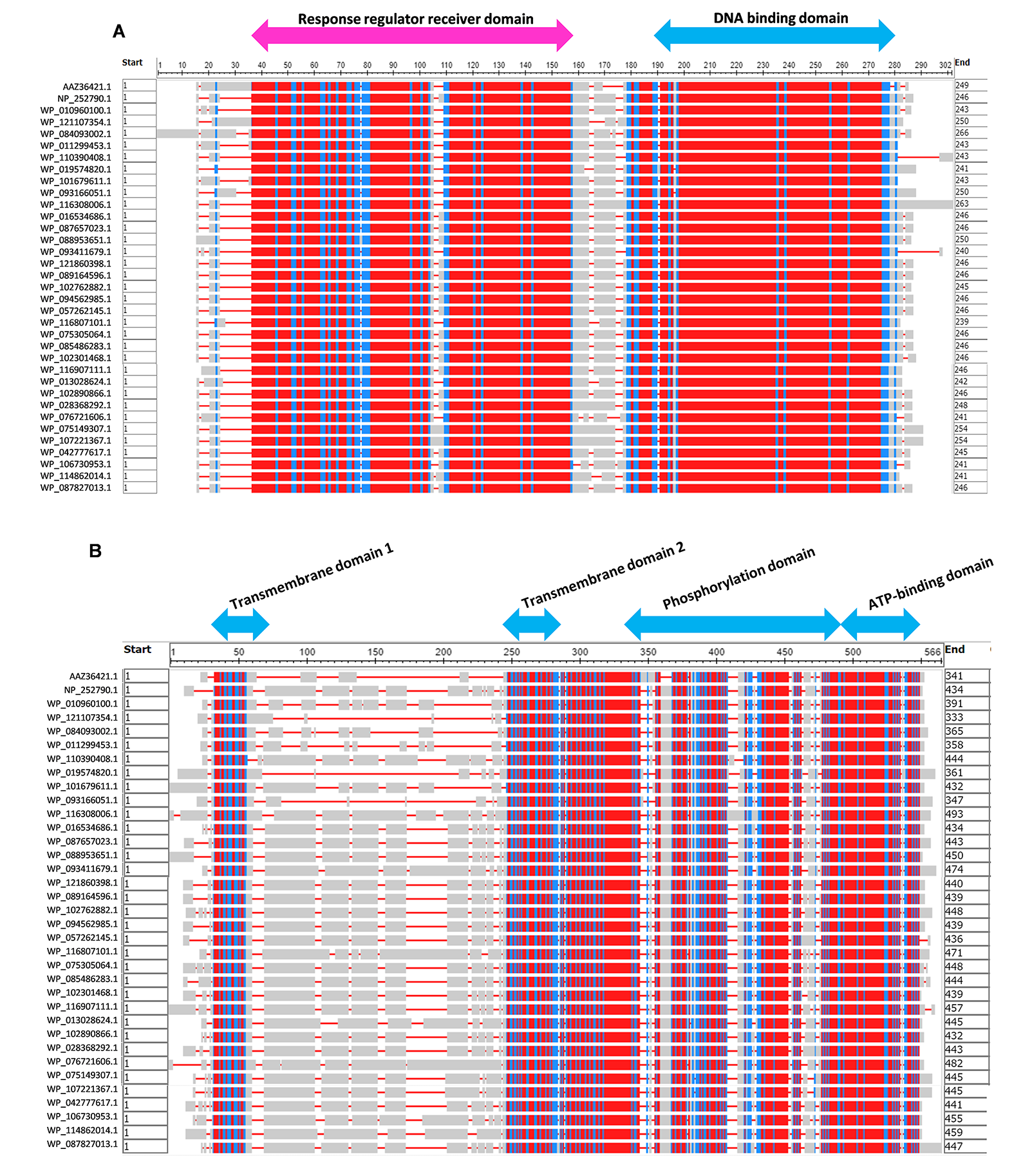

Supplement: FIG S1 [file mBio.02838-18-sf001.tif]

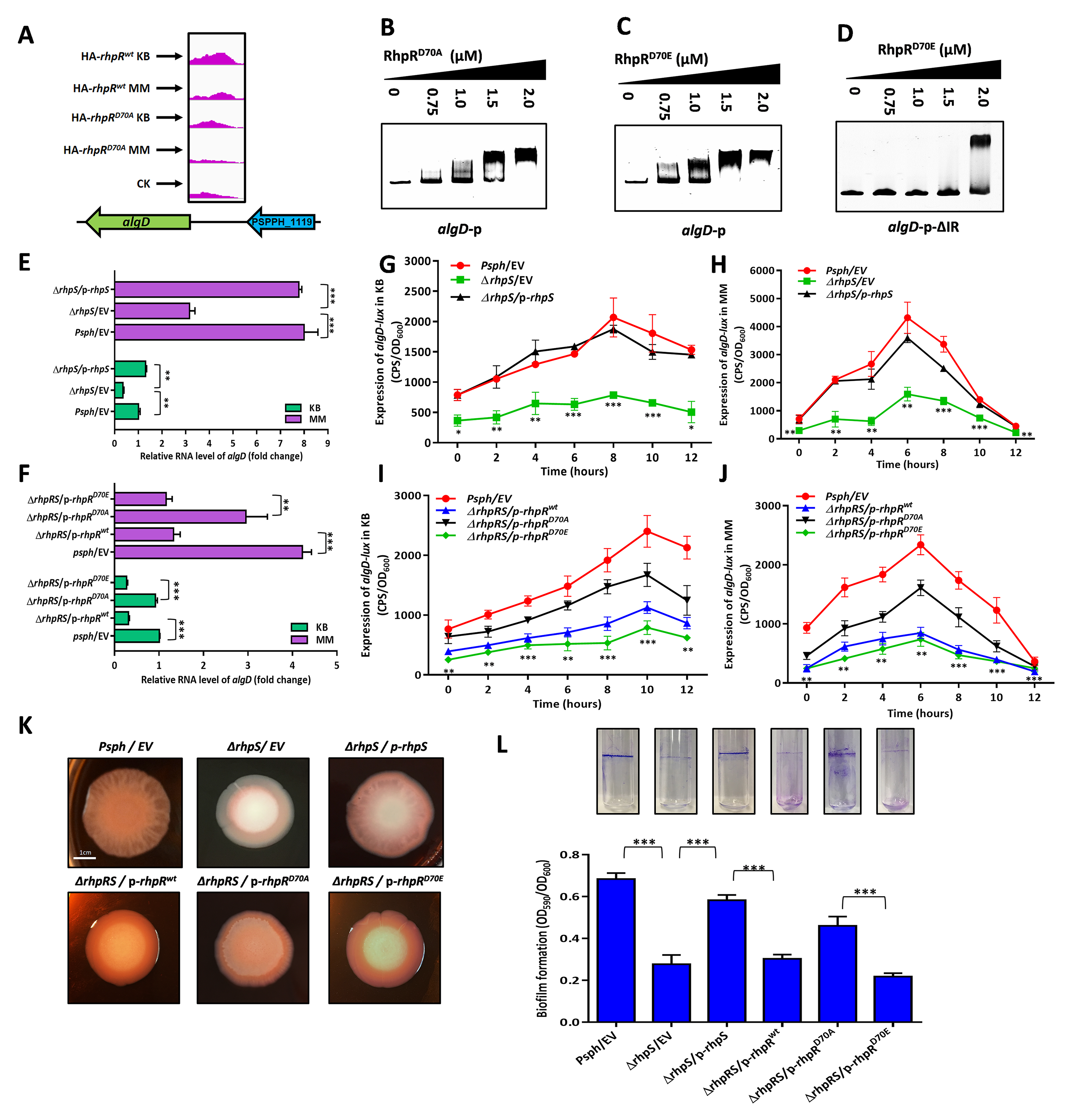

Supplement: FIG S2 [file mBio.02838-18-sf002.tif]

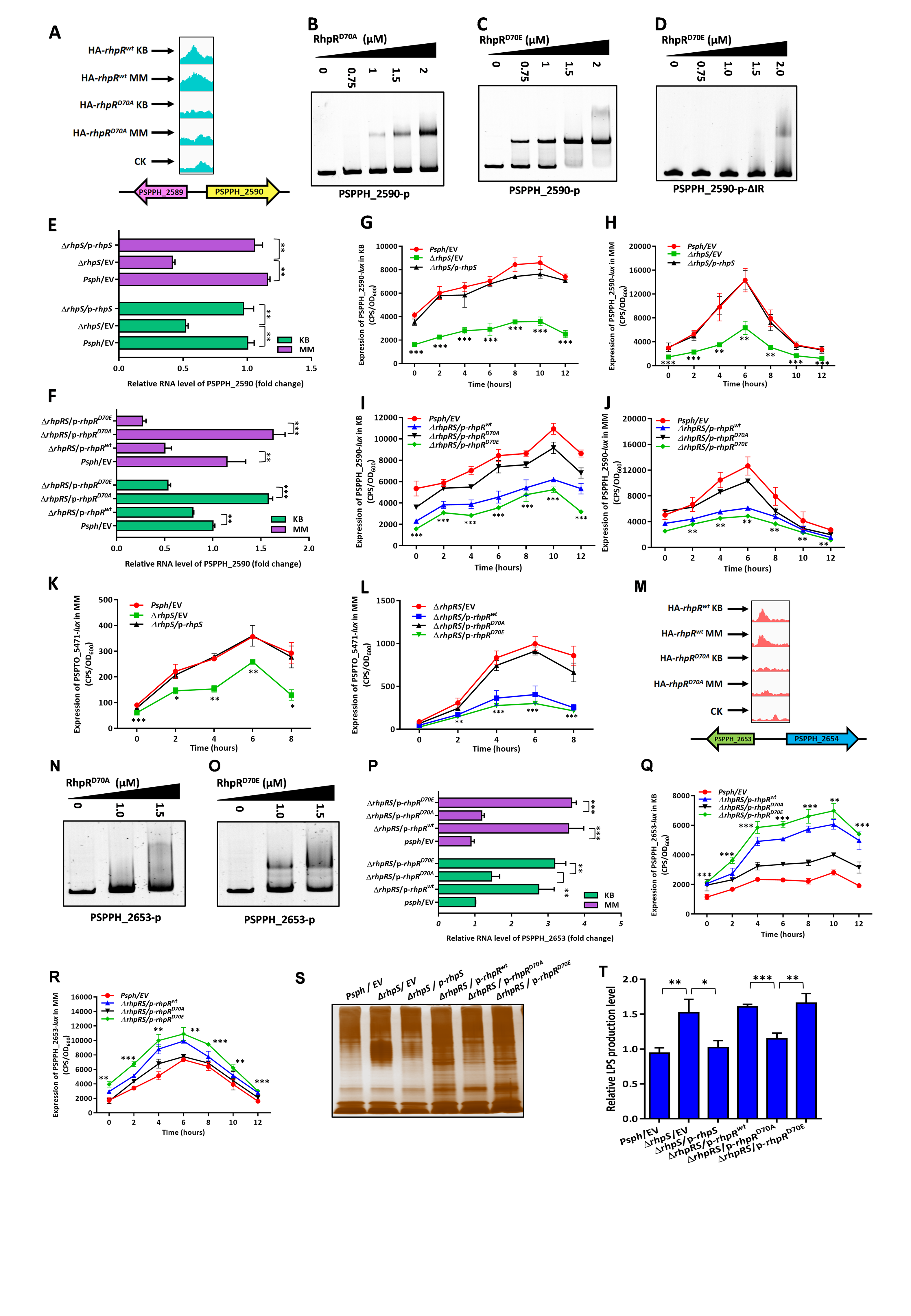

Supplement: FIG S3 [file mBio.02838-18-sf003.tif]

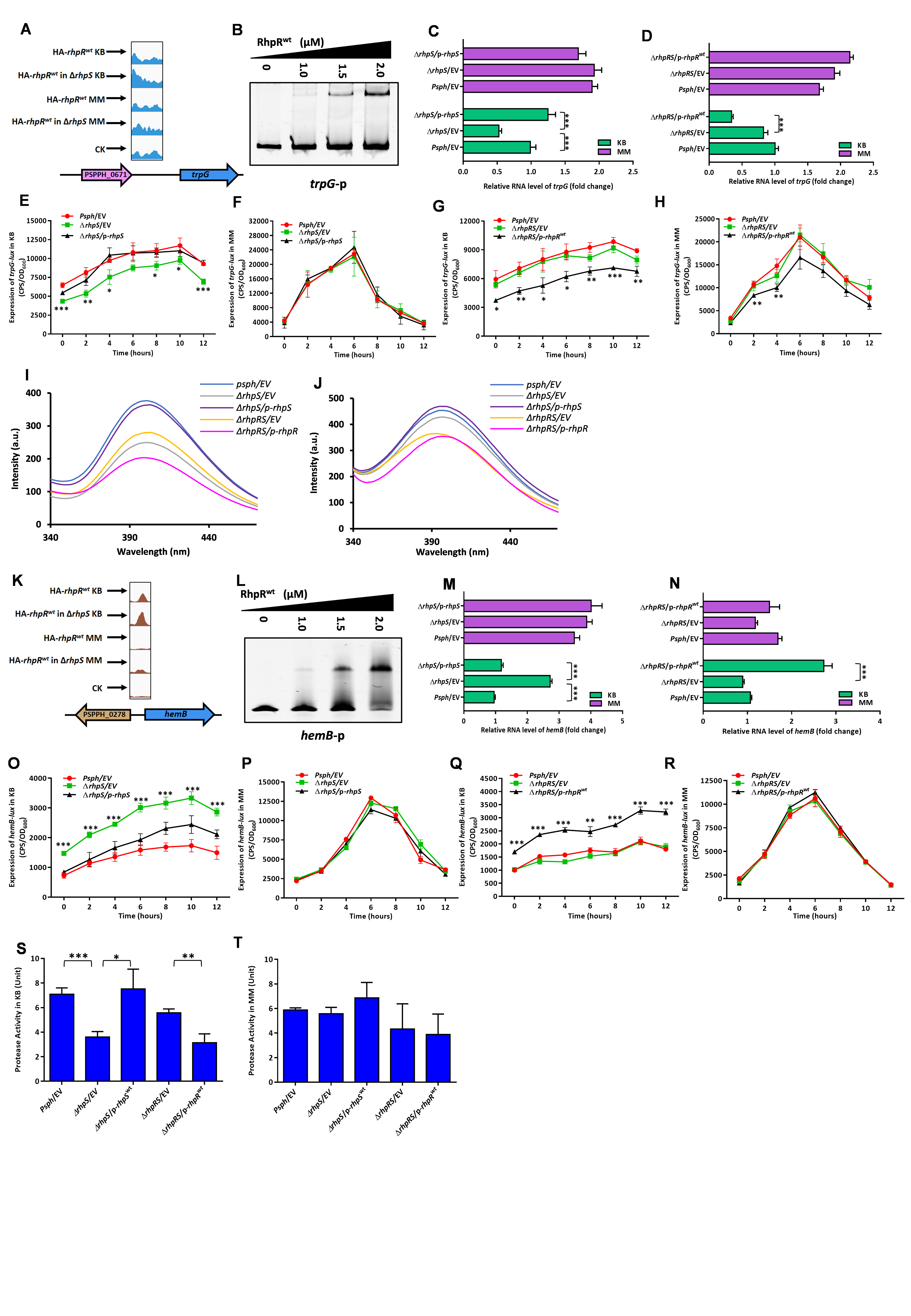

Supplement: FIG S4 [file mBio.02838-18-sf004.tif]

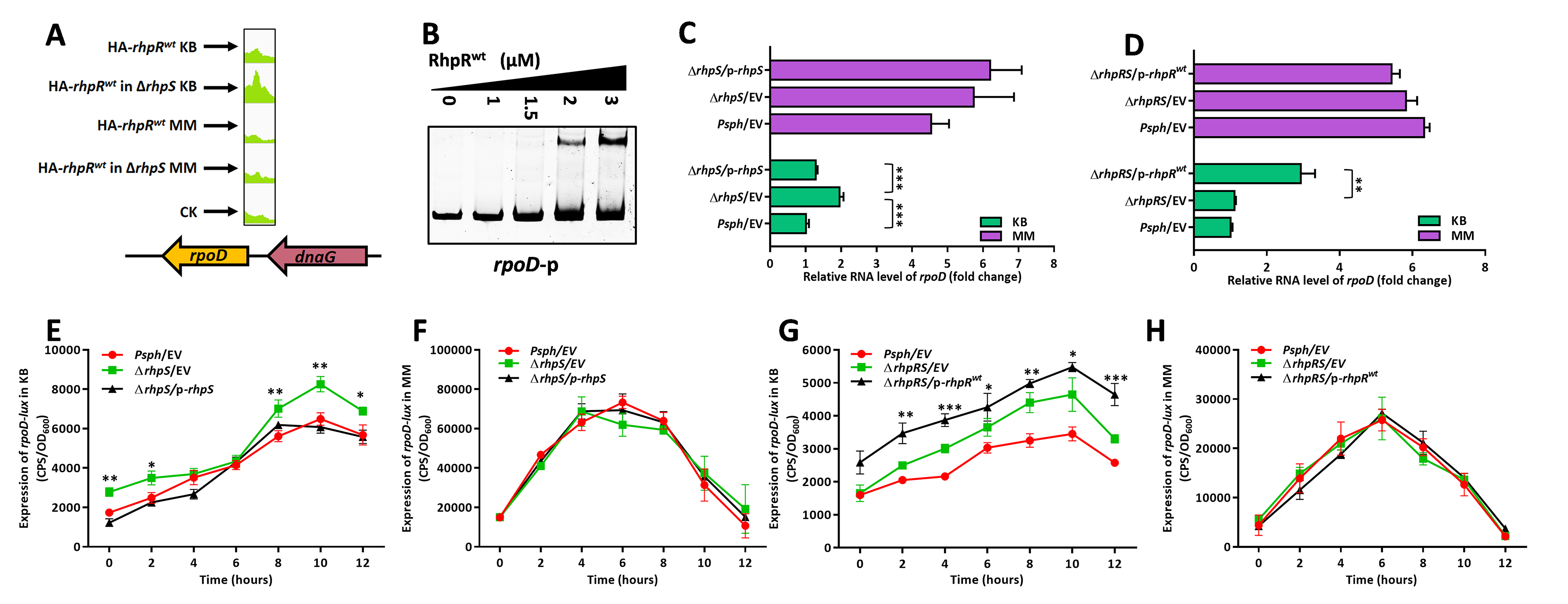

Supplement: FIG S5 [file mBio.02838-18-sf005.tif]

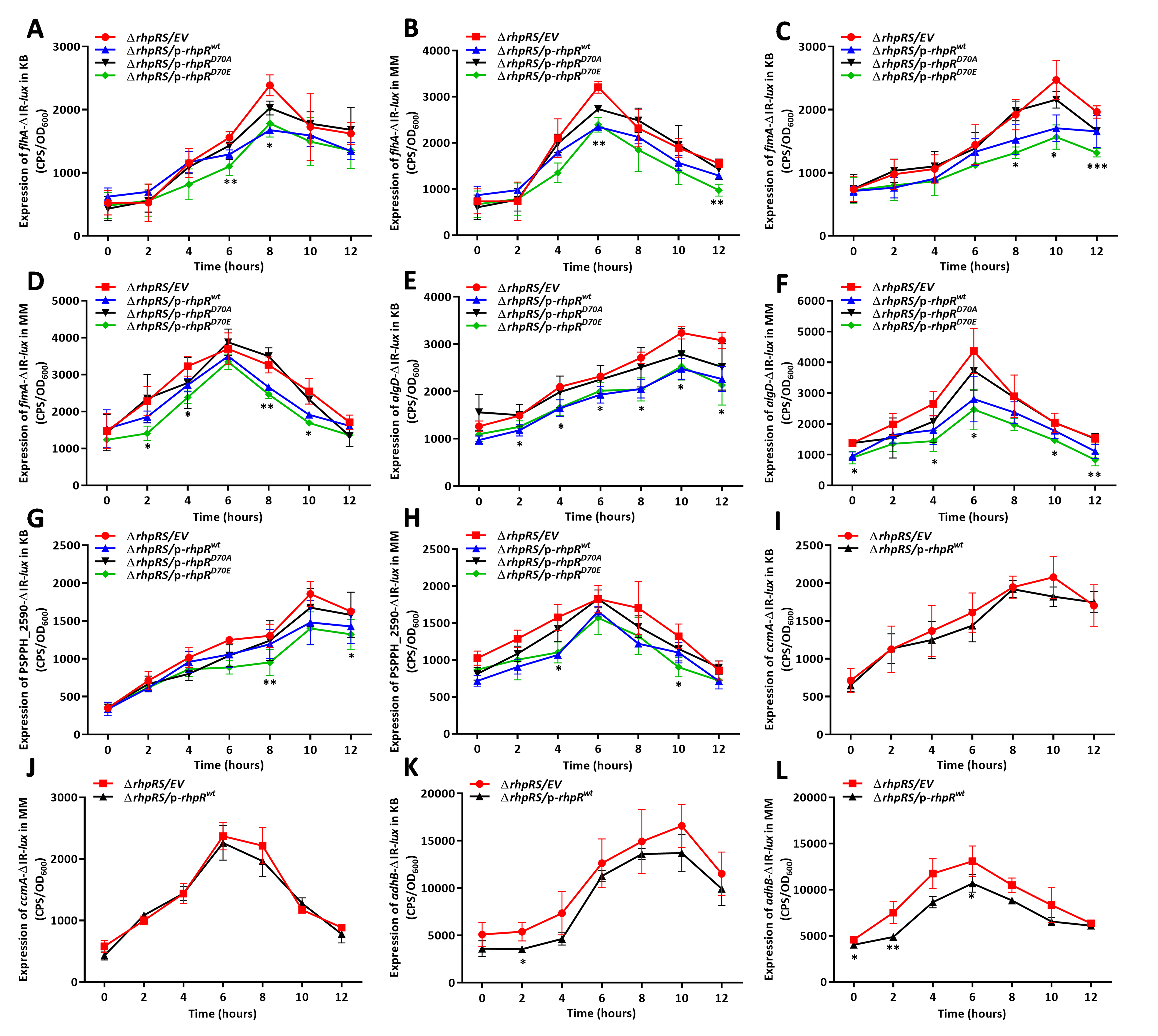

Supplement: FIG S6 [file mBio.02838-18-sf006.tif]
