# Supplementary material for: Pseudomonas savastanoi Two-Component System RhpRS Switches between Virulence and Metabolism by Tuning Phosphorylation State and Sensing Nutritional Conditions
Source: mBio. 2019 Mar 19;10(2):e02838-18. doi: 10.1128/mBio.02838-18 (PMC6426608; doi:10.1128/mBio.02838-18)
Supplement: TABLE S1 [file mBio.02838-18-st001.docx]

**Table S1. RhpR-binding motifs in promoter regions.**

| **Gene** | **IR Sequence** | **Distance to ATG** |
| --- | --- | --- |
| *hrpR* | ATTTC-N6-GATAC | -974 ~ -958 |
| *hopR1* | GTAGA-N6-CCTAT | -90 ~ -74 |
| *flhA* | GCAAC-N6-GATAC | -66 ~ -50 |
| *fimA* | TGATA-N6-GTTAC | -289 ~ -273 |
| *algD* | GTCAC-N6-GCTAC | -553 ~ -537 |
| PSPPH_2590 | GCAGC-N6-GTTAC | -81 ~ -55 |
| *ccmA* | GTGTC-N6-GGTAC | -48 ~ -32 |
| *adhB* | CAAGC-N6-GCTAC | -31 ~ -15 |

**Table S1. RhpR-binding motifs in promoter regions.** Mismatches are underlined in IR sequences.
