# Supplementary material for: Pseudomonas savastanoi Two-Component System RhpRS Switches between Virulence and Metabolism by Tuning Phosphorylation State and Sensing Nutritional Conditions
Source: mBio. 2019 Mar 19;10(2):e02838-18. doi: 10.1128/mBio.02838-18 (PMC6426608; doi:10.1128/mBio.02838-18)
Supplement: TABLE S2 [file mBio.02838-18-st002.docx]

**Table S2. Bacterial strains, plasmids, and primers used in this study.**

| Description | Genotype or relevant phenotype | Source or reference |
| --- | --- | --- |
| *E.coli* Strains |  |  |
| *E.coli* DH5α | supE44 Δlac*U169*(φ80lacZΔM15)*hsdR17recA1 endA1 gyrA96thi-1 relA1*λpir | stratagene |
| *E.coli* BL21(DE3) | *F^-^ ompT hsdS_B_ (r_B_^-^m_B_^-^) gal dcm met* (DE3) | Invitrogen |
| *P. aeruginosa* Strains |  |  |
| *P. savastanoi pv. phaseolicola* 1448A | Wild type | (1) |
| *P. savastanoi pv. phaseolicola* 1448A *ΔrhpS* | clean deletion of *rhpS* | This study |
| *P. savastanoi pv. phaseolicola* 1448A *ΔrhpRS* | clean deletion of *rhpRS* | This study |
| *P. savastanoi pv. phaseolicola* 1448A *ΔhrpS* | clean deletion of *hrpS* | (2) |
| Plasmids | **Description** |  |
| pET28a | His-tag protein expression in *E.coli* | Invitrogen |
| pET28a-*rhpR* | pET28a containing *rhpR* from *P. savastanoi pv. phaseolicola* 1448A | This study |
| pET28a-*rhpR*-D70A | pET28a containing *rhpR-*D70A | This study |
| pET28a*-rhpR*-D70E | pET28a containing *rhpR*-D70E | This study |
| pHM1 | Broad-host-range *cos* IncW derivative of Pri40 | (3) |
| pHM1-*rhpR* | Overexpression *rhpR* from *P. savastanoi pv. phaseolicola* 1448A | This study |
| pHM1-*rhpR*-D70A | Overexpression *rhpR*-D70A | This study |
| pHM1-*rhpR*-D70E | Overexpression *rhpR*-D70E | This study |
| pHM2 | Complementary vector with no promoter before MCS | (4) |
| pHM2-*rhpR*-HA | Translational fusion between *rhpR* ORF and HA tag in pHM2 | This study |
| pMS402 | Reporter plasmid carring the promoterless *lux* CDABE | (5) |
| pMS402-*hrpR* | Transcriptional fusion between *hrpR* promoter and *lux* in pMS402 | This study |
| pMS402-*hrpR-*ΔIR | Transcriptional fusion between *hrpR* promoter lacking IR element and *lux* in pMS402 | This study |
| pMS402-*hopR1* | Transcriptional fusion between *hopR1* promoter and *lux* in pMS402 | This study |
| pMS402-*hopR1-*ΔIR | Transcriptional fusion between *hopR1* promoter lacking IR element and *lux* in pMS402 | This study |
| pMS402-*flhA* | Transcriptional fusion between *flhA* promoter and *lux* in pMS402 | This study |
| pMS402-*flhA-*ΔIR | Transcriptional fusion between *flhA* promoter lacking IR element and *lux* in pMS402 | This study |
| pMS402-*fimA* | Transcriptional fusion between *fimA* promoter and *lux* in pMS402 | This study |
| pMS402-*fimA-*ΔIR | Transcriptional fusion between *fimA* promoter lacking IR element and *lux* in pMS402 | This study |
| pMS402-*algD* | Transcriptional fusion between *algD* promoter and *lux* in pMS402 | This study |
| pMS402-*algD-*ΔIR | Transcriptional fusion between *algD* promoter lacking IR element and *lux* in pMS402 | This study |
| pMS402-PSPPH_2590 | Transcriptional fusion between PSPPH_2590 promoter and *lux* in pMS402 | This study |
| pMS402-PSPPH_2590*-*ΔIR | Transcriptional fusion between PSPPH_2590 promoter lacking IR element and *lux* in pMS402 | This study |
| pMS402-PSPPH_2653 | Transcriptional fusion between PSPPH_2590 promoter and *lux* in pMS402 | This study |
| pMS402-PSPPH_2653*-*ΔIR | Transcriptional fusion between PSPPH_2590 promoter lacking IR element and *lux* in pMS402 | This study |
| pMS402-*ccmA* | Transcriptional fusion between *ccmA* promoter and *lux* in pMS402 | This study |
| pMS402-*ccmA-*ΔIR | Transcriptional fusion between *ccmA* promoter lacking IR element and *lux* in pMS402 | This study |
| pMS402-*adhB* | Transcriptional fusion between *adhB* promoter and *lux* in pMS402 | This study |
| pMS402-*adhB-*ΔIR | Transcriptional fusion between *adhB* promoter lacking IR element and *lux* in pMS402 | This study |
| pMS402-*trpG* | Transcriptional fusion between *trpG* promoter and *lux* in pMS402 | This study |
| pMS402-*hemB* | Transcriptional fusion between *hemB* promoter and *lux* in pMS402 | This study |
| pMS402-*rpoD* | Transcriptional fusion between *rpoD* promoter and *lux* in pMS402 | This study |
| Primers | **Sequence** |  |
| *hrpR*-pro-XhoI-F | TTCTCGAGTAACCTGCGGTTTTAAAG |  |
| *hrpR*-pro-Rm | GAACAAAAACAAGGACATAGCGTCTTAACGG |  |
| *hrpR*-pro-Fm | CCGTTAAGACGCTATGTCCTTGTTTTTGTTC |  |
| *hrpR*-pro-BamHI-R | TTGGATCCCGATCACTCTCACTGTG |  |
| *rhpR*(-pET28a)-BamHI F | TTGGATCCATGCAAGCACTTCCCGACAC |  |
| *rhpR*(-pET28a)-XhoI R | TTCTCGAGTCAACCCAGCTCCCTGGCA |  |
| *rhpR*[(D70E)-pET28a]-Fm | TGGTGCTCGAACTGATGCTG |  |
| *rhpR*[(D70E)-pET28a]-Rm | CAGCATCAGTTCGAGCACCA |  |
| *rhpR*-HM1-HindIII F | TT AAGCTT ATGCAAGCACTTCCCGACAC |  |
| *rhpR*-HM1-PstI R | TT CTGCAG GATGCCAGGGAGCTGGGTTGA |  |
| *rhpR*D70A-pHM1-Fm | TGGTGCTCGCCCTGATGCTG |  |
| *rhpR*D70A-pHM1-Rm | CAGCATCAGGGCGAGCACCA |  |
| *flhA*-pro-XhoI-F | ATATATCTCGAGCCCTTTGCTTTGTCGCCAGC |  |
| *flhA*-pro-BamHI-R | GACGGATCCTGATTGTTTACCTGCGCACTAGATC |  |
| *ccmA*-pro XhoI F | TT CTCGAG TGAAATATCGACCTGAGG |  |
| *ccmA*-pro BamHI R | TT GGATCC CGGGCGTTTCCAGTGCTG |  |
| *rpoD*-pro XhoI F | TT CTCGAG GGTCATAGCTCGGGTATAAT |  |
| *rpoD*-pro BamHI R | TT GGATCC AACACCCTATCCACTGAAGG |  |
| *trpG*-pro XhoI F | TT CTCGAG TCAATTCTAGCGCACAG |  |
| *trpG*-pro BamHI R | TT GGATCC AACCTTTTGAATTTGCTCAC |  |
| *fimA* pro XhoI F | TT CTCGAG AACAAGGGCCTGCCTG |  |
| *fimA* pro BamHI R | TT GGATCC CCTTCTAAGTATTAAAGTCACAAG |  |
| *algD* pro XhoI F | TT CTCGAG ACACTGTAACCGTCG |  |
| *algD* pro BamHI R | TT GGATCC CGTATTCTCCTCGATTTTTC |  |
| PSPPH_2653 pro XhoI F | TT CTCGAG GTCAGGAACCTCCG |  |
| PSPPH_2653 pro BamHI R | TT GGATCC GACTACCCTGTAAGC |  |
| PSPPH_2590-pro XhoI F | TT CTCGAG GACTGTCACCTGTTCAG |  |
| PSPPH_2590-pro BamHI R | TT GGATCC GGAAACACCAGATGC |  |
| *adhB* pro XhoI F | TT CTCGAG AACAGCCACCCTGCAC |  |
| *adhB* pro BamHI R | TT GGATCC GACGGACCCTCGTGGG |  |
| *hopR1* Rm | AACGGCATCATGCCGATCTCCATTACAAGCTCG |  |
| *hopR1* Fm | CGAGCTTGTAATGGAGATCGGCATGATGCCGTT |  |
| *flhA* Fm | AAAGTTGGACAGCTTTTTAGGTCGCCATCGGCG |  |
| *flhA* Rm | CGCCGATGGCGACCTAAAAAGCTGTCCAACTTT |  |
| PSPPH_2590 Fm | GAACTTTGCAACAATGCACTTTGGTCATGGTTTTTAC |  |
| PSPPH_2590 Rm | GTAAAAACCATGACCAAAGTGCATTGTTGCAAAGTTC |  |
| *fimA* Fm | GCCCGAACACAAAAAAACATTTGCCCTTCGGATTC |  |
| *fimA* Rm | GAATCCGAAGGGCAAATGTTTTTTTGTGTTCGGGC |  |
| *algD* Fm | CGCGATACTGCCCCTAGCAAAATCCGCAAAT |  |
| *algD* Rm | ATTTGCGGATTTTGCTAGGGGCAGTATCGCG |  |
| *ccmA* Fm | GCCTTTTACTGCGTATATTTCGCGACGCTCCAG |  |
| *ccmA* Rm | CTGGAGCGTCGCGAAATATACGCAGTAAAAGGC |  |
| *adhB* BamHI R（IR） | TT GGATCC GACGGACCCTCGTGGGGTGATAGCCCAAG |  |
| *trpG* Fm | AATGATATTGCACCGTATGTCCTGCAACCTCC |  |
| *trpG* Rm | GGAGGTTGCAGGACATACGGTGCAATATCATT |  |
| *adhB* RT F | CTGCCTCATGTTCAGCGTTT |  |
| *adhB* RT R | ATCCTCTTCCTTGACGCCAA |  |
| *hopR1* RT R | ATCTGAGTGCTATCGGGGTG |  |
| *hopG1* RT F | CGGCTATGCATCACGATCTG |  |
| *hopG1* RT R | AGGACTTAGGCGCTCACTTT |  |
| *flhA* RT F | CGCACTTCTCCTTCATCAGC |  |
| *flhA* RT R | GTCGATAGGGGTGACGTCAT |  |
| PSPPH_2653 RT F | GGCAAATGTAGAGGCACCAG |  |
| PSPPH_2653 RT R | GGAAAGTCCCGGTCAGTACA |  |
| PSPPH_2590-RT F | CTTTGTCTCTCGCCACATCG |  |
| PSPPH_2590-RT R | GTGGGAGTTTTCATGCTGCA |  |
| *rpoD* RT F | GCCACTGTTGAAAGCCTGAA |  |
| *rpoD* RT R | GCAGTGCCTTGGCTTCTATC |  |
| *hemB* RT F | GGTGGGTTCTTCGCTGAATC |  |
| *hemB* RT R | ATGTGCATCGCGTATTCACC |  |
| *trpG* RT F | TACAACGTCGTGCAGTACCT |  |
| *trpG* RT R | TTGCCCGCGAAATGTTTGAT |  |
| *ccmA* RT F | GCCGATGCCATTTTCAATGC |  |
| *ccmA* RT R | ATGATCATGCCGCCCTTTTC |  |
| *hrpL*-RT-F | GCAACGAGCACAAGTTTCAA |  |
| *hrpL*-RT-R | ATATCGCCATTCCATTCCAG |  |
| *hrpR*-RT-F | GATCCTGCCATTGTTCGACC |  |
| *hrpR*-RT-R | GGAACCCCAATACAAACCGC |  |
| *rhpR*-RT-F | CCGACGATTACATGGCCAAG |  |
| *rhpR*-RT-R | AACTGTTCACGGCTAAGCAC |  |
| *fimA*-RT-F | ATCCGTACTAGGCCTTGCTC |  |
| *fimA*-RT-R | CCCATGTTAACCAGGTTGCC |  |
| *algD*-RT-F | CCTATCGTTGAACCGGGTCT |  |
| *algD*-RT-R | GTTCCAGATCGCCGTTCTTC |  |
| *rhpS-*Up-F | TTAAGCTTCTCCTTTGCTACCGATTGCC |  |
| *rhpS-*Up-R | TTGGATCCCATGAGGTTGAAGGTCTGCG |  |
| *rhpS*-Down-F | TTGGATCCCACATTCTGTATCACACCGC |  |
| *rhpS*-Down-R | TTGAATTCTATAGCCTGCTGACCGACTG |  |
| *rhpRS-*Up-F | TTGAATTC AATCTGCCGCTGGTGATT |  |
| *rhpRS-*Up-R | TT CTCGAG C ACATTCTGTATCACACC |  |
| *rhpRS*-Down-F | TTCTCGAG AGTGCGTCTGTCGCCTGC |  |
| *rhpRS*-Down-R | TT AAGCTT GGGCTGTGCATGAGCGAG |  |
| *rhpS*-ORF-F | CACCCTCATTGTTGCTGCAT |  |
| *rhpS*-ORF-R | TAAGCTTTGTACGCGGTGTG |  |
| *rhpRS*-ORF-F | GAGATAAATGTGTCCGGTAGCG |  |
| *rhpRS*-ORF-R | GGAAAGCACGTTCGACAGTTTT |  |
| *rhpS-*RT-F | GACTCAGGACCTCAACGACA |  |
| *rhpS-*RT-R | CTGCACATCATCGCCATTGT |  |
| *rhpS*-comp-F | TGACCATGATTACGCCAAGCTTGTTCGATGCCAGGGAGCT |  |
| *rhpS*-comp-R | CTCTAGAGTCGACAACTGCAGTCAAAGGCGCGGCAGATT |  |

**Reference**

1. Deng X, Liang H, Chen K, He C, Lan L, Tang X. 2014. Molecular mechanisms of two-component system RhpRS regulating type III secretion system in *Pseudomonas syringae*. Nucleic Acids Res 42:11472-86.

2. Wang J, Shao X, Zhang Y, Zhu Y, Yang P, Yuan J, Wang T, Yin C, Wang W, Chen S, Liang H, Deng X. 2018. HrpS Is a Global Regulator on Type III Secretion System (T3SS) and Non-T3SS Genes in *Pseudomonas savastanoi* pv*. phaseolicola*. Mol Plant Microbe Interact doi:10.1094/MPMI-02-18-0035-R:MPMI02180035R.

3. Innes RW, Hirose MA, Kuempel PL. 1988. Induction of nitrogen-fixing nodules on clover requires only 32 kilobase pairs of DNA from the *Rhizobium trifolii* symbiosis plasmid. J Bacteriol 170:3793-802.

4. Liu HM, Chak KF, Piggot PJ. 1982. Isolation and characterization of a recombinant plasmid carrying a functional part of the *Bacillus subtilis* spoIIA locus. J Gen Microbiol 128:2805-12.

5. Duan K, Dammel C, Stein J, Rabin H, Surette MG. 2003. Modulation of *Pseudomonas aeruginosa* gene expression by host microflora through interspecies communication. Mol Microbiol 50:1477-91.
