# Supplementary material for: Pseudomonas savastanoi Two-Component System RhpRS Switches between Virulence and Metabolism by Tuning Phosphorylation State and Sensing Nutritional Conditions
Source: mBio. 2019 Mar 19;10(2):e02838-18. doi: 10.1128/mBio.02838-18 (PMC6426608; doi:10.1128/mBio.02838-18)
Supplement: TABLE S3 [file mBio.02838-18-st003.docx]

**Table S3 ChIP-seq data of RhpR binding sites.**

**A. ChIP-seq result for RhpR binding sites in wild-type** ***Pseudomonas savastanoi* pv. *phaseolicola* 1448A in KB**

| seqnames | start | end | width | strand | peak | feature | start_position | end_position | feature_strand | insideFeature | distancetoFeature | shortestDistance | fromOverlappingOrNearest | id | fold_enrichment |
| --- | --- | --- | --- | --- | --- | --- | --- | --- | --- | --- | --- | --- | --- | --- | --- |
| chromosome | 12552 | 12680 | 129 | * | rhpR_KB_R1_peak_1 | 9 | 11095 | 12453 | - | upstream | -99 | 99 | NearestLocation | PSPPH_0008 | 2.98977 |
| chromosome | 167840 | 167958 | 119 | * | rhpR_KB_R1_peak_2 | 145 | 166900 | 167736 | - | upstream | -104 | 104 | NearestLocation | PSPPH_0140 | 2.36031 |
| chromosome | 236856 | 236972 | 117 | * | rhpR_KB_R1_peak_3 | 208 | 236596 | 236937 | - | overlapStart | 81 | 35 | NearestLocation | PSPPH_0198 | 1.80533 |
| chromosome | 320874 | 321048 | 175 | * | rhpR_KB_R1_peak_4 | 289 | 321012 | 322022 | + | overlapStart | -138 | 36 | NearestLocation | PSPPH_0279 | 3.57356 |
| chromosome | 744391 | 744493 | 103 | * | rhpR_KB_R1_peak_5 | 642 | 743123 | 744394 | - | overlapStart | 3 | 3 | NearestLocation | PSPPH_0628 | 2.13128 |
| chromosome | 882168 | 882259 | 92 | * | rhpR_KB_R1_peak_6 | 772 | 882507 | 883145 | + | upstream | -339 | 248 | NearestLocation | PSPPH_0755 | 2.56392 |
| chromosome | 889054 | 889200 | 147 | * | rhpR_KB_R1_peak_7 | 776 | 889391 | 889831 | - | downstream | 777 | 191 | NearestLocation | PSPPH_0759 | 3.21821 |
| chromosome | 1345960 | 1346106 | 147 | * | rhpR_KB_R1_peak_8 | 1176 | 1345074 | 1345496 | - | upstream | -464 | 464 | NearestLocation | PSPPH_1146 | 2.89656 |
| chromosome | 1359366 | 1359467 | 102 | * | rhpR_KB_R1_peak_9 | 1188 | 1357817 | 1359421 | - | overlapStart | 55 | 46 | NearestLocation | PSPPH_1157 | 2.71689 |
| chromosome | 1362263 | 1362386 | 124 | * | rhpR_KB_R1_peak_10 | 1191 | 1361947 | 1362159 | - | upstream | -104 | 104 | NearestLocation | PSPPH_1160 | 2.1062 |
| chromosome | 1418087 | 1418166 | 80 | * | rhpR_KB_R1_peak_11 | 1239 | 1417026 | 1417922 | - | upstream | -165 | 165 | NearestLocation | PSPPH_1207 | 2.2915 |
| chromosome | 1651869 | 1652203 | 335 | * | rhpR_KB_R1_peak_12 | 1465 | 1651696 | 1651851 | + | downstream | 173 | 18 | NearestLocation | PSPPH_1423 | 4.47155 |
| chromosome | 1708842 | 1708949 | 108 | * | rhpR_KB_R1_peak_13 | 1508 | 1708032 | 1708760 | - | upstream | -82 | 82 | NearestLocation | PSPPH_1465 | 2.23383 |
| chromosome | 1725007 | 1725123 | 117 | * | rhpR_KB_R1_peak_14 | 1528 | 1726042 | 1727358 | + | upstream | -1035 | 919 | NearestLocation | PSPPH_1485 | 1.74662 |
| chromosome | 1904155 | 1904315 | 161 | * | rhpR_KB_R1_peak_15 | 1693 | 1903900 | 1904643 | + | inside | 255 | 255 | NearestLocation | PSPPH_1641 | 2.26376 |
| chromosome | 2068256 | 2068398 | 143 | * | rhpR_KB_R1_peak_16 | 1826 | 2068454 | 2069206 | + | upstream | -198 | 56 | NearestLocation | PSPPH_1771 | 2.79919 |
| chromosome | 2352582 | 2353789 | 1208 | * | rhpR_KB_R1_peak_17 | 2066 | 2352536 | 2353285 | - | overlapStart | 703 | 46 | NearestLocation | PSPPH_2004 | 5.48173 |
| chromosome | 2497735 | 2497817 | 83 | * | rhpR_KB_R1_peak_18 | 2206 | 2497808 | 2498110 | + | overlapStart | -73 | 9 | NearestLocation | PSPPH_2141 | 2.44559 |
| chromosome | 2579596 | 2579810 | 215 | * | rhpR_KB_R1_peak_19 | 2293 | 2579623 | 2580372 | - | overlapEnd | 776 | 27 | NearestLocation | PSPPH_2221 | 2.41481 |
| chromosome | 2936627 | 2937000 | 374 | * | rhpR_KB_R1_peak_20 | 2630 | 2934900 | 2936543 | - | upstream | -84 | 84 | NearestLocation | PSPPH_2545 | 3.34067 |
| chromosome | 3686427 | 3686568 | 142 | * | rhpR_KB_R1_peak_21 | 3289 | 3686569 | 3686865 | + | upstream | -142 | 1 | NearestLocation | PSPPH_3179 | 3.4671 |
| chromosome | 3836615 | 3836736 | 122 | * | rhpR_KB_R1_peak_22 | 3427 | 3835994 | 3836635 | - | overlapStart | 20 | 20 | NearestLocation | PSPPH_3314 | 3.6266 |
| chromosome | 3878976 | 3879165 | 190 | * | rhpR_KB_R1_peak_23 | 3464 | 3879030 | 3879500 | - | overlapEnd | 524 | 54 | NearestLocation | PSPPH_3350 | 2.56073 |
| chromosome | 3915619 | 3915713 | 95 | * | rhpR_KB_R1_peak_24 | 3500 | 3913997 | 3915211 | - | upstream | -408 | 408 | NearestLocation | PSPPH_3386 | 2.6949 |
| chromosome | 4222387 | 4222468 | 82 | * | rhpR_KB_R1_peak_25 | 3796 | 4221051 | 4221836 | - | upstream | -551 | 551 | NearestLocation | PSPPH_3674 | 2.47576 |
| chromosome | 4233263 | 4233343 | 81 | * | rhpR_KB_R1_peak_26 | 3807 | 4232944 | 4233237 | + | downstream | 319 | 26 | NearestLocation | PSPPH_3685 | 2.4158 |
| chromosome | 4233452 | 4233705 | 254 | * | rhpR_KB_R1_peak_27 | 3807 | 4232944 | 4233237 | + | downstream | 508 | 215 | NearestLocation | PSPPH_3685 | 5.47758 |
| chromosome | 4479691 | 4479833 | 143 | * | rhpR_KB_R1_peak_28 | 4046 | 4479661 | 4482798 | + | inside | 30 | 30 | NearestLocation | PSPPH_3922 | 2.55535 |
| chromosome | 4559642 | 4559827 | 186 | * | rhpR_KB_R1_peak_29 | 4121 | 4558558 | 4559706 | - | overlapStart | 64 | 64 | NearestLocation | PSPPH_3994 | 3.91159 |
| chromosome | 4580371 | 4580466 | 96 | * | rhpR_KB_R1_peak_30 | 4139 | 4580595 | 4582079 | + | upstream | -224 | 129 | NearestLocation | PSPPH_4010 | 2.60275 |
| chromosome | 4596375 | 4596483 | 109 | * | rhpR_KB_R1_peak_31 | 4150 | 4596548 | 4596856 | + | upstream | -173 | 65 | NearestLocation | PSPPH_4021 | 2.22557 |
| chromosome | 4736463 | 4736554 | 92 | * | rhpR_KB_R1_peak_32 | 4289 | 4736613 | 4736921 | + | upstream | -150 | 59 | NearestLocation | PSPPH_4152 | 2.91679 |
| chromosome | 4860243 | 4860366 | 124 | * | rhpR_KB_R1_peak_33 | 4397 | 4860267 | 4861985 | + | overlapStart | -24 | 24 | NearestLocation | PSPPH_4259 | 2.05147 |
| chromosome | 4909030 | 4909143 | 114 | * | rhpR_KB_R1_peak_34 | 4436 | 4907552 | 4908361 | - | upstream | -669 | 669 | NearestLocation | PSPPH_4298 | 2.36701 |
| chromosome | 5148602 | 5148706 | 105 | * | rhpR_KB_R1_peak_35 | 4658 | 5148798 | 5149484 | + | upstream | -196 | 92 | NearestLocation | PSPPH_4518 | 2.4795 |
| chromosome | 5148824 | 5149070 | 247 | * | rhpR_KB_R1_peak_36 | 4658 | 5148798 | 5149484 | + | inside | 26 | 26 | NearestLocation | PSPPH_4518 | 2.92866 |
| chromosome | 5172389 | 5172489 | 101 | * | rhpR_KB_R1_peak_37 | 4678 | 5172524 | 5173330 | + | upstream | -135 | 35 | NearestLocation | PSPPH_4538 | 2.36701 |
| chromosome | 5492503 | 5492581 | 79 | * | rhpR_KB_R1_peak_38 | 4983 | 5493584 | 5494279 | + | upstream | -1081 | 1003 | NearestLocation | PSPPH_4834 | 2.38092 |
| chromosome | 5903129 | 5903235 | 107 | * | rhpR_KB_R1_peak_39 | 5368 | 5903367 | 5904626 | + | upstream | -238 | 132 | NearestLocation | PSPPH_5202 | 2.44559 |

**B.** **ChIP-seq result for RhpR binding sites in wild-type** ***Pseudomonas savastanoi* pv. *phaseolicola* 1448A in MM**

| seqnames | start | end | width | strand | peak | feature | start_position | end_position | feature_strand | insideFeature | distancetoFeature | shortestDistance | fromOverlappingOrNearest | id |
| --- | --- | --- | --- | --- | --- | --- | --- | --- | --- | --- | --- | --- | --- | --- |
| chromosome | 744364 | 744550 | 187 | * | rhpR_MM_R1_peak_1 | 642 | 743123 | 744394 | - | overlapStart | 30 | 30 | NearestLocation | PSPPH_0628 |
| chromosome | 2127282 | 2127363 | 82 | * | rhpR_MM_R1_peak_2 | 1883 | 2127447 | 2127788 | + | upstream | -165 | 84 | NearestLocation | PSPPH_1827 |
| chromosome | 2352574 | 2353792 | 1219 | * | rhpR_MM_R1_peak_3 | 2066 | 2352536 | 2353285 | - | overlapStart | 711 | 38 | NearestLocation | PSPPH_2004 |
| chromosome | 4066954 | 4067029 | 76 | * | rhpR_MM_R1_peak_4 | 3647 | 4065640 | 4066416 | - | upstream | -538 | 538 | NearestLocation | PSPPH_3527 |
| chromosome | 4233527 | 4233630 | 104 | * | rhpR_MM_R1_peak_5 | 3807 | 4232944 | 4233237 | + | downstream | 583 | 290 | NearestLocation | PSPPH_3685 |
| chromosome | 4479722 | 4479836 | 115 | * | rhpR_MM_R1_peak_6 | 4046 | 4479661 | 4482798 | + | inside | 61 | 61 | NearestLocation | PSPPH_3922 |
| chromosome | 4580571 | 4580715 | 145 | * | rhpR_MM_R1_peak_7 | 4139 | 4580595 | 4582079 | + | overlapStart | -24 | 24 | NearestLocation | PSPPH_4010 |

**C. ChIP-seq result for RhpR binding sites in *Pseudomonas savastanoi* pv. *phaseolicola* 1448A *rhpS* mutant** **in KB**

| seqnames | start | end | width | strand | peak | feature | start_position | end_position | feature_strand | insideFeature | distancetoFeature | shortestDistance | fromOverlappingOrNearest | id |
| --- | --- | --- | --- | --- | --- | --- | --- | --- | --- | --- | --- | --- | --- | --- |
| chromosome | 148 | 227 | 80 | * | rhpR_rhpS-Mutant_KB_R1_peak_1 | 1 | 238 | 1773 | + | upstream | -90 | 11 | NearestLocation | PSPPH_0001 |
| chromosome | 4043 | 4194 | 152 | * | rhpR_rhpS-Mutant_KB_R1_peak_2 | 4 | 4046 | 6463 | + | overlapStart | -3 | 3 | NearestLocation | PSPPH_0004 |
| chromosome | 12588 | 12710 | 123 | * | rhpR_rhpS-Mutant_KB_R1_peak_3 | 9 | 11095 | 12453 | - | upstream | -135 | 135 | NearestLocation | PSPPH_0008 |
| chromosome | 111861 | 112043 | 183 | * | rhpR_rhpS-Mutant_KB_R1_peak_4 | 104 | 112706 | 113614 | + | upstream | -845 | 663 | NearestLocation | PSPPH_0101 |
| chromosome | 113897 | 114014 | 118 | * | rhpR_rhpS-Mutant_KB_R1_peak_5 | 105 | 113614 | 114579 | + | inside | 283 | 283 | NearestLocation | PSPPH_0102 |
| chromosome | 135146 | 135317 | 172 | * | rhpR_rhpS-Mutant_KB_R1_peak_6 | 120 | 134132 | 136063 | - | inside | 917 | 746 | NearestLocation | PSPPH_0117 |
| chromosome | 164372 | 164456 | 85 | * | rhpR_rhpS-Mutant_KB_R1_peak_7 | 143 | 163896 | 164447 | - | overlapStart | 75 | 9 | NearestLocation | PSPPH_0138 |
| chromosome | 182777 | 182859 | 83 | * | rhpR_rhpS-Mutant_KB_R1_peak_8 | 161 | 182883 | 184157 | + | upstream | -106 | 24 | NearestLocation | PSPPH_0155 |
| chromosome | 189781 | 189863 | 83 | * | rhpR_rhpS-Mutant_KB_R1_peak_9 | 171 | 189713 | 190558 | + | inside | 68 | 68 | NearestLocation | PSPPH_0165 |
| chromosome | 204253 | 204433 | 181 | * | rhpR_rhpS-Mutant_KB_R1_peak_10 | 178 | 198685 | 204564 | - | inside | 311 | 131 | NearestLocation | PSPPH_0171 |
| chromosome | 206405 | 206497 | 93 | * | rhpR_rhpS-Mutant_KB_R1_peak_11 | 180 | 206633 | 206890 | - | downstream | 485 | 136 | NearestLocation | PSPPH_0172 |
| chromosome | 206405 | 206497 | 93 | * | rhpR_rhpS-Mutant_KB_R1_peak_11 | 181 | 206633 | 206890 | - | downstream | 485 | 136 | NearestLocation | PSPPH_0172 |
| chromosome | 211126 | 211231 | 106 | * | rhpR_rhpS-Mutant_KB_R1_peak_12 | 186 | 210746 | 211510 | + | inside | 380 | 279 | NearestLocation | PSPPH_0177 |
| chromosome | 212804 | 212928 | 125 | * | rhpR_rhpS-Mutant_KB_R1_peak_13 | 188 | 212966 | 214093 | + | upstream | -162 | 38 | NearestLocation | PSPPH_0179 |
| chromosome | 317706 | 317809 | 104 | * | rhpR_rhpS-Mutant_KB_R1_peak_14 | 285 | 317114 | 317779 | - | overlapStart | 73 | 30 | NearestLocation | PSPPH_0275 |
| chromosome | 317987 | 318117 | 131 | * | rhpR_rhpS-Mutant_KB_R1_peak_15 | 285 | 317114 | 317779 | - | upstream | -208 | 208 | NearestLocation | PSPPH_0275 |
| chromosome | 320810 | 321009 | 200 | * | rhpR_rhpS-Mutant_KB_R1_peak_16 | 289 | 321012 | 322022 | + | upstream | -202 | 3 | NearestLocation | PSPPH_0279 |
| chromosome | 364575 | 364904 | 330 | * | rhpR_rhpS-Mutant_KB_R1_peak_17 | 332 | 364889 | 365785 | + | overlapStart | -314 | 15 | NearestLocation | PSPPH_0320 |
| chromosome | 539619 | 539721 | 103 | * | rhpR_rhpS-Mutant_KB_R1_peak_18 | 488 | 539049 | 539621 | - | overlapStart | 2 | 2 | NearestLocation | PSPPH_0476 |
| chromosome | 554950 | 555028 | 79 | * | rhpR_rhpS-Mutant_KB_R1_peak_19 | 499 | 555043 | 556275 | + | upstream | -93 | 15 | NearestLocation | PSPPH_0487 |
| chromosome | 630657 | 630775 | 119 | * | rhpR_rhpS-Mutant_KB_R1_peak_20 | 549 | 630951 | 632069 | + | upstream | -294 | 176 | NearestLocation | PSPPH_0536 |
| chromosome | 642359 | 642471 | 113 | * | rhpR_rhpS-Mutant_KB_R1_peak_21 | 561 | 642501 | 643199 | + | upstream | -142 | 30 | NearestLocation | PSPPH_0548 |
| chromosome | 718545 | 718664 | 120 | * | rhpR_rhpS-Mutant_KB_R1_peak_22 | 622 | 718866 | 719993 | + | upstream | -321 | 202 | NearestLocation | PSPPH_0609 |
| chromosome | 721894 | 721985 | 92 | * | rhpR_rhpS-Mutant_KB_R1_peak_23 | 624 | 722115 | 723011 | + | upstream | -221 | 130 | NearestLocation | PSPPH_0611 |
| chromosome | 732480 | 732759 | 280 | * | rhpR_rhpS-Mutant_KB_R1_peak_24 | 631 | 731881 | 732564 | - | overlapStart | 84 | 84 | NearestLocation | PSPPH_0618 |
| chromosome | 732480 | 732759 | 280 | * | rhpR_rhpS-Mutant_KB_R1_peak_24 | 632 | 731881 | 732564 | - | overlapStart | 84 | 84 | NearestLocation | PSPPH_0618 |
| chromosome | 734508 | 734835 | 328 | * | rhpR_rhpS-Mutant_KB_R1_peak_25 | 633 | 732770 | 734620 | - | overlapStart | 112 | 112 | NearestLocation | PSPPH_0619 |
| chromosome | 736817 | 736988 | 172 | * | rhpR_rhpS-Mutant_KB_R1_peak_26 | 634 | 734688 | 736643 | - | upstream | -174 | 174 | NearestLocation | PSPPH_0620 |
| chromosome | 744385 | 744465 | 81 | * | rhpR_rhpS-Mutant_KB_R1_peak_27 | 642 | 743123 | 744394 | - | overlapStart | 9 | 9 | NearestLocation | PSPPH_0628 |
| chromosome | 746546 | 746623 | 78 | * | rhpR_rhpS-Mutant_KB_R1_peak_28 | 643 | 744516 | 746438 | - | upstream | -108 | 108 | NearestLocation | PSPPH_0629 |
| chromosome | 770857 | 770992 | 136 | * | rhpR_rhpS-Mutant_KB_R1_peak_29 | 664 | 768944 | 770866 | - | overlapStart | 9 | 9 | NearestLocation | PSPPH_0650 |
| chromosome | 771258 | 771360 | 103 | * | rhpR_rhpS-Mutant_KB_R1_peak_30 | 665 | 770972 | 771238 | - | upstream | -20 | 20 | NearestLocation | PSPPH_0651 |
| chromosome | 782730 | 782833 | 104 | * | rhpR_rhpS-Mutant_KB_R1_peak_31 | 682 | 783191 | 784366 | + | upstream | -461 | 358 | NearestLocation | PSPPH_0667 |
| chromosome | 788446 | 788661 | 216 | * | rhpR_rhpS-Mutant_KB_R1_peak_32 | 687 | 788757 | 789356 | + | upstream | -311 | 96 | NearestLocation | PSPPH_0672 |
| chromosome | 854677 | 854756 | 80 | * | rhpR_rhpS-Mutant_KB_R1_peak_33 | 747 | 854922 | 855326 | + | upstream | -245 | 166 | NearestLocation | PSPPH_0731 |
| chromosome | 864149 | 864236 | 88 | * | rhpR_rhpS-Mutant_KB_R1_peak_34 | 753 | 863034 | 864626 | - | inside | 477 | 390 | NearestLocation | PSPPH_0737 |
| chromosome | 867986 | 868120 | 135 | * | rhpR_rhpS-Mutant_KB_R1_peak_35 | 758 | 868016 | 870580 | + | overlapStart | -30 | 30 | NearestLocation | PSPPH_0742 |
| chromosome | 875181 | 875299 | 119 | * | rhpR_rhpS-Mutant_KB_R1_peak_36 | 765 | 873205 | 874737 | - | upstream | -444 | 444 | NearestLocation | PSPPH_0749 |
| chromosome | 882201 | 882500 | 300 | * | rhpR_rhpS-Mutant_KB_R1_peak_37 | 772 | 882507 | 883145 | + | upstream | -306 | 7 | NearestLocation | PSPPH_0755 |
| chromosome | 883598 | 883747 | 150 | * | rhpR_rhpS-Mutant_KB_R1_peak_38 | 773 | 884445 | 887003 | + | upstream | -847 | 698 | NearestLocation | PSPPH_0756 |
| chromosome | 889027 | 889104 | 78 | * | rhpR_rhpS-Mutant_KB_R1_peak_39 | 776 | 889391 | 889831 | - | downstream | 804 | 287 | NearestLocation | PSPPH_0759 |
| chromosome | 896782 | 896896 | 115 | * | rhpR_rhpS-Mutant_KB_R1_peak_40 | 784 | 896731 | 896976 | - | inside | 194 | 51 | NearestLocation | PSPPH_0766 |
| chromosome | 896782 | 896896 | 115 | * | rhpR_rhpS-Mutant_KB_R1_peak_40 | 785 | 896731 | 896976 | - | inside | 194 | 51 | NearestLocation | PSPPH_0766 |
| chromosome | 897390 | 897494 | 105 | * | rhpR_rhpS-Mutant_KB_R1_peak_41 | 784 | 896731 | 896976 | - | upstream | -414 | 414 | NearestLocation | PSPPH_0766 |
| chromosome | 897390 | 897494 | 105 | * | rhpR_rhpS-Mutant_KB_R1_peak_41 | 785 | 896731 | 896976 | - | upstream | -414 | 414 | NearestLocation | PSPPH_0766 |
| chromosome | 898957 | 899058 | 102 | * | rhpR_rhpS-Mutant_KB_R1_peak_42 | 786 | 897416 | 898963 | - | overlapStart | 6 | 6 | NearestLocation | PSPPH_0767 |
| chromosome | 902857 | 902957 | 101 | * | rhpR_rhpS-Mutant_KB_R1_peak_43 | 789 | 901688 | 903976 | + | inside | 1169 | 1019 | NearestLocation | PSPPH_0770 |
| chromosome | 905396 | 905561 | 166 | * | rhpR_rhpS-Mutant_KB_R1_peak_44 | 790 | 904056 | 904868 | - | upstream | -528 | 528 | NearestLocation | PSPPH_0771 |
| chromosome | 910538 | 910733 | 196 | * | rhpR_rhpS-Mutant_KB_R1_peak_45 | 795 | 910262 | 910975 | - | inside | 437 | 242 | NearestLocation | PSPPH_0775 |
| chromosome | 914183 | 914262 | 80 | * | rhpR_rhpS-Mutant_KB_R1_peak_46 | 798 | 914293 | 914931 | + | upstream | -110 | 31 | NearestLocation | PSPPH_0778 |
| chromosome | 935615 | 935753 | 139 | * | rhpR_rhpS-Mutant_KB_R1_peak_47 | 814 | 935833 | 936333 | + | upstream | -218 | 80 | NearestLocation | PSPPH_0791 |
| chromosome | 957950 | 958271 | 322 | * | rhpR_rhpS-Mutant_KB_R1_peak_48 | 836 | 958236 | 960398 | + | overlapStart | -286 | 35 | NearestLocation | PSPPH_0813 |
| chromosome | 976350 | 976443 | 94 | * | rhpR_rhpS-Mutant_KB_R1_peak_49 | 839 | 977780 | 977977 | - | downstream | 1627 | 1337 | NearestLocation | PSPPH_0816 |
| chromosome | 982735 | 983022 | 288 | * | rhpR_rhpS-Mutant_KB_R1_peak_50 | 844 | 982619 | 983014 | + | overlapEnd | 116 | 8 | NearestLocation | PSPPH_0821 |
| chromosome | 1024797 | 1024930 | 134 | * | rhpR_rhpS-Mutant_KB_R1_peak_51 | 880 | 1023074 | 1024549 | - | upstream | -248 | 248 | NearestLocation | PSPPH_0856 |
| chromosome | 1037873 | 1037980 | 108 | * | rhpR_rhpS-Mutant_KB_R1_peak_52 | 892 | 1038016 | 1040337 | + | upstream | -143 | 36 | NearestLocation | PSPPH_0868 |
| chromosome | 1041956 | 1042072 | 117 | * | rhpR_rhpS-Mutant_KB_R1_peak_53 | 895 | 1041434 | 1041850 | - | upstream | -106 | 106 | NearestLocation | PSPPH_0871 |
| chromosome | 1125700 | 1126057 | 358 | * | rhpR_rhpS-Mutant_KB_R1_peak_54 | 975 | 1125905 | 1125981 | - | includeFeature | 281 | 76 | NearestLocation | PSPPH_0948 |
| chromosome | 1126371 | 1126492 | 122 | * | rhpR_rhpS-Mutant_KB_R1_peak_55 | 975 | 1125905 | 1125981 | - | upstream | -390 | 390 | NearestLocation | PSPPH_0948 |
| chromosome | 1127340 | 1127494 | 155 | * | rhpR_rhpS-Mutant_KB_R1_peak_56 | 977 | 1127559 | 1129616 | + | upstream | -219 | 65 | NearestLocation | PSPPH_0950 |
| chromosome | 1132895 | 1132974 | 80 | * | rhpR_rhpS-Mutant_KB_R1_peak_57 | 980 | 1131805 | 1132836 | - | upstream | -59 | 59 | NearestLocation | PSPPH_0953 |
| chromosome | 1135389 | 1135547 | 159 | * | rhpR_rhpS-Mutant_KB_R1_peak_58 | 983 | 1135374 | 1136954 | + | inside | 15 | 15 | NearestLocation | PSPPH_0956 |
| chromosome | 1140057 | 1140200 | 144 | * | rhpR_rhpS-Mutant_KB_R1_peak_59 | 985 | 1139722 | 1140804 | + | inside | 335 | 335 | NearestLocation | PSPPH_0958 |
| chromosome | 1148355 | 1148461 | 107 | * | rhpR_rhpS-Mutant_KB_R1_peak_60 | 992 | 1147760 | 1149175 | - | inside | 820 | 595 | NearestLocation | PSPPH_0965 |
| chromosome | 1150013 | 1150177 | 165 | * | rhpR_rhpS-Mutant_KB_R1_peak_61 | 993 | 1149304 | 1149723 | + | downstream | 709 | 290 | NearestLocation | PSPPH_0966 |
| chromosome | 1153581 | 1153733 | 153 | * | rhpR_rhpS-Mutant_KB_R1_peak_62 | 996 | 1152260 | 1153360 | - | upstream | -221 | 221 | NearestLocation | PSPPH_0969 |
| chromosome | 1186093 | 1186271 | 179 | * | rhpR_rhpS-Mutant_KB_R1_peak_63 | 1018 | 1185154 | 1186095 | - | overlapStart | 2 | 2 | NearestLocation | PSPPH_0991 |
| chromosome | 1189588 | 1189724 | 137 | * | rhpR_rhpS-Mutant_KB_R1_peak_64 | 1023 | 1189698 | 1190975 | + | overlapStart | -110 | 26 | NearestLocation | PSPPH_0996 |
| chromosome | 1194275 | 1194407 | 133 | * | rhpR_rhpS-Mutant_KB_R1_peak_65 | 1028 | 1194579 | 1195097 | + | upstream | -304 | 172 | NearestLocation | PSPPH_1001 |
| chromosome | 1223066 | 1223320 | 255 | * | rhpR_rhpS-Mutant_KB_R1_peak_66 | 1062 | 1223313 | 1224131 | + | overlapStart | -247 | 7 | NearestLocation | PSPPH_1035 |
| chromosome | 1232403 | 1232569 | 167 | * | rhpR_rhpS-Mutant_KB_R1_peak_67 | 1069 | 1232666 | 1235402 | + | upstream | -263 | 97 | NearestLocation | PSPPH_1042 |
| chromosome | 1232403 | 1232569 | 167 | * | rhpR_rhpS-Mutant_KB_R1_peak_67 | 1070 | 1232666 | 1235402 | + | upstream | -263 | 97 | NearestLocation | PSPPH_1042 |
| chromosome | 1267155 | 1267244 | 90 | * | rhpR_rhpS-Mutant_KB_R1_peak_68 | 1105 | 1266510 | 1267055 | + | downstream | 645 | 100 | NearestLocation | PSPPH_1077 |
| chromosome | 1277248 | 1277412 | 165 | * | rhpR_rhpS-Mutant_KB_R1_peak_69 | 1112 | 1277491 | 1277757 | - | downstream | 509 | 79 | NearestLocation | PSPPH_1084 |
| chromosome | 1279082 | 1279250 | 169 | * | rhpR_rhpS-Mutant_KB_R1_peak_70 | 1114 | 1278735 | 1279067 | - | upstream | -15 | 15 | NearestLocation | PSPPH_1086 |
| chromosome | 1284131 | 1284212 | 82 | * | rhpR_rhpS-Mutant_KB_R1_peak_71 | 1119 | 1283687 | 1284115 | - | upstream | -16 | 16 | NearestLocation | PSPPH_1090 |
| chromosome | 1318729 | 1319046 | 318 | * | rhpR_rhpS-Mutant_KB_R1_peak_72 | 1148 | 1317259 | 1318575 | - | upstream | -154 | 154 | NearestLocation | PSPPH_1118 |
| chromosome | 1319322 | 1319434 | 113 | * | rhpR_rhpS-Mutant_KB_R1_peak_73 | 1149 | 1319388 | 1320167 | - | overlapEnd | 845 | 46 | NearestLocation | PSPPH_1119 |
| chromosome | 1320232 | 1320415 | 184 | * | rhpR_rhpS-Mutant_KB_R1_peak_74 | 1150 | 1320350 | 1321549 | + | overlapStart | -118 | 65 | NearestLocation | PSPPH_1120 |
| chromosome | 1345958 | 1346098 | 141 | * | rhpR_rhpS-Mutant_KB_R1_peak_75 | 1176 | 1345074 | 1345496 | - | upstream | -462 | 462 | NearestLocation | PSPPH_1146 |
| chromosome | 1347960 | 1348101 | 142 | * | rhpR_rhpS-Mutant_KB_R1_peak_76 | 1177 | 1345493 | 1347374 | - | upstream | -586 | 586 | NearestLocation | PSPPH_1147 |
| chromosome | 1347960 | 1348101 | 142 | * | rhpR_rhpS-Mutant_KB_R1_peak_76 | 1178 | 1345493 | 1347374 | - | upstream | -586 | 586 | NearestLocation | PSPPH_1147 |
| chromosome | 1350179 | 1350347 | 169 | * | rhpR_rhpS-Mutant_KB_R1_peak_77 | 1180 | 1349050 | 1350198 | - | overlapStart | 19 | 19 | NearestLocation | PSPPH_1149 |
| chromosome | 1359375 | 1359472 | 98 | * | rhpR_rhpS-Mutant_KB_R1_peak_78 | 1188 | 1357817 | 1359421 | - | overlapStart | 46 | 46 | NearestLocation | PSPPH_1157 |
| chromosome | 1362280 | 1362517 | 238 | * | rhpR_rhpS-Mutant_KB_R1_peak_79 | 1191 | 1361947 | 1362159 | - | upstream | -121 | 121 | NearestLocation | PSPPH_1160 |
| chromosome | 1387191 | 1387329 | 139 | * | rhpR_rhpS-Mutant_KB_R1_peak_80 | 1212 | 1387352 | 1388638 | + | upstream | -161 | 23 | NearestLocation | PSPPH_1181 |
| chromosome | 1418099 | 1418180 | 82 | * | rhpR_rhpS-Mutant_KB_R1_peak_81 | 1239 | 1417026 | 1417922 | - | upstream | -177 | 177 | NearestLocation | PSPPH_1207 |
| chromosome | 1419220 | 1419414 | 195 | * | rhpR_rhpS-Mutant_KB_R1_peak_82 | 1242 | 1419508 | 1420449 | + | upstream | -288 | 94 | NearestLocation | PSPPH_1210 |
| chromosome | 1484357 | 1484825 | 469 | * | rhpR_rhpS-Mutant_KB_R1_peak_83 | 1309 | 1485638 | 1486558 | + | upstream | -1281 | 813 | NearestLocation | PSPPH_1270 |
| chromosome | 1543564 | 1543650 | 87 | * | rhpR_rhpS-Mutant_KB_R1_peak_84 | 1373 | 1543651 | 1545120 | + | upstream | -87 | 1 | NearestLocation | PSPPH_1333 |
| chromosome | 1587512 | 1587650 | 139 | * | rhpR_rhpS-Mutant_KB_R1_peak_85 | 1409 | 1587441 | 1588172 | + | inside | 71 | 71 | NearestLocation | PSPPH_1368 |
| chromosome | 1594548 | 1594669 | 122 | * | rhpR_rhpS-Mutant_KB_R1_peak_86 | 1414 | 1595303 | 1595491 | - | downstream | 943 | 634 | NearestLocation | PSPPH_1373 |
| chromosome | 1596455 | 1596541 | 87 | * | rhpR_rhpS-Mutant_KB_R1_peak_87 | 1416 | 1596630 | 1596812 | - | downstream | 357 | 89 | NearestLocation | PSPPH_1375 |
| chromosome | 1772107 | 1772193 | 87 | * | rhpR_rhpS-Mutant_KB_R1_peak_88 | 1567 | 1772229 | 1773839 | + | upstream | -122 | 36 | NearestLocation | PSPPH_1523 |
| chromosome | 1904142 | 1904257 | 116 | * | rhpR_rhpS-Mutant_KB_R1_peak_89 | 1693 | 1903900 | 1904643 | + | inside | 242 | 242 | NearestLocation | PSPPH_1641 |
| chromosome | 1930921 | 1931076 | 156 | * | rhpR_rhpS-Mutant_KB_R1_peak_90 | 1721 | 1929241 | 1930776 | - | upstream | -145 | 145 | NearestLocation | PSPPH_1669 |
| chromosome | 1952459 | 1952751 | 293 | * | rhpR_rhpS-Mutant_KB_R1_peak_91 | 1741 | 1952689 | 1952764 | + | overlapStart | -230 | 13 | NearestLocation | PSPPH_1689 |
| chromosome | 1957045 | 1957149 | 105 | * | rhpR_rhpS-Mutant_KB_R1_peak_92 | 1746 | 1957205 | 1958197 | - | downstream | 1152 | 56 | NearestLocation | PSPPH_1694 |
| chromosome | 1961958 | 1962055 | 98 | * | rhpR_rhpS-Mutant_KB_R1_peak_93 | 1750 | 1962205 | 1962846 | + | upstream | -247 | 150 | NearestLocation | PSPPH_1698 |
| chromosome | 1966456 | 1966572 | 117 | * | rhpR_rhpS-Mutant_KB_R1_peak_94 | 1753 | 1966990 | 1967262 | + | upstream | -534 | 418 | NearestLocation | PSPPH_1701 |
| chromosome | 1999686 | 1999866 | 181 | * | rhpR_rhpS-Mutant_KB_R1_peak_95 | 1777 | 1998652 | 1999770 | - | overlapStart | 84 | 84 | NearestLocation | PSPPH_1725 |
| chromosome | 2084996 | 2085081 | 86 | * | rhpR_rhpS-Mutant_KB_R1_peak_96 | 1835 | 2084780 | 2085943 | + | inside | 216 | 216 | NearestLocation | PSPPH_1780 |
| chromosome | 2109652 | 2109877 | 226 | * | rhpR_rhpS-Mutant_KB_R1_peak_97 | 1861 | 2109063 | 2109713 | - | overlapStart | 61 | 61 | NearestLocation | PSPPH_1805 |
| chromosome | 2109930 | 2110243 | 314 | * | rhpR_rhpS-Mutant_KB_R1_peak_98 | 1861 | 2109063 | 2109713 | - | upstream | -217 | 217 | NearestLocation | PSPPH_1805 |
| chromosome | 2127268 | 2127386 | 119 | * | rhpR_rhpS-Mutant_KB_R1_peak_99 | 1883 | 2127447 | 2127788 | + | upstream | -179 | 61 | NearestLocation | PSPPH_1827 |
| chromosome | 2137907 | 2138002 | 96 | * | rhpR_rhpS-Mutant_KB_R1_peak_100 | 1897 | 2137193 | 2137723 | - | upstream | -184 | 184 | NearestLocation | PSPPH_1840 |
| chromosome | 2168329 | 2168430 | 102 | * | rhpR_rhpS-Mutant_KB_R1_peak_101 | 1925 | 2168726 | 2169211 | + | upstream | -397 | 296 | NearestLocation | PSPPH_1868 |
| chromosome | 2190379 | 2190531 | 153 | * | rhpR_rhpS-Mutant_KB_R1_peak_102 | 1948 | 2190515 | 2190874 | + | overlapStart | -136 | 16 | NearestLocation | PSPPH_1888 |
| chromosome | 2201469 | 2201552 | 84 | * | rhpR_rhpS-Mutant_KB_R1_peak_103 | 1957 | 2200419 | 2201546 | - | overlapStart | 77 | 6 | NearestLocation | PSPPH_1897 |
| chromosome | 2208915 | 2209034 | 120 | * | rhpR_rhpS-Mutant_KB_R1_peak_104 | 1963 | 2208997 | 2209428 | + | overlapStart | -82 | 37 | NearestLocation | PSPPH_1903 |
| chromosome | 2318405 | 2318507 | 103 | * | rhpR_rhpS-Mutant_KB_R1_peak_105 | 2030 | 2318480 | 2319223 | + | overlapStart | -75 | 27 | NearestLocation | PSPPH_1970 |
| chromosome | 2322026 | 2322136 | 111 | * | rhpR_rhpS-Mutant_KB_R1_peak_106 | 2036 | 2321961 | 2322335 | + | inside | 65 | 65 | NearestLocation | PSPPH_1976 |
| chromosome | 2350478 | 2350578 | 101 | * | rhpR_rhpS-Mutant_KB_R1_peak_107 | 2064 | 2350640 | 2351398 | - | downstream | 920 | 62 | NearestLocation | PSPPH_2002 |
| chromosome | 2351358 | 2351587 | 230 | * | rhpR_rhpS-Mutant_KB_R1_peak_108 | 2064 | 2350640 | 2351398 | - | overlapStart | 40 | 40 | NearestLocation | PSPPH_2002 |
| chromosome | 2352569 | 2352701 | 133 | * | rhpR_rhpS-Mutant_KB_R1_peak_109 | 2065 | 2351514 | 2352539 | - | upstream | -30 | 30 | NearestLocation | PSPPH_2003 |
| chromosome | 2353088 | 2353198 | 111 | * | rhpR_rhpS-Mutant_KB_R1_peak_110 | 2066 | 2352536 | 2353285 | - | inside | 197 | 87 | NearestLocation | PSPPH_2004 |
| chromosome | 2353383 | 2353793 | 411 | * | rhpR_rhpS-Mutant_KB_R1_peak_111 | 2067 | 2353651 | 2354559 | + | overlapStart | -268 | 142 | NearestLocation | PSPPH_2005 |
| chromosome | 2422857 | 2423039 | 183 | * | rhpR_rhpS-Mutant_KB_R1_peak_112 | 2133 | 2423246 | 2424280 | + | upstream | -389 | 207 | NearestLocation | PSPPH_2068 |
| chromosome | 2434285 | 2434361 | 77 | * | rhpR_rhpS-Mutant_KB_R1_peak_113 | 2140 | 2434443 | 2435018 | - | downstream | 733 | 82 | NearestLocation | PSPPH_2075 |
| chromosome | 2437903 | 2438091 | 189 | * | rhpR_rhpS-Mutant_KB_R1_peak_114 | 2143 | 2437666 | 2437992 | - | overlapStart | 89 | 89 | NearestLocation | PSPPH_2078 |
| chromosome | 2440689 | 2440830 | 142 | * | rhpR_rhpS-Mutant_KB_R1_peak_115 | 2145 | 2440865 | 2443255 | + | upstream | -176 | 35 | NearestLocation | PSPPH_2080 |
| chromosome | 2478886 | 2479063 | 178 | * | rhpR_rhpS-Mutant_KB_R1_peak_116 | 2184 | 2478966 | 2479223 | + | overlapStart | -80 | 80 | NearestLocation | PSPPH_2119 |
| chromosome | 2489324 | 2489442 | 119 | * | rhpR_rhpS-Mutant_KB_R1_peak_117 | 2197 | 2489070 | 2489291 | - | upstream | -33 | 33 | NearestLocation | PSPPH_2132 |
| chromosome | 2497677 | 2497847 | 171 | * | rhpR_rhpS-Mutant_KB_R1_peak_118 | 2206 | 2497808 | 2498110 | + | overlapStart | -131 | 39 | NearestLocation | PSPPH_2141 |
| chromosome | 2515238 | 2515337 | 100 | * | rhpR_rhpS-Mutant_KB_R1_peak_119 | 2225 | 2514967 | 2516157 | + | inside | 271 | 271 | NearestLocation | PSPPH_2159 |
| chromosome | 2540838 | 2540948 | 111 | * | rhpR_rhpS-Mutant_KB_R1_peak_120 | 2255 | 2539472 | 2540776 | - | upstream | -62 | 62 | NearestLocation | PSPPH_2185 |
| chromosome | 2541094 | 2541266 | 173 | * | rhpR_rhpS-Mutant_KB_R1_peak_121 | 2256 | 2541290 | 2542897 | + | upstream | -196 | 24 | NearestLocation | PSPPH_2186 |
| chromosome | 2550576 | 2550663 | 88 | * | rhpR_rhpS-Mutant_KB_R1_peak_122 | 2264 | 2550022 | 2550513 | - | upstream | -63 | 63 | NearestLocation | PSPPH_2194 |
| chromosome | 2554348 | 2554465 | 118 | * | rhpR_rhpS-Mutant_KB_R1_peak_123 | 2267 | 2553044 | 2554293 | - | upstream | -55 | 55 | NearestLocation | PSPPH_2197 |
| chromosome | 2554348 | 2554465 | 118 | * | rhpR_rhpS-Mutant_KB_R1_peak_123 | 2268 | 2553044 | 2554293 | - | upstream | -55 | 55 | NearestLocation | PSPPH_2197 |
| chromosome | 2575966 | 2576065 | 100 | * | rhpR_rhpS-Mutant_KB_R1_peak_124 | 2292 | 2576054 | 2579539 | + | overlapStart | -88 | 11 | NearestLocation | PSPPH_2220 |
| chromosome | 2644978 | 2645076 | 99 | * | rhpR_rhpS-Mutant_KB_R1_peak_125 | 2353 | 2645116 | 2646675 | + | upstream | -138 | 40 | NearestLocation | PSPPH_2279 |
| chromosome | 2936631 | 2936741 | 111 | * | rhpR_rhpS-Mutant_KB_R1_peak_126 | 2630 | 2934900 | 2936543 | - | upstream | -88 | 88 | NearestLocation | PSPPH_2545 |
| chromosome | 2981695 | 2981823 | 129 | * | rhpR_rhpS-Mutant_KB_R1_peak_127 | 2675 | 2981819 | 2984701 | + | overlapStart | -124 | 4 | NearestLocation | PSPPH_2590 |
| chromosome | 3018419 | 3018533 | 115 | * | rhpR_rhpS-Mutant_KB_R1_peak_128 | 2704 | 3016634 | 3018292 | - | upstream | -127 | 127 | NearestLocation | PSPPH_2618 |
| chromosome | 3018915 | 3018990 | 76 | * | rhpR_rhpS-Mutant_KB_R1_peak_129 | 2705 | 3018875 | 3019948 | + | inside | 40 | 40 | NearestLocation | PSPPH_2619 |
| chromosome | 3033856 | 3033974 | 119 | * | rhpR_rhpS-Mutant_KB_R1_peak_130 | 2715 | 3032695 | 3033858 | - | overlapStart | 2 | 2 | NearestLocation | PSPPH_2628 |
| chromosome | 3033856 | 3033974 | 119 | * | rhpR_rhpS-Mutant_KB_R1_peak_130 | 2716 | 3032695 | 3033858 | - | overlapStart | 2 | 2 | NearestLocation | PSPPH_2628 |
| chromosome | 3034284 | 3034361 | 78 | * | rhpR_rhpS-Mutant_KB_R1_peak_131 | 2715 | 3032695 | 3033858 | - | upstream | -426 | 426 | NearestLocation | PSPPH_2628 |
| chromosome | 3034284 | 3034361 | 78 | * | rhpR_rhpS-Mutant_KB_R1_peak_131 | 2716 | 3032695 | 3033858 | - | upstream | -426 | 426 | NearestLocation | PSPPH_2628 |
| chromosome | 3034493 | 3034582 | 90 | * | rhpR_rhpS-Mutant_KB_R1_peak_132 | 2715 | 3032695 | 3033858 | - | upstream | -635 | 635 | NearestLocation | PSPPH_2628 |
| chromosome | 3034493 | 3034582 | 90 | * | rhpR_rhpS-Mutant_KB_R1_peak_132 | 2716 | 3032695 | 3033858 | - | upstream | -635 | 635 | NearestLocation | PSPPH_2628 |
| chromosome | 3077391 | 3077489 | 99 | * | rhpR_rhpS-Mutant_KB_R1_peak_133 | 2742 | 3076506 | 3077354 | - | upstream | -37 | 37 | NearestLocation | PSPPH_2653 |
| chromosome | 3163031 | 3163178 | 148 | * | rhpR_rhpS-Mutant_KB_R1_peak_134 | 2821 | 3162689 | 3163060 | - | overlapStart | 29 | 29 | NearestLocation | PSPPH_2731 |
| chromosome | 3178813 | 3178960 | 148 | * | rhpR_rhpS-Mutant_KB_R1_peak_135 | 2837 | 3179070 | 3179609 | + | upstream | -257 | 110 | NearestLocation | PSPPH_2747 |
| chromosome | 3225874 | 3225954 | 81 | * | rhpR_rhpS-Mutant_KB_R1_peak_136 | 2878 | 3225566 | 3225958 | - | inside | 84 | 4 | NearestLocation | PSPPH_2787 |
| chromosome | 3231118 | 3231236 | 119 | * | rhpR_rhpS-Mutant_KB_R1_peak_137 | 2882 | 3230334 | 3231464 | - | inside | 346 | 228 | NearestLocation | PSPPH_2791 |
| chromosome | 3231564 | 3231707 | 144 | * | rhpR_rhpS-Mutant_KB_R1_peak_138 | 2883 | 3231624 | 3231971 | + | overlapStart | -60 | 60 | NearestLocation | PSPPH_2792 |
| chromosome | 3271715 | 3271827 | 113 | * | rhpR_rhpS-Mutant_KB_R1_peak_139 | 2919 | 3272037 | 3272318 | + | upstream | -322 | 210 | NearestLocation | PSPPH_2825 |
| chromosome | 3533526 | 3533646 | 121 | * | rhpR_rhpS-Mutant_KB_R1_peak_140 | 3143 | 3533706 | 3534569 | + | upstream | -180 | 60 | NearestLocation | PSPPH_3042 |
| chromosome | 3575363 | 3575625 | 263 | * | rhpR_rhpS-Mutant_KB_R1_peak_141 | 3181 | 3573928 | 3575358 | - | upstream | -5 | 5 | NearestLocation | PSPPH_3076 |
| chromosome | 3575363 | 3575625 | 263 | * | rhpR_rhpS-Mutant_KB_R1_peak_141 | 3182 | 3573928 | 3575358 | - | upstream | -5 | 5 | NearestLocation | PSPPH_3076 |
| chromosome | 3575879 | 3575954 | 76 | * | rhpR_rhpS-Mutant_KB_R1_peak_142 | 3181 | 3573928 | 3575358 | - | upstream | -521 | 521 | NearestLocation | PSPPH_3076 |
| chromosome | 3575879 | 3575954 | 76 | * | rhpR_rhpS-Mutant_KB_R1_peak_142 | 3182 | 3573928 | 3575358 | - | upstream | -521 | 521 | NearestLocation | PSPPH_3076 |
| chromosome | 3596205 | 3596300 | 96 | * | rhpR_rhpS-Mutant_KB_R1_peak_143 | 3205 | 3595796 | 3596158 | - | upstream | -47 | 47 | NearestLocation | PSPPH_3098 |
| chromosome | 3624985 | 3625141 | 157 | * | rhpR_rhpS-Mutant_KB_R1_peak_144 | 3230 | 3624094 | 3625017 | + | overlapEnd | 891 | 32 | NearestLocation | PSPPH_3123 |
| chromosome | 3630510 | 3630590 | 81 | * | rhpR_rhpS-Mutant_KB_R1_peak_145 | 3235 | 3630124 | 3630432 | - | upstream | -78 | 78 | NearestLocation | PSPPH_3128 |
| chromosome | 3686390 | 3686561 | 172 | * | rhpR_rhpS-Mutant_KB_R1_peak_146 | 3289 | 3686569 | 3686865 | + | upstream | -179 | 8 | NearestLocation | PSPPH_3179 |
| chromosome | 3733190 | 3733274 | 85 | * | rhpR_rhpS-Mutant_KB_R1_peak_147 | 3333 | 3732095 | 3733012 | - | upstream | -178 | 178 | NearestLocation | PSPPH_3221 |
| chromosome | 3836471 | 3836875 | 405 | * | rhpR_rhpS-Mutant_KB_R1_peak_148 | 3428 | 3836826 | 3838463 | + | overlapStart | -355 | 49 | NearestLocation | PSPPH_3315 |
| chromosome | 3857198 | 3857309 | 112 | * | rhpR_rhpS-Mutant_KB_R1_peak_149 | 3445 | 3857400 | 3857924 | + | upstream | -202 | 91 | NearestLocation | PSPPH_3331 |
| chromosome | 3896021 | 3896149 | 129 | * | rhpR_rhpS-Mutant_KB_R1_peak_150 | 3480 | 3893910 | 3896039 | - | overlapStart | 18 | 18 | NearestLocation | PSPPH_3366 |
| chromosome | 3900477 | 3900564 | 88 | * | rhpR_rhpS-Mutant_KB_R1_peak_151 | 3486 | 3900030 | 3900488 | - | overlapStart | 11 | 11 | NearestLocation | PSPPH_3372 |
| chromosome | 3902040 | 3902187 | 148 | * | rhpR_rhpS-Mutant_KB_R1_peak_152 | 3488 | 3901528 | 3902028 | - | upstream | -12 | 12 | NearestLocation | PSPPH_3374 |
| chromosome | 3937419 | 3937520 | 102 | * | rhpR_rhpS-Mutant_KB_R1_peak_153 | 3516 | 3936650 | 3937390 | - | upstream | -29 | 29 | NearestLocation | PSPPH_3402 |
| chromosome | 3946854 | 3946995 | 142 | * | rhpR_rhpS-Mutant_KB_R1_peak_154 | 3528 | 3946917 | 3947675 | + | overlapStart | -63 | 63 | NearestLocation | PSPPH_3414 |
| chromosome | 4012876 | 4012997 | 122 | * | rhpR_rhpS-Mutant_KB_R1_peak_155 | 3591 | 4013029 | 4015101 | + | upstream | -153 | 32 | NearestLocation | PSPPH_3473 |
| chromosome | 4031443 | 4031598 | 156 | * | rhpR_rhpS-Mutant_KB_R1_peak_156 | 3608 | 4031056 | 4031223 | + | downstream | 387 | 220 | NearestLocation | PSPPH_3490 |
| chromosome | 4043157 | 4043243 | 87 | * | rhpR_rhpS-Mutant_KB_R1_peak_157 | 3624 | 4043310 | 4043636 | + | upstream | -153 | 67 | NearestLocation | PSPPH_3504 |
| chromosome | 4069073 | 4069151 | 79 | * | rhpR_rhpS-Mutant_KB_R1_peak_158 | 3649 | 4069214 | 4069480 | + | upstream | -141 | 63 | NearestLocation | PSPPH_3529 |
| chromosome | 4102131 | 4102225 | 95 | * | rhpR_rhpS-Mutant_KB_R1_peak_159 | 3680 | 4097194 | 4102053 | - | upstream | -78 | 78 | NearestLocation | PSPPH_3560 |
| chromosome | 4197097 | 4197292 | 196 | * | rhpR_rhpS-Mutant_KB_R1_peak_160 | 3776 | 4197240 | 4197809 | + | overlapStart | -143 | 52 | NearestLocation | PSPPH_3654 |
| chromosome | 4202455 | 4202532 | 78 | * | rhpR_rhpS-Mutant_KB_R1_peak_161 | 3780 | 4201524 | 4202504 | - | overlapStart | 49 | 28 | NearestLocation | PSPPH_3658 |
| chromosome | 4222319 | 4222449 | 131 | * | rhpR_rhpS-Mutant_KB_R1_peak_162 | 3796 | 4221051 | 4221836 | - | upstream | -483 | 483 | NearestLocation | PSPPH_3674 |
| chromosome | 4233219 | 4233323 | 105 | * | rhpR_rhpS-Mutant_KB_R1_peak_163 | 3807 | 4232944 | 4233237 | + | overlapEnd | 275 | 18 | NearestLocation | PSPPH_3685 |
| chromosome | 4233467 | 4233664 | 198 | * | rhpR_rhpS-Mutant_KB_R1_peak_164 | 3807 | 4232944 | 4233237 | + | downstream | 523 | 230 | NearestLocation | PSPPH_3685 |
| chromosome | 4236163 | 4236313 | 151 | * | rhpR_rhpS-Mutant_KB_R1_peak_165 | 3810 | 4236460 | 4236762 | + | upstream | -297 | 147 | NearestLocation | PSPPH_3688 |
| chromosome | 4285415 | 4285570 | 156 | * | rhpR_rhpS-Mutant_KB_R1_peak_166 | 3859 | 4284455 | 4285192 | - | upstream | -223 | 223 | NearestLocation | PSPPH_3737 |
| chromosome | 4332307 | 4332401 | 95 | * | rhpR_rhpS-Mutant_KB_R1_peak_167 | 3905 | 4332394 | 4334109 | + | overlapStart | -87 | 7 | NearestLocation | PSPPH_3783 |
| chromosome | 4349840 | 4349948 | 109 | * | rhpR_rhpS-Mutant_KB_R1_peak_168 | 3920 | 4349926 | 4350852 | + | overlapStart | -86 | 22 | NearestLocation | PSPPH_3798 |
| chromosome | 4362421 | 4362591 | 171 | * | rhpR_rhpS-Mutant_KB_R1_peak_169 | 3931 | 4361212 | 4362219 | - | upstream | -202 | 202 | NearestLocation | PSPPH_3809 |
| chromosome | 4363270 | 4363352 | 83 | * | rhpR_rhpS-Mutant_KB_R1_peak_170 | 3932 | 4362323 | 4363189 | - | upstream | -81 | 81 | NearestLocation | PSPPH_3810 |
| chromosome | 4402218 | 4402422 | 205 | * | rhpR_rhpS-Mutant_KB_R1_peak_171 | 3967 | 4402378 | 4402725 | + | overlapStart | -160 | 44 | NearestLocation | PSPPH_3845 |
| chromosome | 4477578 | 4477681 | 104 | * | rhpR_rhpS-Mutant_KB_R1_peak_172 | 4044 | 4477670 | 4478281 | + | overlapStart | -92 | 11 | NearestLocation | PSPPH_3920 |
| chromosome | 4514369 | 4514474 | 106 | * | rhpR_rhpS-Mutant_KB_R1_peak_173 | 4075 | 4512549 | 4514336 | - | upstream | -33 | 33 | NearestLocation | PSPPH_3951 |
| chromosome | 4518284 | 4518436 | 153 | * | rhpR_rhpS-Mutant_KB_R1_peak_174 | 4079 | 4517832 | 4518413 | - | overlapStart | 129 | 23 | NearestLocation | PSPPH_3955 |
| chromosome | 4552014 | 4552137 | 124 | * | rhpR_rhpS-Mutant_KB_R1_peak_175 | 4112 | 4551605 | 4552324 | - | inside | 310 | 187 | NearestLocation | PSPPH_3986 |
| chromosome | 4561128 | 4561210 | 83 | * | rhpR_rhpS-Mutant_KB_R1_peak_176 | 4122 | 4559857 | 4561077 | - | upstream | -51 | 51 | NearestLocation | PSPPH_3995 |
| chromosome | 4578378 | 4578534 | 157 | * | rhpR_rhpS-Mutant_KB_R1_peak_177 | 4136 | 4578772 | 4579128 | + | upstream | -394 | 238 | NearestLocation | PSPPH_4007 |
| chromosome | 4600672 | 4600828 | 157 | * | rhpR_rhpS-Mutant_KB_R1_peak_178 | 4154 | 4600070 | 4600450 | + | downstream | 602 | 222 | NearestLocation | PSPPH_4025 |
| chromosome | 4612347 | 4612423 | 77 | * | rhpR_rhpS-Mutant_KB_R1_peak_179 | 4168 | 4611823 | 4612182 | - | upstream | -165 | 165 | NearestLocation | PSPPH_4037 |
| chromosome | 4617421 | 4617563 | 143 | * | rhpR_rhpS-Mutant_KB_R1_peak_180 | 4172 | 4615638 | 4617161 | + | downstream | 1783 | 260 | NearestLocation | PSPPH_4041 |
| chromosome | 4687286 | 4687364 | 79 | * | rhpR_rhpS-Mutant_KB_R1_peak_181 | 4239 | 4686624 | 4687535 | - | inside | 249 | 171 | NearestLocation | PSPPH_4102 |
| chromosome | 4736509 | 4736594 | 86 | * | rhpR_rhpS-Mutant_KB_R1_peak_182 | 4289 | 4736613 | 4736921 | + | upstream | -104 | 19 | NearestLocation | PSPPH_4152 |
| chromosome | 4758236 | 4758321 | 86 | * | rhpR_rhpS-Mutant_KB_R1_peak_183 | 4310 | 4758378 | 4758665 | + | upstream | -142 | 57 | NearestLocation | PSPPH_4173 |
| chromosome | 4780795 | 4780999 | 205 | * | rhpR_rhpS-Mutant_KB_R1_peak_184 | 4329 | 4780755 | 4780831 | - | overlapStart | 36 | 36 | NearestLocation | PSPPH_4192 |
| chromosome | 4786421 | 4786504 | 84 | * | rhpR_rhpS-Mutant_KB_R1_peak_185 | 4335 | 4784486 | 4786399 | - | upstream | -22 | 22 | NearestLocation | PSPPH_4198 |
| chromosome | 4800095 | 4800333 | 239 | * | rhpR_rhpS-Mutant_KB_R1_peak_186 | 4347 | 4800318 | 4800839 | + | overlapStart | -223 | 15 | NearestLocation | PSPPH_4210 |
| chromosome | 4804249 | 4804557 | 309 | * | rhpR_rhpS-Mutant_KB_R1_peak_187 | 4353 | 4804338 | 4804725 | + | overlapStart | -89 | 89 | NearestLocation | PSPPH_4216 |
| chromosome | 4838356 | 4838557 | 202 | * | rhpR_rhpS-Mutant_KB_R1_peak_188 | 4379 | 4838479 | 4839108 | + | overlapStart | -123 | 78 | NearestLocation | PSPPH_4241 |
| chromosome | 4842937 | 4843137 | 201 | * | rhpR_rhpS-Mutant_KB_R1_peak_189 | 4383 | 4843131 | 4843619 | + | overlapStart | -194 | 6 | NearestLocation | PSPPH_4245 |
| chromosome | 4844028 | 4844319 | 292 | * | rhpR_rhpS-Mutant_KB_R1_peak_190 | 4384 | 4843633 | 4844088 | - | overlapStart | 60 | 60 | NearestLocation | PSPPH_4246 |
| chromosome | 4847788 | 4847871 | 84 | * | rhpR_rhpS-Mutant_KB_R1_peak_191 | 4387 | 4848143 | 4848382 | - | downstream | 594 | 272 | NearestLocation | PSPPH_4249 |
| chromosome | 4848187 | 4848318 | 132 | * | rhpR_rhpS-Mutant_KB_R1_peak_192 | 4387 | 4848143 | 4848382 | - | inside | 195 | 44 | NearestLocation | PSPPH_4249 |
| chromosome | 4908991 | 4909202 | 212 | * | rhpR_rhpS-Mutant_KB_R1_peak_193 | 4436 | 4907552 | 4908361 | - | upstream | -630 | 630 | NearestLocation | PSPPH_4298 |
| chromosome | 4931251 | 4931381 | 131 | * | rhpR_rhpS-Mutant_KB_R1_peak_194 | 4455 | 4930056 | 4931276 | - | overlapStart | 25 | 25 | NearestLocation | PSPPH_4317 |
| chromosome | 4935714 | 4935812 | 99 | * | rhpR_rhpS-Mutant_KB_R1_peak_195 | 4459 | 4936398 | 4937021 | - | downstream | 1307 | 586 | NearestLocation | PSPPH_4321 |
| chromosome | 4946901 | 4947043 | 143 | * | rhpR_rhpS-Mutant_KB_R1_peak_196 | 4466 | 4947036 | 4948289 | + | overlapStart | -135 | 7 | NearestLocation | PSPPH_4327 |
| chromosome | 4985769 | 4985899 | 131 | * | rhpR_rhpS-Mutant_KB_R1_peak_197 | 4503 | 4985442 | 4985747 | + | downstream | 327 | 22 | NearestLocation | PSPPH_4363 |
| chromosome | 4994751 | 4994864 | 114 | * | rhpR_rhpS-Mutant_KB_R1_peak_198 | 4513 | 4995109 | 4995801 | + | upstream | -358 | 245 | NearestLocation | PSPPH_4373 |
| chromosome | 5010424 | 5010501 | 78 | * | rhpR_rhpS-Mutant_KB_R1_peak_199 | 4526 | 5010033 | 5010425 | - | overlapStart | 1 | 1 | NearestLocation | PSPPH_4386 |
| chromosome | 5043765 | 5043848 | 84 | * | rhpR_rhpS-Mutant_KB_R1_peak_200 | 4558 | 5043760 | 5044233 | + | inside | 5 | 5 | NearestLocation | PSPPH_4418 |
| chromosome | 5087822 | 5088020 | 199 | * | rhpR_rhpS-Mutant_KB_R1_peak_201 | 4593 | 5087883 | 5088518 | - | overlapEnd | 696 | 61 | NearestLocation | PSPPH_4453 |
| chromosome | 5172318 | 5172471 | 154 | * | rhpR_rhpS-Mutant_KB_R1_peak_202 | 4678 | 5172524 | 5173330 | + | upstream | -206 | 53 | NearestLocation | PSPPH_4538 |
| chromosome | 5173255 | 5173387 | 133 | * | rhpR_rhpS-Mutant_KB_R1_peak_203 | 4679 | 5173332 | 5175449 | + | overlapStart | -77 | 55 | NearestLocation | PSPPH_4539 |
| chromosome | 5177196 | 5177331 | 136 | * | rhpR_rhpS-Mutant_KB_R1_peak_204 | 4682 | 5176817 | 5177851 | + | inside | 379 | 379 | NearestLocation | PSPPH_4541 |
| chromosome | 5180056 | 5180156 | 101 | * | rhpR_rhpS-Mutant_KB_R1_peak_205 | 4685 | 5179499 | 5180248 | - | inside | 192 | 92 | NearestLocation | PSPPH_4544 |
| chromosome | 5197745 | 5197821 | 77 | * | rhpR_rhpS-Mutant_KB_R1_peak_206 | 4699 | 5197852 | 5198625 | - | downstream | 880 | 31 | NearestLocation | PSPPH_4558 |
| chromosome | 5206457 | 5206551 | 95 | * | rhpR_rhpS-Mutant_KB_R1_peak_207 | 4702 | 5204697 | 5206401 | - | upstream | -56 | 56 | NearestLocation | PSPPH_4561 |
| chromosome | 5206457 | 5206551 | 95 | * | rhpR_rhpS-Mutant_KB_R1_peak_207 | 4703 | 5204697 | 5206401 | - | upstream | -56 | 56 | NearestLocation | PSPPH_4561 |
| chromosome | 5208539 | 5208618 | 80 | * | rhpR_rhpS-Mutant_KB_R1_peak_208 | 4705 | 5208582 | 5211416 | + | overlapStart | -43 | 36 | NearestLocation | PSPPH_4563 |
| chromosome | 5216582 | 5216752 | 171 | * | rhpR_rhpS-Mutant_KB_R1_peak_209 | 4712 | 5216253 | 5216609 | - | overlapStart | 27 | 27 | NearestLocation | PSPPH_4570 |
| chromosome | 5220685 | 5220791 | 107 | * | rhpR_rhpS-Mutant_KB_R1_peak_210 | 4720 | 5220254 | 5220646 | - | upstream | -39 | 39 | NearestLocation | PSPPH_4578 |
| chromosome | 5231927 | 5232092 | 166 | * | rhpR_rhpS-Mutant_KB_R1_peak_211 | 4739 | 5231556 | 5231927 | - | overlapStart | 0 | 0 | NearestLocation | PSPPH_4597 |
| chromosome | 5236475 | 5236580 | 106 | * | rhpR_rhpS-Mutant_KB_R1_peak_212 | 4740 | 5232126 | 5236325 | - | upstream | -150 | 150 | NearestLocation | PSPPH_4598 |
| chromosome | 5243527 | 5243607 | 81 | * | rhpR_rhpS-Mutant_KB_R1_peak_213 | 4746 | 5243077 | 5243610 | - | inside | 83 | 3 | NearestLocation | PSPPH_4604 |
| chromosome | 5243799 | 5243918 | 120 | * | rhpR_rhpS-Mutant_KB_R1_peak_214 | 4747 | 5243620 | 5243988 | - | inside | 189 | 70 | NearestLocation | PSPPH_4605 |
| chromosome | 5244050 | 5244240 | 191 | * | rhpR_rhpS-Mutant_KB_R1_peak_215 | 4748 | 5244035 | 5244110 | - | overlapStart | 60 | 15 | NearestLocation | PSPPH_4606 |
| chromosome | 5244309 | 5244508 | 200 | * | rhpR_rhpS-Mutant_KB_R1_peak_216 | 4749 | 5244276 | 5244351 | - | overlapStart | 42 | 33 | NearestLocation | PSPPH_4607 |
| chromosome | 5244565 | 5244674 | 110 | * | rhpR_rhpS-Mutant_KB_R1_peak_217 | 4751 | 5244476 | 5244560 | - | upstream | -5 | 5 | NearestLocation | PSPPH_4609 |
| chromosome | 5255741 | 5255869 | 129 | * | rhpR_rhpS-Mutant_KB_R1_peak_218 | 4761 | 5254425 | 5255771 | - | overlapStart | 30 | 30 | NearestLocation | PSPPH_4619 |
| chromosome | 5317891 | 5318014 | 124 | * | rhpR_rhpS-Mutant_KB_R1_peak_219 | 4824 | 5317528 | 5318289 | - | inside | 398 | 275 | NearestLocation | PSPPH_4682 |
| chromosome | 5323058 | 5323148 | 91 | * | rhpR_rhpS-Mutant_KB_R1_peak_220 | 4829 | 5323252 | 5323328 | - | downstream | 270 | 104 | NearestLocation | PSPPH_4687 |
| chromosome | 5323247 | 5323415 | 169 | * | rhpR_rhpS-Mutant_KB_R1_peak_221 | 4829 | 5323252 | 5323328 | - | includeFeature | 81 | 5 | NearestLocation | PSPPH_4687 |
| chromosome | 5341094 | 5341193 | 100 | * | rhpR_rhpS-Mutant_KB_R1_peak_222 | 4847 | 5341405 | 5341479 | + | upstream | -311 | 212 | NearestLocation | PSPPH_4704 |
| chromosome | 5346973 | 5347394 | 422 | * | rhpR_rhpS-Mutant_KB_R1_peak_223 | 4854 | 5346762 | 5347043 | - | overlapStart | 70 | 70 | NearestLocation | PSPPH_4711 |
| chromosome | 5446433 | 5446623 | 191 | * | rhpR_rhpS-Mutant_KB_R1_peak_224 | 4947 | 5446674 | 5449367 | + | upstream | -241 | 51 | NearestLocation | PSPPH_4801 |
| chromosome | 5554960 | 5555053 | 94 | * | rhpR_rhpS-Mutant_KB_R1_peak_225 | 5039 | 5555056 | 5556345 | + | upstream | -96 | 3 | NearestLocation | PSPPH_4885 |
| chromosome | 5581731 | 5581928 | 198 | * | rhpR_rhpS-Mutant_KB_R1_peak_226 | 5062 | 5580213 | 5581574 | - | upstream | -157 | 157 | NearestLocation | PSPPH_4907 |
| chromosome | 5621316 | 5621452 | 137 | * | rhpR_rhpS-Mutant_KB_R1_peak_227 | 5104 | 5621094 | 5621216 | - | upstream | -100 | 100 | NearestLocation | PSPPH_4948 |
| chromosome | 5643642 | 5644030 | 389 | * | rhpR_rhpS-Mutant_KB_R1_peak_228 | 5133 | 5643544 | 5643945 | + | overlapEnd | 98 | 85 | NearestLocation | PSPPH_4975 |
| chromosome | 5646267 | 5646440 | 174 | * | rhpR_rhpS-Mutant_KB_R1_peak_229 | 5135 | 5646120 | 5646566 | + | inside | 147 | 126 | NearestLocation | PSPPH_4977 |
| chromosome | 5652314 | 5652396 | 83 | * | rhpR_rhpS-Mutant_KB_R1_peak_230 | 5139 | 5651658 | 5652302 | - | upstream | -12 | 12 | NearestLocation | PSPPH_4981 |
| chromosome | 5684153 | 5684242 | 90 | * | rhpR_rhpS-Mutant_KB_R1_peak_231 | 5174 | 5682626 | 5683957 | - | upstream | -196 | 196 | NearestLocation | PSPPH_5016 |
| chromosome | 5692144 | 5692302 | 159 | * | rhpR_rhpS-Mutant_KB_R1_peak_232 | 5181 | 5690781 | 5692085 | - | upstream | -59 | 59 | NearestLocation | PSPPH_5023 |
| chromosome | 5727310 | 5727559 | 250 | * | rhpR_rhpS-Mutant_KB_R1_peak_233 | 5212 | 5726420 | 5727232 | - | upstream | -78 | 78 | NearestLocation | PSPPH_5052 |
| chromosome | 5727984 | 5728470 | 487 | * | rhpR_rhpS-Mutant_KB_R1_peak_234 | 5212 | 5726420 | 5727232 | - | upstream | -752 | 752 | NearestLocation | PSPPH_5052 |
| chromosome | 5748278 | 5748637 | 360 | * | rhpR_rhpS-Mutant_KB_R1_peak_235 | 5229 | 5748637 | 5750496 | + | overlapStart | -359 | 0 | NearestLocation | PSPPH_5069 |
| chromosome | 5750970 | 5751063 | 94 | * | rhpR_rhpS-Mutant_KB_R1_peak_236 | 5231 | 5751110 | 5751574 | + | upstream | -140 | 47 | NearestLocation | PSPPH_5071 |
| chromosome | 5764621 | 5764746 | 126 | * | rhpR_rhpS-Mutant_KB_R1_peak_237 | 5243 | 5764856 | 5765668 | + | upstream | -235 | 110 | NearestLocation | PSPPH_5082 |
| chromosome | 5903121 | 5903251 | 131 | * | rhpR_rhpS-Mutant_KB_R1_peak_238 | 5368 | 5903367 | 5904626 | + | upstream | -246 | 116 | NearestLocation | PSPPH_5202 |
| chromosome | 5922375 | 5922478 | 104 | * | rhpR_rhpS-Mutant_KB_R1_peak_239 | 5384 | 5920516 | 5922408 | - | overlapStart | 33 | 33 | NearestLocation | PSPPH_5218 |

**D.** **ChIP-seq result for RhpR binding sites in *Pseudomonas savastanoi* pv. *phaseolicola* 1448A *rhpS* mutant** **in MM**

| seqnames | start | end | width | strand | peak | feature | start_position | end_position | feature_strand | insideFeature | distancetoFeature | shortestDistance | fromOverlappingOrNearest | id |
| --- | --- | --- | --- | --- | --- | --- | --- | --- | --- | --- | --- | --- | --- | --- |
| chromosome | 4023 | 4204 | 182 | * | rhpR_rhpS-Mutant_MM_R1_peak_1 | 4 | 4046 | 6463 | + | overlapStart | -23 | 23 | NearestLocation | PSPPH_0004 |
| chromosome | 109472 | 109565 | 94 | * | rhpR_rhpS-Mutant_MM_R1_peak_2 | 101 | 109533 | 109609 | + | overlapStart | -61 | 32 | NearestLocation | PSPPH_0098 |
| chromosome | 111854 | 112109 | 256 | * | rhpR_rhpS-Mutant_MM_R1_peak_3 | 104 | 112706 | 113614 | + | upstream | -852 | 597 | NearestLocation | PSPPH_0101 |
| chromosome | 135127 | 135340 | 214 | * | rhpR_rhpS-Mutant_MM_R1_peak_4 | 120 | 134132 | 136063 | - | inside | 936 | 723 | NearestLocation | PSPPH_0117 |
| chromosome | 204188 | 204482 | 295 | * | rhpR_rhpS-Mutant_MM_R1_peak_5 | 178 | 198685 | 204564 | - | inside | 376 | 82 | NearestLocation | PSPPH_0171 |
| chromosome | 207097 | 207205 | 109 | * | rhpR_rhpS-Mutant_MM_R1_peak_6 | 182 | 207138 | 207986 | + | overlapStart | -41 | 41 | NearestLocation | PSPPH_0173 |
| chromosome | 292687 | 292907 | 221 | * | rhpR_rhpS-Mutant_MM_R1_peak_7 | 263 | 292304 | 294091 | + | inside | 383 | 383 | NearestLocation | PSPPH_0253 |
| chromosome | 317626 | 317821 | 196 | * | rhpR_rhpS-Mutant_MM_R1_peak_8 | 285 | 317114 | 317779 | - | overlapStart | 153 | 42 | NearestLocation | PSPPH_0275 |
| chromosome | 317979 | 318226 | 248 | * | rhpR_rhpS-Mutant_MM_R1_peak_9 | 285 | 317114 | 317779 | - | upstream | -200 | 200 | NearestLocation | PSPPH_0275 |
| chromosome | 320810 | 320917 | 108 | * | rhpR_rhpS-Mutant_MM_R1_peak_10 | 288 | 320118 | 320780 | - | upstream | -30 | 30 | NearestLocation | PSPPH_0278 |
| chromosome | 338961 | 339042 | 82 | * | rhpR_rhpS-Mutant_MM_R1_peak_11 | 305 | 338360 | 339097 | - | inside | 136 | 55 | NearestLocation | PSPPH_0295 |
| chromosome | 341484 | 341609 | 126 | * | rhpR_rhpS-Mutant_MM_R1_peak_12 | 307 | 339414 | 341467 | - | upstream | -17 | 17 | NearestLocation | PSPPH_0297 |
| chromosome | 341484 | 341609 | 126 | * | rhpR_rhpS-Mutant_MM_R1_peak_12 | 308 | 339414 | 341467 | - | upstream | -17 | 17 | NearestLocation | PSPPH_0297 |
| chromosome | 364509 | 364908 | 400 | * | rhpR_rhpS-Mutant_MM_R1_peak_13 | 332 | 364889 | 365785 | + | overlapStart | -380 | 19 | NearestLocation | PSPPH_0320 |
| chromosome | 630616 | 630781 | 166 | * | rhpR_rhpS-Mutant_MM_R1_peak_14 | 549 | 630951 | 632069 | + | upstream | -335 | 170 | NearestLocation | PSPPH_0536 |
| chromosome | 718546 | 718677 | 132 | * | rhpR_rhpS-Mutant_MM_R1_peak_15 | 622 | 718866 | 719993 | + | upstream | -320 | 189 | NearestLocation | PSPPH_0609 |
| chromosome | 732409 | 732776 | 368 | * | rhpR_rhpS-Mutant_MM_R1_peak_16 | 631 | 731881 | 732564 | - | overlapStart | 155 | 155 | NearestLocation | PSPPH_0618 |
| chromosome | 732409 | 732776 | 368 | * | rhpR_rhpS-Mutant_MM_R1_peak_16 | 632 | 731881 | 732564 | - | overlapStart | 155 | 155 | NearestLocation | PSPPH_0618 |
| chromosome | 736808 | 737000 | 193 | * | rhpR_rhpS-Mutant_MM_R1_peak_17 | 634 | 734688 | 736643 | - | upstream | -165 | 165 | NearestLocation | PSPPH_0620 |
| chromosome | 770774 | 771038 | 265 | * | rhpR_rhpS-Mutant_MM_R1_peak_18 | 664 | 768944 | 770866 | - | overlapStart | 92 | 92 | NearestLocation | PSPPH_0650 |
| chromosome | 771239 | 771351 | 113 | * | rhpR_rhpS-Mutant_MM_R1_peak_19 | 665 | 770972 | 771238 | - | upstream | -1 | 1 | NearestLocation | PSPPH_0651 |
| chromosome | 864057 | 864317 | 261 | * | rhpR_rhpS-Mutant_MM_R1_peak_20 | 753 | 863034 | 864626 | - | inside | 569 | 309 | NearestLocation | PSPPH_0737 |
| chromosome | 867640 | 867717 | 78 | * | rhpR_rhpS-Mutant_MM_R1_peak_21 | 758 | 868016 | 870580 | + | upstream | -376 | 299 | NearestLocation | PSPPH_0742 |
| chromosome | 867977 | 868125 | 149 | * | rhpR_rhpS-Mutant_MM_R1_peak_22 | 758 | 868016 | 870580 | + | overlapStart | -39 | 39 | NearestLocation | PSPPH_0742 |
| chromosome | 875206 | 875311 | 106 | * | rhpR_rhpS-Mutant_MM_R1_peak_23 | 765 | 873205 | 874737 | - | upstream | -469 | 469 | NearestLocation | PSPPH_0749 |
| chromosome | 882120 | 882431 | 312 | * | rhpR_rhpS-Mutant_MM_R1_peak_24 | 772 | 882507 | 883145 | + | upstream | -387 | 76 | NearestLocation | PSPPH_0755 |
| chromosome | 883579 | 883781 | 203 | * | rhpR_rhpS-Mutant_MM_R1_peak_25 | 773 | 884445 | 887003 | + | upstream | -866 | 664 | NearestLocation | PSPPH_0756 |
| chromosome | 897375 | 897495 | 121 | * | rhpR_rhpS-Mutant_MM_R1_peak_26 | 784 | 896731 | 896976 | - | upstream | -399 | 399 | NearestLocation | PSPPH_0766 |
| chromosome | 897375 | 897495 | 121 | * | rhpR_rhpS-Mutant_MM_R1_peak_26 | 785 | 896731 | 896976 | - | upstream | -399 | 399 | NearestLocation | PSPPH_0766 |
| chromosome | 898983 | 899073 | 91 | * | rhpR_rhpS-Mutant_MM_R1_peak_27 | 786 | 897416 | 898963 | - | upstream | -20 | 20 | NearestLocation | PSPPH_0767 |
| chromosome | 902824 | 902935 | 112 | * | rhpR_rhpS-Mutant_MM_R1_peak_28 | 789 | 901688 | 903976 | + | inside | 1136 | 1041 | NearestLocation | PSPPH_0770 |
| chromosome | 905463 | 905546 | 84 | * | rhpR_rhpS-Mutant_MM_R1_peak_29 | 790 | 904056 | 904868 | - | upstream | -595 | 595 | NearestLocation | PSPPH_0771 |
| chromosome | 910531 | 910745 | 215 | * | rhpR_rhpS-Mutant_MM_R1_peak_30 | 795 | 910262 | 910975 | - | inside | 444 | 230 | NearestLocation | PSPPH_0775 |
| chromosome | 935603 | 935745 | 143 | * | rhpR_rhpS-Mutant_MM_R1_peak_31 | 814 | 935833 | 936333 | + | upstream | -230 | 88 | NearestLocation | PSPPH_0791 |
| chromosome | 957921 | 958283 | 363 | * | rhpR_rhpS-Mutant_MM_R1_peak_32 | 836 | 958236 | 960398 | + | overlapStart | -315 | 47 | NearestLocation | PSPPH_0813 |
| chromosome | 976342 | 976422 | 81 | * | rhpR_rhpS-Mutant_MM_R1_peak_33 | 839 | 977780 | 977977 | - | downstream | 1635 | 1358 | NearestLocation | PSPPH_0816 |
| chromosome | 982722 | 982993 | 272 | * | rhpR_rhpS-Mutant_MM_R1_peak_34 | 844 | 982619 | 983014 | + | inside | 103 | 21 | NearestLocation | PSPPH_0821 |
| chromosome | 1024700 | 1024917 | 218 | * | rhpR_rhpS-Mutant_MM_R1_peak_35 | 880 | 1023074 | 1024549 | - | upstream | -151 | 151 | NearestLocation | PSPPH_0856 |
| chromosome | 1125855 | 1126060 | 206 | * | rhpR_rhpS-Mutant_MM_R1_peak_36 | 975 | 1125905 | 1125981 | - | includeFeature | 126 | 50 | NearestLocation | PSPPH_0948 |
| chromosome | 1127359 | 1127505 | 147 | * | rhpR_rhpS-Mutant_MM_R1_peak_37 | 977 | 1127559 | 1129616 | + | upstream | -200 | 54 | NearestLocation | PSPPH_0950 |
| chromosome | 1135396 | 1135537 | 142 | * | rhpR_rhpS-Mutant_MM_R1_peak_38 | 983 | 1135374 | 1136954 | + | inside | 22 | 22 | NearestLocation | PSPPH_0956 |
| chromosome | 1140009 | 1140221 | 213 | * | rhpR_rhpS-Mutant_MM_R1_peak_39 | 985 | 1139722 | 1140804 | + | inside | 287 | 287 | NearestLocation | PSPPH_0958 |
| chromosome | 1148292 | 1148522 | 231 | * | rhpR_rhpS-Mutant_MM_R1_peak_40 | 992 | 1147760 | 1149175 | - | inside | 883 | 532 | NearestLocation | PSPPH_0965 |
| chromosome | 1149591 | 1149735 | 145 | * | rhpR_rhpS-Mutant_MM_R1_peak_41 | 993 | 1149304 | 1149723 | + | overlapEnd | 287 | 12 | NearestLocation | PSPPH_0966 |
| chromosome | 1153622 | 1153751 | 130 | * | rhpR_rhpS-Mutant_MM_R1_peak_42 | 996 | 1152260 | 1153360 | - | upstream | -262 | 262 | NearestLocation | PSPPH_0969 |
| chromosome | 1194159 | 1194427 | 269 | * | rhpR_rhpS-Mutant_MM_R1_peak_43 | 1028 | 1194579 | 1195097 | + | upstream | -420 | 152 | NearestLocation | PSPPH_1001 |
| chromosome | 1222994 | 1223386 | 393 | * | rhpR_rhpS-Mutant_MM_R1_peak_44 | 1062 | 1223313 | 1224131 | + | overlapStart | -319 | 73 | NearestLocation | PSPPH_1035 |
| chromosome | 1232377 | 1232585 | 209 | * | rhpR_rhpS-Mutant_MM_R1_peak_45 | 1069 | 1232666 | 1235402 | + | upstream | -289 | 81 | NearestLocation | PSPPH_1042 |
| chromosome | 1232377 | 1232585 | 209 | * | rhpR_rhpS-Mutant_MM_R1_peak_45 | 1070 | 1232666 | 1235402 | + | upstream | -289 | 81 | NearestLocation | PSPPH_1042 |
| chromosome | 1277210 | 1277429 | 220 | * | rhpR_rhpS-Mutant_MM_R1_peak_46 | 1112 | 1277491 | 1277757 | - | downstream | 547 | 62 | NearestLocation | PSPPH_1084 |
| chromosome | 1279049 | 1279292 | 244 | * | rhpR_rhpS-Mutant_MM_R1_peak_47 | 1114 | 1278735 | 1279067 | - | overlapStart | 18 | 18 | NearestLocation | PSPPH_1086 |
| chromosome | 1318943 | 1319018 | 76 | * | rhpR_rhpS-Mutant_MM_R1_peak_48 | 1148 | 1317259 | 1318575 | - | upstream | -368 | 368 | NearestLocation | PSPPH_1118 |
| chromosome | 1319319 | 1319477 | 159 | * | rhpR_rhpS-Mutant_MM_R1_peak_49 | 1149 | 1319388 | 1320167 | - | overlapEnd | 848 | 69 | NearestLocation | PSPPH_1119 |
| chromosome | 1320216 | 1320422 | 207 | * | rhpR_rhpS-Mutant_MM_R1_peak_50 | 1150 | 1320350 | 1321549 | + | overlapStart | -134 | 72 | NearestLocation | PSPPH_1120 |
| chromosome | 1387085 | 1387563 | 479 | * | rhpR_rhpS-Mutant_MM_R1_peak_51 | 1212 | 1387352 | 1388638 | + | overlapStart | -267 | 211 | NearestLocation | PSPPH_1181 |
| chromosome | 1484267 | 1484858 | 592 | * | rhpR_rhpS-Mutant_MM_R1_peak_52 | 1309 | 1485638 | 1486558 | + | upstream | -1371 | 780 | NearestLocation | PSPPH_1270 |
| chromosome | 1594547 | 1594712 | 166 | * | rhpR_rhpS-Mutant_MM_R1_peak_53 | 1414 | 1595303 | 1595491 | - | downstream | 944 | 591 | NearestLocation | PSPPH_1373 |
| chromosome | 1738418 | 1738854 | 437 | * | rhpR_rhpS-Mutant_MM_R1_peak_54 | 1540 | 1736957 | 1738255 | - | upstream | -163 | 163 | NearestLocation | PSPPH_1496 |
| chromosome | 1772096 | 1772228 | 133 | * | rhpR_rhpS-Mutant_MM_R1_peak_55 | 1567 | 1772229 | 1773839 | + | upstream | -133 | 1 | NearestLocation | PSPPH_1523 |
| chromosome | 1952539 | 1952723 | 185 | * | rhpR_rhpS-Mutant_MM_R1_peak_56 | 1740 | 1952561 | 1952637 | + | includeFeature | -22 | 22 | NearestLocation | PSPPH_1688 |
| chromosome | 1956987 | 1957154 | 168 | * | rhpR_rhpS-Mutant_MM_R1_peak_57 | 1746 | 1957205 | 1958197 | - | downstream | 1210 | 51 | NearestLocation | PSPPH_1694 |
| chromosome | 1965519 | 1965695 | 177 | * | rhpR_rhpS-Mutant_MM_R1_peak_58 | 1752 | 1964447 | 1966843 | + | inside | 1072 | 1072 | NearestLocation | PSPPH_1700 |
| chromosome | 1966427 | 1966585 | 159 | * | rhpR_rhpS-Mutant_MM_R1_peak_59 | 1753 | 1966990 | 1967262 | + | upstream | -563 | 405 | NearestLocation | PSPPH_1701 |
| chromosome | 1999694 | 1999879 | 186 | * | rhpR_rhpS-Mutant_MM_R1_peak_60 | 1777 | 1998652 | 1999770 | - | overlapStart | 76 | 76 | NearestLocation | PSPPH_1725 |
| chromosome | 2084953 | 2085101 | 149 | * | rhpR_rhpS-Mutant_MM_R1_peak_61 | 1835 | 2084780 | 2085943 | + | inside | 173 | 173 | NearestLocation | PSPPH_1780 |
| chromosome | 2109566 | 2109924 | 359 | * | rhpR_rhpS-Mutant_MM_R1_peak_62 | 1861 | 2109063 | 2109713 | - | overlapStart | 147 | 147 | NearestLocation | PSPPH_1805 |
| chromosome | 2129442 | 2129534 | 93 | * | rhpR_rhpS-Mutant_MM_R1_peak_63 | 1885 | 2129672 | 2130631 | + | upstream | -230 | 138 | NearestLocation | PSPPH_1829 |
| chromosome | 2137883 | 2138022 | 140 | * | rhpR_rhpS-Mutant_MM_R1_peak_64 | 1897 | 2137193 | 2137723 | - | upstream | -160 | 160 | NearestLocation | PSPPH_1840 |
| chromosome | 2190313 | 2190535 | 223 | * | rhpR_rhpS-Mutant_MM_R1_peak_65 | 1948 | 2190515 | 2190874 | + | overlapStart | -202 | 20 | NearestLocation | PSPPH_1888 |
| chromosome | 2318401 | 2318490 | 90 | * | rhpR_rhpS-Mutant_MM_R1_peak_66 | 2030 | 2318480 | 2319223 | + | overlapStart | -79 | 10 | NearestLocation | PSPPH_1970 |
| chromosome | 2350441 | 2350618 | 178 | * | rhpR_rhpS-Mutant_MM_R1_peak_67 | 2064 | 2350640 | 2351398 | - | downstream | 957 | 22 | NearestLocation | PSPPH_2002 |
| chromosome | 2351059 | 2351592 | 534 | * | rhpR_rhpS-Mutant_MM_R1_peak_68 | 2064 | 2350640 | 2351398 | - | overlapStart | 339 | 194 | NearestLocation | PSPPH_2002 |
| chromosome | 2352278 | 2352368 | 91 | * | rhpR_rhpS-Mutant_MM_R1_peak_69 | 2065 | 2351514 | 2352539 | - | inside | 261 | 171 | NearestLocation | PSPPH_2003 |
| chromosome | 2352550 | 2353829 | 1280 | * | rhpR_rhpS-Mutant_MM_R1_peak_70 | 2066 | 2352536 | 2353285 | - | overlapStart | 735 | 14 | NearestLocation | PSPPH_2004 |
| chromosome | 2366260 | 2366335 | 76 | * | rhpR_rhpS-Mutant_MM_R1_peak_71 | 2076 | 2365654 | 2366211 | - | upstream | -49 | 49 | NearestLocation | PSPPH_2013 |
| chromosome | 2434217 | 2434385 | 169 | * | rhpR_rhpS-Mutant_MM_R1_peak_72 | 2140 | 2434443 | 2435018 | - | downstream | 801 | 58 | NearestLocation | PSPPH_2075 |
| chromosome | 2437668 | 2437788 | 121 | * | rhpR_rhpS-Mutant_MM_R1_peak_73 | 2143 | 2437666 | 2437992 | - | inside | 324 | 2 | NearestLocation | PSPPH_2078 |
| chromosome | 2437886 | 2438049 | 164 | * | rhpR_rhpS-Mutant_MM_R1_peak_74 | 2143 | 2437666 | 2437992 | - | overlapStart | 106 | 57 | NearestLocation | PSPPH_2078 |
| chromosome | 2440647 | 2440846 | 200 | * | rhpR_rhpS-Mutant_MM_R1_peak_75 | 2145 | 2440865 | 2443255 | + | upstream | -218 | 19 | NearestLocation | PSPPH_2080 |
| chromosome | 2478818 | 2479064 | 247 | * | rhpR_rhpS-Mutant_MM_R1_peak_76 | 2184 | 2478966 | 2479223 | + | overlapStart | -148 | 98 | NearestLocation | PSPPH_2119 |
| chromosome | 2489286 | 2489508 | 223 | * | rhpR_rhpS-Mutant_MM_R1_peak_77 | 2197 | 2489070 | 2489291 | - | overlapStart | 5 | 5 | NearestLocation | PSPPH_2132 |
| chromosome | 2515222 | 2515353 | 132 | * | rhpR_rhpS-Mutant_MM_R1_peak_78 | 2225 | 2514967 | 2516157 | + | inside | 255 | 255 | NearestLocation | PSPPH_2159 |
| chromosome | 2540935 | 2541346 | 412 | * | rhpR_rhpS-Mutant_MM_R1_peak_79 | 2256 | 2541290 | 2542897 | + | overlapStart | -355 | 56 | NearestLocation | PSPPH_2186 |
| chromosome | 2550517 | 2550671 | 155 | * | rhpR_rhpS-Mutant_MM_R1_peak_80 | 2264 | 2550022 | 2550513 | - | upstream | -4 | 4 | NearestLocation | PSPPH_2194 |
| chromosome | 2554341 | 2554453 | 113 | * | rhpR_rhpS-Mutant_MM_R1_peak_81 | 2267 | 2553044 | 2554293 | - | upstream | -48 | 48 | NearestLocation | PSPPH_2197 |
| chromosome | 2554341 | 2554453 | 113 | * | rhpR_rhpS-Mutant_MM_R1_peak_81 | 2268 | 2553044 | 2554293 | - | upstream | -48 | 48 | NearestLocation | PSPPH_2197 |
| chromosome | 2644908 | 2645093 | 186 | * | rhpR_rhpS-Mutant_MM_R1_peak_82 | 2353 | 2645116 | 2646675 | + | upstream | -208 | 23 | NearestLocation | PSPPH_2279 |
| chromosome | 2884092 | 2884201 | 110 | * | rhpR_rhpS-Mutant_MM_R1_peak_83 | 2579 | 2883466 | 2884311 | - | inside | 219 | 110 | NearestLocation | PSPPH_2496 |
| chromosome | 2981673 | 2981921 | 249 | * | rhpR_rhpS-Mutant_MM_R1_peak_84 | 2675 | 2981819 | 2984701 | + | overlapStart | -146 | 102 | NearestLocation | PSPPH_2590 |
| chromosome | 3018358 | 3018524 | 167 | * | rhpR_rhpS-Mutant_MM_R1_peak_85 | 2704 | 3016634 | 3018292 | - | upstream | -66 | 66 | NearestLocation | PSPPH_2618 |
| chromosome | 3018920 | 3019047 | 128 | * | rhpR_rhpS-Mutant_MM_R1_peak_86 | 2705 | 3018875 | 3019948 | + | inside | 45 | 45 | NearestLocation | PSPPH_2619 |
| chromosome | 3034197 | 3034363 | 167 | * | rhpR_rhpS-Mutant_MM_R1_peak_87 | 2715 | 3032695 | 3033858 | - | upstream | -339 | 339 | NearestLocation | PSPPH_2628 |
| chromosome | 3034197 | 3034363 | 167 | * | rhpR_rhpS-Mutant_MM_R1_peak_87 | 2716 | 3032695 | 3033858 | - | upstream | -339 | 339 | NearestLocation | PSPPH_2628 |
| chromosome | 3034478 | 3034598 | 121 | * | rhpR_rhpS-Mutant_MM_R1_peak_88 | 2715 | 3032695 | 3033858 | - | upstream | -620 | 620 | NearestLocation | PSPPH_2628 |
| chromosome | 3034478 | 3034598 | 121 | * | rhpR_rhpS-Mutant_MM_R1_peak_88 | 2716 | 3032695 | 3033858 | - | upstream | -620 | 620 | NearestLocation | PSPPH_2628 |
| chromosome | 3077361 | 3077545 | 185 | * | rhpR_rhpS-Mutant_MM_R1_peak_89 | 2742 | 3076506 | 3077354 | - | upstream | -7 | 7 | NearestLocation | PSPPH_2653 |
| chromosome | 3178730 | 3179005 | 276 | * | rhpR_rhpS-Mutant_MM_R1_peak_90 | 2837 | 3179070 | 3179609 | + | upstream | -340 | 65 | NearestLocation | PSPPH_2747 |
| chromosome | 3225835 | 3225983 | 149 | * | rhpR_rhpS-Mutant_MM_R1_peak_91 | 2878 | 3225566 | 3225958 | - | overlapStart | 123 | 25 | NearestLocation | PSPPH_2787 |
| chromosome | 3229738 | 3229906 | 169 | * | rhpR_rhpS-Mutant_MM_R1_peak_92 | 2881 | 3229542 | 3229829 | - | overlapStart | 91 | 77 | NearestLocation | PSPPH_2790 |
| chromosome | 3230336 | 3230445 | 110 | * | rhpR_rhpS-Mutant_MM_R1_peak_93 | 2881 | 3229542 | 3229829 | - | upstream | -507 | 507 | NearestLocation | PSPPH_2790 |
| chromosome | 3231114 | 3231233 | 120 | * | rhpR_rhpS-Mutant_MM_R1_peak_94 | 2882 | 3230334 | 3231464 | - | inside | 350 | 231 | NearestLocation | PSPPH_2791 |
| chromosome | 3529192 | 3529289 | 98 | * | rhpR_rhpS-Mutant_MM_R1_peak_95 | 3138 | 3527024 | 3529150 | - | upstream | -42 | 42 | NearestLocation | PSPPH_3037 |
| chromosome | 3533576 | 3533662 | 87 | * | rhpR_rhpS-Mutant_MM_R1_peak_96 | 3143 | 3533706 | 3534569 | + | upstream | -130 | 44 | NearestLocation | PSPPH_3042 |
| chromosome | 3552803 | 3553026 | 224 | * | rhpR_rhpS-Mutant_MM_R1_peak_97 | 3159 | 3551788 | 3552885 | - | overlapStart | 82 | 82 | NearestLocation | PSPPH_3057 |
| chromosome | 3575298 | 3575654 | 357 | * | rhpR_rhpS-Mutant_MM_R1_peak_98 | 3181 | 3573928 | 3575358 | - | overlapStart | 60 | 60 | NearestLocation | PSPPH_3076 |
| chromosome | 3575298 | 3575654 | 357 | * | rhpR_rhpS-Mutant_MM_R1_peak_98 | 3182 | 3573928 | 3575358 | - | overlapStart | 60 | 60 | NearestLocation | PSPPH_3076 |
| chromosome | 3577416 | 3577497 | 82 | * | rhpR_rhpS-Mutant_MM_R1_peak_99 | 3185 | 3576871 | 3577779 | + | inside | 545 | 282 | NearestLocation | PSPPH_3079 |
| chromosome | 3622741 | 3622833 | 93 | * | rhpR_rhpS-Mutant_MM_R1_peak_100 | 3229 | 3623017 | 3624006 | - | downstream | 1265 | 184 | NearestLocation | PSPPH_3122 |
| chromosome | 3624899 | 3625135 | 237 | * | rhpR_rhpS-Mutant_MM_R1_peak_101 | 3230 | 3624094 | 3625017 | + | overlapEnd | 805 | 118 | NearestLocation | PSPPH_3123 |
| chromosome | 3799019 | 3799102 | 84 | * | rhpR_rhpS-Mutant_MM_R1_peak_102 | 3388 | 3796797 | 3798890 | - | upstream | -129 | 129 | NearestLocation | PSPPH_3276 |
| chromosome | 3836440 | 3836964 | 525 | * | rhpR_rhpS-Mutant_MM_R1_peak_103 | 3428 | 3836826 | 3838463 | + | overlapStart | -386 | 138 | NearestLocation | PSPPH_3315 |
| chromosome | 3857180 | 3857325 | 146 | * | rhpR_rhpS-Mutant_MM_R1_peak_104 | 3445 | 3857400 | 3857924 | + | upstream | -220 | 75 | NearestLocation | PSPPH_3331 |
| chromosome | 3895972 | 3896164 | 193 | * | rhpR_rhpS-Mutant_MM_R1_peak_105 | 3480 | 3893910 | 3896039 | - | overlapStart | 67 | 67 | NearestLocation | PSPPH_3366 |
| chromosome | 3900462 | 3900591 | 130 | * | rhpR_rhpS-Mutant_MM_R1_peak_106 | 3486 | 3900030 | 3900488 | - | overlapStart | 26 | 26 | NearestLocation | PSPPH_3372 |
| chromosome | 3902046 | 3902178 | 133 | * | rhpR_rhpS-Mutant_MM_R1_peak_107 | 3488 | 3901528 | 3902028 | - | upstream | -18 | 18 | NearestLocation | PSPPH_3374 |
| chromosome | 3937382 | 3937545 | 164 | * | rhpR_rhpS-Mutant_MM_R1_peak_108 | 3516 | 3936650 | 3937390 | - | overlapStart | 8 | 8 | NearestLocation | PSPPH_3402 |
| chromosome | 3946831 | 3947014 | 184 | * | rhpR_rhpS-Mutant_MM_R1_peak_109 | 3527 | 3945912 | 3946838 | - | overlapStart | 7 | 7 | NearestLocation | PSPPH_3413 |
| chromosome | 4012880 | 4013020 | 141 | * | rhpR_rhpS-Mutant_MM_R1_peak_110 | 3591 | 4013029 | 4015101 | + | upstream | -149 | 9 | NearestLocation | PSPPH_3473 |
| chromosome | 4036296 | 4036426 | 131 | * | rhpR_rhpS-Mutant_MM_R1_peak_111 | 3612 | 4034772 | 4035991 | - | upstream | -305 | 305 | NearestLocation | PSPPH_3494 |
| chromosome | 4036296 | 4036426 | 131 | * | rhpR_rhpS-Mutant_MM_R1_peak_111 | 3613 | 4034772 | 4035991 | - | upstream | -305 | 305 | NearestLocation | PSPPH_3494 |
| chromosome | 4102134 | 4102248 | 115 | * | rhpR_rhpS-Mutant_MM_R1_peak_112 | 3680 | 4097194 | 4102053 | - | upstream | -81 | 81 | NearestLocation | PSPPH_3560 |
| chromosome | 4197035 | 4197354 | 320 | * | rhpR_rhpS-Mutant_MM_R1_peak_113 | 3776 | 4197240 | 4197809 | + | overlapStart | -205 | 114 | NearestLocation | PSPPH_3654 |
| chromosome | 4222310 | 4222448 | 139 | * | rhpR_rhpS-Mutant_MM_R1_peak_114 | 3796 | 4221051 | 4221836 | - | upstream | -474 | 474 | NearestLocation | PSPPH_3674 |
| chromosome | 4236154 | 4236387 | 234 | * | rhpR_rhpS-Mutant_MM_R1_peak_115 | 3810 | 4236460 | 4236762 | + | upstream | -306 | 73 | NearestLocation | PSPPH_3688 |
| chromosome | 4285404 | 4285590 | 187 | * | rhpR_rhpS-Mutant_MM_R1_peak_116 | 3859 | 4284455 | 4285192 | - | upstream | -212 | 212 | NearestLocation | PSPPH_3737 |
| chromosome | 4311266 | 4311370 | 105 | * | rhpR_rhpS-Mutant_MM_R1_peak_117 | 3880 | 4310221 | 4310874 | - | upstream | -392 | 392 | NearestLocation | PSPPH_3758 |
| chromosome | 4311480 | 4311669 | 190 | * | rhpR_rhpS-Mutant_MM_R1_peak_118 | 3881 | 4310952 | 4311974 | - | inside | 494 | 305 | NearestLocation | PSPPH_3759 |
| chromosome | 4349856 | 4349942 | 87 | * | rhpR_rhpS-Mutant_MM_R1_peak_119 | 3920 | 4349926 | 4350852 | + | overlapStart | -70 | 16 | NearestLocation | PSPPH_3798 |
| chromosome | 4402109 | 4402433 | 325 | * | rhpR_rhpS-Mutant_MM_R1_peak_120 | 3967 | 4402378 | 4402725 | + | overlapStart | -269 | 55 | NearestLocation | PSPPH_3845 |
| chromosome | 4477532 | 4477696 | 165 | * | rhpR_rhpS-Mutant_MM_R1_peak_121 | 4044 | 4477670 | 4478281 | + | overlapStart | -138 | 26 | NearestLocation | PSPPH_3920 |
| chromosome | 4514379 | 4514504 | 126 | * | rhpR_rhpS-Mutant_MM_R1_peak_122 | 4075 | 4512549 | 4514336 | - | upstream | -43 | 43 | NearestLocation | PSPPH_3951 |
| chromosome | 4552038 | 4552191 | 154 | * | rhpR_rhpS-Mutant_MM_R1_peak_123 | 4112 | 4551605 | 4552324 | - | inside | 286 | 133 | NearestLocation | PSPPH_3986 |
| chromosome | 4561102 | 4561217 | 116 | * | rhpR_rhpS-Mutant_MM_R1_peak_124 | 4122 | 4559857 | 4561077 | - | upstream | -25 | 25 | NearestLocation | PSPPH_3995 |
| chromosome | 4600612 | 4600814 | 203 | * | rhpR_rhpS-Mutant_MM_R1_peak_125 | 4154 | 4600070 | 4600450 | + | downstream | 542 | 162 | NearestLocation | PSPPH_4025 |
| chromosome | 4612333 | 4612411 | 79 | * | rhpR_rhpS-Mutant_MM_R1_peak_126 | 4168 | 4611823 | 4612182 | - | upstream | -151 | 151 | NearestLocation | PSPPH_4037 |
| chromosome | 4617415 | 4617571 | 157 | * | rhpR_rhpS-Mutant_MM_R1_peak_127 | 4173 | 4617999 | 4619348 | - | downstream | 1933 | 428 | NearestLocation | PSPPH_4042 |
| chromosome | 4806422 | 4806498 | 77 | * | rhpR_rhpS-Mutant_MM_R1_peak_128 | 4355 | 4806712 | 4808529 | + | upstream | -290 | 214 | NearestLocation | PSPPH_4218 |
| chromosome | 4838249 | 4838634 | 386 | * | rhpR_rhpS-Mutant_MM_R1_peak_129 | 4379 | 4838479 | 4839108 | + | overlapStart | -230 | 155 | NearestLocation | PSPPH_4241 |
| chromosome | 4843994 | 4844361 | 368 | * | rhpR_rhpS-Mutant_MM_R1_peak_130 | 4385 | 4844348 | 4846210 | + | overlapStart | -354 | 13 | NearestLocation | PSPPH_4247 |
| chromosome | 4848241 | 4848319 | 79 | * | rhpR_rhpS-Mutant_MM_R1_peak_131 | 4387 | 4848143 | 4848382 | - | inside | 141 | 63 | NearestLocation | PSPPH_4249 |
| chromosome | 4931269 | 4931363 | 95 | * | rhpR_rhpS-Mutant_MM_R1_peak_132 | 4455 | 4930056 | 4931276 | - | overlapStart | 7 | 7 | NearestLocation | PSPPH_4317 |
| chromosome | 4985788 | 4985880 | 93 | * | rhpR_rhpS-Mutant_MM_R1_peak_133 | 4503 | 4985442 | 4985747 | + | downstream | 346 | 41 | NearestLocation | PSPPH_4363 |
| chromosome | 5043779 | 5043859 | 81 | * | rhpR_rhpS-Mutant_MM_R1_peak_134 | 4558 | 5043760 | 5044233 | + | inside | 19 | 19 | NearestLocation | PSPPH_4418 |
| chromosome | 5087815 | 5088024 | 210 | * | rhpR_rhpS-Mutant_MM_R1_peak_135 | 4593 | 5087883 | 5088518 | - | overlapEnd | 703 | 68 | NearestLocation | PSPPH_4453 |
| chromosome | 5173259 | 5173364 | 106 | * | rhpR_rhpS-Mutant_MM_R1_peak_136 | 4679 | 5173332 | 5175449 | + | overlapStart | -73 | 32 | NearestLocation | PSPPH_4539 |
| chromosome | 5177203 | 5177424 | 222 | * | rhpR_rhpS-Mutant_MM_R1_peak_137 | 4683 | 5177776 | 5178747 | + | upstream | -573 | 352 | NearestLocation | PSPPH_4542 |
| chromosome | 5180028 | 5180151 | 124 | * | rhpR_rhpS-Mutant_MM_R1_peak_138 | 4685 | 5179499 | 5180248 | - | inside | 220 | 97 | NearestLocation | PSPPH_4544 |
| chromosome | 5206425 | 5206587 | 163 | * | rhpR_rhpS-Mutant_MM_R1_peak_139 | 4702 | 5204697 | 5206401 | - | upstream | -24 | 24 | NearestLocation | PSPPH_4561 |
| chromosome | 5206425 | 5206587 | 163 | * | rhpR_rhpS-Mutant_MM_R1_peak_139 | 4703 | 5204697 | 5206401 | - | upstream | -24 | 24 | NearestLocation | PSPPH_4561 |
| chromosome | 5208535 | 5208629 | 95 | * | rhpR_rhpS-Mutant_MM_R1_peak_140 | 4705 | 5208582 | 5211416 | + | overlapStart | -47 | 47 | NearestLocation | PSPPH_4563 |
| chromosome | 5216586 | 5216671 | 86 | * | rhpR_rhpS-Mutant_MM_R1_peak_141 | 4712 | 5216253 | 5216609 | - | overlapStart | 23 | 23 | NearestLocation | PSPPH_4570 |
| chromosome | 5231939 | 5232065 | 127 | * | rhpR_rhpS-Mutant_MM_R1_peak_142 | 4739 | 5231556 | 5231927 | - | upstream | -12 | 12 | NearestLocation | PSPPH_4597 |
| chromosome | 5317876 | 5318038 | 163 | * | rhpR_rhpS-Mutant_MM_R1_peak_143 | 4824 | 5317528 | 5318289 | - | inside | 413 | 251 | NearestLocation | PSPPH_4682 |
| chromosome | 5341060 | 5341224 | 165 | * | rhpR_rhpS-Mutant_MM_R1_peak_144 | 4847 | 5341405 | 5341479 | + | upstream | -345 | 181 | NearestLocation | PSPPH_4704 |
| chromosome | 5346983 | 5347407 | 425 | * | rhpR_rhpS-Mutant_MM_R1_peak_145 | 4854 | 5346762 | 5347043 | - | overlapStart | 60 | 60 | NearestLocation | PSPPH_4711 |
| chromosome | 5446424 | 5446624 | 201 | * | rhpR_rhpS-Mutant_MM_R1_peak_146 | 4947 | 5446674 | 5449367 | + | upstream | -250 | 50 | NearestLocation | PSPPH_4801 |
| chromosome | 5581762 | 5581838 | 77 | * | rhpR_rhpS-Mutant_MM_R1_peak_147 | 5062 | 5580213 | 5581574 | - | upstream | -188 | 188 | NearestLocation | PSPPH_4907 |
| chromosome | 5646270 | 5646481 | 212 | * | rhpR_rhpS-Mutant_MM_R1_peak_148 | 5135 | 5646120 | 5646566 | + | inside | 150 | 85 | NearestLocation | PSPPH_4977 |
| chromosome | 5663072 | 5663305 | 234 | * | rhpR_rhpS-Mutant_MM_R1_peak_149 | 5152 | 5663238 | 5664533 | + | overlapStart | -166 | 67 | NearestLocation | PSPPH_4994 |
| chromosome | 5692156 | 5692321 | 166 | * | rhpR_rhpS-Mutant_MM_R1_peak_150 | 5181 | 5690781 | 5692085 | - | upstream | -71 | 71 | NearestLocation | PSPPH_5023 |
| chromosome | 5727286 | 5727611 | 326 | * | rhpR_rhpS-Mutant_MM_R1_peak_151 | 5212 | 5726420 | 5727232 | - | upstream | -54 | 54 | NearestLocation | PSPPH_5052 |
| chromosome | 5727978 | 5728514 | 537 | * | rhpR_rhpS-Mutant_MM_R1_peak_152 | 5212 | 5726420 | 5727232 | - | upstream | -746 | 746 | NearestLocation | PSPPH_5052 |
| chromosome | 5748414 | 5748630 | 217 | * | rhpR_rhpS-Mutant_MM_R1_peak_153 | 5229 | 5748637 | 5750496 | + | upstream | -223 | 7 | NearestLocation | PSPPH_5069 |
| chromosome | 5908987 | 5909081 | 95 | * | rhpR_rhpS-Mutant_MM_R1_peak_154 | 5370 | 5908358 | 5909125 | - | inside | 138 | 44 | NearestLocation | PSPPH_5204 |

**E. ChIP-seq result for RhpR^D70A^ binding sites in *Pseudomonas savastanoi* pv. *phaseolicola* 1448A *rhpS* mutant in KB**

| seqnames | start | end | width | strand | peak | feature | start_position | end_position | feature_strand | insideFeature | distancetoFeature | shortestDistance | fromOverlappingOrNearest | id |
| --- | --- | --- | --- | --- | --- | --- | --- | --- | --- | --- | --- | --- | --- | --- |
| chromosome | 12574 | 12705 | 132 | * | rhpR-D70A_rhpS-Mutant_KB_R1_peak_1 | 9 | 11095 | 12453 | - | upstream | -121 | 121 | NearestLocation | PSPPH_0008 |
| chromosome | 109502 | 109584 | 83 | * | rhpR-D70A_rhpS-Mutant_KB_R1_peak_2 | 101 | 109533 | 109609 | + | overlapStart | -31 | 25 | NearestLocation | PSPPH_0098 |
| chromosome | 182708 | 182807 | 100 | * | rhpR-D70A_rhpS-Mutant_KB_R1_peak_3 | 161 | 182883 | 184157 | + | upstream | -175 | 76 | NearestLocation | PSPPH_0155 |
| chromosome | 212800 | 212905 | 106 | * | rhpR-D70A_rhpS-Mutant_KB_R1_peak_4 | 188 | 212966 | 214093 | + | upstream | -166 | 61 | NearestLocation | PSPPH_0179 |
| chromosome | 219628 | 219703 | 76 | * | rhpR-D70A_rhpS-Mutant_KB_R1_peak_5 | 191 | 220514 | 221149 | - | downstream | 1521 | 811 | NearestLocation | PSPPH_0182 |
| chromosome | 229925 | 230014 | 90 | * | rhpR-D70A_rhpS-Mutant_KB_R1_peak_6 | 200 | 228991 | 229914 | - | upstream | -11 | 11 | NearestLocation | PSPPH_0190 |
| chromosome | 236866 | 236986 | 121 | * | rhpR-D70A_rhpS-Mutant_KB_R1_peak_7 | 208 | 236596 | 236937 | - | overlapStart | 71 | 49 | NearestLocation | PSPPH_0198 |
| chromosome | 320864 | 321029 | 166 | * | rhpR-D70A_rhpS-Mutant_KB_R1_peak_8 | 289 | 321012 | 322022 | + | overlapStart | -148 | 17 | NearestLocation | PSPPH_0279 |
| chromosome | 642345 | 642467 | 123 | * | rhpR-D70A_rhpS-Mutant_KB_R1_peak_9 | 561 | 642501 | 643199 | + | upstream | -156 | 34 | NearestLocation | PSPPH_0548 |
| chromosome | 734516 | 734748 | 233 | * | rhpR-D70A_rhpS-Mutant_KB_R1_peak_10 | 633 | 732770 | 734620 | - | overlapStart | 104 | 104 | NearestLocation | PSPPH_0619 |
| chromosome | 744373 | 744514 | 142 | * | rhpR-D70A_rhpS-Mutant_KB_R1_peak_11 | 642 | 743123 | 744394 | - | overlapStart | 21 | 21 | NearestLocation | PSPPH_0628 |
| chromosome | 746541 | 746637 | 97 | * | rhpR-D70A_rhpS-Mutant_KB_R1_peak_12 | 643 | 744516 | 746438 | - | upstream | -103 | 103 | NearestLocation | PSPPH_0629 |
| chromosome | 788450 | 788564 | 115 | * | rhpR-D70A_rhpS-Mutant_KB_R1_peak_13 | 687 | 788757 | 789356 | + | upstream | -307 | 193 | NearestLocation | PSPPH_0672 |
| chromosome | 889037 | 889204 | 168 | * | rhpR-D70A_rhpS-Mutant_KB_R1_peak_14 | 776 | 889391 | 889831 | - | downstream | 794 | 187 | NearestLocation | PSPPH_0759 |
| chromosome | 893034 | 893118 | 85 | * | rhpR-D70A_rhpS-Mutant_KB_R1_peak_15 | 779 | 892074 | 892562 | - | upstream | -472 | 472 | NearestLocation | PSPPH_0762 |
| chromosome | 982831 | 982938 | 108 | * | rhpR-D70A_rhpS-Mutant_KB_R1_peak_16 | 844 | 982619 | 983014 | + | inside | 212 | 76 | NearestLocation | PSPPH_0821 |
| chromosome | 1041960 | 1042073 | 114 | * | rhpR-D70A_rhpS-Mutant_KB_R1_peak_17 | 895 | 1041434 | 1041850 | - | upstream | -110 | 110 | NearestLocation | PSPPH_0871 |
| chromosome | 1125706 | 1125863 | 158 | * | rhpR-D70A_rhpS-Mutant_KB_R1_peak_18 | 975 | 1125905 | 1125981 | - | downstream | 275 | 42 | NearestLocation | PSPPH_0948 |
| chromosome | 1125933 | 1126049 | 117 | * | rhpR-D70A_rhpS-Mutant_KB_R1_peak_19 | 975 | 1125905 | 1125981 | - | overlapStart | 48 | 28 | NearestLocation | PSPPH_0948 |
| chromosome | 1135394 | 1135496 | 103 | * | rhpR-D70A_rhpS-Mutant_KB_R1_peak_20 | 983 | 1135374 | 1136954 | + | inside | 20 | 20 | NearestLocation | PSPPH_0956 |
| chromosome | 1186101 | 1186259 | 159 | * | rhpR-D70A_rhpS-Mutant_KB_R1_peak_21 | 1019 | 1186141 | 1186215 | - | includeFeature | 114 | 40 | NearestLocation | PSPPH_0992 |
| chromosome | 1189577 | 1189738 | 162 | * | rhpR-D70A_rhpS-Mutant_KB_R1_peak_22 | 1023 | 1189698 | 1190975 | + | overlapStart | -121 | 40 | NearestLocation | PSPPH_0996 |
| chromosome | 1318681 | 1318933 | 253 | * | rhpR-D70A_rhpS-Mutant_KB_R1_peak_23 | 1148 | 1317259 | 1318575 | - | upstream | -106 | 106 | NearestLocation | PSPPH_1118 |
| chromosome | 1320287 | 1320365 | 79 | * | rhpR-D70A_rhpS-Mutant_KB_R1_peak_24 | 1150 | 1320350 | 1321549 | + | overlapStart | -63 | 15 | NearestLocation | PSPPH_1120 |
| chromosome | 1321524 | 1321599 | 76 | * | rhpR-D70A_rhpS-Mutant_KB_R1_peak_25 | 1151 | 1321844 | 1323238 | + | upstream | -320 | 245 | NearestLocation | PSPPH_1121 |
| chromosome | 1345935 | 1346095 | 161 | * | rhpR-D70A_rhpS-Mutant_KB_R1_peak_26 | 1176 | 1345074 | 1345496 | - | upstream | -439 | 439 | NearestLocation | PSPPH_1146 |
| chromosome | 1350159 | 1350347 | 189 | * | rhpR-D70A_rhpS-Mutant_KB_R1_peak_27 | 1180 | 1349050 | 1350198 | - | overlapStart | 39 | 39 | NearestLocation | PSPPH_1149 |
| chromosome | 1359345 | 1359469 | 125 | * | rhpR-D70A_rhpS-Mutant_KB_R1_peak_28 | 1188 | 1357817 | 1359421 | - | overlapStart | 76 | 48 | NearestLocation | PSPPH_1157 |
| chromosome | 1362283 | 1362420 | 138 | * | rhpR-D70A_rhpS-Mutant_KB_R1_peak_29 | 1191 | 1361947 | 1362159 | - | upstream | -124 | 124 | NearestLocation | PSPPH_1160 |
| chromosome | 1418087 | 1418181 | 95 | * | rhpR-D70A_rhpS-Mutant_KB_R1_peak_30 | 1239 | 1417026 | 1417922 | - | upstream | -165 | 165 | NearestLocation | PSPPH_1207 |
| chromosome | 1419231 | 1419308 | 78 | * | rhpR-D70A_rhpS-Mutant_KB_R1_peak_31 | 1242 | 1419508 | 1420449 | + | upstream | -277 | 200 | NearestLocation | PSPPH_1210 |
| chromosome | 1708726 | 1708807 | 82 | * | rhpR-D70A_rhpS-Mutant_KB_R1_peak_32 | 1508 | 1708032 | 1708760 | - | overlapStart | 34 | 34 | NearestLocation | PSPPH_1465 |
| chromosome | 1818358 | 1818478 | 121 | * | rhpR-D70A_rhpS-Mutant_KB_R1_peak_33 | 1614 | 1818544 | 1819482 | + | upstream | -186 | 66 | NearestLocation | PSPPH_1567 |
| chromosome | 1904131 | 1904255 | 125 | * | rhpR-D70A_rhpS-Mutant_KB_R1_peak_34 | 1693 | 1903900 | 1904643 | + | inside | 231 | 231 | NearestLocation | PSPPH_1641 |
| chromosome | 1930983 | 1931090 | 108 | * | rhpR-D70A_rhpS-Mutant_KB_R1_peak_35 | 1721 | 1929241 | 1930776 | - | upstream | -207 | 207 | NearestLocation | PSPPH_1669 |
| chromosome | 1952619 | 1952737 | 119 | * | rhpR-D70A_rhpS-Mutant_KB_R1_peak_36 | 1741 | 1952689 | 1952764 | + | overlapStart | -70 | 27 | NearestLocation | PSPPH_1689 |
| chromosome | 2068247 | 2068377 | 131 | * | rhpR-D70A_rhpS-Mutant_KB_R1_peak_37 | 1826 | 2068454 | 2069206 | + | upstream | -207 | 77 | NearestLocation | PSPPH_1771 |
| chromosome | 2109946 | 2110310 | 365 | * | rhpR-D70A_rhpS-Mutant_KB_R1_peak_38 | 1861 | 2109063 | 2109713 | - | upstream | -233 | 233 | NearestLocation | PSPPH_1805 |
| chromosome | 2127259 | 2127390 | 132 | * | rhpR-D70A_rhpS-Mutant_KB_R1_peak_39 | 1883 | 2127447 | 2127788 | + | upstream | -188 | 57 | NearestLocation | PSPPH_1827 |
| chromosome | 2168329 | 2168429 | 101 | * | rhpR-D70A_rhpS-Mutant_KB_R1_peak_40 | 1925 | 2168726 | 2169211 | + | upstream | -397 | 297 | NearestLocation | PSPPH_1868 |
| chromosome | 2350486 | 2350606 | 121 | * | rhpR-D70A_rhpS-Mutant_KB_R1_peak_41 | 2064 | 2350640 | 2351398 | - | downstream | 912 | 34 | NearestLocation | PSPPH_2002 |
| chromosome | 2351388 | 2351513 | 126 | * | rhpR-D70A_rhpS-Mutant_KB_R1_peak_42 | 2064 | 2350640 | 2351398 | - | overlapStart | 10 | 10 | NearestLocation | PSPPH_2002 |
| chromosome | 2352555 | 2353790 | 1236 | * | rhpR-D70A_rhpS-Mutant_KB_R1_peak_43 | 2066 | 2352536 | 2353285 | - | overlapStart | 730 | 19 | NearestLocation | PSPPH_2004 |
| chromosome | 2437901 | 2438027 | 127 | * | rhpR-D70A_rhpS-Mutant_KB_R1_peak_44 | 2143 | 2437666 | 2437992 | - | overlapStart | 91 | 35 | NearestLocation | PSPPH_2078 |
| chromosome | 2497680 | 2497835 | 156 | * | rhpR-D70A_rhpS-Mutant_KB_R1_peak_45 | 2206 | 2497808 | 2498110 | + | overlapStart | -128 | 27 | NearestLocation | PSPPH_2141 |
| chromosome | 2540823 | 2540901 | 79 | * | rhpR-D70A_rhpS-Mutant_KB_R1_peak_46 | 2255 | 2539472 | 2540776 | - | upstream | -47 | 47 | NearestLocation | PSPPH_2185 |
| chromosome | 2576008 | 2576146 | 139 | * | rhpR-D70A_rhpS-Mutant_KB_R1_peak_47 | 2292 | 2576054 | 2579539 | + | overlapStart | -46 | 46 | NearestLocation | PSPPH_2220 |
| chromosome | 2702582 | 2702733 | 152 | * | rhpR-D70A_rhpS-Mutant_KB_R1_peak_48 | 2404 | 2702658 | 2702894 | - | overlapEnd | 312 | 75 | NearestLocation | PSPPH_2326 |
| chromosome | 2936611 | 2936758 | 148 | * | rhpR-D70A_rhpS-Mutant_KB_R1_peak_49 | 2630 | 2934900 | 2936543 | - | upstream | -68 | 68 | NearestLocation | PSPPH_2545 |
| chromosome | 3018398 | 3018533 | 136 | * | rhpR-D70A_rhpS-Mutant_KB_R1_peak_50 | 2704 | 3016634 | 3018292 | - | upstream | -106 | 106 | NearestLocation | PSPPH_2618 |
| chromosome | 3033856 | 3033970 | 115 | * | rhpR-D70A_rhpS-Mutant_KB_R1_peak_51 | 2715 | 3032695 | 3033858 | - | overlapStart | 2 | 2 | NearestLocation | PSPPH_2628 |
| chromosome | 3033856 | 3033970 | 115 | * | rhpR-D70A_rhpS-Mutant_KB_R1_peak_51 | 2716 | 3032695 | 3033858 | - | overlapStart | 2 | 2 | NearestLocation | PSPPH_2628 |
| chromosome | 3163035 | 3163143 | 109 | * | rhpR-D70A_rhpS-Mutant_KB_R1_peak_52 | 2821 | 3162689 | 3163060 | - | overlapStart | 25 | 25 | NearestLocation | PSPPH_2731 |
| chromosome | 3231589 | 3231707 | 119 | * | rhpR-D70A_rhpS-Mutant_KB_R1_peak_53 | 2883 | 3231624 | 3231971 | + | overlapStart | -35 | 35 | NearestLocation | PSPPH_2792 |
| chromosome | 3276151 | 3276235 | 85 | * | rhpR-D70A_rhpS-Mutant_KB_R1_peak_54 | 2924 | 3275015 | 3276636 | - | inside | 485 | 401 | NearestLocation | PSPPH_2830 |
| chromosome | 3276151 | 3276235 | 85 | * | rhpR-D70A_rhpS-Mutant_KB_R1_peak_54 | 2925 | 3275015 | 3276636 | - | inside | 485 | 401 | NearestLocation | PSPPH_2830 |
| chromosome | 3533543 | 3533624 | 82 | * | rhpR-D70A_rhpS-Mutant_KB_R1_peak_55 | 3143 | 3533706 | 3534569 | + | upstream | -163 | 82 | NearestLocation | PSPPH_3042 |
| chromosome | 3686427 | 3686567 | 141 | * | rhpR-D70A_rhpS-Mutant_KB_R1_peak_56 | 3289 | 3686569 | 3686865 | + | upstream | -142 | 2 | NearestLocation | PSPPH_3179 |
| chromosome | 3882259 | 3882335 | 77 | * | rhpR-D70A_rhpS-Mutant_KB_R1_peak_57 | 3468 | 3881964 | 3882443 | - | inside | 184 | 108 | NearestLocation | PSPPH_3354 |
| chromosome | 4031478 | 4031628 | 151 | * | rhpR-D70A_rhpS-Mutant_KB_R1_peak_58 | 3608 | 4031056 | 4031223 | + | downstream | 422 | 255 | NearestLocation | PSPPH_3490 |
| chromosome | 4036491 | 4036581 | 91 | * | rhpR-D70A_rhpS-Mutant_KB_R1_peak_59 | 3612 | 4034772 | 4035991 | - | upstream | -500 | 500 | NearestLocation | PSPPH_3494 |
| chromosome | 4036491 | 4036581 | 91 | * | rhpR-D70A_rhpS-Mutant_KB_R1_peak_59 | 3613 | 4034772 | 4035991 | - | upstream | -500 | 500 | NearestLocation | PSPPH_3494 |
| chromosome | 4052294 | 4052395 | 102 | * | rhpR-D70A_rhpS-Mutant_KB_R1_peak_60 | 3634 | 4052585 | 4053097 | + | upstream | -291 | 190 | NearestLocation | PSPPH_3514 |
| chromosome | 4069536 | 4069653 | 118 | * | rhpR-D70A_rhpS-Mutant_KB_R1_peak_61 | 3649 | 4069214 | 4069480 | + | downstream | 322 | 56 | NearestLocation | PSPPH_3529 |
| chromosome | 4202424 | 4202512 | 89 | * | rhpR-D70A_rhpS-Mutant_KB_R1_peak_62 | 3780 | 4201524 | 4202504 | - | overlapStart | 80 | 8 | NearestLocation | PSPPH_3658 |
| chromosome | 4233201 | 4233314 | 114 | * | rhpR-D70A_rhpS-Mutant_KB_R1_peak_63 | 3807 | 4232944 | 4233237 | + | overlapEnd | 257 | 36 | NearestLocation | PSPPH_3685 |
| chromosome | 4233447 | 4233715 | 269 | * | rhpR-D70A_rhpS-Mutant_KB_R1_peak_64 | 3807 | 4232944 | 4233237 | + | downstream | 503 | 210 | NearestLocation | PSPPH_3685 |
| chromosome | 4236032 | 4236307 | 276 | * | rhpR-D70A_rhpS-Mutant_KB_R1_peak_65 | 3810 | 4236460 | 4236762 | + | upstream | -428 | 153 | NearestLocation | PSPPH_3688 |
| chromosome | 4332307 | 4332421 | 115 | * | rhpR-D70A_rhpS-Mutant_KB_R1_peak_66 | 3905 | 4332394 | 4334109 | + | overlapStart | -87 | 27 | NearestLocation | PSPPH_3783 |
| chromosome | 4349852 | 4349947 | 96 | * | rhpR-D70A_rhpS-Mutant_KB_R1_peak_67 | 3920 | 4349926 | 4350852 | + | overlapStart | -74 | 21 | NearestLocation | PSPPH_3798 |
| chromosome | 4402260 | 4402391 | 132 | * | rhpR-D70A_rhpS-Mutant_KB_R1_peak_68 | 3967 | 4402378 | 4402725 | + | overlapStart | -118 | 13 | NearestLocation | PSPPH_3845 |
| chromosome | 4518284 | 4518421 | 138 | * | rhpR-D70A_rhpS-Mutant_KB_R1_peak_69 | 4079 | 4517832 | 4518413 | - | overlapStart | 129 | 8 | NearestLocation | PSPPH_3955 |
| chromosome | 4578375 | 4578514 | 140 | * | rhpR-D70A_rhpS-Mutant_KB_R1_peak_70 | 4136 | 4578772 | 4579128 | + | upstream | -397 | 258 | NearestLocation | PSPPH_4007 |
| chromosome | 4580511 | 4580606 | 96 | * | rhpR-D70A_rhpS-Mutant_KB_R1_peak_71 | 4139 | 4580595 | 4582079 | + | overlapStart | -84 | 11 | NearestLocation | PSPPH_4010 |
| chromosome | 4596316 | 4596478 | 163 | * | rhpR-D70A_rhpS-Mutant_KB_R1_peak_72 | 4150 | 4596548 | 4596856 | + | upstream | -232 | 70 | NearestLocation | PSPPH_4021 |
| chromosome | 4720385 | 4720514 | 130 | * | rhpR-D70A_rhpS-Mutant_KB_R1_peak_73 | 4270 | 4719440 | 4720357 | - | upstream | -28 | 28 | NearestLocation | PSPPH_4133 |
| chromosome | 4736434 | 4736564 | 131 | * | rhpR-D70A_rhpS-Mutant_KB_R1_peak_74 | 4289 | 4736613 | 4736921 | + | upstream | -179 | 49 | NearestLocation | PSPPH_4152 |
| chromosome | 4780785 | 4780921 | 137 | * | rhpR-D70A_rhpS-Mutant_KB_R1_peak_75 | 4329 | 4780755 | 4780831 | - | overlapStart | 46 | 30 | NearestLocation | PSPPH_4192 |
| chromosome | 4800111 | 4800382 | 272 | * | rhpR-D70A_rhpS-Mutant_KB_R1_peak_76 | 4347 | 4800318 | 4800839 | + | overlapStart | -207 | 64 | NearestLocation | PSPPH_4210 |
| chromosome | 4804258 | 4804352 | 95 | * | rhpR-D70A_rhpS-Mutant_KB_R1_peak_77 | 4353 | 4804338 | 4804725 | + | overlapStart | -80 | 14 | NearestLocation | PSPPH_4216 |
| chromosome | 4847813 | 4847909 | 97 | * | rhpR-D70A_rhpS-Mutant_KB_R1_peak_78 | 4387 | 4848143 | 4848382 | - | downstream | 569 | 234 | NearestLocation | PSPPH_4249 |
| chromosome | 4908985 | 4909216 | 232 | * | rhpR-D70A_rhpS-Mutant_KB_R1_peak_79 | 4436 | 4907552 | 4908361 | - | upstream | -624 | 624 | NearestLocation | PSPPH_4298 |
| chromosome | 4946894 | 4947056 | 163 | * | rhpR-D70A_rhpS-Mutant_KB_R1_peak_80 | 4466 | 4947036 | 4948289 | + | overlapStart | -142 | 20 | NearestLocation | PSPPH_4327 |
| chromosome | 5148557 | 5148743 | 187 | * | rhpR-D70A_rhpS-Mutant_KB_R1_peak_81 | 4658 | 5148798 | 5149484 | + | upstream | -241 | 55 | NearestLocation | PSPPH_4518 |
| chromosome | 5172345 | 5172468 | 124 | * | rhpR-D70A_rhpS-Mutant_KB_R1_peak_82 | 4678 | 5172524 | 5173330 | + | upstream | -179 | 56 | NearestLocation | PSPPH_4538 |
| chromosome | 5216605 | 5216752 | 148 | * | rhpR-D70A_rhpS-Mutant_KB_R1_peak_83 | 4712 | 5216253 | 5216609 | - | overlapStart | 4 | 4 | NearestLocation | PSPPH_4570 |
| chromosome | 5231927 | 5232090 | 164 | * | rhpR-D70A_rhpS-Mutant_KB_R1_peak_84 | 4739 | 5231556 | 5231927 | - | overlapStart | 0 | 0 | NearestLocation | PSPPH_4597 |
| chromosome | 5243271 | 5243349 | 79 | * | rhpR-D70A_rhpS-Mutant_KB_R1_peak_85 | 4746 | 5243077 | 5243610 | - | inside | 339 | 194 | NearestLocation | PSPPH_4604 |
| chromosome | 5243525 | 5243657 | 133 | * | rhpR-D70A_rhpS-Mutant_KB_R1_peak_86 | 4746 | 5243077 | 5243610 | - | overlapStart | 85 | 47 | NearestLocation | PSPPH_4604 |
| chromosome | 5244064 | 5244192 | 129 | * | rhpR-D70A_rhpS-Mutant_KB_R1_peak_87 | 4748 | 5244035 | 5244110 | - | overlapStart | 46 | 29 | NearestLocation | PSPPH_4606 |
| chromosome | 5244352 | 5244565 | 214 | * | rhpR-D70A_rhpS-Mutant_KB_R1_peak_88 | 4749 | 5244276 | 5244351 | - | upstream | -1 | 1 | NearestLocation | PSPPH_4607 |
| chromosome | 5323252 | 5323435 | 184 | * | rhpR-D70A_rhpS-Mutant_KB_R1_peak_89 | 4829 | 5323252 | 5323328 | - | overlapStart | 76 | 0 | NearestLocation | PSPPH_4687 |
| chromosome | 5492505 | 5492607 | 103 | * | rhpR-D70A_rhpS-Mutant_KB_R1_peak_90 | 4983 | 5493584 | 5494279 | + | upstream | -1079 | 977 | NearestLocation | PSPPH_4834 |
| chromosome | 5545991 | 5546070 | 80 | * | rhpR-D70A_rhpS-Mutant_KB_R1_peak_91 | 5029 | 5545898 | 5546554 | + | inside | 93 | 93 | NearestLocation | PSPPH_4876 |
| chromosome | 5554951 | 5555056 | 106 | * | rhpR-D70A_rhpS-Mutant_KB_R1_peak_92 | 5039 | 5555056 | 5556345 | + | overlapStart | -105 | 0 | NearestLocation | PSPPH_4885 |
| chromosome | 5621339 | 5621437 | 99 | * | rhpR-D70A_rhpS-Mutant_KB_R1_peak_93 | 5104 | 5621094 | 5621216 | - | upstream | -123 | 123 | NearestLocation | PSPPH_4948 |
| chromosome | 5643739 | 5644031 | 293 | * | rhpR-D70A_rhpS-Mutant_KB_R1_peak_94 | 5133 | 5643544 | 5643945 | + | overlapEnd | 195 | 86 | NearestLocation | PSPPH_4975 |
| chromosome | 5652298 | 5652391 | 94 | * | rhpR-D70A_rhpS-Mutant_KB_R1_peak_95 | 5139 | 5651658 | 5652302 | - | overlapStart | 4 | 4 | NearestLocation | PSPPH_4981 |
| chromosome | 5750944 | 5751042 | 99 | * | rhpR-D70A_rhpS-Mutant_KB_R1_peak_96 | 5231 | 5751110 | 5751574 | + | upstream | -166 | 68 | NearestLocation | PSPPH_5071 |
| chromosome | 5790906 | 5791076 | 171 | * | rhpR-D70A_rhpS-Mutant_KB_R1_peak_97 | 5264 | 5791020 | 5791235 | + | overlapStart | -114 | 56 | NearestLocation | PSPPH_5102 |
| chromosome | 5903131 | 5903267 | 137 | * | rhpR-D70A_rhpS-Mutant_KB_R1_peak_98 | 5368 | 5903367 | 5904626 | + | upstream | -236 | 100 | NearestLocation | PSPPH_5202 |
| chromosome | 5922358 | 5922460 | 103 | * | rhpR-D70A_rhpS-Mutant_KB_R1_peak_99 | 5384 | 5920516 | 5922408 | - | overlapStart | 50 | 50 | NearestLocation | PSPPH_5218 |

**F. ChIP-seq result for RhpR^D70A^ binding sites in *Pseudomonas savastanoi* pv. *phaseolicola* 1448A *rhpS* mutant in MM**

| seqnames | start | end | width | strand | peak | feature | start_position | end_position | feature_strand | insideFeature | distancetoFeature | shortestDistance | fromOverlappingOrNearest | id |
| --- | --- | --- | --- | --- | --- | --- | --- | --- | --- | --- | --- | --- | --- | --- |
| chromosome | 982761 | 982916 | 156 | * | rhpR-D70A_rhpS-Mutant_MM_R1_peak_1 | 844 | 982619 | 983014 | + | inside | 142 | 98 | NearestLocation | PSPPH_0821 |
| chromosome | 1024657 | 1024945 | 289 | * | rhpR-D70A_rhpS-Mutant_MM_R1_peak_2 | 880 | 1023074 | 1024549 | - | upstream | -108 | 108 | NearestLocation | PSPPH_0856 |
| chromosome | 1125929 | 1126024 | 96 | * | rhpR-D70A_rhpS-Mutant_MM_R1_peak_3 | 975 | 1125905 | 1125981 | - | overlapStart | 52 | 24 | NearestLocation | PSPPH_0948 |
| chromosome | 1738501 | 1738724 | 224 | * | rhpR-D70A_rhpS-Mutant_MM_R1_peak_4 | 1540 | 1736957 | 1738255 | - | upstream | -246 | 246 | NearestLocation | PSPPH_1496 |
| chromosome | 1952606 | 1952722 | 117 | * | rhpR-D70A_rhpS-Mutant_MM_R1_peak_5 | 1741 | 1952689 | 1952764 | + | overlapStart | -83 | 33 | NearestLocation | PSPPH_1689 |
| chromosome | 2350461 | 2350552 | 92 | * | rhpR-D70A_rhpS-Mutant_MM_R1_peak_6 | 2064 | 2350640 | 2351398 | - | downstream | 937 | 88 | NearestLocation | PSPPH_2002 |
| chromosome | 2351374 | 2351586 | 213 | * | rhpR-D70A_rhpS-Mutant_MM_R1_peak_7 | 2064 | 2350640 | 2351398 | - | overlapStart | 24 | 24 | NearestLocation | PSPPH_2002 |
| chromosome | 2352546 | 2353796 | 1251 | * | rhpR-D70A_rhpS-Mutant_MM_R1_peak_8 | 2066 | 2352536 | 2353285 | - | overlapStart | 739 | 10 | NearestLocation | PSPPH_2004 |
| chromosome | 2437690 | 2437772 | 83 | * | rhpR-D70A_rhpS-Mutant_MM_R1_peak_9 | 2143 | 2437666 | 2437992 | - | inside | 302 | 24 | NearestLocation | PSPPH_2078 |
| chromosome | 2437956 | 2438084 | 129 | * | rhpR-D70A_rhpS-Mutant_MM_R1_peak_10 | 2143 | 2437666 | 2437992 | - | overlapStart | 36 | 36 | NearestLocation | PSPPH_2078 |
| chromosome | 3018408 | 3018495 | 88 | * | rhpR-D70A_rhpS-Mutant_MM_R1_peak_11 | 2704 | 3016634 | 3018292 | - | upstream | -116 | 116 | NearestLocation | PSPPH_2618 |
| chromosome | 3018899 | 3019077 | 179 | * | rhpR-D70A_rhpS-Mutant_MM_R1_peak_12 | 2705 | 3018875 | 3019948 | + | inside | 24 | 24 | NearestLocation | PSPPH_2619 |
| chromosome | 4036516 | 4036602 | 87 | * | rhpR-D70A_rhpS-Mutant_MM_R1_peak_13 | 3612 | 4034772 | 4035991 | - | upstream | -525 | 525 | NearestLocation | PSPPH_3494 |
| chromosome | 4036516 | 4036602 | 87 | * | rhpR-D70A_rhpS-Mutant_MM_R1_peak_13 | 3613 | 4034772 | 4035991 | - | upstream | -525 | 525 | NearestLocation | PSPPH_3494 |
| chromosome | 4236142 | 4236324 | 183 | * | rhpR-D70A_rhpS-Mutant_MM_R1_peak_14 | 3810 | 4236460 | 4236762 | + | upstream | -318 | 136 | NearestLocation | PSPPH_3688 |
| chromosome | 4349858 | 4349948 | 91 | * | rhpR-D70A_rhpS-Mutant_MM_R1_peak_15 | 3920 | 4349926 | 4350852 | + | overlapStart | -68 | 22 | NearestLocation | PSPPH_3798 |
| chromosome | 4712126 | 4712243 | 118 | * | rhpR-D70A_rhpS-Mutant_MM_R1_peak_16 | 4263 | 4711669 | 4712097 | - | upstream | -29 | 29 | NearestLocation | PSPPH_4126 |
| chromosome | 5216568 | 5216747 | 180 | * | rhpR-D70A_rhpS-Mutant_MM_R1_peak_17 | 4712 | 5216253 | 5216609 | - | overlapStart | 41 | 41 | NearestLocation | PSPPH_4570 |
| chromosome | 5231927 | 5232068 | 142 | * | rhpR-D70A_rhpS-Mutant_MM_R1_peak_18 | 4739 | 5231556 | 5231927 | - | overlapStart | 0 | 0 | NearestLocation | PSPPH_4597 |

**G.** **Phosphorylation-dependent RhpR binding sites**

| gene_id | name | Product Name | seqnames | start | end | width | strand | peak | feature | start_position | end_position | feature_strand | insideFeature | distancetoFeature | shortestDistance | fromOverlappingOrNearest |
| --- | --- | --- | --- | --- | --- | --- | --- | --- | --- | --- | --- | --- | --- | --- | --- | --- |
| PSPPH_0001 | dnaA | chromosome replication initiator DnaA | chromosome | 148 | 227 | 80 | * | rhpR_rhpS-Mutant_KB_R1_peak_1 | 1 | 238 | 1773 | + | upstream | -90 | 11 | NearestLocation |
| PSPPH_0101 |  | ParB family protein | chromosome | 111861 | 112043 | 183 | * | rhpR_rhpS-Mutant_KB_R1_peak_4 | 104 | 112706 | 113614 | + | upstream | -845 | 663 | NearestLocation |
| PSPPH_0278 |  | DedA family protein | chromosome | 320810 | 320917 | 108 | * | rhpR_rhpS-Mutant_MM_R1_peak_10 | 288 | 320118 | 320780 | - | upstream | -30 | 30 | NearestLocation |
| PSPPH_0297 |  | pseudogene | chromosome | 341484 | 341609 | 126 | * | rhpR_rhpS-Mutant_MM_R1_peak_12 | 307 | 339414 | 341467 | - | upstream | -17 | 17 | NearestLocation |
| PSPPH_0487 |  | Na+/proline symporter | chromosome | 554950 | 555028 | 79 | * | rhpR_rhpS-Mutant_KB_R1_peak_19 | 499 | 555043 | 556275 | + | upstream | -93 | 15 | NearestLocation |
| PSPPH_0536 | cytX | hydroxymethylpyrimidine transporter CytX | chromosome | 630657 | 630775 | 119 | * | rhpR_rhpS-Mutant_KB_R1_peak_20 | 549 | 630951 | 632069 | + | upstream | -294 | 176 | NearestLocation |
| PSPPH_0609 |  | hypothetical protein | chromosome | 718545 | 718664 | 120 | * | rhpR_rhpS-Mutant_KB_R1_peak_22 | 622 | 718866 | 719993 | + | upstream | -321 | 202 | NearestLocation |
| PSPPH_0611 |  | hypothetical protein | chromosome | 721894 | 721985 | 92 | * | rhpR_rhpS-Mutant_KB_R1_peak_23 | 624 | 722115 | 723011 | + | upstream | -221 | 130 | NearestLocation |
| PSPPH_0620 | dnaG | DNA primase | chromosome | 736817 | 736988 | 172 | * | rhpR_rhpS-Mutant_KB_R1_peak_26 | 634 | 734688 | 736643 | - | upstream | -174 | 174 | NearestLocation |
| PSPPH_0651 |  | prophage PSPPH01 transcriptional regulator | chromosome | 771258 | 771360 | 103 | * | rhpR_rhpS-Mutant_KB_R1_peak_30 | 665 | 770972 | 771238 | - | upstream | -20 | 20 | NearestLocation |
| PSPPH_0667 |  | prophage PSPPH01, site-specific recombinase phage integrase | chromosome | 782730 | 782833 | 104 | * | rhpR_rhpS-Mutant_KB_R1_peak_31 | 682 | 783191 | 784366 | + | upstream | -461 | 358 | NearestLocation |
| PSPPH_0731 |  | type IV pilus biogenesis proteinAdd | chromosome | 854677 | 854756 | 80 | * | rhpR_rhpS-Mutant_KB_R1_peak_33 | 747 | 854922 | 855326 | + | upstream | -245 | 166 | NearestLocation |
| PSPPH_0749 |  | hypothetical protein | chromosome | 875181 | 875299 | 119 | * | rhpR_rhpS-Mutant_KB_R1_peak_36 | 765 | 873205 | 874737 | - | upstream | -444 | 444 | NearestLocation |
| PSPPH_0755 |  | TetR family transcriptional regulator | chromosome | 882201 | 882500 | 300 | * | rhpR_rhpS-Mutant_KB_R1_peak_37 | 772 | 882507 | 883145 | + | upstream | -306 | 7 | NearestLocation |
| PSPPH_0756 |  | glycosyl hydrolase | chromosome | 883598 | 883747 | 150 | * | rhpR_rhpS-Mutant_KB_R1_peak_38 | 773 | 884445 | 887003 | + | upstream | -847 | 698 | NearestLocation |
| PSPPH_0771 |  | diguanylate phosphodiesterase | chromosome | 905396 | 905561 | 166 | * | rhpR_rhpS-Mutant_KB_R1_peak_44 | 790 | 904056 | 904868 | - | upstream | -528 | 528 | NearestLocation |
| PSPPH_0778 |  | LuxR family transcriptional regulator | chromosome | 914183 | 914262 | 80 | * | rhpR_rhpS-Mutant_KB_R1_peak_46 | 798 | 914293 | 914931 | + | upstream | -110 | 31 | NearestLocation |
| PSPPH_0791 |  | lipoprotein | chromosome | 935615 | 935753 | 139 | * | rhpR_rhpS-Mutant_KB_R1_peak_47 | 814 | 935833 | 936333 | + | upstream | -218 | 80 | NearestLocation |
| PSPPH_0856 | pcnB | poly(A) polymerase | chromosome | 1024797 | 1024930 | 134 | * | rhpR_rhpS-Mutant_KB_R1_peak_51 | 880 | 1023074 | 1024549 | - | upstream | -248 | 248 | NearestLocation |
| PSPPH_0868 |  | penicillin-binding protein 1B | chromosome | 1037873 | 1037980 | 108 | * | rhpR_rhpS-Mutant_KB_R1_peak_52 | 892 | 1038016 | 1040337 | + | upstream | -143 | 36 | NearestLocation |
| PSPPH_0950 |  | methyl-accepting chemotaxis protein | chromosome | 1127340 | 1127494 | 155 | * | rhpR_rhpS-Mutant_KB_R1_peak_56 | 977 | 1127559 | 1129616 | + | upstream | -219 | 65 | NearestLocation |
| PSPPH_0953 | gmd | GDP-mannose 4,6-dehydratase | chromosome | 1132895 | 1132974 | 80 | * | rhpR_rhpS-Mutant_KB_R1_peak_57 | 980 | 1131805 | 1132836 | - | upstream | -59 | 59 | NearestLocation |
| PSPPH_0969 | rfbB2 | dTDP-glucose 4,6-dehydratase | chromosome | 1153581 | 1153733 | 153 | * | rhpR_rhpS-Mutant_KB_R1_peak_62 | 996 | 1152260 | 1153360 | - | upstream | -221 | 221 | NearestLocation |
| PSPPH_1001 |  | hypothetical protein | chromosome | 1194275 | 1194407 | 133 | * | rhpR_rhpS-Mutant_KB_R1_peak_65 | 1028 | 1194579 | 1195097 | + | upstream | -304 | 172 | NearestLocation |
| PSPPH_1042 |  | pseudogene | chromosome | 1232403 | 1232569 | 167 | * | rhpR_rhpS-Mutant_KB_R1_peak_67 | 1069 | 1232666 | 1235402 | + | upstream | -263 | 97 | NearestLocation |
| PSPPH_1086 |  | lipoprotein | chromosome | 1279082 | 1279250 | 169 | * | rhpR_rhpS-Mutant_KB_R1_peak_70 | 1114 | 1278735 | 1279067 | - | upstream | -15 | 15 | NearestLocation |
| PSPPH_1090 |  | ribosomal subunit interface protein | chromosome | 1284131 | 1284212 | 82 | * | rhpR_rhpS-Mutant_KB_R1_peak_71 | 1119 | 1283687 | 1284115 | - | upstream | -16 | 16 | NearestLocation |
| PSPPH_1118 | algD | alginate biosynthesis protein AlgD | chromosome | 1318943 | 1319018 | 76 | * | rhpR_rhpS-Mutant_MM_R1_peak_48 | 1148 | 1317259 | 1318575 | - | upstream | -368 | 368 | NearestLocation |
| PSPPH_1147 |  | pseudogene | chromosome | 1347960 | 1348101 | 142 | * | rhpR_rhpS-Mutant_KB_R1_peak_76 | 1177 | 1345493 | 1347374 | - | upstream | -586 | 586 | NearestLocation |
| PSPPH_1181 |  | glucose ABC transporter substrate-binding protein | chromosome | 1387191 | 1387329 | 139 | * | rhpR_rhpS-Mutant_KB_R1_peak_80 | 1212 | 1387352 | 1388638 | + | upstream | -161 | 23 | NearestLocation |
| PSPPH_1270 | hrpR | type III transcriptional regulator HrpR | chromosome | 1484357 | 1484825 | 469 | * | rhpR_rhpS-Mutant_KB_R1_peak_83 | 1309 | 1485638 | 1486558 | + | upstream | -1281 | 813 | NearestLocation |
| PSPPH_1333 | guaB | inosine 5'-monophosphate dehydrogenase | chromosome | 1543564 | 1543650 | 87 | * | rhpR_rhpS-Mutant_KB_R1_peak_84 | 1373 | 1543651 | 1545120 | + | upstream | -87 | 1 | NearestLocation |
| PSPPH_1523 |  | serine protease | chromosome | 1772107 | 1772193 | 87 | * | rhpR_rhpS-Mutant_KB_R1_peak_88 | 1567 | 1772229 | 1773839 | + | upstream | -122 | 36 | NearestLocation |
| PSPPH_1698 | clpP | ATP-dependent Clp protease proteolytic subunit | chromosome | 1961958 | 1962055 | 98 | * | rhpR_rhpS-Mutant_KB_R1_peak_93 | 1750 | 1962205 | 1962846 | + | upstream | -247 | 150 | NearestLocation |
| PSPPH_1701 | hupB | DNA-binding protein HU-beta | chromosome | 1966456 | 1966572 | 117 | * | rhpR_rhpS-Mutant_KB_R1_peak_94 | 1753 | 1966990 | 1967262 | + | upstream | -534 | 418 | NearestLocation |
| PSPPH_1829 |  | hypothetical protein | chromosome | 2129442 | 2129534 | 93 | * | rhpR_rhpS-Mutant_MM_R1_peak_63 | 1885 | 2129672 | 2130631 | + | upstream | -230 | 138 | NearestLocation |
| PSPPH_1840 |  | lipoprotein | chromosome | 2137907 | 2138002 | 96 | * | rhpR_rhpS-Mutant_KB_R1_peak_100 | 1897 | 2137193 | 2137723 | - | upstream | -184 | 184 | NearestLocation |
| PSPPH_2003 | rhpS | sensor histidine kinase | chromosome | 2352569 | 2352701 | 133 | * | rhpR_rhpS-Mutant_KB_R1_peak_109 | 2065 | 2351514 | 2352539 | - | upstream | -30 | 30 | NearestLocation |
| PSPPH_2013 | fimA | type I fimbrial protein FimA | chromosome | 2366260 | 2366335 | 76 | * | rhpR_rhpS-Mutant_MM_R1_peak_71 | 2076 | 2365654 | 2366211 | - | upstream | -49 | 49 | NearestLocation |
| PSPPH_2068 |  | OprF | chromosome | 2422857 | 2423039 | 183 | * | rhpR_rhpS-Mutant_KB_R1_peak_112 | 2133 | 2423246 | 2424280 | + | upstream | -389 | 207 | NearestLocation |
| PSPPH_2080 |  | CHASE domain-containing protein/sensory box histidine kinase | chromosome | 2440689 | 2440830 | 142 | * | rhpR_rhpS-Mutant_KB_R1_peak_115 | 2145 | 2440865 | 2443255 | + | upstream | -176 | 35 | NearestLocation |
| PSPPH_2132 |  | hypothetical protein | chromosome | 2489324 | 2489442 | 119 | * | rhpR_rhpS-Mutant_KB_R1_peak_117 | 2197 | 2489070 | 2489291 | - | upstream | -33 | 33 | NearestLocation |
| PSPPH_2186 |  | metalloprotease | chromosome | 2541094 | 2541266 | 173 | * | rhpR_rhpS-Mutant_KB_R1_peak_121 | 2256 | 2541290 | 2542897 | + | upstream | -196 | 24 | NearestLocation |
| PSPPH_2194 |  | Cro/CI family transcriptional regulator | chromosome | 2550576 | 2550663 | 88 | * | rhpR_rhpS-Mutant_KB_R1_peak_122 | 2264 | 2550022 | 2550513 | - | upstream | -63 | 63 | NearestLocation |
| PSPPH_2197 |  | pseudogene | chromosome | 2554348 | 2554465 | 118 | * | rhpR_rhpS-Mutant_KB_R1_peak_123 | 2267 | 2553044 | 2554293 | - | upstream | -55 | 55 | NearestLocation |
| PSPPH_2279 |  | ABC transporter protein, ATP binding component | chromosome | 2644978 | 2645076 | 99 | * | rhpR_rhpS-Mutant_KB_R1_peak_125 | 2353 | 2645116 | 2646675 | + | upstream | -138 | 40 | NearestLocation |
| PSPPH_2628 |  | pseudogene | chromosome | 3034197 | 3034363 | 167 | * | rhpR_rhpS-Mutant_MM_R1_peak_87 | 2715 | 3032695 | 3033858 | - | upstream | -339 | 339 | NearestLocation |
| PSPPH_2653 |  | lipopolysaccharide core biosynthesis domain-containing protein | chromosome | 3077391 | 3077489 | 99 | * | rhpR_rhpS-Mutant_KB_R1_peak_133 | 2742 | 3076506 | 3077354 | - | upstream | -37 | 37 | NearestLocation |
| PSPPH_2747 |  | ECF subfamily RNA polymerase sigma factor | chromosome | 3178813 | 3178960 | 148 | * | rhpR_rhpS-Mutant_KB_R1_peak_135 | 2837 | 3179070 | 3179609 | + | upstream | -257 | 110 | NearestLocation |
| PSPPH_2825 |  | hypothetical protein | chromosome | 3271715 | 3271827 | 113 | * | rhpR_rhpS-Mutant_KB_R1_peak_139 | 2919 | 3272037 | 3272318 | + | upstream | -322 | 210 | NearestLocation |
| PSPPH_3037 |  | TonB-dependent siderophore receptor | chromosome | 3529192 | 3529289 | 98 | * | rhpR_rhpS-Mutant_MM_R1_peak_95 | 3138 | 3527024 | 3529150 | - | upstream | -42 | 42 | NearestLocation |
| PSPPH_3042 |  | 3-hydroxyacyl-CoA-acyl carrier protein transferase | chromosome | 3533576 | 3533662 | 87 | * | rhpR_rhpS-Mutant_MM_R1_peak_96 | 3143 | 3533706 | 3534569 | + | upstream | -130 | 44 | NearestLocation |
| PSPPH_3076 |  | pseudogene | chromosome | 3575363 | 3575625 | 263 | * | rhpR_rhpS-Mutant_KB_R1_peak_141 | 3181 | 3573928 | 3575358 | - | upstream | -5 | 5 | NearestLocation |
| PSPPH_3098 | clpS | ATP-dependent Clp protease adaptor protein ClpS | chromosome | 3596205 | 3596300 | 96 | * | rhpR_rhpS-Mutant_KB_R1_peak_143 | 3205 | 3595796 | 3596158 | - | upstream | -47 | 47 | NearestLocation |
| PSPPH_3128 |  | peptidase propeptide/YPEB domain-containing protein | chromosome | 3630510 | 3630590 | 81 | * | rhpR_rhpS-Mutant_KB_R1_peak_145 | 3235 | 3630124 | 3630432 | - | upstream | -78 | 78 | NearestLocation |
| PSPPH_3221 |  | lipid kinase | chromosome | 3733190 | 3733274 | 85 | * | rhpR_rhpS-Mutant_KB_R1_peak_147 | 3333 | 3732095 | 3733012 | - | upstream | -178 | 178 | NearestLocation |
| PSPPH_3276 |  | sensory box sensor histidine kinase/response regulator | chromosome | 3799019 | 3799102 | 84 | * | rhpR_rhpS-Mutant_MM_R1_peak_102 | 3388 | 3796797 | 3798890 | - | upstream | -129 | 129 | NearestLocation |
| PSPPH_3331 |  | hypothetical protein | chromosome | 3857198 | 3857309 | 112 | * | rhpR_rhpS-Mutant_KB_R1_peak_149 | 3445 | 3857400 | 3857924 | + | upstream | -202 | 91 | NearestLocation |
| PSPPH_3374 | fliL | flagellar basal body protein FliL | chromosome | 3902040 | 3902187 | 148 | * | rhpR_rhpS-Mutant_KB_R1_peak_152 | 3488 | 3901528 | 3902028 | - | upstream | -12 | 12 | NearestLocation |
| PSPPH_3402 | flgF | flagellar basal body rod protein FlgF | chromosome | 3937419 | 3937520 | 102 | * | rhpR_rhpS-Mutant_KB_R1_peak_153 | 3516 | 3936650 | 3937390 | - | upstream | -29 | 29 | NearestLocation |
| PSPPH_3473 |  | sensory box sensor histidine kinase/response regulator | chromosome | 4012876 | 4012997 | 122 | * | rhpR_rhpS-Mutant_KB_R1_peak_155 | 3591 | 4013029 | 4015101 | + | upstream | -153 | 32 | NearestLocation |
| PSPPH_3504 |  | alginate biosynthesis transcriptional activator | chromosome | 4043157 | 4043243 | 87 | * | rhpR_rhpS-Mutant_KB_R1_peak_157 | 3624 | 4043310 | 4043636 | + | upstream | -153 | 67 | NearestLocation |
| PSPPH_3560 |  | NAD-specific glutamate dehydrogenase | chromosome | 4102131 | 4102225 | 95 | * | rhpR_rhpS-Mutant_KB_R1_peak_159 | 3680 | 4097194 | 4102053 | - | upstream | -78 | 78 | NearestLocation |
| PSPPH_3674 |  | hypothetical protein | chromosome | 4222319 | 4222449 | 131 | * | rhpR_rhpS-Mutant_KB_R1_peak_162 | 3796 | 4221051 | 4221836 | - | upstream | -483 | 483 | NearestLocation |
| PSPPH_3737 | rstA | DNA-binding response regulator RstA | chromosome | 4285415 | 4285570 | 156 | * | rhpR_rhpS-Mutant_KB_R1_peak_166 | 3859 | 4284455 | 4285192 | - | upstream | -223 | 223 | NearestLocation |
| PSPPH_3758 |  | riboflavin biosynthesis protein RibD domain-containing protein | chromosome | 4311266 | 4311370 | 105 | * | rhpR_rhpS-Mutant_MM_R1_peak_117 | 3880 | 4310221 | 4310874 | - | upstream | -392 | 392 | NearestLocation |
| PSPPH_3809 | rpoS | RNA polymerase sigma factor RpoS | chromosome | 4362421 | 4362591 | 171 | * | rhpR_rhpS-Mutant_KB_R1_peak_169 | 3931 | 4361212 | 4362219 | - | upstream | -202 | 202 | NearestLocation |
| PSPPH_3810 |  | lipoprotein | chromosome | 4363270 | 4363352 | 83 | * | rhpR_rhpS-Mutant_KB_R1_peak_170 | 3932 | 4362323 | 4363189 | - | upstream | -81 | 81 | NearestLocation |
| PSPPH_3951 | lepA | GTP-binding protein LepA | chromosome | 4514369 | 4514474 | 106 | * | rhpR_rhpS-Mutant_KB_R1_peak_173 | 4075 | 4512549 | 4514336 | - | upstream | -33 | 33 | NearestLocation |
| PSPPH_3995 |  | hypothetical protein | chromosome | 4561128 | 4561210 | 83 | * | rhpR_rhpS-Mutant_KB_R1_peak_176 | 4122 | 4559857 | 4561077 | - | upstream | -51 | 51 | NearestLocation |
| PSPPH_4037 |  | hypothetical protein | chromosome | 4612347 | 4612423 | 77 | * | rhpR_rhpS-Mutant_KB_R1_peak_179 | 4168 | 4611823 | 4612182 | - | upstream | -165 | 165 | NearestLocation |
| PSPPH_4173 | gatC | aspartyl/glutamyl-tRNA amidotransferase subunit C | chromosome | 4758236 | 4758321 | 86 | * | rhpR_rhpS-Mutant_KB_R1_peak_183 | 4310 | 4758378 | 4758665 | + | upstream | -142 | 57 | NearestLocation |
| PSPPH_4198 | ftsH | ATP-dependent metalloprotease FtsH | chromosome | 4786421 | 4786504 | 84 | * | rhpR_rhpS-Mutant_KB_R1_peak_185 | 4335 | 4784486 | 4786399 | - | upstream | -22 | 22 | NearestLocation |
| PSPPH_4218 |  | hypothetical protein | chromosome | 4806422 | 4806498 | 77 | * | rhpR_rhpS-Mutant_MM_R1_peak_128 | 4355 | 4806712 | 4808529 | + | upstream | -290 | 214 | NearestLocation |
| PSPPH_4373 |  | hypothetical protein | chromosome | 4994751 | 4994864 | 114 | * | rhpR_rhpS-Mutant_KB_R1_peak_198 | 4513 | 4995109 | 4995801 | + | upstream | -358 | 245 | NearestLocation |
| PSPPH_4542 |  | hypothetical protein | chromosome | 5177203 | 5177424 | 222 | * | rhpR_rhpS-Mutant_MM_R1_peak_137 | 4683 | 5177776 | 5178747 | + | upstream | -573 | 352 | NearestLocation |
| PSPPH_4561 |  | pseudogene | chromosome | 5206457 | 5206551 | 95 | * | rhpR_rhpS-Mutant_KB_R1_peak_207 | 4702 | 5204697 | 5206401 | - | upstream | -56 | 56 | NearestLocation |
| PSPPH_4578 | rpsH | 30S ribosomal protein S8 | chromosome | 5220685 | 5220791 | 107 | * | rhpR_rhpS-Mutant_KB_R1_peak_210 | 4720 | 5220254 | 5220646 | - | upstream | -39 | 39 | NearestLocation |
| PSPPH_4598 | rpoC | DNA-directed RNA polymerase subunit beta' | chromosome | 5236475 | 5236580 | 106 | * | rhpR_rhpS-Mutant_KB_R1_peak_212 | 4740 | 5232126 | 5236325 | - | upstream | -150 | 150 | NearestLocation |
| PSPPH_4609 |  | tRNA-Tyr | chromosome | 5244565 | 5244674 | 110 | * | rhpR_rhpS-Mutant_KB_R1_peak_217 | 4751 | 5244476 | 5244560 | - | upstream | -5 | 5 | NearestLocation |
| PSPPH_4704 | pqqA | coenzyme PQQ synthesis protein PqqA | chromosome | 5341094 | 5341193 | 100 | * | rhpR_rhpS-Mutant_KB_R1_peak_222 | 4847 | 5341405 | 5341479 | + | upstream | -311 | 212 | NearestLocation |
| PSPPH_4801 |  | sensory box-containing diguanylate cyclase | chromosome | 5446433 | 5446623 | 191 | * | rhpR_rhpS-Mutant_KB_R1_peak_224 | 4947 | 5446674 | 5449367 | + | upstream | -241 | 51 | NearestLocation |
| PSPPH_4907 |  | OprD family outer membrane porin | chromosome | 5581731 | 5581928 | 198 | * | rhpR_rhpS-Mutant_KB_R1_peak_226 | 5062 | 5580213 | 5581574 | - | upstream | -157 | 157 | NearestLocation |
| PSPPH_5016 | gltP | glutamate/aspartate:proton symporter | chromosome | 5684153 | 5684242 | 90 | * | rhpR_rhpS-Mutant_KB_R1_peak_231 | 5174 | 5682626 | 5683957 | - | upstream | -196 | 196 | NearestLocation |
| PSPPH_5023 |  | hypothetical protein | chromosome | 5692144 | 5692302 | 159 | * | rhpR_rhpS-Mutant_KB_R1_peak_232 | 5181 | 5690781 | 5692085 | - | upstream | -59 | 59 | NearestLocation |
| PSPPH_5052 | tonB4 | ferric siderophore ABC transporter substrate-binding protein | chromosome | 5727310 | 5727559 | 250 | * | rhpR_rhpS-Mutant_KB_R1_peak_233 | 5212 | 5726420 | 5727232 | - | upstream | -78 | 78 | NearestLocation |
| PSPPH_5082 |  | mechanosensitive ion channel protein MscS | chromosome | 5764621 | 5764746 | 126 | * | rhpR_rhpS-Mutant_KB_R1_peak_237 | 5243 | 5764856 | 5765668 | + | upstream | -235 | 110 | NearestLocation |
| PSPPH_0004 | gyrB | DNA gyrase subunit B | chromosome | 4043 | 4194 | 152 | * | rhpR_rhpS-Mutant_KB_R1_peak_2 | 4 | 4046 | 6463 | + | overlapStart | -3 | 3 | NearestLocation |
| PSPPH_0098 |  | tRNA-Arg | chromosome | 109472 | 109565 | 94 | * | rhpR_rhpS-Mutant_MM_R1_peak_2 | 101 | 109533 | 109609 | + | overlapStart | -61 | 32 | NearestLocation |
| PSPPH_0138 |  | hypothetical protein | chromosome | 164372 | 164456 | 85 | * | rhpR_rhpS-Mutant_KB_R1_peak_7 | 143 | 163896 | 164447 | - | overlapStart | 75 | 9 | NearestLocation |
| PSPPH_0173 |  | acetylornithine deacetylase | chromosome | 207097 | 207205 | 109 | * | rhpR_rhpS-Mutant_MM_R1_peak_6 | 182 | 207138 | 207986 | + | overlapStart | -41 | 41 | NearestLocation |
| PSPPH_0275 | cynT | carbonic anhydrase | chromosome | 317706 | 317809 | 104 | * | rhpR_rhpS-Mutant_KB_R1_peak_14 | 285 | 317114 | 317779 | - | overlapStart | 73 | 30 | NearestLocation |
| PSPPH_0320 |  | TauD/TfdA family dioxygenase | chromosome | 364575 | 364904 | 330 | * | rhpR_rhpS-Mutant_KB_R1_peak_17 | 332 | 364889 | 365785 | + | overlapStart | -314 | 15 | NearestLocation |
| PSPPH_0476 |  | hypothetical protein | chromosome | 539619 | 539721 | 103 | * | rhpR_rhpS-Mutant_KB_R1_peak_18 | 488 | 539049 | 539621 | - | overlapStart | 2 | 2 | NearestLocation |
| PSPPH_0618 |  | pseudogene | chromosome | 732480 | 732759 | 280 | * | rhpR_rhpS-Mutant_KB_R1_peak_24 | 631 | 731881 | 732564 | - | overlapStart | 84 | 84 | NearestLocation |
| PSPPH_0650 |  | autotransporting lipase | chromosome | 770857 | 770992 | 136 | * | rhpR_rhpS-Mutant_KB_R1_peak_29 | 664 | 768944 | 770866 | - | overlapStart | 9 | 9 | NearestLocation |
| PSPPH_0742 | clpB2 | clpB protein | chromosome | 867986 | 868120 | 135 | * | rhpR_rhpS-Mutant_KB_R1_peak_35 | 758 | 868016 | 870580 | + | overlapStart | -30 | 30 | NearestLocation |
| PSPPH_0767 | hopG1 | type III effector HopG1 | chromosome | 898957 | 899058 | 102 | * | rhpR_rhpS-Mutant_KB_R1_peak_42 | 786 | 897416 | 898963 | - | overlapStart | 6 | 6 | NearestLocation |
| PSPPH_0813 |  | hypothetical protein | chromosome | 957950 | 958271 | 322 | * | rhpR_rhpS-Mutant_KB_R1_peak_48 | 836 | 958236 | 960398 | + | overlapStart | -286 | 35 | NearestLocation |
| PSPPH_0991 | prsA | ribose-phosphate pyrophosphokinase | chromosome | 1186093 | 1186271 | 179 | * | rhpR_rhpS-Mutant_KB_R1_peak_63 | 1018 | 1185154 | 1186095 | - | overlapStart | 2 | 2 | NearestLocation |
| PSPPH_1035 |  | lipoprotein | chromosome | 1223066 | 1223320 | 255 | * | rhpR_rhpS-Mutant_KB_R1_peak_66 | 1062 | 1223313 | 1224131 | + | overlapStart | -247 | 7 | NearestLocation |
| PSPPH_1120 |  | polysaccharide deacetylase | chromosome | 1320216 | 1320422 | 207 | * | rhpR_rhpS-Mutant_MM_R1_peak_50 | 1150 | 1320350 | 1321549 | + | overlapStart | -134 | 72 | NearestLocation |
| PSPPH_1725 |  | cation transporter | chromosome | 1999686 | 1999866 | 181 | * | rhpR_rhpS-Mutant_KB_R1_peak_95 | 1777 | 1998652 | 1999770 | - | overlapStart | 84 | 84 | NearestLocation |
| PSPPH_1805 | folE | GTP cyclohydrolase I | chromosome | 2109566 | 2109924 | 359 | * | rhpR_rhpS-Mutant_MM_R1_peak_62 | 1861 | 2109063 | 2109713 | - | overlapStart | 147 | 147 | NearestLocation |
| PSPPH_1888 |  | hypothetical protein | chromosome | 2190379 | 2190531 | 153 | * | rhpR_rhpS-Mutant_KB_R1_peak_102 | 1948 | 2190515 | 2190874 | + | overlapStart | -136 | 16 | NearestLocation |
| PSPPH_1897 |  | chorismate mutase | chromosome | 2201469 | 2201552 | 84 | * | rhpR_rhpS-Mutant_KB_R1_peak_103 | 1957 | 2200419 | 2201546 | - | overlapStart | 77 | 6 | NearestLocation |
| PSPPH_1903 |  | universal stress protein family protein | chromosome | 2208915 | 2209034 | 120 | * | rhpR_rhpS-Mutant_KB_R1_peak_104 | 1963 | 2208997 | 2209428 | + | overlapStart | -82 | 37 | NearestLocation |
| PSPPH_1970 |  | hypothetical protein | chromosome | 2318405 | 2318507 | 103 | * | rhpR_rhpS-Mutant_KB_R1_peak_105 | 2030 | 2318480 | 2319223 | + | overlapStart | -75 | 27 | NearestLocation |
| PSPPH_2005 |  | hypothetical protein | chromosome | 2353383 | 2353793 | 411 | * | rhpR_rhpS-Mutant_KB_R1_peak_111 | 2067 | 2353651 | 2354559 | + | overlapStart | -268 | 142 | NearestLocation |
| PSPPH_2119 |  | hypothetical protein | chromosome | 2478886 | 2479063 | 178 | * | rhpR_rhpS-Mutant_KB_R1_peak_116 | 2184 | 2478966 | 2479223 | + | overlapStart | -80 | 80 | NearestLocation |
| PSPPH_2590 |  | GAF domain/GGDEF domain/EAL domain-containing protein | chromosome | 2981695 | 2981823 | 129 | * | rhpR_rhpS-Mutant_KB_R1_peak_127 | 2675 | 2981819 | 2984701 | + | overlapStart | -124 | 4 | NearestLocation |
| PSPPH_2790 |  | prophage PSPPH05, helix-destabilizing protein | chromosome | 3229738 | 3229906 | 169 | * | rhpR_rhpS-Mutant_MM_R1_peak_92 | 2881 | 3229542 | 3229829 | - | overlapStart | 91 | 77 | NearestLocation |
| PSPPH_3057 |  | general secretion pathway protein GspL | chromosome | 3552803 | 3553026 | 224 | * | rhpR_rhpS-Mutant_MM_R1_peak_97 | 3159 | 3551788 | 3552885 | - | overlapStart | 82 | 82 | NearestLocation |
| PSPPH_3315 |  | hypothetical protein | chromosome | 3836471 | 3836875 | 405 | * | rhpR_rhpS-Mutant_KB_R1_peak_148 | 3428 | 3836826 | 3838463 | + | overlapStart | -355 | 49 | NearestLocation |
| PSPPH_3366 | flhA | flagellar biosynthesis protein FlhA | chromosome | 3896021 | 3896149 | 129 | * | rhpR_rhpS-Mutant_KB_R1_peak_150 | 3480 | 3893910 | 3896039 | - | overlapStart | 18 | 18 | NearestLocation |
| PSPPH_3372 | fliN | flagellar motor switch protein | chromosome | 3900477 | 3900564 | 88 | * | rhpR_rhpS-Mutant_KB_R1_peak_151 | 3486 | 3900030 | 3900488 | - | overlapStart | 11 | 11 | NearestLocation |
| PSPPH_3413 |  | chemotaxis protein CheV | chromosome | 3946831 | 3947014 | 184 | * | rhpR_rhpS-Mutant_MM_R1_peak_109 | 3527 | 3945912 | 3946838 | - | overlapStart | 7 | 7 | NearestLocation |
| PSPPH_3414 | flgA | flagellar basal body P-ring biosynthesis protein FlgA | chromosome | 3946854 | 3946995 | 142 | * | rhpR_rhpS-Mutant_KB_R1_peak_154 | 3528 | 3946917 | 3947675 | + | overlapStart | -63 | 63 | NearestLocation |
| PSPPH_3654 |  | TetR family transcriptional regulator | chromosome | 4197097 | 4197292 | 196 | * | rhpR_rhpS-Mutant_KB_R1_peak_160 | 3776 | 4197240 | 4197809 | + | overlapStart | -143 | 52 | NearestLocation |
| PSPPH_3845 |  | arsC family protein | chromosome | 4402109 | 4402433 | 325 | * | rhpR_rhpS-Mutant_MM_R1_peak_120 | 3967 | 4402378 | 4402725 | + | overlapStart | -269 | 55 | NearestLocation |
| PSPPH_3920 |  | hypothetical protein | chromosome | 4477578 | 4477681 | 104 | * | rhpR_rhpS-Mutant_KB_R1_peak_172 | 4044 | 4477670 | 4478281 | + | overlapStart | -92 | 11 | NearestLocation |
| PSPPH_4241 |  | LuxR family transcriptional regulator | chromosome | 4838356 | 4838557 | 202 | * | rhpR_rhpS-Mutant_KB_R1_peak_188 | 4379 | 4838479 | 4839108 | + | overlapStart | -123 | 78 | NearestLocation |
| PSPPH_4245 |  | GAF domain-containing protein | chromosome | 4842937 | 4843137 | 201 | * | rhpR_rhpS-Mutant_KB_R1_peak_189 | 4383 | 4843131 | 4843619 | + | overlapStart | -194 | 6 | NearestLocation |
| PSPPH_4246 |  | acetyltransferase | chromosome | 4844028 | 4844319 | 292 | * | rhpR_rhpS-Mutant_KB_R1_peak_190 | 4384 | 4843633 | 4844088 | - | overlapStart | 60 | 60 | NearestLocation |
| PSPPH_4247 |  | GAF domain/GGDEF domain/EAL domain-containing protein | chromosome | 4843994 | 4844361 | 368 | * | rhpR_rhpS-Mutant_MM_R1_peak_130 | 4385 | 4844348 | 4846210 | + | overlapStart | -354 | 13 | NearestLocation |
| PSPPH_4317 |  | hypothetical protein | chromosome | 4931251 | 4931381 | 131 | * | rhpR_rhpS-Mutant_KB_R1_peak_194 | 4455 | 4930056 | 4931276 | - | overlapStart | 25 | 25 | NearestLocation |
| PSPPH_4386 |  | hypothetical protein | chromosome | 5010424 | 5010501 | 78 | * | rhpR_rhpS-Mutant_KB_R1_peak_199 | 4526 | 5010033 | 5010425 | - | overlapStart | 1 | 1 | NearestLocation |
| PSPPH_4539 |  | transposon Tn7-like transposase B | chromosome | 5173255 | 5173387 | 133 | * | rhpR_rhpS-Mutant_KB_R1_peak_203 | 4679 | 5173332 | 5175449 | + | overlapStart | -77 | 55 | NearestLocation |
| PSPPH_4563 | uvrA | excinuclease ABC subunit A | chromosome | 5208539 | 5208618 | 80 | * | rhpR_rhpS-Mutant_KB_R1_peak_208 | 4705 | 5208582 | 5211416 | + | overlapStart | -43 | 36 | NearestLocation |
| PSPPH_4619 |  | beta alanine--pyruvate transaminase | chromosome | 5255741 | 5255869 | 129 | * | rhpR_rhpS-Mutant_KB_R1_peak_218 | 4761 | 5254425 | 5255771 | - | overlapStart | 30 | 30 | NearestLocation |
| PSPPH_4711 |  | hypothetical protein | chromosome | 5346973 | 5347394 | 422 | * | rhpR_rhpS-Mutant_KB_R1_peak_223 | 4854 | 5346762 | 5347043 | - | overlapStart | 70 | 70 | NearestLocation |
| PSPPH_4994 |  | levansucrase LscC | chromosome | 5663072 | 5663305 | 234 | * | rhpR_rhpS-Mutant_MM_R1_peak_149 | 5152 | 5663238 | 5664533 | + | overlapStart | -166 | 67 | NearestLocation |
| PSPPH_5069 |  | Rhs family protein | chromosome | 5748278 | 5748637 | 360 | * | rhpR_rhpS-Mutant_KB_R1_peak_235 | 5229 | 5748637 | 5750496 | + | overlapStart | -359 | 0 | NearestLocation |
| PSPPH_1119 |  | hypothetical protein | chromosome | 1319322 | 1319434 | 113 | * | rhpR_rhpS-Mutant_KB_R1_peak_73 | 1149 | 1319388 | 1320167 | - | overlapEnd | 845 | 46 | NearestLocation |
| PSPPH_3123 |  | alpha/beta hydrolase | chromosome | 3624985 | 3625141 | 157 | * | rhpR_rhpS-Mutant_KB_R1_peak_144 | 3230 | 3624094 | 3625017 | + | overlapEnd | 891 | 32 | NearestLocation |
| PSPPH_4453 |  | hypothetical protein | chromosome | 5087822 | 5088020 | 199 | * | rhpR_rhpS-Mutant_KB_R1_peak_201 | 4593 | 5087883 | 5088518 | - | overlapEnd | 696 | 61 | NearestLocation |
| PSPPH_0102 |  | ParB-like nuclease | chromosome | 113897 | 114014 | 118 | * | rhpR_rhpS-Mutant_KB_R1_peak_5 | 105 | 113614 | 114579 | + | inside | 283 | 283 | NearestLocation |
| PSPPH_0117 |  | phospholipase D | chromosome | 135146 | 135317 | 172 | * | rhpR_rhpS-Mutant_KB_R1_peak_6 | 120 | 134132 | 136063 | - | inside | 917 | 746 | NearestLocation |
| PSPPH_0165 |  | hypothetical protein | chromosome | 189781 | 189863 | 83 | * | rhpR_rhpS-Mutant_KB_R1_peak_9 | 171 | 189713 | 190558 | + | inside | 68 | 68 | NearestLocation |
| PSPPH_0171 | hopR1 | type III effector HopR1 | chromosome | 204253 | 204433 | 181 | * | rhpR_rhpS-Mutant_KB_R1_peak_10 | 178 | 198685 | 204564 | - | inside | 311 | 131 | NearestLocation |
| PSPPH_0177 |  | LysR family transcriptional regulator | chromosome | 211126 | 211231 | 106 | * | rhpR_rhpS-Mutant_KB_R1_peak_12 | 186 | 210746 | 211510 | + | inside | 380 | 279 | NearestLocation |
| PSPPH_0253 |  | sensory box histidine kinase | chromosome | 292687 | 292907 | 221 | * | rhpR_rhpS-Mutant_MM_R1_peak_7 | 263 | 292304 | 294091 | + | inside | 383 | 383 | NearestLocation |
| PSPPH_0295 |  | hypothetical protein | chromosome | 338961 | 339042 | 82 | * | rhpR_rhpS-Mutant_MM_R1_peak_11 | 305 | 338360 | 339097 | - | inside | 136 | 55 | NearestLocation |
| PSPPH_0737 | pilS | type IV pilus-associated protein | chromosome | 864149 | 864236 | 88 | * | rhpR_rhpS-Mutant_KB_R1_peak_34 | 753 | 863034 | 864626 | - | inside | 477 | 390 | NearestLocation |
| PSPPH_0766 |  | pseudogene | chromosome | 896782 | 896896 | 115 | * | rhpR_rhpS-Mutant_KB_R1_peak_40 | 784 | 896731 | 896976 | - | inside | 194 | 51 | NearestLocation |
| PSPPH_0770 |  | response regulator/sensor histidine kinase | chromosome | 902857 | 902957 | 101 | * | rhpR_rhpS-Mutant_KB_R1_peak_43 | 789 | 901688 | 903976 | + | inside | 1169 | 1019 | NearestLocation |
| PSPPH_0775 |  | periplasmic chaperone protein | chromosome | 910538 | 910733 | 196 | * | rhpR_rhpS-Mutant_KB_R1_peak_45 | 795 | 910262 | 910975 | - | inside | 437 | 242 | NearestLocation |
| PSPPH_0956 |  | WbbD | chromosome | 1135396 | 1135537 | 142 | * | rhpR_rhpS-Mutant_MM_R1_peak_38 | 983 | 1135374 | 1136954 | + | inside | 22 | 22 | NearestLocation |
| PSPPH_0958 |  | CDP-glucose-4,6-dehydratase | chromosome | 1140057 | 1140200 | 144 | * | rhpR_rhpS-Mutant_KB_R1_peak_59 | 985 | 1139722 | 1140804 | + | inside | 335 | 335 | NearestLocation |
| PSPPH_0965 |  | hypothetical protein | chromosome | 1148355 | 1148461 | 107 | * | rhpR_rhpS-Mutant_KB_R1_peak_60 | 992 | 1147760 | 1149175 | - | inside | 820 | 595 | NearestLocation |
| PSPPH_1368 |  | YaeQ family protein | chromosome | 1587512 | 1587650 | 139 | * | rhpR_rhpS-Mutant_KB_R1_peak_85 | 1409 | 1587441 | 1588172 | + | inside | 71 | 71 | NearestLocation |
| PSPPH_1700 | lon1 | ATP-dependent protease La | chromosome | 1965519 | 1965695 | 177 | * | rhpR_rhpS-Mutant_MM_R1_peak_58 | 1752 | 1964447 | 1966843 | + | inside | 1072 | 1072 | NearestLocation |
| PSPPH_1780 |  | hypothetical protein | chromosome | 2084996 | 2085081 | 86 | * | rhpR_rhpS-Mutant_KB_R1_peak_96 | 1835 | 2084780 | 2085943 | + | inside | 216 | 216 | NearestLocation |
| PSPPH_1976 | sdhC | succinate dehydrogenase, cytochrome b556 subunit | chromosome | 2322026 | 2322136 | 111 | * | rhpR_rhpS-Mutant_KB_R1_peak_106 | 2036 | 2321961 | 2322335 | + | inside | 65 | 65 | NearestLocation |
| PSPPH_2159 |  | isocitrate/isopropylmalate family dehydrogenase | chromosome | 2515238 | 2515337 | 100 | * | rhpR_rhpS-Mutant_KB_R1_peak_119 | 2225 | 2514967 | 2516157 | + | inside | 271 | 271 | NearestLocation |
| PSPPH_2496 |  | NAD-dependent deacetylase | chromosome | 2884092 | 2884201 | 110 | * | rhpR_rhpS-Mutant_MM_R1_peak_83 | 2579 | 2883466 | 2884311 | - | inside | 219 | 110 | NearestLocation |
| PSPPH_2619 |  | sugar-binding domain-containing protein | chromosome | 3018915 | 3018990 | 76 | * | rhpR_rhpS-Mutant_KB_R1_peak_129 | 2705 | 3018875 | 3019948 | + | inside | 40 | 40 | NearestLocation |
| PSPPH_2787 |  | dipeptide/oligopeptide/nickel ABC transporter ATPase | chromosome | 3225874 | 3225954 | 81 | * | rhpR_rhpS-Mutant_KB_R1_peak_136 | 2878 | 3225566 | 3225958 | - | inside | 84 | 4 | NearestLocation |
| PSPPH_2791 |  | hypothetical protein | chromosome | 3231118 | 3231236 | 119 | * | rhpR_rhpS-Mutant_KB_R1_peak_137 | 2882 | 3230334 | 3231464 | - | inside | 346 | 228 | NearestLocation |
| PSPPH_3079 |  | LysR family transcriptional regulator | chromosome | 3577416 | 3577497 | 82 | * | rhpR_rhpS-Mutant_MM_R1_peak_99 | 3185 | 3576871 | 3577779 | + | inside | 545 | 282 | NearestLocation |
| PSPPH_3759 |  | zinc-binding oxidoreductase | chromosome | 4311480 | 4311669 | 190 | * | rhpR_rhpS-Mutant_MM_R1_peak_118 | 3881 | 4310952 | 4311974 | - | inside | 494 | 305 | NearestLocation |
| PSPPH_3986 |  | hypothetical protein | chromosome | 4552014 | 4552137 | 124 | * | rhpR_rhpS-Mutant_KB_R1_peak_175 | 4112 | 4551605 | 4552324 | - | inside | 310 | 187 | NearestLocation |
| PSPPH_4102 | lpxC | UDP-3-O-[3-hydroxymyristoyl] N-acetylglucosamine deacetylase | chromosome | 4687286 | 4687364 | 79 | * | rhpR_rhpS-Mutant_KB_R1_peak_181 | 4239 | 4686624 | 4687535 | - | inside | 249 | 171 | NearestLocation |
| PSPPH_4249 |  | hypothetical protein | chromosome | 4848241 | 4848319 | 79 | * | rhpR_rhpS-Mutant_MM_R1_peak_131 | 4387 | 4848143 | 4848382 | - | inside | 141 | 63 | NearestLocation |
| PSPPH_4418 |  | type IV leader peptidase | chromosome | 5043765 | 5043848 | 84 | * | rhpR_rhpS-Mutant_KB_R1_peak_200 | 4558 | 5043760 | 5044233 | + | inside | 5 | 5 | NearestLocation |
| PSPPH_4541 |  | hypothetical protein | chromosome | 5177196 | 5177331 | 136 | * | rhpR_rhpS-Mutant_KB_R1_peak_204 | 4682 | 5176817 | 5177851 | + | inside | 379 | 379 | NearestLocation |
| PSPPH_4544 |  | hypothetical protein | chromosome | 5180056 | 5180156 | 101 | * | rhpR_rhpS-Mutant_KB_R1_peak_205 | 4685 | 5179499 | 5180248 | - | inside | 192 | 92 | NearestLocation |
| PSPPH_4605 | secE | preprotein translocase subunit SecE | chromosome | 5243799 | 5243918 | 120 | * | rhpR_rhpS-Mutant_KB_R1_peak_214 | 4747 | 5243620 | 5243988 | - | inside | 189 | 70 | NearestLocation |
| PSPPH_4682 |  | lipopolysaccharide biosynthesis protein | chromosome | 5317891 | 5318014 | 124 | * | rhpR_rhpS-Mutant_KB_R1_peak_219 | 4824 | 5317528 | 5318289 | - | inside | 398 | 275 | NearestLocation |
| PSPPH_4977 |  | prophage PSPPH06, GNAT family acetyltransferase | chromosome | 5646267 | 5646440 | 174 | * | rhpR_rhpS-Mutant_KB_R1_peak_229 | 5135 | 5646120 | 5646566 | + | inside | 147 | 126 | NearestLocation |
| PSPPH_5204 |  | DeoR family transcriptional regulator | chromosome | 5908987 | 5909081 | 95 | * | rhpR_rhpS-Mutant_MM_R1_peak_154 | 5370 | 5908358 | 5909125 | - | inside | 138 | 44 | NearestLocation |
| PSPPH_1688 |  | tRNA-Arg | chromosome | 1952539 | 1952723 | 185 | * | rhpR_rhpS-Mutant_MM_R1_peak_56 | 1740 | 1952561 | 1952637 | + | includeFeature | -22 | 22 | NearestLocation |
| PSPPH_0172 |  | pseudogene | chromosome | 206405 | 206497 | 93 | * | rhpR_rhpS-Mutant_KB_R1_peak_11 | 180 | 206633 | 206890 | - | downstream | 485 | 136 | NearestLocation |
| PSPPH_0816 |  | zinc-binding protein | chromosome | 976350 | 976443 | 94 | * | rhpR_rhpS-Mutant_KB_R1_peak_49 | 839 | 977780 | 977977 | - | downstream | 1627 | 1337 | NearestLocation |
| PSPPH_0966 |  | ISPsy23, transposition helper protein | chromosome | 1150013 | 1150177 | 165 | * | rhpR_rhpS-Mutant_KB_R1_peak_61 | 993 | 1149304 | 1149723 | + | downstream | 709 | 290 | NearestLocation |
| PSPPH_1077 |  | hypothetical protein | chromosome | 1267155 | 1267244 | 90 | * | rhpR_rhpS-Mutant_KB_R1_peak_68 | 1105 | 1266510 | 1267055 | + | downstream | 645 | 100 | NearestLocation |
| PSPPH_1084 |  | hypothetical protein | chromosome | 1277248 | 1277412 | 165 | * | rhpR_rhpS-Mutant_KB_R1_peak_69 | 1112 | 1277491 | 1277757 | - | downstream | 509 | 79 | NearestLocation |
| PSPPH_1373 |  | hypothetical protein | chromosome | 1594548 | 1594669 | 122 | * | rhpR_rhpS-Mutant_KB_R1_peak_86 | 1414 | 1595303 | 1595491 | - | downstream | 943 | 634 | NearestLocation |
| PSPPH_1375 |  | hypothetical protein | chromosome | 1596455 | 1596541 | 87 | * | rhpR_rhpS-Mutant_KB_R1_peak_87 | 1416 | 1596630 | 1596812 | - | downstream | 357 | 89 | NearestLocation |
| PSPPH_1694 |  | hypothetical protein | chromosome | 1957045 | 1957149 | 105 | * | rhpR_rhpS-Mutant_KB_R1_peak_92 | 1746 | 1957205 | 1958197 | - | downstream | 1152 | 56 | NearestLocation |
| PSPPH_2075 | nasT | response regulator NasT | chromosome | 2434285 | 2434361 | 77 | * | rhpR_rhpS-Mutant_KB_R1_peak_113 | 2140 | 2434443 | 2435018 | - | downstream | 733 | 82 | NearestLocation |
| PSPPH_3122 |  | transcriptional regulator | chromosome | 3622741 | 3622833 | 93 | * | rhpR_rhpS-Mutant_MM_R1_peak_100 | 3229 | 3623017 | 3624006 | - | downstream | 1265 | 184 | NearestLocation |
| PSPPH_4025 |  | transcriptional regulator | chromosome | 4600672 | 4600828 | 157 | * | rhpR_rhpS-Mutant_KB_R1_peak_178 | 4154 | 4600070 | 4600450 | + | downstream | 602 | 222 | NearestLocation |
| PSPPH_4041 |  | fumarate hydratase, class I | chromosome | 4617421 | 4617563 | 143 | * | rhpR_rhpS-Mutant_KB_R1_peak_180 | 4172 | 4615638 | 4617161 | + | downstream | 1783 | 260 | NearestLocation |
| PSPPH_4042 |  | insecticidal toxin protein | chromosome | 4617415 | 4617571 | 157 | * | rhpR_rhpS-Mutant_MM_R1_peak_127 | 4173 | 4617999 | 4619348 | - | downstream | 1933 | 428 | NearestLocation |
| PSPPH_4321 |  | hypothetical protein | chromosome | 4935714 | 4935812 | 99 | * | rhpR_rhpS-Mutant_KB_R1_peak_195 | 4459 | 4936398 | 4937021 | - | downstream | 1307 | 586 | NearestLocation |
| PSPPH_4363 |  | hypothetical protein | chromosome | 4985769 | 4985899 | 131 | * | rhpR_rhpS-Mutant_KB_R1_peak_197 | 4503 | 4985442 | 4985747 | + | downstream | 327 | 22 | NearestLocation |
| PSPPH_4558 |  | MutT domain-containing protein | chromosome | 5197745 | 5197821 | 77 | * | rhpR_rhpS-Mutant_KB_R1_peak_206 | 4699 | 5197852 | 5198625 | - | downstream | 880 | 31 | NearestLocation |

**H.** **KB-dependent RhpR binding sites**

| gene_id | name | Product Name | seqnames | start | end | width | strand | peak | feature | start_position | end_position | feature_strand | insideFeature | distancetoFeature | shortestDistance | fromOverlappingOrNearest |
| --- | --- | --- | --- | --- | --- | --- | --- | --- | --- | --- | --- | --- | --- | --- | --- | --- |
| PSPPH_0001 | dnaA | chromosome replication initiator DnaA | chromosome | 148 | 227 | 80 | * | rhpR_rhpS-Mutant_KB_R1_peak_1 | 1 | 238 | 1773 | + | upstream | -90 | 11 | NearestLocation |
| PSPPH_0008 |  | oxidoreductase, alpha (molybdopterin) subunit, fusion | chromosome | 12552 | 12680 | 129 | * | rhpR_KB_R1_peak_1 | 9 | 11095 | 12453 | - | upstream | -99 | 99 | NearestLocation |
| PSPPH_0140 | fdhD | formate dehydrogenase accessory protein FdhD | chromosome | 167840 | 167958 | 119 | * | rhpR_KB_R1_peak_2 | 145 | 166900 | 167736 | - | upstream | -104 | 104 | NearestLocation |
| PSPPH_0155 |  | OprD family outer membrane porin | chromosome | 182777 | 182859 | 83 | * | rhpR_rhpS-Mutant_KB_R1_peak_8 | 161 | 182883 | 184157 | + | upstream | -106 | 24 | NearestLocation |
| PSPPH_0179 |  | ATPase AAA | chromosome | 212804 | 212928 | 125 | * | rhpR_rhpS-Mutant_KB_R1_peak_13 | 188 | 212966 | 214093 | + | upstream | -162 | 38 | NearestLocation |
| PSPPH_0487 |  | Na+/proline symporter | chromosome | 554950 | 555028 | 79 | * | rhpR_rhpS-Mutant_KB_R1_peak_19 | 499 | 555043 | 556275 | + | upstream | -93 | 15 | NearestLocation |
| PSPPH_0548 |  | lipoprotein | chromosome | 642359 | 642471 | 113 | * | rhpR_rhpS-Mutant_KB_R1_peak_21 | 561 | 642501 | 643199 | + | upstream | -142 | 30 | NearestLocation |
| PSPPH_0611 |  | hypothetical protein | chromosome | 721894 | 721985 | 92 | * | rhpR_rhpS-Mutant_KB_R1_peak_23 | 624 | 722115 | 723011 | + | upstream | -221 | 130 | NearestLocation |
| PSPPH_0629 |  | protein kinase | chromosome | 746546 | 746623 | 78 | * | rhpR_rhpS-Mutant_KB_R1_peak_28 | 643 | 744516 | 746438 | - | upstream | -108 | 108 | NearestLocation |
| PSPPH_0667 |  | prophage PSPPH01, site-specific recombinase phage integrase | chromosome | 782730 | 782833 | 104 | * | rhpR_rhpS-Mutant_KB_R1_peak_31 | 682 | 783191 | 784366 | + | upstream | -461 | 358 | NearestLocation |
| PSPPH_0672 | trpG | anthranilate synthase component II | chromosome | 788446 | 788661 | 216 | * | rhpR_rhpS-Mutant_KB_R1_peak_32 | 687 | 788757 | 789356 | + | upstream | -311 | 96 | NearestLocation |
| PSPPH_0731 |  | type IV pilus biogenesis protein | chromosome | 854677 | 854756 | 80 | * | rhpR_rhpS-Mutant_KB_R1_peak_33 | 747 | 854922 | 855326 | + | upstream | -245 | 166 | NearestLocation |
| PSPPH_0755 |  | TetR family transcriptional regulator | chromosome | 882168 | 882259 | 92 | * | rhpR_KB_R1_peak_6 | 772 | 882507 | 883145 | + | upstream | -339 | 248 | NearestLocation |
| PSPPH_0778 |  | LuxR family transcriptional regulator | chromosome | 914183 | 914262 | 80 | * | rhpR_rhpS-Mutant_KB_R1_peak_46 | 798 | 914293 | 914931 | + | upstream | -110 | 31 | NearestLocation |
| PSPPH_0868 |  | penicillin-binding protein 1B | chromosome | 1037873 | 1037980 | 108 | * | rhpR_rhpS-Mutant_KB_R1_peak_52 | 892 | 1038016 | 1040337 | + | upstream | -143 | 36 | NearestLocation |
| PSPPH_0871 |  | hypothetical protein | chromosome | 1041956 | 1042072 | 117 | * | rhpR_rhpS-Mutant_KB_R1_peak_53 | 895 | 1041434 | 1041850 | - | upstream | -106 | 106 | NearestLocation |
| PSPPH_0953 | gmd | GDP-mannose 4,6-dehydratase | chromosome | 1132895 | 1132974 | 80 | * | rhpR_rhpS-Mutant_KB_R1_peak_57 | 980 | 1131805 | 1132836 | - | upstream | -59 | 59 | NearestLocation |
| PSPPH_1090 |  | ribosomal subunit interface protein | chromosome | 1284131 | 1284212 | 82 | * | rhpR_rhpS-Mutant_KB_R1_peak_71 | 1119 | 1283687 | 1284115 | - | upstream | -16 | 16 | NearestLocation |
| PSPPH_1146 |  | TetR family transcriptional regulator | chromosome | 1345960 | 1346106 | 147 | * | rhpR_KB_R1_peak_8 | 1176 | 1345074 | 1345496 | - | upstream | -464 | 464 | NearestLocation |
| PSPPH_1147 |  | pseudogene | chromosome | 1347960 | 1348101 | 142 | * | rhpR_rhpS-Mutant_KB_R1_peak_76 | 1177 | 1345493 | 1347374 | - | upstream | -586 | 586 | NearestLocation |
| PSPPH_1160 |  | cold shock domain-contain protein | chromosome | 1362263 | 1362386 | 124 | * | rhpR_KB_R1_peak_10 | 1191 | 1361947 | 1362159 | - | upstream | -104 | 104 | NearestLocation |
| PSPPH_1207 |  | chemotaxis protein CheV | chromosome | 1418087 | 1418166 | 80 | * | rhpR_KB_R1_peak_11 | 1239 | 1417026 | 1417922 | - | upstream | -165 | 165 | NearestLocation |
| PSPPH_1210 | cyoA | cytochrome o ubiquinol oxidase subunit II | chromosome | 1419220 | 1419414 | 195 | * | rhpR_rhpS-Mutant_KB_R1_peak_82 | 1242 | 1419508 | 1420449 | + | upstream | -288 | 94 | NearestLocation |
| PSPPH_1333 | guaB | inosine 5'-monophosphate dehydrogenase | chromosome | 1543564 | 1543650 | 87 | * | rhpR_rhpS-Mutant_KB_R1_peak_84 | 1373 | 1543651 | 1545120 | + | upstream | -87 | 1 | NearestLocation |
| PSPPH_1465 |  | hypothetical protein | chromosome | 1708842 | 1708949 | 108 | * | rhpR_KB_R1_peak_13 | 1508 | 1708032 | 1708760 | - | upstream | -82 | 82 | NearestLocation |
| PSPPH_1485 |  | major facilitator family transporter | chromosome | 1725007 | 1725123 | 117 | * | rhpR_KB_R1_peak_14 | 1528 | 1726042 | 1727358 | + | upstream | -1035 | 919 | NearestLocation |
| PSPPH_1669 | ilvA1 | threonine dehydratase | chromosome | 1930921 | 1931076 | 156 | * | rhpR_rhpS-Mutant_KB_R1_peak_90 | 1721 | 1929241 | 1930776 | - | upstream | -145 | 145 | NearestLocation |
| PSPPH_1698 | clpP | ATP-dependent Clp protease proteolytic subunit | chromosome | 1961958 | 1962055 | 98 | * | rhpR_rhpS-Mutant_KB_R1_peak_93 | 1750 | 1962205 | 1962846 | + | upstream | -247 | 150 | NearestLocation |
| PSPPH_1771 |  | GntR family transcriptional regulator | chromosome | 2068256 | 2068398 | 143 | * | rhpR_KB_R1_peak_16 | 1826 | 2068454 | 2069206 | + | upstream | -198 | 56 | NearestLocation |
| PSPPH_1827 |  | hypothetical protein | chromosome | 2127268 | 2127386 | 119 | * | rhpR_rhpS-Mutant_KB_R1_peak_99 | 1883 | 2127447 | 2127788 | + | upstream | -179 | 61 | NearestLocation |
| PSPPH_1868 |  | anti-sigma factor antagonist | chromosome | 2168329 | 2168430 | 102 | * | rhpR_rhpS-Mutant_KB_R1_peak_101 | 1925 | 2168726 | 2169211 | + | upstream | -397 | 296 | NearestLocation |
| PSPPH_2068 |  | OprF | chromosome | 2422857 | 2423039 | 183 | * | rhpR_rhpS-Mutant_KB_R1_peak_112 | 2133 | 2423246 | 2424280 | + | upstream | -389 | 207 | NearestLocation |
| PSPPH_2185 |  | sensor histidine kinase | chromosome | 2540838 | 2540948 | 111 | * | rhpR_rhpS-Mutant_KB_R1_peak_120 | 2255 | 2539472 | 2540776 | - | upstream | -62 | 62 | NearestLocation |
| PSPPH_2545 | mqo2 | malate:quinone oxidoreductase | chromosome | 2936627 | 2937000 | 374 | * | rhpR_KB_R1_peak_20 | 2630 | 2934900 | 2936543 | - | upstream | -84 | 84 | NearestLocation |
| PSPPH_2825 |  | hypothetical protein | chromosome | 3271715 | 3271827 | 113 | * | rhpR_rhpS-Mutant_KB_R1_peak_139 | 2919 | 3272037 | 3272318 | + | upstream | -322 | 210 | NearestLocation |
| PSPPH_3098 | clpS | ATP-dependent Clp protease adaptor protein ClpS | chromosome | 3596205 | 3596300 | 96 | * | rhpR_rhpS-Mutant_KB_R1_peak_143 | 3205 | 3595796 | 3596158 | - | upstream | -47 | 47 | NearestLocation |
| PSPPH_3128 |  | peptidase propeptide/YPEB domain-containing protein | chromosome | 3630510 | 3630590 | 81 | * | rhpR_rhpS-Mutant_KB_R1_peak_145 | 3235 | 3630124 | 3630432 | - | upstream | -78 | 78 | NearestLocation |
| PSPPH_3179 |  | hypothetical protein | chromosome | 3686427 | 3686568 | 142 | * | rhpR_KB_R1_peak_21 | 3289 | 3686569 | 3686865 | + | upstream | -142 | 1 | NearestLocation |
| PSPPH_3221 |  | lipid kinase | chromosome | 3733190 | 3733274 | 85 | * | rhpR_rhpS-Mutant_KB_R1_peak_147 | 3333 | 3732095 | 3733012 | - | upstream | -178 | 178 | NearestLocation |
| PSPPH_3386 | fleS | flagellar sensor histidine kinase FleS | chromosome | 3915619 | 3915713 | 95 | * | rhpR_KB_R1_peak_24 | 3500 | 3913997 | 3915211 | - | upstream | -408 | 408 | NearestLocation |
| PSPPH_3504 |  | alginate biosynthesis transcriptional activator | Add | 4043157 | 4043243 | 87 | * | rhpR_rhpS-Mutant_KB_R1_peak_157 | 3624 | 4043310 | 4043636 | + | upstream | -153 | 67 | NearestLocation |
| PSPPH_3529 |  | hypothetical protein | chromosome | 4069073 | 4069151 | 79 | * | rhpR_rhpS-Mutant_KB_R1_peak_158 | 3649 | 4069214 | 4069480 | + | upstream | -141 | 63 | NearestLocation |
| PSPPH_3674 |  | hypothetical protein | chromosome | 4222387 | 4222468 | 82 | * | rhpR_KB_R1_peak_25 | 3796 | 4221051 | 4221836 | - | upstream | -551 | 551 | NearestLocation |
| PSPPH_3809 | rpoS | RNA polymerase sigma factor RpoS | chromosome | 4362421 | 4362591 | 171 | * | rhpR_rhpS-Mutant_KB_R1_peak_169 | 3931 | 4361212 | 4362219 | - | upstream | -202 | 202 | NearestLocation |
| PSPPH_3810 |  | lipoprotein | chromosome | 4363270 | 4363352 | 83 | * | rhpR_rhpS-Mutant_KB_R1_peak_170 | 3932 | 4362323 | 4363189 | - | upstream | -81 | 81 | NearestLocation |
| PSPPH_4007 |  | hypothetical protein | chromosome | 4578378 | 4578534 | 157 | * | rhpR_rhpS-Mutant_KB_R1_peak_177 | 4136 | 4578772 | 4579128 | + | upstream | -394 | 238 | NearestLocation |
| PSPPH_4021 |  | hypothetical protein | chromosome | 4596375 | 4596483 | 109 | * | rhpR_KB_R1_peak_31 | 4150 | 4596548 | 4596856 | + | upstream | -173 | 65 | NearestLocation |
| PSPPH_4152 | yfiA | ribosomal subunit interface protein | chromosome | 4736463 | 4736554 | 92 | * | rhpR_KB_R1_peak_32 | 4289 | 4736613 | 4736921 | + | upstream | -150 | 59 | NearestLocation |
| PSPPH_4173 | gatC | aspartyl/glutamyl-tRNA amidotransferase subunit C | chromosome | 4758236 | 4758321 | 86 | * | rhpR_rhpS-Mutant_KB_R1_peak_183 | 4310 | 4758378 | 4758665 | + | upstream | -142 | 57 | NearestLocation |
| PSPPH_4198 | ftsH | ATP-dependent metalloprotease FtsH | chromosome | 4786421 | 4786504 | 84 | * | rhpR_rhpS-Mutant_KB_R1_peak_185 | 4335 | 4784486 | 4786399 | - | upstream | -22 | 22 | NearestLocation |
| PSPPH_4298 |  | ISPsy5, transposase truncated | chromosome | 4909030 | 4909143 | 114 | * | rhpR_KB_R1_peak_34 | 4436 | 4907552 | 4908361 | - | upstream | -669 | 669 | NearestLocation |
| PSPPH_4373 |  | hypothetical protein | chromosome | 4994751 | 4994864 | 114 | * | rhpR_rhpS-Mutant_KB_R1_peak_198 | 4513 | 4995109 | 4995801 | + | upstream | -358 | 245 | NearestLocation |
| PSPPH_4518 |  | OmpW family outer membrane protein | chromosome | 5148602 | 5148706 | 105 | * | rhpR_KB_R1_peak_35 | 4658 | 5148798 | 5149484 | + | upstream | -196 | 92 | NearestLocation |
| PSPPH_4538 |  | transposon Tn7-like transposase A | chromosome | 5172389 | 5172489 | 101 | * | rhpR_KB_R1_peak_37 | 4678 | 5172524 | 5173330 | + | upstream | -135 | 35 | NearestLocation |
| PSPPH_4578 | rpsH | 30S ribosomal protein S8 | chromosome | 5220685 | 5220791 | 107 | * | rhpR_rhpS-Mutant_KB_R1_peak_210 | 4720 | 5220254 | 5220646 | - | upstream | -39 | 39 | NearestLocation |
| PSPPH_4598 | rpoC | DNA-directed RNA polymerase subunit beta' | chromosome | 5236475 | 5236580 | 106 | * | rhpR_rhpS-Mutant_KB_R1_peak_212 | 4740 | 5232126 | 5236325 | - | upstream | -150 | 150 | NearestLocation |
| PSPPH_4609 |  | tRNA-Tyr | chromosome | 5244565 | 5244674 | 110 | * | rhpR_rhpS-Mutant_KB_R1_peak_217 | 4751 | 5244476 | 5244560 | - | upstream | -5 | 5 | NearestLocation |
| PSPPH_4834 |  | Rhs family protein | chromosome | 5492503 | 5492581 | 79 | * | rhpR_KB_R1_peak_38 | 4983 | 5493584 | 5494279 | + | upstream | -1081 | 1003 | NearestLocation |
| PSPPH_4885 | serA | D-3-phosphoglycerate dehydrogenase | chromosome | 5554960 | 5555053 | 94 | * | rhpR_rhpS-Mutant_KB_R1_peak_225 | 5039 | 5555056 | 5556345 | + | upstream | -96 | 3 | NearestLocation |
| PSPPH_4948 |  | hypothetical protein | chromosome | 5621316 | 5621452 | 137 | * | rhpR_rhpS-Mutant_KB_R1_peak_227 | 5104 | 5621094 | 5621216 | - | upstream | -100 | 100 | NearestLocation |
| PSPPH_4981 |  | hypothetical protein | chromosome | 5652314 | 5652396 | 83 | * | rhpR_rhpS-Mutant_KB_R1_peak_230 | 5139 | 5651658 | 5652302 | - | upstream | -12 | 12 | NearestLocation |
| PSPPH_5016 | gltP | glutamate/aspartate:proton symporter | chromosome | 5684153 | 5684242 | 90 | * | rhpR_rhpS-Mutant_KB_R1_peak_231 | 5174 | 5682626 | 5683957 | - | upstream | -196 | 196 | NearestLocation |
| PSPPH_5071 |  | hypothetical protein | chromosome | 5750970 | 5751063 | 94 | * | rhpR_rhpS-Mutant_KB_R1_peak_236 | 5231 | 5751110 | 5751574 | + | upstream | -140 | 47 | NearestLocation |
| PSPPH_5082 |  | mechanosensitive ion channel protein MscS | chromosome | 5764621 | 5764746 | 126 | * | rhpR_rhpS-Mutant_KB_R1_peak_237 | 5243 | 5764856 | 5765668 | + | upstream | -235 | 110 | NearestLocation |
| PSPPH_5202 |  | ISPsy18, transposase | chromosome | 5903129 | 5903235 | 107 | * | rhpR_KB_R1_peak_39 | 5368 | 5903367 | 5904626 | + | upstream | -238 | 132 | NearestLocation |
| PSPPH_0138 |  | hypothetical protein | chromosome | 164372 | 164456 | 85 | * | rhpR_rhpS-Mutant_KB_R1_peak_7 | 143 | 163896 | 164447 | - | overlapStart | 75 | 9 | NearestLocation |
| PSPPH_0198 | rpoZ | DNA-directed RNA polymerase subunit omega | chromosome | 236856 | 236972 | 117 | * | rhpR_KB_R1_peak_3 | 208 | 236596 | 236937 | - | overlapStart | 81 | 35 | NearestLocation |
| PSPPH_0279 | hemB | delta-aminolevulinic acid dehydratase | chromosome | 320874 | 321048 | 175 | * | rhpR_KB_R1_peak_4 | 289 | 321012 | 322022 | + | overlapStart | -138 | 36 | NearestLocation |
| PSPPH_0476 |  | hypothetical protein | chromosome | 539619 | 539721 | 103 | * | rhpR_rhpS-Mutant_KB_R1_peak_18 | 488 | 539049 | 539621 | - | overlapStart | 2 | 2 | NearestLocation |
| PSPPH_0619 | rpoD | RNA polymerase sigma factor RpoD | chromosome | 734508 | 734835 | 328 | * | rhpR_rhpS-Mutant_KB_R1_peak_25 | 633 | 732770 | 734620 | - | overlapStart | 112 | 112 | NearestLocation |
| PSPPH_0628 |  | hypothetical protein | chromosome | 744385 | 744465 | 81 | * | rhpR_rhpS-Mutant_KB_R1_peak_27 | 642 | 743123 | 744394 | - | overlapStart | 9 | 9 | NearestLocation |
| PSPPH_0991 | prsA | ribose-phosphate pyrophosphokinase | chromosome | 1186093 | 1186271 | 179 | * | rhpR_rhpS-Mutant_KB_R1_peak_63 | 1018 | 1185154 | 1186095 | - | overlapStart | 2 | 2 | NearestLocation |
| PSPPH_0996 | hemA | glutamyl-tRNA reductase | chromosome | 1189588 | 1189724 | 137 | * | rhpR_rhpS-Mutant_KB_R1_peak_64 | 1023 | 1189698 | 1190975 | + | overlapStart | -110 | 26 | NearestLocation |
| PSPPH_1149 |  | phage integrase site specific recombinase | chromosome | 1350179 | 1350347 | 169 | * | rhpR_rhpS-Mutant_KB_R1_peak_77 | 1180 | 1349050 | 1350198 | - | overlapStart | 19 | 19 | NearestLocation |
| PSPPH_1157 | pepA | leucyl aminopeptidase | chromosome | 1359366 | 1359467 | 102 | * | rhpR_KB_R1_peak_9 | 1188 | 1357817 | 1359421 | - | overlapStart | 55 | 46 | NearestLocation |
| PSPPH_1689 |  | tRNA-His | chromosome | 1952459 | 1952751 | 293 | * | rhpR_rhpS-Mutant_KB_R1_peak_91 | 1741 | 1952689 | 1952764 | + | overlapStart | -230 | 13 | NearestLocation |
| PSPPH_1897 |  | chorismate mutase | chromosome | 2201469 | 2201552 | 84 | * | rhpR_rhpS-Mutant_KB_R1_peak_103 | 1957 | 2200419 | 2201546 | - | overlapStart | 77 | 6 | NearestLocation |
| PSPPH_1903 |  | universal stress protein family protein | Add | 2208915 | 2209034 | 120 | * | rhpR_rhpS-Mutant_KB_R1_peak_104 | 1963 | 2208997 | 2209428 | + | overlapStart | -82 | 37 | NearestLocation |
| PSPPH_2005 |  | hypothetical protein | chromosome | 2353383 | 2353793 | 411 | * | rhpR_rhpS-Mutant_KB_R1_peak_111 | 2067 | 2353651 | 2354559 | + | overlapStart | -268 | 142 | NearestLocation |
| PSPPH_2141 | ihfA | integration host factor subunit alpha | chromosome | 2497735 | 2497817 | 83 | * | rhpR_KB_R1_peak_18 | 2206 | 2497808 | 2498110 | + | overlapStart | -73 | 9 | NearestLocation |
| PSPPH_2220 |  | indolepyruvate ferredoxin oxidoreductase | chromosome | 2575966 | 2576065 | 100 | * | rhpR_rhpS-Mutant_KB_R1_peak_124 | 2292 | 2576054 | 2579539 | + | overlapStart | -88 | 11 | NearestLocation |
| PSPPH_2731 |  | hypothetical protein | chromosome | 3163031 | 3163178 | 148 | * | rhpR_rhpS-Mutant_KB_R1_peak_134 | 2821 | 3162689 | 3163060 | - | overlapStart | 29 | 29 | NearestLocation |
| PSPPH_2792 |  | prophage PSPPH05, DNA-binding protein | Add | 3231564 | 3231707 | 144 | * | rhpR_rhpS-Mutant_KB_R1_peak_138 | 2883 | 3231624 | 3231971 | + | overlapStart | -60 | 60 | NearestLocation |
| PSPPH_3314 | ccmA | cytochrome c biogenesis protein CcmA | chromosome | 3836615 | 3836736 | 122 | * | rhpR_KB_R1_peak_22 | 3427 | 3835994 | 3836635 | - | overlapStart | 20 | 20 | NearestLocation |
| PSPPH_3414 | flgA | flagellar basal body P-ring biosynthesis protein FlgA | chromosome | 3946854 | 3946995 | 142 | * | rhpR_rhpS-Mutant_KB_R1_peak_154 | 3528 | 3946917 | 3947675 | + | overlapStart | -63 | 63 | NearestLocation |
| PSPPH_3658 |  | UDP-glucose 4-epimerase | chromosome | 4202455 | 4202532 | 78 | * | rhpR_rhpS-Mutant_KB_R1_peak_161 | 3780 | 4201524 | 4202504 | - | overlapStart | 49 | 28 | NearestLocation |
| PSPPH_3783 | proS | prolyl-tRNA synthetase | chromosome | 4332307 | 4332401 | 95 | * | rhpR_rhpS-Mutant_KB_R1_peak_167 | 3905 | 4332394 | 4334109 | + | overlapStart | -87 | 7 | NearestLocation |
| PSPPH_3955 | algU | RNA polymerase sigma factor AlgU | chromosome | 4518284 | 4518436 | 153 | * | rhpR_rhpS-Mutant_KB_R1_peak_174 | 4079 | 4517832 | 4518413 | - | overlapStart | 129 | 23 | NearestLocation |
| PSPPH_3994 | adhB | alcohol dehydrogenase | chromosome | 4559642 | 4559827 | 186 | * | rhpR_KB_R1_peak_29 | 4121 | 4558558 | 4559706 | - | overlapStart | 64 | 64 | NearestLocation |
| PSPPH_4192 |  | tRNA-Met | chromosome | 4780795 | 4780999 | 205 | * | rhpR_rhpS-Mutant_KB_R1_peak_184 | 4329 | 4780755 | 4780831 | - | overlapStart | 36 | 36 | NearestLocation |
| PSPPH_4210 | omlA | outer membrane lipoprotein OmlA | chromosome | 4800095 | 4800333 | 239 | * | rhpR_rhpS-Mutant_KB_R1_peak_186 | 4347 | 4800318 | 4800839 | + | overlapStart | -223 | 15 | NearestLocation |
| PSPPH_4216 |  | sRNA | chromosome | 4804249 | 4804557 | 309 | * | rhpR_rhpS-Mutant_KB_R1_peak_187 | 4353 | 4804338 | 4804725 | + | overlapStart | -89 | 89 | NearestLocation |
| PSPPH_4245 |  | GAF domain-containing protein | chromosome | 4842937 | 4843137 | 201 | * | rhpR_rhpS-Mutant_KB_R1_peak_189 | 4383 | 4843131 | 4843619 | + | overlapStart | -194 | 6 | NearestLocation |
| PSPPH_4246 |  | acetyltransferase | chromosome | 4844028 | 4844319 | 292 | * | rhpR_rhpS-Mutant_KB_R1_peak_190 | 4384 | 4843633 | 4844088 | - | overlapStart | 60 | 60 | NearestLocation |
| PSPPH_4259 |  | peptide ABC transporter substrate-binding protein | chromosome | 4860243 | 4860366 | 124 | * | rhpR_KB_R1_peak_33 | 4397 | 4860267 | 4861985 | + | overlapStart | -24 | 24 | NearestLocation |
| PSPPH_4327 | glyA | serine hydroxymethyltransferase | chromosome | 4946901 | 4947043 | 143 | * | rhpR_rhpS-Mutant_KB_R1_peak_196 | 4466 | 4947036 | 4948289 | + | overlapStart | -135 | 7 | NearestLocation |
| PSPPH_4386 |  | hypothetical protein | chromosome | 5010424 | 5010501 | 78 | * | rhpR_rhpS-Mutant_KB_R1_peak_199 | 4526 | 5010033 | 5010425 | - | overlapStart | 1 | 1 | NearestLocation |
| PSPPH_4606 |  | tRNA-Trp | chromosome | 5244050 | 5244240 | 191 | * | rhpR_rhpS-Mutant_KB_R1_peak_215 | 4748 | 5244035 | 5244110 | - | overlapStart | 60 | 15 | NearestLocation |
| PSPPH_4607 |  | tRNA-Thr | chromosome | 5244309 | 5244508 | 200 | * | rhpR_rhpS-Mutant_KB_R1_peak_216 | 4749 | 5244276 | 5244351 | - | overlapStart | 42 | 33 | NearestLocation |
| PSPPH_4619 |  | beta alanine--pyruvate transaminase | chromosome | 5255741 | 5255869 | 129 | * | rhpR_rhpS-Mutant_KB_R1_peak_218 | 4761 | 5254425 | 5255771 | - | overlapStart | 30 | 30 | NearestLocation |
| PSPPH_5218 | gidA | tRNA uridine 5-carboxymethylaminomethyl modification protein GidA | chromosome | 5922375 | 5922478 | 104 | * | rhpR_rhpS-Mutant_KB_R1_peak_239 | 5384 | 5920516 | 5922408 | - | overlapStart | 33 | 33 | NearestLocation |
| PSPPH_2221 | cobM | precorrin-4 C(11)-methyltransferase | chromosome | 2579596 | 2579810 | 215 | * | rhpR_KB_R1_peak_19 | 2293 | 2579623 | 2580372 | - | overlapEnd | 776 | 27 | NearestLocation |
| PSPPH_3350 |  | hypothetical protein | chromosome | 3878976 | 3879165 | 190 | * | rhpR_KB_R1_peak_23 | 3464 | 3879030 | 3879500 | - | overlapEnd | 524 | 54 | NearestLocation |
| PSPPH_3685 |  | hypothetical protein | chromosome | 4233219 | 4233323 | 105 | * | rhpR_rhpS-Mutant_KB_R1_peak_163 | 3807 | 4232944 | 4233237 | + | overlapEnd | 275 | 18 | NearestLocation |
| PSPPH_4975 |  | hypothetical protein | chromosome | 5643642 | 5644030 | 389 | * | rhpR_rhpS-Mutant_KB_R1_peak_228 | 5133 | 5643544 | 5643945 | + | overlapEnd | 98 | 85 | NearestLocation |
| PSPPH_0102 |  | ParB-like nuclease | chromosome | 113897 | 114014 | 118 | * | rhpR_rhpS-Mutant_KB_R1_peak_5 | 105 | 113614 | 114579 | + | inside | 283 | 283 | NearestLocation |
| PSPPH_0165 |  | hypothetical protein | chromosome | 189781 | 189863 | 83 | * | rhpR_rhpS-Mutant_KB_R1_peak_9 | 171 | 189713 | 190558 | + | inside | 68 | 68 | NearestLocation |
| PSPPH_0177 |  | LysR family transcriptional regulator | chromosome | 211126 | 211231 | 106 | * | rhpR_rhpS-Mutant_KB_R1_peak_12 | 186 | 210746 | 211510 | + | inside | 380 | 279 | NearestLocation |
| PSPPH_1368 |  | YaeQ family protein | chromosome | 1587512 | 1587650 | 139 | * | rhpR_rhpS-Mutant_KB_R1_peak_85 | 1409 | 1587441 | 1588172 | + | inside | 71 | 71 | NearestLocation |
| PSPPH_1641 | fabG | 3-ketoacyl-ACP reductase | chromosome | 1904155 | 1904315 | 161 | * | rhpR_KB_R1_peak_15 | 1693 | 1903900 | 1904643 | + | inside | 255 | 255 | NearestLocation |
| PSPPH_1976 | sdhC | succinate dehydrogenase, cytochrome b556 subunit | chromosome | 2322026 | 2322136 | 111 | * | rhpR_rhpS-Mutant_KB_R1_peak_106 | 2036 | 2321961 | 2322335 | + | inside | 65 | 65 | NearestLocation |
| PSPPH_4102 | lpxC | UDP-3-O-[3-hydroxymyristoyl] N-acetylglucosamine deacetylase | chromosome | 4687286 | 4687364 | 79 | * | rhpR_rhpS-Mutant_KB_R1_peak_181 | 4239 | 4686624 | 4687535 | - | inside | 249 | 171 | NearestLocation |
| PSPPH_4541 |  | hypothetical protein | chromosome | 5177196 | 5177331 | 136 | * | rhpR_rhpS-Mutant_KB_R1_peak_204 | 4682 | 5176817 | 5177851 | + | inside | 379 | 379 | NearestLocation |
| PSPPH_4604 | nusG | transcription antitermination protein NusG | chromosome | 5243527 | 5243607 | 81 | * | rhpR_rhpS-Mutant_KB_R1_peak_213 | 4746 | 5243077 | 5243610 | - | inside | 83 | 3 | NearestLocation |
| PSPPH_4605 | secE | preprotein translocase subunit SecE | chromosome | 5243799 | 5243918 | 120 | * | rhpR_rhpS-Mutant_KB_R1_peak_214 | 4747 | 5243620 | 5243988 | - | inside | 189 | 70 | NearestLocation |
| PSPPH_0172 |  | pseudogene | chromosome | 206405 | 206497 | 93 | * | rhpR_rhpS-Mutant_KB_R1_peak_11 | 180 | 206633 | 206890 | - | downstream | 485 | 136 | NearestLocation |
| PSPPH_0759 |  | type IV pilus protein PilM | chromosome | 889054 | 889200 | 147 | * | rhpR_KB_R1_peak_7 | 776 | 889391 | 889831 | - | downstream | 777 | 191 | NearestLocation |
| PSPPH_1077 |  | hypothetical protein | chromosome | 1267155 | 1267244 | 90 | * | rhpR_rhpS-Mutant_KB_R1_peak_68 | 1105 | 1266510 | 1267055 | + | downstream | 645 | 100 | NearestLocation |
| PSPPH_1375 |  | hypothetical protein | chromosome | 1596455 | 1596541 | 87 | * | rhpR_rhpS-Mutant_KB_R1_peak_87 | 1416 | 1596630 | 1596812 | - | downstream | 357 | 89 | NearestLocation |
| PSPPH_1423 |  | hypothetical protein | chromosome | 1651869 | 1652203 | 335 | * | rhpR_KB_R1_peak_12 | 1465 | 1651696 | 1651851 | + | downstream | 173 | 18 | NearestLocation |
| PSPPH_3490 |  | hypothetical protein | chromosome | 4031443 | 4031598 | 156 | * | rhpR_rhpS-Mutant_KB_R1_peak_156 | 3608 | 4031056 | 4031223 | + | downstream | 387 | 220 | NearestLocation |
| PSPPH_4041 |  | fumarate hydratase, class I | chromosome | 4617421 | 4617563 | 143 | * | rhpR_rhpS-Mutant_KB_R1_peak_180 | 4172 | 4615638 | 4617161 | + | downstream | 1783 | 260 | NearestLocation |
| PSPPH_4321 |  | hypothetical protein | chromosome | 4935714 | 4935812 | 99 | * | rhpR_rhpS-Mutant_KB_R1_peak_195 | 4459 | 4936398 | 4937021 | - | downstream | 1307 | 586 | NearestLocation |
| PSPPH_4558 |  | MutT domain-containing protein | chromosome | 5197745 | 5197821 | 77 | * | rhpR_rhpS-Mutant_KB_R1_peak_206 | 4699 | 5197852 | 5198625 | - | downstream | 880 | 31 | NearestLocation |
| PSPPH_4687 |  | tRNA-Met | chromosome | 5323058 | 5323148 | 91 | * | rhpR_rhpS-Mutant_KB_R1_peak_220 | 4829 | 5323252 | 5323328 | - | downstream | 270 | 104 | NearestLocation |
