# Supplementary material for: Pseudomonas savastanoi Two-Component System RhpRS Switches between Virulence and Metabolism by Tuning Phosphorylation State and Sensing Nutritional Conditions
Source: mBio. 2019 Mar 19;10(2):e02838-18. doi: 10.1128/mBio.02838-18 (PMC6426608; doi:10.1128/mBio.02838-18)
Supplement: TABLE S4 [file mBio.02838-18-st004.docx]

**Table S4. RNA-seq data**

**A. List of genes upregulated in *rhpS* mutant in KB**

| gene | annotation | locus | sample_1 | sample_2 | status | value_1 | value_2 | log2(fold_change) | test_stat | p_value | q_value |
| --- | --- | --- | --- | --- | --- | --- | --- | --- | --- | --- | --- |
| PSPPH_3248 | diguanylate cyclase | chromosome:3763464-3765036 | 1448A-rhpS-KB | 1448A-WT-KB | OK | 71.5534 | 35.7692 | -1.0003 | -1.3504 | 0.0557 | 0.215446 |
| PSPPH_0202 | hypothetical protein | chromosome:239581-239950 | 1448A-rhpS-KB | 1448A-WT-KB | OK | 522.635 | 261.219 | -1.00055 | -1.38427 | 0.0514 | 0.20734 |
| PSPPH_1230 | hypothetical protein | chromosome:1439643-1439925 | 1448A-rhpS-KB | 1448A-WT-KB | OK | 779.441 | 389.235 | -1.0018 | -1.28544 | 0.06535 | 0.2347 |
| PSPPH_1826 | hypothetical protein | chromosome:2126961-2127198 | 1448A-rhpS-KB | 1448A-WT-KB | OK | 181.242 | 90.4575 | -1.00261 | -0.589033 | 0.4444 | 0.665111 |
| PSPPH_1003 | hypothetical protein | chromosome:1195536-1195758 | 1448A-rhpS-KB | 1448A-WT-KB | OK | 93.1328 | 46.4407 | -1.0039 | -0.442988 | 0.55525 | 0.750472 |
| PSPPH_5122 | phosphate ABC transporter permease | chromosome:5809409-5811671 | 1448A-rhpS-KB | 1448A-WT-KB | OK | 47.555 | 23.6873 | -1.00548 | -1.36397 | 0.05205 | 0.208051 |
| PSPPH_1342 | hypothetical protein | chromosome:1561022-1561334 | 1448A-rhpS-KB | 1448A-WT-KB | OK | 285.017 | 141.591 | -1.00932 | -0.896601 | 0.20545 | 0.441008 |
| mmsA2 | methylmalonate-semialdehyde dehydrogenase | chromosome:5252826-5254320 | 1448A-rhpS-KB | 1448A-WT-KB | OK | 349.277 | 173.453 | -1.00982 | -2.43635 | 0.0012 | 0.014789 |
| PSPPH_0218 | GntR family transcriptional regulator | chromosome:256501-257923 | 1448A-rhpS-KB | 1448A-WT-KB | OK | 428.986 | 212.934 | -1.01053 | -2.63357 | 0.00045 | 0.00699261 |
| PSPPH_2117 | FecR protein superfamily protein | chromosome:2476250-2477279 | 1448A-rhpS-KB | 1448A-WT-KB | OK | 253.112 | 125.559 | -1.01141 | -1.92005 | 0.0096 | 0.0675402 |
| PSPPH_2862 | AraC family transcriptional regulator | chromosome:3308613-3309447 | 1448A-rhpS-KB | 1448A-WT-KB | OK | 83.7667 | 41.5291 | -1.01226 | -1.11936 | 0.11955 | 0.327161 |
| PSPPH_0896 | acyltransferase | chromosome:1067704-1068931 | 1448A-rhpS-KB | 1448A-WT-KB | NOTEST | 19.2296 | 9.52409 | -1.01367 | 0 | 1 | 1 |
| fliC | flagellin | chromosome:3919859-3920708 | 1448A-rhpS-KB | 1448A-WT-KB | OK | 125.18 | 61.9926 | -1.01384 | -1.30043 | 0.07895 | 0.260948 |
| rpsP | 30S ribosomal protein S16 | chromosome:1571686-1571944 | 1448A-rhpS-KB | 1448A-WT-KB | OK | 5643.76 | 2794.8 | -1.01391 | -2.56824 | 0.00085 | 0.01121 |
| PSPPH_5198 | dTDP-glucose 4,6-dehydratase | chromosome:5898993-5899368 | 1448A-rhpS-KB | 1448A-WT-KB | OK | 64.5199 | 31.9396 | -1.0144 | -0.477538 | 0.4423 | 0.663079 |
| PSPPH_4485 | hypothetical protein | chromosome:5120871-5121882 | 1448A-rhpS-KB | 1448A-WT-KB | OK | 130.216 | 64.4534 | -1.01458 | -1.38533 | 0.05415 | 0.211742 |
| alkB | alkylated DNA repair protein AlkB | chromosome:2619003-2619690 | 1448A-rhpS-KB | 1448A-WT-KB | OK | 134.774 | 66.6863 | -1.01508 | -1.18777 | 0.0889 | 0.277492 |
| PSPPH_1821 | hypothetical protein | chromosome:2121524-2123023 | 1448A-rhpS-KB | 1448A-WT-KB | OK | 156.897 | 77.5978 | -1.01573 | -1.0791 | 0.14035 | 0.354997 |
| PSPPH_0039 | hypothetical protein | chromosome:44109-44325 | 1448A-rhpS-KB | 1448A-WT-KB | OK | 304.34 | 150.507 | -1.01585 | -0.555678 | 0.40785 | 0.636843 |
| PSPPH_1050 | bmp family protein | chromosome:1241701-1246332 | 1448A-rhpS-KB | 1448A-WT-KB | OK | 182.762 | 90.3263 | -1.01675 | -1.56401 | 0.04575 | 0.193047 |
| PSPPH_0034 | CigR | chromosome:39885-40416 | 1448A-rhpS-KB | 1448A-WT-KB | OK | 216.438 | 106.957 | -1.01692 | -1.29975 | 0.08205 | 0.265863 |
| dipZ | thiol:disulfide interchange protein | chromosome:5072933-5074754 | 1448A-rhpS-KB | 1448A-WT-KB | OK | 110.463 | 54.4967 | -1.01932 | -1.74167 | 0.0187 | 0.106484 |
| folC | bifunctional folylpolyglutamate synthase/dihydrofolate synthase | chromosome:1920330-1923195 | 1448A-rhpS-KB | 1448A-WT-KB | OK | 202.365 | 99.763 | -1.02038 | -1.2527 | 0.07695 | 0.25872 |
| PSPPH_3748 | hypothetical protein | chromosome:4300500-4300941 | 1448A-rhpS-KB | 1448A-WT-KB | OK | 132.294 | 65.2134 | -1.02051 | -0.843356 | 0.2192 | 0.456215 |
| cyoC | cytochrome o ubiquinol oxidase subunit III | chromosome:1422468-1423427 | 1448A-rhpS-KB | 1448A-WT-KB | OK | 5775.83 | 2844.17 | -1.02202 | -3.36215 | 0.00005 | 0.00121565 |
| PSPPH_0581 | hypothetical protein | chromosome:681034-683350 | 1448A-rhpS-KB | 1448A-WT-KB | OK | 273.911 | 134.774 | -1.02317 | -2.78498 | 0.00015 | 0.00302881 |
| PSPPH_1556 | hypothetical protein | chromosome:1808066-1808441 | 1448A-rhpS-KB | 1448A-WT-KB | OK | 774.641 | 381.009 | -1.0237 | -1.7326 | 0.02335 | 0.123715 |
| PSPPH_3933 | major facilitator family transporter | chromosome:4493388-4494729 | 1448A-rhpS-KB | 1448A-WT-KB | OK | 117.218 | 57.6301 | -1.02429 | -1.54206 | 0.0379 | 0.171245 |
| pstB | phosphate transporter ATP-binding protein | chromosome:3500875-3503531 | 1448A-rhpS-KB | 1448A-WT-KB | OK | 72.9332 | 35.8299 | -1.02541 | -0.70071 | 0.36475 | 0.599002 |
| PSPPH_3877 | sensor histidine kinase/response regulator | chromosome:4430452-4436346 | 1448A-rhpS-KB | 1448A-WT-KB | OK | 213.723 | 104.99 | -1.0255 | -1.84467 | 0.0098 | 0.0686236 |
| PSPPH_2995 | DNA-binding response regulator | chromosome:3477440-3478115 | 1448A-rhpS-KB | 1448A-WT-KB | OK | 98.9951 | 48.5579 | -1.02765 | -1.02499 | 0.14485 | 0.362531 |
| PSPPH_3806 | competence/damage inducible protein CinA | chromosome:4356558-4357443 | 1448A-rhpS-KB | 1448A-WT-KB | OK | 117.554 | 57.6153 | -1.0288 | -1.30197 | 0.0693 | 0.241812 |
| PSPPH_1723 | HlyD family secretion protein | chromosome:1993962-1996745 | 1448A-rhpS-KB | 1448A-WT-KB | OK | 89.6825 | 43.9432 | -1.02919 | -1.16678 | 0.0955 | 0.286399 |
| prsA | ribose-phosphate pyrophosphokinase | chromosome:1185153-1186095 | 1448A-rhpS-KB | 1448A-WT-KB | OK | 2413.74 | 1182.07 | -1.02995 | -3.61674 | 0.00005 | 0.00121565 |
| PSPPH_0408 | hypothetical protein | chromosome:472133-474040 | 1448A-rhpS-KB | 1448A-WT-KB | OK | 109.331 | 53.4915 | -1.03133 | -0.777643 | 0.2731 | 0.509906 |
| PSPPH_3182 | RNA polymerase-binding protein DksA | chromosome:3690442-3690847 | 1448A-rhpS-KB | 1448A-WT-KB | OK | 1322.35 | 646.759 | -1.0318 | -2.18466 | 0.00355 | 0.0333887 |
| PSPPH_0217 | phospholipase D | chromosome:253703-256429 | 1448A-rhpS-KB | 1448A-WT-KB | OK | 134.164 | 65.615 | -1.0319 | -1.68438 | 0.0223 | 0.119312 |
| PSPPH_0414 | thioester dehydrase | chromosome:476539-481262 | 1448A-rhpS-KB | 1448A-WT-KB | OK | 104.127 | 50.8865 | -1.03299 | -0.557513 | 0.39035 | 0.619107 |
| PSPPH_4072 | inaA protein | chromosome:4654466-4655413 | 1448A-rhpS-KB | 1448A-WT-KB | OK | 470.275 | 229.727 | -1.03358 | -1.77568 | 0.01375 | 0.0865185 |
| PSPPH_2917 | DNA-binding protein | chromosome:3389553-3390129 | 1448A-rhpS-KB | 1448A-WT-KB | OK | 126.055 | 61.577 | -1.03359 | -1.04768 | 0.14905 | 0.369545 |
| PSPPH_2487 | TauD/TfdA family dioxygenase | chromosome:2872637-2873477 | 1448A-rhpS-KB | 1448A-WT-KB | OK | 129.272 | 63.0712 | -1.03536 | -1.28898 | 0.07145 | 0.246965 |
| PSPPH_3023 | abrB protein | chromosome:3509236-3510325 | 1448A-rhpS-KB | 1448A-WT-KB | OK | 270.038 | 131.647 | -1.03649 | -1.97249 | 0.0088 | 0.0639252 |
| PSPPH_2549 | xanthine dehydrogenase accessory factor | chromosome:2941320-2942863 | 1448A-rhpS-KB | 1448A-WT-KB | OK | 54.549 | 26.5801 | -1.0372 | -0.891807 | 0.2154 | 0.451784 |
| PSPPH_0111 | acetyltransferase | chromosome:128274-129446 | 1448A-rhpS-KB | 1448A-WT-KB | OK | 120.689 | 58.7919 | -1.03761 | -0.575625 | 0.3453 | 0.580439 |
| PSPPH_1848 | isochorismatase | chromosome:2144222-2144789 | 1448A-rhpS-KB | 1448A-WT-KB | OK | 88.1363 | 42.9199 | -1.03809 | -0.83175 | 0.24465 | 0.477803 |
| cheB2 | protein-glutamate methylesterase CheB | chromosome:3000449-3003057 | 1448A-rhpS-KB | 1448A-WT-KB | OK | 143.155 | 69.6631 | -1.03911 | -0.865096 | 0.21365 | 0.449432 |
| PSPPH_3175 | monovalent cation/H+ antiporter subunit E | chromosome:3684561-3685702 | 1448A-rhpS-KB | 1448A-WT-KB | OK | 165.953 | 80.7201 | -1.03977 | -0.88132 | 0.25095 | 0.485333 |
| PSPPH_5107 | hypothetical protein | chromosome:5794572-5794950 | 1448A-rhpS-KB | 1448A-WT-KB | OK | 259.456 | 126.181 | -1.04 | -1.03537 | 0.15235 | 0.372689 |
| PSPPH_4419 | LuxR family transcriptional regulator | chromosome:5044415-5045192 | 1448A-rhpS-KB | 1448A-WT-KB | OK | 85.5007 | 41.551 | -1.04105 | -1.03682 | 0.14405 | 0.362305 |
| PSPPH_3075 | protease inhibitor Inh | chromosome:3573487-3573871 | 1448A-rhpS-KB | 1448A-WT-KB | OK | 185.061 | 89.8888 | -1.04179 | -0.905251 | 0.2159 | 0.452141 |
| PSPPH_2813 | hypothetical protein | chromosome:3258678-3259698 | 1448A-rhpS-KB | 1448A-WT-KB | OK | 142.807 | 69.3554 | -1.04199 | -1.49779 | 0.04035 | 0.177819 |
| capA | cold shock protein CapA | chromosome:2489987-2490200 | 1448A-rhpS-KB | 1448A-WT-KB | OK | 2923.57 | 1419.73 | -1.04212 | -1.71858 | 0.0188 | 0.106484 |
| PSPPH_1503 | hypothetical protein | chromosome:1744891-1745224 | 1448A-rhpS-KB | 1448A-WT-KB | OK | 58.7479 | 28.5266 | -1.04223 | -0.446878 | 0.45515 | 0.674324 |
| PSPPH_3969 | lipoprotein | chromosome:4530709-4531201 | 1448A-rhpS-KB | 1448A-WT-KB | OK | 408.865 | 198.522 | -1.04232 | -1.60787 | 0.02705 | 0.135782 |
| hopV1 | type III effector HopV1 | chromosome:2725395-2727093 | 1448A-rhpS-KB | 1448A-WT-KB | NOTEST | 41.4644 | 20.1252 | -1.04287 | 0 | 1 | 1 |
| baeS1 | DNA-binding response regulator BaeS | chromosome:2754671-2756743 | 1448A-rhpS-KB | 1448A-WT-KB | NOTEST | 43.7048 | 21.189 | -1.04448 | 0 | 1 | 1 |
| PSPPH_4562 | major facilitator family transporter | chromosome:5207051-5208452 | 1448A-rhpS-KB | 1448A-WT-KB | OK | 382.988 | 185.617 | -1.04497 | -2.50333 | 0.0007 | 0.00962231 |
| algF | alginate biosynthesis protein AlgF | chromosome:1303715-1304384 | 1448A-rhpS-KB | 1448A-WT-KB | OK | 539.945 | 261.65 | -1.04517 | -2.13749 | 0.0056 | 0.0457995 |
| PSPPH_5123 | phosphate ABC transporter substrate-binding protein | chromosome:5811837-5812836 | 1448A-rhpS-KB | 1448A-WT-KB | NOTEST | 44.3914 | 21.5091 | -1.04533 | 0 | 1 | 1 |
| wspD | chemotaxis protein CheW | chromosome:4430452-4436346 | 1448A-rhpS-KB | 1448A-WT-KB | OK | 247.823 | 120.059 | -1.04556 | -1.09788 | 0.121 | 0.329614 |
| PSPPH_3177 | monovalent cation/H+ antiporter subunit G | chromosome:3684561-3685702 | 1448A-rhpS-KB | 1448A-WT-KB | OK | 311.961 | 151.056 | -1.04628 | -1.04842 | 0.1468 | 0.365619 |
| PSPPH_3833 | zinc metalloprotease | chromosome:4388142-4389495 | 1448A-rhpS-KB | 1448A-WT-KB | OK | 653.76 | 316.547 | -1.04634 | -2.95191 | 0.00005 | 0.00121565 |
| PSPPH_2161 | flavin reductase domain-containing protein | chromosome:2513941-2517414 | 1448A-rhpS-KB | 1448A-WT-KB | OK | 101.693 | 49.2051 | -1.04734 | -0.663613 | 0.40625 | 0.635491 |
| PSPPH_3683 | hypothetical protein | chromosome:4231058-4232018 | 1448A-rhpS-KB | 1448A-WT-KB | OK | 295.076 | 142.473 | -1.0504 | -1.91563 | 0.0107 | 0.0732602 |
| PSPPH_3678 | hypothetical protein | chromosome:4226906-4227860 | 1448A-rhpS-KB | 1448A-WT-KB | OK | 752.102 | 362.353 | -1.05353 | -2.79589 | 0.00035 | 0.00566018 |
| PSPPH_3267 | hypothetical protein | chromosome:3786733-3787321 | 1448A-rhpS-KB | 1448A-WT-KB | OK | 116.969 | 56.3221 | -1.05436 | -0.98051 | 0.17395 | 0.403438 |
| PSPPH_1967 | lipoprotein | chromosome:2315642-2316808 | 1448A-rhpS-KB | 1448A-WT-KB | OK | 1461.62 | 703.501 | -1.05495 | -1.49656 | 0.03985 | 0.177144 |
| pstB2 | phosphate transporter ATP-binding protein | chromosome:5806823-5807657 | 1448A-rhpS-KB | 1448A-WT-KB | OK | 202.16 | 97.3004 | -1.05498 | -1.55313 | 0.03145 | 0.15007 |
| PSPPH_2053 | hypothetical protein | chromosome:2404760-2407273 | 1448A-rhpS-KB | 1448A-WT-KB | OK | 135.717 | 65.2895 | -1.05568 | -1.666 | 0.0208 | 0.113149 |
| PSPPH_1174 | regulatory protein | chromosome:1379244-1380210 | 1448A-rhpS-KB | 1448A-WT-KB | OK | 74.9089 | 35.9823 | -1.05785 | -1.10457 | 0.12915 | 0.340149 |
| PSPPH_3635 | hypothetical protein | chromosome:4180668-4181430 | 1448A-rhpS-KB | 1448A-WT-KB | OK | 112.903 | 54.2075 | -1.05851 | -1.19562 | 0.1014 | 0.296323 |
| pilG | type IV pilus response regulator PilG | chromosome:541959-542370 | 1448A-rhpS-KB | 1448A-WT-KB | OK | 414.224 | 198.85 | -1.05873 | -1.40405 | 0.05665 | 0.217239 |
| PSPPH_1868 | anti-sigma factor antagonist | chromosome:2167352-2169211 | 1448A-rhpS-KB | 1448A-WT-KB | OK | 731.397 | 350.855 | -1.05978 | -1.39263 | 0.0651 | 0.234308 |
| PSPPH_5030 | peptide ABC transporter substrate-binding protein | chromosome:5699456-5701094 | 1448A-rhpS-KB | 1448A-WT-KB | OK | 172.526 | 82.6961 | -1.06092 | -2.12689 | 0.0045 | 0.0394191 |
| dksA1 | RNA polymerase-binding protein DksA | chromosome:895982-896399 | 1448A-rhpS-KB | 1448A-WT-KB | OK | 1071.01 | 513.254 | -1.06122 | -2.11238 | 0.0047 | 0.0406726 |
| PSPPH_3779 | cold shock domain-contain protein | chromosome:4329113-4329335 | 1448A-rhpS-KB | 1448A-WT-KB | OK | 904.926 | 433.397 | -1.06211 | -1.08927 | 0.1246 | 0.334576 |
| hrpJ | type III secretion component protein HrcJ | chromosome:1502227-1505418 | 1448A-rhpS-KB | 1448A-WT-KB | NOTEST | 18.0531 | 8.6447 | -1.06236 | 0 | 1 | 1 |
| PSPPH_1903 | universal stress protein family protein | chromosome:2208996-2209428 | 1448A-rhpS-KB | 1448A-WT-KB | OK | 1634.82 | 782.222 | -1.06349 | -2.49953 | 0.00085 | 0.01121 |
| cyoD | cytochrome o ubiquinol oxidase subunit IV | chromosome:1422468-1423427 | 1448A-rhpS-KB | 1448A-WT-KB | OK | 5724.57 | 2734.85 | -1.0657 | -2.14216 | 0.0028 | 0.0288392 |
| fliF | flagellar MS-ring protein | chromosome:3910208-3911993 | 1448A-rhpS-KB | 1448A-WT-KB | OK | 70.3747 | 33.5795 | -1.06747 | -1.48777 | 0.0411 | 0.180014 |
| PSPPH_3130 | 6-pyruvoyl tetrahydrobiopterin synthase | chromosome:3630954-3631311 | 1448A-rhpS-KB | 1448A-WT-KB | OK | 492.001 | 234.612 | -1.06838 | -1.39033 | 0.0553 | 0.21413 |
| rnpA | ribonuclease P | chromosome:5926138-5926778 | 1448A-rhpS-KB | 1448A-WT-KB | OK | 1105.73 | 527.248 | -1.06845 | -1.93275 | 0.0077 | 0.0575728 |
| PSPPH_3764 | radical SAM domain-containing protein | chromosome:4315439-4316087 | 1448A-rhpS-KB | 1448A-WT-KB | OK | 113.471 | 53.9641 | -1.07226 | -1.06611 | 0.1393 | 0.354997 |
| PSPPH_3043 | RND family efflux transporter MFP subunit | chromosome:3534631-3539978 | 1448A-rhpS-KB | 1448A-WT-KB | OK | 1089.43 | 517.776 | -1.07317 | -2.99286 | 0.00005 | 0.00121565 |
| dadX | alanine racemase | chromosome:262497-263571 | 1448A-rhpS-KB | 1448A-WT-KB | OK | 1208.23 | 574.043 | -1.07367 | -3.33786 | 0.00005 | 0.00121565 |
| PSPPH_5015 | hypothetical protein | chromosome:5682258-5682534 | 1448A-rhpS-KB | 1448A-WT-KB | OK | 634.507 | 301.409 | -1.07391 | -1.22829 | 0.09815 | 0.290869 |
| fabA | 3-hydroxydecanoyl-ACP dehydratase | chromosome:2340021-2340537 | 1448A-rhpS-KB | 1448A-WT-KB | OK | 1557.48 | 739.74 | -1.07413 | -2.71166 | 0.0005 | 0.00757203 |
| PSPPH_1077 | hypothetical protein | chromosome:1266509-1267055 | 1448A-rhpS-KB | 1448A-WT-KB | OK | 44.5613 | 21.149 | -1.0752 | -0.561973 | 0.3842 | 0.616306 |
| gcp | DNA-binding/iron metalloprotein/AP endonuclease | chromosome:737566-738592 | 1448A-rhpS-KB | 1448A-WT-KB | OK | 153.469 | 72.8279 | -1.07538 | -1.60654 | 0.02955 | 0.143885 |
| greA | transcription elongation factor GreA | chromosome:4787730-4791425 | 1448A-rhpS-KB | 1448A-WT-KB | OK | 2013.29 | 954.901 | -1.07613 | -1.51876 | 0.03625 | 0.165463 |
| PSPPH_1726 | aldo/keto reductase | chromosome:1999841-2000663 | 1448A-rhpS-KB | 1448A-WT-KB | OK | 234.02 | 110.898 | -1.0774 | -1.76442 | 0.01635 | 0.0982099 |
| PSPPH_3686 | DNA recombination protein rmuC-like protein | chromosome:4233645-4235124 | 1448A-rhpS-KB | 1448A-WT-KB | OK | 68.3033 | 32.3605 | -1.07772 | -1.34227 | 0.07425 | 0.254429 |
| PSPPH_1803 | lipoprotein | chromosome:2108201-2108588 | 1448A-rhpS-KB | 1448A-WT-KB | OK | 491.976 | 232.918 | -1.07876 | -1.47749 | 0.0424 | 0.18346 |
| PSPPH_3409 | hypothetical protein | chromosome:3943000-3943474 | 1448A-rhpS-KB | 1448A-WT-KB | OK | 418.008 | 197.892 | -1.07881 | -1.58805 | 0.03065 | 0.147038 |
| PSPPH_0725 | type IV pilus biogenesis protein | chromosome:848890-849391 | 1448A-rhpS-KB | 1448A-WT-KB | OK | 170.26 | 80.5253 | -1.08023 | -1.13141 | 0.12915 | 0.340149 |
| PSPPH_0501 | hypothetical protein | chromosome:581323-581620 | 1448A-rhpS-KB | 1448A-WT-KB | OK | 254.03 | 120.124 | -1.08048 | -0.837177 | 0.24515 | 0.478518 |
| PSPPH_0966 | ISPsy23, transposition helper protein | chromosome:1149303-1149723 | 1448A-rhpS-KB | 1448A-WT-KB | NOTEST | 38.2095 | 18.0505 | -1.08189 | 0 | 1 | 1 |
| PSPPH_0311 | hypothetical protein | chromosome:353292-353874 | 1448A-rhpS-KB | 1448A-WT-KB | OK | 58.211 | 27.459 | -1.08401 | -0.751071 | 0.3115 | 0.547785 |
| cyoE | protoheme IX farnesyltransferase | chromosome:1423437-1424325 | 1448A-rhpS-KB | 1448A-WT-KB | OK | 3955.13 | 1864.74 | -1.08475 | -4.04011 | 0.00005 | 0.00121565 |
| PSPPH_5042 | AsnC family transcriptional regulator | chromosome:5713736-5714225 | 1448A-rhpS-KB | 1448A-WT-KB | OK | 214.442 | 101.031 | -1.08578 | -1.24411 | 0.08555 | 0.27082 |
| wspR | response regulator WspR | chromosome:4429402-4430407 | 1448A-rhpS-KB | 1448A-WT-KB | OK | 555.714 | 261.792 | -1.08592 | -2.6502 | 0.00025 | 0.0043799 |
| PSPPH_2047 | universal stress protein family protein | chromosome:2400018-2400507 | 1448A-rhpS-KB | 1448A-WT-KB | OK | 306.125 | 144.14 | -1.08665 | -1.41804 | 0.05395 | 0.211582 |
| hpt | hypoxanthine-guanine phosphoribosyltransferase | chromosome:1210150-1210708 | 1448A-rhpS-KB | 1448A-WT-KB | OK | 369.536 | 173.474 | -1.09099 | -1.71257 | 0.02265 | 0.120823 |
| PSPPH_3736 | sensor histidine kinase | chromosome:4282123-4284450 | 1448A-rhpS-KB | 1448A-WT-KB | NOTEST | 28.908 | 13.5682 | -1.09124 | 0 | 1 | 1 |
| PSPPH_0757 | hypothetical protein | chromosome:887335-888834 | 1448A-rhpS-KB | 1448A-WT-KB | OK | 190.289 | 89.3046 | -1.09139 | -1.04526 | 0.1897 | 0.419028 |
| PSPPH_2052 | hypothetical protein | chromosome:2404760-2407273 | 1448A-rhpS-KB | 1448A-WT-KB | OK | 98.2053 | 46.0574 | -1.09237 | -1.03795 | 0.1637 | 0.391085 |
| PSPPH_0475 | Holliday junction resolvase-like protein | chromosome:538608-539621 | 1448A-rhpS-KB | 1448A-WT-KB | OK | 440.758 | 206.514 | -1.09375 | -1.37091 | 0.0633 | 0.231259 |
| PSPPH_2630 | MerR family transcriptional regulator | chromosome:3035499-3035892 | 1448A-rhpS-KB | 1448A-WT-KB | OK | 1058.69 | 495.153 | -1.09634 | -2.0503 | 0.0065 | 0.0507227 |
| PSPPH_2442 | hypothetical protein | chromosome:2824041-2825234 | 1448A-rhpS-KB | 1448A-WT-KB | NOTEST | 35.5343 | 16.5946 | -1.0985 | 0 | 1 | 1 |
| PSPPH_2238 | hypothetical protein | chromosome:2596962-2597970 | 1448A-rhpS-KB | 1448A-WT-KB | OK | 112.614 | 52.5388 | -1.09993 | -1.42325 | 0.056 | 0.216058 |
| ampD | N-acetyl-anhydromuranmyl-L-alanine amidase | chromosome:1007259-1007808 | 1448A-rhpS-KB | 1448A-WT-KB | OK | 270.903 | 126.176 | -1.10234 | -1.44865 | 0.05205 | 0.208051 |
| PSPPH_3943 | methyltransferase | chromosome:4506260-4508056 | 1448A-rhpS-KB | 1448A-WT-KB | OK | 199.063 | 92.6412 | -1.1035 | -1.37733 | 0.0702 | 0.244299 |
| PSPPH_1904 | response regulator | chromosome:2209435-2213944 | 1448A-rhpS-KB | 1448A-WT-KB | OK | 750.146 | 348.974 | -1.10405 | -1.75895 | 0.0189 | 0.106712 |
| PSPPH_0157 | hypothetical protein | chromosome:184845-185394 | 1448A-rhpS-KB | 1448A-WT-KB | OK | 49.3116 | 22.9384 | -1.10416 | -0.667344 | 0.341 | 0.578003 |
| PSPPH_3141 | polysaccharide biosynthesis domain-containing protein | chromosome:3643524-3645057 | 1448A-rhpS-KB | 1448A-WT-KB | NOTEST | 23.6376 | 10.9858 | -1.10545 | 0 | 1 | 1 |
| PSPPH_1073 | hypothetical protein | chromosome:1263277-1263844 | 1448A-rhpS-KB | 1448A-WT-KB | OK | 111.618 | 51.7999 | -1.10755 | -0.974402 | 0.1822 | 0.41162 |
| PSPPH_0323 | ABC transporter substrate-binding protein | chromosome:367679-368699 | 1448A-rhpS-KB | 1448A-WT-KB | NOTEST | 35.9334 | 16.6682 | -1.10823 | 0 | 1 | 1 |
| PSPPH_1021 | hypothetical protein | chromosome:1211344-1211632 | 1448A-rhpS-KB | 1448A-WT-KB | OK | 602.674 | 279.548 | -1.10828 | -1.25545 | 0.08555 | 0.27082 |
| PSPPH_1449 | hypothetical protein | chromosome:1690554-1691130 | 1448A-rhpS-KB | 1448A-WT-KB | OK | 185.941 | 86.2207 | -1.10874 | -1.2791 | 0.08015 | 0.262322 |
| apt | adenine phosphoribosyltransferase | chromosome:3880448-3880997 | 1448A-rhpS-KB | 1448A-WT-KB | OK | 423.714 | 196.277 | -1.1102 | -1.77384 | 0.0164 | 0.098345 |
| PSPPH_3673 | diguanylate cyclase | chromosome:4220094-4221021 | 1448A-rhpS-KB | 1448A-WT-KB | OK | 215.622 | 99.8046 | -1.11133 | -1.79628 | 0.0159 | 0.0961533 |
| PSPPH_0276 | radical SAM domain-containing protein | chromosome:318059-319118 | 1448A-rhpS-KB | 1448A-WT-KB | OK | 187.764 | 86.8805 | -1.11182 | -1.84504 | 0.0139 | 0.0871554 |
| PSPPH_0857 | Fis family transcriptional regulator | chromosome:1025177-1026611 | 1448A-rhpS-KB | 1448A-WT-KB | OK | 298.583 | 138.051 | -1.11292 | -2.5098 | 0.00055 | 0.0080893 |
| ureF | urease accessory protein UreF | chromosome:5132789-5133961 | 1448A-rhpS-KB | 1448A-WT-KB | NOTEST | 42.7583 | 19.7154 | -1.11688 | 0 | 1 | 1 |
| PSPPH_2203 | MATE efflux family protein | chromosome:2557517-2558891 | 1448A-rhpS-KB | 1448A-WT-KB | OK | 118.606 | 54.65 | -1.11788 | -1.67821 | 0.03015 | 0.145661 |
| dacB | D-alanyl-D-alanine carboxypeptidase/D-alanyl-D-alanine-endopeptidase | chromosome:2443307-2444774 | 1448A-rhpS-KB | 1448A-WT-KB | OK | 222.992 | 102.746 | -1.1179 | -2.29053 | 0.00315 | 0.0310997 |
| PSPPH_1831 | hypothetical protein | chromosome:2131517-2131889 | 1448A-rhpS-KB | 1448A-WT-KB | OK | 222.667 | 102.564 | -1.11836 | -0.989661 | 0.1818 | 0.411497 |
| PSPPH_0321 | ABC transporter ATP-binding protein | chromosome:365801-366683 | 1448A-rhpS-KB | 1448A-WT-KB | NOTEST | 27.4843 | 12.6399 | -1.12062 | 0 | 1 | 1 |
| dgkA | diacylglycerol kinase | chromosome:4350859-4351225 | 1448A-rhpS-KB | 1448A-WT-KB | OK | 262.433 | 120.681 | -1.12076 | -1.06213 | 0.13305 | 0.347095 |
| ureG | urease accessory protein UreG | chromosome:5133982-5134600 | 1448A-rhpS-KB | 1448A-WT-KB | OK | 49.5048 | 22.7391 | -1.1224 | -0.741064 | 0.30965 | 0.546062 |
| mdoD | glucan biosynthesis protein D | chromosome:2202762-2204349 | 1448A-rhpS-KB | 1448A-WT-KB | NOTEST | 43.832 | 20.1252 | -1.12298 | 0 | 1 | 1 |
| PSPPH_1343 | hypothetical protein | chromosome:1561482-1562046 | 1448A-rhpS-KB | 1448A-WT-KB | OK | 213.506 | 98.0169 | -1.12317 | -1.36839 | 0.0677 | 0.239091 |
| PSPPH_1704 | ABC transporter ATP-binding protein | chromosome:1970276-1971887 | 1448A-rhpS-KB | 1448A-WT-KB | OK | 234.108 | 107.389 | -1.12432 | -2.42789 | 0.0018 | 0.0210235 |
| aat | leucyl/phenylalanyl-tRNA--protein transferase | chromosome:3591609-3592299 | 1448A-rhpS-KB | 1448A-WT-KB | OK | 157.776 | 72.3644 | -1.12452 | -1.34032 | 0.06635 | 0.235486 |
| PSPPH_1203 | hypothetical protein | chromosome:1414291-1415290 | 1448A-rhpS-KB | 1448A-WT-KB | NOTEST | 25.7669 | 11.7974 | -1.12705 | 0 | 1 | 1 |
| PSPPH_4699 | hypothetical protein | chromosome:5334598-5335666 | 1448A-rhpS-KB | 1448A-WT-KB | OK | 188.072 | 85.7198 | -1.13358 | -1.81094 | 0.01315 | 0.0846813 |
| secE | preprotein translocase subunit SecE | chromosome:5243619-5243988 | 1448A-rhpS-KB | 1448A-WT-KB | OK | 1290.25 | 587.771 | -1.13432 | -2.15356 | 0.0052 | 0.0434224 |
| PSPPH_1143 | hypothetical protein | chromosome:1343064-1343706 | 1448A-rhpS-KB | 1448A-WT-KB | OK | 154.361 | 70.3123 | -1.13446 | -1.30607 | 0.0774 | 0.259501 |
| PSPPH_1828 | hypothetical protein | chromosome:2128249-2129659 | 1448A-rhpS-KB | 1448A-WT-KB | OK | 162.35 | 73.9344 | -1.13479 | -2.00607 | 0.0098 | 0.0686236 |
| PSPPH_3979 | hypothetical protein | chromosome:4545655-4545880 | 1448A-rhpS-KB | 1448A-WT-KB | NOTEST | 47.478 | 21.5889 | -1.13697 | 0 | 1 | 1 |
| PSPPH_2681 | hypothetical protein | chromosome:3104228-3105080 | 1448A-rhpS-KB | 1448A-WT-KB | NOTEST | 30.7951 | 14 | -1.13727 | 0 | 1 | 1 |
| ahcY | S-adenosyl-L-homocysteine hydrolase | chromosome:513827-515237 | 1448A-rhpS-KB | 1448A-WT-KB | OK | 1058.31 | 480.673 | -1.13863 | -3.74813 | 0.00005 | 0.00121565 |
| PSPPH_5180 | hypothetical protein | chromosome:5878502-5881096 | 1448A-rhpS-KB | 1448A-WT-KB | OK | 45.3049 | 20.5515 | -1.14042 | -1.19794 | 0.10015 | 0.294113 |
| PSPPH_4038 | lipoprotein | chromosome:4612375-4612822 | 1448A-rhpS-KB | 1448A-WT-KB | NOTEST | 36.8554 | 16.69 | -1.14289 | 0 | 1 | 1 |
| PSPPH_3637 | hypothetical protein | chromosome:4182966-4183200 | 1448A-rhpS-KB | 1448A-WT-KB | OK | 180.081 | 81.3438 | -1.14654 | -0.580219 | 0.44595 | 0.66626 |
| PSPPH_1905 | sensory box histidine kinase/response regulator | chromosome:2209435-2213944 | 1448A-rhpS-KB | 1448A-WT-KB | OK | 95.0084 | 42.9086 | -1.14679 | -1.78168 | 0.01705 | 0.10006 |
| pilF | type IV pilus biogenesis protein PilF | chromosome:1527005-1528762 | 1448A-rhpS-KB | 1448A-WT-KB | OK | 352.402 | 158.758 | -1.15039 | -1.64916 | 0.0238 | 0.125274 |
| plsC | hdtS protein | chromosome:12961-13732 | 1448A-rhpS-KB | 1448A-WT-KB | OK | 521.213 | 234.445 | -1.15263 | -2.40853 | 0.0021 | 0.0233087 |
| PSPPH_4067 | pyridoxamine 5'-phosphate oxidase | chromosome:4649912-4650542 | 1448A-rhpS-KB | 1448A-WT-KB | OK | 169.945 | 76.4413 | -1.15264 | -1.36514 | 0.06155 | 0.227722 |
| PSPPH_2262 | sensory box histidine kinase | chromosome:2623838-2625137 | 1448A-rhpS-KB | 1448A-WT-KB | OK | 116.901 | 52.4321 | -1.15676 | -1.67747 | 0.0261 | 0.133069 |
| PSPPH_2074 | levansucrase (beta-D-fructofuranosyl transferase)(sucrose 6-fructosyl transferase) | chromosome:2433131-2434379 | 1448A-rhpS-KB | 1448A-WT-KB | OK | 45.7195 | 20.5003 | -1.15717 | -1.10428 | 0.1284 | 0.338923 |
| PSPPH_0610 | hypothetical protein | chromosome:720730-721777 | 1448A-rhpS-KB | 1448A-WT-KB | OK | 93.8319 | 42.0532 | -1.15786 | -1.35675 | 0.06285 | 0.229914 |
| PSPPH_3206 | hypothetical protein | chromosome:3715692-3716214 | 1448A-rhpS-KB | 1448A-WT-KB | OK | 804.419 | 360.145 | -1.15937 | -2.29226 | 0.0029 | 0.0295288 |
| PSPPH_4848 | hypothetical protein | chromosome:5510105-5510603 | 1448A-rhpS-KB | 1448A-WT-KB | NOTEST | 28.3269 | 12.6766 | -1.16001 | 0 | 1 | 1 |
| PSPPH_1551 | hypothetical protein | chromosome:1803797-1804247 | 1448A-rhpS-KB | 1448A-WT-KB | OK | 186.294 | 83.3624 | -1.16011 | -1.12562 | 0.13485 | 0.349495 |
| PSPPH_2890 | transporter | chromosome:3339870-3340964 | 1448A-rhpS-KB | 1448A-WT-KB | OK | 366.055 | 163.432 | -1.16337 | -1.50382 | 0.04435 | 0.189149 |
| rsuA | ribosomal small subunit pseudouridine synthase A | chromosome:4545915-4546608 | 1448A-rhpS-KB | 1448A-WT-KB | OK | 84.415 | 37.6748 | -1.1639 | -1.07561 | 0.14245 | 0.359545 |
| PSPPH_1517 | hypothetical protein | chromosome:1763650-1764238 | 1448A-rhpS-KB | 1448A-WT-KB | OK | 1004.66 | 448.362 | -1.16397 | -2.68271 | 0.00055 | 0.0080893 |
| PSPPH_0495 | acyl-CoA dehydrogenase | chromosome:569607-571620 | 1448A-rhpS-KB | 1448A-WT-KB | OK | 129.103 | 57.5914 | -1.1646 | -2.20724 | 0.00365 | 0.0339716 |
| PSPPH_1451 | Rhs family protein | chromosome:1692944-1693937 | 1448A-rhpS-KB | 1448A-WT-KB | NOTEST | 30.5847 | 13.6294 | -1.16609 | 0 | 1 | 1 |
| PSPPH_1351 | hypothetical protein | chromosome:1566316-1569024 | 1448A-rhpS-KB | 1448A-WT-KB | OK | 113.548 | 50.5261 | -1.16819 | -1.44155 | 0.0523 | 0.208351 |
| proX | amino acid ABC transporter permease | chromosome:1739527-1740382 | 1448A-rhpS-KB | 1448A-WT-KB | OK | 74.3183 | 32.9715 | -1.1725 | -1.11981 | 0.11805 | 0.325047 |
| PSPPH_2204 | TetR family transcriptional regulator | chromosome:2558913-2559564 | 1448A-rhpS-KB | 1448A-WT-KB | OK | 214.167 | 95.0122 | -1.17255 | -1.55643 | 0.0361 | 0.165429 |
| PSPPH_5041 | hypothetical protein | chromosome:5713032-5713689 | 1448A-rhpS-KB | 1448A-WT-KB | OK | 346.415 | 153.601 | -1.17331 | -1.93575 | 0.0117 | 0.0778693 |
| PSPPH_4179 | hypothetical protein | chromosome:4763472-4764794 | 1448A-rhpS-KB | 1448A-WT-KB | OK | 146.768 | 65.0757 | -1.17335 | -1.46071 | 0.04385 | 0.187464 |
| PSPPH_4238 | LuxR family transcriptional regulator | chromosome:4834586-4835351 | 1448A-rhpS-KB | 1448A-WT-KB | OK | 170.363 | 75.401 | -1.17596 | -1.55346 | 0.0385 | 0.173517 |
| PSPPH_1148 | hypothetical protein | chromosome:1347370-1349026 | 1448A-rhpS-KB | 1448A-WT-KB | OK | 200.069 | 88.5181 | -1.17645 | -2.39118 | 0.00215 | 0.0234271 |
| PSPPH_0426 | intracellular septation protein A | chromosome:490912-491715 | 1448A-rhpS-KB | 1448A-WT-KB | OK | 68.037 | 30.0999 | -1.17656 | -0.769029 | 0.29635 | 0.531171 |
| glpF | glycerol uptake facilitator protein | chromosome:4456058-4456961 | 1448A-rhpS-KB | 1448A-WT-KB | OK | 113.687 | 50.2342 | -1.17833 | -1.3727 | 0.0659 | 0.234822 |
| PSPPH_0778 | LuxR family transcriptional regulator | chromosome:914292-914931 | 1448A-rhpS-KB | 1448A-WT-KB | OK | 1164.41 | 513.708 | -1.18058 | -2.88768 | 0.00035 | 0.00566018 |
| PSPPH_3652 | hypothetical protein | chromosome:4195239-4195446 | 1448A-rhpS-KB | 1448A-WT-KB | OK | 476.327 | 210.047 | -1.18124 | -0.782969 | 0.29205 | 0.527431 |
| PSPPH_4047 | hypothetical protein | chromosome:4631033-4631177 | 1448A-rhpS-KB | 1448A-WT-KB | NOTEST | 289.526 | 127.381 | -1.18454 | 0 | 1 | 1 |
| PSPPH_5102 | hypothetical protein | chromosome:5791019-5791235 | 1448A-rhpS-KB | 1448A-WT-KB | OK | 6395.56 | 2811.58 | -1.18569 | -2.54073 | 0.00095 | 0.0123465 |
| PSPPH_3125 | methyl-accepting chemotaxis protein | chromosome:3626334-3628002 | 1448A-rhpS-KB | 1448A-WT-KB | NOTEST | 30.9844 | 13.6073 | -1.18716 | 0 | 1 | 1 |
| PSPPH_2207 | hypothetical protein | chromosome:2563468-2564347 | 1448A-rhpS-KB | 1448A-WT-KB | OK | 62.4761 | 27.4243 | -1.18785 | -1.03165 | 0.15265 | 0.372913 |
| PSPPH_3599 | Ser/Thr protein phosphatase | chromosome:4144427-4147158 | 1448A-rhpS-KB | 1448A-WT-KB | OK | 256.478 | 112.471 | -1.18928 | -1.76582 | 0.02095 | 0.113447 |
| cyaY | frataxin-like protein | chromosome:5677455-5677815 | 1448A-rhpS-KB | 1448A-WT-KB | OK | 386.253 | 169.21 | -1.19073 | -1.34285 | 0.0674 | 0.238267 |
| PSPPH_2711 | deoxycytidine triphosphate deaminase | chromosome:3138006-3138519 | 1448A-rhpS-KB | 1448A-WT-KB | NOTEST | 35.9847 | 15.7369 | -1.19323 | 0 | 1 | 1 |
| PSPPH_2010 | fimbrial protein | chromosome:2361083-2362199 | 1448A-rhpS-KB | 1448A-WT-KB | OK | 48.3586 | 21.1103 | -1.19583 | -1.10122 | 0.1262 | 0.336847 |
| cvpA | colicin V production protein CvpA | chromosome:1923313-1923901 | 1448A-rhpS-KB | 1448A-WT-KB | OK | 154.047 | 67.2238 | -1.19632 | -1.23039 | 0.09435 | 0.284563 |
| phnM | phosphonate metabolism protein PhnM | chromosome:3400932-3404106 | 1448A-rhpS-KB | 1448A-WT-KB | OK | 197.358 | 86.083 | -1.19701 | -1.76065 | 0.01755 | 0.10249 |
| PSPPH_1843 | hypothetical protein | chromosome:2139779-2141574 | 1448A-rhpS-KB | 1448A-WT-KB | OK | 165.77 | 72.191 | -1.19929 | -2.18778 | 0.0037 | 0.0342585 |
| PSPPH_4051 | hypothetical protein | chromosome:4633842-4637746 | 1448A-rhpS-KB | 1448A-WT-KB | OK | 59.148 | 25.7386 | -1.2004 | -0.347108 | 0.604 | 0.779594 |
| cysI | sulfite reductase (NADPH) hemoprotein subunit beta | chromosome:3016155-3018292 | 1448A-rhpS-KB | 1448A-WT-KB | OK | 2189.16 | 950.186 | -1.20409 | -4.44562 | 0.00005 | 0.00121565 |
| PSPPH_4612 | biotin--protein ligase | chromosome:5245138-5246837 | 1448A-rhpS-KB | 1448A-WT-KB | OK | 662.817 | 287.644 | -1.20433 | -2.76791 | 0.0005 | 0.00757203 |
| rplU | 50S ribosomal protein L21 | chromosome:836975-837287 | 1448A-rhpS-KB | 1448A-WT-KB | OK | 3800.21 | 1649.05 | -1.20444 | -3.02911 | 0.0001 | 0.00221988 |
| PSPPH_0238 | hypothetical protein | chromosome:275078-275426 | 1448A-rhpS-KB | 1448A-WT-KB | OK | 84.9922 | 36.8755 | -1.20467 | -0.609526 | 0.39035 | 0.619107 |
| PSPPH_4287 | hypothetical protein | chromosome:4893051-4893381 | 1448A-rhpS-KB | 1448A-WT-KB | OK | 500.842 | 216.951 | -1.20698 | -1.41704 | 0.05485 | 0.213312 |
| PSPPH_2447 | urea amidolyase | chromosome:2827156-2829000 | 1448A-rhpS-KB | 1448A-WT-KB | OK | 73.8277 | 31.8568 | -1.21256 | -0.995591 | 0.1864 | 0.415074 |
| vfr | cAMP-regulatory protein | chromosome:791996-792641 | 1448A-rhpS-KB | 1448A-WT-KB | OK | 44.6401 | 19.2483 | -1.21361 | -0.718088 | 0.3295 | 0.565769 |
| apaH | diadenosine tetraphosphatase | chromosome:746722-748370 | 1448A-rhpS-KB | 1448A-WT-KB | OK | 436.775 | 188.31 | -1.21378 | -2.09732 | 0.00515 | 0.0434106 |
| PSPPH_4538 | transposon Tn7-like transposase A | chromosome:5172523-5173330 | 1448A-rhpS-KB | 1448A-WT-KB | OK | 1136.93 | 490.079 | -1.21406 | -3.37468 | 0.00005 | 0.00121565 |
| pyrF | orotidine 5'-phosphate decarboxylase | chromosome:2101451-2102228 | 1448A-rhpS-KB | 1448A-WT-KB | OK | 366.389 | 157.864 | -1.2147 | -2.16482 | 0.0052 | 0.0434224 |
| PSPPH_2610 | hypothetical protein | chromosome:3008232-3009816 | 1448A-rhpS-KB | 1448A-WT-KB | OK | 123.591 | 53.1849 | -1.21649 | -1.06786 | 0.1327 | 0.346688 |
| PSPPH_1425 | acyl dehydratase | chromosome:1653812-1654379 | 1448A-rhpS-KB | 1448A-WT-KB | OK | 808.119 | 346.254 | -1.22274 | -2.56059 | 0.00095 | 0.0123465 |
| PSPPH_0458 | hypothetical protein | chromosome:521613-522069 | 1448A-rhpS-KB | 1448A-WT-KB | OK | 158.853 | 68.054 | -1.22294 | -1.07547 | 0.1451 | 0.362902 |
| pnlA | pectin lyase | chromosome:4556706-4557963 | 1448A-rhpS-KB | 1448A-WT-KB | OK | 567.438 | 243.084 | -1.22301 | -3.1922 | 0.00005 | 0.00121565 |
| nirD | nitrite reductase [NAD(P)H] small subunit | chromosome:3498237-3498624 | 1448A-rhpS-KB | 1448A-WT-KB | NOTEST | 31.1479 | 13.3277 | -1.22471 | 0 | 1 | 1 |
| PSPPH_0658 | hypothetical protein | chromosome:774747-775847 | 1448A-rhpS-KB | 1448A-WT-KB | OK | 65.3063 | 27.916 | -1.22613 | -0.610576 | 0.57305 | 0.759956 |
| PSPPH_0387 | nuclease-like protein | chromosome:441676-442396 | 1448A-rhpS-KB | 1448A-WT-KB | OK | 465.579 | 198.351 | -1.23097 | -2.2847 | 0.0031 | 0.0310347 |
| PSPPH_1152 | SAM-dependent methyltransferase | chromosome:1351679-1352666 | 1448A-rhpS-KB | 1448A-WT-KB | OK | 172.837 | 73.6276 | -1.2311 | -1.85977 | 0.01495 | 0.0918064 |
| PSPPH_1045 | alpha/beta hydrolase | chromosome:1237443-1238223 | 1448A-rhpS-KB | 1448A-WT-KB | OK | 46.4123 | 19.759 | -1.232 | -0.860877 | 0.23465 | 0.4693 |
| dnaB | replicative DNA helicase | chromosome:679532-680927 | 1448A-rhpS-KB | 1448A-WT-KB | OK | 377.693 | 160.299 | -1.23645 | -2.94915 | 0.00005 | 0.00121565 |
| PSPPH_3205 | hypothetical protein | chromosome:3715397-3715631 | 1448A-rhpS-KB | 1448A-WT-KB | OK | 483.797 | 204.649 | -1.24125 | -0.948828 | 0.1837 | 0.412402 |
| PSPPH_2658 | hypothetical protein | chromosome:3080758-3081088 | 1448A-rhpS-KB | 1448A-WT-KB | OK | 327.156 | 138.175 | -1.24348 | -1.18831 | 0.10375 | 0.30039 |
| moaA | molybdenum cofactor biosynthesis protein A | chromosome:3288245-3289244 | 1448A-rhpS-KB | 1448A-WT-KB | OK | 160.99 | 67.9132 | -1.24521 | -1.81258 | 0.01805 | 0.10405 |
| PSPPH_0922 | hypothetical protein | chromosome:1097767-1098675 | 1448A-rhpS-KB | 1448A-WT-KB | OK | 1071.85 | 452.064 | -1.24551 | -2.02063 | 0.00555 | 0.0457995 |
| PSPPH_1823 | hypothetical protein | chromosome:2123177-2123369 | 1448A-rhpS-KB | 1448A-WT-KB | OK | 932.418 | 393.075 | -1.24617 | -0.958426 | 0.1833 | 0.412402 |
| PSPPH_2745 | dipeptide transporter dppD-like protein | chromosome:3175454-3178867 | 1448A-rhpS-KB | 1448A-WT-KB | NOTEST | 28.3188 | 11.93 | -1.24716 | 0 | 1 | 1 |
| PSPPH_4539 | transposon Tn7-like transposase B | chromosome:5173331-5175449 | 1448A-rhpS-KB | 1448A-WT-KB | OK | 542.99 | 227.751 | -1.25347 | -3.7994 | 0.00005 | 0.00121565 |
| PSPPH_1102 | fusaric acid resistance protein | chromosome:1295168-1299907 | 1448A-rhpS-KB | 1448A-WT-KB | OK | 48.9003 | 20.5004 | -1.25419 | -1.32004 | 0.07035 | 0.244345 |
| metF | 5,10-methylenetetrahydrofolate reductase | chromosome:512789-513665 | 1448A-rhpS-KB | 1448A-WT-KB | OK | 541.869 | 227.116 | -1.25451 | -2.80797 | 0.00065 | 0.00925538 |
| PSPPH_3403 | hypothetical protein | chromosome:3936649-3937838 | 1448A-rhpS-KB | 1448A-WT-KB | NOTEST | 43.0656 | 18.0114 | -1.25763 | 0 | 1 | 1 |
| PSPPH_1888 | hypothetical protein | chromosome:2190514-2190874 | 1448A-rhpS-KB | 1448A-WT-KB | OK | 83.1386 | 34.5739 | -1.26584 | -0.73228 | 0.41355 | 0.641382 |
| mexT | transcriptional regulator MexT | chromosome:2677224-2678139 | 1448A-rhpS-KB | 1448A-WT-KB | OK | 240.735 | 100.029 | -1.26703 | -2.1836 | 0.0065 | 0.0507227 |
| PSPPH_3674 | hypothetical protein | chromosome:4221050-4221836 | 1448A-rhpS-KB | 1448A-WT-KB | OK | 685.084 | 283.062 | -1.27516 | -2.86085 | 0.00045 | 0.00699261 |
| PSPPH_3263 | acetyltransferase | chromosome:3782161-3782644 | 1448A-rhpS-KB | 1448A-WT-KB | OK | 422.677 | 174.375 | -1.27736 | -1.81331 | 0.01805 | 0.10405 |
| PSPPH_1681 | ABC transporter ATP-binding protein | chromosome:1945327-1947537 | 1448A-rhpS-KB | 1448A-WT-KB | NOTEST | 20.8254 | 8.59121 | -1.27741 | 0 | 1 | 1 |
| PSPPH_0795 | secretion protein | chromosome:940272-943330 | 1448A-rhpS-KB | 1448A-WT-KB | NOTEST | 31.5652 | 13.0071 | -1.27904 | 0 | 1 | 1 |
| PSPPH_4237 | peptide ABC transporter permease | chromosome:4830473-4834247 | 1448A-rhpS-KB | 1448A-WT-KB | OK | 99.7926 | 41.0439 | -1.28177 | -1.19122 | 0.108 | 0.3083 |
| rpmE | 50S ribosomal protein L31 | chromosome:441390-441612 | 1448A-rhpS-KB | 1448A-WT-KB | OK | 4864.77 | 1999.4 | -1.2828 | -2.50006 | 0.00185 | 0.0213977 |
| PSPPH_1684 | ABC transporter permease | chromosome:1948406-1949207 | 1448A-rhpS-KB | 1448A-WT-KB | OK | 85.6377 | 35.1471 | -1.28484 | -1.18969 | 0.1025 | 0.298805 |
| PSPPH_4050 | hypothetical protein | chromosome:4633179-4633761 | 1448A-rhpS-KB | 1448A-WT-KB | OK | 126.081 | 51.7207 | -1.28554 | -1.23777 | 0.09665 | 0.287856 |
| PSPPH_3487 | transcriptional regulator | chromosome:4026795-4027359 | 1448A-rhpS-KB | 1448A-WT-KB | OK | 67.0306 | 27.445 | -1.28828 | -0.8293 | 0.23615 | 0.470195 |
| mltD | membrane-bound lytic murein transglycosylase D | chromosome:1973984-1979391 | 1448A-rhpS-KB | 1448A-WT-KB | OK | 361.615 | 147.991 | -1.28894 | -2.62674 | 0.00125 | 0.0152997 |
| PSPPH_1244 | AsnC family transcriptional regulator | chromosome:1451584-1452064 | 1448A-rhpS-KB | 1448A-WT-KB | OK | 140.637 | 57.48 | -1.29084 | -1.10449 | 0.13135 | 0.343916 |
| PSPPH_3262 | hypothetical protein | chromosome:3781441-3781702 | 1448A-rhpS-KB | 1448A-WT-KB | OK | 1007.95 | 411.458 | -1.2926 | -1.603 | 0.02905 | 0.142421 |
| PSPPH_1717 | hypothetical protein | chromosome:1984977-1985337 | 1448A-rhpS-KB | 1448A-WT-KB | OK | 297.688 | 121.483 | -1.29305 | -1.24148 | 0.0905 | 0.279798 |
| PSPPH_0665 | prophage PSPPH01, chitinase | chromosome:781546-782092 | 1448A-rhpS-KB | 1448A-WT-KB | OK | 101.313 | 41.232 | -1.29698 | -1.03023 | 0.16415 | 0.391899 |
| PSPPH_2619 | sugar-binding domain-containing protein | chromosome:3018874-3019948 | 1448A-rhpS-KB | 1448A-WT-KB | OK | 51.4551 | 20.9344 | -1.29744 | -1.13284 | 0.12065 | 0.329163 |
| PSPPH_1530 | polygalacturonase | chromosome:1784948-1785383 | 1448A-rhpS-KB | 1448A-WT-KB | OK | 73.4717 | 29.8235 | -1.30074 | -0.699661 | 0.3299 | 0.565769 |
| accD | acetyl-CoA carboxylase subunit beta | chromosome:1920330-1923195 | 1448A-rhpS-KB | 1448A-WT-KB | OK | 656.301 | 265.956 | -1.30317 | -2.14181 | 0.00365 | 0.0339716 |
| wspC | chemotaxis protein methyltransferase WspC | chromosome:4430452-4436346 | 1448A-rhpS-KB | 1448A-WT-KB | OK | 157.837 | 63.9044 | -1.30445 | -1.26925 | 0.09875 | 0.291641 |
| PSPPH_2627 | 3-methylcrotonyl-CoA carboxylase subunit beta | chromosome:3031073-3032681 | 1448A-rhpS-KB | 1448A-WT-KB | OK | 89.8187 | 36.3544 | -1.30489 | -1.86862 | 0.0152 | 0.0931815 |
| PSPPH_2661 | amino acid ABC transporter permease | chromosome:3083528-3084191 | 1448A-rhpS-KB | 1448A-WT-KB | OK | 96.8261 | 39.179 | -1.30532 | -1.10349 | 0.12835 | 0.338923 |
| cysC2 | adenylylsulfate kinase | chromosome:5877884-5878487 | 1448A-rhpS-KB | 1448A-WT-KB | OK | 179.688 | 72.7023 | -1.30542 | -1.47532 | 0.0517 | 0.207814 |
| PSPPH_0484 | chemotaxis protein CheW | chromosome:545474-551898 | 1448A-rhpS-KB | 1448A-WT-KB | OK | 73.3887 | 29.6426 | -1.30789 | -0.570648 | 0.5013 | 0.708441 |
| PSPPH_3629 | glutathione peroxidase | chromosome:4173764-4174334 | 1448A-rhpS-KB | 1448A-WT-KB | OK | 435.444 | 175.077 | -1.3145 | -2.17114 | 0.00635 | 0.05021 |
| PSPPH_2784 | dipeptide transport system permease DppC | chromosome:3222221-3224038 | 1448A-rhpS-KB | 1448A-WT-KB | NOTEST | 28.5753 | 11.484 | -1.31514 | 0 | 1 | 1 |
| metK | S-adenosylmethionine synthetase | chromosome:5475420-5476611 | 1448A-rhpS-KB | 1448A-WT-KB | OK | 815.648 | 327.142 | -1.31803 | -3.75079 | 0.00005 | 0.00121565 |
| PSPPH_3586 | BNR/Asp-box repeat-containing protein | chromosome:4132026-4133076 | 1448A-rhpS-KB | 1448A-WT-KB | OK | 87.3286 | 34.9838 | -1.31977 | -1.45632 | 0.0538 | 0.211298 |
| PSPPH_2429 | hypothetical protein | chromosome:2809329-2810277 | 1448A-rhpS-KB | 1448A-WT-KB | NOTEST | 31.1654 | 12.4701 | -1.32147 | 0 | 1 | 1 |
| pchB | isochorismate-pyruvate lyase | chromosome:3371026-3372738 | 1448A-rhpS-KB | 1448A-WT-KB | NOTEST | 35.6293 | 14.2549 | -1.32161 | 0 | 1 | 1 |
| PSPPH_4629 | cpaA protein | chromosome:5266390-5267488 | 1448A-rhpS-KB | 1448A-WT-KB | NOTEST | 42.3093 | 16.9211 | -1.32215 | 0 | 1 | 1 |
| rimI | ribosomal-protein-alanine acetyltransferase | chromosome:1060302-1061551 | 1448A-rhpS-KB | 1448A-WT-KB | OK | 233.365 | 93.1407 | -1.32511 | -1.23256 | 0.09035 | 0.279577 |
| PSPPH_3224 | polysaccharide biosynthesis/export protein | chromosome:3736559-3737339 | 1448A-rhpS-KB | 1448A-WT-KB | OK | 124.961 | 49.766 | -1.32825 | -1.48345 | 0.05195 | 0.208051 |
| PSPPH_4215 | hypothetical protein | chromosome:4803942-4804227 | 1448A-rhpS-KB | 1448A-WT-KB | OK | 336.209 | 133.891 | -1.3283 | -1.04556 | 0.15165 | 0.37173 |
| PSPPH_2142 | MerR family transcriptional regulator | chromosome:2497807-2498447 | 1448A-rhpS-KB | 1448A-WT-KB | OK | 1354.06 | 537.804 | -1.33214 | -2.1727 | 0.00495 | 0.0422227 |
| PSPPH_1516 | hypothetical protein | chromosome:1763235-1763640 | 1448A-rhpS-KB | 1448A-WT-KB | OK | 112.778 | 44.6904 | -1.33544 | -0.843548 | 0.217 | 0.453543 |
| PSPPH_1030 | hypothetical protein | chromosome:1220271-1220523 | 1448A-rhpS-KB | 1448A-WT-KB | OK | 261.238 | 102.995 | -1.34279 | -0.802502 | 0.2756 | 0.510664 |
| PSPPH_1867 | response regulator | chromosome:2167352-2169211 | 1448A-rhpS-KB | 1448A-WT-KB | OK | 720.372 | 283.969 | -1.34301 | -3.40479 | 0.00005 | 0.00121565 |
| PSPPH_2032 | Slt family transglycosylase | chromosome:2385633-2387055 | 1448A-rhpS-KB | 1448A-WT-KB | OK | 93.9862 | 36.9697 | -1.3461 | -1.81925 | 0.01975 | 0.109098 |
| PSPPH_1534 | PerM family membrane protein | chromosome:1788799-1789870 | 1448A-rhpS-KB | 1448A-WT-KB | OK | 217.064 | 85.3056 | -1.34741 | -2.11896 | 0.0069 | 0.0530335 |
| PSPPH_2027 | CHAD domain-containing superfamily | chromosome:2380994-2381756 | 1448A-rhpS-KB | 1448A-WT-KB | OK | 212.737 | 83.5694 | -1.34803 | -1.90068 | 0.01375 | 0.0865185 |
| PSPPH_2891 | Sco1/SenC family protein | chromosome:3339870-3340964 | 1448A-rhpS-KB | 1448A-WT-KB | OK | 215.334 | 84.3648 | -1.35186 | -1.55183 | 0.04155 | 0.181758 |
| PSPPH_0921 | hypothetical protein | chromosome:1097767-1098675 | 1448A-rhpS-KB | 1448A-WT-KB | OK | 938.731 | 367.354 | -1.35354 | -2.17874 | 0.0046 | 0.0400985 |
| cheR3 | chemotaxis protein CheR | chromosome:3945032-3945860 | 1448A-rhpS-KB | 1448A-WT-KB | OK | 513.235 | 200.839 | -1.35358 | -2.76142 | 0.0007 | 0.00962231 |
| PSPPH_0499 | response regulator/sensory box/GGDEF domain/EAL domain-containing protein | chromosome:578610-580749 | 1448A-rhpS-KB | 1448A-WT-KB | OK | 144.933 | 56.5353 | -1.35816 | -2.59145 | 0.0014 | 0.0168471 |
| PSPPH_1971 | proteinase inhibitor | chromosome:2319317-2319575 | 1448A-rhpS-KB | 1448A-WT-KB | OK | 541.601 | 211.213 | -1.35853 | -1.24187 | 0.10285 | 0.299093 |
| PSPPH_1840 | lipoprotein | chromosome:2137192-2137723 | 1448A-rhpS-KB | 1448A-WT-KB | NOTEST | 37.6433 | 14.6719 | -1.35934 | 0 | 1 | 1 |
| PSPPH_2702 | Ser/Thr protein phosphatase | chromosome:3129621-3130419 | 1448A-rhpS-KB | 1448A-WT-KB | OK | 64.1759 | 24.9963 | -1.36032 | -1.12248 | 0.1394 | 0.354997 |
| PSPPH_1464 | lipoprotein | chromosome:1707098-1707962 | 1448A-rhpS-KB | 1448A-WT-KB | OK | 133.269 | 51.6759 | -1.36677 | -1.6643 | 0.03075 | 0.14732 |
| ispB | octylprenyl diphosphate synthase | chromosome:835767-836736 | 1448A-rhpS-KB | 1448A-WT-KB | OK | 846.881 | 328.004 | -1.36844 | -3.61234 | 0.00005 | 0.00121565 |
| PSPPH_1696 | hypothetical protein | chromosome:1960204-1960711 | 1448A-rhpS-KB | 1448A-WT-KB | OK | 150.494 | 58.2439 | -1.36953 | -1.18942 | 0.1055 | 0.303833 |
| PSPPH_1171 | hypothetical protein | chromosome:1375356-1376426 | 1448A-rhpS-KB | 1448A-WT-KB | OK | 93.7188 | 36.2505 | -1.37034 | -0.867093 | 0.23575 | 0.470184 |
| PSPPH_3230 | hypothetical protein | chromosome:3741843-3746385 | 1448A-rhpS-KB | 1448A-WT-KB | OK | 83.6443 | 32.2954 | -1.37294 | -1.34171 | 0.07675 | 0.258534 |
| PSPPH_1771 | GntR family transcriptional regulator | chromosome:2068453-2069206 | 1448A-rhpS-KB | 1448A-WT-KB | OK | 274.674 | 105.958 | -1.37422 | -2.08784 | 0.0059 | 0.0474923 |
| PSPPH_3928 | ABC transporter binding protein | chromosome:4489316-4491813 | 1448A-rhpS-KB | 1448A-WT-KB | OK | 173.106 | 66.7724 | -1.37433 | -2.03454 | 0.00985 | 0.0686236 |
| fliT | motility-like protein FliT | chromosome:3916986-3917283 | 1448A-rhpS-KB | 1448A-WT-KB | OK | 331.013 | 127.657 | -1.37462 | -1.12508 | 0.13575 | 0.350557 |
| PSPPH_3225 | exopolysaccharide biosynthesis protein | chromosome:3737356-3740526 | 1448A-rhpS-KB | 1448A-WT-KB | OK | 94.3054 | 36.2176 | -1.38065 | -2.05074 | 0.0091 | 0.0647876 |
| PSPPH_1377 | (Fe-S)-binding protein | chromosome:1596918-1598396 | 1448A-rhpS-KB | 1448A-WT-KB | OK | 141.093 | 54.1229 | -1.38233 | -1.53793 | 0.0418 | 0.182187 |
| PSPPH_3420 | aminotransferase | chromosome:3952665-3954509 | 1448A-rhpS-KB | 1448A-WT-KB | NOTEST | 42.9902 | 16.4901 | -1.38241 | 0 | 1 | 1 |
| ftsL | cell division protein FtsL | chromosome:4697111-4703978 | 1448A-rhpS-KB | 1448A-WT-KB | OK | 609.619 | 233.816 | -1.38253 | -0.838779 | 0.23685 | 0.470315 |
| iscR | iron-sulfur cluster assembly transcription factor IscR | chromosome:1519658-1520150 | 1448A-rhpS-KB | 1448A-WT-KB | OK | 264.41 | 101.409 | -1.38259 | -1.55463 | 0.0429 | 0.184951 |
| PSPPH_2496 | NAD-dependent deacetylase | chromosome:2881105-2884311 | 1448A-rhpS-KB | 1448A-WT-KB | OK | 80.2252 | 30.7091 | -1.38539 | -1.04213 | 0.1747 | 0.404128 |
| PSPPH_2337 | Fis family transcriptional regulator | chromosome:2710267-2712199 | 1448A-rhpS-KB | 1448A-WT-KB | OK | 296.888 | 113.607 | -1.38587 | -3.33035 | 0.00005 | 0.00121565 |
| gcvH2 | glycine cleavage system protein H | chromosome:1366662-1367046 | 1448A-rhpS-KB | 1448A-WT-KB | OK | 669.117 | 254.76 | -1.39312 | -2.01839 | 0.0087 | 0.0633275 |
| PSPPH_0266 | lipoprotein | chromosome:307264-307981 | 1448A-rhpS-KB | 1448A-WT-KB | OK | 420.288 | 159.848 | -1.39468 | -2.49751 | 0.00195 | 0.0221952 |
| PSPPH_2942 | signal transduction protein | chromosome:3413952-3414780 | 1448A-rhpS-KB | 1448A-WT-KB | NOTEST | 46.0342 | 17.5045 | -1.39498 | 0 | 1 | 1 |
| ihfA | integration host factor subunit alpha | chromosome:2497807-2498447 | 1448A-rhpS-KB | 1448A-WT-KB | OK | 3171.87 | 1206.11 | -1.39498 | -2.84432 | 0.00035 | 0.00566018 |
| bfr1 | bacterioferritin | chromosome:4449293-4449764 | 1448A-rhpS-KB | 1448A-WT-KB | OK | 3079.26 | 1168.7 | -1.39768 | -4.02044 | 0.00005 | 0.00121565 |
| PSPPH_2855 | hypothetical protein | chromosome:3298618-3299758 | 1448A-rhpS-KB | 1448A-WT-KB | OK | 46.9901 | 17.795 | -1.40089 | -1.17452 | 0.1013 | 0.296323 |
| PSPPH_2870 | major facilitator family transporter | chromosome:3314497-3315916 | 1448A-rhpS-KB | 1448A-WT-KB | OK | 68.7534 | 25.9759 | -1.40426 | -1.57318 | 0.05035 | 0.204693 |
| PSPPH_1467 | hypothetical protein | chromosome:1709584-1709773 | 1448A-rhpS-KB | 1448A-WT-KB | OK | 795.895 | 299.031 | -1.41228 | -0.949715 | 0.20435 | 0.439703 |
| PSPPH_3930 | hypothetical protein | chromosome:4489316-4491813 | 1448A-rhpS-KB | 1448A-WT-KB | OK | 391.028 | 146.438 | -1.41699 | -1.02942 | 0.17955 | 0.409778 |
| PSPPH_3101 | mutT/nudix family protein | chromosome:3599599-3600046 | 1448A-rhpS-KB | 1448A-WT-KB | OK | 281.256 | 105.294 | -1.41745 | -1.58694 | 0.0384 | 0.173285 |
| PSPPH_1348 | hypothetical protein | chromosome:1564363-1564975 | 1448A-rhpS-KB | 1448A-WT-KB | OK | 78.2635 | 29.2853 | -1.41817 | -1.0148 | 0.1818 | 0.411497 |
| PSPPH_4702 | carbon-nitrogen family hydrolase | chromosome:5337982-5338777 | 1448A-rhpS-KB | 1448A-WT-KB | OK | 770.629 | 287.511 | -1.42242 | -3.29281 | 0.00005 | 0.00121565 |
| PSPPH_3940 | hypothetical protein | chromosome:4499907-4503029 | 1448A-rhpS-KB | 1448A-WT-KB | NOTEST | 19.8916 | 7.41175 | -1.42427 | 0 | 1 | 1 |
| PSPPH_3704 | NLP/P60 family protein | chromosome:4251421-4251967 | 1448A-rhpS-KB | 1448A-WT-KB | OK | 180.619 | 67.2868 | -1.42456 | -1.39006 | 0.0681 | 0.239793 |
| PSPPH_1827 | hypothetical protein | chromosome:2127446-2127788 | 1448A-rhpS-KB | 1448A-WT-KB | OK | 672.799 | 250.328 | -1.42636 | -1.87789 | 0.0154 | 0.0936048 |
| ccoQ | cytochrome c oxidase, cbb3-type subunit IV | chromosome:3868112-3869278 | 1448A-rhpS-KB | 1448A-WT-KB | OK | 172.535 | 64.1109 | -1.42825 | -0.953943 | 0.5496 | 0.746303 |
| PSPPH_4871 | hypothetical protein | chromosome:5539519-5539969 | 1448A-rhpS-KB | 1448A-WT-KB | OK | 129.72 | 48.1616 | -1.42945 | -1.11914 | 0.13 | 0.341513 |
| PSPPH_2384 | isochorismatase | chromosome:2765334-2766093 | 1448A-rhpS-KB | 1448A-WT-KB | OK | 129.317 | 47.963 | -1.43092 | -1.55652 | 0.0387 | 0.17398 |
| PSPPH_3393 | 3-oxoacyl-ACP synthase | chromosome:3921036-3921963 | 1448A-rhpS-KB | 1448A-WT-KB | OK | 690.796 | 256.077 | -1.43168 | -3.43956 | 0.00005 | 0.00121565 |
| PSPPH_3223 | mannose-1-phosphate guanylyltransferase | chromosome:3733534-3736428 | 1448A-rhpS-KB | 1448A-WT-KB | OK | 107.719 | 39.8838 | -1.43339 | -1.85207 | 0.01365 | 0.0864984 |
| PSPPH_0666 | hypothetical protein | chromosome:782103-782598 | 1448A-rhpS-KB | 1448A-WT-KB | OK | 91.7675 | 33.9344 | -1.43523 | -0.928162 | 0.1835 | 0.412402 |
| fpr1 | ferredoxin-NADP reductase | chromosome:4349022-4349802 | 1448A-rhpS-KB | 1448A-WT-KB | OK | 4692.83 | 1727.55 | -1.44173 | -5.25378 | 0.00005 | 0.00121565 |
| PSPPH_3931 | 3-oxoacyl-ACP reductase | chromosome:4491835-4492585 | 1448A-rhpS-KB | 1448A-WT-KB | OK | 238.452 | 87.7121 | -1.44285 | -2.01723 | 0.01205 | 0.0797531 |
| PSPPH_1420 | major facilitator family transporter | chromosome:1646571-1647951 | 1448A-rhpS-KB | 1448A-WT-KB | NOTEST | 38.4288 | 14.1228 | -1.44416 | 0 | 1 | 1 |
| PSPPH_4850 | hypothetical protein | chromosome:5511365-5512531 | 1448A-rhpS-KB | 1448A-WT-KB | OK | 107.804 | 39.4977 | -1.44858 | -1.04895 | 0.1168 | 0.322849 |
| pilR | type IV fimbriae expression regulatory protein pilR | chromosome:861693-863031 | 1448A-rhpS-KB | 1448A-WT-KB | OK | 220.369 | 80.703 | -1.44922 | -2.71862 | 0.0011 | 0.0137462 |
| PSPPH_3620 | hypothetical protein | chromosome:4167929-4168352 | 1448A-rhpS-KB | 1448A-WT-KB | OK | 112.363 | 41.129 | -1.44994 | -0.926603 | 0.18545 | 0.414249 |
| PSPPH_3227 | glycosyl hydrolase | chromosome:3740527-3741841 | 1448A-rhpS-KB | 1448A-WT-KB | OK | 82.5069 | 30.1839 | -1.45073 | -1.74373 | 0.02395 | 0.125694 |
| PSPPH_2801 | hypothetical protein | chromosome:3242926-3246649 | 1448A-rhpS-KB | 1448A-WT-KB | OK | 45.6034 | 16.6779 | -1.4512 | -2.17168 | 0.0065 | 0.0507227 |
| PSPPH_2113 | glycosyl transferase family protein | chromosome:2472386-2473313 | 1448A-rhpS-KB | 1448A-WT-KB | OK | 453.191 | 165.72 | -1.45137 | -2.97808 | 0.0005 | 0.00757203 |
| glnL | nitrogen regulation protein NR(II) | chromosome:5512814-5515333 | 1448A-rhpS-KB | 1448A-WT-KB | OK | 110.167 | 40.1249 | -1.45713 | -1.52225 | 0.05335 | 0.210688 |
| PSPPH_4822 | ArsR family transcriptional regulator | chromosome:5474404-5475400 | 1448A-rhpS-KB | 1448A-WT-KB | OK | 91.1416 | 33.1418 | -1.45946 | -1.57032 | 0.0454 | 0.192674 |
| rpsU | 30S ribosomal protein S21 | chromosome:737152-737368 | 1448A-rhpS-KB | 1448A-WT-KB | OK | 6449.63 | 2344.77 | -1.45977 | -3.06544 | 0.0002 | 0.00374241 |
| PSPPH_4902 | molybdenum-pterin-binding domain-containing protein | chromosome:5576114-5576330 | 1448A-rhpS-KB | 1448A-WT-KB | OK | 118.79 | 43.0448 | -1.4645 | -1.07548 | 0.54065 | 0.73864 |
| PSPPH_5003 | hypothetical protein | chromosome:5672266-5672596 | 1448A-rhpS-KB | 1448A-WT-KB | OK | 2844.27 | 1030.64 | -1.46451 | -3.39693 | 0.0001 | 0.00221988 |
| accA | acetyl-CoA carboxylase carboxyltransferase subunit alpha | chromosome:4376285-4377233 | 1448A-rhpS-KB | 1448A-WT-KB | OK | 1437.06 | 520.173 | -1.46606 | -4.38882 | 0.00005 | 0.00121565 |
| secG | preprotein translocase subunit SecG | chromosome:4781057-4781438 | 1448A-rhpS-KB | 1448A-WT-KB | OK | 1709.09 | 615.65 | -1.47304 | -3.00518 | 0.00035 | 0.00566018 |
| PSPPH_3971 | ABC transporter substrate-binding protein | chromosome:4531763-4532831 | 1448A-rhpS-KB | 1448A-WT-KB | NOTEST | 22.755 | 8.17321 | -1.47721 | 0 | 1 | 1 |
| hemN | coproporphyrinogen III oxidase | chromosome:3877632-3879015 | 1448A-rhpS-KB | 1448A-WT-KB | OK | 968.393 | 347.515 | -1.47852 | -4.53134 | 0.00005 | 0.00121565 |
| PSPPH_1200 | pili assembly chaperone | chromosome:1410071-1410833 | 1448A-rhpS-KB | 1448A-WT-KB | NOTEST | 33.6999 | 12.0743 | -1.4808 | 0 | 1 | 1 |
| PSPPH_4360 | hypothetical protein | chromosome:4982105-4982768 | 1448A-rhpS-KB | 1448A-WT-KB | OK | 165.278 | 59.2012 | -1.4812 | -1.62215 | 0.03635 | 0.165708 |
| PSPPH_3294 | DNA-binding heavy metal response regulator | chromosome:3817917-3819974 | 1448A-rhpS-KB | 1448A-WT-KB | OK | 58.0726 | 20.7764 | -1.48291 | -0.918364 | 0.23295 | 0.468459 |
| PSPPH_4701 | tryptophan synthase subunit alpha | chromosome:5336333-5337968 | 1448A-rhpS-KB | 1448A-WT-KB | OK | 1165.85 | 416.1 | -1.48637 | -4.95719 | 0.00005 | 0.00121565 |
| PSPPH_0156 | hypothetical protein | chromosome:184190-184721 | 1448A-rhpS-KB | 1448A-WT-KB | OK | 166.178 | 59.2278 | -1.48838 | -1.36458 | 0.0793 | 0.261215 |
| motB | flagellar motor protein MotB | chromosome:653114-654140 | 1448A-rhpS-KB | 1448A-WT-KB | OK | 78.0626 | 27.7558 | -1.49184 | -1.57 | 0.0464 | 0.194869 |
| PSPPH_2250 | hypothetical protein | chromosome:2613335-2613509 | 1448A-rhpS-KB | 1448A-WT-KB | OK | 1203.6 | 427.546 | -1.4932 | -1.01717 | 0.17195 | 0.399953 |
| PSPPH_3675 | sensory box protein | chromosome:4221865-4224328 | 1448A-rhpS-KB | 1448A-WT-KB | OK | 140.554 | 49.9217 | -1.49339 | -2.91427 | 0.00025 | 0.0043799 |
| PSPPH_4634 | bmp family protein | chromosome:5271688-5272783 | 1448A-rhpS-KB | 1448A-WT-KB | NOTEST | 28.484 | 10.1139 | -1.4938 | 0 | 1 | 1 |
| PSPPH_0237 | type IV pilus biogenesis protein | chromosome:273183-274968 | 1448A-rhpS-KB | 1448A-WT-KB | OK | 194.709 | 68.7439 | -1.50201 | -3.00259 | 0.0003 | 0.0050338 |
| PSPPH_3241 | LysR family transcriptional regulator | chromosome:3755659-3756595 | 1448A-rhpS-KB | 1448A-WT-KB | OK | 416.236 | 146.475 | -1.50674 | -2.97296 | 0.00025 | 0.0043799 |
| PSPPH_1297 | serine-aspartate repeat-containing protein | chromosome:1510065-1510434 | 1448A-rhpS-KB | 1448A-WT-KB | OK | 339.432 | 119.386 | -1.5075 | -1.53026 | 0.0449 | 0.191039 |
| PSPPH_1835 | hypothetical protein | chromosome:2135176-2135470 | 1448A-rhpS-KB | 1448A-WT-KB | OK | 580.792 | 203.966 | -1.5097 | -1.57462 | 0.0456 | 0.192869 |
| nusG | transcription antitermination protein NusG | chromosome:5243076-5243610 | 1448A-rhpS-KB | 1448A-WT-KB | OK | 3270.59 | 1145.63 | -1.51341 | -4.577 | 0.00005 | 0.00121565 |
| PSPPH_0155 | OprD family outer membrane porin | chromosome:182882-184157 | 1448A-rhpS-KB | 1448A-WT-KB | OK | 552.846 | 193.408 | -1.51523 | -3.78093 | 0.00005 | 0.00121565 |
| PSPPH_0532 | small multidrug resistance (SMR) family protein | chromosome:625159-625492 | 1448A-rhpS-KB | 1448A-WT-KB | OK | 204.368 | 71.2375 | -1.52046 | -1.35479 | 0.1656 | 0.392998 |
| PSPPH_3861 | hypothetical protein | chromosome:4416378-4416690 | 1448A-rhpS-KB | 1448A-WT-KB | OK | 860.947 | 299.801 | -1.52192 | -1.97858 | 0.01215 | 0.0802664 |
| PSPPH_0728 | hypothetical protein | chromosome:849522-854910 | 1448A-rhpS-KB | 1448A-WT-KB | OK | 270.977 | 94.1758 | -1.52474 | -1.60958 | 0.03475 | 0.162348 |
| PSPPH_2437 | hypothetical protein | chromosome:2820103-2820448 | 1448A-rhpS-KB | 1448A-WT-KB | OK | 405.655 | 140.911 | -1.52547 | -1.54029 | 0.05245 | 0.208483 |
| PSPPH_2971 | methyl-accepting chemotaxis transducer/sensory box protein | chromosome:3448737-3450894 | 1448A-rhpS-KB | 1448A-WT-KB | OK | 257.15 | 89.1688 | -1.528 | -3.63243 | 0.00005 | 0.00121565 |
| PSPPH_3228 | group 1 glycosyl transferase | chromosome:3741843-3746385 | 1448A-rhpS-KB | 1448A-WT-KB | OK | 83.7468 | 29.0022 | -1.52987 | -1.5021 | 0.05355 | 0.210976 |
| PSPPH_1466 | hypothetical protein | chromosome:1708904-1709471 | 1448A-rhpS-KB | 1448A-WT-KB | OK | 221.215 | 76.2619 | -1.53641 | -1.76219 | 0.02665 | 0.135102 |
| PSPPH_1085 | PsiE family protein | chromosome:1278185-1278674 | 1448A-rhpS-KB | 1448A-WT-KB | OK | 194.994 | 67.2227 | -1.53641 | -1.4788 | 0.054 | 0.211582 |
| PSPPH_0816 | zinc-binding protein | chromosome:977779-977977 | 1448A-rhpS-KB | 1448A-WT-KB | OK | 315.579 | 108.743 | -1.53708 | -0.784076 | 0.37355 | 0.606315 |
| PSPPH_1126 | lipoprotein | chromosome:1325704-1326738 | 1448A-rhpS-KB | 1448A-WT-KB | OK | 64.9402 | 22.2841 | -1.5431 | -0.724602 | 0.52855 | 0.728514 |
| shcV | type III chaperone protein ShcV | chromosome:2727200-2727587 | 1448A-rhpS-KB | 1448A-WT-KB | OK | 89.5105 | 30.7136 | -1.54318 | -0.809639 | 0.2861 | 0.522494 |
| PSPPH_2697 | peptide ABC transporter ATP-binding protein | chromosome:3124712-3126699 | 1448A-rhpS-KB | 1448A-WT-KB | OK | 60.359 | 20.7077 | -1.5434 | -1.27388 | 0.1126 | 0.315914 |
| PSPPH_3042 | 3-hydroxyacyl-CoA-acyl carrier protein transferase | chromosome:3533705-3534569 | 1448A-rhpS-KB | 1448A-WT-KB | OK | 4643.62 | 1592.15 | -1.54427 | -5.77954 | 0.00005 | 0.00121565 |
| PSPPH_2990 | phytase domain-containing protein | chromosome:3470465-3472394 | 1448A-rhpS-KB | 1448A-WT-KB | NOTEST | 20.998 | 7.1878 | -1.54663 | 0 | 1 | 1 |
| PSPPH_1480 | sensor histidine kinase | chromosome:1719146-1720331 | 1448A-rhpS-KB | 1448A-WT-KB | OK | 335.402 | 114.445 | -1.55124 | -3.10678 | 0.00025 | 0.0043799 |
| PSPPH_3137 | hypothetical protein | chromosome:3638283-3638727 | 1448A-rhpS-KB | 1448A-WT-KB | OK | 563.871 | 191.986 | -1.55436 | -2.25582 | 0.0054 | 0.0447787 |
| PSPPH_0782 | RulB | chromosome:924516-925533 | 1448A-rhpS-KB | 1448A-WT-KB | OK | 287.668 | 97.6141 | -1.55924 | -2.77024 | 0.0007 | 0.00962231 |
| PSPPH_3317 | hypothetical protein | chromosome:3838905-3839802 | 1448A-rhpS-KB | 1448A-WT-KB | OK | 169.684 | 57.5529 | -1.55989 | -2.04233 | 0.01095 | 0.0746857 |
| PSPPH_3428 | response regulator/TPR domain-containing protein | chromosome:3961127-3963421 | 1448A-rhpS-KB | 1448A-WT-KB | OK | 89.6055 | 30.3566 | -1.56158 | -2.0295 | 0.01275 | 0.0831542 |
| PSPPH_2671 | spemidine/putrescine ABC transporter substrate-binding protein | chromosome:3092363-3093380 | 1448A-rhpS-KB | 1448A-WT-KB | OK | 348.453 | 117.606 | -1.567 | -3.01581 | 0.00045 | 0.00699261 |
| pcaT | dicarboxylic acid transport protein | chromosome:4590429-4591731 | 1448A-rhpS-KB | 1448A-WT-KB | OK | 96.1672 | 32.3967 | -1.5697 | -1.90881 | 0.01825 | 0.104696 |
| PSPPH_0227 | cytochrome c5 | chromosome:264268-264592 | 1448A-rhpS-KB | 1448A-WT-KB | OK | 284.971 | 95.9213 | -1.57089 | -1.28631 | 0.11745 | 0.324144 |
| nrdR | transcriptional regulator NrdR | chromosome:5141467-5141932 | 1448A-rhpS-KB | 1448A-WT-KB | OK | 440.053 | 147.922 | -1.57284 | -2.0584 | 0.0122 | 0.080448 |
| PSPPH_4671 | hypothetical protein | chromosome:5308533-5309343 | 1448A-rhpS-KB | 1448A-WT-KB | OK | 126.979 | 42.6101 | -1.57533 | -1.72196 | 0.0319 | 0.151408 |
| pstC | phosphate ABC transporter permease | chromosome:3500875-3503531 | 1448A-rhpS-KB | 1448A-WT-KB | OK | 83.5458 | 27.99 | -1.57766 | -1.41483 | 0.0777 | 0.259731 |
| PSPPH_2731 | hypothetical protein | chromosome:3162688-3163060 | 1448A-rhpS-KB | 1448A-WT-KB | OK | 332.958 | 111.443 | -1.57903 | -1.53283 | 0.05595 | 0.216058 |
| PSPPH_2446 | allophanate hydrolase subunit 1 | chromosome:2827156-2829000 | 1448A-rhpS-KB | 1448A-WT-KB | OK | 95.862 | 31.9777 | -1.58389 | -1.34097 | 0.0803 | 0.262573 |
| csrA1 | carbon storage regulator | chromosome:3779077-3779266 | 1448A-rhpS-KB | 1448A-WT-KB | OK | 996.034 | 332.055 | -1.58477 | -1.15738 | 0.16155 | 0.387763 |
| PSPPH_4480 | hypothetical protein | chromosome:5116578-5116833 | 1448A-rhpS-KB | 1448A-WT-KB | OK | 318.438 | 106.125 | -1.58525 | -0.98903 | 0.13635 | 0.35122 |
| PSPPH_2793 | prophage PSPPH05, site-specific recombinase phage integrase | chromosome:3232786-3233995 | 1448A-rhpS-KB | 1448A-WT-KB | OK | 176.474 | 58.7296 | -1.5873 | -2.48617 | 0.00245 | 0.0257538 |
| phoB | phosphate regulon transcriptional regulatory protein PhoB | chromosome:5799448-5800138 | 1448A-rhpS-KB | 1448A-WT-KB | OK | 169.339 | 56.191 | -1.5915 | -1.87972 | 0.0236 | 0.124588 |
| PSPPH_1913 | balhimycin biosynthetic protein MbtH | chromosome:2231203-2231464 | 1448A-rhpS-KB | 1448A-WT-KB | OK | 234.994 | 77.9437 | -1.59212 | -1.02703 | 0.22165 | 0.458953 |
| PSPPH_2635 | acetyltransferase | chromosome:3040906-3041578 | 1448A-rhpS-KB | 1448A-WT-KB | OK | 85.8609 | 28.3644 | -1.59792 | -1.24142 | 0.0946 | 0.284747 |
| PSPPH_1695 | lipoprotein | chromosome:1958686-1959331 | 1448A-rhpS-KB | 1448A-WT-KB | OK | 360.519 | 119.061 | -1.59837 | -2.42139 | 0.0036 | 0.0336817 |
| PSPPH_0548 | lipoprotein | chromosome:642500-643199 | 1448A-rhpS-KB | 1448A-WT-KB | OK | 2379.14 | 785.343 | -1.59905 | -4.79557 | 0.00005 | 0.00121565 |
| PSPPH_2012 | chaperone protein PapD | chromosome:2364799-2365498 | 1448A-rhpS-KB | 1448A-WT-KB | OK | 49.7683 | 16.2924 | -1.61103 | -1.01549 | 0.1328 | 0.346696 |
| PSPPH_4743 | methyl-accepting chemotaxis protein | chromosome:5384501-5386127 | 1448A-rhpS-KB | 1448A-WT-KB | OK | 91.0306 | 29.7913 | -1.61146 | -2.15376 | 0.00705 | 0.0537243 |
| PSPPH_3095 | arginyl-tRNA-protein transferase | chromosome:3592347-3593055 | 1448A-rhpS-KB | 1448A-WT-KB | OK | 175.872 | 57.5082 | -1.61268 | -1.92056 | 0.01965 | 0.108882 |
| fliE | flagellar hook-basal body protein FliE | chromosome:3912008-3912338 | 1448A-rhpS-KB | 1448A-WT-KB | OK | 101.415 | 33.1553 | -1.61297 | -0.830669 | 0.23995 | 0.47354 |
| PSPPH_0363 | methyl-accepting chemotaxis protein | chromosome:418127-420044 | 1448A-rhpS-KB | 1448A-WT-KB | OK | 91.1123 | 29.7763 | -1.61348 | -2.3664 | 0.00385 | 0.0353725 |
| PSPPH_2160 | hypothetical protein | chromosome:2513941-2517414 | 1448A-rhpS-KB | 1448A-WT-KB | OK | 171.007 | 55.8566 | -1.61425 | -1.5283 | 0.04015 | 0.177156 |
| osmC | hydroperoxide resistance protein OsmC | chromosome:46050-46482 | 1448A-rhpS-KB | 1448A-WT-KB | OK | 503.619 | 162.698 | -1.63014 | -2.15093 | 0.00845 | 0.0622687 |
| PSPPH_4071 | hypothetical protein | chromosome:4654466-4655413 | 1448A-rhpS-KB | 1448A-WT-KB | OK | 926.03 | 298.998 | -1.63092 | -1.07005 | 0.21925 | 0.456215 |
| PSPPH_2696 | peptide ABC transporter permease | chromosome:3121352-3124701 | 1448A-rhpS-KB | 1448A-WT-KB | NOTEST | 26.7692 | 8.62942 | -1.63324 | 0 | 1 | 1 |
| PSPPH_4409 | iojap domain-containing protein | chromosome:5032852-5033239 | 1448A-rhpS-KB | 1448A-WT-KB | OK | 790.507 | 254.251 | -1.63652 | -2.42755 | 0.0038 | 0.0350031 |
| PSPPH_0083 | metallo-beta-lactamase | chromosome:90954-91839 | 1448A-rhpS-KB | 1448A-WT-KB | OK | 1617.29 | 519.426 | -1.63859 | -4.87973 | 0.00005 | 0.00121565 |
| PSPPH_5034 | hypothetical protein | chromosome:5702959-5708802 | 1448A-rhpS-KB | 1448A-WT-KB | OK | 133.395 | 42.812 | -1.63962 | -0.641517 | 0.41105 | 0.639447 |
| PSPPH_1120 | polysaccharide deacetylase | chromosome:1320349-1321549 | 1448A-rhpS-KB | 1448A-WT-KB | OK | 337.679 | 108.094 | -1.64336 | -3.23218 | 0.0002 | 0.00374241 |
| PSPPH_4073 | hypothetical protein | chromosome:4655417-4656149 | 1448A-rhpS-KB | 1448A-WT-KB | OK | 153.099 | 48.9818 | -1.64414 | -1.78094 | 0.03305 | 0.155422 |
| PSPPH_1079 | hypothetical protein | chromosome:1268152-1269025 | 1448A-rhpS-KB | 1448A-WT-KB | OK | 92.7064 | 29.6569 | -1.6443 | -1.59098 | 0.04275 | 0.18475 |
| rimO | 30S ribosomal protein S12 methylthiotransferase | chromosome:4345005-4346349 | 1448A-rhpS-KB | 1448A-WT-KB | OK | 266.362 | 84.761 | -1.65191 | -3.15576 | 0.0003 | 0.0050338 |
| PSPPH_2124 | lipoprotein | chromosome:2481605-2481875 | 1448A-rhpS-KB | 1448A-WT-KB | OK | 819.411 | 259.938 | -1.65642 | -1.78248 | 0.02995 | 0.145239 |
| PSPPH_2845 | hypothetical protein | chromosome:3290577-3290895 | 1448A-rhpS-KB | 1448A-WT-KB | OK | 100.214 | 31.787 | -1.65658 | -1.15204 | 0.29995 | 0.534407 |
| PSPPH_0768 | thioredoxin | chromosome:899428-900226 | 1448A-rhpS-KB | 1448A-WT-KB | NOTEST | 36.8935 | 11.6872 | -1.65843 | 0 | 1 | 1 |
| PSPPH_0361 | hypothetical protein | chromosome:415067-417925 | 1448A-rhpS-KB | 1448A-WT-KB | OK | 61.7171 | 19.5387 | -1.65933 | -1.30201 | 0.12825 | 0.338923 |
| PSPPH_2589 | zinc-binding oxidoreductase | chromosome:2980474-2981551 | 1448A-rhpS-KB | 1448A-WT-KB | OK | 298.718 | 94.5449 | -1.65971 | -3.00437 | 0.0003 | 0.0050338 |
| PSPPH_2769 | TrapT dctQ-M fusion permease, dicarboxylate transport | chromosome:3206290-3208068 | 1448A-rhpS-KB | 1448A-WT-KB | OK | 51.8438 | 16.3622 | -1.6638 | -0.768744 | 0.36505 | 0.599002 |
| PSPPH_2082 | hypothetical protein | chromosome:2445104-2445449 | 1448A-rhpS-KB | 1448A-WT-KB | OK | 685.463 | 216.119 | -1.66525 | -2.06328 | 0.0119 | 0.0789065 |
| PSPPH_3292 | HupC/HyaC/HydC family protein | chromosome:3816448-3817069 | 1448A-rhpS-KB | 1448A-WT-KB | OK | 187.013 | 58.9629 | -1.66526 | -1.76043 | 0.0304 | 0.146231 |
| PSPPH_4381 | sensor histidine kinase | chromosome:5003793-5005788 | 1448A-rhpS-KB | 1448A-WT-KB | OK | 103.353 | 32.4215 | -1.67256 | -2.56912 | 0.00215 | 0.0234271 |
| PSPPH_3876 | chemotaxis-specific methylesterase | chromosome:4430452-4436346 | 1448A-rhpS-KB | 1448A-WT-KB | OK | 330.745 | 103.516 | -1.67587 | -2.19553 | 0.0063 | 0.0499251 |
| PSPPH_3885 | hypothetical protein | chromosome:4443665-4444130 | 1448A-rhpS-KB | 1448A-WT-KB | NOTEST | 49.1653 | 15.381 | -1.67649 | 0 | 1 | 1 |
| PSPPH_3291 | molybdopterin-binding oxidoreductase | chromosome:3815681-3816329 | 1448A-rhpS-KB | 1448A-WT-KB | OK | 114.034 | 35.6449 | -1.6777 | -1.42174 | 0.06045 | 0.225285 |
| PSPPH_4418 | type IV leader peptidase | chromosome:5043759-5044233 | 1448A-rhpS-KB | 1448A-WT-KB | NOTEST | 44.6491 | 13.8674 | -1.68693 | 0 | 1 | 1 |
| PSPPH_5132 | LysR family transcriptional regulator | chromosome:5819960-5820872 | 1448A-rhpS-KB | 1448A-WT-KB | OK | 212.598 | 66.0242 | -1.68706 | -2.35566 | 0.00515 | 0.0434106 |
| PSPPH_1590 | polyamine ABC transporter ATP-binding protein | chromosome:1844601-1845591 | 1448A-rhpS-KB | 1448A-WT-KB | OK | 58.1346 | 18.0282 | -1.68915 | -1.40729 | 0.0863 | 0.27247 |
| PSPPH_4513 | hypothetical protein | chromosome:5144823-5145246 | 1448A-rhpS-KB | 1448A-WT-KB | OK | 64.9087 | 20.0705 | -1.69334 | -0.852011 | 0.2456 | 0.479134 |
| dxnH | 3-oxoadipate CoA-succinyl transferase subunit beta | chromosome:3660311-3660968 | 1448A-rhpS-KB | 1448A-WT-KB | OK | 189.162 | 58.4847 | -1.69349 | -1.90791 | 0.0193 | 0.108116 |
| fabG | 3-ketoacyl-ACP reductase | chromosome:2143358-2144108 | 1448A-rhpS-KB | 1448A-WT-KB | OK | 82.178 | 25.4028 | -1.69377 | -1.35835 | 0.08285 | 0.266282 |
| PSPPH_3168 | Fis family transcriptional regulator | chromosome:3674007-3676253 | 1448A-rhpS-KB | 1448A-WT-KB | OK | 47.5169 | 14.6872 | -1.69388 | -1.20298 | 0.0987 | 0.291641 |
| greB | transcription elongation factor GreB | chromosome:2388808-2389282 | 1448A-rhpS-KB | 1448A-WT-KB | OK | 387.503 | 119.681 | -1.69501 | -2.05457 | 0.0143 | 0.0887295 |
| tpiA | triosephosphate isomerase | chromosome:4781443-4782199 | 1448A-rhpS-KB | 1448A-WT-KB | OK | 2511.98 | 774.792 | -1.69694 | -5.20303 | 0.00005 | 0.00121565 |
| PSPPH_2817 | phosphoesterase | chromosome:3263127-3263586 | 1448A-rhpS-KB | 1448A-WT-KB | OK | 97.1698 | 29.8909 | -1.7008 | -1.08145 | 0.17825 | 0.407852 |
| PSPPH_4493 | bacterioferritin | chromosome:5128839-5129379 | 1448A-rhpS-KB | 1448A-WT-KB | OK | 451.507 | 138.56 | -1.70424 | -2.56863 | 0.00285 | 0.0291026 |
| PSPPH_4851 | hypothetical protein | chromosome:5511365-5512531 | 1448A-rhpS-KB | 1448A-WT-KB | NOTEST | 40.3457 | 12.3665 | -1.70598 | 0 | 1 | 1 |
| PSPPH_3222 | capsular polysaccharide biosynthesis protein | chromosome:3733534-3736428 | 1448A-rhpS-KB | 1448A-WT-KB | OK | 56.5343 | 17.3246 | -1.70631 | -1.45255 | 0.0985 | 0.291423 |
| PSPPH_0078 | multidrug efflux protein NorA | chromosome:83222-84602 | 1448A-rhpS-KB | 1448A-WT-KB | OK | 174.016 | 52.9896 | -1.71544 | -2.6636 | 0.00215 | 0.0234271 |
| PSPPH_1792 | exonuclease | chromosome:2097921-2098464 | 1448A-rhpS-KB | 1448A-WT-KB | OK | 268.657 | 81.7492 | -1.71649 | -1.96249 | 0.0166 | 0.0983588 |
| PSPPH_1593 | hypothetical protein | chromosome:1845592-1848022 | 1448A-rhpS-KB | 1448A-WT-KB | OK | 75.2113 | 22.8791 | -1.71692 | -1.36994 | 0.09815 | 0.290869 |
| PSPPH_3503 | hypothetical protein | chromosome:4042645-4042864 | 1448A-rhpS-KB | 1448A-WT-KB | OK | 214.465 | 65.2205 | -1.71735 | -1.08567 | 0.3283 | 0.565253 |
| folK1 | 2-amino-4-hydroxy-6- hydroxymethyldihydropteridine pyrophosphokinase | chromosome:739271-740140 | 1448A-rhpS-KB | 1448A-WT-KB | OK | 184.56 | 56.1071 | -1.71784 | -1.51628 | 0.0602 | 0.224587 |
| PSPPH_0629 | protein kinase | chromosome:744515-746438 | 1448A-rhpS-KB | 1448A-WT-KB | OK | 1547.19 | 469.254 | -1.72121 | -6.21506 | 0.00005 | 0.00121565 |
| PSPPH_2578 | hypothetical protein | chromosome:2971736-2972246 | 1448A-rhpS-KB | 1448A-WT-KB | OK | 982.045 | 297.41 | -1.72334 | -3.37477 | 0.00005 | 0.00121565 |
| PSPPH_3607 | SelT/selW/selH domain-containing protein | chromosome:4153518-4153821 | 1448A-rhpS-KB | 1448A-WT-KB | OK | 99.2852 | 30.0576 | -1.72385 | -0.657209 | 0.31025 | 0.546762 |
| PSPPH_3559 | hypothetical protein | chromosome:4096537-4097107 | 1448A-rhpS-KB | 1448A-WT-KB | NOTEST | 44.0736 | 13.3029 | -1.72818 | 0 | 1 | 1 |
| tonB4 | ferric siderophore ABC transporter substrate-binding protein | chromosome:5726419-5727232 | 1448A-rhpS-KB | 1448A-WT-KB | NOTEST | 31.7596 | 9.52412 | -1.73753 | 0 | 1 | 1 |
| PSPPH_1092 | sigma factor regulatory protein FecR/PupR family | chromosome:1287130-1288608 | 1448A-rhpS-KB | 1448A-WT-KB | OK | 65.2711 | 19.5095 | -1.74227 | -1.39008 | 0.0637 | 0.231259 |
| PSPPH_3128 | peptidase propeptide/YPEB domain-containing protein | chromosome:3628139-3630740 | 1448A-rhpS-KB | 1448A-WT-KB | OK | 105.88 | 31.5963 | -1.7446 | -0.66529 | 0.50245 | 0.709224 |
| PSPPH_2984 | AraC family transcriptional regulator | chromosome:3465327-3465717 | 1448A-rhpS-KB | 1448A-WT-KB | OK | 176.469 | 52.5049 | -1.74889 | -1.27987 | 0.1132 | 0.316323 |
| PSPPH_4191 | hypothetical protein | chromosome:4780165-4780642 | 1448A-rhpS-KB | 1448A-WT-KB | OK | 1422.25 | 422.486 | -1.7512 | -3.64453 | 0.00005 | 0.00121565 |
| atpI | F0F1 ATP synthase subunit I | chromosome:5917628-5918036 | 1448A-rhpS-KB | 1448A-WT-KB | OK | 174.272 | 51.6925 | -1.75332 | -1.27154 | 0.1158 | 0.32058 |
| PSPPH_1057 | pressure-regulated protein | chromosome:1250408-1250714 | 1448A-rhpS-KB | 1448A-WT-KB | OK | 139.754 | 41.4414 | -1.75375 | -1.13346 | 0.2734 | 0.509906 |
| PSPPH_3219 | hypothetical protein | chromosome:3730859-3731291 | 1448A-rhpS-KB | 1448A-WT-KB | OK | 328.96 | 97.2493 | -1.75815 | -1.84678 | 0.02455 | 0.127532 |
| PSPPH_4814 | hypothetical protein | chromosome:5466579-5466990 | 1448A-rhpS-KB | 1448A-WT-KB | OK | 559.954 | 165.516 | -1.75834 | -2.27382 | 0.0056 | 0.0457995 |
| PSPPH_3687 | hypothetical protein | chromosome:4235556-4236063 | 1448A-rhpS-KB | 1448A-WT-KB | NOTEST | 58.5846 | 17.3014 | -1.75963 | 0 | 1 | 1 |
| PSPPH_0747 | hypothetical protein | chromosome:871621-873132 | 1448A-rhpS-KB | 1448A-WT-KB | OK | 134.222 | 39.4397 | -1.7669 | -2.37426 | 0.00665 | 0.0515555 |
| PSPPH_2054 | hypothetical protein | chromosome:2407277-2407829 | 1448A-rhpS-KB | 1448A-WT-KB | OK | 114.081 | 33.4893 | -1.76829 | -1.36642 | 0.0793 | 0.261215 |
| PSPPH_3378 | STAS domain-containing protein | chromosome:3904456-3906470 | 1448A-rhpS-KB | 1448A-WT-KB | OK | 334.744 | 97.5671 | -1.77859 | -1.26467 | 0.1099 | 0.311732 |
| PSPPH_0554 | flagellar motor protein MotA | chromosome:652259-653111 | 1448A-rhpS-KB | 1448A-WT-KB | OK | 165.111 | 47.7654 | -1.7894 | -2.11193 | 0.0111 | 0.0752778 |
| PSPPH_0345 | RNA polymerase sigma-70 family protein | chromosome:392820-394092 | 1448A-rhpS-KB | 1448A-WT-KB | OK | 265.286 | 76.6479 | -1.79123 | -3.18669 | 0.0002 | 0.00374241 |
| PSPPH_2829 | hypothetical protein | chromosome:3273989-3274952 | 1448A-rhpS-KB | 1448A-WT-KB | OK | 98.5278 | 28.4288 | -1.79318 | -1.77957 | 0.0327 | 0.153979 |
| hrcR | type III secretion system protein | chromosome:1497106-1497754 | 1448A-rhpS-KB | 1448A-WT-KB | OK | 48.4299 | 13.9619 | -1.79441 | -0.988695 | 0.20055 | 0.43414 |
| glpE | thiosulfate sulfurtransferase | chromosome:746722-748370 | 1448A-rhpS-KB | 1448A-WT-KB | OK | 515.897 | 148.708 | -1.7946 | -1.42396 | 0.06195 | 0.228677 |
| rpmH | 50S ribosomal protein L34 | chromosome:5926794-5926929 | 1448A-rhpS-KB | 1448A-WT-KB | OK | 1781.1 | 512.767 | -1.79639 | -0.74582 | 0.2992 | 0.533515 |
| PSPPH_2463 | glutamine ABC transporter permease | chromosome:2844436-2845177 | 1448A-rhpS-KB | 1448A-WT-KB | NOTEST | 5.44099 | 1.56224 | -1.80025 | 0 | 1 | 1 |
| PSPPH_2826 | FKBP-type peptidylprolyl isomerase | chromosome:3272390-3272810 | 1448A-rhpS-KB | 1448A-WT-KB | OK | 610.502 | 175.165 | -1.80128 | -2.45695 | 0.00565 | 0.0458934 |
| PSPPH_2454 | nitrogen assimilation transcriptional regulator | chromosome:2837034-2837946 | 1448A-rhpS-KB | 1448A-WT-KB | NOTEST | 15.704 | 4.48179 | -1.80899 | 0 | 1 | 1 |
| fliL | flagellar basal body protein FliL | chromosome:3901527-3902028 | 1448A-rhpS-KB | 1448A-WT-KB | NOTEST | 16.8245 | 4.79733 | -1.81026 | 0 | 1 | 1 |
| dadA | D-amino acid dehydrogenase small subunit | chromosome:260782-262409 | 1448A-rhpS-KB | 1448A-WT-KB | OK | 3026.2 | 862.021 | -1.81171 | -6.26975 | 0.00005 | 0.00121565 |
| PSPPH_2190 | hypothetical protein | chromosome:2546184-2547971 | 1448A-rhpS-KB | 1448A-WT-KB | NOTEST | 30.1678 | 8.56606 | -1.81631 | 0 | 1 | 1 |
| PSPPH_0093 | sensory box protein | chromosome:101937-102888 | 1448A-rhpS-KB | 1448A-WT-KB | OK | 356.097 | 100.811 | -1.82061 | -3.19787 | 0.00025 | 0.0043799 |
| PSPPH_0249 | hypothetical protein | chromosome:289171-289336 | 1448A-rhpS-KB | 1448A-WT-KB | OK | 451.131 | 127.566 | -1.82231 | -0.739465 | 0.338 | 0.57415 |
| PSPPH_4776 | hypothetical protein | chromosome:5421032-5421413 | 1448A-rhpS-KB | 1448A-WT-KB | OK | 257.643 | 72.3834 | -1.83164 | -1.52904 | 0.06245 | 0.229154 |
| PSPPH_1806 | Smr domain-containing protein | chromosome:2110050-2110608 | 1448A-rhpS-KB | 1448A-WT-KB | OK | 604.048 | 169.394 | -1.83428 | -2.97433 | 0.001 | 0.0127643 |
| PSPPH_2067 | RNA polymerase sigma factor SigX | chromosome:2422642-2423140 | 1448A-rhpS-KB | 1448A-WT-KB | OK | 2291.77 | 642.518 | -1.83466 | -4.57347 | 0.00005 | 0.00121565 |
| PSPPH_1197 | HlyD family secretion protein | chromosome:1404177-1408445 | 1448A-rhpS-KB | 1448A-WT-KB | OK | 64.4163 | 17.9274 | -1.84526 | -1.62355 | 0.0457 | 0.193047 |
| PSPPH_4417 | hypothetical protein | chromosome:5038443-5043687 | 1448A-rhpS-KB | 1448A-WT-KB | OK | 223.866 | 62.2226 | -1.84713 | -1.14298 | 0.15435 | 0.375781 |
| PSPPH_4211 | hypothetical protein | chromosome:4800903-4803056 | 1448A-rhpS-KB | 1448A-WT-KB | OK | 112.273 | 31.1981 | -1.84749 | -0.684794 | 0.39755 | 0.625647 |
| PSPPH_4386 | hypothetical protein | chromosome:5010032-5010425 | 1448A-rhpS-KB | 1448A-WT-KB | OK | 164.786 | 45.7211 | -1.84966 | -1.25386 | 0.10735 | 0.307181 |
| wspA | methyl-accepting chemotaxis protein WspA | chromosome:4436354-4437977 | 1448A-rhpS-KB | 1448A-WT-KB | OK | 553.422 | 152.953 | -1.85529 | -4.75237 | 0.00005 | 0.00121565 |
| hrcQa | type III secretion component protein HrcQa | chromosome:1497756-1499418 | 1448A-rhpS-KB | 1448A-WT-KB | NOTEST | 49.759 | 13.7499 | -1.85553 | 0 | 1 | 1 |
| PSPPH_1533 | SirA domain-containing protein | chromosome:1788512-1788764 | 1448A-rhpS-KB | 1448A-WT-KB | OK | 540.072 | 149.231 | -1.85561 | -1.37904 | 0.0962 | 0.286994 |
| hrpF | type III secretion component protein HrpF | chromosome:1491470-1492113 | 1448A-rhpS-KB | 1448A-WT-KB | NOTEST | 43.2639 | 11.8892 | -1.86351 | 0 | 1 | 1 |
| PSPPH_2693 | LysR family transcriptional regulator | chromosome:3120155-3121112 | 1448A-rhpS-KB | 1448A-WT-KB | OK | 160.05 | 43.9689 | -1.86397 | -2.24539 | 0.009 | 0.0645904 |
| PSPPH_1026 | hypothetical protein | chromosome:1216797-1217031 | 1448A-rhpS-KB | 1448A-WT-KB | NOTEST | 39.7526 | 10.8986 | -1.8669 | 0 | 1 | 1 |
| PSPPH_0906 | sensory box protein/methyl-accepting chemotaxis protein | chromosome:1079449-1080763 | 1448A-rhpS-KB | 1448A-WT-KB | OK | 294.224 | 80.4228 | -1.87124 | -3.48445 | 0.00005 | 0.00121565 |
| PSPPH_2445 | hypothetical protein | chromosome:2826314-2827070 | 1448A-rhpS-KB | 1448A-WT-KB | OK | 165.846 | 45.0746 | -1.87945 | -2.03774 | 0.0201 | 0.110519 |
| PSPPH_1653 | hypothetical protein | chromosome:1913815-1914376 | 1448A-rhpS-KB | 1448A-WT-KB | OK | 264.525 | 71.8579 | -1.88019 | -2.05942 | 0.01315 | 0.0846813 |
| PSPPH_3684 | hypothetical protein | chromosome:4232118-4232718 | 1448A-rhpS-KB | 1448A-WT-KB | OK | 225.182 | 61.0177 | -1.88379 | -1.98657 | 0.01725 | 0.101068 |
| PSPPH_1436 | hypothetical protein | chromosome:1675205-1675490 | 1448A-rhpS-KB | 1448A-WT-KB | OK | 498.048 | 134.59 | -1.88771 | -1.5633 | 0.05815 | 0.221428 |
| PSPPH_2669 | hypothetical protein | chromosome:3091708-3091975 | 1448A-rhpS-KB | 1448A-WT-KB | OK | 840.983 | 226.674 | -1.89146 | -1.90275 | 0.02505 | 0.12919 |
| PSPPH_0898 | hypothetical protein | chromosome:1069588-1069957 | 1448A-rhpS-KB | 1448A-WT-KB | OK | 533.935 | 143.006 | -1.90059 | -2.06254 | 0.01645 | 0.0983588 |
| PSPPH_1842 | hypothetical protein | chromosome:2138613-2138961 | 1448A-rhpS-KB | 1448A-WT-KB | OK | 558.266 | 149.44 | -1.90138 | -2.05093 | 0.01665 | 0.0983588 |
| PSPPH_0761 | hypothetical protein | chromosome:891098-891575 | 1448A-rhpS-KB | 1448A-WT-KB | OK | 53.6679 | 14.3377 | -1.90425 | -0.83911 | 0.24135 | 0.474992 |
| PSPPH_2256 | hypothetical protein | chromosome:2619714-2619897 | 1448A-rhpS-KB | 1448A-WT-KB | OK | 272.419 | 72.552 | -1.90874 | -2.0591 | 0.39165 | 0.61991 |
| PSPPH_4619 | beta alanine--pyruvate transaminase | chromosome:5254424-5255771 | 1448A-rhpS-KB | 1448A-WT-KB | OK | 264.525 | 70.3173 | -1.91145 | -3.46989 | 0.0001 | 0.00221988 |
| PSPPH_0738 | hypothetical protein | chromosome:863033-864855 | 1448A-rhpS-KB | 1448A-WT-KB | OK | 510.886 | 135.31 | -1.91673 | -1.22651 | 0.1586 | 0.382223 |
| PSPPH_0607 | hypothetical protein | chromosome:717657-717912 | 1448A-rhpS-KB | 1448A-WT-KB | OK | 1110.15 | 293.435 | -1.91964 | -2.10275 | 0.0166 | 0.0983588 |
| maoC2 | acyl dehydratase | chromosome:5296248-5296704 | 1448A-rhpS-KB | 1448A-WT-KB | OK | 426.307 | 112.329 | -1.92416 | -2.31046 | 0.00835 | 0.0617865 |
| fleQ | flagellar regulator FleQ | chromosome:3915333-3916809 | 1448A-rhpS-KB | 1448A-WT-KB | OK | 1550.18 | 408.459 | -1.92418 | -6.25658 | 0.00005 | 0.00121565 |
| ipk | 4-diphosphocytidyl-2-C-methyl-D-erythritol kinase | chromosome:1186252-1187119 | 1448A-rhpS-KB | 1448A-WT-KB | OK | 444.116 | 116.973 | -1.92476 | -3.43069 | 0.00005 | 0.00121565 |
| PSPPH_5050 | biopolymer transport protein ExbD | chromosome:5725257-5726366 | 1448A-rhpS-KB | 1448A-WT-KB | NOTEST | 27.2395 | 7.10029 | -1.93975 | 0 | 1 | 1 |
| PSPPH_1916 | cation ABC transporter ATP-binding protein | chromosome:2232649-2235248 | 1448A-rhpS-KB | 1448A-WT-KB | NOTEST | 34.3157 | 8.93888 | -1.9407 | 0 | 1 | 1 |
| avrF | type III chaperone protein AvrF | chromosome:1477288-1477678 | 1448A-rhpS-KB | 1448A-WT-KB | OK | 114.274 | 29.7293 | -1.94254 | -1.18409 | 0.23205 | 0.468144 |
| PSPPH_0808 | methyl-accepting chemotaxis protein | chromosome:952121-953336 | 1448A-rhpS-KB | 1448A-WT-KB | OK | 85.7733 | 22.308 | -1.94297 | -1.97947 | 0.0223 | 0.119312 |
| fliO | flagellar protein FliO | chromosome:3898836-3900029 | 1448A-rhpS-KB | 1448A-WT-KB | OK | 75.0486 | 19.4526 | -1.94787 | -0.977827 | 0.3265 | 0.563725 |
| treY | malto-oligosyltrehalose synthase | chromosome:2603765-2610437 | 1448A-rhpS-KB | 1448A-WT-KB | OK | 98.3122 | 25.4341 | -1.95061 | -2.65292 | 0.0032 | 0.0312481 |
| PSPPH_1232 | hypothetical protein | chromosome:1440009-1440527 | 1448A-rhpS-KB | 1448A-WT-KB | OK | 396.577 | 101.711 | -1.96313 | -1.93398 | 0.02555 | 0.131201 |
| PSPPH_2911 | luciferase | chromosome:3381822-3382836 | 1448A-rhpS-KB | 1448A-WT-KB | OK | 262.05 | 67.0386 | -1.96678 | -3.08623 | 0.0005 | 0.00757203 |
| PSPPH_3179 | hypothetical protein | chromosome:3686568-3686865 | 1448A-rhpS-KB | 1448A-WT-KB | OK | 232.598 | 59.3652 | -1.97015 | -1.09149 | 0.1575 | 0.380084 |
| PSPPH_2229 | Fis family transcriptional regulator | chromosome:2589656-2592063 | 1448A-rhpS-KB | 1448A-WT-KB | OK | 121.219 | 30.8371 | -1.97487 | -2.35711 | 0.00935 | 0.0661721 |
| PSPPH_1609 | Rieske (2Fe-2S) domain-containing protein | chromosome:1870154-1873726 | 1448A-rhpS-KB | 1448A-WT-KB | NOTEST | 40.8713 | 10.3166 | -1.98612 | 0 | 1 | 1 |
| PSPPH_0627 | SpoVR family protein | chromosome:741563-744394 | 1448A-rhpS-KB | 1448A-WT-KB | OK | 617.802 | 155.843 | -1.98705 | -4.25378 | 0.00005 | 0.00121565 |
| PSPPH_2547 | oxidoreductase | chromosome:2937437-2939092 | 1448A-rhpS-KB | 1448A-WT-KB | OK | 68.1955 | 17.139 | -1.99239 | -1.35425 | 0.1052 | 0.303458 |
| PSPPH_3922 | hypothetical protein | chromosome:4479660-4482798 | 1448A-rhpS-KB | 1448A-WT-KB | OK | 61.0641 | 15.2196 | -2.00439 | -2.80914 | 0.00225 | 0.0242214 |
| mscL | large-conductance mechanosensitive channel | chromosome:4954004-4954451 | 1448A-rhpS-KB | 1448A-WT-KB | OK | 947.805 | 235.87 | -2.0066 | -3.24417 | 0.0004 | 0.00632566 |
| algA | alginate biosynthesis protein AlgA | chromosome:1302145-1303597 | 1448A-rhpS-KB | 1448A-WT-KB | OK | 1059.16 | 263.118 | -2.00914 | -5.94441 | 0.00005 | 0.00121565 |
| PSPPH_4223 | hypothetical protein | chromosome:4815939-4816989 | 1448A-rhpS-KB | 1448A-WT-KB | OK | 336.333 | 82.6097 | -2.02551 | -3.39484 | 0.0002 | 0.00374241 |
| PSPPH_3557 | hypothetical protein | chromosome:4092785-4093256 | 1448A-rhpS-KB | 1448A-WT-KB | OK | 110.583 | 26.9564 | -2.03644 | -1.24885 | 0.12905 | 0.340149 |
| PSPPH_2438 | translation initiation inhibitor | chromosome:2820462-2820867 | 1448A-rhpS-KB | 1448A-WT-KB | OK | 145.769 | 35.3951 | -2.04206 | -1.53531 | 0.12005 | 0.327912 |
| PSPPH_0053 | hypothetical protein | chromosome:55282-55510 | 1448A-rhpS-KB | 1448A-WT-KB | OK | 447.402 | 108.451 | -2.04453 | -1.23147 | 0.1367 | 0.351242 |
| PSPPH_1204 | hypothetical protein | chromosome:1415326-1415608 | 1448A-rhpS-KB | 1448A-WT-KB | OK | 687.346 | 166.285 | -2.04738 | -1.90229 | 0.02835 | 0.139563 |
| PSPPH_2721 | hypothetical protein | chromosome:3148290-3148500 | 1448A-rhpS-KB | 1448A-WT-KB | NOTEST | 65.2531 | 15.7823 | -2.04774 | 0 | 1 | 1 |
| aspA | aspartate ammonia-lyase | chromosome:5821190-5822615 | 1448A-rhpS-KB | 1448A-WT-KB | OK | 402.669 | 97.3549 | -2.04827 | -4.41429 | 0.00005 | 0.00121565 |
| PSPPH_0890 | carbonic anhydrase | chromosome:1062138-1062777 | 1448A-rhpS-KB | 1448A-WT-KB | OK | 835.052 | 201.356 | -2.05212 | -3.86694 | 0.00005 | 0.00121565 |
| fliS | flagellar protein FliS | chromosome:3917309-3917708 | 1448A-rhpS-KB | 1448A-WT-KB | OK | 620.268 | 149 | -2.05758 | -2.45755 | 0.0055 | 0.0455023 |
| PSPPH_4037 | hypothetical protein | chromosome:4611822-4612182 | 1448A-rhpS-KB | 1448A-WT-KB | OK | 868.506 | 208.126 | -2.06108 | -2.75959 | 0.0024 | 0.0256048 |
| PSPPH_4291 | lipoprotein | chromosome:4898827-4899181 | 1448A-rhpS-KB | 1448A-WT-KB | OK | 157.218 | 37.394 | -2.07189 | -1.15968 | 0.14355 | 0.361301 |
| PSPPH_1235 | isochorismatase | chromosome:1440876-1442632 | 1448A-rhpS-KB | 1448A-WT-KB | NOTEST | 31.8519 | 7.54438 | -2.07791 | 0 | 1 | 1 |
| PSPPH_4403 | hypothetical protein | chromosome:5025861-5027200 | 1448A-rhpS-KB | 1448A-WT-KB | NOTEST | 164.327 | 38.905 | -2.07854 | 0 | 1 | 1 |
| PSPPH_1553 | hypothetical protein | chromosome:1804730-1805231 | 1448A-rhpS-KB | 1448A-WT-KB | OK | 114.622 | 27.0511 | -2.08313 | -1.36208 | 0.0843 | 0.268528 |
| dusB | tRNA-dihydrouridine synthase B | chromosome:5079899-5081230 | 1448A-rhpS-KB | 1448A-WT-KB | OK | 366.384 | 85.5279 | -2.09889 | -3.3877 | 0.0002 | 0.00374241 |
| PSPPH_2588 | mutT/nudix family protein | chromosome:2979803-2980166 | 1448A-rhpS-KB | 1448A-WT-KB | OK | 393.245 | 91.2025 | -2.10828 | -1.90543 | 0.0279 | 0.137918 |
| kptA | RNA 2'-phosphotransferase | chromosome:4994086-4994653 | 1448A-rhpS-KB | 1448A-WT-KB | OK | 215.573 | 49.6847 | -2.1173 | -1.97211 | 0.02505 | 0.12919 |
| PSPPH_1375 | hypothetical protein | chromosome:1596629-1596812 | 1448A-rhpS-KB | 1448A-WT-KB | OK | 2231.3 | 511.511 | -2.12505 | -1.96571 | 0.02705 | 0.135782 |
| PSPPH_4063 | amino acid transporter LysE | chromosome:4644443-4645046 | 1448A-rhpS-KB | 1448A-WT-KB | OK | 377.564 | 86.4478 | -2.12682 | -2.72464 | 0.00425 | 0.0376911 |
| PSPPH_3529 | hypothetical protein | chromosome:4069213-4069480 | 1448A-rhpS-KB | 1448A-WT-KB | OK | 1712.62 | 390.192 | -2.13395 | -2.82165 | 0.0019 | 0.0218347 |
| PSPPH_3474 | hypothetical protein | chromosome:4013028-4015435 | 1448A-rhpS-KB | 1448A-WT-KB | OK | 53.2353 | 12.0728 | -2.14062 | -0.634649 | 0.3817 | 0.614226 |
| PSPPH_2543 | response regulator | chromosome:2930403-2931572 | 1448A-rhpS-KB | 1448A-WT-KB | OK | 68.8224 | 15.4334 | -2.15683 | -0.791771 | 0.30015 | 0.534409 |
| PSPPH_1394 | ATP-dependent hsl protease ATP-binding subunit hslU (ATP-bindingprotein lapA) | chromosome:1619141-1619390 | 1448A-rhpS-KB | 1448A-WT-KB | OK | 274.358 | 61.5218 | -2.15689 | -1.11791 | 0.1712 | 0.398612 |
| PSPPH_1594 | ABC transporter substrate-binding protein | chromosome:1848100-1849168 | 1448A-rhpS-KB | 1448A-WT-KB | OK | 335.62 | 74.8516 | -2.16472 | -3.80507 | 0.00005 | 0.00121565 |
| PSPPH_0628 | hypothetical protein | chromosome:741563-744394 | 1448A-rhpS-KB | 1448A-WT-KB | OK | 862.585 | 191.789 | -2.16915 | -4.68443 | 0.00005 | 0.00121565 |
| PSPPH_1104 | HlyD family secretion protein | chromosome:1295168-1299907 | 1448A-rhpS-KB | 1448A-WT-KB | OK | 86.0951 | 19.041 | -2.17682 | -1.32891 | 0.20835 | 0.444828 |
| PSPPH_0859 | hypothetical protein | chromosome:1026633-1029748 | 1448A-rhpS-KB | 1448A-WT-KB | OK | 1186.51 | 260.759 | -2.18593 | -1.10318 | 0.17575 | 0.404723 |
| aer1 | aerotaxis receptor Aer | chromosome:1770015-1771581 | 1448A-rhpS-KB | 1448A-WT-KB | OK | 124.181 | 27.1288 | -2.19455 | -2.9157 | 0.00245 | 0.0257538 |
| PSPPH_1253 | lipoprotein | chromosome:1459470-1460318 | 1448A-rhpS-KB | 1448A-WT-KB | OK | 106.464 | 23.2232 | -2.19672 | -0.923592 | 0.31175 | 0.547785 |
| PSPPH_2219 | AsnC family transcriptional regulator | chromosome:2575401-2575869 | 1448A-rhpS-KB | 1448A-WT-KB | OK | 76.0304 | 16.5558 | -2.19924 | -1.05172 | 0.1806 | 0.410499 |
| PSPPH_1784 | hypothetical protein | chromosome:2090031-2090826 | 1448A-rhpS-KB | 1448A-WT-KB | OK | 96.5264 | 20.8667 | -2.20972 | -1.70656 | 0.06345 | 0.231259 |
| PSPPH_0154 | hypothetical protein | chromosome:182434-182668 | 1448A-rhpS-KB | 1448A-WT-KB | OK | 1136.65 | 244.746 | -2.21543 | -2.03735 | 0.0239 | 0.125616 |
| PSPPH_1419 | diguanylate cyclase | chromosome:1644047-1646339 | 1448A-rhpS-KB | 1448A-WT-KB | OK | 212.651 | 45.6894 | -2.21856 | -4.35092 | 0.00005 | 0.00121565 |
| hopAK1 | type III helper protein HopAK1 | chromosome:1652069-1653644 | 1448A-rhpS-KB | 1448A-WT-KB | OK | 478.247 | 101.898 | -2.23064 | -5.27345 | 0.00005 | 0.00121565 |
| PSPPH_2574 | hypothetical protein | chromosome:2968560-2969046 | 1448A-rhpS-KB | 1448A-WT-KB | OK | 210.612 | 44.8201 | -2.23237 | -1.81065 | 0.03995 | 0.177156 |
| rpsT | 30S ribosomal protein S20 | chromosome:840942-841221 | 1448A-rhpS-KB | 1448A-WT-KB | OK | 2941.64 | 622.593 | -2.24026 | -3.86293 | 0.00005 | 0.00121565 |
| PSPPH_2655 | acetyltransferase | chromosome:3079010-3079577 | 1448A-rhpS-KB | 1448A-WT-KB | NOTEST | 42.3694 | 8.96672 | -2.24037 | 0 | 1 | 1 |
| PSPPH_2093 | exopolysaccharide production protein ExoZ | chromosome:2452526-2453597 | 1448A-rhpS-KB | 1448A-WT-KB | OK | 134.475 | 28.3884 | -2.24397 | -2.32714 | 0.0111 | 0.0752778 |
| glgA | glycogen synthase | chromosome:2610452-2611910 | 1448A-rhpS-KB | 1448A-WT-KB | OK | 251.2 | 52.9875 | -2.24512 | -3.79046 | 0.00005 | 0.00121565 |
| PSPPH_2365 | ribose ABC transporter permease | chromosome:2739811-2743357 | 1448A-rhpS-KB | 1448A-WT-KB | NOTEST | 33.3897 | 7.03128 | -2.24754 | 0 | 1 | 1 |
| PSPPH_1637 | hypothetical protein | chromosome:1901078-1901606 | 1448A-rhpS-KB | 1448A-WT-KB | OK | 2887.59 | 605.295 | -2.25416 | -5.72391 | 0.00005 | 0.00121565 |
| dxnG | 3-oxoadipate CoA-succinyl transferase subunit alpha | chromosome:3659568-3660270 | 1448A-rhpS-KB | 1448A-WT-KB | OK | 425.112 | 88.6102 | -2.2623 | -3.44687 | 0.00025 | 0.0043799 |
| PSPPH_1457 | hypothetical protein | chromosome:1699419-1699686 | 1448A-rhpS-KB | 1448A-WT-KB | NOTEST | 66.6041 | 13.8371 | -2.26707 | 0 | 1 | 1 |
| PSPPH_1544 | gas vesicle protein | chromosome:1795931-1796414 | 1448A-rhpS-KB | 1448A-WT-KB | OK | 1224.74 | 253.93 | -2.26997 | -4.04779 | 0.0001 | 0.00221988 |
| PSPPH_1216 | glycosyl transferase family protein | chromosome:1424582-1425467 | 1448A-rhpS-KB | 1448A-WT-KB | OK | 510.858 | 105.58 | -2.27459 | -4.02836 | 0.00005 | 0.00121565 |
| PSPPH_2228 | hypothetical protein | chromosome:2589062-2589428 | 1448A-rhpS-KB | 1448A-WT-KB | OK | 2773.89 | 564.409 | -2.2971 | -4.71782 | 0.00005 | 0.00121565 |
| PSPPH_4974 | hypothetical protein | chromosome:5642992-5643945 | 1448A-rhpS-KB | 1448A-WT-KB | OK | 885.407 | 177.538 | -2.31821 | -3.74427 | 0.00005 | 0.00121565 |
| PSPPH_3780 | hypothetical protein | chromosome:4329400-4330035 | 1448A-rhpS-KB | 1448A-WT-KB | OK | 617.015 | 123.007 | -2.32656 | -1.16314 | 0.1658 | 0.392998 |
| rmf | ribosome modulation factor-like protein | chromosome:2437665-2437992 | 1448A-rhpS-KB | 1448A-WT-KB | OK | 3597.45 | 713.551 | -2.33389 | -4.80278 | 0.00005 | 0.00121565 |
| PSPPH_4331 | hypothetical protein | chromosome:4952053-4952428 | 1448A-rhpS-KB | 1448A-WT-KB | OK | 282.207 | 55.7868 | -2.33876 | -1.72281 | 0.05525 | 0.21413 |
| PSPPH_0608 | pancortin-3 | chromosome:718228-719993 | 1448A-rhpS-KB | 1448A-WT-KB | OK | 609.305 | 120.348 | -2.33995 | -2.72737 | 0.0062 | 0.0492418 |
| PSPPH_3685 | hypothetical protein | chromosome:4232943-4233237 | 1448A-rhpS-KB | 1448A-WT-KB | OK | 370.328 | 72.3809 | -2.35512 | -1.5048 | 0.1003 | 0.294217 |
| PSPPH_1367 | hypothetical protein | chromosome:1586456-1587335 | 1448A-rhpS-KB | 1448A-WT-KB | OK | 209.988 | 40.8256 | -2.36276 | -2.78142 | 0.00425 | 0.0376911 |
| PSPPH_4839 | hypothetical protein | chromosome:5498640-5498970 | 1448A-rhpS-KB | 1448A-WT-KB | OK | 102.171 | 19.3056 | -2.4039 | -1.12681 | 0.23015 | 0.466972 |
| tonB3 | ferric siderophore transporter, periplasmic energy transduction protein TonB | chromosome:3338452-3339283 | 1448A-rhpS-KB | 1448A-WT-KB | NOTEST | 34.0411 | 6.26912 | -2.44094 | 0 | 1 | 1 |
| cspD | cold shock domain-containing protein CspD | chromosome:3596371-3596656 | 1448A-rhpS-KB | 1448A-WT-KB | OK | 381.723 | 70.0706 | -2.44564 | -1.46938 | 0.1051 | 0.303415 |
| PSPPH_3293 | hypothetical protein | chromosome:3817141-3817747 | 1448A-rhpS-KB | 1448A-WT-KB | OK | 836.241 | 146.995 | -2.50815 | -4.166 | 0.0001 | 0.00221988 |
| PSPPH_1685 | hypothetical protein | chromosome:1949219-1949492 | 1448A-rhpS-KB | 1448A-WT-KB | OK | 179.307 | 31.2814 | -2.51905 | -1.39203 | 0.22575 | 0.463474 |
| PSPPH_4183 | hypothetical protein | chromosome:4767726-4771078 | 1448A-rhpS-KB | 1448A-WT-KB | OK | 107.25 | 18.5292 | -2.53311 | -0.917372 | 0.428 | 0.653427 |
| PSPPH_1789 | hypothetical protein | chromosome:2095243-2095528 | 1448A-rhpS-KB | 1448A-WT-KB | OK | 1140.17 | 196.078 | -2.53975 | -2.63905 | 0.0067 | 0.0516073 |
| PSPPH_2066 | CmpX | chromosome:2421642-2422467 | 1448A-rhpS-KB | 1448A-WT-KB | OK | 1223.84 | 210.308 | -2.54084 | -5.52834 | 0.00005 | 0.00121565 |
| PSPPH_4249 | hypothetical protein | chromosome:4848142-4848382 | 1448A-rhpS-KB | 1448A-WT-KB | OK | 109.153 | 18.0986 | -2.59241 | -1.09227 | 0.2906 | 0.525876 |
| PSPPH_1506 | hypothetical protein | chromosome:1747017-1747245 | 1448A-rhpS-KB | 1448A-WT-KB | OK | 136.682 | 22.6366 | -2.5941 | -1.30376 | 0.32745 | 0.565092 |
| PSPPH_2240 | hypothetical protein | chromosome:2597977-2599996 | 1448A-rhpS-KB | 1448A-WT-KB | OK | 65.5342 | 10.6003 | -2.62814 | -1.26387 | 0.18135 | 0.411259 |
| PSPPH_2951 | hypothetical protein | chromosome:3425803-3426640 | 1448A-rhpS-KB | 1448A-WT-KB | NOTEST | 28.2152 | 4.42738 | -2.67195 | 0 | 1 | 1 |
| PSPPH_3958 | hypothetical protein | chromosome:4518910-4521198 | 1448A-rhpS-KB | 1448A-WT-KB | OK | 493.289 | 73.7526 | -2.74167 | -1.09996 | 0.2275 | 0.46409 |
| PSPPH_1423 | hypothetical protein | chromosome:1651695-1651851 | 1448A-rhpS-KB | 1448A-WT-KB | OK | 1038.47 | 153.698 | -2.7563 | -2.34604 | 0.25255 | 0.487899 |
| PSPPH_0044 | lipoprotein | chromosome:46834-47269 | 1448A-rhpS-KB | 1448A-WT-KB | OK | 83.3049 | 11.4618 | -2.86157 | -1.25624 | 0.21915 | 0.456215 |
| PSPPH_2383 | lipoprotein | chromosome:2764868-2765246 | 1448A-rhpS-KB | 1448A-WT-KB | OK | 596.712 | 81.983 | -2.86364 | -2.70456 | 0.0092 | 0.0653694 |
| PSPPH_0821 | type IV pilin | chromosome:982618-983014 | 1448A-rhpS-KB | 1448A-WT-KB | OK | 1571.42 | 211.508 | -2.89328 | -4.37614 | 0.00005 | 0.00121565 |
| PSPPH_3981 | 3-hydroxyacyl-CoA-acyl carrier protein transferase | chromosome:4546905-4547787 | 1448A-rhpS-KB | 1448A-WT-KB | OK | 371.161 | 49.6942 | -2.9009 | -3.86592 | 0.0004 | 0.00632566 |
| PSPPH_4393 | hypothetical protein | chromosome:5016831-5017161 | 1448A-rhpS-KB | 1448A-WT-KB | OK | 314.692 | 41.5094 | -2.92243 | -1.65749 | 0.0995 | 0.292927 |
| PSPPH_4158 | hypothetical protein | chromosome:4740788-4740989 | 1448A-rhpS-KB | 1448A-WT-KB | OK | 149.894 | 19.3803 | -2.95128 | -1.04839 | 0.32865 | 0.565253 |
| glpD | glycerol-3-phosphate dehydrogenase | chromosome:4459822-4461361 | 1448A-rhpS-KB | 1448A-WT-KB | OK | 2735.39 | 351.303 | -2.96096 | -9.78682 | 0.00005 | 0.00121565 |
| PSPPH_2003 | sensor histidine kinase | chromosome:2351513-2353285 | 1448A-rhpS-KB | 1448A-WT-KB | OK | 409.358 | 51.5678 | -2.98882 | -3.503 | 0.0002 | 0.00374241 |
| PSPPH_1841 | auxin-binding protein | chromosome:2137984-2138488 | 1448A-rhpS-KB | 1448A-WT-KB | OK | 319.362 | 37.6048 | -3.0862 | -2.45768 | 0.02685 | 0.135539 |
| PSPPH_0273 | hypothetical protein | chromosome:314703-315354 | 1448A-rhpS-KB | 1448A-WT-KB | OK | 418.778 | 47.4622 | -3.14133 | -3.39483 | 0.0021 | 0.0233087 |
| PSPPH_2787 | dipeptide/oligopeptide/nickel ABC transporter ATPase | chromosome:3224066-3225958 | 1448A-rhpS-KB | 1448A-WT-KB | NOTEST | 30.3238 | 3.42518 | -3.1462 | 0 | 1 | 1 |
| pstS | phosphate ABC transporter substrate-binding protein | chromosome:3503612-3504632 | 1448A-rhpS-KB | 1448A-WT-KB | OK | 300.786 | 31.9615 | -3.23433 | -3.81382 | 0.0004 | 0.00632566 |
| PSPPH_2065 | crfX protein | chromosome:2421391-2421640 | 1448A-rhpS-KB | 1448A-WT-KB | OK | 2101.22 | 208.611 | -3.33234 | -3.14273 | 0.0034 | 0.0323181 |
| PSPPH_3234 | ISPsy18, transposase | chromosome:3748790-3750050 | 1448A-rhpS-KB | 1448A-WT-KB | NOTEST | 24.2722 | 2.21943 | -3.45104 | 0 | 1 | 1 |
| PSPPH_4711 | hypothetical protein | chromosome:5346761-5347043 | 1448A-rhpS-KB | 1448A-WT-KB | OK | 1479.63 | 128.162 | -3.5292 | -3.04513 | 0.0073 | 0.0552758 |
| PSPPH_2800 | PbsX family transcriptional regulator | chromosome:3241092-3241329 | 1448A-rhpS-KB | 1448A-WT-KB | OK | 269.687 | 22.2766 | -3.59769 | -1.728 | 0.25275 | 0.488022 |
| PSPPH_3327 | CAAX amino terminal protease | chromosome:3851432-3852224 | 1448A-rhpS-KB | 1448A-WT-KB | OK | 67.8014 | 4.25017 | -3.99572 | -1.75446 | 0.241 | 0.474564 |
| PSPPH_4486 | phosphate starvation-inducible protein PsiF | chromosome:5122070-5122382 | 1448A-rhpS-KB | 1448A-WT-KB | OK | 834.743 | 51.1886 | -4.02744 | -2.63603 | 0.0827 | 0.266279 |
| PSPPH_2825 | hypothetical protein | chromosome:3272036-3272318 | 1448A-rhpS-KB | 1448A-WT-KB | OK | 429.647 | 22.2291 | -4.27263 | -2.69413 | 0.2409 | 0.474564 |
| PSPPH_2004 | DNA-binding response regulator | chromosome:2351513-2353285 | 1448A-rhpS-KB | 1448A-WT-KB | OK | 3985.88 | 172.819 | -4.52757 | -8.9591 | 0.00005 | 0.00121565 |
| PSPPH_2910 | hypothetical protein | chromosome:3381044-3381590 | 1448A-rhpS-KB | 1448A-WT-KB | OK | 909.729 | 37.6276 | -4.59557 | -4.31024 | 0.00645 | 0.0506644 |
| PSPPH_A0125 | hypothetical protein | large_plasmid:106664-106814 | 1448A-rhpS-KB | 1448A-WT-KB | OK | 1737.08 | 55.9262 | -4.95699 | -3.01691 | 0.1841 | 0.412635 |
| PSPPH_1471 | mismatched base pair and cruciform DNA recognition protein | chromosome:1712060-1712252 | 1448A-rhpS-KB | 1448A-WT-KB | OK | 605.081 | 18.884 | -5.00189 | -1.65874 | 0.18415 | 0.412635 |
| PSPPH_2002 | calcium-binding protein | chromosome:2350639-2351398 | 1448A-rhpS-KB | 1448A-WT-KB | OK | 3945.17 | 79.4292 | -5.63427 | -8.97794 | 0.00005 | 0.00121565 |

**B. List of genes downregulated in *rhpS* mutant in KB**

| gene | annotation | locus | sample_1 | sample_2 | status | value_1 | value_2 | log2(fold_change) | test_stat | p_value | q_value |
| --- | --- | --- | --- | --- | --- | --- | --- | --- | --- | --- | --- |
| PSPPH_0971 | hypothetical protein | chromosome:1154480-1155806 | 1448A-rhpS-KB | 1448A-WT-KB | OK | 2.87274 | 69.5006 | 4.59653 | 1.67898 | 0.2263 | 0.463474 |
| PSPPH_4643 | copper-translocating P-type ATPase | chromosome:5280482-5283076 | 1448A-rhpS-KB | 1448A-WT-KB | OK | 19.6193 | 459.586 | 4.54999 | 4.44504 | 0.00425 | 0.0376911 |
| PSPPH_3002 | dipeptide ABC transporter ATP-binding protein | chromosome:3485100-3486720 | 1448A-rhpS-KB | 1448A-WT-KB | OK | 3.84743 | 77.3829 | 4.33005 | 2.26898 | 0.1369 | 0.351242 |
| PSPPH_3746 | hypothetical protein | chromosome:4297527-4299012 | 1448A-rhpS-KB | 1448A-WT-KB | OK | 2.98493 | 53.5218 | 4.16436 | 1.65147 | 0.2263 | 0.463474 |
| folK2 | 2-amino-4-hydroxy-6- hydroxymethyldihydropteridine pyrophosphokinase | chromosome:1022574-1024549 | 1448A-rhpS-KB | 1448A-WT-KB | OK | 8.21629 | 141.976 | 4.11101 | 1.27532 | 0.24205 | 0.475584 |
| PSPPH_0644 | polyamine ABC transporter substrate-binding protein | chromosome:762255-763278 | 1448A-rhpS-KB | 1448A-WT-KB | OK | 3.19747 | 50.1841 | 3.97223 | 1.32946 | 0.2263 | 0.463474 |
| PSPPH_1693 | hypothetical protein | chromosome:1955056-1957165 | 1448A-rhpS-KB | 1448A-WT-KB | NOTEST | 1.58305 | 24.6096 | 3.95844 | 0 | 1 | 1 |
| hutF | N-formimino-L-glutamate deiminase | chromosome:397567-398932 | 1448A-rhpS-KB | 1448A-WT-KB | OK | 86.4096 | 1273.67 | 3.88165 | 7.98019 | 0.00005 | 0.00121565 |
| PSPPH_4300 | hypothetical protein | chromosome:4911411-4912629 | 1448A-rhpS-KB | 1448A-WT-KB | OK | 7.99581 | 115.231 | 3.84915 | 2.55572 | 0.13745 | 0.352148 |
| hutU | urocanate hydratase | chromosome:5525339-5527037 | 1448A-rhpS-KB | 1448A-WT-KB | OK | 4.46052 | 59.8225 | 3.7454 | 1.97501 | 0.12955 | 0.340951 |
| PSPPH_4218 | hypothetical protein | chromosome:4806711-4810060 | 1448A-rhpS-KB | 1448A-WT-KB | OK | 3.77419 | 47.2789 | 3.64696 | 1.97829 | 0.17915 | 0.409126 |
| PSPPH_0670 | prophage PSPPH01 tail tape measure domain-containing protein | chromosome:785688-788396 | 1448A-rhpS-KB | 1448A-WT-KB | NOTEST | 2.11143 | 25.568 | 3.59805 | 0 | 1 | 1 |
| PSPPH_2789 | hypothetical protein | chromosome:3227654-3228959 | 1448A-rhpS-KB | 1448A-WT-KB | NOTEST | 3.12463 | 34.9996 | 3.48558 | 0 | 1 | 1 |
| folD2 | bifunctional 5,10-methylene-tetrahydrofolate dehydrogenase/ 5,10-methylene-tetrahydrofolate cyclohydrolase | chromosome:3435544-3438533 | 1448A-rhpS-KB | 1448A-WT-KB | NOTEST | 3.90654 | 43.7375 | 3.48491 | 0 | 1 | 1 |
| PSPPH_3921 | hypothetical protein | chromosome:4478406-4479204 | 1448A-rhpS-KB | 1448A-WT-KB | OK | 4.96586 | 54.392 | 3.45328 | 1.27371 | 0.22655 | 0.463474 |
| PSPPH_0346 | hypothetical protein | chromosome:394195-394729 | 1448A-rhpS-KB | 1448A-WT-KB | OK | 7.55438 | 81.3609 | 3.42895 | 1.26538 | 0.22675 | 0.463618 |
| PSPPH_2736 | xylulokinase | chromosome:3167121-3170908 | 1448A-rhpS-KB | 1448A-WT-KB | NOTEST | 2.0018 | 21.3274 | 3.41334 | 0 | 1 | 1 |
| pchA | salicylate biosynthesis isochorismate synthase | chromosome:3371026-3372738 | 1448A-rhpS-KB | 1448A-WT-KB | NOTEST | 2.15508 | 22.761 | 3.40075 | 0 | 1 | 1 |
| PSPPH_2828 | hypothetical protein | chromosome:3273219-3273924 | 1448A-rhpS-KB | 1448A-WT-KB | OK | 5.60746 | 58.6623 | 3.38701 | 1.23548 | 0.22645 | 0.463474 |
| PSPPH_4307 | pyruvate phosphate dikinase PEP/pyruvate binding subunit | chromosome:4920170-4922102 | 1448A-rhpS-KB | 1448A-WT-KB | OK | 25.0648 | 260.807 | 3.37925 | 4.82965 | 0.00005 | 0.00121565 |
| PSPPH_1098 | hypothetical protein | chromosome:1292036-1292939 | 1448A-rhpS-KB | 1448A-WT-KB | OK | 8.14307 | 83.0595 | 3.3505 | 1.86992 | 0.13995 | 0.354997 |
| PSPPH_3290 | iron ABC transporter substrate-binding protein | chromosome:3813266-3814091 | 1448A-rhpS-KB | 1448A-WT-KB | OK | 7.56845 | 76.8028 | 3.34309 | 1.72095 | 0.13995 | 0.354997 |
| PSPPH_2263 | cointegrate resolution protein T | chromosome:2625147-2626158 | 1448A-rhpS-KB | 1448A-WT-KB | OK | 5.68312 | 57.5486 | 3.34003 | 1.65134 | 0.13995 | 0.354997 |
| dctP | TRAP dicarboxylate transporter subunit DctP | chromosome:1109212-1110238 | 1448A-rhpS-KB | 1448A-WT-KB | OK | 5.82768 | 58.3843 | 3.32459 | 1.67428 | 0.13995 | 0.354997 |
| PSPPH_3553 | EmrB/QacA family drug resistance transporter | chromosome:4088552-4090091 | 1448A-rhpS-KB | 1448A-WT-KB | OK | 9.32804 | 90.6087 | 3.28 | 2.79495 | 0.0257 | 0.131782 |
| PSPPH_4269 | hypothetical protein | chromosome:4875483-4876623 | 1448A-rhpS-KB | 1448A-WT-KB | NOTEST | 2.96447 | 28.7359 | 3.27701 | 0 | 1 | 1 |
| citG | triphosphoribosyl-dephospho-CoA synthase | chromosome:495425-497977 | 1448A-rhpS-KB | 1448A-WT-KB | OK | 65.7854 | 637.68 | 3.27699 | 2.99963 | 0.03575 | 0.164865 |
| araH | L-arabinose transporter permease | chromosome:2895426-2896413 | 1448A-rhpS-KB | 1448A-WT-KB | NOTEST | 3.96966 | 37.101 | 3.22437 | 0 | 1 | 1 |
| PSPPH_4560 | filamentous hemagglutinin | chromosome:5198829-5204672 | 1448A-rhpS-KB | 1448A-WT-KB | NOTEST | 1.8315 | 17.0018 | 3.21459 | 0 | 1 | 1 |
| cobS | cobalamin synthase | chromosome:4240601-4241333 | 1448A-rhpS-KB | 1448A-WT-KB | OK | 5.57554 | 51.3134 | 3.20215 | 1.1812 | 0.22725 | 0.464048 |
| PSPPH_1476 | hypothetical protein | chromosome:1715738-1716239 | 1448A-rhpS-KB | 1448A-WT-KB | OK | 9.75801 | 88.9472 | 3.18829 | 1.30235 | 0.227 | 0.463864 |
| PSPPH_0645 | polyamine ABC transporter permease | chromosome:763355-764603 | 1448A-rhpS-KB | 1448A-WT-KB | NOTEST | 2.79948 | 25.4613 | 3.18508 | 0 | 1 | 1 |
| PSPPH_2689 | LysR family transcriptional regulator | chromosome:3113454-3114360 | 1448A-rhpS-KB | 1448A-WT-KB | NOTEST | 3.44746 | 30.889 | 3.16348 | 0 | 1 | 1 |
| PSPPH_1583 | hypothetical protein | chromosome:1837346-1837799 | 1448A-rhpS-KB | 1448A-WT-KB | OK | 11.3203 | 99.9235 | 3.14191 | 1.31372 | 0.22735 | 0.464048 |
| PSPPH_A0092 | hypothetical protein | large_plasmid:80324-81113 | 1448A-rhpS-KB | 1448A-WT-KB | OK | 20.9661 | 184.629 | 3.1385 | 2.3861 | 0.0274 | 0.13658 |
| PSPPH_3189 | iolD protein | chromosome:3698342-3700280 | 1448A-rhpS-KB | 1448A-WT-KB | NOTEST | 1.60504 | 14.0905 | 3.13404 | 0 | 1 | 1 |
| PSPPH_2795 | hypothetical protein | chromosome:3235531-3237817 | 1448A-rhpS-KB | 1448A-WT-KB | NOTEST | 4.80479 | 42.1416 | 3.1327 | 0 | 1 | 1 |
| PSPPH_2416 | pyridoxal-phosphate dependent enzyme family/ornithine cyclodeaminase | chromosome:2792903-2796835 | 1448A-rhpS-KB | 1448A-WT-KB | NOTEST | 1.37978 | 11.9528 | 3.11484 | 0 | 1 | 1 |
| PSPPH_0126 | hypothetical protein | chromosome:148074-150880 | 1448A-rhpS-KB | 1448A-WT-KB | OK | 23.7219 | 198.259 | 3.0631 | 1.71906 | 0.13895 | 0.354594 |
| PSPPH_4855 | alkane sulfonate ABC transporter substrate-binding protein | chromosome:5516629-5517565 | 1448A-rhpS-KB | 1448A-WT-KB | NOTEST | 3.35896 | 27.8506 | 3.05162 | 0 | 1 | 1 |
| PSPPH_2959 | hypothetical protein | chromosome:3434543-3435272 | 1448A-rhpS-KB | 1448A-WT-KB | NOTEST | 4.78934 | 39.6869 | 3.05076 | 0 | 1 | 1 |
| PSPPH_4311 | fatty acid desaturase | chromosome:4924294-4925431 | 1448A-rhpS-KB | 1448A-WT-KB | OK | 32.8931 | 271.12 | 3.04307 | 3.76052 | 0.00075 | 0.0101534 |
| PSPPH_0186 | citrate transporter | chromosome:223349-224657 | 1448A-rhpS-KB | 1448A-WT-KB | OK | 5.9873 | 48.6698 | 3.02305 | 1.59306 | 0.14035 | 0.354997 |
| PSPPH_3289 | dyp-type peroxidase | chromosome:3811914-3813237 | 1448A-rhpS-KB | 1448A-WT-KB | OK | 5.9095 | 47.8657 | 3.01788 | 1.59748 | 0.14035 | 0.354997 |
| PSPPH_4314 | hypothetical protein | chromosome:4925434-4927721 | 1448A-rhpS-KB | 1448A-WT-KB | OK | 34.7125 | 281.149 | 3.01781 | 2.03813 | 0.0365 | 0.16618 |
| PSPPH_1613 | hypothetical protein | chromosome:1870154-1873726 | 1448A-rhpS-KB | 1448A-WT-KB | NOTEST | 2.53068 | 20.3849 | 3.0099 | 0 | 1 | 1 |
| PSPPH_4299 | hypothetical protein | chromosome:4910032-4911277 | 1448A-rhpS-KB | 1448A-WT-KB | OK | 13.0377 | 104.137 | 2.99772 | 2.69436 | 0.03385 | 0.158766 |
| purU1 | formyltetrahydrofolate deformylase | chromosome:3435544-3438533 | 1448A-rhpS-KB | 1448A-WT-KB | NOTEST | 4.45248 | 35.3297 | 2.9882 | 0 | 1 | 1 |
| PSPPH_3053 | phospholipase/carboxylesterase | chromosome:3547549-3548251 | 1448A-rhpS-KB | 1448A-WT-KB | OK | 5.88861 | 44.9598 | 2.93263 | 1.08298 | 0.22975 | 0.466752 |
| dcd | deoxycytidine triphosphate deaminase | chromosome:4922182-4923258 | 1448A-rhpS-KB | 1448A-WT-KB | OK | 33.3399 | 254.331 | 2.93138 | 2.46568 | 0.1485 | 0.368569 |
| PSPPH_5181 | HemK family modification methylase | chromosome:5878502-5881096 | 1448A-rhpS-KB | 1448A-WT-KB | NOTEST | 3.56292 | 27.1358 | 2.92907 | 0 | 1 | 1 |
| PSPPH_4312 | hypothetical protein | chromosome:4925434-4927721 | 1448A-rhpS-KB | 1448A-WT-KB | OK | 66.2772 | 502.219 | 2.92173 | 2.4611 | 0.01895 | 0.106825 |
| PSPPH_0332 | D-methionine ABC transporter permease | chromosome:378442-380992 | 1448A-rhpS-KB | 1448A-WT-KB | OK | 6.7352 | 50.7252 | 2.91291 | 1.08653 | 0.1841 | 0.412635 |
| PSPPH_4869 | cadmium-translocating P-type ATPase | chromosome:5536701-5538966 | 1448A-rhpS-KB | 1448A-WT-KB | OK | 167.07 | 1241.47 | 2.89353 | 8.64775 | 0.00005 | 0.00121565 |
| mdcE | malonate decarboxylase subunit gamma | chromosome:497978-501483 | 1448A-rhpS-KB | 1448A-WT-KB | OK | 68.3765 | 507.979 | 2.8932 | 3.16146 | 0.0024 | 0.0256048 |
| PSPPH_3917 | hypothetical protein | chromosome:4475444-4476125 | 1448A-rhpS-KB | 1448A-WT-KB | OK | 6.05041 | 44.4734 | 2.87784 | 1.05985 | 0.22985 | 0.466752 |
| mdcD | malonate decarboxylase subunit beta | chromosome:497978-501483 | 1448A-rhpS-KB | 1448A-WT-KB | OK | 102.374 | 743.904 | 2.86127 | 2.87133 | 0.00395 | 0.0361982 |
| PSPPH_0741 | hypothetical protein | chromosome:866156-867844 | 1448A-rhpS-KB | 1448A-WT-KB | OK | 17.4992 | 125.989 | 2.84794 | 1.65287 | 0.07365 | 0.252615 |
| PSPPH_1493 | LysR family transcriptional regulator | chromosome:1734478-1735387 | 1448A-rhpS-KB | 1448A-WT-KB | OK | 13.2198 | 94.8202 | 2.8425 | 2.01163 | 0.04175 | 0.182187 |
| PSPPH_1035 | lipoprotein | chromosome:1223312-1224131 | 1448A-rhpS-KB | 1448A-WT-KB | OK | 73.9555 | 525.868 | 2.82997 | 4.28076 | 0.0001 | 0.00221988 |
| tonB1 | ferric siderophore ABC transporter substrate-binding protein | chromosome:230006-231175 | 1448A-rhpS-KB | 1448A-WT-KB | NOTEST | 4.31507 | 30.3079 | 2.81224 | 0 | 1 | 1 |
| cobN | cobaltochelatase subunit CobN | chromosome:2582359-2588302 | 1448A-rhpS-KB | 1448A-WT-KB | OK | 10.1729 | 71.1649 | 2.80644 | 2.9137 | 0.0131 | 0.0846644 |
| PSPPH_4278 | glycine/betaine ABC transporter substrate-binding protein | chromosome:4885932-4886826 | 1448A-rhpS-KB | 1448A-WT-KB | OK | 7.25557 | 50.612 | 2.80232 | 1.44041 | 0.15405 | 0.375563 |
| bdh2 | 3-oxoacyl-ACP reductase | chromosome:4550864-4551590 | 1448A-rhpS-KB | 1448A-WT-KB | OK | 12.2389 | 84.4861 | 2.78724 | 1.54835 | 0.15145 | 0.371505 |
| PSPPH_3259 | gluconate permease | chromosome:3777167-3778520 | 1448A-rhpS-KB | 1448A-WT-KB | OK | 43.2931 | 297.341 | 2.77991 | 4.09207 | 0.0001 | 0.00221988 |
| oadA | pyruvate carboxylase subunit B | chromosome:5831285-5833094 | 1448A-rhpS-KB | 1448A-WT-KB | OK | 235.202 | 1615.15 | 2.7797 | 8.57129 | 0.00005 | 0.00121565 |
| PSPPH_3458 | hypothetical protein | chromosome:3994850-3996239 | 1448A-rhpS-KB | 1448A-WT-KB | NOTEST | 4.77256 | 32.4431 | 2.76508 | 0 | 1 | 1 |
| PSPPH_1502 | short chain dehydrogenase | chromosome:1743644-1744460 | 1448A-rhpS-KB | 1448A-WT-KB | NOTEST | 5.11256 | 34.7392 | 2.76445 | 0 | 1 | 1 |
| hrpQ | type III secretion component protein HrpQ | chromosome:1499873-1502194 | 1448A-rhpS-KB | 1448A-WT-KB | NOTEST | 3.24426 | 22.0395 | 2.76413 | 0 | 1 | 1 |
| PSPPH_3545 | hypothetical protein | chromosome:4082613-4082913 | 1448A-rhpS-KB | 1448A-WT-KB | OK | 22.2787 | 146.603 | 2.71818 | 1.29018 | 0.2359 | 0.470195 |
| dctM | TRAP dicarboxylate transporter subunit DctM | chromosome:1107341-1108622 | 1448A-rhpS-KB | 1448A-WT-KB | NOTEST | 6.13262 | 39.6878 | 2.69412 | 0 | 1 | 1 |
| PSPPH_0416 | lipoprotein | chromosome:476539-481262 | 1448A-rhpS-KB | 1448A-WT-KB | OK | 8.18421 | 52.6517 | 2.68556 | 0.620687 | 0.2668 | 0.502394 |
| PSPPH_1242 | allophanate hydrolase | chromosome:1449050-1450868 | 1448A-rhpS-KB | 1448A-WT-KB | NOTEST | 1.62588 | 10.3876 | 2.67557 | 0 | 1 | 1 |
| PSPPH_4309 | deoxycytidine triphosphate deaminase | chromosome:4922182-4923258 | 1448A-rhpS-KB | 1448A-WT-KB | OK | 26.8952 | 170.4 | 2.66351 | 1.33964 | 0.0962 | 0.286994 |
| PSPPH_0972 | hypothetical protein | chromosome:1155815-1159604 | 1448A-rhpS-KB | 1448A-WT-KB | OK | 7.12271 | 44.8481 | 2.65455 | 2.93257 | 0.0032 | 0.0312481 |
| PSPPH_4626 | ABC transporter permease | chromosome:5261858-5263885 | 1448A-rhpS-KB | 1448A-WT-KB | NOTEST | 4.58544 | 28.8344 | 2.65266 | 0 | 1 | 1 |
| PSPPH_4663 | lipoprotein | chromosome:5299385-5301707 | 1448A-rhpS-KB | 1448A-WT-KB | OK | 25.5421 | 159.954 | 2.6467 | 1.09183 | 0.4572 | 0.675499 |
| PSPPH_4683 | O-antigen ABC transporter ATP-binding protein | chromosome:5318348-5319325 | 1448A-rhpS-KB | 1448A-WT-KB | NOTEST | 6.21487 | 38.8816 | 2.64529 | 0 | 1 | 1 |
| PSPPH_5085 | HAD family hydrolase | chromosome:5769487-5770177 | 1448A-rhpS-KB | 1448A-WT-KB | OK | 70.8776 | 435.88 | 2.62053 | 3.54034 | 0.00085 | 0.01121 |
| PSPPH_0341 | cystine ABC transporter permease | chromosome:388975-390438 | 1448A-rhpS-KB | 1448A-WT-KB | OK | 45.2576 | 277.839 | 2.61802 | 1.96789 | 0.0302 | 0.145661 |
| PSPPH_4499 | urease accessory protein | chromosome:5134625-5135198 | 1448A-rhpS-KB | 1448A-WT-KB | OK | 7.94683 | 48.66 | 2.61428 | 0.999887 | 0.23785 | 0.471217 |
| glcE | glycolate oxidase FAD binding subunit | chromosome:3769665-3772223 | 1448A-rhpS-KB | 1448A-WT-KB | NOTEST | 3.35287 | 20.4415 | 2.60803 | 0 | 1 | 1 |
| PSPPH_4526 | sensory box protein | chromosome:5159385-5162295 | 1448A-rhpS-KB | 1448A-WT-KB | OK | 9.239 | 56.086 | 2.60183 | 2.78587 | 0.0064 | 0.0503824 |
| PSPPH_3576 | dihydroxy-acid dehydratase | chromosome:4120398-4122141 | 1448A-rhpS-KB | 1448A-WT-KB | OK | 13.6866 | 82.5751 | 2.59294 | 2.78502 | 0.00585 | 0.0471962 |
| PSPPH_4715 | acyl-CoA dehydrogenase | chromosome:5351933-5353739 | 1448A-rhpS-KB | 1448A-WT-KB | OK | 684.021 | 4125.42 | 2.59243 | 9.88756 | 0.00005 | 0.00121565 |
| flgF | flagellar basal body rod protein FlgF | chromosome:3936649-3937838 | 1448A-rhpS-KB | 1448A-WT-KB | NOTEST | 4.83373 | 29.1168 | 2.59064 | 0 | 1 | 1 |
| aer2 | aerotaxis receptor Aer | chromosome:3852320-3853886 | 1448A-rhpS-KB | 1448A-WT-KB | NOTEST | 1.87274 | 11.0545 | 2.56141 | 0 | 1 | 1 |
| PSPPH_4242 | sensor histidine kinase/response regulator | chromosome:4839175-4840744 | 1448A-rhpS-KB | 1448A-WT-KB | NOTEST | 6.29405 | 37.0919 | 2.55904 | 0 | 1 | 1 |
| accC1 | acetyl-CoA carboxylase biotin carboxylase subunit | chromosome:2838562-2841952 | 1448A-rhpS-KB | 1448A-WT-KB | NOTEST | 2.5212 | 14.7353 | 2.54709 | 0 | 1 | 1 |
| PSPPH_2631 | LysR family transcriptional regulator | chromosome:3036057-3036987 | 1448A-rhpS-KB | 1448A-WT-KB | OK | 7.85422 | 45.5862 | 2.53706 | 1.36765 | 0.16615 | 0.392998 |
| PSPPH_5077 | hypothetical protein | chromosome:5759629-5760340 | 1448A-rhpS-KB | 1448A-WT-KB | OK | 12.3104 | 71.1201 | 2.53038 | 1.49493 | 0.16555 | 0.392998 |
| PSPPH_2819 | Rhs family protein | chromosome:3264779-3266623 | 1448A-rhpS-KB | 1448A-WT-KB | OK | 9.87305 | 56.4495 | 2.51539 | 1.16826 | 0.10865 | 0.309168 |
| PSPPH_0697 | MutT/nudix family protein | chromosome:818823-819228 | 1448A-rhpS-KB | 1448A-WT-KB | OK | 11.4923 | 65.5155 | 2.51117 | 0.967644 | 0.24405 | 0.477414 |
| PSPPH_2980 | two-component system sensor protein | chromosome:3460645-3461602 | 1448A-rhpS-KB | 1448A-WT-KB | NOTEST | 3.2892 | 18.7462 | 2.51079 | 0 | 1 | 1 |
| dppC | dipeptide ABC transporter permease | chromosome:4867235-4869168 | 1448A-rhpS-KB | 1448A-WT-KB | OK | 52.1485 | 295.197 | 2.50098 | 3.06347 | 0.00305 | 0.0307062 |
| PSPPH_2764 | sensory box protein/methyl-accepting chemotaxis protein | chromosome:3199815-3201132 | 1448A-rhpS-KB | 1448A-WT-KB | NOTEST | 2.76519 | 15.5379 | 2.49034 | 0 | 1 | 1 |
| PSPPH_3161 | D-isomer specific 2-hydroxyacid dehydrogenase | chromosome:3663846-3664779 | 1448A-rhpS-KB | 1448A-WT-KB | OK | 48.8934 | 274.599 | 2.48961 | 3.34345 | 0.0011 | 0.0137462 |
| PSPPH_3444 | hypothetical protein | chromosome:3978238-3980249 | 1448A-rhpS-KB | 1448A-WT-KB | NOTEST | 7.05408 | 39.4789 | 2.48455 | 0 | 1 | 1 |
| PSPPH_0421 | 4-hydroxybenzoyl-CoA thioesterase | chromosome:482647-490911 | 1448A-rhpS-KB | 1448A-WT-KB | OK | 21.7057 | 120.612 | 2.47423 | 1.26936 | 0.2361 | 0.470195 |
| hutH1 | histidine ammonia-lyase | chromosome:5529883-5531425 | 1448A-rhpS-KB | 1448A-WT-KB | OK | 15.0248 | 82.9653 | 2.46516 | 2.4826 | 0.0083 | 0.061544 |
| PSPPH_3467 | 3-hydroxyisobutyrate dehydrogenase | chromosome:4005885-4006770 | 1448A-rhpS-KB | 1448A-WT-KB | NOTEST | 7.5876 | 41.8338 | 2.46295 | 0 | 1 | 1 |
| cycH | cytochrome c-type biogenesis protein CycH | chromosome:3829675-3831842 | 1448A-rhpS-KB | 1448A-WT-KB | NOTEST | 8.00506 | 44.1143 | 2.46226 | 0 | 1 | 1 |
| PSPPH_2755 | achromobactin biosynthetic protein AcsB | chromosome:3189418-3192045 | 1448A-rhpS-KB | 1448A-WT-KB | NOTEST | 4.37562 | 24.0812 | 2.46035 | 0 | 1 | 1 |
| PSPPH_1009 | hypothetical protein | chromosome:1195847-1202294 | 1448A-rhpS-KB | 1448A-WT-KB | OK | 25.1756 | 137.413 | 2.44842 | 1.13165 | 0.257 | 0.491712 |
| PSPPH_3751 | glyoxalase | chromosome:4303748-4304183 | 1448A-rhpS-KB | 1448A-WT-KB | OK | 25.5765 | 139.215 | 2.44443 | 1.50039 | 0.16475 | 0.392283 |
| PSPPH_4306 | hypothetical protein | chromosome:4916042-4920156 | 1448A-rhpS-KB | 1448A-WT-KB | OK | 44.3615 | 240.49 | 2.4386 | 3.26025 | 0.0021 | 0.0233087 |
| nhaA | pH-dependent sodium/proton antiporter | chromosome:4827571-4828750 | 1448A-rhpS-KB | 1448A-WT-KB | NOTEST | 6.75171 | 36.3417 | 2.4283 | 0 | 1 | 1 |
| PSPPH_0103 | hypothetical protein | chromosome:116301-116691 | 1448A-rhpS-KB | 1448A-WT-KB | OK | 14.9616 | 80.2145 | 2.42259 | 1.04883 | 0.2469 | 0.480621 |
| pcaK | 4-hydroxybenzoate transporter | chromosome:2456424-2457774 | 1448A-rhpS-KB | 1448A-WT-KB | OK | 8.61019 | 46.0123 | 2.4179 | 1.64749 | 0.06005 | 0.224587 |
| PSPPH_3920 | hypothetical protein | chromosome:4477669-4478281 | 1448A-rhpS-KB | 1448A-WT-KB | NOTEST | 6.82865 | 36.3593 | 2.41265 | 0 | 1 | 1 |
| PSPPH_3316 | flagellar biosynthetic FlhB domain-containing protein | chromosome:3836825-3838786 | 1448A-rhpS-KB | 1448A-WT-KB | OK | 18.6733 | 99.344 | 2.41146 | 1.03213 | 0.2868 | 0.522725 |
| PSPPH_2215 | major facilitator superfamily transporter | chromosome:2571872-2573102 | 1448A-rhpS-KB | 1448A-WT-KB | NOTEST | 3.39931 | 18.0474 | 2.40848 | 0 | 1 | 1 |
| PSPPH_1339 | cytidine/deoxycytidylate deaminase | chromosome:1553501-1561017 | 1448A-rhpS-KB | 1448A-WT-KB | OK | 9.04458 | 47.9338 | 2.40592 | 0.509811 | 0.51335 | 0.718928 |
| sun | sun protein | chromosome:22798-26456 | 1448A-rhpS-KB | 1448A-WT-KB | OK | 56.6099 | 299.878 | 2.40525 | 2.81738 | 0.0048 | 0.0414377 |
| PSPPH_2428 | 3-alpha-hydroxysteroid dehydrogenase | chromosome:2808477-2809245 | 1448A-rhpS-KB | 1448A-WT-KB | NOTEST | 5.46692 | 28.8005 | 2.3973 | 0 | 1 | 1 |
| PSPPH_0704 | phosphonate ABC transporter permease | chromosome:827742-830778 | 1448A-rhpS-KB | 1448A-WT-KB | NOTEST | 4.43963 | 23.3467 | 2.39471 | 0 | 1 | 1 |
| rrmA | ribosomal RNA large subunit methyltransferase A | chromosome:4406709-4408670 | 1448A-rhpS-KB | 1448A-WT-KB | OK | 37.0614 | 194.494 | 2.39173 | 1.91933 | 0.03185 | 0.151372 |
| adhC | alcohol dehydrogenase | chromosome:4367307-4368420 | 1448A-rhpS-KB | 1448A-WT-KB | OK | 158.929 | 830.291 | 2.38523 | 5.46809 | 0.00005 | 0.00121565 |
| PSPPH_4310 | fatty acid desaturase | chromosome:4923305-4924289 | 1448A-rhpS-KB | 1448A-WT-KB | OK | 29.5007 | 152.99 | 2.37462 | 2.34087 | 0.00985 | 0.0686236 |
| cumA | multicopper oxidase | chromosome:1553501-1561017 | 1448A-rhpS-KB | 1448A-WT-KB | NOTEST | 4.69381 | 24.1561 | 2.36356 | 0 | 1 | 1 |
| PSPPH_4082 | MGMT family protein | chromosome:4664259-4664616 | 1448A-rhpS-KB | 1448A-WT-KB | OK | 14.9121 | 76.6663 | 2.36211 | 0.965567 | 0.2623 | 0.496799 |
| PSPPH_3341 | VOMI family protein | chromosome:3869349-3870468 | 1448A-rhpS-KB | 1448A-WT-KB | NOTEST | 7.17796 | 36.8975 | 2.36188 | 0 | 1 | 1 |
| PSPPH_0650 | autotransporting lipase | chromosome:768943-770866 | 1448A-rhpS-KB | 1448A-WT-KB | OK | 14.5916 | 74.8519 | 2.3589 | 2.57674 | 0.00595 | 0.0477872 |
| PSPPH_3070 | aldehyde dehydrogenase | chromosome:3565738-3567322 | 1448A-rhpS-KB | 1448A-WT-KB | OK | 62.4686 | 319.626 | 2.35518 | 4.40331 | 0.00005 | 0.00121565 |
| PSPPH_3578 | hypothetical protein | chromosome:4123260-4125135 | 1448A-rhpS-KB | 1448A-WT-KB | NOTEST | 5.48567 | 27.9326 | 2.34821 | 0 | 1 | 1 |
| nhaA | pH-dependent sodium/proton antiporter | chromosome:394834-396010 | 1448A-rhpS-KB | 1448A-WT-KB | OK | 12.3585 | 62.8143 | 2.34558 | 1.92192 | 0.05315 | 0.210328 |
| PSPPH_3694 | alpha-ribazole-5'-phosphate phosphatase | chromosome:4241334-4243474 | 1448A-rhpS-KB | 1448A-WT-KB | OK | 15.3101 | 77.6417 | 2.34235 | 1.08716 | 0.1809 | 0.410499 |
| marR | multiple antibiotic resistance operon repressor MarR | chromosome:4091439-4091928 | 1448A-rhpS-KB | 1448A-WT-KB | OK | 53.0836 | 268.299 | 2.33751 | 1.99837 | 0.01875 | 0.106484 |
| PSPPH_5155 | hypothetical protein | chromosome:5846185-5846560 | 1448A-rhpS-KB | 1448A-WT-KB | OK | 13.175 | 66.5419 | 2.33646 | 0.915839 | 0.26135 | 0.495715 |
| PSPPH_1142 | hypothetical protein | chromosome:1340645-1341851 | 1448A-rhpS-KB | 1448A-WT-KB | OK | 10.0266 | 50.5733 | 2.33454 | 1.57435 | 0.06015 | 0.224587 |
| PSPPH_4979 | prophage PSPPH06 reverse transcriptase/maturase | chromosome:5648996-5650433 | 1448A-rhpS-KB | 1448A-WT-KB | OK | 22.2782 | 112.338 | 2.33414 | 2.47345 | 0.00505 | 0.0427694 |
| PSPPH_2367 | hypothetical protein | chromosome:2744885-2745851 | 1448A-rhpS-KB | 1448A-WT-KB | NOTEST | 3.67667 | 18.5228 | 2.33283 | 0 | 1 | 1 |
| PSPPH_3148 | group 1 glycosyl transferase | chromosome:3651596-3652712 | 1448A-rhpS-KB | 1448A-WT-KB | NOTEST | 2.51867 | 12.672 | 2.33091 | 0 | 1 | 1 |
| cpoF | arylesterase | chromosome:5154048-5155128 | 1448A-rhpS-KB | 1448A-WT-KB | NOTEST | 6.24185 | 31.2192 | 2.32239 | 0 | 1 | 1 |
| PSPPH_2957 | Mn2+/Fe2+ transporter | chromosome:3430866-3432168 | 1448A-rhpS-KB | 1448A-WT-KB | NOTEST | 2.88115 | 14.2815 | 2.30943 | 0 | 1 | 1 |
| PSPPH_0041 | luciferase | chromosome:44836-45838 | 1448A-rhpS-KB | 1448A-WT-KB | OK | 77.4305 | 383.181 | 2.30705 | 3.83565 | 0.00005 | 0.00121565 |
| avrD1 | syringolide biosynthetic protein AvrD1 | large_plasmid:96850-97786 | 1448A-rhpS-KB | 1448A-WT-KB | OK | 38.3611 | 189.275 | 2.30276 | 2.65821 | 0.0042 | 0.0376211 |
| PSPPH_4740 | exonuclease | chromosome:5380943-5383522 | 1448A-rhpS-KB | 1448A-WT-KB | OK | 11.4434 | 56.4328 | 2.30202 | 1.28855 | 0.2099 | 0.446697 |
| PSPPH_2878 | glycosyl hydrolase | chromosome:3324194-3326814 | 1448A-rhpS-KB | 1448A-WT-KB | NOTEST | 5.90275 | 28.8502 | 2.28912 | 0 | 1 | 1 |
| PSPPH_2962 | sarcosine oxidase subunit beta | chromosome:3435544-3438533 | 1448A-rhpS-KB | 1448A-WT-KB | OK | 11.3216 | 55.3147 | 2.28858 | 1.55133 | 0.06875 | 0.240598 |
| PSPPH_A0110 | hypothetical protein | large_plasmid:94896-95862 | 1448A-rhpS-KB | 1448A-WT-KB | OK | 38.925 | 188.584 | 2.27644 | 2.63442 | 0.0071 | 0.0539902 |
| PSPPH_3001 | dipeptide ABC transporter permease DppC | chromosome:3484226-3485066 | 1448A-rhpS-KB | 1448A-WT-KB | OK | 9.81354 | 47.541 | 2.27632 | 1.28179 | 0.18495 | 0.41339 |
| PSPPH_2448 | TetR family transcriptional regulator | chromosome:2829006-2829579 | 1448A-rhpS-KB | 1448A-WT-KB | OK | 18.7777 | 90.8607 | 2.27463 | 1.55562 | 0.10925 | 0.310628 |
| PSPPH_3472 | cytosine/purines uracil thiamine allantoin permease | chromosome:4011242-4012694 | 1448A-rhpS-KB | 1448A-WT-KB | NOTEST | 4.89397 | 23.652 | 2.27288 | 0 | 1 | 1 |
| PSPPH_2333 | sugar ABC transporter substrate-binding protein | chromosome:2707184-2708111 | 1448A-rhpS-KB | 1448A-WT-KB | NOTEST | 3.75937 | 17.9683 | 2.25689 | 0 | 1 | 1 |
| tauA | taurine ABC transporter periplasmic binding protein | chromosome:5582344-5583322 | 1448A-rhpS-KB | 1448A-WT-KB | NOTEST | 2.95309 | 14.0938 | 2.25477 | 0 | 1 | 1 |
| PSPPH_2358 | papain cysteine protease | chromosome:2734364-2735132 | 1448A-rhpS-KB | 1448A-WT-KB | NOTEST | 4.91239 | 23.2026 | 2.23979 | 0 | 1 | 1 |
| PSPPH_5086 | aldo/keto reductase | chromosome:5770197-5771205 | 1448A-rhpS-KB | 1448A-WT-KB | OK | 75.2198 | 354.448 | 2.23639 | 3.57703 | 0.00025 | 0.0043799 |
| PSPPH_4739 | nucleotidyltransferase | chromosome:5380943-5383522 | 1448A-rhpS-KB | 1448A-WT-KB | OK | 11.2463 | 52.9437 | 2.23501 | 2.15784 | 0.08665 | 0.27325 |
| PSPPH_4804 | hypothetical protein | chromosome:5451806-5452103 | 1448A-rhpS-KB | 1448A-WT-KB | OK | 25.7282 | 120.899 | 2.23238 | 1.10816 | 0.2747 | 0.510545 |
| PSPPH_0932 | hypothetical protein | chromosome:1105430-1107258 | 1448A-rhpS-KB | 1448A-WT-KB | NOTEST | 3.93132 | 18.3467 | 2.22243 | 0 | 1 | 1 |
| PSPPH_4544 | hypothetical protein | chromosome:5179498-5181795 | 1448A-rhpS-KB | 1448A-WT-KB | OK | 18.3671 | 85.6769 | 2.22178 | 1.32467 | 0.2016 | 0.435838 |
| PSPPH_3585 | hypothetical protein | chromosome:4129536-4131999 | 1448A-rhpS-KB | 1448A-WT-KB | NOTEST | 3.95347 | 18.4255 | 2.22052 | 0 | 1 | 1 |
| PSPPH_2688 | LysR family transcriptional regulator | chromosome:3112519-3113431 | 1448A-rhpS-KB | 1448A-WT-KB | OK | 11.7967 | 54.9526 | 2.21981 | 1.51683 | 0.1095 | 0.310844 |
| PSPPH_3743 | hypothetical protein | chromosome:4292502-4294374 | 1448A-rhpS-KB | 1448A-WT-KB | NOTEST | 5.71947 | 26.5002 | 2.21205 | 0 | 1 | 1 |
| PSPPH_0537 | cytosine/purines uracil thiamine allantoin permease | chromosome:632078-633129 | 1448A-rhpS-KB | 1448A-WT-KB | OK | 39.0325 | 180.739 | 2.21116 | 1.28505 | 0.25015 | 0.484835 |
| PSPPH_4553 | major facilitator family protein | chromosome:5192081-5193278 | 1448A-rhpS-KB | 1448A-WT-KB | OK | 14.0573 | 64.274 | 2.19291 | 1.6305 | 0.06 | 0.224587 |
| mets | methionyl-tRNA synthetase | chromosome:2787398-2789397 | 1448A-rhpS-KB | 1448A-WT-KB | NOTEST | 2.17091 | 9.90769 | 2.19025 | 0 | 1 | 1 |
| PSPPH_4772 | hypothetical protein | chromosome:5418027-5419086 | 1448A-rhpS-KB | 1448A-WT-KB | OK | 13.2589 | 60.112 | 2.18069 | 1.7798 | 0.04075 | 0.179139 |
| lrp | leucine-responsive regulatory protein | chromosome:260136-260625 | 1448A-rhpS-KB | 1448A-WT-KB | OK | 76.7216 | 347.8 | 2.18055 | 2.69271 | 0.0101 | 0.070092 |
| PSPPH_2881 | ABC transporter ATP-binding protein/permease | chromosome:3328006-3329767 | 1448A-rhpS-KB | 1448A-WT-KB | OK | 10.0091 | 44.9569 | 2.16722 | 1.81272 | 0.04345 | 0.186404 |
| PSPPH_0153 | agmatine deiminase | chromosome:181104-182211 | 1448A-rhpS-KB | 1448A-WT-KB | OK | 79.2401 | 355.767 | 2.16663 | 3.84972 | 0.00005 | 0.00121565 |
| PSPPH_2907 | AcrB/AcrD/AcrF family transporter | chromosome:3374729-3377873 | 1448A-rhpS-KB | 1448A-WT-KB | OK | 17.5871 | 78.808 | 2.16382 | 3.25493 | 0.00035 | 0.00566018 |
| PSPPH_4831 | cation efflux family protein | chromosome:5484609-5487765 | 1448A-rhpS-KB | 1448A-WT-KB | NOTEST | 3.21165 | 14.3775 | 2.16243 | 0 | 1 | 1 |
| PSPPH_3815 | esterase | chromosome:4366393-4367239 | 1448A-rhpS-KB | 1448A-WT-KB | OK | 88.9361 | 395.98 | 2.15458 | 3.48769 | 0.00015 | 0.00302881 |
| PSPPH_5058 | HAD family hydrolase | chromosome:5732577-5733252 | 1448A-rhpS-KB | 1448A-WT-KB | NOTEST | 5.49296 | 24.4477 | 2.15404 | 0 | 1 | 1 |
| madL | malonate transporter subunit MadL | chromosome:501552-501981 | 1448A-rhpS-KB | 1448A-WT-KB | OK | 26.1624 | 116.184 | 2.15085 | 1.30517 | 0.1902 | 0.419614 |
| PSPPH_1933 | Tat pathway signal sequence domain-containing protein | chromosome:2276823-2278497 | 1448A-rhpS-KB | 1448A-WT-KB | NOTEST | 5.42722 | 23.9918 | 2.14426 | 0 | 1 | 1 |
| PSPPH_4355 | hypothetical protein | chromosome:4978269-4979184 | 1448A-rhpS-KB | 1448A-WT-KB | OK | 41.0603 | 178.533 | 2.12037 | 2.40807 | 0.0053 | 0.0440516 |
| vdh | vanillin dehydrogenase | chromosome:2815235-2816684 | 1448A-rhpS-KB | 1448A-WT-KB | OK | 11.4336 | 49.3245 | 2.10902 | 1.85177 | 0.04655 | 0.19527 |
| PSPPH_3007 | iron ABC transporter permease | chromosome:3489422-3492411 | 1448A-rhpS-KB | 1448A-WT-KB | OK | 10.6688 | 45.9636 | 2.1071 | 0.838097 | 0.19745 | 0.429772 |
| PSPPH_2419 | alcohol dehydrogenase | chromosome:2797531-2798686 | 1448A-rhpS-KB | 1448A-WT-KB | NOTEST | 5.96199 | 25.545 | 2.09918 | 0 | 1 | 1 |
| PSPPH_0612 | hypothetical protein | chromosome:722114-724869 | 1448A-rhpS-KB | 1448A-WT-KB | OK | 21.6525 | 92.5391 | 2.09553 | 1.77514 | 0.03605 | 0.165429 |
| PSPPH_4303 | L-arginine:lysine amidinotransferase | chromosome:4914841-4915939 | 1448A-rhpS-KB | 1448A-WT-KB | OK | 67.7699 | 289.284 | 2.09377 | 3.34602 | 0.0002 | 0.00374241 |
| PSPPH_1248 | hypothetical protein | chromosome:1455904-1456321 | 1448A-rhpS-KB | 1448A-WT-KB | OK | 11.7013 | 49.8741 | 2.09162 | 0.804242 | 0.3109 | 0.547098 |
| PSPPH_3734 | siderophore biosynthesis protein | chromosome:4281478-4282123 | 1448A-rhpS-KB | 1448A-WT-KB | OK | 11.54 | 49.0064 | 2.08633 | 1.09848 | 0.20315 | 0.438235 |
| lolB | molecular chaperone LolB | chromosome:1187121-1187739 | 1448A-rhpS-KB | 1448A-WT-KB | OK | 45.81 | 194.487 | 2.08594 | 2.05639 | 0.01965 | 0.108882 |
| PSPPH_2704 | ornithine decarboxylase | chromosome:3130811-3131927 | 1448A-rhpS-KB | 1448A-WT-KB | NOTEST | 2.94719 | 12.4771 | 2.08187 | 0 | 1 | 1 |
| pcaF | beta-ketoadipyl CoA thiolase | chromosome:4591943-4594779 | 1448A-rhpS-KB | 1448A-WT-KB | OK | 27.9512 | 118.169 | 2.07988 | 2.04868 | 0.01925 | 0.108005 |
| PSPPH_0516 | hypothetical protein | chromosome:597387-603334 | 1448A-rhpS-KB | 1448A-WT-KB | OK | 44.2443 | 186.941 | 2.07902 | 1.54093 | 0.12645 | 0.336893 |
| PSPPH_4723 | molybdate transport regulator ModE | chromosome:5360301-5360826 | 1448A-rhpS-KB | 1448A-WT-KB | NOTEST | 9.41476 | 39.6787 | 2.07537 | 0 | 1 | 1 |
| PSPPH_1737 | LysR family transcriptional regulator | chromosome:2016764-2017673 | 1448A-rhpS-KB | 1448A-WT-KB | OK | 13.4009 | 56.2965 | 2.07072 | 1.53075 | 0.0753 | 0.256551 |
| cadR | Cd(II)/Pb(II)-responsive transcriptional regulator | chromosome:5539051-5539498 | 1448A-rhpS-KB | 1448A-WT-KB | OK | 17.2501 | 72.2926 | 2.06724 | 1.10407 | 0.20475 | 0.440299 |
| PSPPH_2460 | hypothetical protein | chromosome:2841956-2842727 | 1448A-rhpS-KB | 1448A-WT-KB | NOTEST | 4.93529 | 20.6767 | 2.0668 | 0 | 1 | 1 |
| PSPPH_4315 | hypothetical protein | chromosome:4927731-4928802 | 1448A-rhpS-KB | 1448A-WT-KB | OK | 71.775 | 300.559 | 2.0661 | 3.22582 | 0.00015 | 0.00302881 |
| PSPPH_1742 | hypothetical protein | chromosome:2020877-2022595 | 1448A-rhpS-KB | 1448A-WT-KB | OK | 13.2692 | 55.4047 | 2.06193 | 0.809619 | 0.3681 | 0.601275 |
| PSPPH_0178 | Mg-chelatase subunits D/I family, ComM subfamily protein | chromosome:211855-212719 | 1448A-rhpS-KB | 1448A-WT-KB | NOTEST | 8.99515 | 37.553 | 2.06171 | 0 | 1 | 1 |
| bchI | magnesium chelatase ATPase subunit I | chromosome:2582359-2588302 | 1448A-rhpS-KB | 1448A-WT-KB | OK | 23.5727 | 98.3768 | 2.0612 | 1.14103 | 0.11665 | 0.322684 |
| PSPPH_4057 | major facilitator family transporter | chromosome:4638253-4639420 | 1448A-rhpS-KB | 1448A-WT-KB | OK | 18.7723 | 78.3045 | 2.06049 | 1.88171 | 0.02975 | 0.144465 |
| PSPPH_2926 | hypothetical protein | chromosome:3397386-3397836 | 1448A-rhpS-KB | 1448A-WT-KB | OK | 11.7047 | 48.7989 | 2.05976 | 0.826947 | 0.28705 | 0.522725 |
| PSPPH_3546 | hypothetical protein | chromosome:4083055-4083469 | 1448A-rhpS-KB | 1448A-WT-KB | OK | 23.0243 | 95.6615 | 2.05478 | 1.21235 | 0.20405 | 0.439587 |
| PSPPH_0313 | NLPA family lipoprotein | chromosome:355075-355858 | 1448A-rhpS-KB | 1448A-WT-KB | OK | 90.4335 | 375.384 | 2.05344 | 3.12708 | 0.0005 | 0.00757203 |
| PSPPH_2071 | nitrite reductase (NAD(P)H), truncated | chromosome:2425139-2429086 | 1448A-rhpS-KB | 1448A-WT-KB | NOTEST | 4.82177 | 19.9984 | 2.05225 | 0 | 1 | 1 |
| PSPPH_5166 | SPFH domain-containing protein | chromosome:5858054-5860001 | 1448A-rhpS-KB | 1448A-WT-KB | OK | 14.5075 | 60.1692 | 2.05223 | 2.16862 | 0.0116 | 0.0774923 |
| PSPPH_1076 | short chain dehydrogenase/reductase oxidoreductase | chromosome:1265374-1266286 | 1448A-rhpS-KB | 1448A-WT-KB | OK | 16.6319 | 68.5705 | 2.04363 | 1.5046 | 0.0679 | 0.23956 |
| PSPPH_2362 | ribose ABC transporter substrate-binding protein | chromosome:2738791-2739796 | 1448A-rhpS-KB | 1448A-WT-KB | NOTEST | 3.18183 | 13.1132 | 2.04309 | 0 | 1 | 1 |
| ugpC | glycerol-3-phosphate ABC transporter ATP-binding protein | chromosome:5164736-5168936 | 1448A-rhpS-KB | 1448A-WT-KB | NOTEST | 2.67189 | 11.0056 | 2.04231 | 0 | 1 | 1 |
| PSPPH_0751 | hypothetical protein | chromosome:876090-877905 | 1448A-rhpS-KB | 1448A-WT-KB | OK | 22.0275 | 90.5724 | 2.03976 | 2.57286 | 0.005 | 0.0425476 |
| PSPPH_4554 | arginine aminomutase | chromosome:5193346-5194504 | 1448A-rhpS-KB | 1448A-WT-KB | OK | 27.9587 | 114.606 | 2.03531 | 2.14015 | 0.0117 | 0.0778693 |
| PSPPH_4524 | TonB-dependent receptor | chromosome:5156437-5158744 | 1448A-rhpS-KB | 1448A-WT-KB | NOTEST | 4.59794 | 18.8417 | 2.03487 | 0 | 1 | 1 |
| PSPPH_5185 | iron ABC transporter substrate-binding protein | chromosome:5885135-5887132 | 1448A-rhpS-KB | 1448A-WT-KB | NOTEST | 6.8692 | 27.9494 | 2.0246 | 0 | 1 | 1 |
| fecD | iron ABC transporter permease | chromosome:5268669-5270609 | 1448A-rhpS-KB | 1448A-WT-KB | NOTEST | 3.81628 | 15.4774 | 2.01992 | 0 | 1 | 1 |
| gspD2 | general secretion pathway protein GspD | chromosome:2532397-2537908 | 1448A-rhpS-KB | 1448A-WT-KB | NOTEST | 2.67073 | 10.8226 | 2.01874 | 0 | 1 | 1 |
| PSPPH_5056 | hypothetical protein | chromosome:5730470-5731201 | 1448A-rhpS-KB | 1448A-WT-KB | OK | 11.2724 | 45.6111 | 2.01659 | 0.771381 | 0.30185 | 0.535922 |
| PSPPH_0295 | hypothetical protein | chromosome:338359-339097 | 1448A-rhpS-KB | 1448A-WT-KB | NOTEST | 9.87448 | 39.9167 | 2.01522 | 0 | 1 | 1 |
| PSPPH_0407 | gluconate transporter family protein | chromosome:470481-471831 | 1448A-rhpS-KB | 1448A-WT-KB | NOTEST | 2.74914 | 11.0278 | 2.00409 | 0 | 1 | 1 |
| PSPPH_2206 | hypothetical protein | chromosome:2562318-2562657 | 1448A-rhpS-KB | 1448A-WT-KB | OK | 18.9352 | 75.5609 | 1.99657 | 0.875663 | 0.342 | 0.578137 |
| PSPPH_4725 | Ser/Thr protein phosphatase | chromosome:5363095-5363929 | 1448A-rhpS-KB | 1448A-WT-KB | NOTEST | 8.62994 | 34.4366 | 1.99652 | 0 | 1 | 1 |
| PSPPH_1523 | serine protease | chromosome:1772228-1773839 | 1448A-rhpS-KB | 1448A-WT-KB | NOTEST | 9.81718 | 39.116 | 1.99438 | 0 | 1 | 1 |
| PSPPH_4463 | cobalt-precorrin-6x reductase | chromosome:5095634-5098665 | 1448A-rhpS-KB | 1448A-WT-KB | OK | 20.0088 | 79.3843 | 1.98822 | 1.19504 | 0.2284 | 0.464864 |
| PSPPH_4261 | OprD family outer membrane porin | chromosome:4864067-4865483 | 1448A-rhpS-KB | 1448A-WT-KB | OK | 162.929 | 641.15 | 1.97641 | 5.05528 | 0.00005 | 0.00121565 |
| PSPPH_1782 | mechanosensitive ion channel protein MscS | chromosome:2087248-2088235 | 1448A-rhpS-KB | 1448A-WT-KB | NOTEST | 8.33571 | 32.7426 | 1.97379 | 0 | 1 | 1 |
| PSPPH_2761 | hypothetical protein | chromosome:3196863-3199066 | 1448A-rhpS-KB | 1448A-WT-KB | OK | 22.8263 | 89.2251 | 1.96675 | 1.27571 | 0.2094 | 0.44627 |
| PSPPH_3425 | gluconate 5-dehydrogenase | chromosome:3959189-3960589 | 1448A-rhpS-KB | 1448A-WT-KB | NOTEST | 10.4288 | 40.7057 | 1.96465 | 0 | 1 | 1 |
| PSPPH_0405 | N-acyl-D-amino-acid deacylase | chromosome:468141-469617 | 1448A-rhpS-KB | 1448A-WT-KB | OK | 18.0606 | 70.48 | 1.96437 | 2.06866 | 0.01845 | 0.105336 |
| PSPPH_1922 | pyoverdine biosynthesis regulatory protein | chromosome:2238231-2239209 | 1448A-rhpS-KB | 1448A-WT-KB | NOTEST | 2.99044 | 11.619 | 1.95806 | 0 | 1 | 1 |
| cobU | adenosylcobinamide kinase | chromosome:4241334-4243474 | 1448A-rhpS-KB | 1448A-WT-KB | OK | 19.3001 | 74.8122 | 1.95467 | 1.0533 | 0.2547 | 0.489099 |
| PSPPH_0124 | hypothetical protein | chromosome:140679-144092 | 1448A-rhpS-KB | 1448A-WT-KB | OK | 33.2285 | 128.687 | 1.95338 | 1.53325 | 0.07825 | 0.259762 |
| PSPPH_0771 | diguanylate phosphodiesterase | chromosome:904055-904868 | 1448A-rhpS-KB | 1448A-WT-KB | NOTEST | 10.5865 | 40.9215 | 1.95063 | 0 | 1 | 1 |
| PSPPH_3163 | TonB-dependent siderophore receptor | chromosome:3666250-3668437 | 1448A-rhpS-KB | 1448A-WT-KB | NOTEST | 5.50654 | 21.2406 | 1.94761 | 0 | 1 | 1 |
| PSPPH_1083 | UDP-N-acetylglucosamine 2-epimerase | chromosome:1275536-1276793 | 1448A-rhpS-KB | 1448A-WT-KB | NOTEST | 9.97304 | 38.2792 | 1.94046 | 0 | 1 | 1 |
| PSPPH_1595 | GntR family transcriptional regulator | chromosome:1849522-1850245 | 1448A-rhpS-KB | 1448A-WT-KB | NOTEST | 4.79566 | 18.3401 | 1.9352 | 0 | 1 | 1 |
| mdcC | malonate decarboxylase subunit delta | chromosome:497978-501483 | 1448A-rhpS-KB | 1448A-WT-KB | OK | 125.302 | 477.377 | 1.92971 | 1.03916 | 0.2867 | 0.522725 |
| PSPPH_2282 | Rhs family protein | chromosome:2647752-2649491 | 1448A-rhpS-KB | 1448A-WT-KB | OK | 14.2448 | 54.2591 | 1.92943 | 1.39983 | 0.08315 | 0.266767 |
| PSPPH_4304 | HAD superfamily hydrolase | chromosome:4916042-4920156 | 1448A-rhpS-KB | 1448A-WT-KB | OK | 43.821 | 166.571 | 1.92645 | 1.67719 | 0.0485 | 0.200392 |
| PSPPH_0348 | hypothetical protein | chromosome:396061-397404 | 1448A-rhpS-KB | 1448A-WT-KB | OK | 55.0054 | 208.304 | 1.92104 | 1.03216 | 0.2153 | 0.451784 |
| PSPPH_0885 | hypothetical protein | chromosome:1055367-1060158 | 1448A-rhpS-KB | 1448A-WT-KB | OK | 46.7265 | 176.131 | 1.91433 | 1.26235 | 0.11265 | 0.315914 |
| PSPPH_3574 | sugar transporter | chromosome:4117842-4118835 | 1448A-rhpS-KB | 1448A-WT-KB | OK | 32.5916 | 122.78 | 1.91351 | 2.1403 | 0.0089 | 0.0645205 |
| livM | leucine/isoleucine/valine transporter permease subunit | chromosome:1638998-1641948 | 1448A-rhpS-KB | 1448A-WT-KB | OK | 48.2828 | 181.821 | 1.91294 | 2.32926 | 0.0075 | 0.056195 |
| hrpP | type III secretion protein HrpP | chromosome:1497756-1499418 | 1448A-rhpS-KB | 1448A-WT-KB | NOTEST | 6.23962 | 23.3996 | 1.90696 | 0 | 1 | 1 |
| PSPPH_4624 | ABC transporter ATP-binding protein | chromosome:5260293-5261847 | 1448A-rhpS-KB | 1448A-WT-KB | NOTEST | 3.71887 | 13.9454 | 1.90685 | 0 | 1 | 1 |
| PSPPH_0221 | hypothetical protein | chromosome:259723-260131 | 1448A-rhpS-KB | 1448A-WT-KB | OK | 40.9947 | 153.193 | 1.90184 | 1.27826 | 0.10775 | 0.307833 |
| PSPPH_2172 | hypothetical protein | chromosome:2526894-2527539 | 1448A-rhpS-KB | 1448A-WT-KB | NOTEST | 5.60306 | 20.9339 | 1.90156 | 0 | 1 | 1 |
| aspB | aspartate aminotransferase | chromosome:2835549-2836929 | 1448A-rhpS-KB | 1448A-WT-KB | NOTEST | 1.94897 | 7.28074 | 1.90137 | 0 | 1 | 1 |
| PSPPH_4346 | hypothetical protein | chromosome:4968604-4969849 | 1448A-rhpS-KB | 1448A-WT-KB | OK | 18.5526 | 69.2156 | 1.89948 | 1.76264 | 0.04015 | 0.177156 |
| PSPPH_4527 | hypothetical protein | chromosome:5162326-5163148 | 1448A-rhpS-KB | 1448A-WT-KB | NOTEST | 10.4407 | 38.898 | 1.89748 | 0 | 1 | 1 |
| PSPPH_0322 | ABC transporter permease | chromosome:366774-367641 | 1448A-rhpS-KB | 1448A-WT-KB | NOTEST | 4.23901 | 15.7525 | 1.89378 | 0 | 1 | 1 |
| PSPPH_3122 | transcriptional regulator | chromosome:3623016-3624006 | 1448A-rhpS-KB | 1448A-WT-KB | NOTEST | 12.1074 | 44.7501 | 1.886 | 0 | 1 | 1 |
| PSPPH_4728 | ABC transporter permease | chromosome:5363953-5368805 | 1448A-rhpS-KB | 1448A-WT-KB | OK | 14.8719 | 54.7801 | 1.88107 | 1.24054 | 0.2446 | 0.477803 |
| PSPPH_2538 | type III secretion component | chromosome:2920152-2922014 | 1448A-rhpS-KB | 1448A-WT-KB | NOTEST | 3.89155 | 14.2814 | 1.87572 | 0 | 1 | 1 |
| PSPPH_2780 | hypothetical protein | chromosome:3217331-3218567 | 1448A-rhpS-KB | 1448A-WT-KB | NOTEST | 8.34372 | 30.6011 | 1.87482 | 0 | 1 | 1 |
| PSPPH_0791 | lipoprotein | chromosome:935832-936333 | 1448A-rhpS-KB | 1448A-WT-KB | OK | 18.7674 | 68.5989 | 1.86996 | 1.08324 | 0.23685 | 0.470315 |
| PSPPH_0102 | ParB-like nuclease | chromosome:111137-114579 | 1448A-rhpS-KB | 1448A-WT-KB | OK | 12.5517 | 45.873 | 1.86976 | 1.0298 | 0.16685 | 0.394132 |
| PSPPH_1959 | major facilitator superfamily transporter phthalate permease | chromosome:2306449-2307760 | 1448A-rhpS-KB | 1448A-WT-KB | OK | 14.435 | 52.7377 | 1.86926 | 1.71492 | 0.06285 | 0.229914 |
| glnQ1 | amino-acid ABC transporter ATP-binding protein YecC | chromosome:386926-388972 | 1448A-rhpS-KB | 1448A-WT-KB | OK | 54.4274 | 198.41 | 1.86608 | 2.49714 | 0.00455 | 0.0397597 |
| PSPPH_1416 | high-affinity branched-chain amino acid ABC transporter permease | chromosome:1638998-1641948 | 1448A-rhpS-KB | 1448A-WT-KB | OK | 38.4155 | 139.735 | 1.86294 | 1.37259 | 0.21275 | 0.449276 |
| PSPPH_0438 | phosphoribosyl-dephospho-CoA transferase | chromosome:497978-501483 | 1448A-rhpS-KB | 1448A-WT-KB | OK | 135.358 | 490.972 | 1.85886 | 2.32939 | 0.00335 | 0.0320989 |
| PSPPH_1800 | MarR family transcriptional regulator | chromosome:2105810-2106281 | 1448A-rhpS-KB | 1448A-WT-KB | OK | 40.2964 | 146.133 | 1.85856 | 1.35697 | 0.0895 | 0.278635 |
| PSPPH_1738 | glyoxalase | chromosome:2017743-2018798 | 1448A-rhpS-KB | 1448A-WT-KB | NOTEST | 8.98861 | 32.5807 | 1.85785 | 0 | 1 | 1 |
| PSPPH_2436 | thiolase | chromosome:2816953-2819964 | 1448A-rhpS-KB | 1448A-WT-KB | NOTEST | 8.71123 | 31.5633 | 1.8573 | 0 | 1 | 1 |
| PSPPH_2925 | lytic transglycosylase | chromosome:3396245-3397354 | 1448A-rhpS-KB | 1448A-WT-KB | OK | 22.1081 | 79.7613 | 1.85111 | 0.905145 | 0.2434 | 0.476925 |
| PSPPH_0294 | hypothetical protein | chromosome:336964-338262 | 1448A-rhpS-KB | 1448A-WT-KB | NOTEST | 5.48065 | 19.7031 | 1.846 | 0 | 1 | 1 |
| PSPPH_2695 | peptide ABC transporter permease | chromosome:3121352-3124701 | 1448A-rhpS-KB | 1448A-WT-KB | NOTEST | 4.19376 | 15.0619 | 1.84458 | 0 | 1 | 1 |
| PSPPH_3988 | hypothetical protein | chromosome:4553233-4554130 | 1448A-rhpS-KB | 1448A-WT-KB | OK | 23.5824 | 84.5922 | 1.84281 | 1.77402 | 0.0983 | 0.291072 |
| PSPPH_4955 | hypothetical protein | chromosome:5628151-5632456 | 1448A-rhpS-KB | 1448A-WT-KB | NOTEST | 2.89837 | 10.3926 | 1.84224 | 0 | 1 | 1 |
| PSPPH_2379 | multidrug efflux transporter | chromosome:2758098-2762654 | 1448A-rhpS-KB | 1448A-WT-KB | NOTEST | 2.15013 | 7.69059 | 1.83867 | 0 | 1 | 1 |
| PSPPH_0466 | hypothetical protein | chromosome:529837-530428 | 1448A-rhpS-KB | 1448A-WT-KB | OK | 329.647 | 1175.61 | 1.83441 | 3.98204 | 0.00005 | 0.00121565 |
| cbiD | cobalt-precorrin-6A synthase | chromosome:5095634-5098665 | 1448A-rhpS-KB | 1448A-WT-KB | OK | 12.7846 | 45.5843 | 1.83413 | 1.01794 | 0.24765 | 0.481295 |
| PSPPH_5143 | pyruvate carboxylase subunit A | chromosome:5833105-5834521 | 1448A-rhpS-KB | 1448A-WT-KB | OK | 447.092 | 1591.95 | 1.83215 | 6.18137 | 0.00005 | 0.00121565 |
| fliR | flagellar biosynthesis protein FliR | chromosome:3897749-3898526 | 1448A-rhpS-KB | 1448A-WT-KB | NOTEST | 5.85292 | 20.818 | 1.83061 | 0 | 1 | 1 |
| PSPPH_0777 | response regulator/sensor histidine kinase | chromosome:911076-913832 | 1448A-rhpS-KB | 1448A-WT-KB | OK | 15.3263 | 54.4524 | 1.82899 | 1.74325 | 0.0285 | 0.139916 |
| glcB | malate synthase G | chromosome:5371993-5374171 | 1448A-rhpS-KB | 1448A-WT-KB | OK | 67.5871 | 239.845 | 1.82728 | 3.97883 | 0.00005 | 0.00121565 |
| epd | D-erythrose 4-phosphate dehydrogenase | chromosome:5470952-5471996 | 1448A-rhpS-KB | 1448A-WT-KB | OK | 43.6162 | 154.416 | 1.82389 | 2.33808 | 0.00715 | 0.054255 |
| PSPPH_4235 | peptide ABC transporter ATP-binding protein | chromosome:4830473-4834247 | 1448A-rhpS-KB | 1448A-WT-KB | NOTEST | 11.4158 | 40.102 | 1.81264 | 0 | 1 | 1 |
| PSPPH_0269 | D-methionine ABC transporter permease | chromosome:310589-312271 | 1448A-rhpS-KB | 1448A-WT-KB | OK | 43.9847 | 154.333 | 1.81097 | 1.52301 | 0.05605 | 0.216058 |
| PSPPH_5065 | hypothetical protein | chromosome:5741855-5742410 | 1448A-rhpS-KB | 1448A-WT-KB | OK | 28.6722 | 100.335 | 1.80711 | 1.44374 | 0.1548 | 0.37662 |
| lhr | helicase | chromosome:1603524-1606670 | 1448A-rhpS-KB | 1448A-WT-KB | NOTEST | 8.96256 | 31.3427 | 1.80615 | 0 | 1 | 1 |
| PSPPH_4686 | ATP/GTP-binding protein | chromosome:5320625-5322029 | 1448A-rhpS-KB | 1448A-WT-KB | OK | 13.7773 | 48.006 | 1.80093 | 1.48985 | 0.07075 | 0.245496 |
| PSPPH_4302 | hypothetical protein | chromosome:4913516-4914782 | 1448A-rhpS-KB | 1448A-WT-KB | OK | 56.6186 | 196.581 | 1.79578 | 2.65333 | 0.00165 | 0.0195917 |
| PSPPH_2791 | hypothetical protein | chromosome:3230333-3231464 | 1448A-rhpS-KB | 1448A-WT-KB | OK | 36.1041 | 125.343 | 1.79565 | 2.03319 | 0.0126 | 0.0824769 |
| PSPPH_3749 | MupB | chromosome:4301192-4302047 | 1448A-rhpS-KB | 1448A-WT-KB | OK | 14.1684 | 49.0761 | 1.79234 | 1.2053 | 0.1238 | 0.334455 |
| cueR | Cu(I)-responsive transcriptional regulator | chromosome:5280482-5283076 | 1448A-rhpS-KB | 1448A-WT-KB | OK | 81.0054 | 280.409 | 1.79144 | 1.08516 | 0.24305 | 0.476501 |
| PSPPH_2485 | HypX | chromosome:2869905-2870598 | 1448A-rhpS-KB | 1448A-WT-KB | OK | 20.3237 | 70.1745 | 1.78778 | 1.34267 | 0.15945 | 0.383754 |
| PSPPH_1864 | hypothetical protein | chromosome:2165379-2165988 | 1448A-rhpS-KB | 1448A-WT-KB | NOTEST | 6.0696 | 20.9473 | 1.78709 | 0 | 1 | 1 |
| PSPPH_5176 | methyl-accepting chemotaxis protein | chromosome:5873460-5875491 | 1448A-rhpS-KB | 1448A-WT-KB | NOTEST | 6.41216 | 22.0881 | 1.78439 | 0 | 1 | 1 |
| oprI | outer membrane lipoprotein OprI | chromosome:2395627-2395879 | 1448A-rhpS-KB | 1448A-WT-KB | OK | 10406.7 | 35800.2 | 1.78245 | 6.39939 | 0.00005 | 0.00121565 |
| PSPPH_4241 | LuxR family transcriptional regulator | chromosome:4838478-4839108 | 1448A-rhpS-KB | 1448A-WT-KB | OK | 63.392 | 217.572 | 1.77912 | 2.2347 | 0.0192 | 0.107894 |
| minE | cell division topological specificity factor MinE | chromosome:1865806-1866873 | 1448A-rhpS-KB | 1448A-WT-KB | OK | 294.785 | 1010.58 | 1.77745 | 1.02144 | 0.17795 | 0.407427 |
| PSPPH_2457 | hypothetical protein | chromosome:2838562-2841952 | 1448A-rhpS-KB | 1448A-WT-KB | NOTEST | 5.51684 | 18.8497 | 1.77262 | 0 | 1 | 1 |
| PSPPH_3155 | LysR family transcriptional regulator | chromosome:3658548-3659439 | 1448A-rhpS-KB | 1448A-WT-KB | NOTEST | 4.54681 | 15.5038 | 1.76969 | 0 | 1 | 1 |
| PSPPH_2473 | transcriptional regulator VanR | chromosome:2856411-2857080 | 1448A-rhpS-KB | 1448A-WT-KB | OK | 20.4815 | 69.8102 | 1.76912 | 1.15548 | 0.1233 | 0.333844 |
| PSPPH_3258 | gluconokinase | chromosome:3776539-3777067 | 1448A-rhpS-KB | 1448A-WT-KB | OK | 110.982 | 377.745 | 1.76709 | 2.30364 | 0.00735 | 0.0554196 |
| macB | macrolide efflux ABC transporter ATP-binding/permease | chromosome:2278900-2282267 | 1448A-rhpS-KB | 1448A-WT-KB | NOTEST | 1.51692 | 5.1504 | 1.76354 | 0 | 1 | 1 |
| PSPPH_1006 | hypothetical protein | chromosome:1195847-1202294 | 1448A-rhpS-KB | 1448A-WT-KB | OK | 28.2304 | 95.7737 | 1.76238 | 0.929805 | 0.2711 | 0.507018 |
| fecE | iron-dicitrate transporter ATP-binding subunit | chromosome:5270613-5271405 | 1448A-rhpS-KB | 1448A-WT-KB | NOTEST | 4.15682 | 14.0658 | 1.75864 | 0 | 1 | 1 |
| ccmF | cytochrome c-type biogenesis protein CcmF | chromosome:3831853-3833827 | 1448A-rhpS-KB | 1448A-WT-KB | NOTEST | 9.47296 | 32.042 | 1.75808 | 0 | 1 | 1 |
| PSPPH_0033 | 3-oxoadipate enol-lactonase | chromosome:38876-39701 | 1448A-rhpS-KB | 1448A-WT-KB | OK | 155.332 | 524.91 | 1.75672 | 3.33687 | 0.0001 | 0.00221988 |
| PSPPH_4236 | peptide ABC transporter permease | chromosome:4830473-4834247 | 1448A-rhpS-KB | 1448A-WT-KB | NOTEST | 4.98681 | 16.7995 | 1.75223 | 0 | 1 | 1 |
| PSPPH_2678 | hypothetical protein | chromosome:3098420-3104173 | 1448A-rhpS-KB | 1448A-WT-KB | NOTEST | 13.0312 | 43.8899 | 1.75192 | 0 | 1 | 1 |
| trkA | potassium transporter peripheral membrane protein | chromosome:22798-26456 | 1448A-rhpS-KB | 1448A-WT-KB | OK | 71.9549 | 242.068 | 1.75024 | 2.11885 | 0.0103 | 0.0712035 |
| PSPPH_4808 | electron transfer flavoprotein subunit beta | chromosome:5458598-5460378 | 1448A-rhpS-KB | 1448A-WT-KB | NOTEST | 9.46131 | 31.7931 | 1.7486 | 0 | 1 | 1 |
| PSPPH_4827 | heavy metal sensor histidine kinase | chromosome:5479708-5481771 | 1448A-rhpS-KB | 1448A-WT-KB | NOTEST | 12.5705 | 42.2353 | 1.7484 | 0 | 1 | 1 |
| PSPPH_4525 | hypothetical protein | chromosome:5158973-5159213 | 1448A-rhpS-KB | 1448A-WT-KB | OK | 38.922 | 130.381 | 1.74408 | 0.906778 | 0.3602 | 0.594704 |
| PSPPH_4305 | hypothetical protein | chromosome:4916042-4920156 | 1448A-rhpS-KB | 1448A-WT-KB | OK | 35.4372 | 118.706 | 1.74405 | 1.47975 | 0.0481 | 0.199431 |
| PSPPH_3419 | glutamine synthetase | chromosome:3951039-3952383 | 1448A-rhpS-KB | 1448A-WT-KB | OK | 31.3054 | 104.641 | 1.74096 | 2.16454 | 0.009 | 0.0645904 |
| PSPPH_3030 | lipoprotein | chromosome:3518893-3519664 | 1448A-rhpS-KB | 1448A-WT-KB | NOTEST | 4.29737 | 14.3512 | 1.73964 | 0 | 1 | 1 |
| PSPPH_3741 | phage integrase | chromosome:4290445-4291432 | 1448A-rhpS-KB | 1448A-WT-KB | NOTEST | 6.73037 | 22.3418 | 1.73099 | 0 | 1 | 1 |
| PSPPH_3554 | multidrug resistance protein | chromosome:4090154-4091363 | 1448A-rhpS-KB | 1448A-WT-KB | OK | 70.9666 | 234.819 | 1.72634 | 2.9116 | 0.0007 | 0.00962231 |
| clpB1 | ATP-dependent Clp protease ATP-binding subunit | chromosome:150905-156854 | 1448A-rhpS-KB | 1448A-WT-KB | OK | 63.5312 | 209.634 | 1.72233 | 2.56129 | 0.0021 | 0.0233087 |
| PSPPH_4737 | response regulator | chromosome:5380198-5380591 | 1448A-rhpS-KB | 1448A-WT-KB | OK | 42.617 | 140.502 | 1.72109 | 1.36638 | 0.1661 | 0.392998 |
| PSPPH_0770 | response regulator/sensor histidine kinase | chromosome:901687-903976 | 1448A-rhpS-KB | 1448A-WT-KB | OK | 18.9998 | 62.3514 | 1.71444 | 2.14694 | 0.00905 | 0.0647876 |
| PSPPH_1012 | hypothetical protein | chromosome:1203209-1204166 | 1448A-rhpS-KB | 1448A-WT-KB | OK | 53.2868 | 174.049 | 1.70765 | 2.30166 | 0.007 | 0.0535717 |
| fadB | multifunctional fatty acid oxidation complex subunit alpha | chromosome:3720585-3722751 | 1448A-rhpS-KB | 1448A-WT-KB | OK | 504.678 | 1648.43 | 1.70765 | 6.18654 | 0.00005 | 0.00121565 |
| hrpS | type III transcriptional regulator HrpS | chromosome:1486608-1487517 | 1448A-rhpS-KB | 1448A-WT-KB | OK | 16.1551 | 52.6882 | 1.70549 | 1.34083 | 0.12085 | 0.329457 |
| fadA | 3-ketoacyl-CoA thiolase | chromosome:3719405-3720581 | 1448A-rhpS-KB | 1448A-WT-KB | OK | 400.813 | 1306.61 | 1.70483 | 5.22687 | 0.00005 | 0.00121565 |
| PSPPH_5186 | hemin ABC transporter permease | chromosome:5885135-5887132 | 1448A-rhpS-KB | 1448A-WT-KB | OK | 34.1674 | 111.37 | 1.70467 | 1.83899 | 0.0206 | 0.112748 |
| PSPPH_0603 | hypothetical protein | chromosome:713112-714129 | 1448A-rhpS-KB | 1448A-WT-KB | OK | 16.4599 | 53.616 | 1.70371 | 1.4519 | 0.08085 | 0.263888 |
| PSPPH_0029 | glycine betaine family ABC transporter substrate-binding protein | chromosome:34759-35686 | 1448A-rhpS-KB | 1448A-WT-KB | NOTEST | 12.273 | 39.9543 | 1.70287 | 0 | 1 | 1 |
| rbsK | ribokinase | chromosome:2486672-2487994 | 1448A-rhpS-KB | 1448A-WT-KB | OK | 45.7691 | 148.512 | 1.69813 | 1.73004 | 0.03485 | 0.162412 |
| glnT | glutamine synthetase | chromosome:3387898-3389233 | 1448A-rhpS-KB | 1448A-WT-KB | NOTEST | 2.49511 | 8.09451 | 1.69784 | 0 | 1 | 1 |
| PSPPH_4359 | hypothetical protein | chromosome:4981203-4981908 | 1448A-rhpS-KB | 1448A-WT-KB | OK | 336.386 | 1087.62 | 1.69299 | 3.97547 | 0.00005 | 0.00121565 |
| PSPPH_0540 | hypothetical protein | chromosome:633306-634397 | 1448A-rhpS-KB | 1448A-WT-KB | OK | 135.804 | 438.794 | 1.69202 | 1.42383 | 0.078 | 0.259731 |
| PSPPH_3332 | outer membrane autotransporter | chromosome:3857988-3860283 | 1448A-rhpS-KB | 1448A-WT-KB | NOTEST | 7.72985 | 24.8909 | 1.6871 | 0 | 1 | 1 |
| PSPPH_1926 | pyoverdine sidechain peptide synthetase IV, D-Asp-L-Ser component | chromosome:2257443-2266071 | 1448A-rhpS-KB | 1448A-WT-KB | NOTEST | 3.77853 | 12.1574 | 1.68594 | 0 | 1 | 1 |
| PSPPH_5145 | RpiR family transcriptional regulator | chromosome:5835913-5836780 | 1448A-rhpS-KB | 1448A-WT-KB | OK | 40.2648 | 129.456 | 1.68487 | 1.86545 | 0.01915 | 0.107783 |
| PSPPH_3757 | hydrolase | chromosome:4309546-4310134 | 1448A-rhpS-KB | 1448A-WT-KB | OK | 24.4877 | 78.6495 | 1.68338 | 1.09632 | 0.1348 | 0.349495 |
| PSPPH_0324 | hypothetical protein | chromosome:369194-369410 | 1448A-rhpS-KB | 1448A-WT-KB | OK | 50.6537 | 162.454 | 1.68129 | 0.898122 | 0.3652 | 0.599002 |
| PSPPH_5023 | hypothetical protein | chromosome:5690780-5692085 | 1448A-rhpS-KB | 1448A-WT-KB | OK | 23.9455 | 76.6304 | 1.67816 | 1.91043 | 0.0345 | 0.161391 |
| PSPPH_2083 | sensor histidine kinase/response regulator | chromosome:2445531-2446098 | 1448A-rhpS-KB | 1448A-WT-KB | NOTEST | 6.66976 | 21.2934 | 1.6747 | 0 | 1 | 1 |
| gltD | glutamate synthase subunit beta | chromosome:460200-461619 | 1448A-rhpS-KB | 1448A-WT-KB | OK | 94.6755 | 302.251 | 1.67468 | 3.38626 | 0.00005 | 0.00121565 |
| PSPPH_4798 | TldD/PmbA family protein | chromosome:5442280-5446423 | 1448A-rhpS-KB | 1448A-WT-KB | OK | 36.98 | 117.854 | 1.67218 | 1.98229 | 0.01665 | 0.0983588 |
| PSPPH_2277 | amino acid ABC transporter permease | chromosome:2643158-2643836 | 1448A-rhpS-KB | 1448A-WT-KB | NOTEST | 12.7316 | 40.5686 | 1.67195 | 0 | 1 | 1 |
| PSPPH_2974 | D-isomer specific 2-hydroxyacid dehydrogenase | chromosome:3452019-3454322 | 1448A-rhpS-KB | 1448A-WT-KB | NOTEST | 10.4833 | 33.3968 | 1.67162 | 0 | 1 | 1 |
| PSPPH_4639 | zinc-binding dehydrogenase oxidoreductase | chromosome:5277286-5278300 | 1448A-rhpS-KB | 1448A-WT-KB | OK | 15.3442 | 48.6574 | 1.66497 | 1.19566 | 0.11085 | 0.31269 |
| PSPPH_0015 | hypothetical protein | chromosome:18980-19598 | 1448A-rhpS-KB | 1448A-WT-KB | OK | 21.28 | 67.2778 | 1.66063 | 1.10313 | 0.138 | 0.352802 |
| PSPPH_5078 | hypothetical protein | chromosome:5760403-5760955 | 1448A-rhpS-KB | 1448A-WT-KB | NOTEST | 7.83439 | 24.7466 | 1.65933 | 0 | 1 | 1 |
| PSPPH_4219 | DNA polymerase I | chromosome:4806711-4810060 | 1448A-rhpS-KB | 1448A-WT-KB | OK | 14.5276 | 45.8212 | 1.65722 | 1.36778 | 0.0745 | 0.254481 |
| PSPPH_1435 | LysR family transcriptional regulator | chromosome:1674184-1675108 | 1448A-rhpS-KB | 1448A-WT-KB | OK | 44.3133 | 139.745 | 1.65698 | 1.97132 | 0.01795 | 0.10405 |
| PSPPH_1182 | glucose ABC transporter permease | chromosome:1388742-1390489 | 1448A-rhpS-KB | 1448A-WT-KB | OK | 29.1535 | 91.8173 | 1.6551 | 1.44585 | 0.08135 | 0.264398 |
| mdcH | malonate decarboxylase subunit epsilon | chromosome:497978-501483 | 1448A-rhpS-KB | 1448A-WT-KB | OK | 63.8823 | 201.132 | 1.65465 | 1.62443 | 0.0422 | 0.183254 |
| PSPPH_2964 | sarcosine oxidase subunit alpha | chromosome:3438546-3441755 | 1448A-rhpS-KB | 1448A-WT-KB | OK | 14.6455 | 46.0752 | 1.65353 | 2.16205 | 0.0091 | 0.0647876 |
| cysC1 | adenylylsulfate kinase | chromosome:4912788-4913394 | 1448A-rhpS-KB | 1448A-WT-KB | OK | 45.911 | 144.32 | 1.65236 | 1.49893 | 0.0607 | 0.225511 |
| glyS | glycyl-tRNA synthetase subunit beta | chromosome:14353-17412 | 1448A-rhpS-KB | 1448A-WT-KB | OK | 185.33 | 578.306 | 1.64174 | 3.98496 | 0.00005 | 0.00121565 |
| PSPPH_1562 | hypothetical protein | chromosome:1813229-1814408 | 1448A-rhpS-KB | 1448A-WT-KB | OK | 30.7261 | 95.6997 | 1.63905 | 1.8981 | 0.0243 | 0.126601 |
| PSPPH_2998 | mandelate racemase | chromosome:3480219-3481374 | 1448A-rhpS-KB | 1448A-WT-KB | NOTEST | 13.832 | 42.9501 | 1.63465 | 0 | 1 | 1 |
| PSPPH_5204 | DeoR family transcriptional regulator | chromosome:5908357-5909125 | 1448A-rhpS-KB | 1448A-WT-KB | NOTEST | 10.6391 | 32.982 | 1.6323 | 0 | 1 | 1 |
| PSPPH_1931 | class V aminotransferase | chromosome:2274011-2276679 | 1448A-rhpS-KB | 1448A-WT-KB | NOTEST | 1.98904 | 6.15673 | 1.63009 | 0 | 1 | 1 |
| PSPPH_4288 | ATP-dependent protease | chromosome:4893566-4895840 | 1448A-rhpS-KB | 1448A-WT-KB | OK | 176.881 | 546.432 | 1.62726 | 4.77988 | 0.00005 | 0.00121565 |
| PSPPH_2978 | regulatory protein | chromosome:3457192-3458970 | 1448A-rhpS-KB | 1448A-WT-KB | NOTEST | 2.89744 | 8.94524 | 1.62634 | 0 | 1 | 1 |
| prpC | methylcitrate synthase | chromosome:2410020-2411148 | 1448A-rhpS-KB | 1448A-WT-KB | OK | 157.959 | 487.583 | 1.6261 | 3.63488 | 0.0001 | 0.00221988 |
| irp4 | yersiniabactin synthetase, thioesterase component Irp4 | chromosome:3368665-3369430 | 1448A-rhpS-KB | 1448A-WT-KB | NOTEST | 8.6707 | 26.7528 | 1.62547 | 0 | 1 | 1 |
| PSPPH_2856 | methyl-accepting chemotaxis protein | chromosome:3300407-3302369 | 1448A-rhpS-KB | 1448A-WT-KB | NOTEST | 3.8039 | 11.7261 | 1.62417 | 0 | 1 | 1 |
| PSPPH_0915 | MotA/TolQ/ExbB proton channel family protein | chromosome:1089700-1090351 | 1448A-rhpS-KB | 1448A-WT-KB | NOTEST | 11.6671 | 35.9098 | 1.62193 | 0 | 1 | 1 |
| PSPPH_5171 | LuxR family transcriptional regulator | chromosome:5866033-5867706 | 1448A-rhpS-KB | 1448A-WT-KB | OK | 20.687 | 63.5685 | 1.61959 | 0.95922 | 0.16745 | 0.394506 |
| PSPPH_2464 | glutamine ABC transporter ATP-binding protein | chromosome:2845187-2845925 | 1448A-rhpS-KB | 1448A-WT-KB | NOTEST | 4.64431 | 14.2712 | 1.61957 | 0 | 1 | 1 |
| PSPPH_1386 | amino acid ABC transporter permease | chromosome:1609057-1610478 | 1448A-rhpS-KB | 1448A-WT-KB | OK | 16.6327 | 51.0761 | 1.61863 | 1.08166 | 0.24665 | 0.480621 |
| PSPPH_1576 | glyoxalase | chromosome:1829880-1830258 | 1448A-rhpS-KB | 1448A-WT-KB | OK | 44.4847 | 136.546 | 1.61801 | 1.26683 | 0.1752 | 0.404499 |
| nikB | nickel ABC transporter permease | chromosome:2658194-2660773 | 1448A-rhpS-KB | 1448A-WT-KB | NOTEST | 3.96506 | 12.1425 | 1.61465 | 0 | 1 | 1 |
| PSPPH_4807 | glycosyl hydrolase | chromosome:5455307-5457728 | 1448A-rhpS-KB | 1448A-WT-KB | NOTEST | 11.3756 | 34.6723 | 1.60784 | 0 | 1 | 1 |
| PSPPH_0422 | phenylalanine ammonia-lyase/histidase | chromosome:482647-490911 | 1448A-rhpS-KB | 1448A-WT-KB | OK | 21.1987 | 64.5859 | 1.60724 | 1.48227 | 0.067 | 0.237087 |
| PSPPH_2350 | endoribonuclease L-PSP | chromosome:2725395-2727093 | 1448A-rhpS-KB | 1448A-WT-KB | NOTEST | 9.87675 | 29.8953 | 1.59781 | 0 | 1 | 1 |
| PSPPH_2728 | ribose ABC transporter ATP-binding protein | chromosome:3153737-3155621 | 1448A-rhpS-KB | 1448A-WT-KB | NOTEST | 3.53228 | 10.6517 | 1.59242 | 0 | 1 | 1 |
| PSPPH_0950 | methyl-accepting chemotaxis protein | chromosome:1127558-1129616 | 1448A-rhpS-KB | 1448A-WT-KB | OK | 20.3089 | 61.2128 | 1.59172 | 2.00673 | 0.0129 | 0.0838265 |
| PSPPH_1774 | xanthine dehydrogenase, C-terminal subunit | chromosome:2071299-2076102 | 1448A-rhpS-KB | 1448A-WT-KB | OK | 66.3969 | 199.999 | 1.5908 | 2.92195 | 0.00065 | 0.00925538 |
| PSPPH_4770 | hypothetical protein | chromosome:5414454-5417215 | 1448A-rhpS-KB | 1448A-WT-KB | OK | 28.0394 | 84.3701 | 1.58928 | 1.48989 | 0.0523 | 0.208351 |
| gcd | quinoprotein glucose dehydrogenase | chromosome:4485636-4488054 | 1448A-rhpS-KB | 1448A-WT-KB | OK | 67.89 | 204.274 | 1.58924 | 3.60085 | 0.00005 | 0.00121565 |
| PSPPH_4523 | fumarylacetoacetate hydrolase | chromosome:5155177-5156026 | 1448A-rhpS-KB | 1448A-WT-KB | OK | 26.5687 | 79.5267 | 1.58171 | 1.39482 | 0.0795 | 0.261392 |
| PSPPH_0132 | hypothetical protein | chromosome:150905-156854 | 1448A-rhpS-KB | 1448A-WT-KB | OK | 140.394 | 418.188 | 1.57466 | 1.53714 | 0.0455 | 0.192674 |
| PSPPH_1611 | dihydrodipicolinate synthetase | chromosome:1870154-1873726 | 1448A-rhpS-KB | 1448A-WT-KB | NOTEST | 9.34497 | 27.7894 | 1.57227 | 0 | 1 | 1 |
| shcM | type III chaperone protein ShcM | chromosome:1474484-1474979 | 1448A-rhpS-KB | 1448A-WT-KB | NOTEST | 9.81339 | 29.1065 | 1.56852 | 0 | 1 | 1 |
| qor2 | quinone oxidoreductase | chromosome:341597-342575 | 1448A-rhpS-KB | 1448A-WT-KB | OK | 26.2431 | 77.8296 | 1.56838 | 1.58674 | 0.0532 | 0.210328 |
| PSPPH_0299 | flagellar basal body protein FliL | chromosome:342705-343113 | 1448A-rhpS-KB | 1448A-WT-KB | OK | 109.59 | 324.89 | 1.56784 | 1.60792 | 0.0408 | 0.179139 |
| PSPPH_1545 | malate dehydrogenase | chromosome:1796725-1798417 | 1448A-rhpS-KB | 1448A-WT-KB | OK | 220.651 | 653.508 | 1.56644 | 4.48599 | 0.00005 | 0.00121565 |
| PSPPH_4760 | hypothetical protein | chromosome:5405596-5406100 | 1448A-rhpS-KB | 1448A-WT-KB | OK | 46.7943 | 138.386 | 1.56429 | 1.52247 | 0.10725 | 0.307141 |
| PSPPH_3068 | phosphinothricin N-acetyltransferase | chromosome:3563919-3564459 | 1448A-rhpS-KB | 1448A-WT-KB | OK | 25.8913 | 76.4011 | 1.56112 | 1.13478 | 0.2219 | 0.458953 |
| PSPPH_A0034 | PilT domain-containing protein | large_plasmid:29009-29924 | 1448A-rhpS-KB | 1448A-WT-KB | OK | 77.7306 | 229.355 | 1.56103 | 1.03445 | 0.1503 | 0.370464 |
| algK | alginate biosynthesis protein AlgK | chromosome:1311570-1314461 | 1448A-rhpS-KB | 1448A-WT-KB | OK | 68.7899 | 202.496 | 1.55763 | 2.51274 | 0.0031 | 0.0310347 |
| PSPPH_0121 | ImpA-like N-terminal family protein | chromosome:138815-139919 | 1448A-rhpS-KB | 1448A-WT-KB | OK | 68.5005 | 200.707 | 1.55091 | 2.5543 | 0.0018 | 0.0210235 |
| PSPPH_4805 | oxidoreductase | chromosome:5452395-5453496 | 1448A-rhpS-KB | 1448A-WT-KB | NOTEST | 10.233 | 29.9509 | 1.54937 | 0 | 1 | 1 |
| catD3 | 3-oxoadipate enol-lactonase | chromosome:2956131-2958458 | 1448A-rhpS-KB | 1448A-WT-KB | OK | 23.6822 | 69.3077 | 1.54921 | 1.15183 | 0.1922 | 0.421943 |
| chrR | transcriptional activator ChrR | chromosome:1101807-1103078 | 1448A-rhpS-KB | 1448A-WT-KB | OK | 35.48 | 103.687 | 1.54716 | 1.3394 | 0.07875 | 0.260846 |
| PSPPH_3295 | heavy metal sensor histidine kinase | chromosome:3817917-3819974 | 1448A-rhpS-KB | 1448A-WT-KB | NOTEST | 8.03025 | 23.4518 | 1.54618 | 0 | 1 | 1 |
| PSPPH_1768 | hypothetical protein | chromosome:2066158-2067600 | 1448A-rhpS-KB | 1448A-WT-KB | OK | 23.5428 | 68.7495 | 1.54606 | 0.716827 | 0.2989 | 0.533515 |
| PSPPH_1963 | electron transfer flavoprotein-ubiquinone oxidoreductase | chromosome:2310830-2312486 | 1448A-rhpS-KB | 1448A-WT-KB | OK | 273.584 | 798.538 | 1.54538 | 4.7948 | 0.00005 | 0.00121565 |
| cca | multifunctional tRNA nucleotidyl transferase/2'3'-cyclic phosphodiesterase/2'nucleotidase/phosphatase | chromosome:740236-741466 | 1448A-rhpS-KB | 1448A-WT-KB | OK | 70.6422 | 206.145 | 1.54506 | 2.6712 | 0.0012 | 0.014789 |
| PSPPH_5190 | sugar ABC transporter permease | chromosome:5890268-5892070 | 1448A-rhpS-KB | 1448A-WT-KB | OK | 164.634 | 479.739 | 1.54299 | 2.39521 | 0.00295 | 0.0299526 |
| hslV | ATP-dependent protease peptidase subunit | chromosome:435563-436094 | 1448A-rhpS-KB | 1448A-WT-KB | OK | 2215.16 | 6454.29 | 1.54285 | 5.22199 | 0.00005 | 0.00121565 |
| PSPPH_0128 | lipoprotein | chromosome:148074-150880 | 1448A-rhpS-KB | 1448A-WT-KB | OK | 53.1245 | 154.77 | 1.54268 | 0.936493 | 0.24605 | 0.479751 |
| PSPPH_1492 | major facilitator family transporter | chromosome:1733595-1734369 | 1448A-rhpS-KB | 1448A-WT-KB | NOTEST | 5.27167 | 15.353 | 1.54219 | 0 | 1 | 1 |
| PSPPH_4453 | hypothetical protein | chromosome:5087882-5088518 | 1448A-rhpS-KB | 1448A-WT-KB | NOTEST | 14.1275 | 41.136 | 1.54189 | 0 | 1 | 1 |
| gltB | glutamate synthase subunit alpha | chromosome:455632-460078 | 1448A-rhpS-KB | 1448A-WT-KB | OK | 111.048 | 322.853 | 1.53969 | 4.8083 | 0.00005 | 0.00121565 |
| PSPPH_1099 | D-isomer specific 2-hydroxyacid dehydrogenase | chromosome:1292975-1293950 | 1448A-rhpS-KB | 1448A-WT-KB | OK | 112.358 | 326.567 | 1.53927 | 2.86183 | 0.0007 | 0.00962231 |
| PSPPH_4799 | Zn-dependent protease | chromosome:5442280-5446423 | 1448A-rhpS-KB | 1448A-WT-KB | OK | 59.4507 | 172.716 | 1.53863 | 1.96166 | 0.0168 | 0.098918 |
| PSPPH_4627 | short chain dehydrogenase | chromosome:5264124-5264814 | 1448A-rhpS-KB | 1448A-WT-KB | OK | 106.099 | 308.026 | 1.53764 | 2.24564 | 0.00525 | 0.0437378 |
| PSPPH_2686 | prolidase | chromosome:3109765-3112332 | 1448A-rhpS-KB | 1448A-WT-KB | NOTEST | 6.86528 | 19.8835 | 1.53418 | 0 | 1 | 1 |
| PSPPH_3334 | hypothetical protein | chromosome:3862552-3864145 | 1448A-rhpS-KB | 1448A-WT-KB | NOTEST | 11.0356 | 31.941 | 1.53324 | 0 | 1 | 1 |
| fruK | 1-phosphofructokinase | chromosome:1010841-1014650 | 1448A-rhpS-KB | 1448A-WT-KB | OK | 99.9604 | 289.151 | 1.53239 | 1.95149 | 0.0109 | 0.0744868 |
| PSPPH_4029 | hypothetical protein | chromosome:4602130-4606623 | 1448A-rhpS-KB | 1448A-WT-KB | NOTEST | 9.20046 | 26.5881 | 1.531 | 0 | 1 | 1 |
| PSPPH_1570 | bile acid transporter family protein | chromosome:1825213-1826161 | 1448A-rhpS-KB | 1448A-WT-KB | OK | 35.588 | 102.64 | 1.52813 | 1.55049 | 0.04015 | 0.177156 |
| hrpR | type III transcriptional regulator HrpR | chromosome:1485637-1486558 | 1448A-rhpS-KB | 1448A-WT-KB | OK | 20.6554 | 59.5121 | 1.52666 | 1.21951 | 0.10675 | 0.307141 |
| PSPPH_1923 | pyoverdine sidechain peptide synthetase I, epsilon-Lys module | chromosome:2239720-2250972 | 1448A-rhpS-KB | 1448A-WT-KB | NOTEST | 2.16816 | 6.23168 | 1.52315 | 0 | 1 | 1 |
| potI | putrescine ABC transporter permease | chromosome:5562633-5565571 | 1448A-rhpS-KB | 1448A-WT-KB | OK | 88.3126 | 252.921 | 1.518 | 1.78594 | 0.02495 | 0.12919 |
| PSPPH_0793 | secretion protein | chromosome:936914-938126 | 1448A-rhpS-KB | 1448A-WT-KB | NOTEST | 8.60176 | 24.6097 | 1.51653 | 0 | 1 | 1 |
| argD | bifunctional N-succinyldiaminopimelate-aminotransferase/acetylornithine transaminase | chromosome:4925434-4927721 | 1448A-rhpS-KB | 1448A-WT-KB | OK | 102.13 | 291.997 | 1.51554 | 2.78009 | 0.00025 | 0.0043799 |
| PSPPH_0108 | lipoprotein | chromosome:126145-126481 | 1448A-rhpS-KB | 1448A-WT-KB | OK | 220.983 | 631.392 | 1.5146 | 1.86136 | 0.01845 | 0.105336 |
| dtd | D-tyrosyl-tRNA(Tyr) deacylase | chromosome:409774-410212 | 1448A-rhpS-KB | 1448A-WT-KB | OK | 72.0526 | 205.468 | 1.51179 | 1.34347 | 0.08145 | 0.264398 |
| madM | malonate transporter subunit MadM | chromosome:501982-502747 | 1448A-rhpS-KB | 1448A-WT-KB | OK | 22.8771 | 65.2033 | 1.51104 | 1.22418 | 0.15115 | 0.371505 |
| PSPPH_0286 | GntR family transcriptional regulator | chromosome:329238-329946 | 1448A-rhpS-KB | 1448A-WT-KB | OK | 18.4707 | 52.6253 | 1.51052 | 0.96934 | 0.18915 | 0.418331 |
| dgoD | galactonate dehydratase | chromosome:2304997-2306146 | 1448A-rhpS-KB | 1448A-WT-KB | OK | 107.216 | 305.285 | 1.50964 | 2.86699 | 0.00075 | 0.0101534 |
| fdhA1 | glutathione-independent formaldehyde dehydrogenase | chromosome:2960549-2961689 | 1448A-rhpS-KB | 1448A-WT-KB | NOTEST | 10.4752 | 29.6409 | 1.50061 | 0 | 1 | 1 |
| PSPPH_4996 | hypothetical protein | chromosome:5665899-5666697 | 1448A-rhpS-KB | 1448A-WT-KB | OK | 15.4986 | 43.8532 | 1.50054 | 0.970297 | 0.18895 | 0.418331 |
| PSPPH_1925 | pyoverdine sidechain peptide synthetase III, L-Thr-L-Ser component | chromosome:2250986-2257442 | 1448A-rhpS-KB | 1448A-WT-KB | NOTEST | 3.49045 | 9.85008 | 1.49672 | 0 | 1 | 1 |
| PSPPH_4928 | MFS permease-like protein | chromosome:5599725-5601459 | 1448A-rhpS-KB | 1448A-WT-KB | OK | 104.463 | 294.579 | 1.49566 | 3.2538 | 0.0001 | 0.00221988 |
| pbpC | penicillin-binding protein 1C | chromosome:5061997-5064346 | 1448A-rhpS-KB | 1448A-WT-KB | NOTEST | 13.3382 | 37.5458 | 1.49308 | 0 | 1 | 1 |
| PSPPH_2346 | amino acid ABC transporter permease | chromosome:2721085-2722838 | 1448A-rhpS-KB | 1448A-WT-KB | NOTEST | 8.54121 | 24.0204 | 1.49175 | 0 | 1 | 1 |
| hppD | 4-hydroxyphenylpyruvate dioxygenase | chromosome:3767649-3768726 | 1448A-rhpS-KB | 1448A-WT-KB | OK | 361.191 | 1015.21 | 1.49095 | 4.26943 | 0.00005 | 0.00121565 |
| PSPPH_4895 | spermidine/putrescine ABC transporter substrate-binding protein | chromosome:5566891-5567989 | 1448A-rhpS-KB | 1448A-WT-KB | OK | 236.413 | 663.985 | 1.48984 | 3.75186 | 0.00005 | 0.00121565 |
| PSPPH_0125 | ImcF-like family protein | chromosome:144123-148044 | 1448A-rhpS-KB | 1448A-WT-KB | OK | 47.3144 | 132.879 | 1.48977 | 3.52329 | 0.00005 | 0.00121565 |
| mdcA | malonate decarboxylase subunit alpha | chromosome:495425-497977 | 1448A-rhpS-KB | 1448A-WT-KB | OK | 193.624 | 541.771 | 1.48442 | 3.71143 | 0.00005 | 0.00121565 |
| acnD | aconitate hydratase | chromosome:2411217-2413809 | 1448A-rhpS-KB | 1448A-WT-KB | OK | 121.711 | 340.533 | 1.48434 | 4.15026 | 0.00005 | 0.00121565 |
| PSPPH_3333 | hypothetical protein | chromosome:3860620-3862501 | 1448A-rhpS-KB | 1448A-WT-KB | NOTEST | 10.304 | 28.7982 | 1.48278 | 0 | 1 | 1 |
| PSPPH_4809 | electron transfer flavoprotein subunit alpha | chromosome:5458598-5460378 | 1448A-rhpS-KB | 1448A-WT-KB | NOTEST | 8.27504 | 23.1086 | 1.48159 | 0 | 1 | 1 |
| PSPPH_5027 | acetyltransferase | chromosome:5695971-5696829 | 1448A-rhpS-KB | 1448A-WT-KB | OK | 110.111 | 306.955 | 1.47907 | 2.45551 | 0.003 | 0.0303739 |
| PSPPH_4785 | ATP-binding protein | chromosome:5430875-5431928 | 1448A-rhpS-KB | 1448A-WT-KB | OK | 51.9921 | 144.876 | 1.47845 | 1.97313 | 0.01525 | 0.0933279 |
| PSPPH_2103 | hypothetical protein | chromosome:2462400-2462808 | 1448A-rhpS-KB | 1448A-WT-KB | NOTEST | 11.4533 | 31.9094 | 1.47821 | 0 | 1 | 1 |
| acnA | aconitate hydratase | chromosome:3848557-3851302 | 1448A-rhpS-KB | 1448A-WT-KB | OK | 106.701 | 297.151 | 1.47762 | 4.01664 | 0.00005 | 0.00121565 |
| PSPPH_2858 | penicillin-binding protein | chromosome:3304624-3306514 | 1448A-rhpS-KB | 1448A-WT-KB | NOTEST | 10.8755 | 30.2831 | 1.47744 | 0 | 1 | 1 |
| PSPPH_0847 | phosphoenolpyruvate-protein phosphotransferase subunit EI/HPr/EIIA | chromosome:1010841-1014650 | 1448A-rhpS-KB | 1448A-WT-KB | OK | 91.2393 | 253.832 | 1.47615 | 3.56489 | 0.00005 | 0.00121565 |
| potG | putrescine ABC transporter ATP-binding protein | chromosome:5562633-5565571 | 1448A-rhpS-KB | 1448A-WT-KB | OK | 127.154 | 352.708 | 1.4719 | 2.64724 | 0.00055 | 0.0080893 |
| PSPPH_4997 | hypothetical protein | chromosome:5666832-5667972 | 1448A-rhpS-KB | 1448A-WT-KB | OK | 20.6222 | 57.1454 | 1.47044 | 1.32978 | 0.08345 | 0.266931 |
| PSPPH_3987 | hypothetical protein | chromosome:4551604-4553196 | 1448A-rhpS-KB | 1448A-WT-KB | OK | 19.3053 | 53.4864 | 1.47018 | 0.805794 | 0.3284 | 0.565253 |
| PSPPH_1911 | peptide synthase | chromosome:2216577-2229588 | 1448A-rhpS-KB | 1448A-WT-KB | NOTEST | 2.99548 | 8.29466 | 1.4694 | 0 | 1 | 1 |
| PSPPH_2503 | cation ABC transporter substrate-binding protein | chromosome:2889411-2890308 | 1448A-rhpS-KB | 1448A-WT-KB | NOTEST | 13.2881 | 36.7331 | 1.46695 | 0 | 1 | 1 |
| oxyR | oxidative stress regulatory protein OxyR | chromosome:228990-229914 | 1448A-rhpS-KB | 1448A-WT-KB | OK | 106.091 | 292.466 | 1.46297 | 2.47505 | 0.00195 | 0.0221952 |
| PSPPH_1528 | ISPsy21, transposase orfA | chromosome:1783339-1784565 | 1448A-rhpS-KB | 1448A-WT-KB | OK | 19.4596 | 53.5897 | 1.46148 | 0.585447 | 0.3578 | 0.592745 |
| PSPPH_4841 | AraC family transcriptional regulator | chromosome:5500509-5501409 | 1448A-rhpS-KB | 1448A-WT-KB | OK | 22.507 | 61.9741 | 1.46129 | 1.23805 | 0.0919 | 0.281449 |
| PSPPH_4049 | cardiolipin synthase | chromosome:4632020-4633178 | 1448A-rhpS-KB | 1448A-WT-KB | OK | 18.8498 | 51.8662 | 1.46024 | 1.36093 | 0.08135 | 0.264398 |
| PSPPH_3430 | decarboxylase | chromosome:3964867-3966289 | 1448A-rhpS-KB | 1448A-WT-KB | OK | 128.21 | 352.62 | 1.45961 | 3.33771 | 0.00005 | 0.00121565 |
| PSPPH_0424 | glycosyl transferase family protein | chromosome:482647-490911 | 1448A-rhpS-KB | 1448A-WT-KB | NOTEST | 16.2296 | 44.5208 | 1.45586 | 0 | 1 | 1 |
| eda2 | 2-dehydro-3-deoxy-6-phosphogalactonate aldolase | chromosome:2304308-2304944 | 1448A-rhpS-KB | 1448A-WT-KB | OK | 95.8465 | 262.883 | 1.45562 | 1.91893 | 0.0165 | 0.0983588 |
| PSPPH_1875 | hypothetical protein | chromosome:2175452-2176785 | 1448A-rhpS-KB | 1448A-WT-KB | OK | 17.1858 | 47.125 | 1.45527 | 0.511003 | 0.5154 | 0.72074 |
| PSPPH_4548 | hypothetical protein | chromosome:5183493-5186909 | 1448A-rhpS-KB | 1448A-WT-KB | OK | 67.064 | 183.676 | 1.45355 | 1.63289 | 0.04715 | 0.197258 |
| quiA | quinate/shikimate dehydrogenase | chromosome:3398436-3400806 | 1448A-rhpS-KB | 1448A-WT-KB | OK | 16.6626 | 45.6058 | 1.4526 | 1.89947 | 0.01845 | 0.105336 |
| PSPPH_3424 | hexapeptide repeat-containing transferase | chromosome:3959189-3960589 | 1448A-rhpS-KB | 1448A-WT-KB | NOTEST | 13.0353 | 35.6562 | 1.45173 | 0 | 1 | 1 |
| PSPPH_0653 | prophage PSPPH01, Cro/CI family transcriptional regulator | chromosome:771738-772386 | 1448A-rhpS-KB | 1448A-WT-KB | OK | 27.9873 | 76.5299 | 1.45125 | 1.24642 | 0.1275 | 0.338429 |
| vanA | vanillate monooxygenase, oxygenase subunit | chromosome:2858104-2859169 | 1448A-rhpS-KB | 1448A-WT-KB | OK | 116.456 | 318.374 | 1.45094 | 2.86417 | 0.0006 | 0.00875265 |
| PSPPH_0417 | hypothetical protein | chromosome:476539-481262 | 1448A-rhpS-KB | 1448A-WT-KB | OK | 26.0566 | 71.0817 | 1.44783 | 0.77857 | 0.23035 | 0.466972 |
| argK | phaseolotoxin-insensitive ornithine carbamoyltransferase | chromosome:4933153-4934137 | 1448A-rhpS-KB | 1448A-WT-KB | OK | 307.241 | 837.993 | 1.44757 | 3.73019 | 0.00005 | 0.00121565 |
| glnQ2 | amino acid ABC transporter ATP-binding protein | chromosome:2641714-2643155 | 1448A-rhpS-KB | 1448A-WT-KB | NOTEST | 9.27415 | 25.2895 | 1.44725 | 0 | 1 | 1 |
| PSPPH_2037 | permease | chromosome:2389319-2392519 | 1448A-rhpS-KB | 1448A-WT-KB | OK | 34.7975 | 94.8598 | 1.44681 | 2.31964 | 0.0047 | 0.0406726 |
| PSPPH_0448 | hypothetical protein | chromosome:509744-510323 | 1448A-rhpS-KB | 1448A-WT-KB | OK | 371.671 | 1012.88 | 1.44637 | 3.30363 | 0.00005 | 0.00121565 |
| PSPPH_2488 | BRO domain-containing protein | chromosome:2873872-2874412 | 1448A-rhpS-KB | 1448A-WT-KB | NOTEST | 12.3765 | 33.725 | 1.44622 | 0 | 1 | 1 |
| PSPPH_0406 | RpiR family transcriptional regulator | chromosome:469620-470481 | 1448A-rhpS-KB | 1448A-WT-KB | NOTEST | 13.1747 | 35.8518 | 1.44428 | 0 | 1 | 1 |
| PSPPH_A0063 | fimbrial chaperone protein, periplasmic | large_plasmid:51921-52659 | 1448A-rhpS-KB | 1448A-WT-KB | OK | 85.634 | 232.802 | 1.44285 | 2.03011 | 0.00865 | 0.0633275 |
| PSPPH_1501 | hypothetical protein | chromosome:1743163-1743541 | 1448A-rhpS-KB | 1448A-WT-KB | NOTEST | 12.7855 | 34.7409 | 1.44212 | 0 | 1 | 1 |
| PSPPH_4863 | histidine ABC transporter ATP-binding protein | chromosome:5528192-5529871 | 1448A-rhpS-KB | 1448A-WT-KB | OK | 31.2785 | 84.9033 | 1.44065 | 1.28824 | 0.0799 | 0.262148 |
| PSPPH_0938 | OprD family outer membrane porin | chromosome:1112305-1113574 | 1448A-rhpS-KB | 1448A-WT-KB | NOTEST | 6.1995 | 16.7928 | 1.43762 | 0 | 1 | 1 |
| PSPPH_0082 | Fis family transcriptional regulator | chromosome:89365-90688 | 1448A-rhpS-KB | 1448A-WT-KB | OK | 34.6377 | 93.7546 | 1.43655 | 1.86028 | 0.01945 | 0.108616 |
| PSPPH_4712 | acyl-CoA dehydrogenase | chromosome:5347125-5348904 | 1448A-rhpS-KB | 1448A-WT-KB | OK | 282.94 | 765.272 | 1.43548 | 4.42083 | 0.00005 | 0.00121565 |
| PSPPH_2223 | hypothetical protein | chromosome:2580404-2581647 | 1448A-rhpS-KB | 1448A-WT-KB | OK | 72.2247 | 195.251 | 1.43477 | 1.77479 | 0.05105 | 0.206628 |
| PSPPH_2812 | PAP2 superfamily protein | chromosome:3257660-3258416 | 1448A-rhpS-KB | 1448A-WT-KB | NOTEST | 10.6185 | 28.6677 | 1.43285 | 0 | 1 | 1 |
| rfbC | dTDP-4-dehydrorhamnose 3,5-epimerase | chromosome:174935-175481 | 1448A-rhpS-KB | 1448A-WT-KB | OK | 169.279 | 455.143 | 1.42691 | 2.17723 | 0.0064 | 0.0503824 |
| PSPPH_2480 | hypothetical protein | chromosome:2863263-2865617 | 1448A-rhpS-KB | 1448A-WT-KB | NOTEST | 5.56064 | 14.9195 | 1.42387 | 0 | 1 | 1 |
| PSPPH_4796 | TraX family protein | chromosome:5440266-5441013 | 1448A-rhpS-KB | 1448A-WT-KB | OK | 35.1898 | 94.3644 | 1.42308 | 1.25933 | 0.09495 | 0.285409 |
| potH | putrescine ABC transporter permease | chromosome:5562633-5565571 | 1448A-rhpS-KB | 1448A-WT-KB | OK | 98.6393 | 263.813 | 1.41928 | 1.94389 | 0.0105 | 0.0721673 |
| PSPPH_1041 | hypothetical protein | chromosome:1231112-1232456 | 1448A-rhpS-KB | 1448A-WT-KB | NOTEST | 16.0902 | 42.8613 | 1.41349 | 0 | 1 | 1 |
| PSPPH_0814 | HlyD family type I secretion membrane fusion protein | chromosome:958235-961743 | 1448A-rhpS-KB | 1448A-WT-KB | NOTEST | 9.97156 | 26.4563 | 1.40772 | 0 | 1 | 1 |
| tkt | transketolase | chromosome:5472147-5474145 | 1448A-rhpS-KB | 1448A-WT-KB | OK | 431.952 | 1141.81 | 1.40238 | 4.87301 | 0.00005 | 0.00121565 |
| cdd | cytosine deaminase | chromosome:1716267-1716705 | 1448A-rhpS-KB | 1448A-WT-KB | OK | 47.9291 | 126.29 | 1.39776 | 0.981726 | 0.1774 | 0.406428 |
| braD | branched-chain amino acid ABC transporter permease | chromosome:699021-699936 | 1448A-rhpS-KB | 1448A-WT-KB | OK | 21.0273 | 55.2469 | 1.39363 | 1.10251 | 0.13475 | 0.349495 |
| PSPPH_0623 | glycerol-3-phosphate acyltransferase PlsY | chromosome:738627-739206 | 1448A-rhpS-KB | 1448A-WT-KB | OK | 59.4906 | 156.171 | 1.39239 | 1.40152 | 0.06015 | 0.224587 |
| PSPPH_1255 | transketolase, C-terminal subunit | chromosome:1461360-1463225 | 1448A-rhpS-KB | 1448A-WT-KB | NOTEST | 10.1235 | 26.5447 | 1.39072 | 0 | 1 | 1 |
| tauB | taurine ABC transporter ATP-binding subunit | chromosome:5583347-5585005 | 1448A-rhpS-KB | 1448A-WT-KB | NOTEST | 9.56491 | 25.0452 | 1.38871 | 0 | 1 | 1 |
| deoC | deoxyribose-phosphate aldolase | chromosome:1034752-1035532 | 1448A-rhpS-KB | 1448A-WT-KB | NOTEST | 12.874 | 33.7061 | 1.38854 | 0 | 1 | 1 |
| PSPPH_2222 | hypothetical protein | chromosome:2580404-2581647 | 1448A-rhpS-KB | 1448A-WT-KB | OK | 32.1156 | 84.0556 | 1.38807 | 0.498236 | 0.34365 | 0.57888 |
| PSPPH_0469 | twitching motility protein | chromosome:532036-533071 | 1448A-rhpS-KB | 1448A-WT-KB | OK | 179.112 | 468.247 | 1.38641 | 2.99623 | 0.0003 | 0.0050338 |
| PSPPH_2336 | Gfo/Idh/MocA family oxidoreductase | chromosome:2708124-2710011 | 1448A-rhpS-KB | 1448A-WT-KB | NOTEST | 3.2045 | 8.37286 | 1.38562 | 0 | 1 | 1 |
| PSPPH_3080 | ABC transporter substrate-binding protein | chromosome:3576870-3578544 | 1448A-rhpS-KB | 1448A-WT-KB | OK | 43.0181 | 112.127 | 1.38212 | 1.12224 | 0.11145 | 0.313887 |
| mtlZ | fructokinase | chromosome:2985655-2986594 | 1448A-rhpS-KB | 1448A-WT-KB | OK | 161.079 | 419.817 | 1.38199 | 2.88362 | 0.00055 | 0.0080893 |
| PSPPH_2378 | multidrug resistance protein | chromosome:2756887-2758096 | 1448A-rhpS-KB | 1448A-WT-KB | NOTEST | 2.52811 | 6.58889 | 1.38198 | 0 | 1 | 1 |
| PSPPH_2739 | hypothetical protein | chromosome:3170918-3171539 | 1448A-rhpS-KB | 1448A-WT-KB | NOTEST | 6.23082 | 16.2254 | 1.38076 | 0 | 1 | 1 |
| terA | tellurium resistance protein TerA | chromosome:996297-998099 | 1448A-rhpS-KB | 1448A-WT-KB | OK | 352.819 | 917.624 | 1.37898 | 3.24618 | 0.00005 | 0.00121565 |
| PSPPH_0331 | ubiquinone/menaquinone biosynthesis methyltransferase ubie | chromosome:377759-378386 | 1448A-rhpS-KB | 1448A-WT-KB | OK | 32.6726 | 84.9619 | 1.37874 | 1.08434 | 0.13425 | 0.348829 |
| PSPPH_2556 | spermidine/putrescine ABC transporter ATP-binding protein | chromosome:2948350-2950317 | 1448A-rhpS-KB | 1448A-WT-KB | OK | 24.7801 | 64.3867 | 1.37758 | 1.32599 | 0.10035 | 0.294217 |
| PSPPH_1387 | amino acid ABC transporter permease | chromosome:1609057-1610478 | 1448A-rhpS-KB | 1448A-WT-KB | NOTEST | 10.3091 | 26.7664 | 1.37651 | 0 | 1 | 1 |
| gntR | gluconate utilization system GNT-I transcriptional repressor | chromosome:3775279-3776296 | 1448A-rhpS-KB | 1448A-WT-KB | OK | 67.0015 | 173.89 | 1.3759 | 2.01554 | 0.00925 | 0.0655942 |
| PSPPH_4842 | MtlE | chromosome:5501690-5503007 | 1448A-rhpS-KB | 1448A-WT-KB | NOTEST | 2.28698 | 5.92934 | 1.37443 | 0 | 1 | 1 |
| PSPPH_3256 | hypothetical protein | chromosome:3773979-3774951 | 1448A-rhpS-KB | 1448A-WT-KB | OK | 26.3649 | 68.272 | 1.37267 | 1.29653 | 0.08005 | 0.262235 |
| PSPPH_2239 | glycosyl transferase family protein | chromosome:2597977-2599996 | 1448A-rhpS-KB | 1448A-WT-KB | NOTEST | 14.0543 | 36.377 | 1.37201 | 0 | 1 | 1 |
| PSPPH_2906 | multidrug resistance protein AcrA/AcrE family | chromosome:3373535-3374696 | 1448A-rhpS-KB | 1448A-WT-KB | OK | 49.3521 | 127.697 | 1.37154 | 2.00222 | 0.0118 | 0.0783888 |
| smf | DNA processing protein DprA | chromosome:28296-29415 | 1448A-rhpS-KB | 1448A-WT-KB | OK | 22.9811 | 59.4584 | 1.37143 | 1.34536 | 0.078 | 0.259731 |
| htpG | heat shock protein 90 | chromosome:2335773-2337681 | 1448A-rhpS-KB | 1448A-WT-KB | OK | 2818.49 | 7288.37 | 1.37068 | 6.27376 | 0.00005 | 0.00121565 |
| scrB | sucrose-6-phosphate hydrolase | chromosome:5893260-5894754 | 1448A-rhpS-KB | 1448A-WT-KB | OK | 275.86 | 711.911 | 1.36776 | 4.1153 | 0.00005 | 0.00121565 |
| dgoK | 2-dehydro-3-deoxygalactonokinase | chromosome:2303302-2304307 | 1448A-rhpS-KB | 1448A-WT-KB | OK | 98.3069 | 253.163 | 1.3647 | 2.3399 | 0.00275 | 0.0284061 |
| cstA | carbon starvation protein CstA | chromosome:4949692-4951768 | 1448A-rhpS-KB | 1448A-WT-KB | OK | 57.4117 | 147.245 | 1.35881 | 2.57047 | 0.0011 | 0.0137462 |
| PSPPH_2491 | LysR family transcriptional regulator | chromosome:2877507-2878413 | 1448A-rhpS-KB | 1448A-WT-KB | NOTEST | 11.0592 | 28.3553 | 1.35837 | 0 | 1 | 1 |
| PSPPH_2802 | aminotransferase | chromosome:3246757-3252777 | 1448A-rhpS-KB | 1448A-WT-KB | NOTEST | 8.02744 | 20.5744 | 1.35784 | 0 | 1 | 1 |
| PSPPH_4542 | hypothetical protein | chromosome:5176816-5178747 | 1448A-rhpS-KB | 1448A-WT-KB | OK | 20.643 | 52.8994 | 1.3576 | 0.997362 | 0.19165 | 0.421512 |
| PSPPH_2892 | TonB-dependent siderophore receptor | chromosome:3341358-3344792 | 1448A-rhpS-KB | 1448A-WT-KB | NOTEST | 5.88185 | 15.0464 | 1.35508 | 0 | 1 | 1 |
| PSPPH_4693 | hypothetical protein | chromosome:5327863-5328253 | 1448A-rhpS-KB | 1448A-WT-KB | OK | 67.4793 | 172.493 | 1.35402 | 1.06456 | 0.1435 | 0.361301 |
| prpB | 2-methylisocitrate lyase | chromosome:2408125-2409723 | 1448A-rhpS-KB | 1448A-WT-KB | OK | 340.514 | 870.054 | 1.35339 | 3.19023 | 0.00015 | 0.00302881 |
| PSPPH_4764 | transmembrane sensor | chromosome:5408344-5409843 | 1448A-rhpS-KB | 1448A-WT-KB | NOTEST | 12.1481 | 31.036 | 1.35321 | 0 | 1 | 1 |
| PSPPH_0117 | phospholipase D | chromosome:134131-136063 | 1448A-rhpS-KB | 1448A-WT-KB | OK | 50.4916 | 128.963 | 1.35284 | 2.37302 | 0.00255 | 0.0266482 |
| PSPPH_5049 | TonB system transport protein | chromosome:5722332-5725140 | 1448A-rhpS-KB | 1448A-WT-KB | NOTEST | 7.21322 | 18.3921 | 1.35037 | 0 | 1 | 1 |
| argD1 | acetylornithine aminotransferase | chromosome:2878516-2879719 | 1448A-rhpS-KB | 1448A-WT-KB | NOTEST | 2.87443 | 7.32743 | 1.35003 | 0 | 1 | 1 |
| PSPPH_3573 | hypothetical protein | chromosome:4117430-4117829 | 1448A-rhpS-KB | 1448A-WT-KB | OK | 85.5517 | 218.062 | 1.34987 | 1.18756 | 0.11175 | 0.314236 |
| hemC | porphobilinogen deaminase | chromosome:67335-69092 | 1448A-rhpS-KB | 1448A-WT-KB | OK | 127.852 | 325.83 | 1.34964 | 2.33317 | 0.00275 | 0.0284061 |
| PSPPH_2301 | LysR family transcriptional regulator | chromosome:2667567-2668476 | 1448A-rhpS-KB | 1448A-WT-KB | NOTEST | 7.75365 | 19.732 | 1.34759 | 0 | 1 | 1 |
| bioA1 | adenosylmethionine-8-amino-7-oxononanoate aminotransferase | chromosome:505336-506743 | 1448A-rhpS-KB | 1448A-WT-KB | OK | 60.0729 | 152.627 | 1.34523 | 2.27748 | 0.00415 | 0.0374548 |
| envZ | osmolarity sensor protein EnvZ | chromosome:287131-288454 | 1448A-rhpS-KB | 1448A-WT-KB | OK | 89.23 | 226.637 | 1.34478 | 2.48745 | 0.00245 | 0.0257538 |
| PSPPH_1743 | L-sorbosone dehydrogenase | chromosome:2020877-2022595 | 1448A-rhpS-KB | 1448A-WT-KB | OK | 30.319 | 77.0068 | 1.34476 | 1.56276 | 0.03925 | 0.175569 |
| metR | transcriptional regulator MetR | chromosome:4471036-4471954 | 1448A-rhpS-KB | 1448A-WT-KB | OK | 55.5136 | 140.971 | 1.34448 | 1.73011 | 0.02265 | 0.120823 |
| gabD | succinate-semialdehyde dehydrogenase I | chromosome:106197-107640 | 1448A-rhpS-KB | 1448A-WT-KB | OK | 416.929 | 1056.01 | 1.34075 | 4.25967 | 0.00005 | 0.00121565 |
| thiC | thiamine biosynthesis protein ThiC | chromosome:628825-630715 | 1448A-rhpS-KB | 1448A-WT-KB | OK | 349.352 | 884.625 | 1.34038 | 4.49447 | 0.00005 | 0.00121565 |
| PSPPH_4277 | glycine betaine/carnitine/choline ABC transporter permease | chromosome:4884111-4885919 | 1448A-rhpS-KB | 1448A-WT-KB | OK | 37.8493 | 95.7262 | 1.33865 | 1.07243 | 0.1491 | 0.369545 |
| PSPPH_4349 | HAD superfamily hydrolase | chromosome:4972107-4972671 | 1448A-rhpS-KB | 1448A-WT-KB | OK | 41.5682 | 105.078 | 1.33791 | 1.11417 | 0.11815 | 0.325072 |
| PSPPH_4896 | aminotransferase | chromosome:5568128-5569496 | 1448A-rhpS-KB | 1448A-WT-KB | OK | 80.0274 | 202.189 | 1.33714 | 2.3848 | 0.00315 | 0.0310997 |
| PSPPH_3654 | TetR family transcriptional regulator | chromosome:4197239-4197809 | 1448A-rhpS-KB | 1448A-WT-KB | OK | 83.2065 | 210.222 | 1.33714 | 1.52259 | 0.0504 | 0.204693 |
| PSPPH_1385 | succinylglutamate desuccinylase/aspartoacylase | chromosome:1607926-1609045 | 1448A-rhpS-KB | 1448A-WT-KB | OK | 26.0057 | 65.6642 | 1.33628 | 1.35006 | 0.0655 | 0.2347 |
| PSPPH_1227 | hypothetical protein | chromosome:1436270-1436843 | 1448A-rhpS-KB | 1448A-WT-KB | OK | 136.405 | 344.005 | 1.33453 | 1.94853 | 0.01255 | 0.0823004 |
| PSPPH_2542 | hypothetical protein | chromosome:2930403-2931572 | 1448A-rhpS-KB | 1448A-WT-KB | NOTEST | 5.32569 | 13.3953 | 1.33069 | 0 | 1 | 1 |
| acsA | acetyl-CoA synthetase | chromosome:4066945-4068901 | 1448A-rhpS-KB | 1448A-WT-KB | OK | 88.2884 | 221.947 | 1.32992 | 2.948 | 0.00015 | 0.00302881 |
| PSPPH_1221 | cyclohexadienyl dehydratase | chromosome:1429640-1430432 | 1448A-rhpS-KB | 1448A-WT-KB | OK | 47.8271 | 120.227 | 1.32986 | 1.45441 | 0.05195 | 0.208051 |
| PSPPH_2749 | outer membrane ferric siderophore receptor | chromosome:3180640-3183049 | 1448A-rhpS-KB | 1448A-WT-KB | NOTEST | 16.2635 | 40.843 | 1.32845 | 0 | 1 | 1 |
| PSPPH_3348 | hypothetical protein | chromosome:3874212-3877536 | 1448A-rhpS-KB | 1448A-WT-KB | NOTEST | 17.7244 | 44.4789 | 1.32739 | 0 | 1 | 1 |
| PSPPH_0705 | alkylphosphonate ABC transporter ATP-binding protein | chromosome:827742-830778 | 1448A-rhpS-KB | 1448A-WT-KB | OK | 25.8108 | 64.7487 | 1.32688 | 0.837507 | 0.2974 | 0.532252 |
| PSPPH_0646 | polyamine ABC transporter permease | chromosome:764614-765436 | 1448A-rhpS-KB | 1448A-WT-KB | OK | 21.0797 | 52.8519 | 1.3261 | 0.925343 | 0.18745 | 0.416374 |
| mpl | UDP-N-acetylmuramate:L-alanyl-gamma-D-glutamyl- meso-diaminopimelate ligase | chromosome:5299385-5301707 | 1448A-rhpS-KB | 1448A-WT-KB | OK | 150.72 | 377.338 | 1.32399 | 3.00285 | 0.00015 | 0.00302881 |
| PSPPH_2941 | methyl-accepting chemotaxis protein | chromosome:3411730-3413620 | 1448A-rhpS-KB | 1448A-WT-KB | NOTEST | 8.00375 | 20.035 | 1.32377 | 0 | 1 | 1 |
| PSPPH_1543 | hypothetical protein | chromosome:1795125-1795663 | 1448A-rhpS-KB | 1448A-WT-KB | OK | 76.8447 | 192.086 | 1.32173 | 0.958859 | 0.4174 | 0.644679 |
| PSPPH_4953 | prophage PSPPH06 tail fiber protein | chromosome:5625698-5628143 | 1448A-rhpS-KB | 1448A-WT-KB | NOTEST | 6.3155 | 15.7789 | 1.32102 | 0 | 1 | 1 |
| PSPPH_5097 | CAIB/BAIF family protein | chromosome:5784953-5785628 | 1448A-rhpS-KB | 1448A-WT-KB | NOTEST | 7.14233 | 17.8318 | 1.31998 | 0 | 1 | 1 |
| PSPPH_1871 | glutamate carboxypeptidase | chromosome:2173048-2174293 | 1448A-rhpS-KB | 1448A-WT-KB | OK | 29.7282 | 74.177 | 1.31914 | 1.49028 | 0.04515 | 0.191874 |
| PSPPH_4373 | hypothetical protein | chromosome:4995108-4995801 | 1448A-rhpS-KB | 1448A-WT-KB | OK | 24.3344 | 60.6854 | 1.31835 | 0.913086 | 0.2062 | 0.441822 |
| PSPPH_0973 | hypothetical protein | chromosome:1159798-1161253 | 1448A-rhpS-KB | 1448A-WT-KB | OK | 23.7624 | 59.1679 | 1.31613 | 1.3806 | 0.05115 | 0.206799 |
| PSPPH_0141 | LysR family transcriptional regulator | chromosome:167839-168721 | 1448A-rhpS-KB | 1448A-WT-KB | OK | 103.65 | 257.823 | 1.31466 | 2.148 | 0.0049 | 0.0418962 |
| PSPPH_0152 | carbon-nitrogen hydrolase | chromosome:180220-181099 | 1448A-rhpS-KB | 1448A-WT-KB | OK | 166.742 | 414.193 | 1.31269 | 2.71493 | 0.0007 | 0.00962231 |
| cobL | precorrin-6Y C5,15-methyltransferase | chromosome:5095634-5098665 | 1448A-rhpS-KB | 1448A-WT-KB | OK | 41.9977 | 104.163 | 1.31046 | 1.13039 | 0.10865 | 0.309168 |
| PSPPH_2665 | IclR family transcriptional regulator | chromosome:3086137-3086902 | 1448A-rhpS-KB | 1448A-WT-KB | OK | 55.3047 | 136.888 | 1.30753 | 1.53885 | 0.04285 | 0.184951 |
| PSPPH_0593 | high affinity branched-chain amino acid ABC transporter ATP-binding protein | chromosome:699938-702837 | 1448A-rhpS-KB | 1448A-WT-KB | OK | 25.3658 | 62.7291 | 1.30625 | 0.877728 | 0.1964 | 0.428269 |
| PSPPH_4053 | hypothetical protein | chromosome:4633842-4637746 | 1448A-rhpS-KB | 1448A-WT-KB | NOTEST | 3.0873 | 7.62771 | 1.3049 | 0 | 1 | 1 |
| PSPPH_1251 | OPT family oligopeptide transporter | chromosome:1457520-1459266 | 1448A-rhpS-KB | 1448A-WT-KB | NOTEST | 11.3909 | 28.1406 | 1.30477 | 0 | 1 | 1 |
| mtaP | 5'-methylthioadenosine phosphorylase | chromosome:3711453-3712194 | 1448A-rhpS-KB | 1448A-WT-KB | OK | 138.258 | 341.463 | 1.30437 | 2.18018 | 0.0049 | 0.0418962 |
| PSPPH_2725 | sorbitol dehydrogenase | chromosome:3150558-3151617 | 1448A-rhpS-KB | 1448A-WT-KB | NOTEST | 9.14401 | 22.56 | 1.30287 | 0 | 1 | 1 |
| PSPPH_4263 | peptide ABC transporter permease | chromosome:4867235-4869168 | 1448A-rhpS-KB | 1448A-WT-KB | OK | 116.445 | 287.118 | 1.302 | 2.23055 | 0.00345 | 0.0327064 |
| PSPPH_3266 | TonB-dependent siderophore receptor | chromosome:3784620-3786675 | 1448A-rhpS-KB | 1448A-WT-KB | NOTEST | 16.3501 | 40.3071 | 1.30173 | 0 | 1 | 1 |
| srl | DeoR family transcriptional regulator | chromosome:3973814-3974585 | 1448A-rhpS-KB | 1448A-WT-KB | OK | 32.5873 | 80.3067 | 1.30121 | 1.20445 | 0.09715 | 0.289104 |
| PSPPH_0334 | D-methionine-binding lipoprotein MetQ | chromosome:378442-380992 | 1448A-rhpS-KB | 1448A-WT-KB | NOTEST | 10.9961 | 27.0765 | 1.30006 | 0 | 1 | 1 |
| PSPPH_A0094 | prevent-host-death family protein | large_plasmid:82423-82672 | 1448A-rhpS-KB | 1448A-WT-KB | OK | 217.339 | 535.127 | 1.29994 | 1.13218 | 0.1258 | 0.336282 |
| PSPPH_2963 | sarcosine oxidase subunit delta | chromosome:3438546-3441755 | 1448A-rhpS-KB | 1448A-WT-KB | OK | 20.8921 | 51.2307 | 1.29405 | 0.488453 | 0.5909 | 0.770954 |
| dppF | dipeptide transporter ATP-binding subunit | chromosome:4869178-4871121 | 1448A-rhpS-KB | 1448A-WT-KB | OK | 143.72 | 351.732 | 1.29121 | 2.27101 | 0.0034 | 0.0323181 |
| PSPPH_4265 | dipeptide ABC transporter ATP-binding protein | chromosome:4869178-4871121 | 1448A-rhpS-KB | 1448A-WT-KB | OK | 131.362 | 321.314 | 1.29043 | 2.11443 | 0.0043 | 0.0379462 |
| amn | AMP nucleosidase | chromosome:5000502-5001966 | 1448A-rhpS-KB | 1448A-WT-KB | OK | 157.351 | 384.653 | 1.28958 | 3.09762 | 0.00015 | 0.00302881 |
| PSPPH_1135 | isoflavone reductase | chromosome:1335134-1336073 | 1448A-rhpS-KB | 1448A-WT-KB | NOTEST | 12.2871 | 30.0063 | 1.28812 | 0 | 1 | 1 |
| rbsR | ribose operon repressor | chromosome:2485625-2486642 | 1448A-rhpS-KB | 1448A-WT-KB | OK | 34.9823 | 85.349 | 1.28675 | 1.38031 | 0.0607 | 0.225511 |
| hslU | ATP-dependent protease ATP-binding subunit HslU | chromosome:434159-435497 | 1448A-rhpS-KB | 1448A-WT-KB | OK | 1980.77 | 4828.09 | 1.28539 | 5.17937 | 0.00005 | 0.00121565 |
| PSPPH_4165 | carbon-nitrogen family hydrolase | chromosome:4748336-4749182 | 1448A-rhpS-KB | 1448A-WT-KB | OK | 64.3139 | 156.668 | 1.28451 | 1.65872 | 0.0284 | 0.139617 |
| PSPPH_0964 | hypothetical protein | chromosome:1145940-1147680 | 1448A-rhpS-KB | 1448A-WT-KB | OK | 61.7208 | 150.267 | 1.2837 | 2.23098 | 0.00585 | 0.0471962 |
| PSPPH_2738 | glutamate-1-semialdehyde aminotransferase | chromosome:3167121-3170908 | 1448A-rhpS-KB | 1448A-WT-KB | NOTEST | 6.47008 | 15.7472 | 1.28324 | 0 | 1 | 1 |
| PSPPH_1054 | ompA family protein | chromosome:1246445-1247135 | 1448A-rhpS-KB | 1448A-WT-KB | OK | 292.377 | 711.345 | 1.28272 | 2.78999 | 0.00015 | 0.00302881 |
| PSPPH_1756 | methyl-accepting chemotaxis protein | chromosome:2054446-2056375 | 1448A-rhpS-KB | 1448A-WT-KB | NOTEST | 7.32044 | 17.7936 | 1.28136 | 0 | 1 | 1 |
| PSPPH_0094 | response regulator | chromosome:103159-104551 | 1448A-rhpS-KB | 1448A-WT-KB | OK | 84.6284 | 205.701 | 1.28134 | 2.37336 | 0.00265 | 0.0275323 |
| PSPPH_4455 | hypothetical protein | chromosome:5088553-5089880 | 1448A-rhpS-KB | 1448A-WT-KB | NOTEST | 5.78446 | 14.0367 | 1.27895 | 0 | 1 | 1 |
| PSPPH_1257 | MFS transporter | chromosome:1463243-1464641 | 1448A-rhpS-KB | 1448A-WT-KB | NOTEST | 5.54899 | 13.4627 | 1.27867 | 0 | 1 | 1 |
| PSPPH_0685 | iron-sulfur cluster insertion protein ErpA | chromosome:799151-799502 | 1448A-rhpS-KB | 1448A-WT-KB | OK | 72.9092 | 176.627 | 1.27654 | 1.19756 | 0.2245 | 0.462663 |
| PSPPH_4628 | hydroxydechloroatrazine ethylaminohydrolase | chromosome:5264937-5266293 | 1448A-rhpS-KB | 1448A-WT-KB | NOTEST | 15.3296 | 37.1353 | 1.27647 | 0 | 1 | 1 |
| proC | pyrroline-5-carboxylate reductase | chromosome:530453-531981 | 1448A-rhpS-KB | 1448A-WT-KB | OK | 451.801 | 1093.98 | 1.27583 | 3.20335 | 0.00005 | 0.00121565 |
| mdoD | glucan biosynthesis protein D | chromosome:5860575-5862195 | 1448A-rhpS-KB | 1448A-WT-KB | OK | 287.991 | 697.056 | 1.27525 | 3.8886 | 0.00005 | 0.00121565 |
| PSPPH_3468 | LysR family transcriptional regulator | chromosome:4006948-4007854 | 1448A-rhpS-KB | 1448A-WT-KB | OK | 83.2669 | 201.431 | 1.27447 | 1.96991 | 0.01135 | 0.0762498 |
| mmsA1 | methylmalonate-semialdehyde dehydrogenase | chromosome:3701204-3702707 | 1448A-rhpS-KB | 1448A-WT-KB | NOTEST | 14.5028 | 34.9774 | 1.27009 | 0 | 1 | 1 |
| PSPPH_3265 | acetyltransferase | chromosome:3784006-3784444 | 1448A-rhpS-KB | 1448A-WT-KB | OK | 22.4095 | 53.9496 | 1.26751 | 0.702229 | 0.41115 | 0.639447 |
| PSPPH_4226 | tail tape meausure protein, truncated | chromosome:4819556-4820942 | 1448A-rhpS-KB | 1448A-WT-KB | NOTEST | 15.3596 | 36.9501 | 1.26643 | 0 | 1 | 1 |
| PSPPH_0962 | hypothetical protein | chromosome:1142614-1144397 | 1448A-rhpS-KB | 1448A-WT-KB | OK | 140.842 | 338.805 | 1.26638 | 1.72673 | 0.01815 | 0.10429 |
| ubiH | 2-octaprenyl-6-methoxyphenyl hydroxylase | chromosome:346191-348710 | 1448A-rhpS-KB | 1448A-WT-KB | OK | 109.239 | 262.756 | 1.26623 | 1.87882 | 0.0166 | 0.0983588 |
| cobA | uroporphyrin-III C-methyltransferase | chromosome:2424367-2425129 | 1448A-rhpS-KB | 1448A-WT-KB | NOTEST | 10.3726 | 24.9067 | 1.26376 | 0 | 1 | 1 |
| PSPPH_3733 | hypothetical protein | chromosome:4280364-4280898 | 1448A-rhpS-KB | 1448A-WT-KB | NOTEST | 18.6674 | 44.7859 | 1.26252 | 0 | 1 | 1 |
| gspD1 | general secretion pathway protein GspD | chromosome:3548347-3550627 | 1448A-rhpS-KB | 1448A-WT-KB | OK | 35.6566 | 85.4649 | 1.26116 | 2.12949 | 0.0052 | 0.0434224 |
| cobO | cob(I)yrinic acid a,c-diamide adenosyltransferase | chromosome:4248815-4249427 | 1448A-rhpS-KB | 1448A-WT-KB | OK | 64.6721 | 154.943 | 1.26052 | 1.34286 | 0.0658 | 0.2347 |
| PSPPH_2999 | peptide ABC transporter substrate-binding protein | chromosome:3481460-3483083 | 1448A-rhpS-KB | 1448A-WT-KB | NOTEST | 4.85843 | 11.6032 | 1.25596 | 0 | 1 | 1 |
| PSPPH_3186 | Gfo/Idh/MocA family oxidoreductase | chromosome:3695266-3696286 | 1448A-rhpS-KB | 1448A-WT-KB | OK | 42.2954 | 100.604 | 1.25011 | 1.46703 | 0.05005 | 0.204433 |
| PSPPH_1121 | PhoH-like protein | chromosome:1321843-1323238 | 1448A-rhpS-KB | 1448A-WT-KB | OK | 259.107 | 615.9 | 1.24915 | 3.47078 | 0.00005 | 0.00121565 |
| PSPPH_2469 | LysR family transcriptional regulator | chromosome:2851372-2852272 | 1448A-rhpS-KB | 1448A-WT-KB | OK | 40.5403 | 96.3327 | 1.24867 | 1.3559 | 0.0683 | 0.24026 |
| sdaA | L-serine ammonia-lyase | chromosome:3999193-4000570 | 1448A-rhpS-KB | 1448A-WT-KB | OK | 803.415 | 1908.88 | 1.24851 | 4.41209 | 0.00005 | 0.00121565 |
| PSPPH_2224 | cobalamin synthesis protein/P47K family protein | chromosome:2582359-2588302 | 1448A-rhpS-KB | 1448A-WT-KB | OK | 67.0254 | 159.008 | 1.24632 | 1.70355 | 0.0233 | 0.123715 |
| PSPPH_4536 | glycosyl hydrolase | chromosome:5168942-5171556 | 1448A-rhpS-KB | 1448A-WT-KB | OK | 32.5474 | 77.1779 | 1.24565 | 1.2053 | 0.09385 | 0.283773 |
| uraA | uracil transporter | chromosome:1208022-1209300 | 1448A-rhpS-KB | 1448A-WT-KB | OK | 82.2723 | 194.903 | 1.24428 | 2.21315 | 0.004 | 0.0364694 |
| PSPPH_2690 | FAD-binding oxidoreductase | chromosome:3114514-3117562 | 1448A-rhpS-KB | 1448A-WT-KB | OK | 50.9858 | 120.524 | 1.24115 | 2.62326 | 0.0009 | 0.0118257 |
| algC | alginate biosynthesis protein AlgC | chromosome:243310-244708 | 1448A-rhpS-KB | 1448A-WT-KB | OK | 457.476 | 1079.58 | 1.23871 | 4.0718 | 0.00005 | 0.00121565 |
| PSPPH_3021 | nucleoside 2-deoxyribosyltransferase | chromosome:3507697-3508213 | 1448A-rhpS-KB | 1448A-WT-KB | OK | 19.2174 | 45.2874 | 1.23669 | 0.696927 | 0.4215 | 0.647375 |
| ggt | gamma-glutamyltranspeptidase | chromosome:826412-827480 | 1448A-rhpS-KB | 1448A-WT-KB | OK | 80.1417 | 188.815 | 1.23635 | 2.01275 | 0.0075 | 0.056195 |
| flgH | flagellar basal body L-ring protein | chromosome:3935016-3935730 | 1448A-rhpS-KB | 1448A-WT-KB | NOTEST | 10.9809 | 25.8539 | 1.23539 | 0 | 1 | 1 |
| PSPPH_2364 | ribose ABC transporter permease | chromosome:2739811-2743357 | 1448A-rhpS-KB | 1448A-WT-KB | NOTEST | 8.31589 | 19.5725 | 1.23488 | 0 | 1 | 1 |
| PSPPH_3534 | major facilitator superfamily transporter | chromosome:4073384-4074584 | 1448A-rhpS-KB | 1448A-WT-KB | OK | 47.4357 | 111.634 | 1.23473 | 1.65536 | 0.02925 | 0.143009 |
| dapF | diaminopimelate epimerase | chromosome:5675005-5675836 | 1448A-rhpS-KB | 1448A-WT-KB | OK | 160.82 | 378.463 | 1.23471 | 2.38613 | 0.00215 | 0.0234271 |
| PSPPH_4854 | aldo/keto reductase | chromosome:5515510-5516548 | 1448A-rhpS-KB | 1448A-WT-KB | OK | 75.9564 | 178.526 | 1.23289 | 1.9586 | 0.01135 | 0.0762498 |
| PSPPH_A0021 | hypothetical protein | large_plasmid:18417-18705 | 1448A-rhpS-KB | 1448A-WT-KB | OK | 105.866 | 248.621 | 1.23171 | 0.852554 | 0.2294 | 0.466634 |
| PSPPH_2168 | hypothetical protein | chromosome:2522849-2524415 | 1448A-rhpS-KB | 1448A-WT-KB | NOTEST | 4.60581 | 10.8087 | 1.23066 | 0 | 1 | 1 |
| terC | tellurium resistance protein TerC | chromosome:998626-999661 | 1448A-rhpS-KB | 1448A-WT-KB | OK | 771.797 | 1809.38 | 1.2292 | 4.05302 | 0.00005 | 0.00121565 |
| PSPPH_2754 | achromobactin biosynthetic protein AcsC | chromosome:3189418-3192045 | 1448A-rhpS-KB | 1448A-WT-KB | NOTEST | 8.83433 | 20.6999 | 1.22843 | 0 | 1 | 1 |
| astD | succinylglutamic semialdehyde dehydrogenase | chromosome:4055967-4058459 | 1448A-rhpS-KB | 1448A-WT-KB | OK | 197.647 | 462.852 | 1.22763 | 2.71426 | 0.00025 | 0.0043799 |
| PSPPH_3612 | hypothetical protein | chromosome:4158161-4160266 | 1448A-rhpS-KB | 1448A-WT-KB | NOTEST | 6.96109 | 16.2304 | 1.22131 | 0 | 1 | 1 |
| gspF2 | general secretion pathway protein GspF | chromosome:2532397-2537908 | 1448A-rhpS-KB | 1448A-WT-KB | NOTEST | 6.53817 | 15.2405 | 1.22095 | 0 | 1 | 1 |
| PSPPH_2152 | hypothetical protein | chromosome:2506775-2507567 | 1448A-rhpS-KB | 1448A-WT-KB | NOTEST | 8.38244 | 19.5329 | 1.22047 | 0 | 1 | 1 |
| hrcU | type III secretion component protein HrcU | chromosome:1494961-1496832 | 1448A-rhpS-KB | 1448A-WT-KB | NOTEST | 3.4526 | 8.02719 | 1.21721 | 0 | 1 | 1 |
| PSPPH_4552 | phosphopantetheine attachment site domain-containing protein | chromosome:5191135-5191360 | 1448A-rhpS-KB | 1448A-WT-KB | OK | 130.62 | 303.293 | 1.21534 | 1.08282 | 0.3204 | 0.558318 |
| PSPPH_2879 | short chain dehydrogenase/reductase oxidoreductase | chromosome:3324194-3326814 | 1448A-rhpS-KB | 1448A-WT-KB | NOTEST | 15.5102 | 35.9674 | 1.21347 | 0 | 1 | 1 |
| PSPPH_4316 | hypothetical protein | chromosome:4929003-4930050 | 1448A-rhpS-KB | 1448A-WT-KB | OK | 315.542 | 731.598 | 1.21322 | 3.05745 | 0.00005 | 0.00121565 |
| pcaH | protocatechuate 3,4-dioxygenase subunit beta | chromosome:2455004-2455724 | 1448A-rhpS-KB | 1448A-WT-KB | OK | 34.4297 | 79.6986 | 1.2109 | 1.09516 | 0.12125 | 0.329793 |
| PSPPH_4858 | LacI family transcriptional regulator | chromosome:5521525-5522572 | 1448A-rhpS-KB | 1448A-WT-KB | NOTEST | 16.1297 | 37.3309 | 1.21066 | 0 | 1 | 1 |
| PSPPH_4666 | aldehyde dehydrogenase | chromosome:5301863-5303384 | 1448A-rhpS-KB | 1448A-WT-KB | OK | 169.565 | 392.264 | 1.20999 | 3.05549 | 0.00005 | 0.00121565 |
| hutC | histidine utilization repressor | chromosome:396061-397404 | 1448A-rhpS-KB | 1448A-WT-KB | OK | 130.064 | 300.486 | 1.20808 | 1.43567 | 0.09355 | 0.283106 |
| PSPPH_3283 | sulfate permease | chromosome:3804974-3806420 | 1448A-rhpS-KB | 1448A-WT-KB | OK | 38.4355 | 88.7151 | 1.20674 | 1.61174 | 0.0359 | 0.165344 |
| sodA | superoxide dismutase | chromosome:4738730-4739342 | 1448A-rhpS-KB | 1448A-WT-KB | NOTEST | 7.32746 | 16.908 | 1.20632 | 0 | 1 | 1 |
| PSPPH_4042 | insecticidal toxin protein | chromosome:4617998-4619348 | 1448A-rhpS-KB | 1448A-WT-KB | OK | 24.6184 | 56.8041 | 1.20626 | 1.26621 | 0.0922 | 0.281685 |
| PSPPH_2413 | FAD-binding oxidoreductase | chromosome:2789401-2791524 | 1448A-rhpS-KB | 1448A-WT-KB | NOTEST | 5.77788 | 13.3137 | 1.2043 | 0 | 1 | 1 |
| PSPPH_3594 | hypothetical protein | chromosome:4139616-4140270 | 1448A-rhpS-KB | 1448A-WT-KB | OK | 151.043 | 347.6 | 1.20246 | 1.94105 | 0.01285 | 0.0836537 |
| ureD | urease accessory protein UreD | chromosome:5111609-5112455 | 1448A-rhpS-KB | 1448A-WT-KB | OK | 25.8203 | 59.3816 | 1.20151 | 1.01601 | 0.1567 | 0.379299 |
| PSPPH_0412 | 3-oxoacyl-ACP synthase | chromosome:476539-481262 | 1448A-rhpS-KB | 1448A-WT-KB | OK | 51.7462 | 118.96 | 1.20095 | 1.54031 | 0.0899 | 0.279064 |
| iucD | siderophore biosynthesis protein | chromosome:4304724-4306089 | 1448A-rhpS-KB | 1448A-WT-KB | OK | 33.1443 | 76.1146 | 1.19941 | 1.46191 | 0.05035 | 0.204693 |
| bioF | 8-amino-7-oxononanoate synthase | chromosome:5355250-5357967 | 1448A-rhpS-KB | 1448A-WT-KB | OK | 179.805 | 412.89 | 1.19932 | 2.36635 | 0.0022 | 0.0238991 |
| PSPPH_A0051 | hypothetical protein | large_plasmid:42802-43464 | 1448A-rhpS-KB | 1448A-WT-KB | OK | 225.859 | 517.961 | 1.19743 | 1.04247 | 0.16465 | 0.392283 |
| PSPPH_4464 | hypothetical protein | chromosome:5098815-5099274 | 1448A-rhpS-KB | 1448A-WT-KB | NOTEST | 10.3529 | 23.7252 | 1.19639 | 0 | 1 | 1 |
| rsmC | ribosomal RNA small subunit methyltransferase C | chromosome:1225013-1226012 | 1448A-rhpS-KB | 1448A-WT-KB | OK | 48.1805 | 110.394 | 1.19614 | 1.46865 | 0.04475 | 0.190628 |
| rluA | ribosomal large subunit pseudouridine synthase A | chromosome:1865080-1865794 | 1448A-rhpS-KB | 1448A-WT-KB | OK | 64.7406 | 148.24 | 1.19519 | 1.46877 | 0.0472 | 0.197258 |
| PSPPH_4727 | ABC transporter permease | chromosome:5363953-5368805 | 1448A-rhpS-KB | 1448A-WT-KB | NOTEST | 11.4802 | 26.2297 | 1.19206 | 0 | 1 | 1 |
| cheW1 | chemotaxis protein CheW | chromosome:947004-947544 | 1448A-rhpS-KB | 1448A-WT-KB | OK | 25.5343 | 58.2017 | 1.18863 | 0.76867 | 0.28935 | 0.52505 |
| fmt | methionyl-tRNA formyltransferase | chromosome:22798-26456 | 1448A-rhpS-KB | 1448A-WT-KB | OK | 195.591 | 445.111 | 1.18633 | 2.21748 | 0.0026 | 0.0270915 |
| lon2 | ATP-dependent protease La | chromosome:4503115-4505533 | 1448A-rhpS-KB | 1448A-WT-KB | OK | 721.046 | 1639.55 | 1.18514 | 4.63411 | 0.00005 | 0.00121565 |
| PSPPH_1051 | ABC transporter ATP-binding protein | chromosome:1241701-1246332 | 1448A-rhpS-KB | 1448A-WT-KB | NOTEST | 7.33257 | 16.6483 | 1.18298 | 0 | 1 | 1 |
| ohr | organic hydroperoxide resistance protein | chromosome:4190623-4191136 | 1448A-rhpS-KB | 1448A-WT-KB | OK | 47.1528 | 107.01 | 1.18234 | 1.00692 | 0.16955 | 0.396838 |
| ccoP | cytochrome c oxidase, cbb3-type subunit III | chromosome:3868112-3869278 | 1448A-rhpS-KB | 1448A-WT-KB | OK | 44.2367 | 100.392 | 1.18232 | 1.33735 | 0.0953 | 0.28622 |
| hom | homoserine dehydrogenase | chromosome:1575672-1576977 | 1448A-rhpS-KB | 1448A-WT-KB | OK | 438.779 | 995.703 | 1.18222 | 3.71459 | 0.00005 | 0.00121565 |
| PSPPH_4876 | HAD family hydrolase | chromosome:5545897-5546554 | 1448A-rhpS-KB | 1448A-WT-KB | OK | 157.636 | 357.446 | 1.18113 | 1.99483 | 0.0101 | 0.070092 |
| PSPPH_4467 | hypothetical protein | chromosome:5100412-5101786 | 1448A-rhpS-KB | 1448A-WT-KB | NOTEST | 18.4015 | 41.6595 | 1.17882 | 0 | 1 | 1 |
| PSPPH_4262 | peptide ABC transporter substrate-binding protein | chromosome:4865565-4867161 | 1448A-rhpS-KB | 1448A-WT-KB | OK | 166.842 | 377.623 | 1.17847 | 3.13741 | 0.00015 | 0.00302881 |
| soxA | sarcosine oxidase subunit alpha | chromosome:5395145-5398459 | 1448A-rhpS-KB | 1448A-WT-KB | NOTEST | 9.05468 | 20.4843 | 1.17778 | 0 | 1 | 1 |
| PSPPH_3067 | hypothetical protein | chromosome:3562753-3563914 | 1448A-rhpS-KB | 1448A-WT-KB | NOTEST | 17.181 | 38.8564 | 1.17734 | 0 | 1 | 1 |
| PSPPH_0531 | FAD-binding oxidoreductase | chromosome:623935-625111 | 1448A-rhpS-KB | 1448A-WT-KB | OK | 212.202 | 479.85 | 1.17715 | 2.85498 | 0.00005 | 0.00121565 |
| terD | tellurium resistance protein TerD | chromosome:999688-1000264 | 1448A-rhpS-KB | 1448A-WT-KB | OK | 819.558 | 1852.69 | 1.1767 | 3.26358 | 0.00005 | 0.00121565 |
| PSPPH_3615 | acyl-CoA dehydrogenase | chromosome:4161344-4162538 | 1448A-rhpS-KB | 1448A-WT-KB | NOTEST | 6.65295 | 15.0022 | 1.17311 | 0 | 1 | 1 |
| PSPPH_4556 | hypothetical protein | chromosome:5195903-5198625 | 1448A-rhpS-KB | 1448A-WT-KB | OK | 80.7454 | 182.015 | 1.17261 | 1.32168 | 0.07155 | 0.247072 |
| PSPPH_4551 | 3-oxoacyl-ACP synthase | chromosome:5188639-5191134 | 1448A-rhpS-KB | 1448A-WT-KB | OK | 98.9175 | 222.627 | 1.17033 | 1.55663 | 0.0404 | 0.17782 |
| PSPPH_2920 | GTP cyclohydrolase | chromosome:3391967-3392873 | 1448A-rhpS-KB | 1448A-WT-KB | NOTEST | 16.934 | 38.0819 | 1.16918 | 0 | 1 | 1 |
| PSPPH_1918 | hypothetical protein | chromosome:2235277-2237935 | 1448A-rhpS-KB | 1448A-WT-KB | NOTEST | 6.72968 | 15.1327 | 1.16906 | 0 | 1 | 1 |
| PSPPH_1080 | hypothetical protein | chromosome:1269056-1273144 | 1448A-rhpS-KB | 1448A-WT-KB | NOTEST | 11.4991 | 25.7734 | 1.16437 | 0 | 1 | 1 |
| grpE | heat shock protein GrpE | chromosome:4797241-4797805 | 1448A-rhpS-KB | 1448A-WT-KB | OK | 2165.9 | 4847.96 | 1.16241 | 4.04322 | 0.00005 | 0.00121565 |
| PSPPH_2915 | glutamine amidotransferase, class-II protein | chromosome:3386173-3387774 | 1448A-rhpS-KB | 1448A-WT-KB | NOTEST | 13.0934 | 29.2944 | 1.16179 | 0 | 1 | 1 |
| PSPPH_0342 | cystine transporter subunit | chromosome:388975-390438 | 1448A-rhpS-KB | 1448A-WT-KB | OK | 222.762 | 497.82 | 1.16012 | 2.34853 | 0.00225 | 0.0242214 |
| PSPPH_2966 | methyl-accepting chemotaxis protein | chromosome:3442545-3444486 | 1448A-rhpS-KB | 1448A-WT-KB | NOTEST | 13.5732 | 30.3316 | 1.16006 | 0 | 1 | 1 |
| PSPPH_0919 | MltA domain-containing protein | chromosome:1093606-1094809 | 1448A-rhpS-KB | 1448A-WT-KB | OK | 103.637 | 231.561 | 1.15986 | 2.1617 | 0.00485 | 0.0417684 |
| PSPPH_2366 | acyl-CoA dehydrogenase | chromosome:2743366-2744605 | 1448A-rhpS-KB | 1448A-WT-KB | NOTEST | 16.466 | 36.7774 | 1.15933 | 0 | 1 | 1 |
| PSPPH_2673 | ABC transporter permease | chromosome:3094176-3094983 | 1448A-rhpS-KB | 1448A-WT-KB | NOTEST | 4.82714 | 10.7709 | 1.1579 | 0 | 1 | 1 |
| PSPPH_3700 | cobalamin biosynthesis protein | chromosome:4243474-4248776 | 1448A-rhpS-KB | 1448A-WT-KB | OK | 26.9762 | 60.1633 | 1.15719 | 0.655551 | 0.36025 | 0.594704 |
| flgG | flagellar basal body rod protein FlgG | chromosome:3935814-3936603 | 1448A-rhpS-KB | 1448A-WT-KB | NOTEST | 16.4301 | 36.5717 | 1.15439 | 0 | 1 | 1 |
| hopAS1 | type III effector HopAS1 | chromosome:5376067-5380153 | 1448A-rhpS-KB | 1448A-WT-KB | NOTEST | 7.8599 | 17.4848 | 1.15352 | 0 | 1 | 1 |
| PSPPH_0116 | lipoprotein | chromosome:133107-134130 | 1448A-rhpS-KB | 1448A-WT-KB | OK | 46.7006 | 103.788 | 1.15212 | 1.40129 | 0.05645 | 0.216705 |
| PSPPH_0145 | hypothetical protein | chromosome:171111-174771 | 1448A-rhpS-KB | 1448A-WT-KB | OK | 91.5192 | 203.182 | 1.15063 | 0.981521 | 0.17545 | 0.404554 |
| PSPPH_0215 | aldehyde dehydrogenase | chromosome:251625-253119 | 1448A-rhpS-KB | 1448A-WT-KB | OK | 597.842 | 1325.87 | 1.1491 | 3.97172 | 0.00005 | 0.00121565 |
| PSPPH_3190 | iolL protein | chromosome:3700338-3701169 | 1448A-rhpS-KB | 1448A-WT-KB | NOTEST | 9.66447 | 21.4212 | 1.14828 | 0 | 1 | 1 |
| fcs | feruloyl-CoA synthase | chromosome:2816953-2819964 | 1448A-rhpS-KB | 1448A-WT-KB | NOTEST | 10.3747 | 22.983 | 1.14749 | 0 | 1 | 1 |
| PSPPH_4317 | hypothetical protein | chromosome:4930055-4931276 | 1448A-rhpS-KB | 1448A-WT-KB | OK | 272.168 | 601.959 | 1.14517 | 3.01779 | 0.0001 | 0.00221988 |
| PSPPH_0552 | bifunctional thiosulfate sulfurtransferase/phosphatidylserine decarboxylase | chromosome:648638-650471 | 1448A-rhpS-KB | 1448A-WT-KB | OK | 169.143 | 374.036 | 1.14493 | 3.06357 | 0.00005 | 0.00121565 |
| PSPPH_3342 | prevent-host-death family protein | chromosome:3871374-3872186 | 1448A-rhpS-KB | 1448A-WT-KB | OK | 64.7174 | 143.046 | 1.14425 | 0.794097 | 0.38325 | 0.615772 |
| PSPPH_4788 | TetR family transcriptional regulator | chromosome:5434179-5434842 | 1448A-rhpS-KB | 1448A-WT-KB | OK | 51.8051 | 114.443 | 1.14347 | 1.13406 | 0.11305 | 0.316323 |
| PSPPH_B0002 | replicase family protein | small_plasmid:133-1617 | 1448A-rhpS-KB | 1448A-WT-KB | NOTEST | 4.81933 | 10.6297 | 1.1412 | 0 | 1 | 1 |
| PSPPH_1749 | non-ribosomal peptide synthetase | chromosome:2029073-2051370 | 1448A-rhpS-KB | 1448A-WT-KB | OK | 24.9247 | 54.7988 | 1.13657 | 2.26528 | 0.0036 | 0.0336817 |
| PSPPH_2493 | hypothetical protein | chromosome:2879843-2880389 | 1448A-rhpS-KB | 1448A-WT-KB | OK | 319.882 | 702.999 | 1.13599 | 2.20217 | 0.0042 | 0.0376211 |
| PSPPH_2392 | S24 family peptidase | chromosome:2771396-2772119 | 1448A-rhpS-KB | 1448A-WT-KB | OK | 193.738 | 425.675 | 1.13564 | 2.08303 | 0.00655 | 0.0510015 |
| PSPPH_3903 | amino acid ABC transporter permease | chromosome:4462755-4464898 | 1448A-rhpS-KB | 1448A-WT-KB | OK | 53.1816 | 116.787 | 1.13489 | 0.709895 | 0.2676 | 0.50308 |
| PSPPH_0127 | hypothetical protein | chromosome:148074-150880 | 1448A-rhpS-KB | 1448A-WT-KB | OK | 111.498 | 244.443 | 1.13248 | 1.60641 | 0.0527 | 0.209045 |
| hopI1 | type III effector HopI1 | chromosome:4988660-4989671 | 1448A-rhpS-KB | 1448A-WT-KB | NOTEST | 16.7774 | 36.7217 | 1.13011 | 0 | 1 | 1 |
| PSPPH_2501 | cation ABC transporter ATP-binding protein | chromosome:2887659-2889400 | 1448A-rhpS-KB | 1448A-WT-KB | NOTEST | 11.7337 | 25.6419 | 1.12785 | 0 | 1 | 1 |
| PSPPH_4541 | hypothetical protein | chromosome:5176816-5178747 | 1448A-rhpS-KB | 1448A-WT-KB | NOTEST | 17.9178 | 39.1516 | 1.12768 | 0 | 1 | 1 |
| trpA | tryptophan synthase subunit alpha | chromosome:40541-42580 | 1448A-rhpS-KB | 1448A-WT-KB | OK | 94.9208 | 206.988 | 1.12475 | 1.45073 | 0.04925 | 0.202089 |
| PSPPH_4944 | DnaJ domain-containing protein | chromosome:5617412-5618609 | 1448A-rhpS-KB | 1448A-WT-KB | NOTEST | 9.79781 | 21.3387 | 1.12294 | 0 | 1 | 1 |
| PSPPH_1558 | oligopeptidase B | chromosome:1809064-1811125 | 1448A-rhpS-KB | 1448A-WT-KB | OK | 101.255 | 220.35 | 1.1218 | 2.63188 | 0.00055 | 0.0080893 |
| PSPPH_2269 | transcriptional regulator | chromosome:2631474-2631837 | 1448A-rhpS-KB | 1448A-WT-KB | OK | 276.173 | 600.857 | 1.12145 | 1.56503 | 0.04005 | 0.177156 |
| PSPPH_2614 | SCP-2 sterol transfer family protein | chromosome:3013224-3013542 | 1448A-rhpS-KB | 1448A-WT-KB | OK | 753.646 | 1638.86 | 1.12074 | 2.13106 | 0.00565 | 0.0458934 |
| PSPPH_4008 | hypothetical protein | chromosome:4579267-4579891 | 1448A-rhpS-KB | 1448A-WT-KB | OK | 94.1198 | 204.623 | 1.1204 | 1.5379 | 0.05785 | 0.220893 |
| PSPPH_4052 | beta-lactamase | chromosome:4633842-4637746 | 1448A-rhpS-KB | 1448A-WT-KB | NOTEST | 16.4107 | 35.6431 | 1.11899 | 0 | 1 | 1 |
| PSPPH_4769 | hypothetical protein | chromosome:5414454-5417215 | 1448A-rhpS-KB | 1448A-WT-KB | OK | 38.4532 | 83.463 | 1.11803 | 1.2687 | 0.09585 | 0.286668 |
| pcaG | protocatechuate 3,4-dioxygenase subunit alpha | chromosome:2455735-2456338 | 1448A-rhpS-KB | 1448A-WT-KB | OK | 28.8155 | 62.5224 | 1.11753 | 0.762779 | 0.2846 | 0.520819 |
| rbsC | ribose ABC transporter permease | chromosome:2483081-2485624 | 1448A-rhpS-KB | 1448A-WT-KB | OK | 54.8558 | 118.917 | 1.11624 | 1.29032 | 0.07585 | 0.257933 |
| PSPPH_0099 | shufflon-specific recombinase | chromosome:109723-110797 | 1448A-rhpS-KB | 1448A-WT-KB | NOTEST | 12.1594 | 26.3108 | 1.11359 | 0 | 1 | 1 |
| mdaB | NAD(P)H quinone dehydrogenase MdaB | chromosome:834143-834731 | 1448A-rhpS-KB | 1448A-WT-KB | OK | 22.6935 | 49.1036 | 1.11355 | 0.748573 | 0.36315 | 0.59797 |
| PSPPH_0134 | hypothetical protein | chromosome:158396-158930 | 1448A-rhpS-KB | 1448A-WT-KB | OK | 503.097 | 1087.19 | 1.1117 | 2.54208 | 0.0008 | 0.0107489 |
| PSPPH_4621 | xanthine/uracil permease | chromosome:5256989-5258387 | 1448A-rhpS-KB | 1448A-WT-KB | NOTEST | 7.60965 | 16.4315 | 1.11056 | 0 | 1 | 1 |
| PSPPH_3320 | alanine racemase | chromosome:3841513-3843029 | 1448A-rhpS-KB | 1448A-WT-KB | NOTEST | 5.88362 | 12.6793 | 1.10769 | 0 | 1 | 1 |
| PSPPH_0583 | transglutaminase | chromosome:686419-689698 | 1448A-rhpS-KB | 1448A-WT-KB | OK | 36.9735 | 79.6547 | 1.10727 | 2.12792 | 0.0056 | 0.0457995 |
| PSPPH_4069 | lipoprotein | chromosome:4652254-4653319 | 1448A-rhpS-KB | 1448A-WT-KB | OK | 66.5473 | 143.324 | 1.10683 | 1.67498 | 0.0275 | 0.136697 |
| PSPPH_1004 | hypothetical protein | chromosome:1195847-1202294 | 1448A-rhpS-KB | 1448A-WT-KB | OK | 27.0072 | 58.0452 | 1.10383 | 0.462407 | 0.5414 | 0.738818 |
| PSPPH_1432 | ABC transporter ATP-binding protein/permease | chromosome:1670745-1672578 | 1448A-rhpS-KB | 1448A-WT-KB | OK | 42.8196 | 92.0077 | 1.10348 | 1.77151 | 0.01605 | 0.0967331 |
| PSPPH_0515 | lipopolysaccharide biosynthesis protein | chromosome:597387-603334 | 1448A-rhpS-KB | 1448A-WT-KB | OK | 80.4024 | 172.678 | 1.10277 | 1.01696 | 0.1636 | 0.391085 |
| hflC | HflC protein | chromosome:666074-668143 | 1448A-rhpS-KB | 1448A-WT-KB | OK | 565.562 | 1214.25 | 1.10231 | 2.05594 | 0.00415 | 0.0374548 |
| pyrC | dihydroorotase | chromosome:535568-537841 | 1448A-rhpS-KB | 1448A-WT-KB | OK | 335.198 | 718.744 | 1.10046 | 2.61037 | 0.00085 | 0.01121 |
| PSPPH_5126 | aldose 1-epimerase | chromosome:5815217-5816129 | 1448A-rhpS-KB | 1448A-WT-KB | OK | 112.848 | 241.695 | 1.0988 | 1.86954 | 0.0133 | 0.0853397 |
| PSPPH_2601 | sensor histidine kinase/response regulator | chromosome:2996887-3000400 | 1448A-rhpS-KB | 1448A-WT-KB | OK | 79.633 | 170.428 | 1.09773 | 2.80081 | 0.0002 | 0.00374241 |
| rbsB | ribose ABC transporter substrate-binding protein | chromosome:2482068-2483028 | 1448A-rhpS-KB | 1448A-WT-KB | OK | 47.5036 | 101.558 | 1.0962 | 1.30201 | 0.0771 | 0.258737 |
| PSPPH_0344 | serine O-acetyltransferase | chromosome:391853-392807 | 1448A-rhpS-KB | 1448A-WT-KB | OK | 173 | 369.391 | 1.09438 | 2.21782 | 0.0035 | 0.0330053 |
| PSPPH_1488 | hypothetical protein | chromosome:1729730-1730603 | 1448A-rhpS-KB | 1448A-WT-KB | NOTEST | 17.7483 | 37.8947 | 1.09431 | 0 | 1 | 1 |
| PSPPH_1256 | transketolase | chromosome:1461360-1463225 | 1448A-rhpS-KB | 1448A-WT-KB | NOTEST | 13.9465 | 29.7541 | 1.09319 | 0 | 1 | 1 |
| PSPPH_0419 | hypothetical protein | chromosome:482647-490911 | 1448A-rhpS-KB | 1448A-WT-KB | OK | 29.3147 | 62.5193 | 1.09268 | 1.31472 | 0.0658 | 0.2347 |
| PSPPH_4635 | 2OG-Fe(II) oxygenase | chromosome:5272858-5273824 | 1448A-rhpS-KB | 1448A-WT-KB | OK | 27.1998 | 57.9341 | 1.09082 | 1.04868 | 0.13355 | 0.347892 |
| PSPPH_0905 | diguanylate cyclase | chromosome:1077997-1079101 | 1448A-rhpS-KB | 1448A-WT-KB | NOTEST | 6.86817 | 14.6274 | 1.09068 | 0 | 1 | 1 |
| recA | recombinase A | chromosome:4355400-4356465 | 1448A-rhpS-KB | 1448A-WT-KB | OK | 377.022 | 802.8 | 1.09039 | 3.06464 | 0.00015 | 0.00302881 |
| arnA | bifunctional UDP-glucuronic acid decarboxylase/UDP-4-amino-4-deoxy-L-arabinose formyltransferase | chromosome:3246757-3252777 | 1448A-rhpS-KB | 1448A-WT-KB | NOTEST | 6.79519 | 14.4635 | 1.08983 | 0 | 1 | 1 |
| PSPPH_0639 | DnaJ-like protein DjlA | chromosome:756193-756961 | 1448A-rhpS-KB | 1448A-WT-KB | OK | 65.9617 | 140.256 | 1.08837 | 1.31276 | 0.0709 | 0.245539 |
| maiA | maleylacetoacetate isomerase | chromosome:3768870-3769506 | 1448A-rhpS-KB | 1448A-WT-KB | OK | 74.6353 | 158.506 | 1.0866 | 1.25154 | 0.09425 | 0.284501 |
| PSPPH_1024 | homocysteine S-methyltransferase | chromosome:1213568-1214465 | 1448A-rhpS-KB | 1448A-WT-KB | OK | 145.888 | 309.702 | 1.08602 | 1.97361 | 0.0085 | 0.0625082 |
| PSPPH_0133 | hypothetical protein | chromosome:156879-158382 | 1448A-rhpS-KB | 1448A-WT-KB | OK | 492.321 | 1045.14 | 1.08602 | 3.61309 | 0.00005 | 0.00121565 |
| ndh | NADH dehydrogenase | chromosome:953818-955117 | 1448A-rhpS-KB | 1448A-WT-KB | OK | 246.507 | 522.233 | 1.08306 | 2.87432 | 0.0003 | 0.0050338 |
| PSPPH_4713 | diguanylate cyclase | chromosome:5349076-5350375 | 1448A-rhpS-KB | 1448A-WT-KB | NOTEST | 2.81858 | 5.97019 | 1.08281 | 0 | 1 | 1 |
| fabG | 3-ketoacyl-ACP reductase | chromosome:476539-481262 | 1448A-rhpS-KB | 1448A-WT-KB | OK | 53.6849 | 113.651 | 1.08203 | 0.882468 | 0.28725 | 0.522725 |
| PSPPH_4260 | peptide ABC transporter substrate-binding protein | chromosome:4862319-4863912 | 1448A-rhpS-KB | 1448A-WT-KB | OK | 117.025 | 247.336 | 1.07966 | 2.45821 | 0.0012 | 0.014789 |
| hisH | imidazole glycerol phosphate synthase subunit HisH | chromosome:5596669-5598166 | 1448A-rhpS-KB | 1448A-WT-KB | OK | 296.368 | 626.176 | 1.07918 | 1.81075 | 0.014 | 0.0874755 |
| PSPPH_3084 | dsrH family protein | chromosome:3580488-3582480 | 1448A-rhpS-KB | 1448A-WT-KB | OK | 23.5226 | 49.6945 | 1.07904 | 0.39982 | 0.5554 | 0.750472 |
| uxuB/xylB | D-mannonate oxidoreductase/xylulokinase | chromosome:2986616-2989538 | 1448A-rhpS-KB | 1448A-WT-KB | OK | 141.812 | 299.533 | 1.07873 | 3.17903 | 0.00005 | 0.00121565 |
| secF | preprotein translocase subunit SecF | chromosome:1515293-1516205 | 1448A-rhpS-KB | 1448A-WT-KB | OK | 196.652 | 415.029 | 1.07757 | 2.23788 | 0.0035 | 0.0330053 |
| PSPPH_1897 | chorismate mutase | chromosome:2200418-2201546 | 1448A-rhpS-KB | 1448A-WT-KB | OK | 152.686 | 321.664 | 1.07499 | 2.23816 | 0.00325 | 0.0315639 |
| PSPPH_4472 | branched-chain amino acid ABC transporter ATP-binding protein | chromosome:5107236-5110762 | 1448A-rhpS-KB | 1448A-WT-KB | NOTEST | 13.7202 | 28.8969 | 1.07462 | 0 | 1 | 1 |
| PSPPH_4983 | prophage PSPPH06 tail tape meausure domain-containing protein | chromosome:5653456-5654695 | 1448A-rhpS-KB | 1448A-WT-KB | NOTEST | 20.4755 | 43.1161 | 1.07433 | 0 | 1 | 1 |
| recO | DNA repair protein RecO | chromosome:4508865-4509549 | 1448A-rhpS-KB | 1448A-WT-KB | OK | 29.8705 | 62.8831 | 1.07395 | 0.823251 | 0.2423 | 0.475813 |
| PSPPH_0425 | AMP-binding protein | chromosome:482647-490911 | 1448A-rhpS-KB | 1448A-WT-KB | OK | 24.4079 | 51.3468 | 1.07293 | 1.06973 | 0.13775 | 0.352664 |
| PSPPH_4293 | ABC transporter ATP-binding protein | chromosome:4899815-4901507 | 1448A-rhpS-KB | 1448A-WT-KB | OK | 165.411 | 347.577 | 1.07127 | 2.72472 | 0.0004 | 0.00632566 |
| PSPPH_0931 | aldose 1-epimerase | chromosome:1105430-1107258 | 1448A-rhpS-KB | 1448A-WT-KB | OK | 32.6544 | 68.5875 | 1.07067 | 1.04116 | 0.3702 | 0.602746 |
| pobA | 4-hydroxybenzoate 3-monooxygenase | chromosome:3967362-3968547 | 1448A-rhpS-KB | 1448A-WT-KB | OK | 38.3147 | 80.4507 | 1.07021 | 1.28808 | 0.07615 | 0.258404 |
| ilvD | dihydroxy-acid dehydratase | chromosome:523789-525637 | 1448A-rhpS-KB | 1448A-WT-KB | OK | 127.181 | 266.652 | 1.06807 | 2.58762 | 0.00055 | 0.0080893 |
| PSPPH_1001 | hypothetical protein | chromosome:1194578-1195097 | 1448A-rhpS-KB | 1448A-WT-KB | OK | 741.631 | 1554.11 | 1.06732 | 2.73478 | 0.0006 | 0.00875265 |
| PSPPH_3742 | hypothetical protein | chromosome:4291494-4292421 | 1448A-rhpS-KB | 1448A-WT-KB | OK | 22.0869 | 46.2748 | 1.06703 | 0.852486 | 0.2101 | 0.446697 |
| PSPPH_2808 | hypothetical protein | chromosome:3253183-3254969 | 1448A-rhpS-KB | 1448A-WT-KB | NOTEST | 13.312 | 27.8555 | 1.06524 | 0 | 1 | 1 |
| PSPPH_2399 | hypothetical protein | chromosome:2776591-2778045 | 1448A-rhpS-KB | 1448A-WT-KB | NOTEST | 8.77802 | 18.3674 | 1.06518 | 0 | 1 | 1 |
| rnk | nucleoside diphosphate kinase regulator | chromosome:5678185-5678593 | 1448A-rhpS-KB | 1448A-WT-KB | OK | 92.8532 | 194.213 | 1.06462 | 1.18161 | 0.18355 | 0.412402 |
| PSPPH_3129 | lipoprotein | chromosome:3628139-3630740 | 1448A-rhpS-KB | 1448A-WT-KB | OK | 22.2727 | 46.5787 | 1.06439 | 0.396528 | 0.56585 | 0.75715 |
| PSPPH_4638 | LysR family transcriptional regulator | chromosome:5276250-5277174 | 1448A-rhpS-KB | 1448A-WT-KB | NOTEST | 18.6921 | 39.0573 | 1.06316 | 0 | 1 | 1 |
| PSPPH_2154 | prophage PSPPH03, host specificity protein J, truncated | chromosome:2508585-2510475 | 1448A-rhpS-KB | 1448A-WT-KB | NOTEST | 3.963 | 8.27003 | 1.0613 | 0 | 1 | 1 |
| PSPPH_0316 | hypothetical protein | chromosome:358746-359364 | 1448A-rhpS-KB | 1448A-WT-KB | OK | 58.3773 | 121.743 | 1.06036 | 1.0422 | 0.14095 | 0.356263 |
| PSPPH_2781 | TspO/MBR family protein | chromosome:3218608-3219049 | 1448A-rhpS-KB | 1448A-WT-KB | NOTEST | 11.5338 | 24.0417 | 1.05967 | 0 | 1 | 1 |
| tas | aldo/keto reductase | chromosome:3326881-3327919 | 1448A-rhpS-KB | 1448A-WT-KB | OK | 284.429 | 592.239 | 1.05811 | 2.71486 | 0.0007 | 0.00962231 |
| PSPPH_1453 | Rhs family protein | chromosome:1694237-1695928 | 1448A-rhpS-KB | 1448A-WT-KB | NOTEST | 16.1276 | 33.5684 | 1.05757 | 0 | 1 | 1 |
| PSPPH_2832 | IclR family transcriptional regulator | chromosome:3278768-3279608 | 1448A-rhpS-KB | 1448A-WT-KB | OK | 31.4549 | 65.4164 | 1.05637 | 0.959912 | 0.1754 | 0.404554 |
| PSPPH_0123 | OmpA domain-containing protein | chromosome:140679-144092 | 1448A-rhpS-KB | 1448A-WT-KB | OK | 41.8327 | 86.9907 | 1.05623 | 1.71537 | 0.0197 | 0.10899 |
| PSPPH_0883 | pectate lyase L | chromosome:1054118-1055243 | 1448A-rhpS-KB | 1448A-WT-KB | NOTEST | 11.2498 | 23.3069 | 1.05086 | 0 | 1 | 1 |
| PSPPH_0337 | acyl-CoA dehydrogenase | chromosome:383836-385159 | 1448A-rhpS-KB | 1448A-WT-KB | NOTEST | 4.50605 | 9.33359 | 1.05057 | 0 | 1 | 1 |
| PSPPH_3923 | PadR family transcriptional regulator | chromosome:4482885-4484240 | 1448A-rhpS-KB | 1448A-WT-KB | OK | 36.5743 | 75.6809 | 1.0491 | 0.682711 | 0.29075 | 0.525881 |
| PSPPH_0494 | hypothetical protein | chromosome:566940-569586 | 1448A-rhpS-KB | 1448A-WT-KB | NOTEST | 12.6007 | 26.0287 | 1.0466 | 0 | 1 | 1 |
| PSPPH_3242 | cyanate transport protein | chromosome:3756663-3757890 | 1448A-rhpS-KB | 1448A-WT-KB | NOTEST | 17.0525 | 35.1835 | 1.04491 | 0 | 1 | 1 |
| PSPPH_0560 | hypothetical protein | chromosome:658015-659506 | 1448A-rhpS-KB | 1448A-WT-KB | OK | 71.0148 | 146.473 | 1.04444 | 1.76505 | 0.02075 | 0.11305 |
| lexA2 | LexA repressor | chromosome:3714292-3714901 | 1448A-rhpS-KB | 1448A-WT-KB | OK | 87.9216 | 181.088 | 1.0424 | 1.27162 | 0.0826 | 0.266279 |
| trpB | tryptophan synthase subunit beta | chromosome:40541-42580 | 1448A-rhpS-KB | 1448A-WT-KB | OK | 136.245 | 280.417 | 1.04137 | 2.08951 | 0.00425 | 0.0376911 |
| PSPPH_4787 | rhodanese domain-containing protein | chromosome:5433267-5434125 | 1448A-rhpS-KB | 1448A-WT-KB | OK | 253.545 | 521.207 | 1.03962 | 2.30613 | 0.00245 | 0.0257538 |
| iolG | myo-inositol 2-dehydrogenase | chromosome:3697264-3698281 | 1448A-rhpS-KB | 1448A-WT-KB | NOTEST | 10.0029 | 20.5527 | 1.03891 | 0 | 1 | 1 |
| PSPPH_0050 | hexapeptide repeat-containing transferase | chromosome:52201-52747 | 1448A-rhpS-KB | 1448A-WT-KB | OK | 122.561 | 251.766 | 1.03859 | 1.40112 | 0.0594 | 0.223705 |
| glyQ | glycyl-tRNA synthetase subunit alpha | chromosome:14353-17412 | 1448A-rhpS-KB | 1448A-WT-KB | OK | 408.251 | 837.727 | 1.03702 | 2.29331 | 0.0014 | 0.0168471 |
| PSPPH_1750 | non-ribosomal peptide synthetase | chromosome:2029073-2051370 | 1448A-rhpS-KB | 1448A-WT-KB | NOTEST | 18.0963 | 37.0932 | 1.03546 | 0 | 1 | 1 |
| PSPPH_4258 | peptide ABC transporter substrate-binding protein | chromosome:4857642-4860100 | 1448A-rhpS-KB | 1448A-WT-KB | OK | 136.724 | 279.799 | 1.03313 | 2.39697 | 0.002 | 0.0226921 |
| PSPPH_2293 | nickel ABC transporter substrate-binding protein | chromosome:2658194-2660773 | 1448A-rhpS-KB | 1448A-WT-KB | NOTEST | 8.30438 | 16.9781 | 1.03173 | 0 | 1 | 1 |
| PSPPH_2786 | ABC transporter substrate-binding protein | chromosome:3224066-3225958 | 1448A-rhpS-KB | 1448A-WT-KB | NOTEST | 7.02361 | 14.3385 | 1.02961 | 0 | 1 | 1 |
| PSPPH_3935 | hypothetical protein | chromosome:4495715-4497089 | 1448A-rhpS-KB | 1448A-WT-KB | NOTEST | 7.10693 | 14.5013 | 1.02888 | 0 | 1 | 1 |
| ampC | beta-lactamase | chromosome:3806564-3808817 | 1448A-rhpS-KB | 1448A-WT-KB | OK | 23.6158 | 48.1802 | 1.02869 | 0.950236 | 0.18345 | 0.412402 |
| PSPPH_0017 | oxidoreductase alpha (molybdopterin) subunit, fusion | chromosome:21015-22644 | 1448A-rhpS-KB | 1448A-WT-KB | NOTEST | 3.71136 | 7.56791 | 1.02795 | 0 | 1 | 1 |
| mqo1 | malate:quinone oxidoreductase | chromosome:1214567-1216076 | 1448A-rhpS-KB | 1448A-WT-KB | OK | 648.957 | 1322.64 | 1.02723 | 3.56692 | 0.00005 | 0.00121565 |
| PSPPH_4030 | hypothetical protein | chromosome:4602130-4606623 | 1448A-rhpS-KB | 1448A-WT-KB | NOTEST | 13.3446 | 27.1976 | 1.02722 | 0 | 1 | 1 |
| PSPPH_0136 | hypothetical protein | chromosome:160912-161734 | 1448A-rhpS-KB | 1448A-WT-KB | NOTEST | 15.685 | 31.9141 | 1.02481 | 0 | 1 | 1 |
| PSPPH_A0103 | addiction module antitoxin | large_plasmid:89934-90500 | 1448A-rhpS-KB | 1448A-WT-KB | OK | 360.707 | 731.55 | 1.02013 | 1.09816 | 0.3259 | 0.56322 |
| PSPPH_0138 | hypothetical protein | chromosome:163895-164447 | 1448A-rhpS-KB | 1448A-WT-KB | OK | 302.828 | 612.029 | 1.0151 | 1.80476 | 0.01275 | 0.0831542 |
| rbsA | ribose ABC transporter ATP-binding protein | chromosome:2483081-2485624 | 1448A-rhpS-KB | 1448A-WT-KB | OK | 45.7595 | 92.3668 | 1.0133 | 1.42394 | 0.04865 | 0.200703 |
| PSPPH_0057 | ABC transporter ATP-binding protein | chromosome:59094-61050 | 1448A-rhpS-KB | 1448A-WT-KB | OK | 111.491 | 224.84 | 1.01198 | 2.39769 | 0.00195 | 0.0221952 |
| xylC | benzaldehyde dehydrogenase | chromosome:2806909-2808391 | 1448A-rhpS-KB | 1448A-WT-KB | NOTEST | 11.8326 | 23.8558 | 1.01157 | 0 | 1 | 1 |
| hopAN1 | HopAN1 protein | chromosome:518542-520470 | 1448A-rhpS-KB | 1448A-WT-KB | NOTEST | 13.6399 | 27.4937 | 1.01127 | 0 | 1 | 1 |
| gap2 | glyceraldehyde-3-phosphate dehydrogenase | chromosome:2151801-2153265 | 1448A-rhpS-KB | 1448A-WT-KB | OK | 232.012 | 467.018 | 1.00928 | 2.70822 | 0.00035 | 0.00566018 |
| PSPPH_4857 | TonB-dependent receptor | chromosome:5519037-5521353 | 1448A-rhpS-KB | 1448A-WT-KB | NOTEST | 6.59809 | 13.2811 | 1.00925 | 0 | 1 | 1 |
| PSPPH_5039 | D-isomer specific 2-hydroxyacid dehydrogenase | chromosome:5710432-5711398 | 1448A-rhpS-KB | 1448A-WT-KB | OK | 95.8868 | 192.932 | 1.00869 | 1.60204 | 0.0268 | 0.135478 |
| ppc | phosphoenolpyruvate carboxylase | chromosome:4418621-4421258 | 1448A-rhpS-KB | 1448A-WT-KB | OK | 131.677 | 264.919 | 1.00854 | 2.75369 | 0.0002 | 0.00374241 |
| PSPPH_1048 | luciferase | chromosome:1238661-1240481 | 1448A-rhpS-KB | 1448A-WT-KB | NOTEST | 8.47415 | 17.0471 | 1.00838 | 0 | 1 | 1 |
| PSPPH_3000 | peptide ABC transporter permease | chromosome:3483123-3484161 | 1448A-rhpS-KB | 1448A-WT-KB | NOTEST | 11.8601 | 23.8473 | 1.00771 | 0 | 1 | 1 |
| fliM | flagellar motor switch protein FliM | chromosome:3900549-3901518 | 1448A-rhpS-KB | 1448A-WT-KB | NOTEST | 7.52712 | 15.1265 | 1.00691 | 0 | 1 | 1 |
| nadC | nicotinate-nucleotide pyrophosphorylase | chromosome:1003622-1004471 | 1448A-rhpS-KB | 1448A-WT-KB | OK | 170.352 | 342.008 | 1.00552 | 1.8958 | 0.00985 | 0.0686236 |
| PSPPH_2132 | hypothetical protein | chromosome:2489069-2489291 | 1448A-rhpS-KB | 1448A-WT-KB | OK | 161.627 | 324.279 | 1.00457 | 0.614961 | 0.34755 | 0.58243 |
| gabP | GABA permease | chromosome:5610172-5611564 | 1448A-rhpS-KB | 1448A-WT-KB | OK | 66.3552 | 133.103 | 1.00426 | 1.62875 | 0.0254 | 0.130618 |
| PSPPH_0675 | hypothetical protein | chromosome:791289-791985 | 1448A-rhpS-KB | 1448A-WT-KB | OK | 116.326 | 233.213 | 1.00347 | 1.46102 | 0.046 | 0.193873 |
| prc | tail-specific protease | chromosome:1840635-1842750 | 1448A-rhpS-KB | 1448A-WT-KB | OK | 533.857 | 1070.29 | 1.00347 | 3.62128 | 0.00005 | 0.00121565 |
| hutH2 | histidine ammonia-lyase | chromosome:5531480-5533028 | 1448A-rhpS-KB | 1448A-WT-KB | OK | 62.8932 | 126.086 | 1.00343 | 1.68859 | 0.0201 | 0.110519 |
| ribBA2 | bifunctional 3,4-dihydroxy-2-butanone 4-phosphate synthase/GTP cyclohydrolase II-like protein | chromosome:5138502-5139594 | 1448A-rhpS-KB | 1448A-WT-KB | OK | 385.488 | 772.491 | 1.00283 | 2.85515 | 0.00015 | 0.00302881 |
| elbB | isoprenoid biosynthesis protein | chromosome:319241-319910 | 1448A-rhpS-KB | 1448A-WT-KB | OK | 70.603 | 141.361 | 1.00158 | 1.21153 | 0.0898 | 0.279064 |
| PSPPH_4367 | endoribonuclease L-PSP | chromosome:4989793-4990177 | 1448A-rhpS-KB | 1448A-WT-KB | OK | 44.1691 | 88.4346 | 1.00157 | 0.629478 | 0.34105 | 0.578003 |

**C. List of genes upregulated in *rhpS* mutant in MM**

| gene | Annotation | locus | sample_1 | sample_2 | status | value_1 | value_2 | log2(fold_change) | test_stat | p_value | q_value |
| --- | --- | --- | --- | --- | --- | --- | --- | --- | --- | --- | --- |
| PSPPH_1612 | hypothetical protein | chromosome:1870154-1873726 | 1448A-rhpS-MM | 1448A-WT-MM | NOTEST | 19.8253 | 9.91082 | -1.00027 | 0 | 1 | 1 |
| PSPPH_2871 | hypothetical protein | chromosome:3315938-3316214 | 1448A-rhpS-MM | 1448A-WT-MM | OK | 366.631 | 183.266 | -1.00039 | -0.770855 | 0.24215 | 0.592702 |
| PSPPH_1385 | succinylglutamate desuccinylase/aspartoacylase | chromosome:1607926-1609045 | 1448A-rhpS-MM | 1448A-WT-MM | NOTEST | 63.23 | 31.603 | -1.00054 | 0 | 1 | 1 |
| PSPPH_2685 | polysaccharide deacetylase | chromosome:3108978-3109758 | 1448A-rhpS-MM | 1448A-WT-MM | NOTEST | 52.1226 | 25.9925 | -1.00382 | 0 | 1 | 1 |
| PSPPH_0110 | short chain dehydrogenase/reductase oxidoreductase | chromosome:127469-128234 | 1448A-rhpS-MM | 1448A-WT-MM | OK | 105.38 | 52.5259 | -1.0045 | -0.977656 | 0.1499 | 0.496942 |
| PSPPH_A0085 | transposase, truncated | large_plasmid:74168-74843 | 1448A-rhpS-MM | 1448A-WT-MM | OK | 239.814 | 119.435 | -1.00569 | -1.39256 | 0.0523 | 0.326115 |
| PSPPH_2462 | glutamine ABC transporter permease | chromosome:2843728-2844430 | 1448A-rhpS-MM | 1448A-WT-MM | NOTEST | 26.8034 | 13.3402 | -1.00664 | 0 | 1 | 1 |
| ptsN | PTS transporter subunit IIA-like nitrogen-regulatory protein PtsN | chromosome:4736932-4737397 | 1448A-rhpS-MM | 1448A-WT-MM | OK | 510 | 253.768 | -1.00699 | -1.54714 | 0.0331 | 0.261409 |
| PSPPH_1620 | hypothetical protein | chromosome:1881194-1881605 | 1448A-rhpS-MM | 1448A-WT-MM | OK | 380.918 | 189.412 | -1.00795 | -1.25794 | 0.0804 | 0.388044 |
| nth | endonuclease III | chromosome:1596918-1598396 | 1448A-rhpS-MM | 1448A-WT-MM | OK | 128.161 | 63.652 | -1.00968 | -0.828487 | 0.21865 | 0.568636 |
| PSPPH_1876 | hypothetical protein | chromosome:2177183-2178107 | 1448A-rhpS-MM | 1448A-WT-MM | OK | 211.665 | 105.003 | -1.01135 | -1.50286 | 0.04865 | 0.313339 |
| PSPPH_1476 | hypothetical protein | chromosome:1715738-1716239 | 1448A-rhpS-MM | 1448A-WT-MM | OK | 197.58 | 98.0031 | -1.01154 | -1.00048 | 0.1439 | 0.494043 |
| PSPPH_4642 | hypothetical protein | chromosome:5280032-5280413 | 1448A-rhpS-MM | 1448A-WT-MM | OK | 280.229 | 138.93 | -1.01225 | -0.934374 | 0.15205 | 0.499569 |
| PSPPH_2715 | hypothetical protein | chromosome:3142283-3144463 | 1448A-rhpS-MM | 1448A-WT-MM | NOTEST | 53.1808 | 26.3508 | -1.01306 | 0 | 1 | 1 |
| PSPPH_0485 | hypothetical protein | chromosome:552521-553985 | 1448A-rhpS-MM | 1448A-WT-MM | OK | 90.3552 | 44.7599 | -1.0134 | -1.38009 | 0.0503 | 0.318567 |
| rpsT | 30S ribosomal protein S20 | chromosome:840942-841221 | 1448A-rhpS-MM | 1448A-WT-MM | OK | 1085.21 | 536.988 | -1.01501 | -1.45768 | 0.05985 | 0.344725 |
| ispH | 4-hydroxy-3-methylbut-2-enyl diphosphate reductase | chromosome:847936-848884 | 1448A-rhpS-MM | 1448A-WT-MM | OK | 321.791 | 159.187 | -1.0154 | -1.92327 | 0.0107 | 0.140207 |
| PSPPH_1704 | ABC transporter ATP-binding protein | chromosome:1970276-1971887 | 1448A-rhpS-MM | 1448A-WT-MM | OK | 393.348 | 194.344 | -1.01719 | -2.54273 | 0.0008 | 0.0231619 |
| PSPPH_3535 | peptidyl-tRNA hydrolase domain-containing protein | chromosome:4074843-4076952 | 1448A-rhpS-MM | 1448A-WT-MM | OK | 170.336 | 84.1089 | -1.01805 | -0.850115 | 0.21365 | 0.566293 |
| PSPPH_5053 | class V aminotransferase | chromosome:5728409-5729564 | 1448A-rhpS-MM | 1448A-WT-MM | OK | 496.778 | 244.695 | -1.02162 | -2.50155 | 0.00085 | 0.0237064 |
| pdxH | pyridoxamine 5'-phosphate oxidase | chromosome:1634278-1634926 | 1448A-rhpS-MM | 1448A-WT-MM | OK | 210.852 | 103.849 | -1.02174 | -1.28118 | 0.0826 | 0.392449 |
| PSPPH_3687 | hypothetical protein | chromosome:4235556-4236063 | 1448A-rhpS-MM | 1448A-WT-MM | OK | 103.347 | 50.8212 | -1.024 | -0.721682 | 0.2607 | 0.611159 |
| PSPPH_2947 | methyl-accepting chemotaxis protein | chromosome:3419776-3421390 | 1448A-rhpS-MM | 1448A-WT-MM | NOTEST | 38.3403 | 18.8509 | -1.02423 | 0 | 1 | 1 |
| PSPPH_5151 | cytosolic long-chain acyl-CoA thioester hydrolase | chromosome:5843140-5843653 | 1448A-rhpS-MM | 1448A-WT-MM | OK | 527.217 | 259.054 | -1.02514 | -1.69729 | 0.02305 | 0.218293 |
| rpsN | 30S ribosomal protein S14 | chromosome:5220860-5221166 | 1448A-rhpS-MM | 1448A-WT-MM | OK | 3519.76 | 1728.09 | -1.0263 | -2.46182 | 0.00155 | 0.0373968 |
| PSPPH_2419 | alcohol dehydrogenase | chromosome:2797531-2798686 | 1448A-rhpS-MM | 1448A-WT-MM | NOTEST | 23.1882 | 11.3777 | -1.02718 | 0 | 1 | 1 |
| PSPPH_1082 | hypothetical protein | chromosome:1273156-1275298 | 1448A-rhpS-MM | 1448A-WT-MM | NOTEST | 26.0603 | 12.7807 | -1.02789 | 0 | 1 | 1 |
| PSPPH_0851 | hypothetical protein | chromosome:1017698-1018568 | 1448A-rhpS-MM | 1448A-WT-MM | OK | 147.252 | 72.156 | -1.0291 | -1.32419 | 0.06705 | 0.370604 |
| mdoD | glucan biosynthesis protein D | chromosome:2202762-2204349 | 1448A-rhpS-MM | 1448A-WT-MM | NOTEST | 40.939 | 20.0552 | -1.0295 | 0 | 1 | 1 |
| PSPPH_1375 | hypothetical protein | chromosome:1596629-1596812 | 1448A-rhpS-MM | 1448A-WT-MM | OK | 2561.05 | 1253.39 | -1.0309 | -1.22928 | 0.0885 | 0.410817 |
| PSPPH_3706 | NAD-dependent deacetylase | chromosome:4252855-4253599 | 1448A-rhpS-MM | 1448A-WT-MM | OK | 83.3928 | 40.7912 | -1.03167 | -0.867215 | 0.18975 | 0.538883 |
| PSPPH_4401 | hypothetical protein | chromosome:5023836-5025804 | 1448A-rhpS-MM | 1448A-WT-MM | OK | 421.478 | 206.013 | -1.03272 | -0.624571 | 0.37005 | 0.696565 |
| PSPPH_2917 | DNA-binding protein | chromosome:3389553-3390129 | 1448A-rhpS-MM | 1448A-WT-MM | OK | 136.726 | 66.8259 | -1.03281 | -0.9775 | 0.1606 | 0.506614 |
| arsB | arsenical pump membrane protein | chromosome:4148478-4150708 | 1448A-rhpS-MM | 1448A-WT-MM | NOTEST | 43.016 | 20.988 | -1.03531 | 0 | 1 | 1 |
| pntB | NAD(P) transhydrogenase subunit beta | chromosome:5787250-5789025 | 1448A-rhpS-MM | 1448A-WT-MM | NOTEST | 43.8262 | 21.3734 | -1.03598 | 0 | 1 | 1 |
| PSPPH_4558 | MutT domain-containing protein | chromosome:5195903-5198625 | 1448A-rhpS-MM | 1448A-WT-MM | OK | 223.06 | 108.768 | -1.03618 | -0.765444 | 0.3653 | 0.694073 |
| PSPPH_1055 | acyltransferase | chromosome:1247271-1248177 | 1448A-rhpS-MM | 1448A-WT-MM | NOTEST | 58.0288 | 28.2932 | -1.03632 | 0 | 1 | 1 |
| PSPPH_2032 | Slt family transglycosylase | chromosome:2385633-2387055 | 1448A-rhpS-MM | 1448A-WT-MM | OK | 94.5758 | 46.0902 | -1.03701 | -1.38874 | 0.05445 | 0.332216 |
| PSPPH_5063 | hypothetical protein | chromosome:5739403-5741341 | 1448A-rhpS-MM | 1448A-WT-MM | NOTEST | 74.6541 | 36.3052 | -1.04005 | 0 | 1 | 1 |
| rimK | ribosomal protein S6 modification protein | chromosome:5684413-5685792 | 1448A-rhpS-MM | 1448A-WT-MM | OK | 121.355 | 58.9934 | -1.04061 | -1.16442 | 0.1094 | 0.450249 |
| PSPPH_3056 | general secretion pathway protein GspM | chromosome:3550674-3555047 | 1448A-rhpS-MM | 1448A-WT-MM | NOTEST | 61.7746 | 30.018 | -1.04119 | 0 | 1 | 1 |
| dehII2 | haloacid dehalogenase | chromosome:5697363-5698032 | 1448A-rhpS-MM | 1448A-WT-MM | OK | 131.586 | 63.9253 | -1.04155 | -1.06724 | 0.13195 | 0.477688 |
| PSPPH_2588 | mutT/nudix family protein | chromosome:2979803-2980166 | 1448A-rhpS-MM | 1448A-WT-MM | OK | 456.873 | 221.902 | -1.04187 | -1.20785 | 0.09055 | 0.416448 |
| algK | alginate biosynthesis protein AlgK | chromosome:1311570-1314461 | 1448A-rhpS-MM | 1448A-WT-MM | OK | 147.186 | 71.3529 | -1.04459 | -1.61211 | 0.03325 | 0.261409 |
| PSPPH_2090 | hypothetical protein | chromosome:2451213-2451965 | 1448A-rhpS-MM | 1448A-WT-MM | OK | 233.951 | 113.354 | -1.04537 | -0.704657 | 0.3954 | 0.711252 |
| PSPPH_3802 | hypothetical protein | chromosome:4353191-4353608 | 1448A-rhpS-MM | 1448A-WT-MM | OK | 541.027 | 262.062 | -1.0458 | -1.49255 | 0.04695 | 0.308222 |
| PSPPH_0733 | RND efflux transporter | chromosome:856468-860643 | 1448A-rhpS-MM | 1448A-WT-MM | NOTEST | 26.8358 | 12.9792 | -1.04796 | 0 | 1 | 1 |
| PSPPH_1014 | hypothetical protein | chromosome:1204399-1205598 | 1448A-rhpS-MM | 1448A-WT-MM | OK | 823.752 | 398.363 | -1.04813 | -2.79788 | 0.00025 | 0.00987013 |
| cyaA | adenylate cyclase | chromosome:5678762-5681609 | 1448A-rhpS-MM | 1448A-WT-MM | NOTEST | 45.8954 | 22.1479 | -1.05118 | 0 | 1 | 1 |
| fabG | 3-ketoacyl-ACP reductase | chromosome:476539-481262 | 1448A-rhpS-MM | 1448A-WT-MM | OK | 100.405 | 48.435 | -1.05171 | -0.735839 | 0.37415 | 0.699835 |
| PSPPH_3831 | outer membrane protein OmpH | chromosome:4385143-4385647 | 1448A-rhpS-MM | 1448A-WT-MM | OK | 871.441 | 420.354 | -1.0518 | -2.07825 | 0.0069 | 0.101333 |
| PSPPH_1546 | hypothetical protein | chromosome:1798652-1800296 | 1448A-rhpS-MM | 1448A-WT-MM | OK | 76.9697 | 37.0108 | -1.05635 | -1.33078 | 0.07835 | 0.386887 |
| PSPPH_0503 | solute-binding family 3 protein | chromosome:582367-585284 | 1448A-rhpS-MM | 1448A-WT-MM | OK | 94.5329 | 45.4192 | -1.05752 | -0.818612 | 0.28495 | 0.631947 |
| PSPPH_2191 | sodium:solute symporter family protein | chromosome:2546184-2547971 | 1448A-rhpS-MM | 1448A-WT-MM | NOTEST | 18.2156 | 8.73212 | -1.06077 | 0 | 1 | 1 |
| PSPPH_4385 | hypothetical protein | chromosome:5009450-5009999 | 1448A-rhpS-MM | 1448A-WT-MM | OK | 271.249 | 129.848 | -1.06279 | -1.29398 | 0.0748 | 0.383183 |
| fdhD | formate dehydrogenase accessory protein FdhD | chromosome:164566-167736 | 1448A-rhpS-MM | 1448A-WT-MM | OK | 409.156 | 195.769 | -1.0635 | -1.39733 | 0.05115 | 0.322529 |
| PSPPH_0046 | ISPsy18, transposase | chromosome:47646-48906 | 1448A-rhpS-MM | 1448A-WT-MM | NOTEST | 24.793 | 11.8545 | -1.0645 | 0 | 1 | 1 |
| PSPPH_4546 | hypothetical protein | chromosome:5181947-5183480 | 1448A-rhpS-MM | 1448A-WT-MM | OK | 145.993 | 69.7929 | -1.06474 | -1.76626 | 0.0178 | 0.189203 |
| PSPPH_4374 | hypothetical protein | chromosome:4996458-4996878 | 1448A-rhpS-MM | 1448A-WT-MM | OK | 478.25 | 228.211 | -1.0674 | -1.46837 | 0.044 | 0.296585 |
| PSPPH_2372 | branched-chain amino acid ABC transporter ATP-binding protein | chromosome:2750146-2750926 | 1448A-rhpS-MM | 1448A-WT-MM | NOTEST | 15.9986 | 7.62861 | -1.06845 | 0 | 1 | 1 |
| dsbE | thiol:disulfide interchange protein DsbE | chromosome:3829675-3831842 | 1448A-rhpS-MM | 1448A-WT-MM | NOTEST | 18.8682 | 8.99318 | -1.06905 | 0 | 1 | 1 |
| PSPPH_4467 | hypothetical protein | chromosome:5100412-5101786 | 1448A-rhpS-MM | 1448A-WT-MM | OK | 1574.42 | 750.14 | -1.06959 | -3.48408 | 5.00E-05 | 0.00230303 |
| ipk | 4-diphosphocytidyl-2-C-methyl-D-erythritol kinase | chromosome:1186252-1187119 | 1448A-rhpS-MM | 1448A-WT-MM | OK | 158.777 | 75.6201 | -1.07016 | -1.41371 | 0.0621 | 0.351836 |
| PSPPH_1634 | HAD superfamily hydrolase | chromosome:1897706-1899315 | 1448A-rhpS-MM | 1448A-WT-MM | OK | 126.831 | 60.3546 | -1.07137 | -1.02587 | 0.1507 | 0.497755 |
| PSPPH_2023 | lipoprotein | chromosome:2376421-2376700 | 1448A-rhpS-MM | 1448A-WT-MM | OK | 352.156 | 167.393 | -1.07298 | -0.811322 | 0.23185 | 0.581538 |
| PSPPH_3938 | hypothetical protein | chromosome:4498600-4499904 | 1448A-rhpS-MM | 1448A-WT-MM | NOTEST | 39.5948 | 18.8064 | -1.07409 | 0 | 1 | 1 |
| PSPPH_4629 | cpaA protein | chromosome:5266390-5267488 | 1448A-rhpS-MM | 1448A-WT-MM | NOTEST | 58.8819 | 27.9592 | -1.0745 | 0 | 1 | 1 |
| PSPPH_3208 | hypothetical protein | chromosome:3719063-3719393 | 1448A-rhpS-MM | 1448A-WT-MM | OK | 113.844 | 54.0345 | -1.0751 | -0.676669 | 0.39255 | 0.709906 |
| pilH | type IV pilus response regulator PilH | chromosome:542416-542782 | 1448A-rhpS-MM | 1448A-WT-MM | OK | 434.526 | 205.932 | -1.07728 | -1.26437 | 0.09235 | 0.419647 |
| PSPPH_5112 | chorismate--pyruvate lyase | chromosome:5797829-5799280 | 1448A-rhpS-MM | 1448A-WT-MM | NOTEST | 40.4348 | 19.1543 | -1.07793 | 0 | 1 | 1 |
| PSPPH_2241 | hypothetical protein | chromosome:2597977-2599996 | 1448A-rhpS-MM | 1448A-WT-MM | NOTEST | 16.2758 | 7.70243 | -1.07934 | 0 | 1 | 1 |
| PSPPH_5074 | Rhs family protein | chromosome:5756380-5756764 | 1448A-rhpS-MM | 1448A-WT-MM | OK | 173.517 | 82.0779 | -1.08001 | -0.863924 | 0.23465 | 0.585181 |
| PSPPH_2062 | esterase | chromosome:2418723-2419716 | 1448A-rhpS-MM | 1448A-WT-MM | OK | 333.964 | 157.942 | -1.08029 | -2.15235 | 0.00555 | 0.0865231 |
| PSPPH_4840 | Na+/H+ antiporter NhaP | chromosome:5499249-5500509 | 1448A-rhpS-MM | 1448A-WT-MM | OK | 144.006 | 68.0968 | -1.08047 | -1.63973 | 0.0292 | 0.245377 |
| PSPPH_1644 | 4-amino-4-deoxychorismate lyase | chromosome:1905199-1907259 | 1448A-rhpS-MM | 1448A-WT-MM | OK | 286.499 | 135.46 | -1.08067 | -0.857514 | 0.2459 | 0.596121 |
| cyoC | cytochrome o ubiquinol oxidase subunit III | chromosome:1422468-1423427 | 1448A-rhpS-MM | 1448A-WT-MM | OK | 2732 | 1291.55 | -1.08085 | -2.84364 | 0.0003 | 0.0108571 |
| PSPPH_3010 | acetyltransferase | chromosome:3494581-3495208 | 1448A-rhpS-MM | 1448A-WT-MM | NOTEST | 29.7908 | 14.0741 | -1.08182 | 0 | 1 | 1 |
| PSPPH_2238 | hypothetical protein | chromosome:2596962-2597970 | 1448A-rhpS-MM | 1448A-WT-MM | OK | 300.484 | 141.882 | -1.0826 | -2.02497 | 0.0076 | 0.110019 |
| PSPPH_1102 | fusaric acid resistance protein | chromosome:1295168-1299907 | 1448A-rhpS-MM | 1448A-WT-MM | NOTEST | 11.8554 | 5.58874 | -1.08495 | 0 | 1 | 1 |
| ligD | ATP-dependent DNA ligase | chromosome:3670091-3672692 | 1448A-rhpS-MM | 1448A-WT-MM | NOTEST | 30.2197 | 14.2362 | -1.08592 | 0 | 1 | 1 |
| PSPPH_2161 | flavin reductase domain-containing protein | chromosome:2513941-2517414 | 1448A-rhpS-MM | 1448A-WT-MM | OK | 149.544 | 70.4112 | -1.0867 | -0.867243 | 0.21325 | 0.566183 |
| PSPPH_4409 | iojap domain-containing protein | chromosome:5032852-5033239 | 1448A-rhpS-MM | 1448A-WT-MM | OK | 428.197 | 201.48 | -1.08764 | -1.26311 | 0.0808 | 0.388044 |
| PSPPH_3060 | general secretion pathway protein GspI | chromosome:3550674-3555047 | 1448A-rhpS-MM | 1448A-WT-MM | OK | 128.124 | 60.2555 | -1.08838 | -0.526663 | 0.482 | 0.769176 |
| fabZ | (3R)-hydroxymyristoyl-ACP dehydratase | chromosome:4382762-4383976 | 1448A-rhpS-MM | 1448A-WT-MM | OK | 522.777 | 245.751 | -1.08899 | -1.086 | 0.14745 | 0.494335 |
| PSPPH_1193 | diguanylate cyclase | chromosome:1400397-1401927 | 1448A-rhpS-MM | 1448A-WT-MM | OK | 146.726 | 68.9431 | -1.08965 | -1.81862 | 0.01595 | 0.177612 |
| mnmA | tRNA-specific 2-thiouridylase MnmA | chromosome:3600106-3601857 | 1448A-rhpS-MM | 1448A-WT-MM | OK | 129.59 | 60.8103 | -1.09156 | -1.32636 | 0.0773 | 0.386887 |
| PSPPH_1126 | lipoprotein | chromosome:1325704-1326738 | 1448A-rhpS-MM | 1448A-WT-MM | NOTEST | 36.9223 | 17.281 | -1.0953 | 0 | 1 | 1 |
| lolB | molecular chaperone LolB | chromosome:1187121-1187739 | 1448A-rhpS-MM | 1448A-WT-MM | OK | 140.697 | 65.8054 | -1.09632 | -1.06892 | 0.13975 | 0.488174 |
| PSPPH_1197 | HlyD family secretion protein | chromosome:1404177-1408445 | 1448A-rhpS-MM | 1448A-WT-MM | NOTEST | 27.1794 | 12.6654 | -1.10162 | 0 | 1 | 1 |
| cobI | precorrin-2 C(20)-methyltransferase | chromosome:5091114-5095509 | 1448A-rhpS-MM | 1448A-WT-MM | NOTEST | 74.0752 | 34.4777 | -1.10332 | 0 | 1 | 1 |
| PSPPH_4251 | major facilitator superfamily transporter phthalate permease | chromosome:4850202-4851588 | 1448A-rhpS-MM | 1448A-WT-MM | NOTEST | 56.4389 | 26.2584 | -1.10391 | 0 | 1 | 1 |
| PSPPH_0174 | hypothetical protein | chromosome:208087-208642 | 1448A-rhpS-MM | 1448A-WT-MM | NOTEST | 69.1085 | 32.0395 | -1.10901 | 0 | 1 | 1 |
| PSPPH_2196 | PqiB family protein | chromosome:2550751-2553051 | 1448A-rhpS-MM | 1448A-WT-MM | OK | 257.86 | 119.433 | -1.11038 | -2.31882 | 0.00255 | 0.0503377 |
| PSPPH_4355 | hypothetical protein | chromosome:4978269-4979184 | 1448A-rhpS-MM | 1448A-WT-MM | NOTEST | 44.6644 | 20.6577 | -1.11245 | 0 | 1 | 1 |
| PSPPH_A0114 | transposase family protein | large_plasmid:97939-98182 | 1448A-rhpS-MM | 1448A-WT-MM | NOTEST | 96.8751 | 44.7989 | -1.11266 | 0 | 1 | 1 |
| algL | poly(beta-D-mannuronate) lyase | chromosome:1307328-1308471 | 1448A-rhpS-MM | 1448A-WT-MM | OK | 198.476 | 91.6888 | -1.11414 | -1.84578 | 0.0155 | 0.174435 |
| PSPPH_3784 | lipoprotein | chromosome:4334179-4335136 | 1448A-rhpS-MM | 1448A-WT-MM | OK | 168.723 | 77.9142 | -1.1147 | -1.6029 | 0.03385 | 0.26251 |
| PSPPH_2956 | carbon-nitrogen family hydrolase | chromosome:3429963-3430704 | 1448A-rhpS-MM | 1448A-WT-MM | OK | 106.16 | 48.9181 | -1.11781 | -1.05603 | 0.1439 | 0.494043 |
| capA | cold shock protein CapA | chromosome:2489987-2490200 | 1448A-rhpS-MM | 1448A-WT-MM | OK | 3296.24 | 1518.83 | -1.11786 | -1.85307 | 0.01505 | 0.172 |
| pqqF | coenzyme PQQ biosynthesis protein PqqF | chromosome:5338947-5341269 | 1448A-rhpS-MM | 1448A-WT-MM | NOTEST | 66.0934 | 30.4313 | -1.11895 | 0 | 1 | 1 |
| PSPPH_1045 | alpha/beta hydrolase | chromosome:1237443-1238223 | 1448A-rhpS-MM | 1448A-WT-MM | NOTEST | 11.1313 | 5.11089 | -1.12298 | 0 | 1 | 1 |
| cyoE | protoheme IX farnesyltransferase | chromosome:1423437-1424325 | 1448A-rhpS-MM | 1448A-WT-MM | OK | 1866.29 | 856.787 | -1.12317 | -3.43178 | 5.00E-05 | 0.00230303 |
| PSPPH_1970 | hypothetical protein | chromosome:2318479-2319223 | 1448A-rhpS-MM | 1448A-WT-MM | OK | 103.325 | 47.2911 | -1.12755 | -1.01708 | 0.16695 | 0.514249 |
| PSPPH_3908 | 2-hydroxychromene-2-carboxylate isomerase | chromosome:4468355-4469003 | 1448A-rhpS-MM | 1448A-WT-MM | OK | 166.502 | 76.1384 | -1.12884 | -1.18073 | 0.10135 | 0.439521 |
| PSPPH_4328 | cobalamin synthesis protein/P47K family protein | chromosome:4948394-4949372 | 1448A-rhpS-MM | 1448A-WT-MM | OK | 236.859 | 108.195 | -1.13039 | -1.87267 | 0.01435 | 0.169674 |
| PSPPH_3221 | lipid kinase | chromosome:3731448-3733012 | 1448A-rhpS-MM | 1448A-WT-MM | OK | 141.222 | 64.4727 | -1.1312 | -1.36965 | 0.06475 | 0.361174 |
| PSPPH_4215 | hypothetical protein | chromosome:4803942-4804227 | 1448A-rhpS-MM | 1448A-WT-MM | OK | 549.107 | 250.318 | -1.13332 | -1.16143 | 0.1287 | 0.475299 |
| algF | alginate biosynthesis protein AlgF | chromosome:1303715-1304384 | 1448A-rhpS-MM | 1448A-WT-MM | OK | 402.101 | 183.041 | -1.13539 | -1.94139 | 0.0106 | 0.139498 |
| PSPPH_2202 | hypothetical protein | chromosome:2557134-2557389 | 1448A-rhpS-MM | 1448A-WT-MM | NOTEST | 78.8856 | 35.8818 | -1.13651 | 0 | 1 | 1 |
| PSPPH_3031 | GntR family transcriptional regulator | chromosome:3519677-3520391 | 1448A-rhpS-MM | 1448A-WT-MM | OK | 163.736 | 74.1865 | -1.14214 | -1.29093 | 0.0785 | 0.386887 |
| PSPPH_2699 | peptide ABC transporter substrate-binding protein | chromosome:3126750-3129501 | 1448A-rhpS-MM | 1448A-WT-MM | NOTEST | 27.5447 | 12.4433 | -1.1464 | 0 | 1 | 1 |
| amiC | N-acetylmuramoyl-L-alanine amidase | chromosome:5848955-5850194 | 1448A-rhpS-MM | 1448A-WT-MM | OK | 331.668 | 149.83 | -1.14641 | -2.40396 | 0.00205 | 0.0445143 |
| PSPPH_2234 | hypothetical protein | chromosome:2592311-2593927 | 1448A-rhpS-MM | 1448A-WT-MM | OK | 155.89 | 70.3994 | -1.14689 | -0.652446 | 0.404 | 0.719152 |
| PSPPH_5175 | methyl-accepting chemotaxis protein | chromosome:5871356-5873279 | 1448A-rhpS-MM | 1448A-WT-MM | NOTEST | 16.1416 | 7.28398 | -1.14798 | 0 | 1 | 1 |
| PSPPH_1560 | glutamine amidotransferase | chromosome:1811809-1812598 | 1448A-rhpS-MM | 1448A-WT-MM | OK | 97.6185 | 43.9556 | -1.15111 | -1.04862 | 0.1474 | 0.494335 |
| PSPPH_2193 | LysR family transcriptional regulator | chromosome:2549078-2549990 | 1448A-rhpS-MM | 1448A-WT-MM | NOTEST | 57.5717 | 25.8979 | -1.15252 | 0 | 1 | 1 |
| PSPPH_1395 | hypothetical protein | chromosome:1619654-1619984 | 1448A-rhpS-MM | 1448A-WT-MM | NOTEST | 60.0636 | 27.0173 | -1.15261 | 0 | 1 | 1 |
| PSPPH_1367 | hypothetical protein | chromosome:1586456-1587335 | 1448A-rhpS-MM | 1448A-WT-MM | OK | 219.513 | 98.6032 | -1.1546 | -1.69271 | 0.0239 | 0.219099 |
| PSPPH_1748 | acetyltransferase | chromosome:2027935-2028454 | 1448A-rhpS-MM | 1448A-WT-MM | OK | 93.5145 | 41.9599 | -1.15618 | -0.777213 | 0.26255 | 0.612429 |
| PSPPH_1116 | alginate biosynthesis protein Alg44 | chromosome:1314463-1315636 | 1448A-rhpS-MM | 1448A-WT-MM | OK | 225.091 | 100.868 | -1.15803 | -2.00586 | 0.00855 | 0.120893 |
| PSPPH_4730 | LacI family transcriptional regulator | chromosome:5363953-5368805 | 1448A-rhpS-MM | 1448A-WT-MM | NOTEST | 10.4653 | 4.68661 | -1.15899 | 0 | 1 | 1 |
| PSPPH_4036 | hypothetical protein | chromosome:4610450-4611794 | 1448A-rhpS-MM | 1448A-WT-MM | NOTEST | 63.0091 | 28.1799 | -1.16089 | 0 | 1 | 1 |
| PSPPH_2567 | hypothetical protein | chromosome:2958506-2959097 | 1448A-rhpS-MM | 1448A-WT-MM | OK | 140.132 | 62.6354 | -1.16174 | -1.04156 | 0.1584 | 0.506614 |
| PSPPH_3126 | sensor histidine kinase | chromosome:3628139-3630740 | 1448A-rhpS-MM | 1448A-WT-MM | NOTEST | 34.2409 | 15.3047 | -1.16174 | 0 | 1 | 1 |
| PSPPH_2119 | hypothetical protein | chromosome:2478965-2480011 | 1448A-rhpS-MM | 1448A-WT-MM | OK | 733.773 | 327.273 | -1.16484 | -0.809617 | 0.21885 | 0.568636 |
| PSPPH_0301 | hypothetical protein | chromosome:343762-343915 | 1448A-rhpS-MM | 1448A-WT-MM | OK | 978.082 | 436.07 | -1.1654 | -0.928716 | 0.4017 | 0.71749 |
| PSPPH_0603 | hypothetical protein | chromosome:713112-714129 | 1448A-rhpS-MM | 1448A-WT-MM | NOTEST | 17.1861 | 7.66203 | -1.16545 | 0 | 1 | 1 |
| PSPPH_5174 | ABC transporter permease | chromosome:5869113-5870868 | 1448A-rhpS-MM | 1448A-WT-MM | NOTEST | 47.4132 | 21.1217 | -1.16656 | 0 | 1 | 1 |
| PSPPH_4291 | lipoprotein | chromosome:4898827-4899181 | 1448A-rhpS-MM | 1448A-WT-MM | OK | 514.965 | 229.208 | -1.16782 | -1.41352 | 0.0698 | 0.3744 |
| PSPPH_2840 | sodium/hydrogen exchanger family protein | chromosome:3286638-3287967 | 1448A-rhpS-MM | 1448A-WT-MM | NOTEST | 13.8384 | 6.14885 | -1.17029 | 0 | 1 | 1 |
| cbrC | achromobactin transport system permease CbrC | chromosome:3192049-3196853 | 1448A-rhpS-MM | 1448A-WT-MM | NOTEST | 46.5349 | 20.6453 | -1.1725 | 0 | 1 | 1 |
| PSPPH_0671 | prophage PSPPH01, ImpB/MucB/SamB family protein | chromosome:785688-788396 | 1448A-rhpS-MM | 1448A-WT-MM | NOTEST | 29.9399 | 13.2645 | -1.1745 | 0 | 1 | 1 |
| PSPPH_4815 | hypothetical protein | chromosome:5467045-5467465 | 1448A-rhpS-MM | 1448A-WT-MM | OK | 347.652 | 153.757 | -1.17699 | -1.32562 | 0.06755 | 0.371617 |
| PSPPH_4065 | diguanylate cyclase | chromosome:4646207-4648259 | 1448A-rhpS-MM | 1448A-WT-MM | OK | 130.338 | 57.4888 | -1.18091 | -2.09156 | 0.0065 | 0.0988 |
| PSPPH_1160 | cold shock domain-contain protein | chromosome:1361946-1362159 | 1448A-rhpS-MM | 1448A-WT-MM | OK | 2811.5 | 1237.98 | -1.18335 | -1.83712 | 0.02155 | 0.207975 |
| PSPPH_3575 | major facilitator family transporter | chromosome:4118870-4120184 | 1448A-rhpS-MM | 1448A-WT-MM | OK | 115.385 | 50.6499 | -1.18782 | -1.61329 | 0.0338 | 0.26251 |
| PSPPH_0015 | hypothetical protein | chromosome:18980-19598 | 1448A-rhpS-MM | 1448A-WT-MM | NOTEST | 54.3894 | 23.8041 | -1.19212 | 0 | 1 | 1 |
| PSPPH_2383 | lipoprotein | chromosome:2764868-2765246 | 1448A-rhpS-MM | 1448A-WT-MM | OK | 401.264 | 175.179 | -1.19572 | -1.29072 | 0.0726 | 0.381181 |
| PSPPH_0541 | hypothetical protein | chromosome:634594-634969 | 1448A-rhpS-MM | 1448A-WT-MM | NOTEST | 42.7796 | 18.6428 | -1.1983 | 0 | 1 | 1 |
| PSPPH_3694 | alpha-ribazole-5'-phosphate phosphatase | chromosome:4241334-4243474 | 1448A-rhpS-MM | 1448A-WT-MM | NOTEST | 45.408 | 19.7506 | -1.20105 | 0 | 1 | 1 |
| aas | acyltransferase | chromosome:1823253-1825116 | 1448A-rhpS-MM | 1448A-WT-MM | NOTEST | 71.98 | 31.2813 | -1.2023 | 0 | 1 | 1 |
| PSPPH_0723 | FKBP-type peptidylprolyl isomerase | chromosome:844079-847879 | 1448A-rhpS-MM | 1448A-WT-MM | OK | 351.78 | 152.683 | -1.20413 | -0.823326 | 0.2409 | 0.59107 |
| PSPPH_1146 | TetR family transcriptional regulator | chromosome:1344086-1345496 | 1448A-rhpS-MM | 1448A-WT-MM | OK | 264.882 | 114.661 | -1.20797 | -1.08713 | 0.1456 | 0.494335 |
| PSPPH_3228 | group 1 glycosyl transferase | chromosome:3741843-3746385 | 1448A-rhpS-MM | 1448A-WT-MM | OK | 85.2298 | 36.869 | -1.20895 | -1.07265 | 0.15925 | 0.506614 |
| PSPPH_4697 | Fic family protein | chromosome:5332938-5333850 | 1448A-rhpS-MM | 1448A-WT-MM | NOTEST | 32.6992 | 14.1438 | -1.20908 | 0 | 1 | 1 |
| PSPPH_3565 | hypothetical protein | chromosome:4104748-4110605 | 1448A-rhpS-MM | 1448A-WT-MM | NOTEST | 21.9219 | 9.45703 | -1.21292 | 0 | 1 | 1 |
| PSPPH_0605 | O-antigen ABC transporter permease | chromosome:715283-715979 | 1448A-rhpS-MM | 1448A-WT-MM | NOTEST | 57.9166 | 24.9688 | -1.21385 | 0 | 1 | 1 |
| PSPPH_0814 | HlyD family type I secretion membrane fusion protein | chromosome:958235-961743 | 1448A-rhpS-MM | 1448A-WT-MM | NOTEST | 6.02359 | 2.59277 | -1.21613 | 0 | 1 | 1 |
| PSPPH_1012 | hypothetical protein | chromosome:1203209-1204166 | 1448A-rhpS-MM | 1448A-WT-MM | OK | 160.758 | 68.8821 | -1.22269 | -1.64608 | 0.02905 | 0.245311 |
| bfr2 | bacterioferritin | chromosome:5211510-5211975 | 1448A-rhpS-MM | 1448A-WT-MM | OK | 1086.44 | 465.175 | -1.22377 | -2.44121 | 0.0023 | 0.0475646 |
| PSPPH_0750 | hypothetical protein | chromosome:874873-875842 | 1448A-rhpS-MM | 1448A-WT-MM | NOTEST | 24.423 | 10.4359 | -1.22669 | 0 | 1 | 1 |
| PSPPH_3966 | hypothetical protein | chromosome:4528768-4529803 | 1448A-rhpS-MM | 1448A-WT-MM | NOTEST | 41.7742 | 17.8367 | -1.22777 | 0 | 1 | 1 |
| PSPPH_4832 | hypothetical protein | chromosome:5488055-5488643 | 1448A-rhpS-MM | 1448A-WT-MM | OK | 151.197 | 64.5304 | -1.22838 | -1.13479 | 0.11725 | 0.457362 |
| PSPPH_1117 | alginate biosynthesis protein Alg8 | chromosome:1315676-1317158 | 1448A-rhpS-MM | 1448A-WT-MM | OK | 207.769 | 88.6701 | -1.22846 | -2.30051 | 0.0036 | 0.0647574 |
| PSPPH_3004 | GntR family transcriptional regulator | chromosome:3487682-3488465 | 1448A-rhpS-MM | 1448A-WT-MM | OK | 163.603 | 69.7515 | -1.22991 | -1.52183 | 0.04605 | 0.30433 |
| PSPPH_0890 | carbonic anhydrase | chromosome:1062138-1062777 | 1448A-rhpS-MM | 1448A-WT-MM | OK | 976.285 | 416.183 | -1.23009 | -2.75728 | 0.00065 | 0.01976 |
| purD | phosphoribosylamine--glycine ligase | chromosome:5082974-5084327 | 1448A-rhpS-MM | 1448A-WT-MM | OK | 382.747 | 162.974 | -1.23175 | -2.75859 | 0.00075 | 0.0219231 |
| tauA | taurine ABC transporter periplasmic binding protein | chromosome:5582344-5583322 | 1448A-rhpS-MM | 1448A-WT-MM | NOTEST | 13.6337 | 5.79564 | -1.23414 | 0 | 1 | 1 |
| PSPPH_3321 | endoribonuclease L-PSP | chromosome:3841513-3843029 | 1448A-rhpS-MM | 1448A-WT-MM | NOTEST | 37.3645 | 15.8108 | -1.24076 | 0 | 1 | 1 |
| PSPPH_5123 | phosphate ABC transporter substrate-binding protein | chromosome:5811837-5812836 | 1448A-rhpS-MM | 1448A-WT-MM | NOTEST | 31.7609 | 13.4301 | -1.24178 | 0 | 1 | 1 |
| PSPPH_3868 | ompA family protein | chromosome:4422110-4422893 | 1448A-rhpS-MM | 1448A-WT-MM | OK | 924.095 | 390.6 | -1.24235 | -2.97351 | 0.00025 | 0.00987013 |
| PSPPH_4183 | hypothetical protein | chromosome:4767726-4771078 | 1448A-rhpS-MM | 1448A-WT-MM | OK | 99.5389 | 41.9656 | -1.24605 | -0.684719 | 0.4522 | 0.75657 |
| PSPPH_4953 | prophage PSPPH06 tail fiber protein | chromosome:5625698-5628143 | 1448A-rhpS-MM | 1448A-WT-MM | NOTEST | 15.6279 | 6.57942 | -1.24809 | 0 | 1 | 1 |
| PSPPH_2810 | dolichyl-phosphate-mannose-protein mannosyltransferase | chromosome:3255003-3256623 | 1448A-rhpS-MM | 1448A-WT-MM | OK | 104.587 | 44.0071 | -1.24889 | -1.76282 | 0.02595 | 0.229994 |
| PSPPH_2907 | AcrB/AcrD/AcrF family transporter | chromosome:3374729-3377873 | 1448A-rhpS-MM | 1448A-WT-MM | NOTEST | 20.7235 | 8.71675 | -1.2494 | 0 | 1 | 1 |
| PSPPH_4021 | hypothetical protein | chromosome:4596547-4596856 | 1448A-rhpS-MM | 1448A-WT-MM | OK | 698.343 | 293.44 | -1.25087 | -1.43646 | 0.06325 | 0.354294 |
| algJ | alginate biosynthesis protein AlgJ | chromosome:1304396-1305572 | 1448A-rhpS-MM | 1448A-WT-MM | OK | 156.058 | 65.5196 | -1.25209 | -1.88135 | 0.01705 | 0.186323 |
| PSPPH_0226 | DNA-binding protein | chromosome:263695-264244 | 1448A-rhpS-MM | 1448A-WT-MM | OK | 120.65 | 50.5664 | -1.25457 | -1.03282 | 0.17675 | 0.522685 |
| PSPPH_2683 | 30S ribosomal protein S6 modification protein | chromosome:3106018-3107605 | 1448A-rhpS-MM | 1448A-WT-MM | NOTEST | 48.7019 | 20.4024 | -1.25524 | 0 | 1 | 1 |
| PSPPH_4974 | hypothetical protein | chromosome:5642992-5643945 | 1448A-rhpS-MM | 1448A-WT-MM | OK | 552.946 | 231.244 | -1.25773 | -1.7699 | 0.03425 | 0.262929 |
| dtd | D-tyrosyl-tRNA(Tyr) deacylase | chromosome:409774-410212 | 1448A-rhpS-MM | 1448A-WT-MM | NOTEST | 61.1674 | 25.5373 | -1.26015 | 0 | 1 | 1 |
| PSPPH_3305 | lipoprotein | chromosome:3829256-3829664 | 1448A-rhpS-MM | 1448A-WT-MM | OK | 283.833 | 118.355 | -1.26192 | -1.252 | 0.089 | 0.411185 |
| PSPPH_2539 | LuxR family transcriptional regulator | chromosome:2922030-2924841 | 1448A-rhpS-MM | 1448A-WT-MM | OK | 88.4177 | 36.8453 | -1.26285 | -2.1933 | 0.0045 | 0.0747541 |
| PSPPH_3859 | hypothetical protein | chromosome:4414690-4415398 | 1448A-rhpS-MM | 1448A-WT-MM | NOTEST | 43.3018 | 18.0366 | -1.2635 | 0 | 1 | 1 |
| PSPPH_1374 | LuxR family transcriptional regulator | chromosome:1595841-1596468 | 1448A-rhpS-MM | 1448A-WT-MM | OK | 146.711 | 61.0906 | -1.26395 | -1.18237 | 0.10915 | 0.450249 |
| PSPPH_3041 | sensor histidine kinase | chromosome:3531152-3533211 | 1448A-rhpS-MM | 1448A-WT-MM | OK | 122.997 | 51.1836 | -1.26487 | -1.51096 | 0.0458 | 0.303338 |
| PSPPH_0857 | Fis family transcriptional regulator | chromosome:1025177-1026611 | 1448A-rhpS-MM | 1448A-WT-MM | OK | 270.991 | 112.656 | -1.26631 | -2.57676 | 0.0017 | 0.0394504 |
| rnhB | ribonuclease HII | chromosome:4380952-4382760 | 1448A-rhpS-MM | 1448A-WT-MM | OK | 240.138 | 99.8112 | -1.26659 | -1.37141 | 0.07445 | 0.382824 |
| PSPPH_0751 | hypothetical protein | chromosome:876090-877905 | 1448A-rhpS-MM | 1448A-WT-MM | NOTEST | 26.5514 | 11.0298 | -1.26738 | 0 | 1 | 1 |
| PSPPH_4544 | hypothetical protein | chromosome:5179498-5181795 | 1448A-rhpS-MM | 1448A-WT-MM | NOTEST | 54.9676 | 22.824 | -1.26803 | 0 | 1 | 1 |
| PSPPH_0924 | sensory box protein | chromosome:1099950-1100433 | 1448A-rhpS-MM | 1448A-WT-MM | OK | 91.7531 | 38.0752 | -1.26891 | -0.814584 | 0.2721 | 0.620278 |
| PSPPH_1807 | hypothetical protein | chromosome:2110653-2110971 | 1448A-rhpS-MM | 1448A-WT-MM | OK | 104.873 | 43.3796 | -1.27356 | -0.678636 | 0.39465 | 0.711252 |
| PSPPH_3495 | diguanylate cyclase | chromosome:4036669-4038583 | 1448A-rhpS-MM | 1448A-WT-MM | NOTEST | 49.0787 | 20.2645 | -1.27614 | 0 | 1 | 1 |
| colS | sensor histidine kinase ColS | chromosome:4656299-4658262 | 1448A-rhpS-MM | 1448A-WT-MM | OK | 90.367 | 37.2747 | -1.2776 | -1.14543 | 0.14705 | 0.494335 |
| PSPPH_3301 | NodT family outer membrane efflux lipoprotein | chromosome:3821217-3826271 | 1448A-rhpS-MM | 1448A-WT-MM | NOTEST | 64.4592 | 26.5609 | -1.27908 | 0 | 1 | 1 |
| PSPPH_2370 | branched-chain amino acid ABC transporter permease | chromosome:2747694-2748762 | 1448A-rhpS-MM | 1448A-WT-MM | NOTEST | 32.631 | 13.4431 | -1.27938 | 0 | 1 | 1 |
| tonB3 | ferric siderophore transporter, periplasmic energy transduction protein TonB | chromosome:3338452-3339283 | 1448A-rhpS-MM | 1448A-WT-MM | NOTEST | 9.67648 | 3.98424 | -1.28018 | 0 | 1 | 1 |
| PSPPH_3729 | sensor histidine kinase PhoQ | chromosome:4276885-4278906 | 1448A-rhpS-MM | 1448A-WT-MM | OK | 391.168 | 161.014 | -1.2806 | -2.1328 | 0.0036 | 0.0647574 |
| PSPPH_0317 | hypothetical protein | chromosome:359418-361411 | 1448A-rhpS-MM | 1448A-WT-MM | OK | 224.94 | 92.4427 | -1.28291 | -1.34466 | 0.0913 | 0.418 |
| PSPPH_1085 | PsiE family protein | chromosome:1278185-1278674 | 1448A-rhpS-MM | 1448A-WT-MM | OK | 250.281 | 102.69 | -1.28525 | -1.31407 | 0.07675 | 0.385653 |
| PSPPH_1820 | ankyrin domain-containing protein | chromosome:2120940-2121486 | 1448A-rhpS-MM | 1448A-WT-MM | OK | 165.073 | 67.6007 | -1.288 | -1.14113 | 0.11385 | 0.454805 |
| PSPPH_0731 | type IV pilus biogenesis protein | chromosome:854921-855326 | 1448A-rhpS-MM | 1448A-WT-MM | OK | 325.008 | 132.99 | -1.28916 | -1.28848 | 0.0811 | 0.388258 |
| gcd | quinoprotein glucose dehydrogenase | chromosome:4485636-4488054 | 1448A-rhpS-MM | 1448A-WT-MM | OK | 208.936 | 85.4059 | -1.29065 | -2.98784 | 0.0003 | 0.0108571 |
| PSPPH_2671 | spemidine/putrescine ABC transporter substrate-binding protein | chromosome:3092363-3093380 | 1448A-rhpS-MM | 1448A-WT-MM | OK | 316.865 | 129.453 | -1.29145 | -2.39445 | 0.00315 | 0.0583902 |
| PSPPH_1418 | hypothetical protein | chromosome:1643627-1643933 | 1448A-rhpS-MM | 1448A-WT-MM | OK | 146.06 | 59.6633 | -1.29165 | -0.638961 | 0.3462 | 0.680315 |
| PSPPH_4351 | acetyltransferase | chromosome:4973703-4974273 | 1448A-rhpS-MM | 1448A-WT-MM | OK | 85.9901 | 35.0833 | -1.29339 | -0.937329 | 0.2405 | 0.590565 |
| PSPPH_1947 | hypothetical protein | chromosome:2293282-2293744 | 1448A-rhpS-MM | 1448A-WT-MM | OK | 199.192 | 81.2355 | -1.29397 | -1.10784 | 0.11855 | 0.459086 |
| PSPPH_0179 | ATPase AAA | chromosome:212965-216300 | 1448A-rhpS-MM | 1448A-WT-MM | OK | 1087.21 | 442.412 | -1.29717 | -2.71413 | 0.0003 | 0.0108571 |
| PSPPH_0056 | hypothetical protein | chromosome:58484-59078 | 1448A-rhpS-MM | 1448A-WT-MM | OK | 128.296 | 52.1555 | -1.29859 | -1.14833 | 0.13315 | 0.480342 |
| PSPPH_3925 | hypothetical protein | chromosome:4484255-4484912 | 1448A-rhpS-MM | 1448A-WT-MM | NOTEST | 41.2828 | 16.7521 | -1.3012 | 0 | 1 | 1 |
| PSPPH_2106 | TetR family transcriptional regulator | chromosome:2466856-2467528 | 1448A-rhpS-MM | 1448A-WT-MM | OK | 124.591 | 50.4598 | -1.30399 | -1.11305 | 0.117 | 0.457362 |
| rnhA | ribonuclease H | chromosome:1981159-1981609 | 1448A-rhpS-MM | 1448A-WT-MM | OK | 172.79 | 69.9273 | -1.30509 | -1.01049 | 0.1604 | 0.506614 |
| PSPPH_3122 | transcriptional regulator | chromosome:3623016-3624006 | 1448A-rhpS-MM | 1448A-WT-MM | NOTEST | 45.5624 | 18.4309 | -1.30572 | 0 | 1 | 1 |
| PSPPH_3460 | LysR family transcriptional regulator | chromosome:3998152-3999064 | 1448A-rhpS-MM | 1448A-WT-MM | NOTEST | 51.8392 | 20.9693 | -1.30577 | 0 | 1 | 1 |
| PSPPH_2103 | hypothetical protein | chromosome:2462400-2462808 | 1448A-rhpS-MM | 1448A-WT-MM | NOTEST | 67.0512 | 27.1181 | -1.30601 | 0 | 1 | 1 |
| PSPPH_1052 | branched-chain amino acid ABC transporter permease | chromosome:1241701-1246332 | 1448A-rhpS-MM | 1448A-WT-MM | NOTEST | 24.5733 | 9.92858 | -1.30743 | 0 | 1 | 1 |
| osmE | DNA-binding transcriptional activator OsmE | chromosome:5123904-5124243 | 1448A-rhpS-MM | 1448A-WT-MM | OK | 580.537 | 233.293 | -1.31524 | -1.58656 | 0.04365 | 0.296585 |
| PSPPH_0727 | pre-pilin leader sequence | chromosome:849522-854910 | 1448A-rhpS-MM | 1448A-WT-MM | OK | 198.252 | 79.6298 | -1.31595 | -0.79747 | 0.31545 | 0.655033 |
| PSPPH_4861 | histidine ABC transporter substrate-binding protein | chromosome:5527150-5528119 | 1448A-rhpS-MM | 1448A-WT-MM | NOTEST | 34.4103 | 13.745 | -1.32394 | 0 | 1 | 1 |
| cheD | chemoreceptor glutamine deamidase CheD | chromosome:944616-946995 | 1448A-rhpS-MM | 1448A-WT-MM | OK | 129.515 | 51.7147 | -1.32448 | -0.975124 | 0.16045 | 0.506614 |
| PSPPH_1893 | prophage PSPPH02 chitinase | chromosome:2193798-2194344 | 1448A-rhpS-MM | 1448A-WT-MM | NOTEST | 22.8082 | 9.08664 | -1.32773 | 0 | 1 | 1 |
| PSPPH_4191 | hypothetical protein | chromosome:4780165-4780642 | 1448A-rhpS-MM | 1448A-WT-MM | OK | 539.165 | 214.632 | -1.32886 | -1.94182 | 0.01315 | 0.160546 |
| PSPPH_0017 | oxidoreductase alpha (molybdopterin) subunit, fusion | chromosome:21015-22644 | 1448A-rhpS-MM | 1448A-WT-MM | NOTEST | 10.8099 | 4.29604 | -1.33127 | 0 | 1 | 1 |
| PSPPH_2574 | hypothetical protein | chromosome:2968560-2969046 | 1448A-rhpS-MM | 1448A-WT-MM | OK | 168.634 | 66.9457 | -1.33283 | -1.13142 | 0.1237 | 0.467721 |
| PSPPH_2512 | amino acid transporter | chromosome:2898566-2899883 | 1448A-rhpS-MM | 1448A-WT-MM | NOTEST | 16.8121 | 6.65642 | -1.33668 | 0 | 1 | 1 |
| PSPPH_1894 | prophage PSPPH02 adenine modification methytransferase | chromosome:2194524-2195319 | 1448A-rhpS-MM | 1448A-WT-MM | NOTEST | 52.0903 | 20.6149 | -1.33732 | 0 | 1 | 1 |
| PSPPH_3471 | GntR family transcriptional regulator | chromosome:4010178-4010898 | 1448A-rhpS-MM | 1448A-WT-MM | OK | 102.193 | 40.3779 | -1.33966 | -1.1956 | 0.11365 | 0.454805 |
| PSPPH_4432 | pilin protein | chromosome:5064870-5065059 | 1448A-rhpS-MM | 1448A-WT-MM | OK | 513.37 | 202.828 | -1.33974 | -0.619317 | 0.3253 | 0.664591 |
| PSPPH_3751 | glyoxalase | chromosome:4303748-4304183 | 1448A-rhpS-MM | 1448A-WT-MM | OK | 86.0751 | 33.9679 | -1.34142 | -0.83223 | 0.31185 | 0.652909 |
| PSPPH_2551 | oxidoreductase | chromosome:2943021-2943606 | 1448A-rhpS-MM | 1448A-WT-MM | OK | 127.175 | 50.1287 | -1.3431 | -1.10383 | 0.129 | 0.475299 |
| PSPPH_4076 | PAP2 superfamily protein | chromosome:4658439-4659180 | 1448A-rhpS-MM | 1448A-WT-MM | OK | 286.451 | 112.721 | -1.34554 | -1.98687 | 0.0148 | 0.171576 |
| PSPPH_3006 | iron ABC transporter substrate-binding protein | chromosome:3489422-3492411 | 1448A-rhpS-MM | 1448A-WT-MM | NOTEST | 59.5502 | 23.4211 | -1.3463 | 0 | 1 | 1 |
| pfpi | protease PfpI | chromosome:5438193-5438880 | 1448A-rhpS-MM | 1448A-WT-MM | OK | 108.38 | 42.6127 | -1.34675 | -1.31496 | 0.10955 | 0.450249 |
| PSPPH_4364 | ATP-dependent helicase HrpB | chromosome:4985921-4987139 | 1448A-rhpS-MM | 1448A-WT-MM | NOTEST | 21.2024 | 8.33162 | -1.34756 | 0 | 1 | 1 |
| katE | hydroperoxidase II | chromosome:308132-310283 | 1448A-rhpS-MM | 1448A-WT-MM | OK | 131.247 | 51.4427 | -1.35125 | -2.44698 | 0.002 | 0.044058 |
| PSPPH_3796 | hypothetical protein | chromosome:4348250-4348943 | 1448A-rhpS-MM | 1448A-WT-MM | OK | 131.336 | 51.2938 | -1.3564 | -1.30956 | 0.08025 | 0.388044 |
| PSPPH_4713 | diguanylate cyclase | chromosome:5349076-5350375 | 1448A-rhpS-MM | 1448A-WT-MM | NOTEST | 27.1329 | 10.5613 | -1.36126 | 0 | 1 | 1 |
| PSPPH_3704 | NLP/P60 family protein | chromosome:4251421-4251967 | 1448A-rhpS-MM | 1448A-WT-MM | OK | 202.757 | 78.7982 | -1.36352 | -1.35745 | 0.077 | 0.386271 |
| murI | glutamate racemase | chromosome:1192054-1194460 | 1448A-rhpS-MM | 1448A-WT-MM | OK | 132.042 | 51.2965 | -1.36407 | -1.13198 | 0.13955 | 0.488174 |
| PSPPH_3298 | hypothetical protein | chromosome:3821217-3826271 | 1448A-rhpS-MM | 1448A-WT-MM | NOTEST | 25.4349 | 9.87751 | -1.36459 | 0 | 1 | 1 |
| PSPPH_1806 | Smr domain-containing protein | chromosome:2110050-2110608 | 1448A-rhpS-MM | 1448A-WT-MM | OK | 353.139 | 137.138 | -1.36461 | -1.80698 | 0.0213 | 0.207975 |
| gspG1 | general secretion pathway protein GspG | chromosome:3555055-3555487 | 1448A-rhpS-MM | 1448A-WT-MM | OK | 215.005 | 83.474 | -1.36497 | -1.22319 | 0.108 | 0.449978 |
| PSPPH_1152 | SAM-dependent methyltransferase | chromosome:1351679-1352666 | 1448A-rhpS-MM | 1448A-WT-MM | NOTEST | 62.0634 | 24.0918 | -1.3652 | 0 | 1 | 1 |
| hisI | phosphoribosyl-AMP cyclohydrolase | chromosome:422756-423149 | 1448A-rhpS-MM | 1448A-WT-MM | OK | 546.389 | 212.084 | -1.36529 | -1.74186 | 0.03055 | 0.25237 |
| PSPPH_2445 | hypothetical protein | chromosome:2826314-2827070 | 1448A-rhpS-MM | 1448A-WT-MM | OK | 285.719 | 110.52 | -1.37028 | -2.00143 | 0.01145 | 0.146869 |
| PSPPH_3433 | major facilitator superfamily transporter | chromosome:3968843-3970430 | 1448A-rhpS-MM | 1448A-WT-MM | OK | 95.3369 | 36.5491 | -1.3832 | -0.599563 | 0.4785 | 0.768021 |
| PSPPH_3481 | short chain dehydrogenase/reductase oxidoreductase | chromosome:4021197-4021986 | 1448A-rhpS-MM | 1448A-WT-MM | NOTEST | 61.3713 | 23.4669 | -1.38694 | 0 | 1 | 1 |
| algG | alginate biosynthesis protein AlgG | chromosome:1309933-1311544 | 1448A-rhpS-MM | 1448A-WT-MM | OK | 134.384 | 51.2207 | -1.39157 | -2.20135 | 0.0059 | 0.0910457 |
| PSPPH_3105 | hypothetical protein | chromosome:3603419-3605010 | 1448A-rhpS-MM | 1448A-WT-MM | OK | 132.094 | 50.2329 | -1.39486 | -1.73855 | 0.02235 | 0.214334 |
| PSPPH_5104 | hypothetical protein | chromosome:5791949-5792255 | 1448A-rhpS-MM | 1448A-WT-MM | NOTEST | 69.6318 | 26.4003 | -1.39919 | 0 | 1 | 1 |
| PSPPH_1106 | hypothetical protein | chromosome:1300916-1301381 | 1448A-rhpS-MM | 1448A-WT-MM | OK | 117.479 | 44.4666 | -1.4016 | -1.02922 | 0.1991 | 0.548217 |
| PSPPH_4740 | exonuclease | chromosome:5380943-5383522 | 1448A-rhpS-MM | 1448A-WT-MM | NOTEST | 61.8901 | 23.4108 | -1.40254 | 0 | 1 | 1 |
| PSPPH_4140 | toluene tolerance protein Ttg2F | chromosome:4727810-4728050 | 1448A-rhpS-MM | 1448A-WT-MM | OK | 395.717 | 149.004 | -1.40911 | -0.919653 | 0.2317 | 0.581538 |
| PSPPH_2622 | exopolysaccharide production protein ExoZ | chromosome:3026328-3027348 | 1448A-rhpS-MM | 1448A-WT-MM | NOTEST | 65.8824 | 24.784 | -1.41048 | 0 | 1 | 1 |
| PSPPH_2845 | hypothetical protein | chromosome:3290577-3290895 | 1448A-rhpS-MM | 1448A-WT-MM | OK | 188.962 | 71.0621 | -1.41094 | -1.08081 | 0.2525 | 0.600525 |
| PSPPH_4120 | hypothetical protein | chromosome:4706349-4708535 | 1448A-rhpS-MM | 1448A-WT-MM | OK | 116.415 | 43.7303 | -1.41257 | -0.629477 | 0.45975 | 0.76115 |
| PSPPH_3289 | dyp-type peroxidase | chromosome:3811914-3813237 | 1448A-rhpS-MM | 1448A-WT-MM | OK | 221.661 | 82.8479 | -1.41982 | -2.5674 | 0.00085 | 0.0237064 |
| PSPPH_2283 | hypothetical protein | chromosome:2649491-2650091 | 1448A-rhpS-MM | 1448A-WT-MM | OK | 109.005 | 40.7186 | -1.42064 | -1.0415 | 0.14835 | 0.4945 |
| PSPPH_2458 | urea amidolyase | chromosome:2838562-2841952 | 1448A-rhpS-MM | 1448A-WT-MM | NOTEST | 10.9343 | 4.07181 | -1.42512 | 0 | 1 | 1 |
| gspH1 | general secretion pathway protein GspH | chromosome:3550674-3555047 | 1448A-rhpS-MM | 1448A-WT-MM | OK | 109.505 | 40.7389 | -1.42652 | -0.756717 | 0.2674 | 0.61583 |
| PSPPH_1089 | hypothetical protein | chromosome:1282425-1282758 | 1448A-rhpS-MM | 1448A-WT-MM | OK | 400.874 | 149.002 | -1.42782 | -1.30553 | 0.08255 | 0.392449 |
| PSPPH_2615 | phosphoglycerate mutase | chromosome:3013597-3014308 | 1448A-rhpS-MM | 1448A-WT-MM | OK | 277.027 | 102.592 | -1.4331 | -1.97845 | 0.0141 | 0.169674 |
| PSPPH_4979 | prophage PSPPH06 reverse transcriptase/maturase | chromosome:5648996-5650433 | 1448A-rhpS-MM | 1448A-WT-MM | OK | 111.633 | 41.2722 | -1.43552 | -1.73901 | 0.02745 | 0.236397 |
| PSPPH_0749 | hypothetical protein | chromosome:873204-874737 | 1448A-rhpS-MM | 1448A-WT-MM | NOTEST | 18.9027 | 6.92636 | -1.44842 | 0 | 1 | 1 |
| PSPPH_1036 | hypothetical protein | chromosome:1224182-1224767 | 1448A-rhpS-MM | 1448A-WT-MM | OK | 657.011 | 240.256 | -1.45135 | -2.59469 | 0.00135 | 0.0333659 |
| cysI | sulfite reductase (NADPH) hemoprotein subunit beta | chromosome:3016155-3018292 | 1448A-rhpS-MM | 1448A-WT-MM | OK | 1396.02 | 509.182 | -1.45507 | -4.46538 | 5.00E-05 | 0.00230303 |
| PSPPH_4061 | 3-beta hydroxysteroid dehydrogenase/isomerase | chromosome:4642382-4643375 | 1448A-rhpS-MM | 1448A-WT-MM | OK | 312.479 | 113.95 | -1.45536 | -2.48229 | 0.0028 | 0.0538734 |
| PSPPH_4710 | hypothetical protein | chromosome:5346559-5346757 | 1448A-rhpS-MM | 1448A-WT-MM | OK | 750.658 | 273.514 | -1.45654 | -0.980987 | 0.21975 | 0.569968 |
| PSPPH_4513 | hypothetical protein | chromosome:5144823-5145246 | 1448A-rhpS-MM | 1448A-WT-MM | OK | 136.806 | 49.6094 | -1.46345 | -0.86859 | 0.197 | 0.548217 |
| PSPPH_4037 | hypothetical protein | chromosome:4611822-4612182 | 1448A-rhpS-MM | 1448A-WT-MM | OK | 1137.75 | 412.215 | -1.46472 | -2.40199 | 0.00405 | 0.0695593 |
| cysZ | sulfate transport protein CysZ | chromosome:1258550-1259312 | 1448A-rhpS-MM | 1448A-WT-MM | OK | 129.329 | 46.7688 | -1.46742 | -1.39793 | 0.0695 | 0.3744 |
| PSPPH_4931 | hypothetical protein | chromosome:5604137-5605474 | 1448A-rhpS-MM | 1448A-WT-MM | OK | 205.26 | 74.0563 | -1.47076 | -0.65241 | 0.35515 | 0.687679 |
| PSPPH_3327 | CAAX amino terminal protease | chromosome:3851432-3852224 | 1448A-rhpS-MM | 1448A-WT-MM | NOTEST | 16.4646 | 5.9174 | -1.47633 | 0 | 1 | 1 |
| algD | alginate biosynthesis protein AlgD | chromosome:1317258-1318575 | 1448A-rhpS-MM | 1448A-WT-MM | OK | 959.894 | 344.66 | -1.4777 | -4.18924 | 5.00E-05 | 0.00230303 |
| PSPPH_1474 | acetyltransferase | chromosome:1714058-1714469 | 1448A-rhpS-MM | 1448A-WT-MM | OK | 90.3977 | 32.4324 | -1.47885 | -0.691435 | 0.3036 | 0.646772 |
| PSPPH_3581 | ISPsy2, transposase | chromosome:4125947-4126928 | 1448A-rhpS-MM | 1448A-WT-MM | NOTEST | 27.4052 | 9.82374 | -1.48011 | 0 | 1 | 1 |
| PSPPH_3415 | flagellin synthesis, negative regulator, FlgM | chromosome:3947836-3948151 | 1448A-rhpS-MM | 1448A-WT-MM | OK | 556.528 | 198.995 | -1.48372 | -1.46876 | 0.06875 | 0.373882 |
| PSPPH_2087 | ISPsy18, transposase, truncated | chromosome:2448895-2449570 | 1448A-rhpS-MM | 1448A-WT-MM | NOTEST | 25.6521 | 9.16589 | -1.48473 | 0 | 1 | 1 |
| PSPPH_3928 | ABC transporter binding protein | chromosome:4489316-4491813 | 1448A-rhpS-MM | 1448A-WT-MM | OK | 88.0972 | 31.4438 | -1.48632 | -1.47186 | 0.0757 | 0.384441 |
| miaE | tRNA-(ms[2]io[6]A)-hydroxylase | chromosome:4092077-4092725 | 1448A-rhpS-MM | 1448A-WT-MM | NOTEST | 47.8524 | 17.069 | -1.48721 | 0 | 1 | 1 |
| PSPPH_1093 | RNA polymerase sigma factor | chromosome:1287130-1288608 | 1448A-rhpS-MM | 1448A-WT-MM | NOTEST | 38.3136 | 13.6419 | -1.48981 | 0 | 1 | 1 |
| PSPPH_2280 | ABC transporter permease | chromosome:2645115-2647648 | 1448A-rhpS-MM | 1448A-WT-MM | NOTEST | 12.497 | 4.44655 | -1.49083 | 0 | 1 | 1 |
| PSPPH_5181 | HemK family modification methylase | chromosome:5878502-5881096 | 1448A-rhpS-MM | 1448A-WT-MM | NOTEST | 40.592 | 14.4047 | -1.49466 | 0 | 1 | 1 |
| PSPPH_1742 | hypothetical protein | chromosome:2020877-2022595 | 1448A-rhpS-MM | 1448A-WT-MM | OK | 83.5846 | 29.4272 | -1.50609 | -0.763819 | 0.37665 | 0.700073 |
| PSPPH_2784 | FLA | chromosome:3222221-3224038 | 1448A-rhpS-MM | 1448A-WT-MM | NOTEST | 18.8827 | 6.64195 | -1.50739 | 0 | 1 | 1 |
| PSPPH_2614 | SCP-2 sterol transfer family protein | chromosome:3013224-3013542 | 1448A-rhpS-MM | 1448A-WT-MM | OK | 1459.81 | 509.86 | -1.5176 | -2.40704 | 0.00395 | 0.0686171 |
| PSPPH_1533 | SirA domain-containing protein | chromosome:1788512-1788764 | 1448A-rhpS-MM | 1448A-WT-MM | OK | 469.064 | 163.512 | -1.52039 | -1.07866 | 0.15475 | 0.503178 |
| relE | addiction module toxin RelE | chromosome:186130-186650 | 1448A-rhpS-MM | 1448A-WT-MM | OK | 373.98 | 129.777 | -1.52693 | -0.949251 | 0.1483 | 0.4945 |
| putP | sodium/proline symporter | chromosome:575966-577526 | 1448A-rhpS-MM | 1448A-WT-MM | OK | 183.467 | 63.6261 | -1.52783 | -2.56616 | 0.00295 | 0.0557019 |
| gspF1 | general secretion pathway protein GspF | chromosome:3555512-3558469 | 1448A-rhpS-MM | 1448A-WT-MM | OK | 112.365 | 38.8914 | -1.53067 | -1.71232 | 0.0314 | 0.255746 |
| gspK1 | general secretion pathway protein GspK | chromosome:3550674-3555047 | 1448A-rhpS-MM | 1448A-WT-MM | NOTEST | 48.1974 | 16.6686 | -1.53183 | 0 | 1 | 1 |
| PSPPH_5102 | hypothetical protein | chromosome:5791019-5791235 | 1448A-rhpS-MM | 1448A-WT-MM | OK | 2613.68 | 896.818 | -1.5432 | -2.11235 | 0.0172 | 0.186743 |
| PSPPH_3506 | hypothetical protein | chromosome:4045581-4046073 | 1448A-rhpS-MM | 1448A-WT-MM | OK | 98.7123 | 33.7204 | -1.54961 | -0.995167 | 0.2119 | 0.563982 |
| PSPPH_5073 | hypothetical protein | chromosome:5755795-5756146 | 1448A-rhpS-MM | 1448A-WT-MM | OK | 212.586 | 72.5212 | -1.55157 | -1.25773 | 0.1793 | 0.525038 |
| PSPPH_2770 | C4-dicarboxylate transport system permease large protein | chromosome:3206290-3208068 | 1448A-rhpS-MM | 1448A-WT-MM | NOTEST | 31.6662 | 10.796 | -1.55245 | 0 | 1 | 1 |
| rplL | 50S ribosomal protein L7/L12 | chromosome:5240684-5241050 | 1448A-rhpS-MM | 1448A-WT-MM | OK | 3195.39 | 1085.64 | -1.55745 | -3.68797 | 5.00E-05 | 0.00230303 |
| PSPPH_1810 | hypothetical protein | chromosome:2112926-2113730 | 1448A-rhpS-MM | 1448A-WT-MM | OK | 91.5389 | 30.97 | -1.56351 | -1.24401 | 0.10825 | 0.449978 |
| sdaA | L-serine ammonia-lyase | chromosome:3999193-4000570 | 1448A-rhpS-MM | 1448A-WT-MM | OK | 448.444 | 151.66 | -1.56409 | -3.5324 | 5.00E-05 | 0.00230303 |
| PSPPH_3175 | monovalent cation/H+ antiporter subunit E | chromosome:3684561-3685702 | 1448A-rhpS-MM | 1448A-WT-MM | OK | 95.5602 | 32.3078 | -1.56453 | -0.921915 | 0.3035 | 0.646772 |
| treS | trehalose synthase | chromosome:2802797-2806651 | 1448A-rhpS-MM | 1448A-WT-MM | NOTEST | 70.5885 | 23.8609 | -1.56478 | 0 | 1 | 1 |
| PSPPH_3151 | capsular polysaccharide biosynthesis protein | chromosome:3655519-3656608 | 1448A-rhpS-MM | 1448A-WT-MM | NOTEST | 10.0548 | 3.3982 | -1.56504 | 0 | 1 | 1 |
| PSPPH_0038 | hypothetical protein | chromosome:43616-43943 | 1448A-rhpS-MM | 1448A-WT-MM | OK | 631.193 | 212.899 | -1.56791 | -1.76499 | 0.03775 | 0.275204 |
| PSPPH_2975 | aliphatic sulfonate ABC transporter substrate-binding protein | chromosome:3454398-3455373 | 1448A-rhpS-MM | 1448A-WT-MM | NOTEST | 11.6141 | 3.9029 | -1.57326 | 0 | 1 | 1 |
| PSPPH_4418 | type IV leader peptidase | chromosome:5043759-5044233 | 1448A-rhpS-MM | 1448A-WT-MM | OK | 94.1854 | 31.5094 | -1.57972 | -1.04369 | 0.2379 | 0.587503 |
| PSPPH_3393 | 3-oxoacyl-ACP synthase | chromosome:3921036-3921963 | 1448A-rhpS-MM | 1448A-WT-MM | OK | 868.29 | 290.067 | -1.58179 | -3.80367 | 5.00E-05 | 0.00230303 |
| PSPPH_4857 | TonB-dependent receptor | chromosome:5519037-5521353 | 1448A-rhpS-MM | 1448A-WT-MM | NOTEST | 4.26616 | 1.42376 | -1.58324 | 0 | 1 | 1 |
| cobT | nicotinate-nucleotide--dimethylbenzimidazole phosphoribosyltransferase | chromosome:4241334-4243474 | 1448A-rhpS-MM | 1448A-WT-MM | OK | 114.779 | 38.288 | -1.5839 | -1.54531 | 0.0959 | 0.428729 |
| PSPPH_3143 | low molecular weight protein-tyrosine-phosphatase | chromosome:3645096-3646438 | 1448A-rhpS-MM | 1448A-WT-MM | NOTEST | 48.0775 | 15.9642 | -1.59052 | 0 | 1 | 1 |
| PSPPH_4676 | hypothetical protein | chromosome:5312839-5315217 | 1448A-rhpS-MM | 1448A-WT-MM | NOTEST | 64.0986 | 21.1299 | -1.60101 | 0 | 1 | 1 |
| pyrR | bifunctional pyrimidine regulatory protein PyrR/uracil phosphoribosyltransferase | chromosome:537853-538366 | 1448A-rhpS-MM | 1448A-WT-MM | OK | 442.941 | 145.919 | -1.60195 | -2.13947 | 0.01045 | 0.138725 |
| PSPPH_3008 | iron ABC transporter ATP-binding protein | chromosome:3489422-3492411 | 1448A-rhpS-MM | 1448A-WT-MM | NOTEST | 38.6081 | 12.6912 | -1.60507 | 0 | 1 | 1 |
| PSPPH_0039 | hypothetical protein | chromosome:44109-44325 | 1448A-rhpS-MM | 1448A-WT-MM | OK | 454.696 | 149.058 | -1.60903 | -1.32526 | 0.24205 | 0.592702 |
| PSPPH_3960 | hypothetical protein | chromosome:4522407-4523229 | 1448A-rhpS-MM | 1448A-WT-MM | OK | 143.927 | 47.0709 | -1.61243 | -1.67163 | 0.03275 | 0.261312 |
| PSPPH_0977 | glycosyl transferase family protein | chromosome:1165357-1166941 | 1448A-rhpS-MM | 1448A-WT-MM | NOTEST | 25.1732 | 8.19713 | -1.6187 | 0 | 1 | 1 |
| PSPPH_3237 | hypothetical protein | chromosome:3752434-3752761 | 1448A-rhpS-MM | 1448A-WT-MM | OK | 505.135 | 163.89 | -1.62394 | -1.60963 | 0.0429 | 0.294736 |
| PSPPH_3023 | abrB protein | chromosome:3509236-3510325 | 1448A-rhpS-MM | 1448A-WT-MM | OK | 732.925 | 237.461 | -1.62598 | -3.87832 | 5.00E-05 | 0.00230303 |
| PSPPH_2485 | HypX | chromosome:2869905-2870598 | 1448A-rhpS-MM | 1448A-WT-MM | NOTEST | 27.4391 | 8.85982 | -1.63088 | 0 | 1 | 1 |
| nhaA | pH-dependent sodium/proton antiporter | chromosome:4827571-4828750 | 1448A-rhpS-MM | 1448A-WT-MM | NOTEST | 14.4429 | 4.65918 | -1.63221 | 0 | 1 | 1 |
| PSPPH_4790 | ferredoxin, 4Fe-4S | chromosome:5435734-5435986 | 1448A-rhpS-MM | 1448A-WT-MM | OK | 440.308 | 141.832 | -1.63433 | -1.56218 | 0.12645 | 0.472246 |
| ruvC | Holliday junction resolvase | chromosome:4323525-4324050 | 1448A-rhpS-MM | 1448A-WT-MM | OK | 125.277 | 40.1931 | -1.6401 | -1.14717 | 0.11015 | 0.451897 |
| PSPPH_4871 | hypothetical protein | chromosome:5539519-5539969 | 1448A-rhpS-MM | 1448A-WT-MM | OK | 127.661 | 40.7668 | -1.64685 | -1.06944 | 0.14765 | 0.494335 |
| PSPPH_3027 | sugar diacide regulator | chromosome:3514775-3515195 | 1448A-rhpS-MM | 1448A-WT-MM | NOTEST | 44.8145 | 14.2658 | -1.6514 | 0 | 1 | 1 |
| amt1 | ammonium transporter | chromosome:3383005-3384331 | 1448A-rhpS-MM | 1448A-WT-MM | NOTEST | 39.4904 | 12.5338 | -1.65567 | 0 | 1 | 1 |
| ampE | inner membrane protein AmpE | chromosome:1007823-1008660 | 1448A-rhpS-MM | 1448A-WT-MM | OK | 360.904 | 114.517 | -1.65605 | -2.62226 | 0.00245 | 0.0496533 |
| PSPPH_4656 | lipoprotein | chromosome:5293755-5294322 | 1448A-rhpS-MM | 1448A-WT-MM | OK | 254.123 | 80.4778 | -1.65886 | -1.76492 | 0.0329 | 0.261409 |
| PSPPH_3262 | hypothetical protein | chromosome:3781441-3781702 | 1448A-rhpS-MM | 1448A-WT-MM | OK | 746.44 | 235.573 | -1.66385 | -1.53623 | 0.0702 | 0.3744 |
| PSPPH_4438 | type II secretion system protein F domain | chromosome:5070960-5071845 | 1448A-rhpS-MM | 1448A-WT-MM | NOTEST | 36.9077 | 11.6157 | -1.66785 | 0 | 1 | 1 |
| PSPPH_4122 | lipoprotein | chromosome:4708710-4709873 | 1448A-rhpS-MM | 1448A-WT-MM | OK | 661.037 | 207.966 | -1.66839 | -2.71039 | 0.0018 | 0.0405333 |
| PSPPH_4652 | acyl dehydratase | chromosome:5291049-5291907 | 1448A-rhpS-MM | 1448A-WT-MM | NOTEST | 73.6854 | 23.1342 | -1.67135 | 0 | 1 | 1 |
| PSPPH_2228 | hypothetical protein | chromosome:2589062-2589428 | 1448A-rhpS-MM | 1448A-WT-MM | OK | 7665.68 | 2406.49 | -1.67148 | -4.9505 | 5.00E-05 | 0.00230303 |
| PSPPH_1986 | lipoprotein | chromosome:2335199-2335460 | 1448A-rhpS-MM | 1448A-WT-MM | OK | 1908.58 | 594.366 | -1.68308 | -2.43771 | 0.0066 | 0.0998209 |
| PSPPH_0514 | lipopolysaccharide core biosynthesis protein | chromosome:597387-603334 | 1448A-rhpS-MM | 1448A-WT-MM | OK | 143.541 | 44.617 | -1.6858 | -1.22447 | 0.11405 | 0.454805 |
| ccmB | heme exporter protein CcmB | chromosome:3835325-3836635 | 1448A-rhpS-MM | 1448A-WT-MM | NOTEST | 53.9619 | 16.7419 | -1.68848 | 0 | 1 | 1 |
| PSPPH_4727 | ABC transporter permease | chromosome:5363953-5368805 | 1448A-rhpS-MM | 1448A-WT-MM | NOTEST | 19.601 | 6.03928 | -1.69848 | 0 | 1 | 1 |
| PSPPH_4230 | methyl-accepting chemotaxis protein | chromosome:4825705-4827010 | 1448A-rhpS-MM | 1448A-WT-MM | NOTEST | 23.3097 | 7.16568 | -1.70176 | 0 | 1 | 1 |
| PSPPH_0668 | prophage PSPPH01, DNA-binding protein | chromosome:784729-785664 | 1448A-rhpS-MM | 1448A-WT-MM | OK | 245.052 | 75.2809 | -1.70273 | -1.23257 | 0.46275 | 0.76115 |
| PSPPH_1073 | hypothetical protein | chromosome:1263277-1263844 | 1448A-rhpS-MM | 1448A-WT-MM | OK | 75.4684 | 23.1249 | -1.70643 | -1.03847 | 0.19735 | 0.548217 |
| PSPPH_2993 | diguanylate cyclase | chromosome:3475471-3476386 | 1448A-rhpS-MM | 1448A-WT-MM | NOTEST | 15.5213 | 4.74724 | -1.70909 | 0 | 1 | 1 |
| PSPPH_4274 | F-box domain-containing protein | chromosome:4881440-4883473 | 1448A-rhpS-MM | 1448A-WT-MM | NOTEST | 33.1945 | 10.1103 | -1.71511 | 0 | 1 | 1 |
| PSPPH_0311 | hypothetical protein | chromosome:353292-353874 | 1448A-rhpS-MM | 1448A-WT-MM | OK | 119.098 | 36.2435 | -1.71636 | -1.21179 | 0.10495 | 0.446221 |
| PSPPH_4184 | osmotically inducible protein | chromosome:4771216-4771570 | 1448A-rhpS-MM | 1448A-WT-MM | OK | 1822.8 | 554.202 | -1.71767 | -3.20977 | 0.0003 | 0.0108571 |
| PSPPH_1464 | lipoprotein | chromosome:1707098-1707962 | 1448A-rhpS-MM | 1448A-WT-MM | OK | 155.024 | 47.1256 | -1.7179 | -1.82435 | 0.0293 | 0.245377 |
| bioD | dithiobiotin synthetase | chromosome:5354539-5355220 | 1448A-rhpS-MM | 1448A-WT-MM | OK | 197.065 | 59.0169 | -1.73947 | -1.71914 | 0.02845 | 0.242011 |
| PSPPH_2848 | XpsI | chromosome:3292197-3292593 | 1448A-rhpS-MM | 1448A-WT-MM | NOTEST | 48.6382 | 14.5611 | -1.73997 | 0 | 1 | 1 |
| PSPPH_2805 | polysaccharide deacetylase | chromosome:3246757-3252777 | 1448A-rhpS-MM | 1448A-WT-MM | NOTEST | 21.9322 | 6.55984 | -1.74132 | 0 | 1 | 1 |
| PSPPH_0282 | amino acid ABC transporter ATP-binding protein | chromosome:326113-328177 | 1448A-rhpS-MM | 1448A-WT-MM | OK | 89.764 | 26.8212 | -1.74277 | -1.31484 | 0.0981 | 0.433277 |
| ureE | urease accessory protein UreE | chromosome:5132789-5133961 | 1448A-rhpS-MM | 1448A-WT-MM | NOTEST | 46.482 | 13.7597 | -1.75623 | 0 | 1 | 1 |
| PSPPH_0522 | hypothetical protein | chromosome:609739-614913 | 1448A-rhpS-MM | 1448A-WT-MM | NOTEST | 55.0871 | 16.2955 | -1.75724 | 0 | 1 | 1 |
| phoB | phosphate regulon transcriptional regulatory protein PhoB | chromosome:5799448-5800138 | 1448A-rhpS-MM | 1448A-WT-MM | OK | 194.922 | 57.5491 | -1.76003 | -1.8141 | 0.02615 | 0.230423 |
| fliJ | flagellar biosynthesis chaperone | chromosome:3906555-3907005 | 1448A-rhpS-MM | 1448A-WT-MM | NOTEST | 39.401 | 11.5998 | -1.76413 | 0 | 1 | 1 |
| PSPPH_2274 | methyl-accepting chemotaxis protein | chromosome:2639718-2641647 | 1448A-rhpS-MM | 1448A-WT-MM | NOTEST | 23.07 | 6.79067 | -1.76439 | 0 | 1 | 1 |
| PSPPH_0414 | thioester dehydrase | chromosome:476539-481262 | 1448A-rhpS-MM | 1448A-WT-MM | OK | 108.136 | 31.612 | -1.7743 | -0.738109 | 0.2889 | 0.636083 |
| hisE | phosphoribosyl-ATP pyrophosphatase | chromosome:422421-422754 | 1448A-rhpS-MM | 1448A-WT-MM | OK | 575.163 | 167.427 | -1.78044 | -1.76109 | 0.0407 | 0.286097 |
| PSPPH_5194 | hypothetical protein | chromosome:5895914-5896370 | 1448A-rhpS-MM | 1448A-WT-MM | OK | 98.6606 | 28.6235 | -1.78528 | -0.850745 | 0.2035 | 0.553617 |
| PSPPH_0985 | hypothetical protein | chromosome:1177966-1179100 | 1448A-rhpS-MM | 1448A-WT-MM | NOTEST | 45.6236 | 13.2003 | -1.78921 | 0 | 1 | 1 |
| PSPPH_4554 | arginine aminomutase | chromosome:5193346-5194504 | 1448A-rhpS-MM | 1448A-WT-MM | OK | 178.109 | 51.1584 | -1.79972 | -2.26714 | 0.01135 | 0.146203 |
| holB | DNA polymerase III subunit delta' | chromosome:1908436-1910048 | 1448A-rhpS-MM | 1448A-WT-MM | OK | 199.996 | 57.2929 | -1.80354 | -2.10342 | 0.01505 | 0.172 |
| PSPPH_3795 | hypothetical protein | chromosome:4347775-4348237 | 1448A-rhpS-MM | 1448A-WT-MM | OK | 128.935 | 36.8695 | -1.80614 | -1.31758 | 0.1525 | 0.499569 |
| PSPPH_2800 | PbsX family transcriptional regulator | chromosome:3241092-3241329 | 1448A-rhpS-MM | 1448A-WT-MM | OK | 359.473 | 102.793 | -1.80614 | -1.36506 | 0.22415 | 0.57455 |
| PSPPH_5128 | hypothetical protein | chromosome:5816765-5817011 | 1448A-rhpS-MM | 1448A-WT-MM | OK | 492.124 | 140.414 | -1.80933 | -1.21986 | 0.1293 | 0.475299 |
| PSPPH_0283 | amino acid ABC transporter permease | chromosome:326113-328177 | 1448A-rhpS-MM | 1448A-WT-MM | NOTEST | 27.6172 | 7.853 | -1.81425 | 0 | 1 | 1 |
| PSPPH_3780 | hypothetical protein | chromosome:4329400-4330035 | 1448A-rhpS-MM | 1448A-WT-MM | OK | 282.487 | 79.223 | -1.83419 | -0.944049 | 0.35215 | 0.686241 |
| PSPPH_4050 | hypothetical protein | chromosome:4633179-4633761 | 1448A-rhpS-MM | 1448A-WT-MM | NOTEST | 68.0523 | 19.074 | -1.83504 | 0 | 1 | 1 |
| folK2 | 2-amino-4-hydroxy-6- hydroxymethyldihydropteridine pyrophosphokinase | chromosome:1022574-1024549 | 1448A-rhpS-MM | 1448A-WT-MM | OK | 118.903 | 33.3053 | -1.83596 | -1.00597 | 0.1695 | 0.515796 |
| PSPPH_0432 | PAAR motif-containing protein | chromosome:494770-495163 | 1448A-rhpS-MM | 1448A-WT-MM | NOTEST | 71.8204 | 19.9555 | -1.84761 | 0 | 1 | 1 |
| PSPPH_3277 | hypothetical protein | chromosome:3799097-3799316 | 1448A-rhpS-MM | 1448A-WT-MM | OK | 216.411 | 59.7143 | -1.85763 | -1.25885 | 0.3661 | 0.694073 |
| PSPPH_2698 | peptide ABC transporter ATP-binding protein | chromosome:3124712-3126699 | 1448A-rhpS-MM | 1448A-WT-MM | NOTEST | 14.8434 | 4.08788 | -1.8604 | 0 | 1 | 1 |
| PSPPH_2561 | hypothetical protein | chromosome:2953784-2954075 | 1448A-rhpS-MM | 1448A-WT-MM | OK | 314.657 | 86.6398 | -1.86068 | -1.65419 | 0.1435 | 0.494043 |
| PSPPH_4226 | tail tape meausure protein, truncated | chromosome:4819556-4820942 | 1448A-rhpS-MM | 1448A-WT-MM | NOTEST | 20.3366 | 5.58079 | -1.86553 | 0 | 1 | 1 |
| PSPPH_1467 | hypothetical protein | chromosome:1709584-1709773 | 1448A-rhpS-MM | 1448A-WT-MM | OK | 744.026 | 202.243 | -1.87926 | -1.76355 | 0.2088 | 0.560735 |
| PSPPH_2066 | CmpX | chromosome:2421642-2422467 | 1448A-rhpS-MM | 1448A-WT-MM | OK | 985.997 | 267.386 | -1.88266 | -4.22824 | 5.00E-05 | 0.00230303 |
| PSPPH_2082 | hypothetical protein | chromosome:2445104-2445449 | 1448A-rhpS-MM | 1448A-WT-MM | OK | 556.91 | 150.771 | -1.88508 | -1.82499 | 0.0362 | 0.271961 |
| PSPPH_2777 | zinc-binding oxidoreductase, truncated | chromosome:3215108-3215750 | 1448A-rhpS-MM | 1448A-WT-MM | NOTEST | 23.775 | 6.43002 | -1.88655 | 0 | 1 | 1 |
| PSPPH_4480 | hypothetical protein | chromosome:5116578-5116833 | 1448A-rhpS-MM | 1448A-WT-MM | OK | 432.27 | 116.648 | -1.88977 | -1.2599 | 0.12585 | 0.471163 |
| moaD | molybdopterin converting factor subunit 1 | chromosome:1323596-1324324 | 1448A-rhpS-MM | 1448A-WT-MM | OK | 469.889 | 126.731 | -1.89055 | -1.46684 | 0.2138 | 0.566293 |
| PSPPH_0458 | hypothetical protein | chromosome:521613-522069 | 1448A-rhpS-MM | 1448A-WT-MM | NOTEST | 58.7547 | 15.806 | -1.89423 | 0 | 1 | 1 |
| PSPPH_1104 | HlyD family secretion protein | chromosome:1295168-1299907 | 1448A-rhpS-MM | 1448A-WT-MM | NOTEST | 15.4236 | 4.1427 | -1.89649 | 0 | 1 | 1 |
| PSPPH_0420 | hypothetical protein | chromosome:482647-490911 | 1448A-rhpS-MM | 1448A-WT-MM | OK | 91.8064 | 24.5664 | -1.90191 | -0.997849 | 0.2086 | 0.560735 |
| PSPPH_4541 | hypothetical protein | chromosome:5176816-5178747 | 1448A-rhpS-MM | 1448A-WT-MM | NOTEST | 20.3703 | 5.42068 | -1.90992 | 0 | 1 | 1 |
| PSPPH_2299 | glyoxalase superfamily | chromosome:2666116-2666542 | 1448A-rhpS-MM | 1448A-WT-MM | OK | 182.144 | 48.4627 | -1.91013 | -1.16821 | 0.1116 | 0.453768 |
| PSPPH_0973 | hypothetical protein | chromosome:1159798-1161253 | 1448A-rhpS-MM | 1448A-WT-MM | NOTEST | 38.321 | 10.1864 | -1.91149 | 0 | 1 | 1 |
| PSPPH_4711 | hypothetical protein | chromosome:5346761-5347043 | 1448A-rhpS-MM | 1448A-WT-MM | OK | 4477.88 | 1188.18 | -1.91406 | -4.0844 | 5.00E-05 | 0.00230303 |
| PSPPH_0364 | 16S ribosomal RNA methyltransferase RsmE | chromosome:420134-422097 | 1448A-rhpS-MM | 1448A-WT-MM | OK | 120.859 | 32.0389 | -1.91542 | -1.37191 | 0.11705 | 0.457362 |
| PSPPH_4724 | Tat pathway signal sequence domain-containing protein | chromosome:5360907-5362824 | 1448A-rhpS-MM | 1448A-WT-MM | NOTEST | 23.3201 | 6.16752 | -1.91881 | 0 | 1 | 1 |
| PSPPH_2786 | ABC transporter substrate-binding protein | chromosome:3224066-3225958 | 1448A-rhpS-MM | 1448A-WT-MM | NOTEST | 8.65717 | 2.28504 | -1.92167 | 0 | 1 | 1 |
| PSPPH_2091 | transmembrane protein | chromosome:2451969-2452284 | 1448A-rhpS-MM | 1448A-WT-MM | OK | 492.941 | 129.453 | -1.92899 | -1.57327 | 0.06 | 0.344725 |
| PSPPH_2745 | dipeptide transporter dppD-like protein | chromosome:3175454-3178867 | 1448A-rhpS-MM | 1448A-WT-MM | NOTEST | 52.4255 | 13.7426 | -1.93162 | 0 | 1 | 1 |
| ppiC2 | peptidyl-prolyl cis-trans isomerase C | chromosome:2700509-2700791 | 1448A-rhpS-MM | 1448A-WT-MM | OK | 281.062 | 73.6327 | -1.93247 | -1.51897 | 0.17155 | 0.51686 |
| nasT | response regulator NasT | chromosome:2434442-2435018 | 1448A-rhpS-MM | 1448A-WT-MM | OK | 86.8148 | 22.6318 | -1.93959 | -1.22027 | 0.1705 | 0.516255 |
| PSPPH_2827 | hypothetical protein | chromosome:3272940-3273168 | 1448A-rhpS-MM | 1448A-WT-MM | OK | 619.849 | 160.944 | -1.94536 | -1.30926 | 0.1236 | 0.467721 |
| PSPPH_0300 | hypothetical protein | chromosome:343119-343569 | 1448A-rhpS-MM | 1448A-WT-MM | OK | 159.482 | 41.3422 | -1.94771 | -1.44673 | 0.10335 | 0.443137 |
| fecE | iron-dicitrate transporter ATP-binding subunit | chromosome:5270613-5271405 | 1448A-rhpS-MM | 1448A-WT-MM | NOTEST | 18.4046 | 4.76932 | -1.94821 | 0 | 1 | 1 |
| mdcA | malonate decarboxylase subunit alpha | chromosome:495425-497977 | 1448A-rhpS-MM | 1448A-WT-MM | OK | 223.009 | 57.726 | -1.94981 | -3.37459 | 0.00035 | 0.0122299 |
| fliL | flagellar basal body protein FliL | chromosome:3901527-3902028 | 1448A-rhpS-MM | 1448A-WT-MM | NOTEST | 53.5089 | 13.7597 | -1.95933 | 0 | 1 | 1 |
| PSPPH_2035 | hypothetical protein | chromosome:2388432-2388762 | 1448A-rhpS-MM | 1448A-WT-MM | OK | 630.02 | 161.509 | -1.96378 | -1.89276 | 0.0356 | 0.269214 |
| PSPPH_4053 | hypothetical protein | chromosome:4633842-4637746 | 1448A-rhpS-MM | 1448A-WT-MM | NOTEST | 14.6313 | 3.71723 | -1.97675 | 0 | 1 | 1 |
| PSPPH_4659 | LrgA family protein | chromosome:5294967-5296160 | 1448A-rhpS-MM | 1448A-WT-MM | NOTEST | 45.5881 | 11.5689 | -1.97841 | 0 | 1 | 1 |
| PSPPH_4549 | hypothetical protein | chromosome:5187222-5188266 | 1448A-rhpS-MM | 1448A-WT-MM | OK | 314.042 | 79.508 | -1.98179 | -2.79486 | 0.00135 | 0.0333659 |
| PSPPH_0795 | secretion protein | chromosome:940272-943330 | 1448A-rhpS-MM | 1448A-WT-MM | NOTEST | 14.4027 | 3.63725 | -1.98542 | 0 | 1 | 1 |
| PSPPH_2373 | acyl-CoA dehydrogenase | chromosome:2751110-2752325 | 1448A-rhpS-MM | 1448A-WT-MM | NOTEST | 11.3104 | 2.85326 | -1.98697 | 0 | 1 | 1 |
| PSPPH_1784 | hypothetical protein | chromosome:2090031-2090826 | 1448A-rhpS-MM | 1448A-WT-MM | OK | 91.7047 | 23.1309 | -1.98717 | -1.40892 | 0.088 | 0.410817 |
| allA | ureidoglycolate hydrolase | chromosome:2064366-2064879 | 1448A-rhpS-MM | 1448A-WT-MM | NOTEST | 52.8277 | 13.3003 | -1.98983 | 0 | 1 | 1 |
| sfsA | sugar fermentation stimulation protein A | chromosome:1032293-1034179 | 1448A-rhpS-MM | 1448A-WT-MM | NOTEST | 58.6222 | 14.6949 | -1.99614 | 0 | 1 | 1 |
| PSPPH_1586 | diguanylate phosphodiesterase | chromosome:1838745-1840578 | 1448A-rhpS-MM | 1448A-WT-MM | NOTEST | 30.7152 | 7.68113 | -1.99956 | 0 | 1 | 1 |
| PSPPH_0914 | TonB-dependent receptor | chromosome:1087524-1089675 | 1448A-rhpS-MM | 1448A-WT-MM | NOTEST | 25.5336 | 6.37085 | -2.00284 | 0 | 1 | 1 |
| PSPPH_2094 | hypothetical protein | chromosome:2453654-2453897 | 1448A-rhpS-MM | 1448A-WT-MM | OK | 178.724 | 44.5555 | -2.00406 | -1.01494 | 0.311 | 0.652909 |
| PSPPH_4386 | hypothetical protein | chromosome:5010032-5010425 | 1448A-rhpS-MM | 1448A-WT-MM | NOTEST | 69.5528 | 17.2864 | -2.00847 | 0 | 1 | 1 |
| aspA | aspartate ammonia-lyase | chromosome:5821190-5822615 | 1448A-rhpS-MM | 1448A-WT-MM | OK | 204.84 | 50.6454 | -2.01599 | -3.13179 | 0.001 | 0.0266667 |
| PSPPH_2789 | hypothetical protein | chromosome:3227654-3228959 | 1448A-rhpS-MM | 1448A-WT-MM | NOTEST | 16.8068 | 4.14916 | -2.01815 | 0 | 1 | 1 |
| PSPPH_2762 | hypothetical protein | chromosome:3196863-3199066 | 1448A-rhpS-MM | 1448A-WT-MM | OK | 122.544 | 29.9681 | -2.03181 | -1.53776 | 0.18625 | 0.535161 |
| PSPPH_0100 | recombinase | chromosome:111137-114579 | 1448A-rhpS-MM | 1448A-WT-MM | NOTEST | 39.3329 | 9.60082 | -2.03451 | 0 | 1 | 1 |
| PSPPH_5171 | LuxR family transcriptional regulator | chromosome:5866033-5867706 | 1448A-rhpS-MM | 1448A-WT-MM | OK | 98.4 | 23.9114 | -2.04096 | -1.10703 | 0.13215 | 0.477688 |
| PSPPH_5023 | hypothetical protein | chromosome:5690780-5692085 | 1448A-rhpS-MM | 1448A-WT-MM | NOTEST | 34.3852 | 8.29833 | -2.05089 | 0 | 1 | 1 |
| PSPPH_4321 | hypothetical protein | chromosome:4936397-4937788 | 1448A-rhpS-MM | 1448A-WT-MM | OK | 163.113 | 39.246 | -2.05526 | -1.48937 | 0.0714 | 0.378806 |
| PSPPH_3609 | sulfonate ABC transporter permease | chromosome:4154681-4155539 | 1448A-rhpS-MM | 1448A-WT-MM | NOTEST | 20.4905 | 4.88443 | -2.06869 | 0 | 1 | 1 |
| cysC1 | adenylylsulfate kinase | chromosome:4912788-4913394 | 1448A-rhpS-MM | 1448A-WT-MM | NOTEST | 44.3215 | 10.565 | -2.06872 | 0 | 1 | 1 |
| PSPPH_2307 | hypothetical protein | chromosome:2674534-2675227 | 1448A-rhpS-MM | 1448A-WT-MM | NOTEST | 26.9209 | 6.40522 | -2.07141 | 0 | 1 | 1 |
| PSPPH_3567 | hypothetical protein | chromosome:4104748-4110605 | 1448A-rhpS-MM | 1448A-WT-MM | NOTEST | 41.8938 | 9.85625 | -2.08763 | 0 | 1 | 1 |
| PSPPH_4426 | esterase | chromosome:5053214-5055400 | 1448A-rhpS-MM | 1448A-WT-MM | NOTEST | 50.7262 | 11.6732 | -2.11953 | 0 | 1 | 1 |
| PSPPH_3136 | cysteine transporter | chromosome:3637121-3638237 | 1448A-rhpS-MM | 1448A-WT-MM | NOTEST | 54.8071 | 12.5994 | -2.12101 | 0 | 1 | 1 |
| PSPPH_4331 | hypothetical protein | chromosome:4952053-4952428 | 1448A-rhpS-MM | 1448A-WT-MM | OK | 455.275 | 104.246 | -2.12674 | -1.8812 | 0.0349 | 0.26638 |
| PSPPH_0521 | Mig-14 family protein | chromosome:609739-614913 | 1448A-rhpS-MM | 1448A-WT-MM | OK | 99.7923 | 22.8001 | -2.12989 | -1.19426 | 0.1312 | 0.477091 |
| PSPPH_2997 | hypothetical protein | chromosome:3479003-3479672 | 1448A-rhpS-MM | 1448A-WT-MM | NOTEST | 31.637 | 7.13481 | -2.14867 | 0 | 1 | 1 |
| rluE | ribosomal large subunit pseudouridine synthase E | chromosome:4999804-5000437 | 1448A-rhpS-MM | 1448A-WT-MM | OK | 103.346 | 23.2229 | -2.15385 | -1.44317 | 0.0936 | 0.420923 |
| ppqD | pyrroloquinoline quinone biosynthesis protein PqqD | chromosome:5342487-5346475 | 1448A-rhpS-MM | 1448A-WT-MM | OK | 293.997 | 65.4923 | -2.16641 | -1.05588 | 0.25295 | 0.600525 |
| PSPPH_3333 | hypothetical protein | chromosome:3860620-3862501 | 1448A-rhpS-MM | 1448A-WT-MM | NOTEST | 9.37922 | 2.05748 | -2.18859 | 0 | 1 | 1 |
| PSPPH_4249 | hypothetical protein | chromosome:4848142-4848382 | 1448A-rhpS-MM | 1448A-WT-MM | OK | 243.043 | 53.1807 | -2.19224 | -1.34345 | 0.28105 | 0.628438 |
| PSPPH_3167 | alkanesulfonate monooxygenase | chromosome:3674007-3676253 | 1448A-rhpS-MM | 1448A-WT-MM | NOTEST | 66.8407 | 14.5529 | -2.19942 | 0 | 1 | 1 |
| PSPPH_4830 | cation efflux family protein | chromosome:5483331-5484585 | 1448A-rhpS-MM | 1448A-WT-MM | NOTEST | 12.8028 | 2.78363 | -2.20142 | 0 | 1 | 1 |
| sodC | superoxide dismutase, Cu-Zn | chromosome:1432195-1432714 | 1448A-rhpS-MM | 1448A-WT-MM | OK | 120.922 | 26.1638 | -2.20843 | -1.46019 | 0.13025 | 0.476462 |
| PSPPH_4049 | cardiolipin synthase | chromosome:4632020-4633178 | 1448A-rhpS-MM | 1448A-WT-MM | NOTEST | 29.7583 | 6.35738 | -2.22679 | 0 | 1 | 1 |
| PSPPH_1873 | hypothetical protein | chromosome:2174904-2175189 | 1448A-rhpS-MM | 1448A-WT-MM | OK | 266.697 | 56.1746 | -2.24721 | -1.43688 | 0.17545 | 0.522119 |
| PSPPH_0272 | cytochrome C oxidase assembly protein | chromosome:313945-314434 | 1448A-rhpS-MM | 1448A-WT-MM | OK | 161.895 | 33.5352 | -2.27131 | -1.53738 | 0.09125 | 0.418 |
| PSPPH_4373 | hypothetical protein | chromosome:4995108-4995801 | 1448A-rhpS-MM | 1448A-WT-MM | NOTEST | 35.9889 | 7.24043 | -2.3134 | 0 | 1 | 1 |
| PSPPH_1026 | hypothetical protein | chromosome:1216797-1217031 | 1448A-rhpS-MM | 1448A-WT-MM | OK | 227.121 | 45.6704 | -2.31413 | -1.1835 | 0.29285 | 0.637725 |
| PSPPH_2843 | hypothetical protein | chromosome:3289834-3290393 | 1448A-rhpS-MM | 1448A-WT-MM | OK | 214.045 | 42.507 | -2.33214 | -1.60756 | 0.07385 | 0.382824 |
| PSPPH_0883 | pectate lyase L | chromosome:1054118-1055243 | 1448A-rhpS-MM | 1448A-WT-MM | NOTEST | 43.2586 | 8.56705 | -2.33612 | 0 | 1 | 1 |
| PSPPH_3462 | amino acid transporter LysE | chromosome:4000583-4001225 | 1448A-rhpS-MM | 1448A-WT-MM | NOTEST | 50.0814 | 9.78565 | -2.35553 | 0 | 1 | 1 |
| PSPPH_2188 | DeoR family transcriptional regulator | chromosome:2544642-2545479 | 1448A-rhpS-MM | 1448A-WT-MM | NOTEST | 47.4476 | 9.25199 | -2.3585 | 0 | 1 | 1 |
| cheW2 | chemotaxis protein CheW | chromosome:3881963-3882443 | 1448A-rhpS-MM | 1448A-WT-MM | OK | 267.473 | 51.7898 | -2.36865 | -1.86366 | 0.04175 | 0.290435 |
| bdh2 | 3-oxoacyl-ACP reductase | chromosome:4550864-4551590 | 1448A-rhpS-MM | 1448A-WT-MM | NOTEST | 38.1471 | 7.34254 | -2.37722 | 0 | 1 | 1 |
| PSPPH_4921 | hypothetical protein | chromosome:5594093-5594903 | 1448A-rhpS-MM | 1448A-WT-MM | NOTEST | 66.5553 | 12.8037 | -2.378 | 0 | 1 | 1 |
| PSPPH_4324 | RtrR protein | chromosome:4938654-4938885 | 1448A-rhpS-MM | 1448A-WT-MM | OK | 252.081 | 48.0522 | -2.39121 | -1.27336 | 0.2765 | 0.623099 |
| PSPPH_1199 | fimbrial protein | chromosome:1409423-1410008 | 1448A-rhpS-MM | 1448A-WT-MM | NOTEST | 43.5217 | 8.19083 | -2.40965 | 0 | 1 | 1 |
| PSPPH_0063 | DsbB family protein | chromosome:64175-64763 | 1448A-rhpS-MM | 1448A-WT-MM | NOTEST | 59.0916 | 11.0031 | -2.42505 | 0 | 1 | 1 |
| PSPPH_2089 | hypothetical protein | chromosome:2451213-2451965 | 1448A-rhpS-MM | 1448A-WT-MM | OK | 370.82 | 68.3967 | -2.43872 | -1.5651 | 0.1611 | 0.506981 |
| PSPPH_4363 | hypothetical protein | chromosome:4985441-4985747 | 1448A-rhpS-MM | 1448A-WT-MM | OK | 169.6 | 31.1875 | -2.44309 | -1.36641 | 0.26195 | 0.612089 |
| PSPPH_2482 | hypothetical protein | chromosome:2865619-2866777 | 1448A-rhpS-MM | 1448A-WT-MM | NOTEST | 64.2748 | 11.3753 | -2.49834 | 0 | 1 | 1 |
| pilP | type IV pilus biogenesis protein PilP | chromosome:446939-449747 | 1448A-rhpS-MM | 1448A-WT-MM | OK | 133.784 | 23.2157 | -2.52673 | -1.22118 | 0.17035 | 0.516255 |
| PSPPH_3053 | phospholipase/carboxylesterase | chromosome:3547549-3548251 | 1448A-rhpS-MM | 1448A-WT-MM | OK | 89.0516 | 15.4113 | -2.53065 | -1.25272 | 0.12755 | 0.474024 |
| PSPPH_0254 | N-acetylmuramoyl-L-alanine amidase | chromosome:294159-294939 | 1448A-rhpS-MM | 1448A-WT-MM | NOTEST | 63.8752 | 10.8504 | -2.55751 | 0 | 1 | 1 |
| PSPPH_2414 | receptor family ligand binding protein | chromosome:2789401-2791524 | 1448A-rhpS-MM | 1448A-WT-MM | NOTEST | 25.893 | 3.99663 | -2.69571 | 0 | 1 | 1 |
| PSPPH_1745 | methyl-accepting chemotaxis protein | chromosome:2024105-2025746 | 1448A-rhpS-MM | 1448A-WT-MM | NOTEST | 15.1061 | 2.28763 | -2.7232 | 0 | 1 | 1 |
| PSPPH_0034 | CigR | chromosome:39885-40416 | 1448A-rhpS-MM | 1448A-WT-MM | OK | 516.068 | 77.1826 | -2.74121 | -2.96297 | 0.0051 | 0.0816 |
| PSPPH_4465 | hypothetical protein | chromosome:5099418-5100328 | 1448A-rhpS-MM | 1448A-WT-MM | OK | 78.1008 | 11.644 | -2.74575 | -1.01129 | 0.2606 | 0.611159 |
| PSPPH_3934 | polysaccharide deacetylase | chromosome:4494756-4495638 | 1448A-rhpS-MM | 1448A-WT-MM | NOTEST | 59.686 | 8.75352 | -2.76946 | 0 | 1 | 1 |
| PSPPH_0897 | alkylphosphonate utilization protein PhnA | chromosome:1069138-1069480 | 1448A-rhpS-MM | 1448A-WT-MM | OK | 286.936 | 40.2304 | -2.83437 | -1.73022 | 0.1558 | 0.503178 |
| PSPPH_4500 | hypothetical protein | chromosome:5135252-5135657 | 1448A-rhpS-MM | 1448A-WT-MM | OK | 192.928 | 26.7598 | -2.84992 | -1.51811 | 0.15565 | 0.503178 |
| glpD | glycerol-3-phosphate dehydrogenase | chromosome:4459822-4461361 | 1448A-rhpS-MM | 1448A-WT-MM | OK | 1796.81 | 247.911 | -2.85754 | -8.16139 | 5.00E-05 | 0.00230303 |
| PSPPH_3645 | MarR family transcriptional regulator | chromosome:4190003-4190477 | 1448A-rhpS-MM | 1448A-WT-MM | OK | 182.69 | 24.9948 | -2.8697 | -1.61107 | 0.15555 | 0.503178 |
| PSPPH_1921 | peptidase | chromosome:2235277-2237935 | 1448A-rhpS-MM | 1448A-WT-MM | NOTEST | 21.8705 | 2.9718 | -2.87958 | 0 | 1 | 1 |
| PSPPH_3176 | monovalent cation/H+ antiporter subunit F | chromosome:3684561-3685702 | 1448A-rhpS-MM | 1448A-WT-MM | OK | 279.767 | 37.6594 | -2.89314 | -1.31029 | 0.1762 | 0.522222 |
| PSPPH_3529 | hypothetical protein | chromosome:4069213-4069480 | 1448A-rhpS-MM | 1448A-WT-MM | OK | 1146.87 | 152.623 | -2.90966 | -2.33688 | 0.0274 | 0.236397 |
| PSPPH_1725 | cation transporter | chromosome:1998651-1999770 | 1448A-rhpS-MM | 1448A-WT-MM | NOTEST | 28.9015 | 3.80572 | -2.9249 | 0 | 1 | 1 |
| PSPPH_4526 | sensory box protein | chromosome:5159385-5162295 | 1448A-rhpS-MM | 1448A-WT-MM | NOTEST | 12.8499 | 1.63105 | -2.97789 | 0 | 1 | 1 |
| PSPPH_2065 | crfX protein | chromosome:2421391-2421640 | 1448A-rhpS-MM | 1448A-WT-MM | OK | 2801.56 | 351.964 | -2.99273 | -3.31227 | 0.00375 | 0.0662791 |
| arnT | 4-amino-4-deoxy-L-arabinose transferase | chromosome:3246757-3252777 | 1448A-rhpS-MM | 1448A-WT-MM | NOTEST | 20.187 | 2.47164 | -3.02989 | 0 | 1 | 1 |
| PSPPH_5204 | DeoR family transcriptional regulator | chromosome:5908357-5909125 | 1448A-rhpS-MM | 1448A-WT-MM | OK | 101.869 | 12.2272 | -3.05854 | -1.51847 | 0.15525 | 0.503178 |
| PSPPH_5080 | hypothetical protein | chromosome:5761247-5764167 | 1448A-rhpS-MM | 1448A-WT-MM | OK | 125.432 | 14.5113 | -3.11166 | -1.4758 | 0.22345 | 0.57398 |
| PSPPH_3969 | lipoprotein | chromosome:4530709-4531201 | 1448A-rhpS-MM | 1448A-WT-MM | OK | 424.531 | 48.5721 | -3.12767 | -2.54562 | 0.0242 | 0.219606 |
| PSPPH_1471 | mismatched base pair and cruciform DNA recognition protein | chromosome:1712060-1712252 | 1448A-rhpS-MM | 1448A-WT-MM | OK | 875.81 | 99.8926 | -3.13217 | -2.66409 | 0.2447 | 0.596074 |
| PSPPH_0315 | hypothetical protein | chromosome:357399-358626 | 1448A-rhpS-MM | 1448A-WT-MM | NOTEST | 30.408 | 3.42385 | -3.15076 | 0 | 1 | 1 |
| PSPPH_2003 | sensor histidine kinase | chromosome:2351513-2353285 | 1448A-rhpS-MM | 1448A-WT-MM | OK | 238.553 | 25.542 | -3.22336 | -2.42971 | 0.05015 | 0.31828 |
| PSPPH_3355 | chemotaxis protein CheW | chromosome:3882500-3883358 | 1448A-rhpS-MM | 1448A-WT-MM | NOTEST | 47.5699 | 5.01749 | -3.24501 | 0 | 1 | 1 |
| PSPPH_0380 | 1-(5-phosphoribosyl)-5-[(5- phosphoribosylamino)methylideneamino] imidazole-4-carboxamide isomerase | chromosome:433687-434107 | 1448A-rhpS-MM | 1448A-WT-MM | OK | 175.072 | 17.9384 | -3.28682 | -1.71976 | 0.24415 | 0.595679 |
| PSPPH_0753 | DNA repair ATPase | chromosome:879827-881366 | 1448A-rhpS-MM | 1448A-WT-MM | NOTEST | 38.8872 | 3.37454 | -3.52653 | 0 | 1 | 1 |
| PSPPH_2004 | DNA-binding response regulator | chromosome:2351513-2353285 | 1448A-rhpS-MM | 1448A-WT-MM | OK | 2971.15 | 179.375 | -4.04997 | -7.5745 | 5.00E-05 | 0.00230303 |
| PSPPH_2002 | calcium-binding protein | chromosome:2350639-2351398 | 1448A-rhpS-MM | 1448A-WT-MM | OK | 23317.9 | 66.7152 | -8.44921 | -11.4208 | 5.00E-05 | 0.00230303 |

**D. List of genes downregulated in *rhpS* mutant in MM**

| gene | Annotation | locus | sample_1 | sample_2 | status | value_1 | value_2 | log2(fold_change) | test_stat | p_value | q_value |
| --- | --- | --- | --- | --- | --- | --- | --- | --- | --- | --- | --- |
| avrF | type III chaperone protein AvrF | chromosome:1477288-1477678 | 1448A-rhpS-MM | 1448A-WT-MM | OK | 11.9939 | 606.493 | 5.66012 | 1.88532 | 0.2252 | 0.575128 |
| PSPPH_0650 | autotransporting lipase | chromosome:768943-770866 | 1448A-rhpS-MM | 1448A-WT-MM | OK | 102.798 | 2148.6 | 4.3855 | 10.7724 | 5.00E-05 | 0.00230303 |
| glyA | serine hydroxymethyltransferase | chromosome:5392608-5393862 | 1448A-rhpS-MM | 1448A-WT-MM | OK | 6.87002 | 142.532 | 4.37482 | 2.31138 | 0.12715 | 0.473696 |
| hrpL | RNA polymerase sigma factor HrpL | chromosome:1505661-1506216 | 1448A-rhpS-MM | 1448A-WT-MM | OK | 45.1757 | 733.99 | 4.02214 | 3.48269 | 0.0164 | 0.181188 |
| PSPPH_1386 | amino acid ABC transporter permease | chromosome:1609057-1610478 | 1448A-rhpS-MM | 1448A-WT-MM | OK | 5.15627 | 82.081 | 3.99265 | 1.13384 | 0.11395 | 0.454805 |
| hopR1 | type III effector HopR1 | chromosome:198684-204564 | 1448A-rhpS-MM | 1448A-WT-MM | OK | 266.064 | 3309.83 | 3.63691 | 12.1181 | 5.00E-05 | 0.00230303 |
| hrpT | HrpT protein | chromosome:1492114-1494414 | 1448A-rhpS-MM | 1448A-WT-MM | OK | 134.154 | 1504.85 | 3.48766 | 1.14907 | 0.209 | 0.560777 |
| PSPPH_3438 | short chain dehydrogenase/reductase oxidoreductase | chromosome:3973088-3973775 | 1448A-rhpS-MM | 1448A-WT-MM | NOTEST | 4.61618 | 47.0276 | 3.34874 | 0 | 1 | 1 |
| hrpW1 | type III helper protein HrpW1 | chromosome:1472984-1474181 | 1448A-rhpS-MM | 1448A-WT-MM | OK | 696.389 | 6795.69 | 3.28665 | 10.6102 | 5.00E-05 | 0.00230303 |
| PSPPH_3499 | type III chaperone protein ShcF | chromosome:4040269-4040665 | 1448A-rhpS-MM | 1448A-WT-MM | OK | 131.065 | 1256.97 | 3.2616 | 3.62415 | 0.0032 | 0.0589576 |
| shcV | type III chaperone protein ShcV | chromosome:2727200-2727587 | 1448A-rhpS-MM | 1448A-WT-MM | OK | 104.161 | 957.889 | 3.20104 | 3.25314 | 0.0069 | 0.101333 |
| PSPPH_2477 | glutamine amidotransferase | chromosome:2860331-2861087 | 1448A-rhpS-MM | 1448A-WT-MM | OK | 52.7781 | 472.929 | 3.16361 | 3.5665 | 0.0025 | 0.05 |
| PSPPH_3171 | hypothetical protein | chromosome:3678541-3679465 | 1448A-rhpS-MM | 1448A-WT-MM | NOTEST | 3.53553 | 31.0103 | 3.13275 | 0 | 1 | 1 |
| PSPPH_2350 | endoribonuclease L-PSP | chromosome:2725395-2727093 | 1448A-rhpS-MM | 1448A-WT-MM | OK | 65.0668 | 569.968 | 3.13089 | 1.68711 | 0.06665 | 0.369064 |
| PSPPH_4023 | hypothetical protein | chromosome:4598545-4598782 | 1448A-rhpS-MM | 1448A-WT-MM | OK | 32.6515 | 285.724 | 3.1294 | 1.35801 | 0.2336 | 0.584481 |
| hrpB | type III secretion component protein HrpB | chromosome:1489053-1489428 | 1448A-rhpS-MM | 1448A-WT-MM | OK | 690.505 | 6018.89 | 3.12377 | 7.05695 | 5.00E-05 | 0.00230303 |
| PSPPH_1063 | hypothetical protein | chromosome:1255949-1256567 | 1448A-rhpS-MM | 1448A-WT-MM | OK | 9.6356 | 82.5666 | 3.09911 | 1.36915 | 0.25065 | 0.599981 |
| PSPPH_2692 | diguanylate cyclase | chromosome:3118997-3120053 | 1448A-rhpS-MM | 1448A-WT-MM | NOTEST | 4.95817 | 40.9171 | 3.04482 | 0 | 1 | 1 |
| soxB | sarcosine oxidase subunit beta | chromosome:5393879-5395130 | 1448A-rhpS-MM | 1448A-WT-MM | OK | 15.3678 | 125.563 | 3.03042 | 2.5607 | 0.0203 | 0.201673 |
| PSPPH_2417 | hypothetical protein | chromosome:2792903-2796835 | 1448A-rhpS-MM | 1448A-WT-MM | NOTEST | 2.80432 | 22.9126 | 3.03042 | 0 | 1 | 1 |
| PSPPH_1181 | glucose ABC transporter substrate-binding protein | chromosome:1387351-1388638 | 1448A-rhpS-MM | 1448A-WT-MM | OK | 127.756 | 1031.62 | 3.01345 | 6.58826 | 5.00E-05 | 0.00230303 |
| PSPPH_2791 | hypothetical protein | chromosome:3230333-3231464 | 1448A-rhpS-MM | 1448A-WT-MM | NOTEST | 3.67139 | 29.0911 | 2.98618 | 0 | 1 | 1 |
| PSPPH_A0033 | trbA protein, truncated | large_plasmid:29009-29924 | 1448A-rhpS-MM | 1448A-WT-MM | OK | 137.357 | 1050.13 | 2.93456 | 2.79471 | 0.0097 | 0.132321 |
| hrpA2 | type III helper protein HrpA2 | chromosome:1487608-1487935 | 1448A-rhpS-MM | 1448A-WT-MM | OK | 13074 | 96393.1 | 2.88222 | 9.87209 | 5.00E-05 | 0.00230303 |
| hrpF | type III secretion component protein HrpF | chromosome:1491470-1492113 | 1448A-rhpS-MM | 1448A-WT-MM | OK | 1139.26 | 8346.14 | 2.87301 | 4.18943 | 5.00E-05 | 0.00230303 |
| PSPPH_4765 | RNA polymerase sigma factor | chromosome:5408344-5409843 | 1448A-rhpS-MM | 1448A-WT-MM | NOTEST | 7.57488 | 55.399 | 2.87056 | 0 | 1 | 1 |
| PSPPH_5144 | LysR family transcriptional regulator | chromosome:5834697-5835633 | 1448A-rhpS-MM | 1448A-WT-MM | NOTEST | 6.58382 | 47.5558 | 2.85262 | 0 | 1 | 1 |
| hrcS | type III secretion component protein HrcS | chromosome:1496832-1497099 | 1448A-rhpS-MM | 1448A-WT-MM | OK | 66.3111 | 475.477 | 2.84205 | 1.24812 | 0.0701 | 0.3744 |
| hrpQ | type III secretion component protein HrpQ | chromosome:1499873-1502194 | 1448A-rhpS-MM | 1448A-WT-MM | OK | 198.786 | 1388.15 | 2.80388 | 4.97205 | 5.00E-05 | 0.00230303 |
| PSPPH_2465 | glutamine ABC transporter substrate-binding protein | chromosome:2845976-2846810 | 1448A-rhpS-MM | 1448A-WT-MM | OK | 15.9873 | 111.026 | 2.79589 | 2.0121 | 0.07795 | 0.386887 |
| PSPPH_2388 | hypothetical protein | chromosome:2768564-2769041 | 1448A-rhpS-MM | 1448A-WT-MM | NOTEST | 8.73874 | 60.1298 | 2.78258 | 0 | 1 | 1 |
| hrpZ1 | type III restriction system endonuclease | chromosome:1487988-1489026 | 1448A-rhpS-MM | 1448A-WT-MM | OK | 2544.46 | 17485.1 | 2.7807 | 9.59197 | 5.00E-05 | 0.00230303 |
| ugpA | glycerol-3-phosphate ABC transporter permease | chromosome:5164736-5168936 | 1448A-rhpS-MM | 1448A-WT-MM | NOTEST | 3.68079 | 25.2608 | 2.77881 | 0 | 1 | 1 |
| shcM | type III chaperone protein ShcM | chromosome:1474484-1474979 | 1448A-rhpS-MM | 1448A-WT-MM | OK | 101.834 | 694.194 | 2.76912 | 3.12713 | 0.0041 | 0.0700225 |
| ureF | urease accessory protein UreF | chromosome:5132789-5133961 | 1448A-rhpS-MM | 1448A-WT-MM | NOTEST | 5.07221 | 33.938 | 2.74222 | 0 | 1 | 1 |
| thiC | thiamine biosynthesis protein ThiC | chromosome:628825-630715 | 1448A-rhpS-MM | 1448A-WT-MM | OK | 92.2277 | 601.626 | 2.7056 | 6.33488 | 5.00E-05 | 0.00230303 |
| PSPPH_A0032 | transposase family protein, truncated | large_plasmid:26414-28783 | 1448A-rhpS-MM | 1448A-WT-MM | OK | 1514.16 | 9842.68 | 2.70053 | 1.79833 | 0.01835 | 0.191041 |
| hopAE1 | type III effector HopAE1 | chromosome:4943954-4946693 | 1448A-rhpS-MM | 1448A-WT-MM | OK | 420.095 | 2726.95 | 2.6985 | 9.16717 | 5.00E-05 | 0.00230303 |
| hopV1 | type III effector HopV1 | chromosome:2725395-2727093 | 1448A-rhpS-MM | 1448A-WT-MM | OK | 73.1728 | 471.451 | 2.68773 | 4.97635 | 5.00E-05 | 0.00230303 |
| PSPPH_2772 | LysR family transcriptional regulator | chromosome:3208093-3210186 | 1448A-rhpS-MM | 1448A-WT-MM | NOTEST | 8.20137 | 51.4545 | 2.64936 | 0 | 1 | 1 |
| PSPPH_4298 | ISPsy5, transposase truncated | chromosome:4907551-4908361 | 1448A-rhpS-MM | 1448A-WT-MM | NOTEST | 11.1363 | 69.7137 | 2.64618 | 0 | 1 | 1 |
| PSPPH_2976 | amidase | chromosome:3455433-3456969 | 1448A-rhpS-MM | 1448A-WT-MM | NOTEST | 1.8642 | 11.6601 | 2.64496 | 0 | 1 | 1 |
| PSPPH_0250 | hypothetical protein | chromosome:289393-289714 | 1448A-rhpS-MM | 1448A-WT-MM | OK | 26.6408 | 164.747 | 2.62854 | 1.33406 | 0.26015 | 0.611159 |
| PSPPH_2415 | lysine N6-hydroxylase/L-ornithine N5-oxygenase | chromosome:2791578-2792898 | 1448A-rhpS-MM | 1448A-WT-MM | NOTEST | 2.25675 | 13.8614 | 2.61875 | 0 | 1 | 1 |
| hrpV | type III negative regulator of hrp expression HrpV | chromosome:1494416-1494764 | 1448A-rhpS-MM | 1448A-WT-MM | OK | 267.992 | 1617.62 | 2.59361 | 3.62615 | 0.0006 | 0.0194043 |
| PSPPH_2854 | hypothetical protein | chromosome:3297452-3298421 | 1448A-rhpS-MM | 1448A-WT-MM | NOTEST | 3.43847 | 20.5929 | 2.58231 | 0 | 1 | 1 |
| PSPPH_2915 | glutamine amidotransferase, class-II protein | chromosome:3386173-3387774 | 1448A-rhpS-MM | 1448A-WT-MM | NOTEST | 3.61147 | 21.5735 | 2.57861 | 0 | 1 | 1 |
| hopX1 | type III effector HopX1 | chromosome:1508694-1509837 | 1448A-rhpS-MM | 1448A-WT-MM | OK | 331.544 | 1979.93 | 2.57817 | 7.15682 | 5.00E-05 | 0.00230303 |
| PSPPH_2132 | hypothetical protein | chromosome:2489069-2489291 | 1448A-rhpS-MM | 1448A-WT-MM | OK | 61.2149 | 363.634 | 2.57053 | 1.98234 | 0.2771 | 0.62329 |
| PSPPH_2168 | hypothetical protein | chromosome:2522849-2524415 | 1448A-rhpS-MM | 1448A-WT-MM | NOTEST | 3.1677 | 18.7014 | 2.56164 | 0 | 1 | 1 |
| PSPPH_4653 | hypothetical protein | chromosome:5292027-5293079 | 1448A-rhpS-MM | 1448A-WT-MM | OK | 49.2487 | 289.739 | 2.55659 | 1.06473 | 0.21475 | 0.566522 |
| hrcN | type III secretion component protein HrcN | chromosome:1499873-1502194 | 1448A-rhpS-MM | 1448A-WT-MM | OK | 157.84 | 927.293 | 2.55457 | 4.80681 | 5.00E-05 | 0.00230303 |
| PSPPH_3477 | hypothetical protein | chromosome:4018903-4019218 | 1448A-rhpS-MM | 1448A-WT-MM | OK | 18.283 | 107.229 | 2.55212 | 0.895973 | 0.26765 | 0.615939 |
| PSPPH_1931 | class V aminotransferase | chromosome:2274011-2276679 | 1448A-rhpS-MM | 1448A-WT-MM | NOTEST | 1.91801 | 10.9481 | 2.51299 | 0 | 1 | 1 |
| PSPPH_3083 | sulfur relay protein TusC | chromosome:3580488-3582480 | 1448A-rhpS-MM | 1448A-WT-MM | OK | 21.3459 | 121.255 | 2.50602 | 0.908452 | 0.19945 | 0.548217 |
| hopI1 | type III effector HopI1 | chromosome:4988660-4989671 | 1448A-rhpS-MM | 1448A-WT-MM | OK | 356.22 | 2004.23 | 2.49221 | 7.00381 | 5.00E-05 | 0.00230303 |
| PSPPH_3963 | TctC protein | chromosome:4525758-4526739 | 1448A-rhpS-MM | 1448A-WT-MM | NOTEST | 10.8153 | 60.7854 | 2.49065 | 0 | 1 | 1 |
| hrcJ | type III secretion component protein HrcJ | chromosome:1489436-1490770 | 1448A-rhpS-MM | 1448A-WT-MM | OK | 685.011 | 3843.71 | 2.4883 | 7.04842 | 5.00E-05 | 0.00230303 |
| PSPPH_2708 | hypothetical protein | chromosome:3135372-3135840 | 1448A-rhpS-MM | 1448A-WT-MM | NOTEST | 9.02302 | 50.385 | 2.48131 | 0 | 1 | 1 |
| PSPPH_4343 | (Fe-S)-binding protein | chromosome:4964180-4965697 | 1448A-rhpS-MM | 1448A-WT-MM | NOTEST | 11.8575 | 65.7523 | 2.47124 | 0 | 1 | 1 |
| hrpK1 | type III effector HrpK1 | chromosome:1506289-1508599 | 1448A-rhpS-MM | 1448A-WT-MM | OK | 594.988 | 3297.24 | 2.47033 | 8.49853 | 5.00E-05 | 0.00230303 |
| PSPPH_2289 | ISPsy19, transposase truncated | chromosome:2654780-2655588 | 1448A-rhpS-MM | 1448A-WT-MM | NOTEST | 7.2304 | 40.0229 | 2.46868 | 0 | 1 | 1 |
| hrcV | type III secretion component protein HrcV | chromosome:1502227-1505418 | 1448A-rhpS-MM | 1448A-WT-MM | OK | 118.246 | 650.53 | 2.45983 | 5.33938 | 5.00E-05 | 0.00230303 |
| PSPPH_1260 | short chain dehydrogenase/reductase oxidoreductase | chromosome:1466609-1467014 | 1448A-rhpS-MM | 1448A-WT-MM | NOTEST | 12.1946 | 65.9079 | 2.43421 | 0 | 1 | 1 |
| hrpD | type III secretion component protein HrpD | chromosome:1489436-1490770 | 1448A-rhpS-MM | 1448A-WT-MM | OK | 187.203 | 1006.04 | 2.42602 | 2.37862 | 0.00315 | 0.0583902 |
| PSPPH_4991 | glycosyl hydrolase | chromosome:5661141-5661621 | 1448A-rhpS-MM | 1448A-WT-MM | OK | 22.9796 | 122.906 | 2.41913 | 1.04192 | 0.07315 | 0.382824 |
| PSPPH_4694 | hypothetical protein | chromosome:5328334-5328586 | 1448A-rhpS-MM | 1448A-WT-MM | OK | 74.9708 | 392.676 | 2.38894 | 1.02562 | 0.07455 | 0.382824 |
| PSPPH_1191 | hypothetical protein | chromosome:1399398-1399638 | 1448A-rhpS-MM | 1448A-WT-MM | OK | 49.9522 | 259.128 | 2.37504 | 1.40004 | 0.2728 | 0.620278 |
| PSPPH_1914 | ABC transporter substrate-binding protein | chromosome:2231731-2232613 | 1448A-rhpS-MM | 1448A-WT-MM | NOTEST | 6.66705 | 34.3658 | 2.36585 | 0 | 1 | 1 |
| PSPPH_0407 | gluconate transporter family protein | chromosome:470481-471831 | 1448A-rhpS-MM | 1448A-WT-MM | NOTEST | 3.73965 | 19.1153 | 2.35375 | 0 | 1 | 1 |
| PSPPH_3297 | MarR family transcriptional regulator | chromosome:3821217-3826271 | 1448A-rhpS-MM | 1448A-WT-MM | NOTEST | 8.46366 | 43.2596 | 2.35367 | 0 | 1 | 1 |
| PSPPH_1757 | hypothetical protein | chromosome:2056405-2057344 | 1448A-rhpS-MM | 1448A-WT-MM | NOTEST | 5.69689 | 28.8711 | 2.34138 | 0 | 1 | 1 |
| PSPPH_3421 | hypothetical protein | chromosome:3952665-3954509 | 1448A-rhpS-MM | 1448A-WT-MM | NOTEST | 7.81721 | 39.2264 | 2.3271 | 0 | 1 | 1 |
| PSPPH_1393 | repressor protein c2 | chromosome:1618086-1619013 | 1448A-rhpS-MM | 1448A-WT-MM | NOTEST | 13.154 | 65.4463 | 2.31481 | 0 | 1 | 1 |
| flgB | flagellar basal-body rod protein FlgB | chromosome:3942313-3942721 | 1448A-rhpS-MM | 1448A-WT-MM | NOTEST | 10.2507 | 50.9396 | 2.31307 | 0 | 1 | 1 |
| PSPPH_0871 | hypothetical protein | chromosome:1041433-1041850 | 1448A-rhpS-MM | 1448A-WT-MM | OK | 64.5836 | 318.554 | 2.3023 | 1.87893 | 0.04745 | 0.308222 |
| PSPPH_3885 | hypothetical protein | chromosome:4443665-4444130 | 1448A-rhpS-MM | 1448A-WT-MM | NOTEST | 9.3456 | 46.0488 | 2.3008 | 0 | 1 | 1 |
| PSPPH_1096 | hypothetical protein | chromosome:1290703-1291900 | 1448A-rhpS-MM | 1448A-WT-MM | NOTEST | 16.2209 | 79.7146 | 2.29699 | 0 | 1 | 1 |
| PSPPH_3128 | peptidase propeptide/YPEB domain-containing protein | chromosome:3628139-3630740 | 1448A-rhpS-MM | 1448A-WT-MM | OK | 28.6599 | 140.726 | 2.29578 | 1.09443 | 0.2976 | 0.6413 |
| hrcC | type III outer membrane protein HrcC | chromosome:1492114-1494414 | 1448A-rhpS-MM | 1448A-WT-MM | OK | 325.951 | 1593.99 | 2.28992 | 7.2717 | 5.00E-05 | 0.00230303 |
| PSPPH_3741 | phage integrase | chromosome:4290445-4291432 | 1448A-rhpS-MM | 1448A-WT-MM | NOTEST | 6.47407 | 31.0226 | 2.26058 | 0 | 1 | 1 |
| PSPPH_2852 | TonB domain-containing protein | chromosome:3296108-3296936 | 1448A-rhpS-MM | 1448A-WT-MM | NOTEST | 12.3631 | 59.0492 | 2.25588 | 0 | 1 | 1 |
| fumC | fumarate hydratase | chromosome:4742393-4744170 | 1448A-rhpS-MM | 1448A-WT-MM | NOTEST | 5.80686 | 27.5705 | 2.24729 | 0 | 1 | 1 |
| hopD1 | type III effector HopD1 | large_plasmid:7146-9279 | 1448A-rhpS-MM | 1448A-WT-MM | OK | 677.255 | 3198.87 | 2.23979 | 7.70893 | 5.00E-05 | 0.00230303 |
| ribH | riboflavin synthase subunit beta | chromosome:4052584-4053097 | 1448A-rhpS-MM | 1448A-WT-MM | OK | 108.324 | 511.28 | 2.23876 | 2.67501 | 0.0045 | 0.0747541 |
| PSPPH_4625 | ABC transporter permease | chromosome:5261858-5263885 | 1448A-rhpS-MM | 1448A-WT-MM | NOTEST | 4.72264 | 22.263 | 2.23698 | 0 | 1 | 1 |
| PSPPH_0660 | prophage PSPPH01 tail protein | chromosome:775907-776545 | 1448A-rhpS-MM | 1448A-WT-MM | OK | 42.4538 | 199.917 | 2.23544 | 1.14287 | 0.2434 | 0.594804 |
| PSPPH_2689 | LysR family transcriptional regulator | chromosome:3113454-3114360 | 1448A-rhpS-MM | 1448A-WT-MM | NOTEST | 9.70738 | 45.6193 | 2.23249 | 0 | 1 | 1 |
| fliN | flagellar motor switch protein | chromosome:3900029-3900488 | 1448A-rhpS-MM | 1448A-WT-MM | NOTEST | 9.13223 | 42.8618 | 2.23065 | 0 | 1 | 1 |
| hopAF1 | type III effector HopAF1 | chromosome:1685201-1686056 | 1448A-rhpS-MM | 1448A-WT-MM | OK | 57.0263 | 265.011 | 2.21635 | 2.65139 | 0.00555 | 0.0865231 |
| hrpP | type III secretion protein HrpP | chromosome:1497756-1499418 | 1448A-rhpS-MM | 1448A-WT-MM | OK | 170.502 | 790.079 | 2.21221 | 3.15595 | 0.0005 | 0.0168889 |
| ccmA | cytochrome c biogenesis protein CcmA | chromosome:3835325-3836635 | 1448A-rhpS-MM | 1448A-WT-MM | NOTEST | 10.8952 | 50.3455 | 2.20817 | 0 | 1 | 1 |
| PSPPH_4764 | transmembrane sensor | chromosome:5408344-5409843 | 1448A-rhpS-MM | 1448A-WT-MM | NOTEST | 8.71271 | 40.1205 | 2.20314 | 0 | 1 | 1 |
| PSPPH_2073 | nitrate transporter | chromosome:2431031-2432243 | 1448A-rhpS-MM | 1448A-WT-MM | NOTEST | 2.67472 | 12.3024 | 2.20148 | 0 | 1 | 1 |
| PSPPH_0294 | hypothetical protein | chromosome:336964-338262 | 1448A-rhpS-MM | 1448A-WT-MM | NOTEST | 5.09701 | 23.2717 | 2.19085 | 0 | 1 | 1 |
| PSPPH_1237 | isochorismatase | chromosome:1443871-1444936 | 1448A-rhpS-MM | 1448A-WT-MM | NOTEST | 4.9092 | 22.388 | 2.18916 | 0 | 1 | 1 |
| avrE1 | type III effector AvrE1 | chromosome:1477726-1482871 | 1448A-rhpS-MM | 1448A-WT-MM | OK | 25.3322 | 115.257 | 2.18581 | 4.59439 | 5.00E-05 | 0.00230303 |
| avrB2 | type III effector AvrB2 | large_plasmid:102432-103491 | 1448A-rhpS-MM | 1448A-WT-MM | OK | 220.617 | 993.522 | 2.17101 | 5.01002 | 5.00E-05 | 0.00230303 |
| soxA | sarcosine oxidase subunit alpha | chromosome:5395145-5398459 | 1448A-rhpS-MM | 1448A-WT-MM | OK | 34.3689 | 153.707 | 2.16101 | 4.09896 | 5.00E-05 | 0.00230303 |
| hrcQa | type III secretion component protein HrcQa | chromosome:1497756-1499418 | 1448A-rhpS-MM | 1448A-WT-MM | OK | 134.937 | 603.243 | 2.16045 | 3.17169 | 0.00055 | 0.0179785 |
| PSPPH_1171 | hypothetical protein | chromosome:1375356-1376426 | 1448A-rhpS-MM | 1448A-WT-MM | OK | 40.5112 | 178.032 | 2.13574 | 1.28446 | 0.1005 | 0.438772 |
| PSPPH_0608 | pancortin-3 | chromosome:718228-719993 | 1448A-rhpS-MM | 1448A-WT-MM | OK | 21.5664 | 94.3392 | 2.12907 | 0.983689 | 0.29485 | 0.639332 |
| hopF3 | type III effector HopF3 | chromosome:4039662-4040259 | 1448A-rhpS-MM | 1448A-WT-MM | OK | 741.235 | 3234.01 | 2.12532 | 5.70403 | 5.00E-05 | 0.00230303 |
| hrpR | type III transcriptional regulator HrpR | chromosome:1485637-1486558 | 1448A-rhpS-MM | 1448A-WT-MM | OK | 56.7764 | 247.567 | 2.12445 | 2.68633 | 0.00405 | 0.0695593 |
| PSPPH_2963 | sarcosine oxidase subunit delta | chromosome:3438546-3441755 | 1448A-rhpS-MM | 1448A-WT-MM | OK | 18.0183 | 78.5519 | 2.12418 | 0.678098 | 0.3667 | 0.694073 |
| PSPPH_4996 | hypothetical protein | chromosome:5665899-5666697 | 1448A-rhpS-MM | 1448A-WT-MM | NOTEST | 16.0838 | 70.076 | 2.12331 | 0 | 1 | 1 |
| PSPPH_5033 | peptide ABC transporter ATP-binding protein | chromosome:5702959-5708802 | 1448A-rhpS-MM | 1448A-WT-MM | NOTEST | 5.29969 | 23.0879 | 2.12316 | 0 | 1 | 1 |
| PSPPH_2981 | sulfate permease | chromosome:3461625-3462954 | 1448A-rhpS-MM | 1448A-WT-MM | NOTEST | 6.2463 | 26.7799 | 2.10007 | 0 | 1 | 1 |
| hrpO | type III secretion component protein HrpO | chromosome:1499424-1499871 | 1448A-rhpS-MM | 1448A-WT-MM | OK | 140.771 | 596.405 | 2.08294 | 2.53613 | 0.0069 | 0.101333 |
| PSPPH_3758 | riboflavin biosynthesis protein RibD domain-containing protein | chromosome:4310220-4310874 | 1448A-rhpS-MM | 1448A-WT-MM | OK | 159.8 | 672.749 | 2.0738 | 3.48608 | 0.0003 | 0.0108571 |
| PSPPH_3759 | zinc-binding oxidoreductase | chromosome:4310951-4311974 | 1448A-rhpS-MM | 1448A-WT-MM | OK | 589.326 | 2480.78 | 2.07366 | 6.19568 | 5.00E-05 | 0.00230303 |
| PSPPH_1061 | group 1 glycosyl transferase | chromosome:1252015-1254308 | 1448A-rhpS-MM | 1448A-WT-MM | NOTEST | 7.24699 | 30.4918 | 2.07297 | 0 | 1 | 1 |
| PSPPH_2723 | glycerone kinase | chromosome:3148868-3149513 | 1448A-rhpS-MM | 1448A-WT-MM | NOTEST | 14.6937 | 61.6138 | 2.06806 | 0 | 1 | 1 |
| hopAT1 | type III effector HopAT1 | chromosome:894852-895116 | 1448A-rhpS-MM | 1448A-WT-MM | OK | 296.145 | 1239.19 | 2.06502 | 2.2849 | 0.0173 | 0.187035 |
| PSPPH_2808 | hypothetical protein | chromosome:3253183-3254969 | 1448A-rhpS-MM | 1448A-WT-MM | OK | 29.1357 | 121.776 | 2.06337 | 0.871068 | 0.185 | 0.532576 |
| PSPPH_2746 | dipeptide ABC transporter ATP binding protein | chromosome:3175454-3178867 | 1448A-rhpS-MM | 1448A-WT-MM | NOTEST | 10.1007 | 42.1447 | 2.0609 | 0 | 1 | 1 |
| PSPPH_1492 | major facilitator family transporter | chromosome:1733595-1734369 | 1448A-rhpS-MM | 1448A-WT-MM | NOTEST | 4.56351 | 19.0277 | 2.05988 | 0 | 1 | 1 |
| PSPPH_0232 | iron ABC transporter substrate-binding protein | chromosome:268097-269099 | 1448A-rhpS-MM | 1448A-WT-MM | NOTEST | 6.18563 | 25.6828 | 2.05381 | 0 | 1 | 1 |
| cysW | sulfate ABC transporter permease CysW | chromosome:95149-96022 | 1448A-rhpS-MM | 1448A-WT-MM | NOTEST | 16.5828 | 67.7565 | 2.03067 | 0 | 1 | 1 |
| PSPPH_1051 | ABC transporter ATP-binding protein | chromosome:1241701-1246332 | 1448A-rhpS-MM | 1448A-WT-MM | NOTEST | 8.53246 | 34.6781 | 2.02299 | 0 | 1 | 1 |
| hopAK1 | type III helper protein HopAK1 | chromosome:1652069-1653644 | 1448A-rhpS-MM | 1448A-WT-MM | OK | 344.017 | 1394.06 | 2.01874 | 6.24072 | 5.00E-05 | 0.00230303 |
| PSPPH_4429 | hypothetical protein | chromosome:5061608-5061959 | 1448A-rhpS-MM | 1448A-WT-MM | NOTEST | 16.3 | 66.0306 | 2.01826 | 0 | 1 | 1 |
| PSPPH_1617 | MarR family transcriptional regulator | chromosome:1876765-1877218 | 1448A-rhpS-MM | 1448A-WT-MM | OK | 71.9662 | 290.936 | 2.01531 | 1.8596 | 0.03415 | 0.262929 |
| PSPPH_2695 | peptide ABC transporter permease | chromosome:3121352-3124701 | 1448A-rhpS-MM | 1448A-WT-MM | NOTEST | 3.75156 | 15.0979 | 2.00878 | 0 | 1 | 1 |
| PSPPH_4673 | Cro/CI family transcriptional regulator | chromosome:5310338-5311070 | 1448A-rhpS-MM | 1448A-WT-MM | NOTEST | 4.13334 | 16.5241 | 1.99919 | 0 | 1 | 1 |
| PSPPH_2534 | type III secretion component | chromosome:2918208-2919872 | 1448A-rhpS-MM | 1448A-WT-MM | NOTEST | 7.06167 | 28.2296 | 1.99913 | 0 | 1 | 1 |
| hopAV1 | type III effector HopAV1 | large_plasmid:45494-48066 | 1448A-rhpS-MM | 1448A-WT-MM | OK | 542.374 | 2148.62 | 1.98605 | 6.76974 | 5.00E-05 | 0.00230303 |
| PSPPH_2582 | TerC family membrane protein | chromosome:2974922-2975681 | 1448A-rhpS-MM | 1448A-WT-MM | OK | 253.496 | 1004.16 | 1.98596 | 4.16899 | 5.00E-05 | 0.00230303 |
| PSPPH_0762 | hypothetical protein | chromosome:892073-892562 | 1448A-rhpS-MM | 1448A-WT-MM | OK | 138.432 | 544.685 | 1.97625 | 2.42287 | 0.00665 | 0.100079 |
| hopAB1 | type III effector HopAB1 | large_plasmid:106990-108726 | 1448A-rhpS-MM | 1448A-WT-MM | OK | 1048.02 | 4094.1 | 1.96588 | 6.7982 | 5.00E-05 | 0.00230303 |
| PSPPH_0295 | hypothetical protein | chromosome:338359-339097 | 1448A-rhpS-MM | 1448A-WT-MM | NOTEST | 4.25989 | 16.3544 | 1.94079 | 0 | 1 | 1 |
| PSPPH_1182 | glucose ABC transporter permease | chromosome:1388742-1390489 | 1448A-rhpS-MM | 1448A-WT-MM | OK | 82.8682 | 317.958 | 1.93995 | 2.54823 | 0.00535 | 0.0851518 |
| ccmC | heme exporter protein CcmC | chromosome:3833830-3835256 | 1448A-rhpS-MM | 1448A-WT-MM | NOTEST | 15.3161 | 58.5039 | 1.93349 | 0 | 1 | 1 |
| PSPPH_1009 | hypothetical protein | chromosome:1195847-1202294 | 1448A-rhpS-MM | 1448A-WT-MM | NOTEST | 11.4744 | 43.7216 | 1.92993 | 0 | 1 | 1 |
| PSPPH_4805 | oxidoreductase | chromosome:5452395-5453496 | 1448A-rhpS-MM | 1448A-WT-MM | OK | 25.6825 | 97.4334 | 1.92363 | 1.75919 | 0.03505 | 0.26638 |
| PSPPH_4992 | hypothetical protein | chromosome:5661918-5662275 | 1448A-rhpS-MM | 1448A-WT-MM | OK | 21.9714 | 83.0744 | 1.91878 | 0.896999 | 0.33275 | 0.671241 |
| PSPPH_1692 | hypothetical protein | chromosome:1953399-1955032 | 1448A-rhpS-MM | 1448A-WT-MM | OK | 31.1241 | 117.607 | 1.91787 | 1.30275 | 0.1062 | 0.4484 |
| PSPPH_2076 | nitrate transporter component | chromosome:2435032-2436331 | 1448A-rhpS-MM | 1448A-WT-MM | NOTEST | 14.1322 | 52.9299 | 1.9051 | 0 | 1 | 1 |
| hrpJ | type III secretion component protein HrcJ | chromosome:1502227-1505418 | 1448A-rhpS-MM | 1448A-WT-MM | OK | 128.658 | 481.786 | 1.90485 | 2.85753 | 0.0009 | 0.0244286 |
| PSPPH_2256 | hypothetical protein | chromosome:2619714-2619897 | 1448A-rhpS-MM | 1448A-WT-MM | OK | 152.492 | 569.762 | 1.90163 | 1.50936 | 0.2733 | 0.620941 |
| PSPPH_2282 | Rhs family protein | chromosome:2647752-2649491 | 1448A-rhpS-MM | 1448A-WT-MM | NOTEST | 6.53083 | 24.2173 | 1.8907 | 0 | 1 | 1 |
| PSPPH_2380 | hypothetical protein | chromosome:2758098-2762654 | 1448A-rhpS-MM | 1448A-WT-MM | NOTEST | 9.48293 | 35.131 | 1.88934 | 0 | 1 | 1 |
| hopAW1 | type III effector HopAW1 | large_plasmid:104813-105470 | 1448A-rhpS-MM | 1448A-WT-MM | OK | 474.559 | 1750.14 | 1.88281 | 4.58682 | 5.00E-05 | 0.00230303 |
| PSPPH_2460 | hypothetical protein | chromosome:2841956-2842727 | 1448A-rhpS-MM | 1448A-WT-MM | NOTEST | 8.63482 | 31.8145 | 1.88145 | 0 | 1 | 1 |
| pcaG | protocatechuate 3,4-dioxygenase subunit alpha | chromosome:2455735-2456338 | 1448A-rhpS-MM | 1448A-WT-MM | OK | 27.5014 | 101.194 | 1.87955 | 1.33235 | 0.11895 | 0.459086 |
| hopAJ1 | type III helper protein HopAJ1 | chromosome:892800-894042 | 1448A-rhpS-MM | 1448A-WT-MM | OK | 517.71 | 1891.83 | 1.86956 | 5.74218 | 5.00E-05 | 0.00230303 |
| PSPPH_4242 | sensor histidine kinase/response regulator | chromosome:4839175-4840744 | 1448A-rhpS-MM | 1448A-WT-MM | NOTEST | 10.2234 | 37.244 | 1.86513 | 0 | 1 | 1 |
| PSPPH_1769 | polysaccharide deacetylase | chromosome:2066158-2067600 | 1448A-rhpS-MM | 1448A-WT-MM | NOTEST | 14.6796 | 53.4264 | 1.86374 | 0 | 1 | 1 |
| hrpG | type III secretion component protein HrpG | chromosome:1491470-1492113 | 1448A-rhpS-MM | 1448A-WT-MM | OK | 748.16 | 2717.56 | 1.86089 | 3.91749 | 5.00E-05 | 0.00230303 |
| hrcU | type III secretion component protein HrcU | chromosome:1494961-1496832 | 1448A-rhpS-MM | 1448A-WT-MM | OK | 24.1843 | 87.4704 | 1.85472 | 1.59448 | 0.09315 | 0.420646 |
| PSPPH_0537 | cytosine/purines uracil thiamine allantoin permease | chromosome:632078-633129 | 1448A-rhpS-MM | 1448A-WT-MM | OK | 33.5471 | 121.237 | 1.85357 | 1.00539 | 0.332 | 0.671165 |
| phnG | phosphonate metabolism protein PhnG | chromosome:3404965-3407989 | 1448A-rhpS-MM | 1448A-WT-MM | OK | 45.186 | 163.248 | 1.85312 | 1.06326 | 0.1537 | 0.502417 |
| hrpE | type III secretion component protein HrpE | chromosome:1490805-1491387 | 1448A-rhpS-MM | 1448A-WT-MM | OK | 281.506 | 1011.57 | 1.84535 | 3.63346 | 5.00E-05 | 0.00230303 |
| PSPPH_2049 | phosphoadenosine phosphosulfate reductase | chromosome:2401732-2402467 | 1448A-rhpS-MM | 1448A-WT-MM | OK | 62.0306 | 222.9 | 1.84535 | 2.07214 | 0.0155 | 0.174435 |
| PSPPH_1525 | hypothetical protein | chromosome:1774850-1782566 | 1448A-rhpS-MM | 1448A-WT-MM | OK | 50.3828 | 180.203 | 1.83862 | 5.36893 | 5.00E-05 | 0.00230303 |
| PSPPH_1949 | heat shock protein 20 | chromosome:2294868-2295312 | 1448A-rhpS-MM | 1448A-WT-MM | OK | 2161.7 | 7684.5 | 1.82978 | 5.71627 | 5.00E-05 | 0.00230303 |
| PSPPH_3286 | DNA-3-methyladenine glycosylase 1 | chromosome:3808887-3809502 | 1448A-rhpS-MM | 1448A-WT-MM | NOTEST | 9.69826 | 34.4626 | 1.82923 | 0 | 1 | 1 |
| PSPPH_1744 | carbohydrate transporter | chromosome:2022845-2024045 | 1448A-rhpS-MM | 1448A-WT-MM | NOTEST | 5.59578 | 19.5477 | 1.80459 | 0 | 1 | 1 |
| PSPPH_0238 | hypothetical protein | chromosome:275078-275426 | 1448A-rhpS-MM | 1448A-WT-MM | OK | 22.9808 | 80.1112 | 1.80158 | 0.830368 | 0.35375 | 0.687056 |
| PSPPH_1762 | AsnC family transcriptional regulator | chromosome:2060167-2060686 | 1448A-rhpS-MM | 1448A-WT-MM | NOTEST | 16.5158 | 57.5098 | 1.79997 | 0 | 1 | 1 |
| PSPPH_4813 | dipeptidase | chromosome:5465373-5466351 | 1448A-rhpS-MM | 1448A-WT-MM | OK | 42.8798 | 148.932 | 1.79628 | 2.08447 | 0.01955 | 0.197149 |
| relB | bifunctional antitoxin/transcriptional repressor RelB | chromosome:186130-186650 | 1448A-rhpS-MM | 1448A-WT-MM | OK | 49.9522 | 171.812 | 1.78221 | 1.13027 | 0.44785 | 0.75219 |
| PSPPH_1342 | hypothetical protein | chromosome:1561022-1561334 | 1448A-rhpS-MM | 1448A-WT-MM | OK | 79.701 | 272.809 | 1.77522 | 1.25611 | 0.1311 | 0.477091 |
| PSPPH_3233 | LysR family transcriptional regulator | chromosome:3746400-3748703 | 1448A-rhpS-MM | 1448A-WT-MM | NOTEST | 11.1802 | 38.256 | 1.77474 | 0 | 1 | 1 |
| PSPPH_2661 | amino acid ABC transporter permease | chromosome:3083528-3084191 | 1448A-rhpS-MM | 1448A-WT-MM | OK | 23.5547 | 80.3922 | 1.77104 | 1.13398 | 0.1425 | 0.492833 |
| PSPPH_4728 | ABC transporter permease | chromosome:5363953-5368805 | 1448A-rhpS-MM | 1448A-WT-MM | NOTEST | 10.4478 | 35.5046 | 1.7648 | 0 | 1 | 1 |
| ppiC1 | peptidyl-prolyl cis-trans isomerase C | chromosome:2693133-2693409 | 1448A-rhpS-MM | 1448A-WT-MM | OK | 59.7405 | 201.828 | 1.75634 | 0.741062 | 0.1234 | 0.467721 |
| PSPPH_1907 | sensor histidine kinase | chromosome:2209435-2213944 | 1448A-rhpS-MM | 1448A-WT-MM | NOTEST | 20.1744 | 68.0858 | 1.75483 | 0 | 1 | 1 |
| PSPPH_2200 | hypothetical protein | chromosome:2556233-2556575 | 1448A-rhpS-MM | 1448A-WT-MM | OK | 84.4062 | 282.989 | 1.74533 | 1.26075 | 0.1011 | 0.439063 |
| PSPPH_1134 | cyanate MFS transporter | chromosome:1333862-1335092 | 1448A-rhpS-MM | 1448A-WT-MM | NOTEST | 5.21881 | 17.3876 | 1.73627 | 0 | 1 | 1 |
| grxC | glutaredoxin 3 | chromosome:5586844-5587096 | 1448A-rhpS-MM | 1448A-WT-MM | OK | 93.837 | 311.686 | 1.73186 | 1.32174 | 0.2174 | 0.567979 |
| PSPPH_4499 | urease accessory protein | chromosome:5134625-5135198 | 1448A-rhpS-MM | 1448A-WT-MM | NOTEST | 6.89679 | 22.7938 | 1.72465 | 0 | 1 | 1 |
| PSPPH_4228 | acetyltransferase | chromosome:4822543-4822978 | 1448A-rhpS-MM | 1448A-WT-MM | OK | 25.4758 | 83.8191 | 1.71815 | 0.728999 | 0.1275 | 0.474024 |
| PSPPH_2305 | TetR family transcriptional regulator | chromosome:2672503-2673130 | 1448A-rhpS-MM | 1448A-WT-MM | OK | 30.8114 | 101.325 | 1.71746 | 1.2049 | 0.10565 | 0.44732 |
| PSPPH_2775 | monooxygenase flavin-binding family protein | chromosome:3212819-3215035 | 1448A-rhpS-MM | 1448A-WT-MM | NOTEST | 17.7658 | 58.4071 | 1.71704 | 0 | 1 | 1 |
| PSPPH_3068 | phosphinothricin N-acetyltransferase | chromosome:3563919-3564459 | 1448A-rhpS-MM | 1448A-WT-MM | NOTEST | 18.9146 | 61.8657 | 1.70964 | 0 | 1 | 1 |
| hrcT | type III secretion component protein HrcT | chromosome:1494961-1496832 | 1448A-rhpS-MM | 1448A-WT-MM | NOTEST | 22.6192 | 73.4758 | 1.69972 | 0 | 1 | 1 |
| PSPPH_4300 | hypothetical protein | chromosome:4911411-4912629 | 1448A-rhpS-MM | 1448A-WT-MM | NOTEST | 15.8218 | 51.3327 | 1.69796 | 0 | 1 | 1 |
| PSPPH_0029 | glycine betaine family ABC transporter substrate-binding protein | chromosome:34759-35686 | 1448A-rhpS-MM | 1448A-WT-MM | NOTEST | 16.7441 | 53.9776 | 1.6887 | 0 | 1 | 1 |
| PSPPH_2278 | ABC transporter binding protein-like protein AabH | chromosome:2643893-2644766 | 1448A-rhpS-MM | 1448A-WT-MM | NOTEST | 13.0459 | 41.8172 | 1.6805 | 0 | 1 | 1 |
| PSPPH_2913 | glutamate synthase | chromosome:3384823-3386158 | 1448A-rhpS-MM | 1448A-WT-MM | NOTEST | 13.4635 | 43.0376 | 1.67654 | 0 | 1 | 1 |
| PSPPH_1768 | hypothetical protein | chromosome:2066158-2067600 | 1448A-rhpS-MM | 1448A-WT-MM | NOTEST | 19.391 | 61.8718 | 1.6739 | 0 | 1 | 1 |
| flaG | flagellin FlaG | chromosome:3919394-3919793 | 1448A-rhpS-MM | 1448A-WT-MM | OK | 28.7685 | 91.7688 | 1.67351 | 0.709588 | 0.1317 | 0.477688 |
| PSPPH_0154 | hypothetical protein | chromosome:182434-182668 | 1448A-rhpS-MM | 1448A-WT-MM | OK | 218.217 | 695.36 | 1.67199 | 1.31129 | 0.09235 | 0.419647 |
| PSPPH_0500 | hypothetical protein | chromosome:580782-581307 | 1448A-rhpS-MM | 1448A-WT-MM | OK | 43.3272 | 137.952 | 1.67083 | 1.24345 | 0.1024 | 0.440929 |
| PSPPH_1139 | hypothetical protein | chromosome:1337542-1338175 | 1448A-rhpS-MM | 1448A-WT-MM | OK | 34.2564 | 108.813 | 1.66741 | 1.20404 | 0.1024 | 0.440929 |
| PSPPH_2532 | hypothetical protein | chromosome:2917681-2918167 | 1448A-rhpS-MM | 1448A-WT-MM | NOTEST | 9.07178 | 28.7608 | 1.66465 | 0 | 1 | 1 |
| PSPPH_4763 | hypothetical protein | chromosome:5406910-5408223 | 1448A-rhpS-MM | 1448A-WT-MM | NOTEST | 7.22917 | 22.8649 | 1.66123 | 0 | 1 | 1 |
| PSPPH_0915 | MotA/TolQ/ExbB proton channel family protein | chromosome:1089700-1090351 | 1448A-rhpS-MM | 1448A-WT-MM | NOTEST | 5.234 | 16.3732 | 1.64535 | 0 | 1 | 1 |
| PSPPH_2809 | UDP-glucose 6-dehydrogenase | chromosome:3253183-3254969 | 1448A-rhpS-MM | 1448A-WT-MM | NOTEST | 7.91445 | 24.6695 | 1.64017 | 0 | 1 | 1 |
| PSPPH_0271 | Sco1/SenC family protein | chromosome:313249-313909 | 1448A-rhpS-MM | 1448A-WT-MM | OK | 25.4616 | 79.324 | 1.63944 | 1.13484 | 0.1345 | 0.481808 |
| PSPPH_3557 | hypothetical protein | chromosome:4092785-4093256 | 1448A-rhpS-MM | 1448A-WT-MM | NOTEST | 18.447 | 57.417 | 1.63809 | 0 | 1 | 1 |
| PSPPH_2452 | multidrug transporter | chromosome:2833979-2835539 | 1448A-rhpS-MM | 1448A-WT-MM | NOTEST | 5.27274 | 16.3998 | 1.63705 | 0 | 1 | 1 |
| PSPPH_3007 | iron ABC transporter permease | chromosome:3489422-3492411 | 1448A-rhpS-MM | 1448A-WT-MM | NOTEST | 9.08716 | 28.2129 | 1.63445 | 0 | 1 | 1 |
| hutF | N-formimino-L-glutamate deiminase | chromosome:397567-398932 | 1448A-rhpS-MM | 1448A-WT-MM | OK | 47.7101 | 147.906 | 1.63231 | 2.28284 | 0.0074 | 0.107636 |
| PSPPH_2349 | amine oxidase | chromosome:2723807-2725391 | 1448A-rhpS-MM | 1448A-WT-MM | OK | 31.8019 | 98.3125 | 1.62826 | 2.10073 | 0.0116 | 0.146933 |
| PSPPH_4961 | prophage PSPPH06 lysozyme | chromosome:5632949-5633953 | 1448A-rhpS-MM | 1448A-WT-MM | NOTEST | 7.13917 | 22.0279 | 1.62551 | 0 | 1 | 1 |
| PSPPH_A0133 | hypothetical protein | large_plasmid:114024-120480 | 1448A-rhpS-MM | 1448A-WT-MM | OK | 185.917 | 572.704 | 1.62313 | 5.6151 | 5.00E-05 | 0.00230303 |
| trpI | transcriptional regulator TrpI | chromosome:42707-43616 | 1448A-rhpS-MM | 1448A-WT-MM | NOTEST | 15.4099 | 47.4456 | 1.62242 | 0 | 1 | 1 |
| hrcR | type III secretion system protein | chromosome:1497106-1497754 | 1448A-rhpS-MM | 1448A-WT-MM | OK | 105.723 | 325.428 | 1.62206 | 2.19916 | 0.0086 | 0.121037 |
| PSPPH_0427 | acyl carrier protein | chromosome:490912-491715 | 1448A-rhpS-MM | 1448A-WT-MM | OK | 40.8897 | 125.779 | 1.62108 | 0.631605 | 0.36945 | 0.696298 |
| phnE | phosphonate ABC transporter permease | chromosome:3408774-3409563 | 1448A-rhpS-MM | 1448A-WT-MM | NOTEST | 17.1172 | 52.4442 | 1.61534 | 0 | 1 | 1 |
| PSPPH_2148 | hypothetical protein | chromosome:2501090-2502199 | 1448A-rhpS-MM | 1448A-WT-MM | NOTEST | 7.33186 | 22.4388 | 1.61374 | 0 | 1 | 1 |
| PSPPH_3815 | esterase | chromosome:4366393-4367239 | 1448A-rhpS-MM | 1448A-WT-MM | OK | 96.1262 | 292.166 | 1.60379 | 2.41062 | 0.00455 | 0.0751739 |
| tauB | taurine ABC transporter ATP-binding subunit | chromosome:5583347-5585005 | 1448A-rhpS-MM | 1448A-WT-MM | NOTEST | 7.07541 | 21.3829 | 1.59557 | 0 | 1 | 1 |
| PSPPH_3376 | Hpt domain-containing protein | chromosome:3904066-3904423 | 1448A-rhpS-MM | 1448A-WT-MM | OK | 113.801 | 343.613 | 1.59427 | 1.41616 | 0.06845 | 0.373587 |
| PSPPH_2510 | sensor histidine kinase | chromosome:2897105-2897765 | 1448A-rhpS-MM | 1448A-WT-MM | OK | 28.1881 | 85.0149 | 1.59263 | 1.05804 | 0.11815 | 0.458718 |
| PSPPH_1233 | GntR family transcriptional regulator | chromosome:1440876-1442632 | 1448A-rhpS-MM | 1448A-WT-MM | NOTEST | 22.5598 | 67.8837 | 1.58931 | 0 | 1 | 1 |
| PSPPH_3705 | NLP/P60 family protein | chromosome:4252057-4252786 | 1448A-rhpS-MM | 1448A-WT-MM | OK | 154.687 | 464.4 | 1.58602 | 2.61017 | 0.00165 | 0.0388837 |
| PSPPH_1269 | lytic murein transglycosylase | chromosome:1483092-1484556 | 1448A-rhpS-MM | 1448A-WT-MM | OK | 74.1884 | 222.519 | 1.58466 | 2.80332 | 0.00065 | 0.01976 |
| PSPPH_2107 | shikimate 5-dehydrogenase | chromosome:2467531-2468386 | 1448A-rhpS-MM | 1448A-WT-MM | NOTEST | 24.2904 | 72.7538 | 1.58264 | 0 | 1 | 1 |
| thiS | sulfur carrier protein ThiS | chromosome:5420719-5420992 | 1448A-rhpS-MM | 1448A-WT-MM | OK | 60.6033 | 181.261 | 1.5806 | 0.653992 | 0.16385 | 0.513666 |
| PSPPH_A0094 | prevent-host-death family protein | large_plasmid:82423-82672 | 1448A-rhpS-MM | 1448A-WT-MM | OK | 270.326 | 808.302 | 1.5802 | 1.42859 | 0.0742 | 0.382824 |
| PSPPH_0284 | amino acid ABC transporter permease | chromosome:326113-328177 | 1448A-rhpS-MM | 1448A-WT-MM | NOTEST | 8.73277 | 25.9732 | 1.57251 | 0 | 1 | 1 |
| glpF | glycerol uptake facilitator protein | chromosome:4456058-4456961 | 1448A-rhpS-MM | 1448A-WT-MM | OK | 26.3944 | 78.4786 | 1.57207 | 1.31163 | 0.12195 | 0.466693 |
| hrcQb | type III secretion component protein HrcQb | chromosome:1497756-1499418 | 1448A-rhpS-MM | 1448A-WT-MM | OK | 242.184 | 719.683 | 1.57126 | 1.87881 | 0.01805 | 0.190528 |
| PSPPH_2387 | S-layer protein | chromosome:2768201-2768543 | 1448A-rhpS-MM | 1448A-WT-MM | NOTEST | 15.6594 | 46.4696 | 1.56926 | 0 | 1 | 1 |
| PSPPH_1101 | NodT family outer membrane efflux lipoprotein | chromosome:1295168-1299907 | 1448A-rhpS-MM | 1448A-WT-MM | NOTEST | 18.0322 | 53.4142 | 1.56665 | 0 | 1 | 1 |
| PSPPH_4951 | hypothetical protein | chromosome:5623648-5625536 | 1448A-rhpS-MM | 1448A-WT-MM | NOTEST | 8.92915 | 26.4179 | 1.56492 | 0 | 1 | 1 |
| tonB2 | ferric siderophore ABC transporter substrate-binding protein | chromosome:1086610-1087372 | 1448A-rhpS-MM | 1448A-WT-MM | NOTEST | 7.37864 | 21.819 | 1.56416 | 0 | 1 | 1 |
| PSPPH_0698 | PAAR motif-containing protein | chromosome:820295-821465 | 1448A-rhpS-MM | 1448A-WT-MM | NOTEST | 7.41325 | 21.7705 | 1.55419 | 0 | 1 | 1 |
| PSPPH_1035 | lipoprotein | chromosome:1223312-1224131 | 1448A-rhpS-MM | 1448A-WT-MM | OK | 85.596 | 248.973 | 1.54038 | 2.13497 | 0.00975 | 0.132321 |
| PSPPH_2905 | transcriptional regulator NfxB | chromosome:3372797-3373370 | 1448A-rhpS-MM | 1448A-WT-MM | NOTEST | 14.034 | 40.7388 | 1.53747 | 0 | 1 | 1 |
| PSPPH_4954 | hypothetical protein | chromosome:5628151-5632456 | 1448A-rhpS-MM | 1448A-WT-MM | NOTEST | 7.87098 | 22.6673 | 1.52599 | 0 | 1 | 1 |
| PSPPH_2342 | hypothetical protein | chromosome:2716118-2716955 | 1448A-rhpS-MM | 1448A-WT-MM | OK | 28.9726 | 83.4058 | 1.52546 | 1.2069 | 0.111 | 0.45294 |
| PSPPH_2486 | acetyltransferase | chromosome:2871995-2872499 | 1448A-rhpS-MM | 1448A-WT-MM | OK | 51.3787 | 147.737 | 1.52379 | 1.23255 | 0.124 | 0.468055 |
| PSPPH_1770 | transthyretin family protein | chromosome:2067988-2068342 | 1448A-rhpS-MM | 1448A-WT-MM | OK | 53.6512 | 153.975 | 1.52102 | 0.855122 | 0.24585 | 0.596121 |
| PSPPH_0044 | lipoprotein | chromosome:46834-47269 | 1448A-rhpS-MM | 1448A-WT-MM | NOTEST | 20.1375 | 57.6846 | 1.51831 | 0 | 1 | 1 |
| rubA | rubredoxin | chromosome:5797475-5797643 | 1448A-rhpS-MM | 1448A-WT-MM | OK | 216.142 | 616.969 | 1.51322 | 1.16779 | 0.3917 | 0.709635 |
| PSPPH_1491 | major facilitator family transporter | chromosome:1733166-1733592 | 1448A-rhpS-MM | 1448A-WT-MM | NOTEST | 18.8351 | 53.6197 | 1.50934 | 0 | 1 | 1 |
| PSPPH_2847 | general secretion pathway protein GspK | chromosome:3291451-3292171 | 1448A-rhpS-MM | 1448A-WT-MM | NOTEST | 16.7776 | 47.6643 | 1.50637 | 0 | 1 | 1 |
| PSPPH_1906 | LuxR family transcriptional regulator | chromosome:2209435-2213944 | 1448A-rhpS-MM | 1448A-WT-MM | OK | 58.9278 | 166.556 | 1.49899 | 1.3301 | 0.0885 | 0.410817 |
| PSPPH_2231 | nicotinamide mononucleotide transporter PnuC | chromosome:2589656-2592063 | 1448A-rhpS-MM | 1448A-WT-MM | OK | 35.8657 | 100.736 | 1.4899 | 0.924149 | 0.2974 | 0.6413 |
| PSPPH_1629 | tetraacyldisaccharide 4'-kinase | chromosome:1889240-1892305 | 1448A-rhpS-MM | 1448A-WT-MM | OK | 164.45 | 459.48 | 1.48236 | 0.986521 | 0.40555 | 0.720556 |
| PSPPH_1387 | amino acid ABC transporter permease | chromosome:1609057-1610478 | 1448A-rhpS-MM | 1448A-WT-MM | NOTEST | 18.9774 | 52.9865 | 1.48135 | 0 | 1 | 1 |
| PSPPH_2576 | shikimate 5-dehydrogenase | chromosome:2970064-2970880 | 1448A-rhpS-MM | 1448A-WT-MM | OK | 77.6853 | 216.895 | 1.48128 | 1.9492 | 0.0214 | 0.207975 |
| PSPPH_A0110 | hypothetical protein | large_plasmid:94896-95862 | 1448A-rhpS-MM | 1448A-WT-MM | OK | 273.009 | 761.844 | 1.48055 | 3.45492 | 0.0001 | 0.00434286 |
| PSPPH_3757 | hydrolase | chromosome:4309546-4310134 | 1448A-rhpS-MM | 1448A-WT-MM | OK | 179.199 | 499.872 | 1.47999 | 2.25468 | 0.0068 | 0.101333 |
| PSPPH_3497 | transposase, truncated | chromosome:4039253-4039592 | 1448A-rhpS-MM | 1448A-WT-MM | NOTEST | 24.086 | 67.0912 | 1.47793 | 0 | 1 | 1 |
| PSPPH_1396 | hypothetical protein | chromosome:1619993-1620485 | 1448A-rhpS-MM | 1448A-WT-MM | NOTEST | 13.224 | 36.7517 | 1.47465 | 0 | 1 | 1 |
| PSPPH_4957 | prophage PSPPH06, TP901 family tail tape measure protein | chromosome:5628151-5632456 | 1448A-rhpS-MM | 1448A-WT-MM | NOTEST | 5.33334 | 14.8175 | 1.47419 | 0 | 1 | 1 |
| PSPPH_4090 | hypothetical protein | chromosome:4671940-4673883 | 1448A-rhpS-MM | 1448A-WT-MM | OK | 37.8948 | 105.033 | 1.47076 | 0.734975 | 0.46215 | 0.76115 |
| PSPPH_5067 | hypothetical protein | chromosome:5746193-5747983 | 1448A-rhpS-MM | 1448A-WT-MM | OK | 38.3519 | 106.131 | 1.46848 | 0.700435 | 0.4543 | 0.759087 |
| PSPPH_1345 | mutT/nudix family protein | chromosome:1562811-1563408 | 1448A-rhpS-MM | 1448A-WT-MM | OK | 140.101 | 384.422 | 1.45622 | 2.11597 | 0.0085 | 0.120748 |
| PSPPH_3487 | transcriptional regulator | chromosome:4026795-4027359 | 1448A-rhpS-MM | 1448A-WT-MM | NOTEST | 14.2555 | 38.945 | 1.44992 | 0 | 1 | 1 |
| PSPPH_2563 | GntR family transcriptional regulator | chromosome:2954691-2955612 | 1448A-rhpS-MM | 1448A-WT-MM | OK | 59.7796 | 162.813 | 1.44549 | 1.84139 | 0.0229 | 0.218232 |
| PSPPH_1923 | pyoverdine sidechain peptide synthetase I, epsilon-Lys module | chromosome:2239720-2250972 | 1448A-rhpS-MM | 1448A-WT-MM | NOTEST | 2.19099 | 5.92495 | 1.43522 | 0 | 1 | 1 |
| PSPPH_2281 | hypothetical protein | chromosome:2647752-2649491 | 1448A-rhpS-MM | 1448A-WT-MM | NOTEST | 6.90865 | 18.6465 | 1.43243 | 0 | 1 | 1 |
| PSPPH_2839 | ABC transporter substrate-binding protein | chromosome:3282562-3286471 | 1448A-rhpS-MM | 1448A-WT-MM | NOTEST | 13.3819 | 35.9113 | 1.42416 | 0 | 1 | 1 |
| PSPPH_4806 | Rieske (2Fe-2S) domain-containing protein | chromosome:5453871-5455104 | 1448A-rhpS-MM | 1448A-WT-MM | OK | 58.3724 | 156.379 | 1.42169 | 2.12138 | 0.0104 | 0.138667 |
| PSPPH_1609 | Rieske (2Fe-2S) domain-containing protein | chromosome:1870154-1873726 | 1448A-rhpS-MM | 1448A-WT-MM | NOTEST | 35.71 | 95.6389 | 1.42127 | 0 | 1 | 1 |
| PSPPH_2371 | hypothetical protein | chromosome:2748809-2750141 | 1448A-rhpS-MM | 1448A-WT-MM | NOTEST | 6.06895 | 16.2163 | 1.41792 | 0 | 1 | 1 |
| PSPPH_4770 | hypothetical protein | chromosome:5414454-5417215 | 1448A-rhpS-MM | 1448A-WT-MM | NOTEST | 26.2553 | 70.1413 | 1.41765 | 0 | 1 | 1 |
| accC1 | acetyl-CoA carboxylase biotin carboxylase subunit | chromosome:2838562-2841952 | 1448A-rhpS-MM | 1448A-WT-MM | NOTEST | 8.18666 | 21.8661 | 1.41735 | 0 | 1 | 1 |
| avrD1 | syringolide biosynthetic protein AvrD1 | large_plasmid:96850-97786 | 1448A-rhpS-MM | 1448A-WT-MM | OK | 187.842 | 499.923 | 1.41219 | 2.80908 | 0.00035 | 0.0122299 |
| PSPPH_0907 | hypothetical protein | chromosome:1080937-1082203 | 1448A-rhpS-MM | 1448A-WT-MM | NOTEST | 11.1815 | 29.7051 | 1.4096 | 0 | 1 | 1 |
| kdtA | 3-deoxy-D-manno-octulosonic-acid transferase | chromosome:625561-626842 | 1448A-rhpS-MM | 1448A-WT-MM | OK | 37.4489 | 99.2107 | 1.40557 | 1.66497 | 0.03045 | 0.252229 |
| PSPPH_3459 | hypothetical protein | chromosome:3996811-3998140 | 1448A-rhpS-MM | 1448A-WT-MM | NOTEST | 11.1066 | 29.3733 | 1.4031 | 0 | 1 | 1 |
| PSPPH_1244 | AsnC family transcriptional regulator | chromosome:1451584-1452064 | 1448A-rhpS-MM | 1448A-WT-MM | OK | 65.8086 | 173.479 | 1.39842 | 1.30696 | 0.13935 | 0.488174 |
| PSPPH_2636 | OMP85 family outer membrane protein | chromosome:3041637-3043362 | 1448A-rhpS-MM | 1448A-WT-MM | NOTEST | 16.8143 | 44.3138 | 1.39806 | 0 | 1 | 1 |
| PSPPH_4314 | hypothetical protein | chromosome:4925434-4927721 | 1448A-rhpS-MM | 1448A-WT-MM | OK | 70.0832 | 184.332 | 1.39516 | 0.948482 | 0.2274 | 0.577042 |
| PSPPH_3641 | hypothetical protein | chromosome:4186273-4188138 | 1448A-rhpS-MM | 1448A-WT-MM | NOTEST | 12.1357 | 31.8842 | 1.39359 | 0 | 1 | 1 |
| noxB | NADH:flavin oxidoreductase | chromosome:5462683-5464744 | 1448A-rhpS-MM | 1448A-WT-MM | NOTEST | 7.32134 | 19.2075 | 1.39149 | 0 | 1 | 1 |
| kptA | RNA 2'-phosphotransferase | chromosome:4994086-4994653 | 1448A-rhpS-MM | 1448A-WT-MM | OK | 39.63 | 103.942 | 1.39111 | 0.988763 | 0.1731 | 0.517937 |
| PSPPH_4009 | hypothetical protein | chromosome:4579891-4580131 | 1448A-rhpS-MM | 1448A-WT-MM | NOTEST | 32.2798 | 84.6205 | 1.39037 | 0 | 1 | 1 |
| PSPPH_2653 | lipopolysaccharide core biosynthesis domain-containing protein | chromosome:3076505-3077354 | 1448A-rhpS-MM | 1448A-WT-MM | OK | 135.5 | 352.867 | 1.38083 | 2.33813 | 0.00375 | 0.0662791 |
| PSPPH_4285 | ISPsy19, transposase | chromosome:4891723-4892918 | 1448A-rhpS-MM | 1448A-WT-MM | NOTEST | 2.95985 | 7.69439 | 1.37828 | 0 | 1 | 1 |
| PSPPH_3756 | hypothetical protein | chromosome:4309086-4309545 | 1448A-rhpS-MM | 1448A-WT-MM | OK | 182.059 | 472.448 | 1.37575 | 1.79184 | 0.0285 | 0.242011 |
| PSPPH_2147 | hypothetical protein | chromosome:2501090-2502199 | 1448A-rhpS-MM | 1448A-WT-MM | NOTEST | 16.352 | 42.1956 | 1.36762 | 0 | 1 | 1 |
| PSPPH_2711 | deoxycytidine triphosphate deaminase | chromosome:3138006-3138519 | 1448A-rhpS-MM | 1448A-WT-MM | NOTEST | 27.3338 | 70.5235 | 1.36742 | 0 | 1 | 1 |
| PSPPH_4413 | 3-methyladenine DNA glycosylase | chromosome:5036314-5036986 | 1448A-rhpS-MM | 1448A-WT-MM | NOTEST | 22.3606 | 57.4844 | 1.36221 | 0 | 1 | 1 |
| PSPPH_1203 | hypothetical protein | chromosome:1414291-1415290 | 1448A-rhpS-MM | 1448A-WT-MM | NOTEST | 3.16987 | 8.14584 | 1.36164 | 0 | 1 | 1 |
| PSPPH_0615 | hypothetical protein | chromosome:727228-728077 | 1448A-rhpS-MM | 1448A-WT-MM | OK | 42.0369 | 107.575 | 1.35561 | 1.36897 | 0.09905 | 0.435133 |
| dnaK | molecular chaperone DnaK | chromosome:4795214-4797131 | 1448A-rhpS-MM | 1448A-WT-MM | OK | 2293.63 | 5869.37 | 1.35557 | 4.82649 | 5.00E-05 | 0.00230303 |
| PSPPH_3160 | hypothetical protein | chromosome:3662678-3663736 | 1448A-rhpS-MM | 1448A-WT-MM | OK | 165.774 | 423.961 | 1.35472 | 2.32351 | 0.0038 | 0.0663908 |
| PSPPH_3403 | hypothetical protein | chromosome:3936649-3937838 | 1448A-rhpS-MM | 1448A-WT-MM | NOTEST | 23.0488 | 58.9161 | 1.35397 | 0 | 1 | 1 |
| PSPPH_2167 | luciferase | chromosome:2521875-2522838 | 1448A-rhpS-MM | 1448A-WT-MM | NOTEST | 3.22801 | 8.25105 | 1.35393 | 0 | 1 | 1 |
| cueR | Cu(I)-responsive transcriptional regulator | chromosome:5280482-5283076 | 1448A-rhpS-MM | 1448A-WT-MM | OK | 40.8752 | 104.088 | 1.34851 | 0.715412 | 0.3107 | 0.652909 |
| PSPPH_A0043 | hypothetical protein | large_plasmid:36472-36583 | 1448A-rhpS-MM | 1448A-WT-MM | OK | 1078.18 | 2738.9 | 1.345 | 6.53335 | 0.48735 | 0.773486 |
| PSPPH_3735 | ferric iron reductase FhuF | chromosome:4282123-4284450 | 1448A-rhpS-MM | 1448A-WT-MM | NOTEST | 24.5414 | 62.2715 | 1.34336 | 0 | 1 | 1 |
| PSPPH_0111 | acetyltransferase | chromosome:128274-129446 | 1448A-rhpS-MM | 1448A-WT-MM | NOTEST | 18.6958 | 47.418 | 1.34272 | 0 | 1 | 1 |
| PSPPH_0607 | hypothetical protein | chromosome:717657-717912 | 1448A-rhpS-MM | 1448A-WT-MM | OK | 201.693 | 511.081 | 1.34139 | 1.03761 | 0.1425 | 0.492833 |
| PSPPH_4244 | hypothetical protein | chromosome:4841518-4842970 | 1448A-rhpS-MM | 1448A-WT-MM | NOTEST | 29.0153 | 73.5128 | 1.34118 | 0 | 1 | 1 |
| PSPPH_2819 | Rhs family protein | chromosome:3264779-3266623 | 1448A-rhpS-MM | 1448A-WT-MM | NOTEST | 8.29009 | 20.9953 | 1.34061 | 0 | 1 | 1 |
| recQ | ATP-dependent DNA helicase RecQ | chromosome:1764414-1766544 | 1448A-rhpS-MM | 1448A-WT-MM | OK | 72.1729 | 182.564 | 1.33887 | 2.75245 | 0.00095 | 0.0255575 |
| PSPPH_3140 | aminotransferase | chromosome:3642397-3643519 | 1448A-rhpS-MM | 1448A-WT-MM | NOTEST | 16.7431 | 42.3344 | 1.33826 | 0 | 1 | 1 |
| PSPPH_1034 | hypothetical protein | chromosome:1222500-1223130 | 1448A-rhpS-MM | 1448A-WT-MM | NOTEST | 25.0117 | 63.2093 | 1.33753 | 0 | 1 | 1 |
| PSPPH_1543 | hypothetical protein | chromosome:1795125-1795663 | 1448A-rhpS-MM | 1448A-WT-MM | OK | 145.509 | 367.653 | 1.33724 | 0.794454 | 0.2283 | 0.577827 |
| PSPPH_2909 | luciferase | chromosome:3379829-3380930 | 1448A-rhpS-MM | 1448A-WT-MM | NOTEST | 8.07881 | 20.3855 | 1.33533 | 0 | 1 | 1 |
| PSPPH_2375 | NtaA/SnaA/SoxA family monooxygenase | chromosome:2752785-2754186 | 1448A-rhpS-MM | 1448A-WT-MM | NOTEST | 3.58674 | 9.04025 | 1.33369 | 0 | 1 | 1 |
| PSPPH_0322 | ABC transporter permease | chromosome:366774-367641 | 1448A-rhpS-MM | 1448A-WT-MM | NOTEST | 3.8498 | 9.61491 | 1.32049 | 0 | 1 | 1 |
| PSPPH_2072 | serine/threonine protein kinase | chromosome:2429362-2431024 | 1448A-rhpS-MM | 1448A-WT-MM | NOTEST | 6.2197 | 15.5187 | 1.31909 | 0 | 1 | 1 |
| PSPPH_4534 | glycerophosphodiester phosphodiesterase | chromosome:5168942-5171556 | 1448A-rhpS-MM | 1448A-WT-MM | NOTEST | 12.8995 | 32.148 | 1.31742 | 0 | 1 | 1 |
| tliE | ABC transporter substrate-binding protein | chromosome:3570291-3571578 | 1448A-rhpS-MM | 1448A-WT-MM | NOTEST | 6.2518 | 15.5708 | 1.3165 | 0 | 1 | 1 |
| PSPPH_2761 | hypothetical protein | chromosome:3196863-3199066 | 1448A-rhpS-MM | 1448A-WT-MM | NOTEST | 22.4754 | 55.9709 | 1.31633 | 0 | 1 | 1 |
| PSPPH_4469 | branched-chain amino acid ABC transporter substrate-binding protein | chromosome:5105727-5106993 | 1448A-rhpS-MM | 1448A-WT-MM | NOTEST | 15.5008 | 38.3782 | 1.30794 | 0 | 1 | 1 |
| PSPPH_4520 | lipopolysaccharide biosynthesis protein | chromosome:5149767-5151574 | 1448A-rhpS-MM | 1448A-WT-MM | OK | 89.5964 | 221.797 | 1.30773 | 1.16321 | 0.12285 | 0.46721 |
| PSPPH_2422 | ThiJ/PfpI family protein | chromosome:2800938-2801544 | 1448A-rhpS-MM | 1448A-WT-MM | NOTEST | 11.5693 | 28.5683 | 1.30411 | 0 | 1 | 1 |
| PSPPH_4358 | hypothetical protein | chromosome:4980456-4980933 | 1448A-rhpS-MM | 1448A-WT-MM | OK | 89.4262 | 220.71 | 1.30338 | 1.31278 | 0.08395 | 0.39752 |
| PSPPH_2408 | zinc-binding oxidoreductase | chromosome:2784515-2785499 | 1448A-rhpS-MM | 1448A-WT-MM | OK | 68.3836 | 168.596 | 1.30184 | 1.79305 | 0.0215 | 0.207975 |
| PSPPH_0291 | cation transport protein chaC | chromosome:334659-335328 | 1448A-rhpS-MM | 1448A-WT-MM | OK | 82.3785 | 202.921 | 1.30058 | 1.55489 | 0.04745 | 0.308222 |
| PSPPH_1855 | thiamine biosynthesis lipoprotein | chromosome:2156449-2157442 | 1448A-rhpS-MM | 1448A-WT-MM | OK | 56.0285 | 137.846 | 1.29882 | 1.59658 | 0.0399 | 0.284732 |
| hisP | histidine ABC transporter ATP-binding protein | chromosome:4062269-4063034 | 1448A-rhpS-MM | 1448A-WT-MM | OK | 74.8163 | 184.033 | 1.29854 | 1.55473 | 0.04285 | 0.294736 |
| PSPPH_3155 | LysR family transcriptional regulator | chromosome:3658548-3659439 | 1448A-rhpS-MM | 1448A-WT-MM | NOTEST | 10.0326 | 24.5945 | 1.29364 | 0 | 1 | 1 |
| PSPPH_2866 | hypothetical protein | chromosome:3310791-3310995 | 1448A-rhpS-MM | 1448A-WT-MM | OK | 78.0639 | 191.215 | 1.29247 | 1.0314 | 0.47995 | 0.768565 |
| PSPPH_1897 | chorismate mutase | chromosome:2200418-2201546 | 1448A-rhpS-MM | 1448A-WT-MM | OK | 221.362 | 542.131 | 1.29224 | 2.98001 | 0.0003 | 0.0108571 |
| pin | hypothetical protein | chromosome:4811269-4811839 | 1448A-rhpS-MM | 1448A-WT-MM | OK | 539.255 | 1319.97 | 1.29146 | 2.96247 | 0.0002 | 0.00810667 |
| phnC | phosphonate ABC transporter ATP-binding protein | chromosome:3410640-3411474 | 1448A-rhpS-MM | 1448A-WT-MM | NOTEST | 4.40453 | 10.754 | 1.28782 | 0 | 1 | 1 |
| PSPPH_A0034 | PilT domain-containing protein | large_plasmid:29009-29924 | 1448A-rhpS-MM | 1448A-WT-MM | OK | 210.07 | 512.414 | 1.28644 | 1.28753 | 0.07895 | 0.387735 |
| PSPPH_2598 | precorrin 6A synthase | chromosome:2994112-2996088 | 1448A-rhpS-MM | 1448A-WT-MM | OK | 87.4861 | 213.277 | 1.2856 | 1.49062 | 0.05215 | 0.326115 |
| mmsA1 | methylmalonate-semialdehyde dehydrogenase | chromosome:3701204-3702707 | 1448A-rhpS-MM | 1448A-WT-MM | NOTEST | 21.5208 | 52.4152 | 1.28425 | 0 | 1 | 1 |
| PSPPH_2159 | isocitrate/isopropylmalate family dehydrogenase | chromosome:2513941-2517414 | 1448A-rhpS-MM | 1448A-WT-MM | OK | 40.0717 | 97.4949 | 1.28274 | 1.2514 | 0.1164 | 0.457362 |
| PSPPH_0286 | GntR family transcriptional regulator | chromosome:329238-329946 | 1448A-rhpS-MM | 1448A-WT-MM | NOTEST | 8.07106 | 19.508 | 1.27323 | 0 | 1 | 1 |
| PSPPH_4804 | hypothetical protein | chromosome:5451806-5452103 | 1448A-rhpS-MM | 1448A-WT-MM | OK | 199.682 | 481.854 | 1.27089 | 1.19622 | 0.10745 | 0.449928 |
| PSPPH_2341 | pyridine nucleotide-disulfide oxidoreductase domain-containing protein | chromosome:2714884-2716117 | 1448A-rhpS-MM | 1448A-WT-MM | OK | 62.6085 | 150.931 | 1.26946 | 1.8893 | 0.0149 | 0.171576 |
| PSPPH_0156 | hypothetical protein | chromosome:184190-184721 | 1448A-rhpS-MM | 1448A-WT-MM | NOTEST | 28.3664 | 68.1915 | 1.26541 | 0 | 1 | 1 |
| PSPPH_2123 | hypothetical protein | chromosome:2481265-2481550 | 1448A-rhpS-MM | 1448A-WT-MM | OK | 136.133 | 326.511 | 1.26211 | 0.948982 | 0.2002 | 0.548295 |
| PSPPH_1138 | GIY-YIG nuclease superfamily protein | chromosome:1336652-1337439 | 1448A-rhpS-MM | 1448A-WT-MM | OK | 36.7352 | 88.0901 | 1.26182 | 0.525606 | 0.46505 | 0.762375 |
| PSPPH_3931 | 3-oxoacyl-ACP reductase | chromosome:4491835-4492585 | 1448A-rhpS-MM | 1448A-WT-MM | NOTEST | 27.1027 | 64.8681 | 1.25908 | 0 | 1 | 1 |
| PSPPH_2735 | hypothetical protein | chromosome:3166229-3167111 | 1448A-rhpS-MM | 1448A-WT-MM | NOTEST | 18.0948 | 43.1868 | 1.25502 | 0 | 1 | 1 |
| PSPPH_4693 | hypothetical protein | chromosome:5327863-5328253 | 1448A-rhpS-MM | 1448A-WT-MM | OK | 126.368 | 301.593 | 1.25497 | 1.20848 | 0.09165 | 0.418342 |
| PSPPH_2957 | Mn2+/Fe2+ transporter | chromosome:3430866-3432168 | 1448A-rhpS-MM | 1448A-WT-MM | NOTEST | 8.05703 | 19.2093 | 1.25349 | 0 | 1 | 1 |
| PSPPH_4490 | hypothetical protein | chromosome:5124437-5124926 | 1448A-rhpS-MM | 1448A-WT-MM | OK | 51.4373 | 122.615 | 1.25325 | 1.06575 | 0.2087 | 0.560735 |
| PSPPH_3524 | succinylglutamate desuccinylase/aspartoacylase | chromosome:4063056-4064169 | 1448A-rhpS-MM | 1448A-WT-MM | OK | 39.158 | 93.3347 | 1.25311 | 1.37599 | 0.06255 | 0.352133 |
| PSPPH_1551 | hypothetical protein | chromosome:1803797-1804247 | 1448A-rhpS-MM | 1448A-WT-MM | OK | 60.2655 | 143.433 | 1.25098 | 0.979898 | 0.19735 | 0.548217 |
| gabT3 | 4-aminobutyrate aminotransferase | chromosome:5711646-5712951 | 1448A-rhpS-MM | 1448A-WT-MM | NOTEST | 30.888 | 73.1586 | 1.24398 | 0 | 1 | 1 |
| rimI | ribosomal-protein-alanine acetyltransferase | chromosome:1060302-1061551 | 1448A-rhpS-MM | 1448A-WT-MM | NOTEST | 30.0237 | 71.0887 | 1.24352 | 0 | 1 | 1 |
| PSPPH_3037 | TonB-dependent siderophore receptor | chromosome:3527023-3529150 | 1448A-rhpS-MM | 1448A-WT-MM | NOTEST | 14.6257 | 34.6013 | 1.24232 | 0 | 1 | 1 |
| PSPPH_1652 | lipoprotein | chromosome:1913192-1913795 | 1448A-rhpS-MM | 1448A-WT-MM | NOTEST | 22.8464 | 54.0175 | 1.24146 | 0 | 1 | 1 |
| PSPPH_3323 | acetyltransferase | chromosome:3844257-3845118 | 1448A-rhpS-MM | 1448A-WT-MM | OK | 42.0671 | 99.2125 | 1.23783 | 1.26516 | 0.10045 | 0.438772 |
| PSPPH_0642 | LuxR family transcriptional regulator | chromosome:758094-760733 | 1448A-rhpS-MM | 1448A-WT-MM | OK | 55.6584 | 130.959 | 1.23445 | 1.01328 | 0.16635 | 0.514081 |
| PSPPH_4485 | hypothetical protein | chromosome:5120871-5121882 | 1448A-rhpS-MM | 1448A-WT-MM | OK | 33.0203 | 77.4669 | 1.23023 | 1.13068 | 0.11595 | 0.457362 |
| PSPPH_3192 | iolB protein | chromosome:3702735-3703545 | 1448A-rhpS-MM | 1448A-WT-MM | NOTEST | 6.81666 | 15.9788 | 1.22902 | 0 | 1 | 1 |
| PSPPH_4647 | transporter | chromosome:5285297-5286326 | 1448A-rhpS-MM | 1448A-WT-MM | NOTEST | 23.183 | 54.2989 | 1.22785 | 0 | 1 | 1 |
| mutT1 | mutT/nudix family protein | chromosome:1562109-1562661 | 1448A-rhpS-MM | 1448A-WT-MM | OK | 114.456 | 267.873 | 1.22676 | 1.54089 | 0.0446 | 0.297987 |
| PSPPH_2710 | fatty acid desaturase | chromosome:3136985-3138002 | 1448A-rhpS-MM | 1448A-WT-MM | NOTEST | 8.33472 | 19.4912 | 1.22561 | 0 | 1 | 1 |
| PSPPH_2399 | hypothetical protein | chromosome:2776591-2778045 | 1448A-rhpS-MM | 1448A-WT-MM | NOTEST | 16.8887 | 39.4773 | 1.22497 | 0 | 1 | 1 |
| PSPPH_3858 | hypothetical protein | chromosome:4414002-4414656 | 1448A-rhpS-MM | 1448A-WT-MM | NOTEST | 13.6805 | 31.9401 | 1.22324 | 0 | 1 | 1 |
| PSPPH_4066 | lipoprotein | chromosome:4648428-4649787 | 1448A-rhpS-MM | 1448A-WT-MM | OK | 129.103 | 301.33 | 1.22283 | 2.59751 | 0.00065 | 0.01976 |
| PSPPH_3047 | major facilitator family transporter | chromosome:3540879-3542166 | 1448A-rhpS-MM | 1448A-WT-MM | NOTEST | 14.684 | 34.256 | 1.22211 | 0 | 1 | 1 |
| PSPPH_5089 | hypothetical protein | chromosome:5773891-5778270 | 1448A-rhpS-MM | 1448A-WT-MM | NOTEST | 10.2903 | 23.9115 | 1.21642 | 0 | 1 | 1 |
| PSPPH_1530 | polygalacturonase | chromosome:1784948-1785383 | 1448A-rhpS-MM | 1448A-WT-MM | OK | 65.6681 | 152.511 | 1.21565 | 0.969084 | 0.2108 | 0.562627 |
| PSPPH_1195 | DNA-binding heavy metal response regulator | chromosome:1402135-1404167 | 1448A-rhpS-MM | 1448A-WT-MM | NOTEST | 32.6171 | 75.7214 | 1.21507 | 0 | 1 | 1 |
| PSPPH_4068 | lipoprotein | chromosome:4650742-4652176 | 1448A-rhpS-MM | 1448A-WT-MM | NOTEST | 23.2274 | 53.8079 | 1.21199 | 0 | 1 | 1 |
| PSPPH_5039 | D-isomer specific 2-hydroxyacid dehydrogenase | chromosome:5710432-5711398 | 1448A-rhpS-MM | 1448A-WT-MM | NOTEST | 19.696 | 45.5934 | 1.21092 | 0 | 1 | 1 |
| dxnG | asnC family transcriptional regulator | chromosome:3659568-3660270 | 1448A-rhpS-MM | 1448A-WT-MM | OK | 74.7356 | 172.853 | 1.20968 | 1.4283 | 0.0566 | 0.33672 |
| fliD | flagellar hook-associated protein FliD | chromosome:3917833-3919312 | 1448A-rhpS-MM | 1448A-WT-MM | OK | 948.387 | 2191.05 | 1.20807 | 4.12005 | 5.00E-05 | 0.00230303 |
| tonB4 | ferric siderophore ABC transporter substrate-binding protein | chromosome:5726419-5727232 | 1448A-rhpS-MM | 1448A-WT-MM | NOTEST | 3.13673 | 7.24663 | 1.20805 | 0 | 1 | 1 |
| PSPPH_3932 | glucose 1-dehydrogenase | chromosome:4492616-4493378 | 1448A-rhpS-MM | 1448A-WT-MM | NOTEST | 27.1346 | 62.6739 | 1.20773 | 0 | 1 | 1 |
| rbsK | ribokinase | chromosome:2486672-2487994 | 1448A-rhpS-MM | 1448A-WT-MM | OK | 53.3932 | 123.288 | 1.20731 | 0.948796 | 0.2906 | 0.636083 |
| PSPPH_1516 | hypothetical protein | chromosome:1763235-1763640 | 1448A-rhpS-MM | 1448A-WT-MM | OK | 71.3951 | 164.64 | 1.20542 | 0.951358 | 0.2108 | 0.562627 |
| PSPPH_2717 | hypothetical protein | chromosome:3144477-3146645 | 1448A-rhpS-MM | 1448A-WT-MM | NOTEST | 27.8149 | 64.1085 | 1.20466 | 0 | 1 | 1 |
| PSPPH_0334 | D-methionine-binding lipoprotein MetQ | chromosome:378442-380992 | 1448A-rhpS-MM | 1448A-WT-MM | NOTEST | 11.0742 | 25.4634 | 1.20123 | 0 | 1 | 1 |
| PSPPH_2557 | spermidine/putrescine ABC transporter permease | chromosome:2948350-2950317 | 1448A-rhpS-MM | 1448A-WT-MM | OK | 39.6008 | 90.9919 | 1.20021 | 0.980444 | 0.17785 | 0.523899 |
| proC | pyrroline-5-carboxylate reductase | chromosome:530453-531981 | 1448A-rhpS-MM | 1448A-WT-MM | OK | 204.429 | 469.539 | 1.19965 | 2.31929 | 0.002 | 0.044058 |
| PSPPH_3713 | hypothetical protein | chromosome:4260013-4260193 | 1448A-rhpS-MM | 1448A-WT-MM | OK | 323.691 | 743.085 | 1.19891 | 0.698054 | 0.31945 | 0.659389 |
| PSPPH_4225 | hypothetical protein | chromosome:4818335-4818797 | 1448A-rhpS-MM | 1448A-WT-MM | NOTEST | 25.0116 | 57.2689 | 1.19515 | 0 | 1 | 1 |
| PSPPH_1206 | acetyltransferase | chromosome:1416498-1416960 | 1448A-rhpS-MM | 1448A-WT-MM | OK | 105.857 | 242.308 | 1.19472 | 1.20794 | 0.09655 | 0.429556 |
| PSPPH_0892 | HD domain-containing protein | chromosome:1064632-1065292 | 1448A-rhpS-MM | 1448A-WT-MM | OK | 107.295 | 245.451 | 1.19385 | 1.52504 | 0.0379 | 0.275341 |
| PSPPH_2505 | senescence marker protein-30 family protein | chromosome:2891757-2892630 | 1448A-rhpS-MM | 1448A-WT-MM | OK | 42.5952 | 97.3797 | 1.19293 | 1.1957 | 0.0982 | 0.433277 |
| PSPPH_0115 | lipoprotein | chromosome:132506-133010 | 1448A-rhpS-MM | 1448A-WT-MM | OK | 33.7518 | 77.0028 | 1.18995 | 0.802455 | 0.32235 | 0.662051 |
| dinF | DNA-damage-inducible protein F | chromosome:5053214-5055400 | 1448A-rhpS-MM | 1448A-WT-MM | NOTEST | 18.2634 | 41.6374 | 1.18893 | 0 | 1 | 1 |
| PSPPH_1705 | ABC transporter permease | chromosome:1971888-1972908 | 1448A-rhpS-MM | 1448A-WT-MM | NOTEST | 30.2394 | 68.938 | 1.18887 | 0 | 1 | 1 |
| PSPPH_4452 | multiple antibiotic resistance protein MarC | chromosome:5087279-5087876 | 1448A-rhpS-MM | 1448A-WT-MM | NOTEST | 26.7157 | 60.8602 | 1.18781 | 0 | 1 | 1 |
| PSPPH_1048 | luciferase | chromosome:1238661-1240481 | 1448A-rhpS-MM | 1448A-WT-MM | NOTEST | 7.41939 | 16.9006 | 1.1877 | 0 | 1 | 1 |
| htpG | heat shock protein 90 | chromosome:2335773-2337681 | 1448A-rhpS-MM | 1448A-WT-MM | OK | 1850.5 | 4209.83 | 1.18585 | 4.23896 | 5.00E-05 | 0.00230303 |
| PSPPH_0423 | hypothetical protein | chromosome:482647-490911 | 1448A-rhpS-MM | 1448A-WT-MM | NOTEST | 15.7988 | 35.91 | 1.18457 | 0 | 1 | 1 |
| PSPPH_2301 | LysR family transcriptional regulator | chromosome:2667567-2668476 | 1448A-rhpS-MM | 1448A-WT-MM | NOTEST | 19.9276 | 45.1503 | 1.17996 | 0 | 1 | 1 |
| PSPPH_0426 | intracellular septation protein A | chromosome:490912-491715 | 1448A-rhpS-MM | 1448A-WT-MM | NOTEST | 12.3198 | 27.8748 | 1.17798 | 0 | 1 | 1 |
| xylR | xylose operon regluatory protein | chromosome:2733267-2734359 | 1448A-rhpS-MM | 1448A-WT-MM | OK | 84.6015 | 191.359 | 1.17753 | 1.89109 | 0.0154 | 0.174435 |
| gspE2 | general secretion pathway protein GspE | chromosome:2532397-2537908 | 1448A-rhpS-MM | 1448A-WT-MM | NOTEST | 18.7205 | 42.287 | 1.1756 | 0 | 1 | 1 |
| PSPPH_0326 | hypothetical protein | chromosome:370541-371576 | 1448A-rhpS-MM | 1448A-WT-MM | NOTEST | 11.1965 | 25.2702 | 1.17439 | 0 | 1 | 1 |
| PSPPH_0048 | hypothetical protein | chromosome:49623-50679 | 1448A-rhpS-MM | 1448A-WT-MM | OK | 75.3862 | 169.596 | 1.16973 | 1.70995 | 0.0265 | 0.232832 |
| PSPPH_0927 | RNA polymerase sigma factor RpoE | chromosome:1101807-1103078 | 1448A-rhpS-MM | 1448A-WT-MM | NOTEST | 21.9034 | 49.2567 | 1.16917 | 0 | 1 | 1 |
| PSPPH_0157 | hypothetical protein | chromosome:184845-185394 | 1448A-rhpS-MM | 1448A-WT-MM | NOTEST | 12.8084 | 28.7855 | 1.16825 | 0 | 1 | 1 |
| PSPPH_4679 | hypothetical protein | chromosome:5315220-5316904 | 1448A-rhpS-MM | 1448A-WT-MM | NOTEST | 20.3697 | 45.7619 | 1.16772 | 0 | 1 | 1 |
| PSPPH_3264 | bem46 protein | chromosome:3782998-3783952 | 1448A-rhpS-MM | 1448A-WT-MM | NOTEST | 20.3746 | 45.7632 | 1.16741 | 0 | 1 | 1 |
| PSPPH_0488 | LamB/YcsF family protein | chromosome:556290-557055 | 1448A-rhpS-MM | 1448A-WT-MM | OK | 118.273 | 265.45 | 1.16632 | 1.82739 | 0.0186 | 0.191675 |
| PSPPH_1576 | glyoxalase | chromosome:1829880-1830258 | 1448A-rhpS-MM | 1448A-WT-MM | OK | 87.001 | 195.072 | 1.1649 | 0.934694 | 0.22945 | 0.578532 |
| PSPPH_1815 | acireductone dioxygenase | chromosome:2117980-2118526 | 1448A-rhpS-MM | 1448A-WT-MM | OK | 109.301 | 244.516 | 1.16162 | 1.38773 | 0.06925 | 0.3744 |
| PSPPH_4323 | hypothetical protein | chromosome:4937838-4938339 | 1448A-rhpS-MM | 1448A-WT-MM | NOTEST | 22.0117 | 49.0354 | 1.15555 | 0 | 1 | 1 |
| soxG | sarcosine oxidase subunit gamma | chromosome:5398510-5399143 | 1448A-rhpS-MM | 1448A-WT-MM | OK | 73.5907 | 163.51 | 1.15178 | 1.2636 | 0.09755 | 0.432923 |
| betC | choline sulfatase | chromosome:35723-37229 | 1448A-rhpS-MM | 1448A-WT-MM | NOTEST | 14.5894 | 32.4075 | 1.15141 | 0 | 1 | 1 |
| PSPPH_4028 | hypothetical protein | chromosome:4602130-4606623 | 1448A-rhpS-MM | 1448A-WT-MM | OK | 35.3283 | 78.4605 | 1.15114 | 0.974701 | 0.181 | 0.525038 |
| PSPPH_0538 | lipoprotein | chromosome:632078-633129 | 1448A-rhpS-MM | 1448A-WT-MM | OK | 153.6 | 340.642 | 1.14908 | 1.88489 | 0.01265 | 0.157607 |
| PSPPH_0304 | hypothetical protein | chromosome:344875-345411 | 1448A-rhpS-MM | 1448A-WT-MM | OK | 268.636 | 595.325 | 1.14802 | 0.663077 | 0.34225 | 0.679436 |
| PSPPH_2249 | acetyltransferase | chromosome:2612686-2613178 | 1448A-rhpS-MM | 1448A-WT-MM | OK | 80.1284 | 177.46 | 1.14711 | 1.01159 | 0.15885 | 0.506614 |
| dipZ | thiol:disulfide interchange protein | chromosome:5072933-5074754 | 1448A-rhpS-MM | 1448A-WT-MM | NOTEST | 25.1317 | 55.6135 | 1.14593 | 0 | 1 | 1 |
| PSPPH_3411 | DNA-binding transcriptional regulator CynR | chromosome:3944061-3944928 | 1448A-rhpS-MM | 1448A-WT-MM | NOTEST | 27.4913 | 60.8322 | 1.14586 | 0 | 1 | 1 |
| flgF | flagellar basal body rod protein FlgF | chromosome:3936649-3937838 | 1448A-rhpS-MM | 1448A-WT-MM | NOTEST | 24.9397 | 54.7815 | 1.13525 | 0 | 1 | 1 |
| PSPPH_2179 | general secretion pathway protein GspL | chromosome:2531229-2532393 | 1448A-rhpS-MM | 1448A-WT-MM | NOTEST | 31.6017 | 69.3856 | 1.13463 | 0 | 1 | 1 |
| PSPPH_1519 | MarR family transcriptional regulator | chromosome:1766637-1767072 | 1448A-rhpS-MM | 1448A-WT-MM | OK | 189.876 | 416.279 | 1.1325 | 1.50548 | 0.04725 | 0.308222 |
| groEL | molecular chaperone GroEL | chromosome:4659261-4660905 | 1448A-rhpS-MM | 1448A-WT-MM | OK | 4471.22 | 9801.72 | 1.13237 | 3.96969 | 5.00E-05 | 0.00230303 |
| hrpS | type III transcriptional regulator HrpS | chromosome:1486608-1487517 | 1448A-rhpS-MM | 1448A-WT-MM | OK | 66.6341 | 145.91 | 1.13075 | 1.42509 | 0.0538 | 0.332216 |
| PSPPH_3345 | hypothetical protein | chromosome:3873673-3874210 | 1448A-rhpS-MM | 1448A-WT-MM | NOTEST | 13.8965 | 30.3054 | 1.12485 | 0 | 1 | 1 |
| tldD | TldD protein | chromosome:4746894-4748334 | 1448A-rhpS-MM | 1448A-WT-MM | OK | 301.68 | 657.879 | 1.1248 | 3.10978 | 0.00015 | 0.00624658 |
| gufA | gufA protein | chromosome:2121524-2123023 | 1448A-rhpS-MM | 1448A-WT-MM | NOTEST | 28.9444 | 63.1135 | 1.12467 | 0 | 1 | 1 |
| PSPPH_0683 | hypothetical protein | chromosome:797455-797884 | 1448A-rhpS-MM | 1448A-WT-MM | OK | 77.4509 | 168.854 | 1.12442 | 0.858254 | 0.2209 | 0.570549 |
| cbiD | cobalt-precorrin-6A synthase | chromosome:5095634-5098665 | 1448A-rhpS-MM | 1448A-WT-MM | NOTEST | 23.272 | 50.7336 | 1.12435 | 0 | 1 | 1 |
| hopAN1 | HopAN1 protein | chromosome:518542-520470 | 1448A-rhpS-MM | 1448A-WT-MM | NOTEST | 12.8362 | 27.9613 | 1.12321 | 0 | 1 | 1 |
| PSPPH_0024 | SUA5/yciO/yrdC domain-containing protein | chromosome:29453-30011 | 1448A-rhpS-MM | 1448A-WT-MM | OK | 140.002 | 304.49 | 1.12095 | 1.52739 | 0.0441 | 0.296602 |
| PSPPH_2487 | TauD/TfdA family dioxygenase | chromosome:2872637-2873477 | 1448A-rhpS-MM | 1448A-WT-MM | OK | 115.309 | 250.782 | 1.12093 | 1.72211 | 0.02285 | 0.218232 |
| PSPPH_1417 | branched-chain amino acid ABC transporter substrate-binding protein | chromosome:1642175-1643369 | 1448A-rhpS-MM | 1448A-WT-MM | OK | 1251.88 | 2716.42 | 1.11762 | 3.81703 | 5.00E-05 | 0.00230303 |
| PSPPH_2263 | cointegrate resolution protein T | chromosome:2625147-2626158 | 1448A-rhpS-MM | 1448A-WT-MM | OK | 501.388 | 1087.48 | 1.11698 | 3.20766 | 5.00E-05 | 0.00230303 |
| PSPPH_2295 | LysR family transcriptional regulator | chromosome:2662830-2663733 | 1448A-rhpS-MM | 1448A-WT-MM | OK | 53.8062 | 116.556 | 1.11517 | 1.25687 | 0.0799 | 0.388044 |
| PSPPH_1029 | hypothetical protein | chromosome:1217930-1220164 | 1448A-rhpS-MM | 1448A-WT-MM | OK | 134.195 | 289.932 | 1.11139 | 1.50117 | 0.0429 | 0.294736 |
| htpX | heat shock protein HtpX | chromosome:4176465-4177353 | 1448A-rhpS-MM | 1448A-WT-MM | OK | 229.33 | 493.833 | 1.1066 | 2.31312 | 0.00235 | 0.0482703 |
| PSPPH_2579 | major facilitator family transporter | chromosome:2972456-2974510 | 1448A-rhpS-MM | 1448A-WT-MM | NOTEST | 26.9263 | 57.8849 | 1.10417 | 0 | 1 | 1 |
| mets | methionyl-tRNA synthetase | chromosome:2787398-2789397 | 1448A-rhpS-MM | 1448A-WT-MM | NOTEST | 6.64598 | 14.2744 | 1.10287 | 0 | 1 | 1 |
| PSPPH_3341 | VOMI family protein | chromosome:3869349-3870468 | 1448A-rhpS-MM | 1448A-WT-MM | NOTEST | 10.8004 | 23.1703 | 1.10119 | 0 | 1 | 1 |
| gabP | GABA permease | chromosome:5610172-5611564 | 1448A-rhpS-MM | 1448A-WT-MM | OK | 315.095 | 674.433 | 1.09789 | 3.00535 | 0.0001 | 0.00434286 |
| PSPPH_4627 | short chain dehydrogenase | chromosome:5264124-5264814 | 1448A-rhpS-MM | 1448A-WT-MM | OK | 67.3508 | 143.953 | 1.09583 | 1.1977 | 0.11705 | 0.457362 |
| PSPPH_5078 | hypothetical protein | chromosome:5760403-5760955 | 1448A-rhpS-MM | 1448A-WT-MM | NOTEST | 11.2323 | 23.9961 | 1.09515 | 0 | 1 | 1 |
| PSPPH_1574 | hypothetical protein | chromosome:1828119-1828515 | 1448A-rhpS-MM | 1448A-WT-MM | NOTEST | 18.4518 | 39.4188 | 1.09512 | 0 | 1 | 1 |
| PSPPH_3619 | cupin | chromosome:4167049-4167772 | 1448A-rhpS-MM | 1448A-WT-MM | NOTEST | 7.8584 | 16.7855 | 1.09491 | 0 | 1 | 1 |
| PSPPH_5169 | hypothetical protein | chromosome:5862693-5865964 | 1448A-rhpS-MM | 1448A-WT-MM | NOTEST | 7.91603 | 16.9051 | 1.09461 | 0 | 1 | 1 |
| PSPPH_3607 | SelT/selW/selH domain-containing protein | chromosome:4153518-4153821 | 1448A-rhpS-MM | 1448A-WT-MM | NOTEST | 29.7854 | 63.5964 | 1.09434 | 0 | 1 | 1 |
| PSPPH_0550 | phosphoserine phosphatase SerB | chromosome:645314-646529 | 1448A-rhpS-MM | 1448A-WT-MM | OK | 64.5448 | 137.777 | 1.09396 | 1.59782 | 0.03165 | 0.255746 |
| PSPPH_2381 | hypothetical protein | chromosome:2762768-2764508 | 1448A-rhpS-MM | 1448A-WT-MM | OK | 96.8527 | 206.677 | 1.09352 | 2.30373 | 0.00255 | 0.0503377 |
| iscR | iron-sulfur cluster assembly transcription factor IscR | chromosome:1519658-1520150 | 1448A-rhpS-MM | 1448A-WT-MM | OK | 92.7088 | 197.155 | 1.08855 | 1.07838 | 0.1428 | 0.493309 |
| PSPPH_2876 | hypothetical protein | chromosome:3320961-3322664 | 1448A-rhpS-MM | 1448A-WT-MM | NOTEST | 6.3151 | 13.4284 | 1.0884 | 0 | 1 | 1 |
| PSPPH_0860 | glutamyl-Q tRNA(Asp) synthetase | chromosome:1029846-1030734 | 1448A-rhpS-MM | 1448A-WT-MM | OK | 37.8339 | 80.3712 | 1.087 | 1.04281 | 0.15445 | 0.503178 |
| PSPPH_1848 | isochorismatase | chromosome:2144222-2144789 | 1448A-rhpS-MM | 1448A-WT-MM | NOTEST | 26.7468 | 56.6921 | 1.08378 | 0 | 1 | 1 |
| leuA | 2-isopropylmalate synthase | chromosome:1538624-1540295 | 1448A-rhpS-MM | 1448A-WT-MM | OK | 449.893 | 952.869 | 1.0827 | 3.37175 | 5.00E-05 | 0.00230303 |
| PSPPH_4936 | hypothetical protein | chromosome:5609639-5610125 | 1448A-rhpS-MM | 1448A-WT-MM | OK | 142.707 | 301.471 | 1.07897 | 1.26795 | 0.08815 | 0.410817 |
| brnQ | branched-chain amino acid transport system II carrier protein | chromosome:2333777-2335091 | 1448A-rhpS-MM | 1448A-WT-MM | NOTEST | 19.132 | 40.3936 | 1.07814 | 0 | 1 | 1 |
| PSPPH_2291 | nickel ABC transporter permease | chromosome:2655614-2658190 | 1448A-rhpS-MM | 1448A-WT-MM | NOTEST | 11.4815 | 24.2365 | 1.07787 | 0 | 1 | 1 |
| cobM | precorrin-4 C(11)-methyltransferase | chromosome:2579622-2580372 | 1448A-rhpS-MM | 1448A-WT-MM | OK | 77.2395 | 163.04 | 1.07782 | 1.32914 | 0.07255 | 0.381181 |
| PSPPH_1170 | hypothetical protein | chromosome:1374901-1375258 | 1448A-rhpS-MM | 1448A-WT-MM | OK | 267.085 | 562.618 | 1.07485 | 1.40461 | 0.05965 | 0.344725 |
| PSPPH_2361 | methyl-accepting chemotaxis protein | chromosome:2736946-2738575 | 1448A-rhpS-MM | 1448A-WT-MM | NOTEST | 10.3791 | 21.8347 | 1.07294 | 0 | 1 | 1 |
| PSPPH_0454 | gluconate transporter family protein | chromosome:516949-518407 | 1448A-rhpS-MM | 1448A-WT-MM | NOTEST | 14.5276 | 30.5194 | 1.07093 | 0 | 1 | 1 |
| PSPPH_2239 | glycosyl transferase family protein | chromosome:2597977-2599996 | 1448A-rhpS-MM | 1448A-WT-MM | OK | 38.7889 | 81.4805 | 1.07081 | 0.874329 | 0.36055 | 0.691528 |
| PSPPH_2358 | papain cysteine protease | chromosome:2734364-2735132 | 1448A-rhpS-MM | 1448A-WT-MM | OK | 170.645 | 358.098 | 1.06935 | 1.85401 | 0.01415 | 0.169674 |
| macB | macrolide efflux ABC transporter ATP-binding/permease | chromosome:2278900-2282267 | 1448A-rhpS-MM | 1448A-WT-MM | NOTEST | 4.16665 | 8.74324 | 1.06928 | 0 | 1 | 1 |
| PSPPH_2716 | hypothetical protein | chromosome:3142283-3144463 | 1448A-rhpS-MM | 1448A-WT-MM | OK | 36.6472 | 76.8089 | 1.06757 | 0.99424 | 0.1793 | 0.525038 |
| dppB | ABC transporter permease | chromosome:3175454-3178867 | 1448A-rhpS-MM | 1448A-WT-MM | NOTEST | 12.2639 | 25.6833 | 1.0664 | 0 | 1 | 1 |
| gspG2 | general secretion pathway protein GspG | chromosome:2529791-2530241 | 1448A-rhpS-MM | 1448A-WT-MM | NOTEST | 23.8995 | 50.0321 | 1.06587 | 0 | 1 | 1 |
| hslU | ATP-dependent protease ATP-binding subunit HslU | chromosome:434159-435497 | 1448A-rhpS-MM | 1448A-WT-MM | OK | 567.793 | 1187.27 | 1.06421 | 3.30963 | 5.00E-05 | 0.00230303 |
| PSPPH_3067 | hypothetical protein | chromosome:3562753-3563914 | 1448A-rhpS-MM | 1448A-WT-MM | NOTEST | 17.6959 | 36.9661 | 1.06279 | 0 | 1 | 1 |
| PSPPH_1172 | heme oxygenase | chromosome:1375356-1376426 | 1448A-rhpS-MM | 1448A-WT-MM | NOTEST | 30.8224 | 64.3414 | 1.06177 | 0 | 1 | 1 |
| rsmC | ribosomal RNA small subunit methyltransferase C | chromosome:1225013-1226012 | 1448A-rhpS-MM | 1448A-WT-MM | NOTEST | 28.8443 | 60.0415 | 1.05768 | 0 | 1 | 1 |
| PSPPH_4359 | hypothetical protein | chromosome:4981203-4981908 | 1448A-rhpS-MM | 1448A-WT-MM | OK | 871.841 | 1808.93 | 1.053 | 3.06659 | 5.00E-05 | 0.00230303 |
| PSPPH_0806 | STAS domain-containing protein | chromosome:951429-951720 | 1448A-rhpS-MM | 1448A-WT-MM | OK | 124.763 | 258.666 | 1.0519 | 0.776796 | 0.2627 | 0.612429 |
| PSPPH_3564 | hypothetical protein | chromosome:4104748-4110605 | 1448A-rhpS-MM | 1448A-WT-MM | NOTEST | 20.0594 | 41.5589 | 1.05087 | 0 | 1 | 1 |
| PSPPH_4624 | ABC transporter ATP-binding protein | chromosome:5260293-5261847 | 1448A-rhpS-MM | 1448A-WT-MM | NOTEST | 8.09333 | 16.7418 | 1.04865 | 0 | 1 | 1 |
| gltK | glucose ABC transporter ATP-binding protein | chromosome:1390491-1391658 | 1448A-rhpS-MM | 1448A-WT-MM | OK | 179.167 | 370.305 | 1.04741 | 2.24918 | 0.00335 | 0.060982 |
| PSPPH_2214 | TetR family transcriptional regulator | chromosome:2571220-2571766 | 1448A-rhpS-MM | 1448A-WT-MM | OK | 56.1278 | 115.727 | 1.04394 | 0.840233 | 0.2146 | 0.566522 |
| lolD | lipoprotein releasing system, ATP-binding protein LolD | chromosome:2161788-2162472 | 1448A-rhpS-MM | 1448A-WT-MM | OK | 59.0544 | 121.757 | 1.04389 | 1.09204 | 0.1496 | 0.496489 |
| PSPPH_0416 | lipoprotein | chromosome:476539-481262 | 1448A-rhpS-MM | 1448A-WT-MM | NOTEST | 21.1041 | 43.4302 | 1.04117 | 0 | 1 | 1 |
| PSPPH_3849 | thiazole biosynthesis adenylyltransferase ThiF | chromosome:4403811-4406302 | 1448A-rhpS-MM | 1448A-WT-MM | OK | 83.6073 | 171.897 | 1.03985 | 1.19066 | 0.1071 | 0.449928 |
| PSPPH_2733 | TetR family transcriptional regulator | chromosome:3164931-3165612 | 1448A-rhpS-MM | 1448A-WT-MM | NOTEST | 28.4575 | 58.4393 | 1.03813 | 0 | 1 | 1 |
| PSPPH_0950 | methyl-accepting chemotaxis protein | chromosome:1127558-1129616 | 1448A-rhpS-MM | 1448A-WT-MM | OK | 61.4695 | 126.22 | 1.038 | 1.89934 | 0.01025 | 0.137269 |
| PSPPH_2951 | hypothetical protein | chromosome:3425803-3426640 | 1448A-rhpS-MM | 1448A-WT-MM | NOTEST | 22.3839 | 45.9527 | 1.03769 | 0 | 1 | 1 |
| PSPPH_1370 | xenobiotic reductase | chromosome:1590063-1591170 | 1448A-rhpS-MM | 1448A-WT-MM | OK | 41.8499 | 85.8839 | 1.03716 | 1.18088 | 0.10525 | 0.446248 |
| PSPPH_2264 | cointegrate resolution protein S | chromosome:2626334-2627294 | 1448A-rhpS-MM | 1448A-WT-MM | OK | 221.191 | 453.324 | 1.03525 | 2.21344 | 0.00295 | 0.0557019 |
| PSPPH_3940 | hypothetical protein | chromosome:4499907-4503029 | 1448A-rhpS-MM | 1448A-WT-MM | NOTEST | 11.3478 | 23.2196 | 1.03294 | 0 | 1 | 1 |
| PSPPH_2823 | acetyltransferase | chromosome:3269170-3269746 | 1448A-rhpS-MM | 1448A-WT-MM | OK | 48.7456 | 99.7309 | 1.03277 | 0.786193 | 0.23915 | 0.589632 |
| PSPPH_1624 | ABC transporter permease | chromosome:1885081-1886817 | 1448A-rhpS-MM | 1448A-WT-MM | NOTEST | 34.396 | 70.3601 | 1.03252 | 0 | 1 | 1 |
| PSPPH_0337 | acyl-CoA dehydrogenase | chromosome:383836-385159 | 1448A-rhpS-MM | 1448A-WT-MM | NOTEST | 5.8944 | 11.9893 | 1.02433 | 0 | 1 | 1 |
| proX | amino acid ABC transporter permease | chromosome:1739527-1740382 | 1448A-rhpS-MM | 1448A-WT-MM | OK | 45.3519 | 92.1192 | 1.02234 | 1.00479 | 0.1435 | 0.494043 |
| PSPPH_2818 | acetyltransferase | chromosome:3263635-3264094 | 1448A-rhpS-MM | 1448A-WT-MM | NOTEST | 20.5737 | 41.7813 | 1.02206 | 0 | 1 | 1 |
| PSPPH_0609 | hypothetical protein | chromosome:718228-719993 | 1448A-rhpS-MM | 1448A-WT-MM | OK | 88.9404 | 180.468 | 1.02083 | 1.37973 | 0.0579 | 0.341779 |
| scrY | sucrose porin | chromosome:5887227-5888805 | 1448A-rhpS-MM | 1448A-WT-MM | OK | 635.967 | 1290.14 | 1.0205 | 3.35736 | 5.00E-05 | 0.00230303 |
| PSPPH_2104 | transporter | chromosome:2462895-2464224 | 1448A-rhpS-MM | 1448A-WT-MM | OK | 88.1415 | 178.753 | 1.02008 | 1.77845 | 0.0168 | 0.184375 |
| PSPPH_0134 | hypothetical protein | chromosome:158396-158930 | 1448A-rhpS-MM | 1448A-WT-MM | OK | 368.065 | 746.406 | 1.02 | 2.01066 | 0.0096 | 0.132321 |
| PSPPH_1252 | hypothetical protein | chromosome:1459470-1460318 | 1448A-rhpS-MM | 1448A-WT-MM | NOTEST | 24.2853 | 49.2463 | 1.01993 | 0 | 1 | 1 |
| PSPPH_3279 | phenazine biosynthesis family protein | chromosome:3800717-3801551 | 1448A-rhpS-MM | 1448A-WT-MM | OK | 76.8185 | 155.766 | 1.01986 | 1.33199 | 0.0675 | 0.371617 |
| PSPPH_4729 | ABC transporter substrate-binding protein | chromosome:5363953-5368805 | 1448A-rhpS-MM | 1448A-WT-MM | NOTEST | 22.2488 | 45.0298 | 1.01715 | 0 | 1 | 1 |
| arsC | arsenate reductase | chromosome:4271749-4272711 | 1448A-rhpS-MM | 1448A-WT-MM | OK | 105.241 | 212.795 | 1.01577 | 0.744821 | 0.2476 | 0.597683 |
| PSPPH_1670 | AraC family transcriptional regulator | chromosome:1931323-1932364 | 1448A-rhpS-MM | 1448A-WT-MM | OK | 42.7838 | 86.5053 | 1.01572 | 1.12971 | 0.10395 | 0.444275 |
| PSPPH_1097 | AraC family transcriptional regulator | chromosome:1290703-1291900 | 1448A-rhpS-MM | 1448A-WT-MM | OK | 38.7609 | 78.3562 | 1.01545 | 0.953537 | 0.1712 | 0.51683 |
| PSPPH_2346 | amino acid ABC transporter permease | chromosome:2721085-2722838 | 1448A-rhpS-MM | 1448A-WT-MM | NOTEST | 7.57581 | 15.2791 | 1.01209 | 0 | 1 | 1 |
| PSPPH_3620 | hypothetical protein | chromosome:4167929-4168352 | 1448A-rhpS-MM | 1448A-WT-MM | OK | 148.083 | 298.089 | 1.00934 | 1.08572 | 0.11415 | 0.454805 |
| PSPPH_4261 | OprD family outer membrane porin | chromosome:4864067-4865483 | 1448A-rhpS-MM | 1448A-WT-MM | OK | 571.294 | 1148.13 | 1.00698 | 3.11528 | 5.00E-05 | 0.00230303 |
| PSPPH_0136 | hypothetical protein | chromosome:160912-161734 | 1448A-rhpS-MM | 1448A-WT-MM | NOTEST | 17.4885 | 35.1413 | 1.00676 | 0 | 1 | 1 |
| avrRps4 | type III effector AvrRps4 | large_plasmid:75989-76655 | 1448A-rhpS-MM | 1448A-WT-MM | OK | 1238.11 | 2482.88 | 1.00387 | 3.05788 | 5.00E-05 | 0.00230303 |
| hopQ1 | type III effector HopQ1 | large_plasmid:9296-10738 | 1448A-rhpS-MM | 1448A-WT-MM | OK | 305.413 | 611.89 | 1.00251 | 2.63639 | 0.0003 | 0.0108571 |
| dinP | DNA polymerase IV | chromosome:4335175-4336237 | 1448A-rhpS-MM | 1448A-WT-MM | NOTEST | 26.9204 | 53.9146 | 1.00198 | 0 | 1 | 1 |
| PSPPH_0697 | MutT/nudix family protein | chromosome:818823-819228 | 1448A-rhpS-MM | 1448A-WT-MM | NOTEST | 19.6753 | 39.383 | 1.00118 | 0 | 1 | 1 |
| PSPPH_2491 | LysR family transcriptional regulator | chromosome:2877507-2878413 | 1448A-rhpS-MM | 1448A-WT-MM | NOTEST | 33.9545 | 67.9161 | 1.00015 | 0 | 1 | 1 |

**E. List of genes upregulated in *rhpRS* mutant in KB**

| gene | Annotation | locus | sample_1 | sample_2 | status | value_1 | value_2 | log2(fold_change) | test_stat | p_value | q_value |
| --- | --- | --- | --- | --- | --- | --- | --- | --- | --- | --- | --- |
| PSPPH_2067 | RNA polymerase sigma factor SigX | chromosome:2422642-2423140 | 1448A-rhpRS-KB | 1448A-WT-KB | OK | 1377.09 | 687.716 | -1.00174 | -2.76904 | 0.0001 | 0.00174292 |
| PSPPH_2370 | branched-chain amino acid ABC transporter permease | chromosome:2747694-2748762 | 1448A-rhpRS-KB | 1448A-WT-KB | NOTEST | 18.2665 | 9.11513 | -1.00286 | 0 | 1 | 1 |
| PSPPH_3082 | sulfur transfer complex subunit TusD | chromosome:3580094-3580487 | 1448A-rhpRS-KB | 1448A-WT-KB | OK | 173.061 | 86.3459 | -1.00308 | -1.15803 | 0.1074 | 0.298706 |
| PSPPH_4324 | RtrR protein | chromosome:4938654-4938885 | 1448A-rhpRS-KB | 1448A-WT-KB | OK | 310.223 | 154.775 | -1.00313 | -0.868237 | 0.19915 | 0.431629 |
| PSPPH_1784 | hypothetical protein | chromosome:2090031-2090826 | 1448A-rhpRS-KB | 1448A-WT-KB | OK | 44.8717 | 22.3735 | -1.00401 | -0.964231 | 0.1576 | 0.378439 |
| PSPPH_3226 | group 1 glycosyl transferase | chromosome:3737356-3740526 | 1448A-rhpRS-KB | 1448A-WT-KB | OK | 93.4978 | 46.6027 | -1.00452 | -1.4004 | 0.0582 | 0.202538 |
| PSPPH_2452 | multidrug transporter | chromosome:2833979-2835539 | 1448A-rhpRS-KB | 1448A-WT-KB | NOTEST | 20.419 | 10.1663 | -1.00611 | 0 | 1 | 1 |
| PSPPH_1104 | HlyD family secretion protein | chromosome:1295168-1299907 | 1448A-rhpRS-KB | 1448A-WT-KB | OK | 40.7361 | 20.2808 | -1.00619 | -0.6608 | 0.3484 | 0.582095 |
| PSPPH_1347 | hypothetical protein | chromosome:1563499-1564242 | 1448A-rhpRS-KB | 1448A-WT-KB | OK | 341.331 | 169.847 | -1.00694 | -0.768444 | 0.2949 | 0.535119 |
| PSPPH_5123 | phosphate ABC transporter substrate-binding protein | chromosome:5811837-5812836 | 1448A-rhpRS-KB | 1448A-WT-KB | OK | 46.2098 | 22.9692 | -1.0085 | -1.1503 | 0.09885 | 0.283601 |
| PSPPH_1792 | exonuclease | chromosome:2097921-2098464 | 1448A-rhpRS-KB | 1448A-WT-KB | OK | 176.59 | 87.7526 | -1.00889 | -1.47942 | 0.04395 | 0.170067 |
| PSPPH_2524 | type III secretion component | chromosome:2911837-2912449 | 1448A-rhpRS-KB | 1448A-WT-KB | NOTEST | 23.3414 | 11.5758 | -1.01178 | 0 | 1 | 1 |
| flhB | flagellar biosynthesis protein FlhB | chromosome:3896611-3897748 | 1448A-rhpRS-KB | 1448A-WT-KB | OK | 43.1931 | 21.3851 | -1.01419 | -1.14646 | 0.1032 | 0.290697 |
| hrcQb | type III secretion component protein HrcQb | chromosome:1497756-1499418 | 1448A-rhpRS-KB | 1448A-WT-KB | OK | 34.0812 | 16.838 | -1.01726 | -0.425911 | 0.4189 | 0.636408 |
| PSPPH_3861 | hypothetical protein | chromosome:4416378-4416690 | 1448A-rhpRS-KB | 1448A-WT-KB | OK | 650.794 | 321.114 | -1.01912 | -1.72657 | 0.02105 | 0.105326 |
| PSPPH_0454 | gluconate transporter family protein | chromosome:516949-518407 | 1448A-rhpRS-KB | 1448A-WT-KB | NOTEST | 19.1897 | 9.46621 | -1.01947 | 0 | 1 | 1 |
| PSPPH_4713 | diguanylate cyclase | chromosome:5349076-5350375 | 1448A-rhpRS-KB | 1448A-WT-KB | NOTEST | 12.9114 | 6.36714 | -1.01993 | 0 | 1 | 1 |
| PSPPH_2276 | amino acid ABC transporter substrate-binding protein | chromosome:2641714-2643155 | 1448A-rhpRS-KB | 1448A-WT-KB | OK | 35.3793 | 17.4309 | -1.02126 | -0.75171 | 0.3792 | 0.60797 |
| PSPPH_4409 | iojap domain-containing protein | chromosome:5032852-5033239 | 1448A-rhpRS-KB | 1448A-WT-KB | OK | 552.405 | 271.998 | -1.02213 | -1.87388 | 0.01135 | 0.0687077 |
| trkH | potassium uptake protein TrkH | chromosome:4086913-4088368 | 1448A-rhpRS-KB | 1448A-WT-KB | OK | 65.1979 | 32.1022 | -1.02215 | -1.62589 | 0.027 | 0.123164 |
| glnL | nitrogen regulation protein NR(II) | chromosome:5512814-5515333 | 1448A-rhpRS-KB | 1448A-WT-KB | OK | 87.3874 | 43.0043 | -1.02295 | -1.10821 | 0.1185 | 0.318155 |
| PSPPH_2843 | hypothetical protein | chromosome:3289834-3290393 | 1448A-rhpRS-KB | 1448A-WT-KB | OK | 124.108 | 60.9954 | -1.02482 | -1.08002 | 0.12165 | 0.322882 |
| secG | preprotein translocase subunit SecG | chromosome:4781057-4781438 | 1448A-rhpRS-KB | 1448A-WT-KB | OK | 1340.29 | 658.191 | -1.02597 | -2.4801 | 0.00185 | 0.0179133 |
| PSPPH_1466 | hypothetical protein | chromosome:1708904-1709471 | 1448A-rhpRS-KB | 1448A-WT-KB | OK | 166.389 | 81.6361 | -1.02728 | -1.52118 | 0.03795 | 0.153525 |
| dadA | D-amino acid dehydrogenase small subunit | chromosome:260782-262409 | 1448A-rhpRS-KB | 1448A-WT-KB | OK | 1883.23 | 923.33 | -1.0283 | -3.58142 | 0.00005 | 0.000991232 |
| PSPPH_2207 | hypothetical protein | chromosome:2563468-2564347 | 1448A-rhpRS-KB | 1448A-WT-KB | OK | 59.8195 | 29.3285 | -1.02831 | -1.20568 | 0.08665 | 0.260573 |
| PSPPH_2697 | peptide ABC transporter ATP-binding protein | chromosome:3124712-3126699 | 1448A-rhpRS-KB | 1448A-WT-KB | OK | 45.3177 | 22.2087 | -1.02895 | -1.04938 | 0.15215 | 0.370888 |
| PSPPH_1375 | hypothetical protein | chromosome:1596629-1596812 | 1448A-rhpRS-KB | 1448A-WT-KB | OK | 1121.36 | 549.05 | -1.03024 | -1.19034 | 0.10405 | 0.292108 |
| mraW | S-adenosyl-methyltransferase MraW | chromosome:4697111-4703978 | 1448A-rhpRS-KB | 1448A-WT-KB | OK | 517.935 | 253.569 | -1.03039 | -1.56203 | 0.03155 | 0.137473 |
| PSPPH_5003 | hypothetical protein | chromosome:5672266-5672596 | 1448A-rhpRS-KB | 1448A-WT-KB | OK | 2257.3 | 1104.2 | -1.0316 | -2.76189 | 0.00045 | 0.0060721 |
| PSPPH_3222 | capsular polysaccharide biosynthesis protein | chromosome:3733534-3736428 | 1448A-rhpRS-KB | 1448A-WT-KB | OK | 37.7756 | 18.473 | -1.03204 | -0.890387 | 0.27795 | 0.517386 |
| PSPPH_4236 | peptide ABC transporter permease | chromosome:4830473-4834247 | 1448A-rhpRS-KB | 1448A-WT-KB | OK | 36.7682 | 17.9239 | -1.03657 | -0.588028 | 0.43195 | 0.647151 |
| PSPPH_4211 | hypothetical protein | chromosome:4800903-4803056 | 1448A-rhpRS-KB | 1448A-WT-KB | OK | 68.385 | 33.3141 | -1.03755 | -0.488091 | 0.5495 | 0.740276 |
| PSPPH_3150 | capsular polysaccharide biosynthesis protein | chromosome:3654028-3655438 | 1448A-rhpRS-KB | 1448A-WT-KB | NOTEST | 16.9415 | 8.24115 | -1.03964 | 0 | 1 | 1 |
| PSPPH_A0085 | transposase, truncated | large_plasmid:74168-74843 | 1448A-rhpRS-KB | 1448A-WT-KB | OK | 230.515 | 112.067 | -1.0405 | -1.89246 | 0.0106 | 0.0659818 |
| PSPPH_2515 | hypothetical protein | chromosome:2901024-2904519 | 1448A-rhpRS-KB | 1448A-WT-KB | OK | 44.4712 | 21.6128 | -1.04099 | -2.06684 | 0.00555 | 0.0402351 |
| PSPPH_0285 | amino acid ABC transporter substrate-binding protein | chromosome:328206-329169 | 1448A-rhpRS-KB | 1448A-WT-KB | OK | 42.4441 | 20.5476 | -1.0466 | -1.13244 | 0.11105 | 0.304542 |
| PSPPH_4495 | TetR family transcriptional regulator | chromosome:5131971-5132604 | 1448A-rhpRS-KB | 1448A-WT-KB | OK | 168.924 | 81.7707 | -1.04672 | -1.6335 | 0.0275 | 0.124225 |
| PSPPH_3862 | hypothetical protein | chromosome:4416927-4417653 | 1448A-rhpRS-KB | 1448A-WT-KB | OK | 328.782 | 159.094 | -1.04725 | -2.1656 | 0.0043 | 0.032943 |
| PSPPH_2835 | ABC transporter permease | chromosome:3281750-3282545 | 1448A-rhpRS-KB | 1448A-WT-KB | NOTEST | 25.4388 | 12.3092 | -1.04729 | 0 | 1 | 1 |
| rimO | 30S ribosomal protein S12 methylthiotransferase | chromosome:4345005-4346349 | 1448A-rhpRS-KB | 1448A-WT-KB | OK | 187.797 | 90.7916 | -1.04854 | -2.3779 | 0.00185 | 0.0179133 |
| PSPPH_5204 | DeoR family transcriptional regulator | chromosome:5908357-5909125 | 1448A-rhpRS-KB | 1448A-WT-KB | OK | 72.9807 | 35.2696 | -1.04909 | -1.22746 | 0.09295 | 0.273379 |
| PSPPH_1464 | lipoprotein | chromosome:1707098-1707962 | 1448A-rhpRS-KB | 1448A-WT-KB | OK | 114.474 | 55.3147 | -1.04929 | -1.66461 | 0.0252 | 0.118707 |
| PSPPH_0629 | protein kinase | chromosome:744515-746438 | 1448A-rhpRS-KB | 1448A-WT-KB | OK | 1040.38 | 502.68 | -1.0494 | -3.86867 | 0.00005 | 0.000991232 |
| hemN | coproporphyrinogen III oxidase | chromosome:3877632-3879015 | 1448A-rhpRS-KB | 1448A-WT-KB | OK | 770.193 | 371.989 | -1.04996 | -3.4385 | 0.00005 | 0.000991232 |
| PSPPH_0891 | methyl-accepting chemotaxis protein | chromosome:1062848-1064525 | 1448A-rhpRS-KB | 1448A-WT-KB | OK | 79.9579 | 38.6136 | -1.05013 | -1.96829 | 0.0096 | 0.0614959 |
| PSPPH_1594 | ABC transporter substrate-binding protein | chromosome:1848100-1849168 | 1448A-rhpRS-KB | 1448A-WT-KB | OK | 166.182 | 80.1759 | -1.05152 | -2.15388 | 0.00455 | 0.0344171 |
| flgA | flagellar basal body P-ring biosynthesis protein FlgA | chromosome:3946916-3947675 | 1448A-rhpRS-KB | 1448A-WT-KB | OK | 33.4995 | 16.1604 | -1.05168 | -0.803485 | 0.2433 | 0.483219 |
| PSPPH_4084 | hypothetical protein | chromosome:4666249-4666582 | 1448A-rhpRS-KB | 1448A-WT-KB | OK | 99.4511 | 47.9713 | -1.05181 | -0.735974 | 0.28295 | 0.523476 |
| accA | acetyl-CoA carboxylase carboxyltransferase subunit alpha | chromosome:4376285-4377233 | 1448A-rhpRS-KB | 1448A-WT-KB | OK | 1154.6 | 556.88 | -1.05196 | -3.37069 | 0.00005 | 0.000991232 |
| PSPPH_0757 | hypothetical protein | chromosome:887335-888834 | 1448A-rhpRS-KB | 1448A-WT-KB | OK | 198.308 | 95.5873 | -1.05285 | -1.40228 | 0.05575 | 0.197965 |
| PSPPH_2946 | methyl-accepting chemotaxis protein | chromosome:3417814-3419611 | 1448A-rhpRS-KB | 1448A-WT-KB | OK | 48.8316 | 23.5365 | -1.05292 | -1.68396 | 0.01995 | 0.101769 |
| PSPPH_2969 | hypothetical protein | chromosome:3446582-3446981 | 1448A-rhpRS-KB | 1448A-WT-KB | OK | 108.924 | 52.385 | -1.05609 | -0.964493 | 0.17745 | 0.405171 |
| PSPPH_2443 | hypothetical protein | chromosome:2824041-2825234 | 1448A-rhpRS-KB | 1448A-WT-KB | OK | 92.2449 | 44.341 | -1.05683 | -0.908412 | 0.26995 | 0.508878 |
| nrdR | transcriptional regulator NrdR | chromosome:5141467-5141932 | 1448A-rhpRS-KB | 1448A-WT-KB | OK | 329 | 158.11 | -1.05716 | -1.78547 | 0.01745 | 0.0926312 |
| PSPPH_2849 | TonB-dependent receptor | chromosome:3292632-3294933 | 1448A-rhpRS-KB | 1448A-WT-KB | OK | 69.7192 | 33.5011 | -1.05735 | -2.13964 | 0.00505 | 0.0374542 |
| PSPPH_4254 | ABC transporter ATP-binding protein | chromosome:4853807-4855541 | 1448A-rhpRS-KB | 1448A-WT-KB | OK | 130.705 | 62.7914 | -1.05768 | -2.30024 | 0.0017 | 0.0169312 |
| PSPPH_3894 | hypothetical protein | chromosome:4452549-4452843 | 1448A-rhpRS-KB | 1448A-WT-KB | OK | 548.597 | 263.393 | -1.05853 | -1.56472 | 0.03315 | 0.141786 |
| PSPPH_3741 | phage integrase | chromosome:4290445-4291432 | 1448A-rhpRS-KB | 1448A-WT-KB | OK | 49.8053 | 23.9005 | -1.05926 | -1.23437 | 0.09315 | 0.273436 |
| recD | exodeoxyribonuclease V subunit alpha | chromosome:809447-818703 | 1448A-rhpRS-KB | 1448A-WT-KB | OK | 78.0772 | 37.4494 | -1.05996 | -1.46503 | 0.04525 | 0.173175 |
| PSPPH_2027 | CHAD domain-containing superfamily | chromosome:2380994-2381756 | 1448A-rhpRS-KB | 1448A-WT-KB | OK | 187.135 | 89.551 | -1.0633 | -1.935 | 0.01115 | 0.0681878 |
| PSPPH_5042 | AsnC family transcriptional regulator | chromosome:5713736-5714225 | 1448A-rhpRS-KB | 1448A-WT-KB | OK | 225.946 | 108.109 | -1.06349 | -1.62238 | 0.0276 | 0.124542 |
| PSPPH_4673 | Cro/CI family transcriptional regulator | chromosome:5310338-5311070 | 1448A-rhpRS-KB | 1448A-WT-KB | NOTEST | 13.9755 | 6.68308 | -1.06432 | 0 | 1 | 1 |
| PSPPH_0816 | zinc-binding protein | chromosome:977779-977977 | 1448A-rhpRS-KB | 1448A-WT-KB | OK | 242.972 | 116.098 | -1.06544 | -0.63287 | 0.355 | 0.586264 |
| PSPPH_1807 | hypothetical protein | chromosome:2110653-2110971 | 1448A-rhpRS-KB | 1448A-WT-KB | OK | 312.212 | 149.056 | -1.06667 | -1.31339 | 0.0801 | 0.24795 |
| PSPPH_1204 | hypothetical protein | chromosome:1415326-1415608 | 1448A-rhpRS-KB | 1448A-WT-KB | OK | 373.131 | 177.857 | -1.06896 | -1.26996 | 0.07615 | 0.239861 |
| PSPPH_4068 | lipoprotein | chromosome:4650742-4652176 | 1448A-rhpRS-KB | 1448A-WT-KB | OK | 80.4001 | 38.3177 | -1.06919 | -1.9052 | 0.01245 | 0.0730412 |
| PSPPH_3427 | sensor histidine kinase | chromosome:3961127-3963421 | 1448A-rhpRS-KB | 1448A-WT-KB | OK | 71.9601 | 34.2693 | -1.07028 | -0.85811 | 0.2685 | 0.507517 |
| PSPPH_1967 | lipoprotein | chromosome:2315642-2316808 | 1448A-rhpRS-KB | 1448A-WT-KB | OK | 1582.59 | 753.395 | -1.07081 | -1.98079 | 0.00715 | 0.04895 |
| PSPPH_2685 | polysaccharide deacetylase | chromosome:3108978-3109758 | 1448A-rhpRS-KB | 1448A-WT-KB | OK | 82.1293 | 39.0904 | -1.07108 | -1.3866 | 0.0606 | 0.208805 |
| PSPPH_0607 | hypothetical protein | chromosome:717657-717912 | 1448A-rhpRS-KB | 1448A-WT-KB | OK | 659.729 | 313.775 | -1.07214 | -1.48972 | 0.04125 | 0.162782 |
| PSPPH_2707 | ornithine aminotransferase | chromosome:3132422-3135360 | 1448A-rhpRS-KB | 1448A-WT-KB | OK | 34.3219 | 16.3214 | -1.07236 | -1.12368 | 0.1498 | 0.368596 |
| PSPPH_1903 | universal stress protein family protein | chromosome:2208996-2209428 | 1448A-rhpRS-KB | 1448A-WT-KB | OK | 1763 | 837.887 | -1.07321 | -2.95807 | 0.00005 | 0.000991232 |
| PSPPH_3933 | major facilitator family transporter | chromosome:4493388-4494729 | 1448A-rhpRS-KB | 1448A-WT-KB | OK | 130.018 | 61.5884 | -1.07799 | -2.12009 | 0.0045 | 0.0341624 |
| PSPPH_3030 | lipoprotein | chromosome:3518893-3519664 | 1448A-rhpRS-KB | 1448A-WT-KB | OK | 32.4906 | 15.3885 | -1.07817 | -0.855891 | 0.222 | 0.459944 |
| PSPPH_3459 | hypothetical protein | chromosome:3996811-3998140 | 1448A-rhpRS-KB | 1448A-WT-KB | NOTEST | 28.6924 | 13.5782 | -1.07937 | 0 | 1 | 1 |
| PSPPH_5184 | iron-compound ABC transporter ATP-binding protein | chromosome:5882210-5885134 | 1448A-rhpRS-KB | 1448A-WT-KB | OK | 51.8836 | 24.5387 | -1.08022 | -0.899454 | 0.26905 | 0.508098 |
| aroH | chorismate mutase | chromosome:5510729-5511287 | 1448A-rhpRS-KB | 1448A-WT-KB | OK | 63.6411 | 30.0758 | -1.08135 | -0.993084 | 0.17595 | 0.404913 |
| arnC | glycosyl transferase ArnC | chromosome:3246757-3252777 | 1448A-rhpRS-KB | 1448A-WT-KB | NOTEST | 22.6756 | 10.7152 | -1.08149 | 0 | 1 | 1 |
| PSPPH_5004 | HAD family hydrolase | chromosome:5672670-5674255 | 1448A-rhpRS-KB | 1448A-WT-KB | OK | 270.559 | 127.752 | -1.08259 | -1.78175 | 0.01785 | 0.0938022 |
| PSPPH_1092 | sigma factor regulatory protein FecR/PupR family | chromosome:1287130-1288608 | 1448A-rhpRS-KB | 1448A-WT-KB | OK | 44.2181 | 20.8675 | -1.08338 | -1.12048 | 0.1183 | 0.318155 |
| PSPPH_4053 | hypothetical protein | chromosome:4633842-4637746 | 1448A-rhpRS-KB | 1448A-WT-KB | NOTEST | 17.2646 | 8.13457 | -1.08568 | 0 | 1 | 1 |
| PSPPH_3934 | polysaccharide deacetylase | chromosome:4494756-4495638 | 1448A-rhpRS-KB | 1448A-WT-KB | OK | 211.004 | 99.3734 | -1.08634 | -2.2303 | 0.0027 | 0.0235294 |
| PSPPH_4744 | AraC family transcriptional regulator | chromosome:5386238-5387342 | 1448A-rhpRS-KB | 1448A-WT-KB | OK | 62.497 | 29.4244 | -1.08677 | -1.43935 | 0.0474 | 0.178465 |
| PSPPH_2626 | gamma-carboxygeranoyl-CoA hydratase | chromosome:3030198-3031059 | 1448A-rhpRS-KB | 1448A-WT-KB | OK | 173.702 | 81.721 | -1.08783 | -1.98031 | 0.00865 | 0.056981 |
| PSPPH_1593 | hypothetical protein | chromosome:1845592-1848022 | 1448A-rhpRS-KB | 1448A-WT-KB | OK | 51.9816 | 24.4536 | -1.08795 | -1.05387 | 0.15155 | 0.370344 |
| PSPPH_4054 | acyltransferase domain-containing protein | chromosome:4633842-4637746 | 1448A-rhpRS-KB | 1448A-WT-KB | OK | 47.5403 | 22.3549 | -1.08856 | -0.879009 | 0.22435 | 0.462978 |
| rstA | DNA-binding response regulator RstA | chromosome:4284454-4285192 | 1448A-rhpRS-KB | 1448A-WT-KB | OK | 148.969 | 70.0314 | -1.08894 | -1.73212 | 0.02265 | 0.110943 |
| PSPPH_0407 | gluconate transporter family protein | chromosome:470481-471831 | 1448A-rhpRS-KB | 1448A-WT-KB | NOTEST | 25.1564 | 11.8241 | -1.08919 | 0 | 1 | 1 |
| PSPPH_4073 | hypothetical protein | chromosome:4655417-4656149 | 1448A-rhpRS-KB | 1448A-WT-KB | OK | 111.645 | 52.4635 | -1.08953 | -1.50185 | 0.0376 | 0.153051 |
| PSPPH_1203 | hypothetical protein | chromosome:1414291-1415290 | 1448A-rhpRS-KB | 1448A-WT-KB | NOTEST | 26.706 | 12.5495 | -1.08954 | 0 | 1 | 1 |
| PSPPH_4671 | hypothetical protein | chromosome:5308533-5309343 | 1448A-rhpRS-KB | 1448A-WT-KB | OK | 97.0195 | 45.587 | -1.08965 | -1.55675 | 0.0344 | 0.145349 |
| PSPPH_2826 | FKBP-type peptidylprolyl isomerase | chromosome:3272390-3272810 | 1448A-rhpRS-KB | 1448A-WT-KB | OK | 400.008 | 187.664 | -1.09188 | -1.887 | 0.01135 | 0.0687077 |
| PSPPH_1205 | hypothetical protein | chromosome:1415626-1416433 | 1448A-rhpRS-KB | 1448A-WT-KB | OK | 162.526 | 76.2183 | -1.09246 | -1.89356 | 0.01065 | 0.0661946 |
| PSPPH_3409 | hypothetical protein | chromosome:3943000-3943474 | 1448A-rhpRS-KB | 1448A-WT-KB | OK | 452.349 | 211.965 | -1.09361 | -2.11983 | 0.00555 | 0.0402351 |
| tpiA | triosephosphate isomerase | chromosome:4781443-4782199 | 1448A-rhpRS-KB | 1448A-WT-KB | OK | 1771.11 | 829.618 | -1.09414 | -3.57439 | 0.00005 | 0.000991232 |
| PSPPH_2160 | hypothetical protein | chromosome:2513941-2517414 | 1448A-rhpRS-KB | 1448A-WT-KB | OK | 127.775 | 59.7893 | -1.09565 | -1.21561 | 0.09015 | 0.268206 |
| PSPPH_2971 | methyl-accepting chemotaxis transducer/sensory box protein | chromosome:3448737-3450894 | 1448A-rhpRS-KB | 1448A-WT-KB | OK | 204.284 | 95.425 | -1.09814 | -2.97178 | 0.0002 | 0.00308708 |
| PSPPH_2728 | ribose ABC transporter ATP-binding protein | chromosome:3153737-3155621 | 1448A-rhpRS-KB | 1448A-WT-KB | NOTEST | 24.3898 | 11.3847 | -1.09918 | 0 | 1 | 1 |
| PSPPH_1835 | hypothetical protein | chromosome:2135176-2135470 | 1448A-rhpRS-KB | 1448A-WT-KB | OK | 468.041 | 218.143 | -1.10136 | -1.50836 | 0.0389 | 0.155861 |
| ftsL | cell division protein FtsL | chromosome:4697111-4703978 | 1448A-rhpRS-KB | 1448A-WT-KB | OK | 538.065 | 250.48 | -1.10308 | -0.670808 | 0.35025 | 0.58387 |
| PSPPH_2240 | hypothetical protein | chromosome:2597977-2599996 | 1448A-rhpRS-KB | 1448A-WT-KB | NOTEST | 24.3196 | 11.3168 | -1.10364 | 0 | 1 | 1 |
| ampD | N-acetyl-anhydromuranmyl-L-alanine amidase | chromosome:1007259-1007808 | 1448A-rhpRS-KB | 1448A-WT-KB | OK | 290.479 | 135.145 | -1.10392 | -1.91205 | 0.01135 | 0.0687077 |
| PSPPH_3321 | endoribonuclease L-PSP | chromosome:3841513-3843029 | 1448A-rhpRS-KB | 1448A-WT-KB | NOTEST | 25.6894 | 11.9439 | -1.1049 | 0 | 1 | 1 |
| PSPPH_4480 | hypothetical protein | chromosome:5116578-5116833 | 1448A-rhpRS-KB | 1448A-WT-KB | OK | 243.368 | 113.091 | -1.10565 | -0.927404 | 0.1842 | 0.413478 |
| PSPPH_0905 | diguanylate cyclase | chromosome:1077997-1079101 | 1448A-rhpRS-KB | 1448A-WT-KB | OK | 33.7683 | 15.6777 | -1.10696 | -1.0515 | 0.1399 | 0.351895 |
| ssuC | aliphatic sulfonate ABC transporter permease | chromosome:5576356-5577963 | 1448A-rhpRS-KB | 1448A-WT-KB | NOTEST | 20.181 | 9.36352 | -1.10787 | 0 | 1 | 1 |
| PSPPH_2747 | ECF subfamily RNA polymerase sigma factor | chromosome:3179069-3180571 | 1448A-rhpRS-KB | 1448A-WT-KB | OK | 30.595 | 14.185 | -1.10893 | -0.510833 | 0.46205 | 0.67247 |
| PSPPH_4910 | taurine ABC transporter permease | chromosome:5583347-5585005 | 1448A-rhpRS-KB | 1448A-WT-KB | OK | 33.3863 | 15.4757 | -1.10925 | -0.692873 | 0.2391 | 0.479231 |
[truncated: 344,804 more chars]
